# Ferric carboxymaltose for anemia in late pregnancy: a randomized controlled trial

**DOI:** 10.1038/s41591-024-03385-w

**Published:** 2025-01-06

**Authors:** Sant-Rayn Pasricha, Ernest Moya, Ricardo Ataíde, Glory Mzembe, Rebecca Harding, Martin N. Mwangi, Truwah Zinenani, Khic-Houy Prang, Justina Kaunda, Owen P. L. Mtambo, Maclean Vokhiwa, Gomezgani Mhango, Elisabeth Mamani-Mategula, Katherine Fielding, Ayşe Demir, Naomi Von Dinklage, Hans Verhoef, Alistair RD McLean, Lucinda Manda-Taylor, Sabine Braat, Kamija S. Phiri

**Affiliations:** 1 Walter and Eliza Hall Institute of Medical Research, Parkville, Victoria Australia; 2 Diagnostic Haematology, The Royal Melbourne Hospital, Parkville, Victoria Australia; 3 Clinical Haematology, The Peter MacCallum Cancer Centre and The Royal Melbourne Hospital, Parkville, Victoria Australia; 4 Department of Medical Biology, The University of Melbourne, Parkville, Victoria Australia; 5 Training and Research Unit of Excellence (TRUE), Blantyre, Malawi; 6 Department of Public Health, School of Public and Global Health, Kamuzu University of Health Sciences, Blantyre, Malawi; 7 Department of Infectious Diseases at the Peter Doherty Institute, The University of Melbourne, Melbourne, Victoria Australia; 8 The Micronutrient Forum, Healthy Mothers Healthy Babies Consortium, Washington, DC USA; 9 Centre for Health Policy, Melbourne School of Population and Global Health, The University of Melbourne, Parkville, Victoria Australia; 10 Division of Human Nutrition and Health, Wageningen University, Wageningen, The Netherlands

**Keywords:** Anaemia, Randomized controlled trials, Reproductive signs and symptoms

## Abstract

Over 46% of African pregnant women are anemic. Oral iron is recommended but often suboptimal, particularly late in pregnancy. Intravenous ferric carboxymaltose (FCM) could treat anemia in women in the third trimester in sub-Saharan Africa. In an open-label, individually randomized trial in antenatal clinics in southern Malawi, we randomized 590 women at 27–35 weeks of gestation with capillary hemoglobin <10.0 g dl^−1^ to FCM (20 mg kg^−1^ up to 1,000 mg, once at enrollment) or standard of care (60 mg elemental iron, twice daily for 90 days). Participants and their infants were followed to 4 weeks postpartum. Primary outcomes were maternal anemia at 36 weeks’ gestation or delivery (whichever occurred first) and neonatal birthweight. At the primary timepoint, 126 of 270 (46.7%) of women in the FCM group were anemic, compared to 170 of 271 (67.3%) women in the standard-of-care group (PR, 0.74 (95% CI 0.64, 0.87); *P* = 0.0002). There was no difference between groups in birthweight (mean difference 10.9 g (−65.7, 87.5 g); *P* = 0.78). No serious infusion-related reactions occurred, and there were no differences in adverse events between groups. In Malawian women in late pregnancy, FCM effectively and safely reduced anemia before childbirth. Australia New Zealand Clinical Trial registration: ANZCTR12621001239853

## Abstract (web summary)

A randomized controlled trial in the third trimester of pregnancy in Malawian women with anemia found a single dose of intravenous ferric carboxymaltose to be more effective than standard of care (that is, twice-daily oral iron) in reducing anemia rates before childbirth.

## Main

The prevalence of anemia in pregnancy is 36.5% globally, including 46.2% in sub-Saharan Africa^1^. Anemia at childbirth is associated with maternal mortality and postpartum hemorrhage^2^ and promotes postpartum anemia. Anemia at childbirth is also associated with adverse outcomes for the newborn, including premature birth and low birthweight^3^. Iron deficiency is a major cause of antenatal anemia^4^. The World Health Organization (WHO) recommends treating antenatal anemia with oral iron supplements (for example, 30–60 mg elemental iron daily)^5,6^. However, by the third trimester of gestation, the available time before onset of labor may be too limited for oral iron interventions to restore hemoglobin concentrations and alleviate anemia. Furthermore, sufficient use of oral iron requires ongoing adherence^7^, but in sub-Saharan Africa, only 29% of pregnant women consume an adequate treatment course of oral iron^8^.

Ferric carboxymaltose (FCM) is an intravenous iron formulation that enables up to 1,000 mg (20 mg kg^−1^) to be administered in a single 15-min infusion^9,10^. FCM is widely used in high-income settings (including in primary care contexts)^11^, often to avoid transfusion in scenarios where rapid increases in hemoglobin are required, including in late pregnancy^12^.

Malawi, in southern sub-Saharan Africa, is a low-income country where pregnant women endure a high burden of anemia^1^, infectious diseases including malaria and HIV^1,13^, limited access to early antenatal care^1,14^ and high risks of perinatal complications including premature delivery, stillbirth, low birthweight and neonatal mortality^1,14^. Sophisticated laboratory testing for anemia and iron deficiency is seldom routinely available. We previously evaluated the efficacy and safety of a single dose of FCM (compared to standard-of-care (SOC) oral iron) to treat moderate or severe anemia in the second trimester of pregnancy in Malawian women (REVAMP)^15^. FCM could be safely administered in outpatient settings by research staff, with no infusion-related serious adverse events. However, FCM (compared to SOC) did not reduce the primary maternal outcome of anemia prevalence at 36 weeks’ gestation or influence birthweight, although FCM did reduce maternal iron deficiency and iron deficiency anemia^15^. Interestingly, women receiving FCM had a lower prevalence of anemia than those receiving SOC at the earliest timepoint 4 weeks after infusion.

We therefore reasoned that in the third trimester, when childbirth is impending, FCM may offer an ideal solution to rapidly optimize hemoglobin concentration and alleviate the risk of anemia. We also considered that FCM could be administered in the Malawian primary health care setting even though infrastructure is basic. We conducted a randomized controlled trial to determine whether, in Malawian women in their third trimester of pregnancy with moderate or severe anemia, a single dose of intravenous FCM (20 mg kg^−1^ up to 1,000 mg) would be superior to SOC (oral iron) in reducing anemia prevalence either at 36 weeks’ gestation or during delivery (whichever came first), would improve other key maternal and neonatal outcomes, and would be safe in terms of infusion-related and other adverse events.

## Results

### Patient disposition

**Fig. 1:**
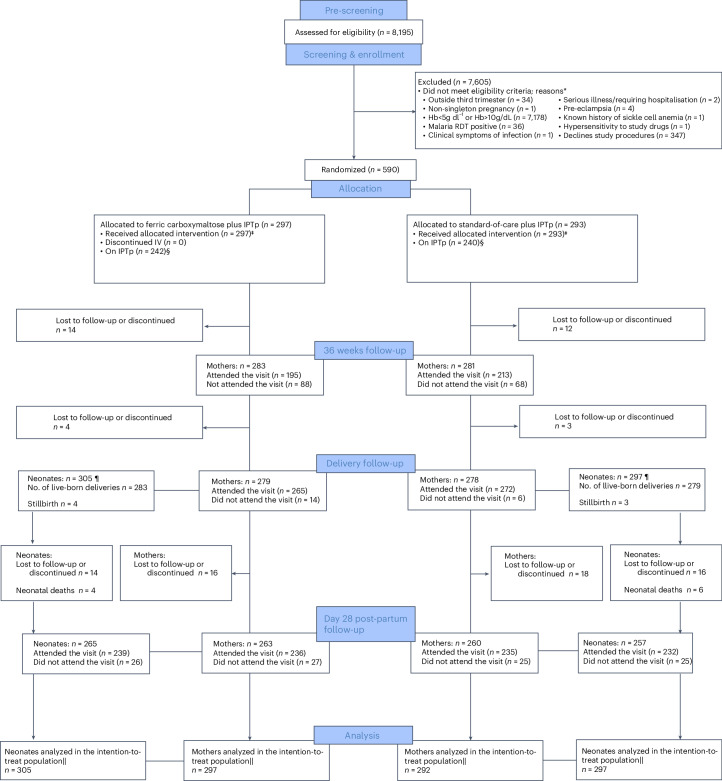
Trial profile with an overview of screening, enrollment and follow-up of participants. Hb, hemoglobin; IV, intravenous; IPTp, intermittent preventative therapy; RDT, rapid diagnostic test. *Reasons for not meeting eligibility criteria were assessed using the questions on the eligibility data collection forms. ‡Reasons for not receiving the treatment were collected on the participant randomization form. §Defined as those who are on IPTp with sulfadoxine–pyrimethamine (SP) at enrollment. Common reasons not to be on IPTp include recent malaria therapy (usually with artemether–lumefantrine) or being HIV positive and receiving cotrimoxazole. ¶A total of 23 randomized women withdrew (this includes the non-pregnant woman) and 10 were lost to-follow-up before delivery, as a result we do not have information on whether they had a live-born baby or not on 18 women in the FCM group and 15 women in the SOC (oral iron) group. In addition, there were 7 stillborn babies. There were 16 twins born in the FCM group and 8 in the SOC (oral iron) group. ||The intention-to-treat basis indicates maternal and neonatal outcomes analyzed according to randomly allocated group of the woman. One woman who was randomized in the SOC (oral iron) but later found to be not pregnant was excluded.

Between 24 November 2021 and 22 February 2023, 8,195 women were screened for eligibility, and 590 were enrolled and randomized; the final 4-week postpartum visit was undertaken on 10 June 2023. Figure 1 presents the participant flow. Among the 7,605 ineligible women, the main reason for exclusion was a hemoglobin concentration outside the eligibility range (94.4%). Among 8,186 women who underwent capillary hemoglobin measurement as part of screening, 34.5% were anemic (mild: 22.5%, moderate: 11.7%, severe: 0.3%). All randomized participants received their allocated treatment. In the FCM arm, 263 out of 297 patients (88.6%) received 1,000 mg, and the remaining 11.4% weighed <50 kg and received a lower dose; 7.7% of doses were fully administered by government health workers under supervision of study staff.

Baseline characteristics of trial participants are presented in Table 1 and Supplementary Table 1. The median gestational age (30 weeks), prevalence of anemia by venous hemoglobin assessment (537/588, 91.3%; noting that screening and eligibility for the trial was based on capillary hemoglobin measurement), HIV positivity (119/589, 20.2%), inflammation (172/571, 30.1%) and the proportion of primigravidae (208/589, 35.3%) were balanced between groups. The prevalence of iron deficiency was lower in the intervention group (198/288, 68.8%) than in the SOC group (210/281, 74.7%).

**Table 1:** Baseline characteristics of the participating pregnant women

	FCM *n* = 297	SOC (oral iron) *n* = 292^a^
Age (years), mean (s.d.)	24.5 (6.4)	24.7 (6.5)
Primigravid, *n* (%)^b^	107 (36.0)	101 (34.6)
Gestational age (weeks), median (IQR)^c^	30.0 (28.0–32.0)	30.0 (28.0–32.0)
Height (cm), mean (s.d.)	157.1 (5.7)	157.0 (5.6)
Weight (kg), mean (s.d.)	58.6 (7.8)	59.5 (8.6)
Body mass index (kg/m^2^), mean (s.d.)^d^	23.7 (3.0)	24.1 (3.0)
Religion, *n* (%)^b^		
None	1 (0.3)	0 (0.0)
Christian	209 (70.4)	203 (69.5)
Muslim	82 (27.6)	83 (28.4)
Other	5 (1.7)	6 (2.1)
Education, *n* (%)^b^		
None	10 (3.4)	8 (2.7)
Lower primary (1–5)	69 (23.2)	72 (24.7)
Upper primary (6–8)	132 (44.4)	113 (38.7)
Lower secondary (1–2)	36 (12.1)	41 (14.0)
Upper secondary (3–4)	47 (15.8)	53 (18.2)
Tertiary	3 (1.0)	5 (1.7)
Marital status, *n* (%)^b^		
Single	29 (9.8)	19 (6.5)
Married	250 (84.2)	253 (86.6)
Widowed	2 (0.7)	1 (0.3)
Divorced/separated	15 (5.1)	18 (6.2)
Other	1 (0.3)	1 (0.3)
Income source, *n* (%)^b^		
None	45 (15.2)	48 (16.4)
Subsistence farming	86 (29.0)	71 (24.3)
Large scale farming	2 (0.7)	1 (0.3)
Employed	19 (6.4)	14 (4.8)
Casual work for wages	78 (26.3)	97 (33.2)
Business	62 (20.9)	58 (19.9)
Other	5 (1.7)	3 (1.0)
HIV positive, *n* (%)^b^	62 (20.9)	57 (19.5)
Malaria RDT positive, *n* (%)^e^	0 (0.0)	0 (0.0)
Capillary Hb <10 g dl^−1^, *n* (%)	297 (100.0)	292 (100.0)
Venous Hb (g dl^−1^), mean (s.d.)^f^	9.32 (1.33)	9.40 (1.25)
Anemia (venous), *n* (%)^f^		
No (Hb ≥11 g dl^−1^)	24 (8.1)	27 (9.2)
Mild (10 g dl^−1^ ≤ Hb < 11 g dl^−1^)	64 (21.6)	61 (20.9)
Moderate (7 g dl^−1^ ≤ Hb < 10 g dl^−1^)	195 (65.9)	194 (66.4)
Severe (Hb <7 g dl^−1^)	13 (4.4)	10 (3.4)
Ferritin (µg liter^−1^), median (IQR)^g^	13.0 (8.5–20.9)	11.7 (8.0–17.0)
CRP (mg liter^−1^), median (IQR)^h^	2.8 (1.6–5.5)	3.0 (1.5–6.1)
Ferritin <15 µg liter^−1^ or ferritin <30 µg liter^−1^ if CRP >5 mg liter^−1,^^i^		
Iron deficient, *n* (%)^i^	198 (68.8)	210 (74.7)
Iron deficient anemia, *n* (%)^i^	185 (64.2)	195 (69.4)
Ferritin <15 µg liter^−1^ or ferritin <70 µg liter^−1^ if CRP >5 mg liter^−1,j^		
Iron deficient, *n* (%)^j^	210 (72.9)	222 (79.0)
Iron deficient anemia, *n* (%)^j^	197 (68.4)	205 (73.0)
Inflammation, *n* (%)^k^	82 (28.4)	90 (31.9)
Anemia and inflammation, *n* (%)^k^	75 (26.0)	82 (29.1)

Participants were enrolled across eight primary antenatal clinics; Bimbi (*n* = 46), City (*n* = 58), Domasi (*n* = 89), Lambulira (*n* = 64), Likangala (*n* = 126), Matawale (*n* = 134), Naisi (*n* = 56), Sadzi (*n* = 16). CRP, C-reactive protein; Hb, hemoglobin; HIV, human immunodeficiency virus; IQR, interquartile range (25th to 75th percentile); RDT, rapid diagnostic test; s.d., standard deviation.

^a^One woman who was randomized in the SOC (oral iron) but later found to be not pregnant was excluded.

^b^Religion, education, marital status, income source, gravidity and HIV status were self-reported.

^c^Estimated gestational age (weeks) is dated either using first day of the last menstrual period or fundal height.

^d^Body mass index is the weight in kilograms divided by the square of the height in meters.

^e^Malaria RDT positive was based on confirmatory RDT testing by laboratory personnel on venous blood collected at enrollment.

^f^There is one missing venous Hb value in the FCM group.

^g^There are 9 missing serum ferritin values in the FCM group and 11 in the SOC (oral iron) group.

^h^There are 8 missing CRP values in the FCM group and 10 in the SOC (oral iron) group.

^i^There are 9 missing serum ferritin values in the FCM group and 11 in the SOC (oral iron) group. Iron deficient indicates serum ferritin <15 µg liter^−1^ or serum ferritin <30 µg liter^−1^ if CRP >5 mg liter^−1^, and iron deficient anemia indicates Hb <11 g dl^−1^ and serum ferritin <15 µg liter^−1^ or serum ferritin <30 µg liter^−1^ if CRP >5 mg liter^−1^.

^j^Ferritin<70 μg liter^−1^ in context of inflammation reflects WHO recommendation. There are 9 missing serum ferritin values in the FCM group and 11 in the SOC (oral iron) group. Iron deficient indicates serum ferritin <15 µg liter^−1^ or serum ferritin <70 µg liter^−1^ if CRP >5 mg liter^−1^, and iron deficient anemia indicates Hb <11 g dl^−1^ and serum ferritin <15 µg liter^−1^ or serum ferritin <70 µg liter^−1^ if CRP >5 mg liter^−1^.

^k^There is one missing venous Hb value in the FCM group. There are 8 missing CRP values in the FCM group and 10 in the SOC (oral iron) group. Inflammation indicates CRP >5 mg liter^−1^, and anemia and inflammation indicate Hb <11.0 g dl^−1^ and CRP >5 mg liter^−1^.

A total of 23 randomized women withdrew from the trial, and 10 were lost to follow-up before delivery. Although clinically determined multiple pregnancy was an exclusion criterion, there were 16 twins (8 pairs) born to the FCM group and 8 (4 pairs) to the SOC group. Cesarean deliveries occurred in 26 of 262 patients (9.9%) in the FCM group and 20 out of 269 (7.4%) in the SOC group.

### Primary outcomes

Table 2 presents the effects of FCM compared to SOC on maternal efficacy and neonatal efficacy outcomes. The prevalence of anemia at 36 weeks’ gestation or delivery (whichever came first) was lower among women randomized to the FCM group (126/270, 46.7%) than in those randomized to SOC (170/271, 62.7%; prevalence ratio (PR) 0.74 (95% CI 0.64, 0.87), *P* = 0.0002). The median duration from treatment administration to hemoglobin assessment at the primary outcome timepoint was 55 (FCM arm) and 53 (SOC arm) days. The number of women with moderate or severe anemia needed to treat with FCM (vs SOC) to prevent one case of anemia at the primary outcome timepoint was 6 (95% CI 4, 13). Among those women with anemia at 36 weeks’ gestation, the prevalence of concurrent inflammation (elevated (CRP)) was 33/100 (33.0%) in the FCM group compared to 35/136 (25.7%) in the SOC group.

**Table 2:** Effects of FCM on maternal and neonatal efficacy outcomes

	FCM *n* = 297	SOC (oral iron) *n* = 292^a^	PR, mean difference or geometric mean ratio (95% CI)^b^	*P* value^f^
**Maternal efficacy outcomes**				
**Primary outcome**				
Anemia at 36 weeks’ gestation or at delivery, (whichever came first)^c–e^	126/270 (46.7%)	170/271 (62.7%)	0.74 (0.64, 0.87)	0.0002^g^
**Key secondary outcomes**				
Venous Hb (g dl^−1^) absolute change from baseline				
36 weeks’ gestation or at delivery (whichever came first)^c^	1.75 (1.65)	1.15 (1.38)	0.52 (0.30, 0.73)	<0.0001^f^
1 month postpartum	2.99 (1.64)	2.22 (1.51)	0.67 (0.43, 0.90)	<0.0001^f^
Ferritin (µg liter^−1^) absolute change from baseline				
36 weeks’ gestation or at delivery (whichever came first)^c^	144.6 (70.6–270.7)	10.8 (3.4–25.6)	6.06 (5.23, 7.02)^h^	<0.0001^f^
1 month postpartum	87.4 (48.3–153.9)	17.3 (3.9–42.4)	3.22 (2.78, 3.72)^h^	<0.0001^f^
**Other secondary outcomes**				
Anemia^d^				
Delivery	52/251 (20.7%)	90/258 (34.9%)	0.61 (0.46, 0.81)	–
1 month postpartum	85/236 (36.0%)	136/235 (57.9%)	0.62 (0.51, 0.75)	–
Moderate/severe anemia^d^				
36 weeks’ gestation or at delivery (whichever came first)^c^	53/270 (19.6%)	91/271 (33.6%)	0.60 (0.46, 0.80)	–
Delivery	19/251 (7.6%)	41/258 (15.9%)	0.47 (0.28, 0.78)	–
1 month postpartum	26/236 (11.0%)	54/235 (23.0%)	0.50 (0.33, 0.76)	–
Venous Hb (g dl^−1^) absolute change from baseline				
Delivery	2.59 (1.81)	2.05 (1.86)	0.46 (0.19, 0.73)	–
Ferritin (µg liter^−1^) absolute change from baseline				
Delivery	106.2 (51.0-223.6)	15.0 (5.2-34.2)	4.25 (3.64, 4.95)^h^	–
Iron deficient^i^				
36 weeks’ gestation or at delivery (whichever came first)^c^	6/248 (2.4%)	90/257 (35.0%)	0.08 (0.04, 0.17)	–
Delivery	15/204 (7.4%)	83/223 (37.2%)	0.20 (0.12, 0.34)	–
1 month postpartum	9/218 (4.1%)	57/218 (26.1%)	0.16 (0.08, 0.31)	–
Iron deficient anemia^i^				
36 weeks’ gestation or at delivery (whichever came first)^c^	1/247 (0.4%)	70/255 (27.5%)	0.02 (0.00, 0.13)	–
Delivery	1/201 (0.5%)	36/217 (16.6%)	0.04 (0.01, 0.23)	–
1 month postpartum	8/218 (3.7%)	49/218 (22.5%)	0.17 (0.08, 0.35)	–

	FCM *n* = 305		SOC (oral iron) *n* = 296		PR, mean difference or risk ratio (95% CI)^j^	*P* value^f^
**Neonatal efficacy outcomes**	**Total no**.		**Total no**.			
**Key outcome**						
Birthweight (g)	270	2975.1 (431.4)	273	2958.7 (479.5)	10.9 (−65.7, 87.5)	0.78^g^
**Key secondary outcomes**						
Venous Hb (g dl^−1^) at 1 month of age	181	12.38 (1.57)	175	12.49 (1.61)	−0.10 (−0.44, 0.24)	0.55^f^
Weight (g) at 1 month of age	239	3969.1 (637.7)	232	3942.9 (712.8)	35.5 (−86.0, 156.9)	0.57^f^
**Other secondary outcomes**						
Birth length (cm)	268	47.6 (2.7)	272	47.5 (3.1)	0.004 (−0.49, 0.50)	–
Length (cm)	239	52.0 (2.8)	232	51.6 (3.3)	0.33 (−0.22, 0.89)	–
Low birthweight (<2,500 g)^k^	270	35 (13.0%)	273	39 (14.3%)	0.92 (0.60, 1.41)	–
Stillbirth^l^	305	4 (1.3%)	296	3 (1.0%)	1.29 (0.29, 5.73)	–
Weight for age *z*-score	229	−0.6 (1.1)	229	−0.6 (1.3)	0.04 (−0.19, 0.26)	–
Length for age *z*-score	229	−1.0 (1.4)	229	−1.2 (1.6)	0.15 (−0.13, 0.43)	–
Weight for length *z*-score	226	0.2 (1.7)	224	0.5 (1.9)	−0.23 (−0.56, 0.10)	–

Data are presented as number (percentage) of patients, mean (standard deviation) or median (25^th^ to 75^th^ percentile). Total no. denotes the number of participants in each randomization group with relevant outcome data.

^a^One woman who was randomized in the SOC (oral iron) but later found to be not pregnant was excluded.

^b^A PR and two-sided 95% CI of FCM versus SOC (oral iron) is displayed for anemia, moderate/severe anemia, iron deficient and iron deficient anemia at 36 weeks’ gestation or at delivery (whichever came first), delivery and 1 month postpartum following analyses using a mixed-effects logistic regression model from which the marginal estimate was obtained. An absolute mean difference and two-sided 95% CI of FCM and SOC (oral iron) of the estimated change from baseline to 36 weeks’ gestation or delivery (whichever came first), delivery and 1 month postpartum is displayed for venous hemoglobin concentration following analyses using a likelihood-based longitudinal data analysis model. A geometric mean ratio and two-sided 95% CI is displayed for serum ferritin concentration after a log base e transformation due to skewness.

^c^Estimated gestational age (weeks) is dated either using first day of the last menstrual period or fundal height.

^d^Anemia based on venous Hb indicates Hb<11.0 g dl^−1^ up to and including delivery and Hb <12.0 g dl^−1^ postpartum. Moderate/severe anemia based on venous Hb indicates Hb <10.0 g dl^−1^ up to and including delivery and Hb <11.0 g dl^−1^ postpartum.

^e^The number needed to treat for anemia at 36 weeks’ gestation or at delivery (whichever came first) is 6 (95% CI 4–13), meaning on average 6 women in their third trimester of pregnancy with moderate or severe anemia (capillary Hb <10.0 g dl^−1^) would need to be treated with FCM in order for one additional woman to not have maternal anemia (venous Hb >11.0 g dl^−1^) at 36 weeks’ gestation or at delivery (whichever came first).

^f^The Holm procedure was applied to the key secondary maternal outcomes. The *P* value for hemoglobin and ferritin concentration at 36 weeks’ gestation or at delivery (whichever came first) and 1 month postpartum remained significant after controlling for multiple comparisons with the Holm procedure. The Holm procedure was planned for the key secondary neonatal outcomes but not executed due to non-statistically significant treatment effects. No *P* values are presented for other maternal and neonatal secondary outcomes to reduce multiple testing.

^g^The *P* values and two-sided 95% CIs presented have not been adjusted for multiple comparisons. The intervals may not be used in place of hypothesis testing.

^h^The absolute median (IQR) ferritin levels at 36 weeks’ gestation or at delivery (whichever came first) were 175.6 (90.4–305.2) in the FCM group and 23.5 (14.6–40.1) in the SOC (oral iron) group. The estimated treatment effect of 6.06 is obtained as the ratio of the estimated geometric mean ratio (95% CI) of the 36 weeks’ gestation or at delivery (whichever came first) value to baseline value 186.8 (162.4 to 214.9) in the FCM group divided by 30.8 (32.1 to 36.1) in the SOC (oral iron) group. The absolute median (IQR) ferritin levels at 1 month postpartum were 113.4 (62.9–171.4) in the FCM group and 29.9 (16.5–56.7) in the SOC (oral iron) group. The estimated treatment effect of 3.22 is obtained as the ratio of the estimated geometric mean ratio (95% CI) of the 1 month postpartum value to baseline value 109.4 (95.1, 125.9) in the FCM group divided by 34.0 (32.1, 36.1) in the SOC (oral iron) group. The absolute median (IQR) ferritin levels at delivery were 132.0 (66.8–250.4) in the FCM group and 29.5 (16.6–48.6) in the SOC (oral iron) group. The estimated treatment effect of 4.25 is obtained as the ratio of the estimated geometric mean ratio (95% CI) of the delivery value to baseline value 141.6 (122.0, 164.2) in the FCM group divided by 33.3 (31.4, 35.3) in the SOC (oral iron) group.

^i^Iron deficient indicates serum ferritin <15 µg liter^−1^ or serum ferritin <30 μg liter^−1^ if CRP >5 mg liter^−1^, and iron deficient anemia indicates Hb <11.0 g dl^−1^ and serum ferritin <15 µg liter^−1^ or serum ferritin <30 μg liter^−1^ if CRP >5 mg liter^−1^.

^j^A PR of FCM versus standard of care (oral iron) is displayed for low birthweight and stillbirth following analyses using a log-binomial regression model. An absolute mean difference for weight at delivery and 1 month of age, length at delivery and 1 month of age, hemoglobin concentration at 1 month of age, length-for-age *z*-score, weight-for-age *z*-score, and weight-for-length *z*-score at 1 month of age between FCM and SOC (oral iron) is displayed following fitting a linear regression model. Birthweight, low birthweight and birth length were multiply imputed before analyses.

^k^The number needed to treat for low birthweight (<2,500 g) is 90. The 95% CI is not provided because there is no statistically significant treatment effect on low birthweight.

^l^The risk ratio of FCM versus standard of care displayed for stillbirth excludes the stratification factor (site) in the log-binomial regression model due to data separation issues. Firth logistic regression with site in the model was run as a post hoc sensitivity analyses (risk ratio 1.20 (95% CI 0.31, 4.65); *P* = 0.80).

Mean birthweight in infants born to mothers receiving FCM was 2,975.1 g (s.d. = 431.4) and in infants born to mothers receiving SOC was 2,958.7 g (s.d. = 479.5), mean difference 10.9 g (95% CI −65.7, 87.5), *P* = 0.78.

### Secondary outcomes

FCM reduced anemia prevalence compared to SOC at delivery (20.7% vs 34.9%, PR 0.61 (95% CI 0.46, 0.81)) and 1 month postpartum (36.0% vs 57.9%, PR 0.62 (95% CI 0.51, 0.75)). Compared to SOC, FCM also reduced the prevalence of moderate and severe anemia, iron deficiency and iron deficiency anemia and increased mean hemoglobin and ferritin from baseline at all time points. There was no evidence FCM influenced neonatal length-for-age or weight-for-age *z*-scores or neonatal hemoglobin concentration at 1 month of age (Table 2). Of the 252 out of 405 (62.2%) of women with anemia at 36 weeks’ gestation, 68 of 236 (28.8%) had biochemical evidence of inflammation.

### Safety outcomes

There were no deaths during the trial among all participating women. An adverse event during or up to 30 min following FCM administration was reported by 9 of 297 participants (3%) receiving this drug; no infusion-related serious adverse events occurred, and no medical intervention was required for any infusion reaction (Table 3). From randomization to 4 weeks postpartum, a total of 128 maternal adverse events occurred in 118 women, with 18.2% of women receiving FCM and 21.9% receiving SOC reporting at least one adverse event (risk ratio 0.81 (95% CI 0.58, 1.11), *P* = 0.19) (Table 4). There was no evidence of a difference between groups in the proportion of women with infection overall or with malaria (Table 4), or in any specific infection or other adverse event class (Supplementary Table 2). At least one serious adverse event occurred in 5.7% women who received FCM compared with 6.5% who received SOC. The proportion of women who experienced the individual or composite safety outcome of death, hemorrhage, transfusion, or intensive care admission were similar between the two groups. The prevalence of hypophosphatemia and inflammation (measured by CRP) did no differ between groups (Table 3). Neonatal death or stillbirth occurred in 8 of 305 (2.6%) in the FCM group and 9 of 296 (3.0%) in the SOC group. From birth to 1 month of age, 120 adverse events occurred in 116 neonates with 18.4% born to mothers who received FCM and 20.3% born to mother who received SOC (risk ratio 0.90 (95% CI 0.65, 1.25), *P* = 0.54).

**Table 3:** Maternal safety outcome: adverse reactions during FCM administration

FCM-related adverse events, *n* (%)	FCM *n* = 297
At least one infusion-related adverse event	9 (3.0)
Headache	0 (0.0)
Dizziness	3 (1.0)
Discoloration of the skin	0 (0.0)
Nausea	6 (2.0)
Vomiting	1 (0.3)
Upper abdominal pain	0 (0.0)
Dyspepsia	0 (0.0)
Anaphylaxis	0 (0.0)
Flushing	0 (0.0)
Shortness of breath	0 (0.0)
Chest pains	0 (0.0)
Other^a^	2 (0.7)

Includes all women who were randomized to the FCM group. There were 12 FCM-related adverse events in 9 women.

^a^Includes pruritis on both hands and isolated hypotension.

**Table 4:** Effects of FCM on maternal and neonatal safety outcomes

	FCM	SOC (oral iron)^a^	Risk ratio (95% CI)^b^	*P* value^c^
	*n* = 297	*n* = 292		
**Maternal safety outcomes**^**d**^				
Adverse events				
At least one adverse event	54 (18.2)	64 (21.9)	0.81 (0.58, 1.11)	0.19
At least one serious adverse event	17 (5.7)	19 (6.5)	0.85 (0.45, 1.59)	0.61
Death	0 (0.0)	0 (0.0)	–	–
**Adverse events of special interest**				
Composite severe medical event^e^	12 (4.0)	7 (2.4)	1.69 (0.67, 4.22)	0.27
Individual components of severe medical event				
Death	0 (0.0)	0 (0.0)	–	–
Hemorrhage	8 (2.7)	4 (1.4)	1.97 (0.60, 6.46)	0.27
Blood transfusion^f^	11 (3.7)	7 (2.4)	1.54 (0.61, 3.93)	0.36
ICU care^g^	1 (0.3)	0 (0.0)	–	–
Malaria (preferred term)	1 (0.3)	1 (0.3)	0.98 (0.06, 15.64)	0.99
**Common adverse events occurring in** >**5% in any treatment group**				
System organ class				
Pregnancy, puerperium and perinatal conditions	35 (11.8)	46 (15.8)	0.75 (0.50, 1.13)	0.16
Preferred term				
Pregnancy, puerperium and perinatal conditions, other	34 (11.4)	46 (15.8)	0.73 (0.48, 1.10)	0.13
Perineal tear	17 (5.7)	27 (9.2)	0.62 (0.34, 1.11)	0.11
**Safety biomarkers**				
Hypophosphatemia^h^				
36 weeks’ gestation or delivery (whichever came first)				
Any	9 (3.6)	3 (1.2)	3.07 (0.84, 11.22)	0.089
Mild	7 (2.8)	2 (0.8)	–	–
Moderate	2 (0.8)	1 (0.4)	–	–
Severe	0 (0.0)	0 (0.0)	–	–
Delivery				
Any	3 (1.5)	3 (1.4)	1.11 (0.23, 5.41)	0.90
Mild	3 (1.5)	2 (0.9)	–	–
Moderate	0 (0.0)	1 (0.5)	–	–
Severe	0 (0.0)	0 (0.0)	–	–
1 month postpartum				
Any	2 (0.9)	1 (0.5)	1.98 (0.18, 21.69)	0.58
Mild	2 (0.9)	0 (0.0)	–	–
Moderate	0 (0.0)	1 (0.5)	–	–
Severe	0 (0.0)	0 (0.0)	–	–
Inflammation (elevated CRP)^i^				
36 weeks’ gestation or delivery (whichever came first)	102 (41.1)	106 (41.2)	0.99 (0.81, 1.22)	0.94
Delivery	153 (75.0)	150 (67.3)	1.11 (0.98, 1.25)	0.098
1 month postpartum	35 (16.1)	34 (15.6)	1.02 (0.66, 1.56)	0.94
**Neonate safety outcomes**^j^	***n*** = **305**	***n*** = **296**		
Adverse events				
At least one adverse event	56 (18.4)	60 (20.3)	0.90 (0.65, 1.25)	0.54
At least one serious adverse event	34 (11.1)	34 (11.5)	0.97 (0.62, 1.52)	0.90
Neonatal death^k^	4 (1.3)	6 (2.0)	0.65 (0.18, 2.27)	0.50
Neonatal death and stillbirth^k^	8 (2.6)	9 (3.0)	0.86 (0.34, 2.21)	0.76
Adverse events of special interest				
Malaria (preferred term)	0 (0.0)	0 (0.0)	–	–
**Common adverse events occurring in** >**5% in any treatment group**				
System organ class				
Infections and Infestations	24 (7.9)	25 (8.4)	0.93 (0.54, 1.59)	0.80
Preferred term				
Infections and Infestations, other	19 (6.2)	20 (6.8)	0.92 (0.50, 1.69)	0.79
Respiratory infection	15 (4.9)	15 (5.1)	0.97 (0.48, 1.95)	0.93

Adverse events were coded using version 5.0 of the Common Terminology Criteria for Adverse Events (US Department of Health Human Services, 27 November 2020) and use an appropriate Preferred Term and System Order Class for each adverse event verbatim term. Data are presented as number (percentage) of patients.

^a^One woman who was randomized in the SOC (oral iron) but later found to be not pregnant was excluded, she reported no adverse events.

^b^Outcomes are analyzed using a log-binomial regression model and display the estimate of the risk ratio of FCM versus standard of care (oral iron) and two-sided 95% CI. The risk ratio excludes the stratification factor (site) in the log- model due to data separation issues.

^c^The *P* values and two-sided 95% CIs presented have not been adjusted for multiple comparisons. The intervals should not be used in place of hypothesis testing.

^d^Includes all women who were treated, presented according to treated group. This includes 12 mothers with multiple pregnancies (8 in the FCM group and 4 in the SOC (oral iron) group. In the FCM group, there were a total of 60 adverse events and 18 serious adverse events. In the SOC group (oral iron), there were a total of 68 adverse events and 19 serious adverse events.

^e^The composite severe medical event outcome was women with at least one severe medical event of death, hemorrhage, blood transfusion or ICU care recorded in the mother. Death, hemorrhage, blood transfusion and admission to ICU are captured across the whole trial period. There were 20 such events in the FCM group and 11 in the SOC (oral iron) group.

^f^Blood transfusion denotes that a blood transfusion was required for the mother.

^g^ICU care denotes mothers required ICU admission.

^h^Any hypophosphatemia is defined as (PO_4_ < 0.80 mmol liter^−1^), mild hypophosphatemia is defined as (0.64 < PO_4_ < 0.80 mmol liter^−1^), moderate hypophosphatemia is defined as (0.32 < PO_4_ < 0.64 mmol liter^−1^) and severe hypophosphatemia is defined as (PO_4_ < 0.32 mmol liter^−1^). Severity of hypophosphatemia does not have statistical testing due to sparse data. Hypophosphatemia was seen at baseline in 3 (1.6%) of 193 women treated with FCM and 3 (1.5%) of 195 women treated with standard of care (oral iron).

^i^Inflammation indicates CRP >5 mg liter^−1^.

^j^Includes all live-born neonates (except for the death and stillbirth) born to pregnant women who were treated, presented according to treated group of the mother. This includes 24 twins (16 in the FCM group and 8 in the SOC (oral iron) group). In the FCM group, there were a total of 57 adverse events and 35 serious adverse events. In the SOC group (oral iron), there were a total of 63 adverse events and 35 serious adverse events.

^k^Neonatal death denotes death in the first 28 days of life of a child with evidence of life following delivery. Stillbirth denotes pregnancy loss after 22 weeks and prior to delivery with no evidence of life at delivery.

### Subgroup analyses

**Fig. 2:**
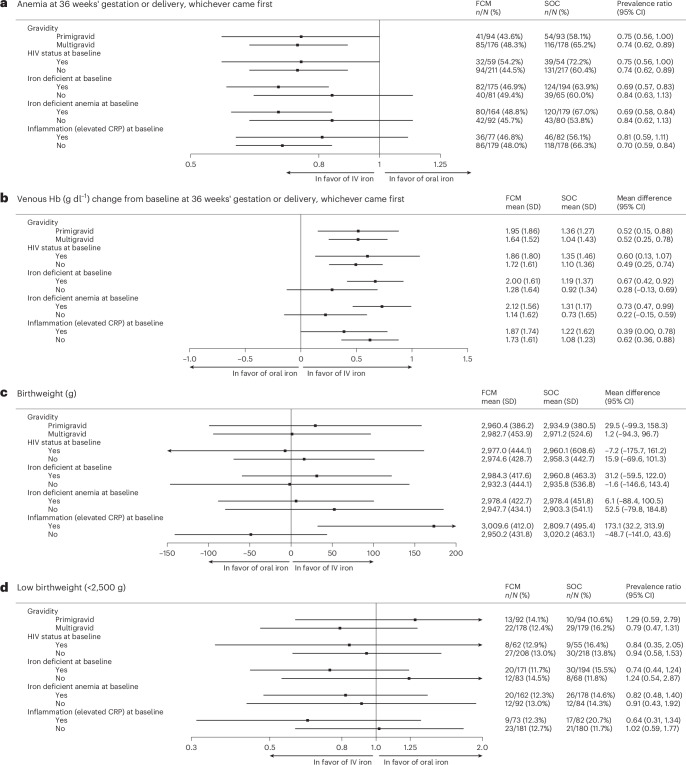
Subgroup analyses. **a**, A PR and 95% CI of FCM versus standard of care (oral iron) is displayed for anemia at 36 weeks’ gestation or at delivery (whichever came first) using a log binomial regression model, including study participants (mothers) as a random intercept to account for multiple time points and adjusting for stratification factor (site). **b**, An absolute mean difference and 95% CI of venous hemoglobin concentration (g dl^−1^) change from baseline to 36 weeks’ gestation or delivery (whichever came first) between FCM and standard of care (oral iron) is displayed following fitting a likelihood-based longitudinal data analysis model. **c**, An absolute mean difference and 95% CI of birthweight (g) between FCM and standard of care (oral iron) is displayed following fitting a linear regression model. **d**, A PR and 95% CI of FCM versus standard of care (oral iron) is displayed for low birthweight using a log binomial regression model. Birthweight and low birthweight were multiply imputed before analyses. The models in both (**a**) and (**b**) include subgroup (main effect) and subgroup-by-treatment-by-visit interactions (and subgroup-by-treatment and subgroup-by-visit interactions) to evaluate how the treatment effect differs between subgroup categories. The models in both (**c**) and (**d**) include subgroup (main effect) and subgroup-by-treatment interactions to evaluate how the treatment effect differs between subgroup categories. The intention-to-treat basis indicates outcomes analyzed according to the randomly allocated group of the woman. The 95% CIs presented have not been adjusted for multiple comparisons. The intervals cannot be used in place of hypothesis testing. FCM, ferric carboxymaltose; SOC, standard of care. No statistical analysis was performed due to the small sample size in the subgroup of those with severe anemia at baseline (*n* = 23).

Prespecified subgroup analyses were performed for maternal hemoglobin and anemia, birthweight and low birthweight (Fig. 2a–d and Supplementary Table 3). At the primary timepoint, women with baseline iron deficiency anemia (venous measurement) experienced a greater increase in hemoglobin from FCM (compared to SOC) than women who did not have iron deficiency anemia. Babies born to mothers with baseline inflammation exhibited a higher birth weight with FCM compared to SOC; this was not seen in babies born to non-inflamed mothers. No heterogeneity was observed for gravidity and baseline HIV status.

### Additional analyses

To confirm the robustness of the findings to missing data assumptions, analysis set or analysis methods, we prospectively planned and conducted several additional analyses that did not alter the results (Supplementary Table 4). We also confirmed that the treatment effect on anemia remains statistically significant if defined at 36 weeks’ gestation (aligned with REVAMP trial) (Supplementary Table 4). In a post-hoc analysis excluding participants who received blood transfusions (11 FCM and 7 SOC), the treatment effect on anemia at the primary timepoint did not materially change (FCM 34.2% (78/228) vs SOC 57.4% (132/230), PR 0.59 (95% CI 0.48, 0.72)).

## Discussion

In Malawian women in the third trimester of pregnancy with moderate or severe anemia detected by capillary hemoglobin measurement, treatment with a single dose of FCM was superior to SOC in reducing the prevalence of anemia prior to childbirth. In this population, treatment of six women with moderate or severe anemia with FCM would be needed to alleviate one case of anemia. FCM also reduced the prevalence of anemia, iron deficiency and iron deficiency anemia at each follow-up timepoint (including 1 month postpartum). FCM did not influence infant birthweight or infant hemoglobin concentration. Administration of FCM to women in health centers with limited resources appeared safe.

Our results complement the findings of our previous trial (REVAMP) that recruited Malawian women in the second trimester of pregnancy with otherwise similar eligibility criteria, and did not identify a benefit from FCM on anemia prevalence at 36 weeks’ gestation or delivery, although it did indicate a benefit from FCM on anemia prevalence 4 weeks after infusion^15^. Our findings highlight the faster response from intravenous iron compared to oral iron in raising hemoglobin concentration, as previously suggested in a network meta-analysis of trials of intravenous iron in pregnancy^16^, and emphasize the role of intravenous iron when a rapid response is needed. FCM induced marked increases in ferritin; although the clinical significance of early post-treatment increases are uncertain, the sustained elevation 4 weeks postpartum indicates superiority of FCM on iron stores.

Another difference between the two trials is the higher prevalence of baseline iron deficiency in REVAMP-TT (71.7%) compared with REVAMP (43.4%). We observed enhanced efficacy of FCM in reducing the prevalence of anemia in women with baseline iron deficiency. In the previous trial, women were presenting for their first antenatal clinic visit and had not yet received Intermittent Preventive Therapy in pregnancy (IPTp), which is implemented in Malawi. As such, even though these women may have been RDT negative, they may have harbored subpatent *Plasmodium* parasitemia that may have driven their anemia and been refractory to iron therapy. Conversely, women in the third trimester of pregnancy may likely have already received prior antenatal care (although these data were unavailable in this study), including IPTp, and their anemia was more often caused by iron deficiency. A substantial proportion (about a third) of residual anemia was attributable to inflammation. Taken together, the REVAMP-TT and REVAMP trials define a use-case for anemia treatment with intravenous iron in sub-Saharan Africa in late pregnancy. Our data also provide evidence to support recommendations for management of iron deficiency anemia in high-income settings such as the United Kingdom^12^ and United States^17^.

Previous systematic reviews have found treatment of antenatal iron deficiency anemia with oral^18^ or IV iron may increase birthweight^19^. However, consistent with the REVAMP trial, we did not observe a beneficial effect from FCM on birthweight or other neonatal outcomes^15^. Beyond iron deficiency, in this setting, placental malaria may occur from late in the first trimester (prior to the first antenatal clinic visit and treatment with IPTp) and is an important driver of low birthweight in sub-Saharan Africa^20^. Maternal systemic inflammation^21^ and gut dysbiosis^22^ have also been associated with low birthweight and preterm birth. The ongoing high prevalence of anemia may also in part be driven by inflammation. Inflammation is not resolved by IV iron: to improve birthweight and further reduce antenatal anemia in sub-Saharan Africa, future studies could evaluate combining iron with infection control.

We observed heterogeneity in the treatment effect on birthweight between neonates born to women with and without inflammation at baseline. Specifically, birthweight was higher in neonates born to women with baseline inflammation randomized to FCM compared with SOC; no such benefit from FCM was seen in women without inflammation at baseline. This result was discrepant with the previous REVAMP trial, where we observed that birthweight may have been lower in women with inflammation randomized to FCM. One possible explanation is different causes of inflammation between the two populations: for example, women in the REVAMP trial were treated in the second trimester of pregnancy at their first antenatal clinic visit, and had not yet received IPTp; in these cases, inflammation may have been driven by sub-microscopic *Plasmodium* parasitemia; conversely, in REVAMP-TT, most women had experienced prior antenatal care and receipt of IPTp, potentially meaning inflammation may have different drivers (for example, bacterial or viral infections). Further studies to define differences in the causes of inflammation across pregnancy would help refine a role for FCM in influencing birthweight in this population. This finding was detected in a prespecified subgroup analyses but should interpreted with caution until it has been mechanistically explained or reproduced in other studies.

In this trial, we delivered FCM in government-run antenatal care centers where infrastructure was limited. Prior to launching the intervention, we conducted a readiness assessment and ensured centers were equipped with medications and equipment for resuscitation and we provided education to local health workers (consistent with European Medicines Agency guidelines)^23^ whom were ultimately able to independently administer the drug in several cases. Beyond this, the centers had few resources or basic furnishings, and regularly experienced lack of electricity. Across REVAMP and REVAMP-TT, we have delivered 727 FCM infusions in an outpatient rural sub-Saharan African setting without an infusion-related serious adverse event. Thus, delivery of FCM could be feasible and safe even where infrastructure is basic. Hypophosphatemia was seen in 3.6% of women randomized to FCM at the primary timepoint and was rare thereafter; the primary timepoint (~55 days post infusion) may have been too late to detect the peak in this common FCM-related side effect, which usually peaks at 7–14 days post infusion^24^, however our data indicate FCM does not cause sustained hypophosphatemia in this population.

Our trial has several strengths. It was set in health centers of rural Malawi, where the need for innovative solutions for anemia are immense, and thus provides evidence that directly informs antenatal care in such settings. Importantly, even in cases where government staff administered the study intervention all follow-up procedures including outcome assessments were undertaken by dedicated study staff and using laboratory and clinical study equipment, ensuring the quality of our data. We applied pragmatic eligibility criteria, with inclusion based on capillary measurement of hemoglobin concentration, as point of care tests for iron status are not presently available for deployment in the field. This simple approach means the results of our trial can be translated for implementation in resource limited settings even without the advent of new point of care iron-testing technology or a change in practice towards venous blood measurement. However, non-iron deficient individuals were included and treated in our study; these women appeared to receive less benefit from the intervention, diluting the responses to iron across the cohort overall. WHO recommendations for definition of anemia have recently changed^25,26^. However, these do not change definitions of anemia in the third trimester of pregnancy and do not affect the interpretation of our results.

Our study also had limitations. The trial was open-label, with participants and study staff unblinded to the intervention. FCM is a black liquid, and it would have been challenging to administer placebo in the field setting. However, the primary maternal and neonate outcomes were measured by laboratory staff and midwives blinded to the intervention group. The SOC arm provided twice-daily oral iron consistent with government guidelines: twice daily dosing in non-pregnant women has been shown to be associated with reduced efficiency of absorption and increased gastrointestinal adverse events^27,28^. We were unable to assess adherence to oral iron in this study as we were uncertain reporting would be robust and concerned questioning could influence participants’ behavior^29^. The trial did not collect data on prior use of iron supplements. Some women delivered before their scheduled week 36 visit, which might have been due to uncertainty around their estimated 36 weeks’ gestation. We used their hemoglobin concentration prior to delivery to determine their anemia status for the statistical analysis at the primary timepoint. Postpartum hemorrhage was not a primary or key secondary outcome of the trial and was recorded as an adverse event based on midwife assessment. Direct measurement of intrapartum blood loss, for example, by use of a calibrated drape, is difficult to implement routinely and may be contaminated by amniotic fluid^30^; as such we did not use this in our field study. The incidence of postpartum hemorrhage may therefore be underestimated in our trial. As prespecified, we report outcomes to four weeks postpartum^29^; an extension study comprising follow-up of women and infants to 12 months postpartum will provide interesting results on long term effects of FCM on maternal and infant hemoglobin and iron status.

Our randomized controlled trial in rural Malawi indicates that women in low-income settings with anemia in the third trimester of pregnancy can be safely treated with FCM to reduce anemia prior to delivery.

## Methods

REVAMP-TT (randomized trial of intraVenous iron for Anemia in Malawian Pregnant women in the Third Trimester) was an open-label, parallel-group, individually randomized controlled trial run in primary health care settings across Zomba district in southern Malawi. The trial protocol and statistical analysis plan are included in the Supplemental Materials and were published before database lock^29^. The trial was registered (ANZCTR12621001239853) before recruitment commenced. No commercial support for the trial was provided.

### Inclusion and ethics statement

The REVAMP-TT trial was conceived within a long-standing partnership between Training and Research Unit of Excellence (TRUE, Malawi) and The Walter and Eliza Hall Institute of Medical Research (WEHI, Australia), with input from The School of Population and Global Health, University of Melbourne. TRUE and WEHI researchers co-developed the study design, selected the outcomes and planned the analysis. Consumers and local health workers were deeply involved in development of study procedures through formal co-design workshops held with TRUE and University of Melbourne researchers prior to study implementation^31^. Through the REVAMP trials program, three Malawian students undertook PhD programs.

The trial was approved by ethics committees in Malawi (National Health Sciences Research Committee of Malawi Approval – NHSRC 20/11/2622) and Australia (Human Research Ethics, WEHI 20/25). An independent Data Safety Monitoring Committee monitored the trial.

### Participants

Participants were eligible for the trial if they had a confirmed pregnancy 27 to 35 weeks’ gestation based on either last menstrual period or fundal height (ultrasound-based gestational age screening is not effective in late pregnancy^32^); a capillary hemoglobin concentration less than 10 g dl^−1^ (moderate or severe anemia) measured by HemoCue 301+ (Angelholm), a negative malaria rapid diagnostic test, were afebrile and were expecting to reside and deliver their baby within the Zomba study site catchment area. We utilized capillary hemoglobin measurement but not iron parameters as we judged that screening large populations of women for anemia in pregnancy using venous blood and iron biomarkers is not presently feasible and that applying these parameters in inclusion criteria would limit the applicability of our findings. Women were excluded if they had been previously or were currently enrolled in another trial (including the REVAMP trial), had a known hypersensitivity to any study drug, exhibited clinical symptoms of malaria or other infection, had a known history of sickle cell or sickle-hemoglobin C anemia, were clinically unstable with a low hemoglobin level requiring a blood transfusion (usually hemoglobin <5 g dl^−1^), or had evidence of pre-eclampsia. HIV positivity was not an exclusion criterion.

Women attending one of eight primary antenatal clinics in Zomba district (Likangala, Bimbi, Lambulira, Domasi, Naisi, Matawale, Sadzi and City) were screened for eligibility during their antenatal visit. These clinics provide midwife-driven routine antenatal and perinatal care. Women were screened using data collected as part of their routine antental care, and written consent was obtained from all eligible women before enrollment to the trial.

### Randomization, allocation concealment and blinding

A randomization schedule of randomly permuted blocks of size 4 or 6, stratified by site, was used to randomly allocate participants 1:1 to one of the two treatment groups within site. The randomization list was computer-generated by an independent statistician. Allocation codes were contained in sealed, opaque envelopes to randomly assign participants. Although the trial was open-label, laboratory scientists measuring hemoglobin concentration, midwives collecting birth outcome data, data managers, and investigators and statisticians at WEHI (Australia) were all blinded to the treatment allocation until the database was locked for unblinding. As trial treatments were delivered in primary care centers, it was judged unfeasible to provide placebo infusions.

### Study interventions and procedures

The trial operated in government-run primary health care facilities using local infrastructure. In all clinics, government staff (for example, nurses) received training and were trained by study staff to eventually administer the study intervention. The rooms were equipped with medications, fluids and oxygen. Women in the intervention group received a single dose of FCM (CSL-Vifor, purchased commercially), 20 mg kg^−1^ up to 1,000 mg, diluted in 250 ml saline, given intravenously over 15 min^15^. Women were clinically monitored post infusion and fetal heartbeat monitored by fetoscope. Women in the SOC group were provided oral iron (60 mg elemental iron as ferrous sulfate, twice daily for 90 days, aligned to Malawian government policy) accompanied by the standardized educational message aligned with local practice (for example, frequency of use, safe storage, common side effects). In addition, all participants received intermittent preventive treatment of malaria (IPTp) with sulfadoxine–pyrimethamine as recommended in national guidelines: 1,500 mg sulfadoxine and 75 mg pyrimethamine (SP, 3 tablets of SP strength at 500 mg/25 mg)^33^. Following enrollment and treatment, participants were followed up at 36 weeks’ estimated gestation, delivery and 1 month postpartum.

Eligibility assessments and informed consent procedures were undertaken by study staff. The study staff also provided all the initial interventions until government health workers were sufficiently trained, after which government health staff administered the interventions. All outcome assessments were undertaken by study staff.

Data were recorded in digital form with REDCap, using electronic tablets, and backed up daily to a local backup server at TRUE, Blantyre, Malawi, with a deidentified fortnightly backup to WEHI, Melbourne, Australia.

### Outcomes

The primary outcome was anemia (venous hemoglobin <11 g dl^−1^) at 36 weeks’ gestation or during delivery^34^, whichever occurred first, enabling the trial to evaluate the impact of FCM on anemia as a woman enters childbirth.

Maternal secondary outcomes included hemoglobin and ferritin concentrations, and anemia, iron deficiency (defined by ferritin <15 μg liter^−1^ or ferritin <30 μg liter^−1^ if CRP >5 mg liter^−1^)^35^, and iron deficiency anemia at 36 weeks’ gestation or during delivery, whichever occurred first, delivery and at 1 month postpartum. Neonatal outcomes included birthweight and low birthweight (birthweight <2,500 g) measured within 24 hours of delivery, and infant length-for-age, weight-for-age, and weight-for-length *z*-scores, as well as hemoglobin concentration at 1 month of age. Because of uncertainty in the precise gestational age at recruitment, we did not include preterm birth as an outcome. The trial provided 24-hour midwifery cover for trial participants (and any other women in labor needing care), enabling measurement of neonatal outcomes^36^.

### Safety outcomes

Participants had access to a seven-days-a-week clinical service provided by the study team to report and receive treatment for any adverse event affecting themselves or their baby, regardless of apparent causality, at any time following randomization to trial exit. These presentations were recorded and coded against version 5.0 of the Common Terminology Criteria for Adverse Events to classify adverse events^37^. Safety outcomes include maternal and infant adverse events and serious adverse effects (unplanned hospitalization or death, including pregnancy loss and neonatal death), reported at any time between randomization and 1 month postpartum. We also measured hypophosphatemia (serum phosphate <0.80 mmol liter^−1^), which has been observed with FCM administration^24^, and inflammation (CRP >5 mg liter^−1^) as an indicator of infection, at 36 weeks, delivery, and 1 month postpartum. We also recorded adverse events occurring during FCM infusion (using a checklist administered to women in the FCM group).

### Statistical analysis

The sample size was 590 women (295 women per group) when accounting for a 10% drop out in the primary outcome. We planned to detect a 14% absolute reduction in anemia prevalence by FCM compared with SOC with 90% power (two-sided alpha of 5%), consistent with the pivotal FCM vs oral iron trial (Fer-ASAP), which demonstrated a 14% absolute reduction in anemia prevalence compared with oral iron^38^. After accounting for a miscarriage and stillbirth rate of 1%, this sample size also had 72% to 97% power to detect a 100 to 150 g absolute difference in birthweight between FCM and SOC, assuming a standard deviation of 450 g (ref. ^7^) (two-sided alpha of 5%). After 77.8% (459/590) women had non-missing primary outcome data, we conducted an adaptive sample size re-estimation as per the ‘promising zone’ methodology of Mehta and Pocock^39^ defined by conditional power between 0.388 and 0.9. The preplanned sample size of 590 was confirmed after execution of the method by an independent statistician and recommendation from the Data Safety Monitoring Committee; all investigators remained blinded to this analysis until database lock.

Maternal anemia was analyzed using a mixed-effects logistic regression model, with fixed effects of treatment, study visit, treatment by study visit and a random intercept for women. The marginal standardization technique was used to obtain the PR with CIs calculated by the delta-method. We applied similar analyses to repeatedly measured dichotomous secondary maternal outcomes. Secondary maternal outcomes of hemoglobin and log-transformed ferritin were analyzed using a likelihood-based longitudinal data analysis model^40^ assuming a common baseline mean across the two groups, and an unstructured variance–covariance among the repeated measurements. Birthweight (and other continuous neonatal secondary outcomes) was analyzed with a linear regression model. Binary secondary neonatal outcomes were analyzed with a log-binomial regression model, as were maternal and neonate adverse event outcomes and maternal safety biomarkers (hypophosphatemia and inflammation). Additional analyses for efficacy outcomes included analyses excluding participants with protocol violations (that is, multiple births) and adjusted analyses with prespecified covariates. Six subgroups (gravidity, HIV status, severe anemia, iron deficiency, iron deficiency anemia, and inflammation) were predefined for two maternal (anemia, hemoglobin at the primary timepoint) and neonate (birthweight, low birthweight) outcomes. All subgroup analyses were performed except severe anemia due to small sample size.

Analysis models used all available data according to the maternal randomized treatment group. Birthweight, low birthweight and birth length were multiply imputed before analyses. To assess sensitivity of results for the primary maternal outcome (anemia) to the missing-at-random assumption, we conducted an analysis based on pattern-mixture models. Results are presented as point estimates and two-sided 95% CIs alongside *P* values. The Holm procedure^41^ was used to control family-wise error rate at 5% for the secondary maternal outcomes (hemoglobin, ferritin at the primary timepoint and 1 month postpartum) and secondary neonatal outcomes (hemoglobin, weight at 1 month of age), separately. No *P* values are presented for other maternal and neonatal secondary outcomes to reduce multiple testing. Based on chance alone, up to one interaction test of the subgroup analyses would be expected to have a *P* value < 0.05. No multiplicity adjustment is applied to CIs, and these cannot be used in place of hypothesis testing. Analyses were adjusted for the stratification factor (site) where possible. Analyses were performed using Stata SE, version 18.0 (StataCorp).

### Reporting summary

Further information on research design is available in the Nature Portfolio Reporting Summary linked to this article.

## Online content

Any methods, additional references, Nature Portfolio reporting summaries, source data, extended data, supplementary information, acknowledgements, peer review information; details of author contributions and competing interests; and statements of data and code availability are available at 10.1038/s41591-024-03385-w.

## Supplementary information

Supplementary Information Supplementary Data Tables 1–4 CONSORT Diagram Protocol Paper Statistical Analysis Plan

Reporting Summary

## Data Availability

Underlying deidentified individual participant data encompassing the reported trial results and a data dictionary are accessible at figshare (10.26188/26968171.v1). Data are available under the terms of Creative Commons Attribution 4.0 International License (CC-BY-4.0).
