# Author Correction: Ferric carboxymaltose for anemia in late pregnancy: a randomized controlled trial

**DOI:** 10.1038/s41591-025-03492-2

**Published:** 2025-01-13

**Authors:** Sant-Rayn Pasricha, Ernest Moya, Ricardo Ataíde, Glory Mzembe, Rebecca Harding, Martin N. Mwangi, Truwah Zinenani, Khic-Houy Prang, Justina Kaunda, Owen P. L. Mtambo, Maclean Vokhiwa, Gomezgani Mhango, Elisabeth Mamani-Mategula, Katherine Fielding, Ayşe Demir, Naomi Von Dinklage, Hans Verhoef, Alistair RD McLean, Lucinda Manda-Taylor, Sabine Braat, Kamija S. Phiri

**Affiliations:** 1 Walter and Eliza Hall Institute of Medical Research, Parkville, Victoria Australia; 2 Diagnostic Haematology, The Royal Melbourne Hospital, Parkville, Victoria Australia; 3 Clinical Haematology, The Peter MacCallum Cancer Centre and The Royal Melbourne Hospital, Parkville, Victoria Australia; 4 Department of Medical Biology, The University of Melbourne, Parkville, Victoria Australia; 5 Training and Research Unit of Excellence (TRUE), Blantyre, Malawi; 6 Department of Public Health, School of Public and Global Health, Kamuzu University of Health Sciences, Blantyre, Malawi; 7 Department of Infectious Diseases at the Peter Doherty Institute, The University of Melbourne, Melbourne, Victoria Australia; 8 The Micronutrient Forum, Healthy Mothers Healthy Babies Consortium, Washington, DC USA; 9 Centre for Health Policy, Melbourne School of Population and Global Health, The University of Melbourne, Parkville, Victoria Australia; 10 Division of Human Nutrition and Health, Wageningen University, Wageningen, The Netherlands

**Keywords:** Anaemia, Randomized controlled trials, Reproductive signs and symptoms

Correction to: *Nature Medicine* 10.1038/s41591-024-03385-w, published online 6 January 2025.

In the version of the article initially published, in Fig. 2c, the labels “In favor of IV iron” and “In favor of oral iron” were switched and have now been amended so that “In favor of oral iron” is on the left and “In favor of IV iron” is on the right in the HTML and PDF versions of the article.

