# Supplementary material for: Ferric carboxymaltose for anemia in late pregnancy: a randomized controlled trial
Source: Nat Med. 2025 Jan 6;31(1):197–206. doi: 10.1038/s41591-024-03385-w (PMC11750709; doi:10.1038/s41591-024-03385-w)
Supplement: Supplementary file 1 — Supplementary Data Tables 1–4 CONSORT Diagram Protocol Paper Statistical Analysis Plan [file 41591_2024_3385_MOESM1_ESM.pdf]

nature medicine

Supplementary information https://doi.org/10.1038/s41591-024-03385-w

Ferric carboxymaltose for anemia in late pregnancy: a randomized controlled trial

In the format provided by the authors and unedited

REVAMP-TT Extended Data

Supplementary Data Table 1. Baseline characteristics of the participating pregnant women (per-protocol population)........2

Supplementary Data Table 2. Effects of ferric carboxymaltose on maternal and neonatal adverse events classified by systemic organ class or preferred term (Safety Population)*........4

Supplementary Data Table 3. Subgroup analyses of effect of ferric carboxymaltose on maternal and neonate efficacy outcomes according to maternal baseline characteristics (intention-to-treat population)........10

*Supplementary Data Table 4. Additional analyses on maternal and neonate outcomes**........14

Supplementary Data Table 1. Baseline characteristics of the participating pregnant women (per-protocol population)

	**Ferric carboxymaltose N = 289**	**Standard of care – Oral Iron N = 288**
Age (years), mean(SD)	24.4 (6.4)	24.7 (6.5)
Primigravid, no. (%)‡	106 (36.7%)	100 (34.7%)
Gestational age (weeks), median (IQR)*	30.0 (28.0-32.0)	30.0 (28.0-32.0)
Height (cm), mean (SD)	157.1 (5.7)	157.0 (5.5)
Weight (kg), mean (SD)	58.5 (7.8)	59.5 (8.6)
Body mass index (kg/m^2), mean (SD)†	23.7 (3.0)	24.1 (3.0)
Religion, no. (%)‡		
None	1 (0.3%)	0 (0.0%)
Christian	204 (70.6%)	200 (69.4%)
Muslim	80 (27.7%)	82 (28.5%)
Other	4 (1.4%)	6 (2.1%)
Education, no. (%)‡		
None	10 (3.5%)	8 (2.8%)
Lower Primary (1-5)	68 (23.5%)	70 (24.3%)
Upper Primary (6-8)	126 (43.6%)	112 (38.9%)
Lower Secondary (1-2)	35 (12.1%)	41 (14.2%)
Upper Secondary (3-4)	47 (16.3%)	52 (18.1%)
Tertiary	3 (1.0%)	5 (1.7%)
Marital status, no. (%)‡		
Single	28 (9.7%)	18 (6.2%)
Married	244 (84.4%)	250 (86.8%)
Widowed	2 (0.7%)	1 (0.3%)
Divorced/Separated	14 (4.8%)	18 (6.2%)
Other	1 (0.3%)	1 (0.3%)
Income source, no. (%)‡		
None	43 (14.9%)	48 (16.7%)
Subsistence farming	82 (28.4%)	69 (24.0%)
Large scale farming	2 (0.7%)	1 (0.3%)
Employed	19 (6.6%)	14 (4.9%)
Casual work for wages	77 (26.6%)	97 (33.7%)
Business	61 (21.1%)	56 (19.4%)
Other	5 (1.7%)	3 (1.0%)
HIV positive, no. (%)‡	60 (20.8%)	57 (19.8%)
Malaria RDT positive, no. (%)§	0 (0.0%)	0 (0.0%)
Capillary Hb<10 g/dL, no. (%)	289 (100.0%)	288 (100.0%)
Venous Hb (g/dL), mean(SD)¶	9.30 (1.32)	9.39 (1.23)
Anaemia (venous), no. (%)¶		
No (Hb≥11g/dL)	23 (8.0%)	26 (9.0%)

	Ferric carboxymaltose N = 289	Standard of care – Oral Iron N = 288
Mild (10g/dL≤Hb<11g/dL)	61 (21.2%)	60 (20.8%)
Moderate (7g/dL≤Hb<10g/dL)	191 (66.3%)	192 (66.7%)
Severe (Hb<7g/dL)	13 (4.5%)	10 (3.5%)
Ferritin (μg/L), median (IQR)‖	13.0 (8.5-20.9)	11.7 (8.0-17.0)
C-reactive protein (μg/L), median (IQR)	2.8 (1.6-5.6)	3.0 (1.5-6.0)
Iron deficient, no. (%)‖	195 (69.4%)	206 (74.4%)
Iron deficient anaemia, no. (%)‖	182 (64.8%)	192 (69.3%)
Inflammation, no. (%)**	81 (28.7%)	88 (31.7%)
Anaemia and inflammation, no. (%)**	74 (26.2%)	81 (29.1%)

The per-protocol population excludes 12 women due to multiple pregnancy. One woman who was randomised in the standard-of-care (oral iron) but later found to be not pregnant was excluded.

Participants were enrolled across eight primary antenatal clinics; Bimbi (n=46), City (n=58), Domasi (n=86), Lambulira (n=63), Likangala (n=124), Matawale (n=131), Naisi (n=53), Sadzi (n=16).

Hb denotes haemoglobin, HIV denotes human immunodeficiency virus, IQR denotes interquartile range (25th to 75th percentile), RDT denotes rapid diagnostic test, and SD standard deviation.

*Estimated gestational age (weeks) is dated either using First Day of the Last Menstrual Period or fundal height.

†Body mass index is the weight in kilograms divided by the square of the height in meters.

‡Religion, Education, Marital status, Income source, gravidity and HIV status were self-reported.

§Malaria RDT positive was based on confirmatory RDT testing by laboratory personnel on venous blood collected at enrolment.

¶There is one missing Venous Hb value in the Ferric carboxymaltose group.

‖There are 8 missing serum ferritin values in the Ferric carboxymaltose group and 11 in the standard-of-care (oral iron) group. Iron deficient indicates serum ferritin <15ug/L or serum ferritin <30ug/L if C-reactive protein >5μg/L, and iron deficient anaemia indicates Hb <11g/dL and serum ferritin <15 ug/L or serum ferritin <30 ug/L if C-reactive protein >5μg/L.

**There are 7 missing C-reactive protein values in the Ferric carboxymaltose group and 10 in the standard-of-care (oral iron) group. Inflammation indicates C-reactive protein >5μg/L, and anaemia and inflammation indicates Hb<11.0g/dL and C-reactive protein >5μg/L.

Supplementary Data Table 2. Effects of ferric carboxymaltose on maternal and neonatal adverse events classified by systemic organ class or preferred term* (Safety Population)

	**Ferric carboxymaltose**<br><br>**N = 297**	**Standard of care – Oral Iron**<br><br>**N = 292**	**Risk Ratio (95% CI)†‡**	**P-value‡**
**Maternal safety outcomes§**				
**System Organ Class**				
Blood and lymphatic system disorders	3 (1.0%)	6 (2.1%)	0.49 (0.12, 1.95)	0.31
**Preferred Term**				
Anaemia	3 (1.0%)	6 (2.1%)	0.49 (0.12, 1.95)	0.31
**System Organ Class**				
Cardiac disorders	1 (0.3%)	0 (0.0%)	—	—
**Preferred Term**				
Palpitations	1 (0.3%)	0 (0.0%)	—	—
**System Organ Class**				
Gastrointestinal disorders	1 (0.3%)	2 (0.7%)	0.49 (0.04, 5.39)	0.56
**Preferred Term**				
Abdominal pain	0 (0.0%)	1 (0.3%)	—	—
Dental Caries	0 (0.0%)	1 (0.3%)	—	—
Toothache	1 (0.3%)	0 (0.0%)	—	—
**System Organ Class**				
Infections and Infestations	14 (4.7%)	8 (2.7%)	1.72 (0.73, 4.04)	0.21
**Preferred Term**				
Sepsis	1 (0.3%)	0 (0.0%)	—	—
Urinary Tract Infection	0 (0.0%)	2 (0.7%)	—	—
Wound infection	1 (0.3%)	0 (0.0%)	—	—
Infections and Infestations, other	12 (4.0%)	6 (2.1%)	1.97 (0.75, 5.17)	0.17

COVID-19	1 (0.3%)	0 (0.0%)	—	—
Infection without focus	0 (0.0%)	1 (0.3%)	—	—
Malaria	1 (0.3%)	1 (0.3%)	0.98 (0.06, 15.64)	0.99
Puerperal sepsis	2 (0.7%)	0 (0.0%)	—	—
Respiratory infection	7 (2.4%)	3 (1.0%)	2.29 (0.60, 8.79)	0.23
Scabies	1 (0.3%)	0 (0.0%)	—	—
Varicella	0 (0.0%)	1 (0.3%)	—	—
**System Organ Class**				
Musculoskeletal and connective tissue disorders	1 (0.3%)	4 (1.4%)	0.25 (0.03, 2.19)	0.21
**Preferred Term**				
Back pain	0 (0.0%)	1 (0.3%)	—	—
Myalgia	1 (0.3%)	3 (1.0%)	0.33 (0.03, 3.13)	0.33
**System Organ Class**				
Pregnancy, puerperium and perinatal conditions	35 (11.8%)	46 (15.8%)	0.75 (0.50, 1.13)	0.16
**Preferred Term**				
Premature delivery	1 (0.3%)	0 (0.0%)	—	—
Pregnancy, puerperium and perinatal conditions, other	34 (11.4%)	46 (15.8%)	0.73 (0.48, 1.10)	0.13
Abruption	1 (0.3%)	0 (0.0%)	—	—
Antepartum Haemorrhage	3 (1.0%)	1 (0.3%)	2.95 (0.31, 28.19)	0.35
Eclampsia	1 (0.3%)	1 (0.3%)	0.98 (0.06, 15.64)	0.99
Episiotomy	1 (0.3%)	3 (1.0%)	0.33 (0.03, 3.13)	0.33
Fetal distress	2 (0.7%)	1 (0.3%)	1.97 (0.18, 21.57)	0.58
Obstructed labor	3 (1.0%)	2 (0.7%)	1.47 (0.25, 8.76)	0.67
Perineal Tear	17 (5.7%)	27 (9.2%)	0.62 (0.34, 1.11)	0.11
Postpartum Haemorrhage	4 (1.3%)	1 (0.3%)	3.93 (0.44, 34.98)	0.22
Pre-eclampsia	1 (0.3%)	6 (2.1%)	0.16 (0.02, 1.35)	0.093
Premature Rupture of Membranes	0 (0.0%)	1 (0.3%)	—	—

Preterm labor	1 (0.3%)	1 (0.3%)	0.98 (0.06, 15.64)	0.99
Prolonged labor	1 (0.3%)	0 (0.0%)	—	—
Retained Products	0 (0.0%)	1 (0.3%)	—	—
Uterine rupture	0 (0.0%)	1 (0.3%)	—	—
**System Organ Class**				
Reproductive system and breast disorders	0 (0.0%)	1 (0.3%)	—	—
**Preferred Term**				
Vaginal inflammation	0 (0.0%)	1 (0.3%)	—	—
**System Organ Class**				
Skin and subcutaneous disorders	1 (0.3%)	0 (0.0%)	—	—
**Preferred Term**				
Urticaria	1 (0.3%)	0 (0.0%)	—	—
**System Organ Class**				
Vascular disorders	3 (1.0%)	1 (0.3%)	2.95 (0.31, 28.19)	0.35
**Preferred Term**				
Hypertension	2 (0.7%)	1 (0.3%)	1.97 (0.18, 21.57)	0.58
Thromboembolic event	1 (0.3%)	0 (0.0%)	—	—
	**Ferric carboxymaltose**<br><br>**N = 305**	**Standard of care – Oral Iron**<br><br>**N = 296**	**Risk Ratio (95% CI)†‡**	**P-value ‡**
**Neonatal safety outcomes¶**				
**System Organ Class**				
Congenital, familial and genetic disorders	2 (0.7%)	2 (0.7%)	0.97 (0.14, 6.84)	0.98
**Preferred Term**				
Congenital, familial and genetic disorders - Other	2 (0.7%)	2 (0.7%)	0.97 (0.14, 6.84)	0.98
Cleft Palate	1 (0.3%)	0 (0.0%)	—	—
Polydactyly	0 (0.0%)	1 (0.3%)	—	—
Tongue Tie	0 (0.0%)	1 (0.3%)	—	—

Twin	1 (0.3%)	0 (0.0%)	—	—
**System Organ Class**				
General disorders and administration site conditions	2 (0.7%)	2 (0.7%)	0.97 (0.14, 6.84)	0.98
**Preferred Term**				
Death Neonatal	2 (0.7%)	1 (0.3%)	1.94 (0.18, 21.29)	0.59
General disorders and administration site conditions, Other	0 (0.0%)	1 (0.3%)	—	—
Irritable baby	0 (0.0%)	1 (0.3%)	—	—
**System Organ Class**				
Infections and Infestations	24 (7.9%)	25 (8.4%)	0.93 (0.54, 1.59)	0.80
**Preferred Term**				
Conjunctivitis	0 (0.0%)	2 (0.7%)	—	—
Folliculitis	1 (0.3%)	0 (0.0%)	—	—
Sepsis	2 (0.7%)	2 (0.7%)	0.97 (0.14, 6.84)	0.98
Skin infection	2 (0.7%)	1 (0.3%)	1.94 (0.18, 21.29)	0.59
Infections and Infestations, other	19 (6.2%)	20 (6.8%)	0.92 (0.50, 1.69)	0.79
Gastroenteritis	1 (0.3%)	2 (0.7%)	0.49 (0.04, 5.32)	0.55
Infection without focus	3 (1.0%)	1 (0.3%)	2.91 (0.30, 27.83)	0.35
Respiratory infection	15 (4.9%)	15 (5.1%)	0.97 (0.48, 1.95)	0.93
Varicella	0 (0.0%)	2 (0.7%)	—	—
**System Organ Class**				
Injury, poisoning and procedural complications	1 (0.3%)	0 (0.0%)	—	—
**Preferred Term**				
Fracture	1 (0.3%)	0 (0.0%)	—	—
**System Organ Class**				
Metabolism and nutrition disorders	0 (0.0%)	1 (0.3%)	—	—
**Preferred Term**				
Hypoglycemia	0 (0.0%)	1 (0.3%)	—	—

**System Organ Class**				
Pregnancy, puerperium and perinatal conditions	25 (8.2%)	23 (7.8%)	1.05 (0.61, 1.82)	0.85
**Preferred Term**				
Pregnancy loss	4 (1.3%)	3 (1.0%)	1.29 (0.29, 5.73)	0.73
Premature delivery	1 (0.3%)	0 (0.0%)	—	—
Pregnancy, puerperium and perinatal conditions, other	20 (6.6%)	20 (6.8%)	0.97 (0.53, 1.77)	0.92
Birth Asphyxia	14 (4.6%)	13 (4.4%)	1.05 (0.50, 2.19)	0.91
Low APGAR score	0 (0.0%)	1 (0.3%)	—	—
Low Birthweight	0 (0.0%)	2 (0.7%)	—	—
Meconium Aspiration	6 (2.0%)	4 (1.4%)	1.46 (0.41, 5.11)	0.56
**System Organ Class**				
Respiratory, thoracic and mediastinal disorders	2 (0.7%)	6 (2.0%)	0.32 (0.07, 1.59)	0.16
**Preferred Term**				
Respiratory, thoracic and mediastinal disorders, other	2 (0.7%)	6 (2.0%)	0.32 (0.07, 1.59)	0.16
Respiratory Distress Syndrome	2 (0.7%)	6 (2.0%)	0.32 (0.07, 1.59)	0.16
**System Organ Class**				
Skin and subcutaneous disorders	1 (0.3%)	2 (0.7%)	0.49 (0.04, 5.32)	0.55
**Preferred Term**				
Eczema	0 (0.0%)	1 (0.3%)	—	—
Rash-maculopapular	1 (0.3%)	1 (0.3%)	0.97 (0.06, 15.44)	0.98

CI denotes confidence interval, COVID-19 denotes coronavirus disease 2019.
* Adverse events (AEs) were coded using version 5.0 of the Common Terminology Criteria for Adverse Events (CTCAE) (US Department of Health Human Services, 27 November 2020) and use an appropriate Preferred Term and System Order Class for each AE verbatim term.
† These outcomes are analysed using a log-binomial regression model and display the estimate of the risk ratio of Ferric carboxymaltose versus standard of care (oral iron) and two-sided 95% confidence interval. The risk ratio excludes the stratification factor (site) in the log- model due to data separation issues.

‡ The P-values and two-sided 95% CIs presented have not been adjusted for multiple comparisons. The intervals should not be used in place of hypothesis testing.
§ The safety population includes all women who were treated, presented according to treated group. This includes 12 mothers with multiple pregnancies (8 in the ferric carboxymaltose group and 4 in the standard-of-care (oral iron) group. One woman who was randomised in the standard-of-care (oral iron) but later found to be not pregnant was excluded.
¶ The safety population includes all live-born neonates born to pregnant women who were treated, presented according to treated group of the mother. This includes 24 twins (16 in the Ferric carboxymaltose group and 8 in the standard-of-care (oral iron) group).

Supplementary Data Table 3. Subgroup analyses of effect of ferric carboxymaltose on maternal and neonate efficacy outcomes according to maternal baseline characteristics (intention-to-treat population)

Outcome, n/N(%) or mean(SD)	Ferric carboxy maltose	Standard of care – Oral Iron *	Prevalence Ratio or Mean Difference or Risk Ratio (95% CI)†‡	P-value	Ferric carboxy maltose	Standard of care – Oral Iron *	Prevalence Ratio or Mean Difference or Risk Ratio (95% CI)†‡	P-value	P-value for interaction
**Maternal efficacy outcomes (at 36 weeks' gestation or delivery, whichever came first)**									
	**Primigravida at baseline**				**Multigravida at baseline**				
Anaemia	41/94 (43.6%)	54/93 (58.1%)	0.75 (0.56, 1.00)	0.046	85/176 (48.3%)	116/178 (65.2%)	0.74 (0.62, 0.89)	0.0017	0.98
Venous Hb (g/dL) absolute change from baseline	1.95 (1.86)	1.36 (1.27)	0.52 (0.15, 0.88)	0.0054	1.64 (1.52)	1.04 (1.43)	0.52 (0.25, 0.78)	0.0001	0.99
	**HIV positive at baseline**				**HIV negative at baseline**				
Anaemia	32/59 (54.2%)	39/54 (72.2%)	0.75 (0.56, 1.00)	0.046	94/211 (44.5%)	131/217 (60.4%)	0.74 (0.62, 0.89)	0.0014	0.97
Venous Hb (g/dL) absolute change from baseline	1.86 (1.80)	1.35 (1.46)	0.60 (0.13, 1.07)	0.012	1.72 (1.61)	1.10 (1.36)	0.49 (0.25, 0.74)	<0.0001	0.70
	**Severe anaemia at baseline §**				**No severe anaemia at baseline §**				
Anaemia	6/13 (46.2%)	7/8 (87.5%)	–	–	119/256 (46.5%)	163/263 (62.0%)	–	–	–
Venous Hb (g/dL) absolute change from baseline	4.46 (2.38)	3.00 (1.95)	–	–	1.61 (1.48)	1.09 (1.32)	–	–	–
	**Iron deficient at baseline ¶**				**Not iron deficient at baseline ¶**				
Anaemia	81/180 (45.0%)	125/195 (64.1%)	0.69 (0.57, 0.83)	<0.0001	40/82 (48.8%)	39/65 (60.0%)	0.84 (0.63, 1.13)	0.25	0.26
Venous Hb (g/dL) absolute change from baseline	1.96 (1.62)	1.19 (1.37)	0.67 (0.42, 0.92)	<0.0001	1.29 (1.63)	0.91 (1.32)	0.28 (-0.13, 0.69)	0.18	0.11
	**Iron deficient anaemia at baseline ‖**				**No iron deficient anaemia at baseline ‖**				

Anaemia	79/167 (47.3%)	121/180 (67.2%)	0.69 (0.58, 0.84)	<0.0001	42/95 (44.2%)	43/80 (53.8%)	0.84 (0.62, 1.13)	0.26	0.29
Venous Hb (g/dL) absolute change from baseline	2.12 (1.55)	1.30 (1.18)	0.73 (0.47, 0.99)	<0.0001	1.11 (1.63)	0.71 (1.64)	0.22 (-0.15, 0.59)	0.24	0.029
	**Inflammation at baseline ****				**No inflammation at baseline ****				
Anaemia	34/77 (44.2%)	46/82 (56.1%)	0.81 (0.59, 1.11)	0.20	88/186 (47.3%)	119/179 (66.5%)	0.70 (0.59, 0.84)	<0.0001	0.42
Venous Hb (g/dL) absolute change from baseline	1.86 (1.75)	1.22 (1.62)	0.39 (0.00, 0.78)	0.052	1.70 (1.60)	1.06 (1.22)	0.62 (0.36, 0.88)	<0.0001	0.33
**Neonate efficacy outcomes (at delivery)**									
	**Primigravida at baseline**				**Multigravida at baseline**				
Birthweight (g)	2960.4 (386.2)	2934.9 (380.5)	29.52 (-99.29, 158.34)	0.65	2982.7 (453.9)	2971.2 (524.6)	1.18 (-94.30, 96.66)	0.98	0.73
Low birthweight (<2,500g)	13/92 (14.1%)	10/94 (10.6%)	1.29 (0.59, 2.79)	0.52	22/178 (12.4%)	29/179 (16.2%)	0.79 (0.47, 1.31)	0.36	0.30
	**HIV positive at baseline**				**HIV negative at baseline**				
Birthweight (g)	2977.0 (444.1)	2960.1 (608.6)	-7.24 (-175.73, 161.24)	0.93	2974.6 (428.7)	2958.3 (442.7)	15.85 (-69.63, 101.33)	0.72	0.81
Low birthweight (<2,500g)	8/62 (12.9%)	9/55 (16.4%)	0.84 (0.35, 2.05)	0.71	27/208 (13.0%)	30/218 (13.8%)	0.94 (0.58, 1.53)	0.81	0.83
	**Severe anaemia at baseline §**				**No severe anaemia at baseline §**				
Birthweight (g)	2798.5 (437.7)	2877.6 (492.6)	–	–	2986.8 (428.7)	2961.1 (479.8)	–	–	–
Low birthweight (<2,500g)	4/13 (30.8%)	2/8 (25.0%)	–	–	30/256 (11.7%)	37/265 (14.0%)	–	–	–
	**Iron deficient at baseline ¶**				**Not iron deficient at baseline ¶**				
Birthweight (g)	2994.8 (415.0)	2955.7 (464.1)	31.24 (-59.55, 122.03)	0.50	2929.7 (440.1)	2932.1 (536.6)	-1.60 (-146.58, 143.38)	0.98	0.71
Low birthweight (<2,500g)	20/176 (11.4%)	31/196 (15.8%)	0.74 (0.44, 1.24)	0.25	12/84 (14.3%)	8/68 (11.8%)	1.24 (0.54, 2.87)	0.61	0.29
	**Iron deficient anaemia at baseline ||**				**No iron deficient anaemia at baseline ||**				

Birthweight (g)	2985.8 (420.9)	2972.7 (453.1)	6.08 (-88.36, 100.51)	0.90	2952.9 (429.6)	2900.2 (540.8)	52.48 (-79.82, 184.78)	0.44	0.57
Low birthweight (<2,500g)	20/165 (12.1%)	27/180 (15.0%)	0.82 (0.48, 1.40)	0.47	12/95 (12.6%)	12/84 (14.3%)	0.91 (0.43, 1.92)	0.81	0.83
	**Inflammation at baseline ****				**No inflammation at baseline ****				
Birthweight (g)	3019.2 (409.4)	2809.7 (495.4)	173.05 (32.16, 313.95)	0.016	2960.2 (431.3)	3012.7 (464.8)	-48.67 (-140.96, 43.62)	0.30	0.0098
Low birthweight (<2,500g)	9/73 (12.3%)	17/82 (20.7%)	0.64 (0.31, 1.34)	0.24	23/188 (12.2%)	22/182 (12.1%)	1.02 (0.59, 1.77)	0.95	0.33

Data are presented as n/N (%), mean (SD). CI denotes confidence interval, Hb denotes haemoglobin.
Anaemia based on venous Hb indicates Hb<11.0 g/dL.
The Intention-to-treat basis indicates maternal and neonatal outcomes analysed according to randomly allocated group of the woman.
* One woman who was randomised in the standard-of-care (oral iron) but later found to be not pregnant was excluded.
† Maternal outcomes: A prevalence ratio and two-sided 95% confidence interval of Ferric carboxymaltose versus standard-of-care (oral iron) is displayed for anaemia at 36 weeks' gestation or delivery (whichever came first) using a using a mixed-effects logistic regression model from which the marginal estimate was obtained. Subgroup (main effect) and subgroup-by-treatment-by-visit interaction (as well as subgroup-by-treatment and subgroup-by-visit interaction) have been added to the model to evaluate where the treatment effect differs between subgroup categories. An absolute mean difference and two-sided 95% confidence interval for venous haemoglobin concentration between Ferric carboxymaltose and standard-of-care (oral iron) is displayed following fitting a linear regression model. Subgroup (main effect) and subgroup-by-treatment interactions terms have been added to the models to evaluate where the treatment effect differs between subgroup categories. The P-values and two-sided 95% CIs presented have not been adjusted for multiple comparisons. The intervals may not be used in place of hypothesis testing.
‡ Neonate outcomes: An absolute mean difference and two-sided 95% confidence interval for birthweight between Ferric carboxymaltose and standard-of-care (oral iron) is displayed following fitting a linear regression model. A risk ratio and two-sided 95% confidence interval of Ferric carboxymaltose versus standard of care is displayed for low birthweight following analyses using a log-binomial regression model. Subgroup (main effect) and subgroup-by-treatment interactions terms have been added to the model to evaluate where the treatment effect differs between subgroup categories. Birthweight and low birthweight were multiply imputed before analyses. The P-values and two-sided 95% CIs presented have not been adjusted for multiple comparisons. The intervals may not be used in place of hypothesis testing.
§ Severe anaemia indicates Hb <7g/dL. Severe anaemia data is missing for one participant in the Ferric carboxymaltose group and no participants in the standard-of-care (oral iron) group. No statistical analysis was performed due to the small sample size in the subgroup of those with severe anaemia at baseline (n=23).
¶ Iron deficient indicates serum ferritin <15ug/L or serum ferritin <30ug/L if C-reactive protein >5mg/L. Iron deficient data is missing for nine participants in the Ferric carboxymaltose group and 11 participants in the standard-of-care (oral iron) group.

|| Iron deficiency anaemia indicates Hb <11g/dL and serum ferritin <15 ug/L or ferritin <30 ug/L if C-reactive protein >5mg/L. Iron deficiency anaemia data are missing for nine participants in the Ferric carboxymaltose group and 11 participants in the standard-of-care (oral iron) group.
** Inflammation indicates C-reactive protein >5mg/L. Inflammation data is missing for eight participants in the Ferric carboxymaltose group and 10 participants in the standard-of-care (oral iron) group.

Supplementary Data Table 4. Additional analyses on maternal and neonate outcomes*

Outcome/Visit	Additional analysis	Prevalence Ratio or Mean Difference or Geometric Mean Ratio or Risk Ratio (95% CI)	P-value ||||||
**Maternal efficacy outcomes**			
**Primary outcome**			
Anaemia at 36 weeks' gestation or at delivery (whichever came first) †	Maternal first adjusted model, ITT§	0.74 (0.64, 0.87) ***	0.0002
	Maternal second adjusted model, ITT¶	0.71 (0.60, 0.82) ***	<0.0001
	Adjusted for site, PP	0.73 (0.63, 0.86) ***	0.0002
	Maternal second adjusted model, PP¶	0.69 (0.59, 0.81) ***	<0.0001
	Adjusted for site, multiple imputation (delta -2.0) ¶¶	0.78 (0.67, 0.89) ***	0.0004
	Adjusted for site, multiple imputation (delta -1.5) ¶¶	0.77 (0.67, 0.89) ***	0.0004
	Adjusted for site, multiple imputation (delta -1.0) ¶¶	0.77 (0.66, 0.89) ***	0.0004
	Adjusted for site, multiple imputation (delta -0.5) ¶¶	0.76 (0.65, 0.88) ***	0.0003
	Adjusted for site, multiple imputation (delta 0) ¶¶	0.75 (0.64, 0.87) ***	0.0002
	Adjusted for site, multiple imputation (delta +0.5) ¶¶	0.73 (0.63, 0.86) ***	0.0001
	Adjusted for site, multiple imputation (delta +1.0) ¶¶	0.73 (0.62, 0.85) ***	<0.0001
	Adjusted for site, multiple imputation (delta +1.5) ¶¶	0.72 (0.62, 0.85) ***	<0.0001
	Adjusted for site, multiple imputation (delta +2.0) ¶¶	0.72 (0.62, 0.85) ***	0.0001
**Key secondary outcomes**			
Venous Hb (g/dL) absolute change from baseline			
36 weeks gestation or at delivery (whichever came first)	Maternal first adjusted model, ITT§	0.52 (0.30, 0.73) †††	<0.0001
	Maternal second adjusted model, ITT||	0.54 (0.32, 0.76) †††	<0.0001
	Adjusted for site, PP	0.52 (0.31, 0.74) †††	<0.0001
	Maternal second adjusted model, PP||	0.58 (0.37, 0.80) †††	<0.0001
	Adjusted for site, multiple imputation (delta -2.0) ¶¶	0.48 (0.25, 0.72) †††	<0.0001
	Adjusted for site, multiple imputation (delta -1.5) ¶¶	0.49 (0.26, 0.72) †††	<0.0001
	Adjusted for site, multiple imputation (delta -1.0) ¶¶	0.50 (0.28, 0.72) †††	<0.0001
	Adjusted for site, multiple imputation (delta -0.5) ¶¶	0.51 (0.29, 0.73) †††	<0.0001
	Adjusted for site, multiple imputation (delta 0) ¶¶	0.52 (0.30, 0.73) †††	<0.0001
	Adjusted for site, multiple imputation (delta +0.5) ¶¶	0.52 (0.31, 0.74) †††	<0.0001
	Adjusted for site, multiple imputation (delta +1.0) ¶¶	0.53 (0.31, 0.75) †††	<0.0001
	Adjusted for site, multiple imputation (delta +1.5) ¶¶	0.54 (0.31, 0.77) †††	<0.0001

	Adjusted for site, multiple imputation (delta +2.0) ¶¶	0.55 (0.31, 0.78) †††	<0.0001
1 month post-partum	Maternal first adjusted model, ITT§	0.67 (0.43, 0.90) †††	<0.0001
	Maternal second adjusted model, ITT‖	0.71 (0.47, 0.95) †††	<0.0001
	Adjusted for site, PP	0.67 (0.43, 0.90) †††	<0.0001
	Maternal second adjusted model, PP‖	0.78 (0.55, 1.00) †††	<0.0001
	Adjusted for site, multiple imputation (delta -2.0) ¶¶	0.66 (0.38, 0.94) †††	<0.0001
	Adjusted for site, multiple imputation (delta -1.5) ¶¶	0.67 (0.40, 0.93) †††	<0.0001
	Adjusted for site, multiple imputation (delta -1.0) ¶¶	0.67 (0.42, 0.93) †††	<0.0001
	Adjusted for site, multiple imputation (delta -0.5) ¶¶	0.68 (0.43, 0.93) †††	<0.0001
	Adjusted for site, multiple imputation (delta 0) ¶¶	0.69 (0.44, 0.93) †††	<0.0001
	Adjusted for site, multiple imputation (delta +0.5) ¶¶	0.69 (0.45, 0.94) †††	<0.0001
	Adjusted for site, multiple imputation (delta +1.0) ¶¶	0.70 (0.45, 0.95) †††	<0.0001
	Adjusted for site, multiple imputation (delta +1.5) ¶¶	0.71 (0.45, 0.97) †††	<0.0001
	Adjusted for site, multiple imputation (delta +2.0) ¶¶	0.71 (0.44, 0.99) †††	<0.0001
Ferritin (µg/L) relative change from baseline			
36 weeks gestation or at delivery (whichever came first)	Maternal first adjusted model, ITT§	6.07 (5.24, 7.03) ‡‡‡	<0.0001
	Maternal second adjusted model, ITT¶	5.99 (5.13, 6.98) ‡‡‡	<0.0001
	Adjusted for site, PP	5.91 (5.09, 6.86) ‡‡‡	<0.0001
	Maternal second adjusted model, PP¶	5.88 (5.03, 6.87) ‡‡‡	<0.0001
1 month post-partum	Maternal first adjusted model, ITT§	3.21 (2.78, 3.72) ‡‡‡	<0.0001
	Maternal second adjusted model, ITT¶	3.20 (2.74, 3.74) ‡‡‡	<0.0001
	Adjusted for site, PP	3.23 (2.79, 3.74) ‡‡‡	<0.0001
	Maternal second adjusted model, PP¶	3.21 (2.74, 3.76) ‡‡‡	<0.0001
**Other secondary outcomes**			
Anaemia†			
36 weeks gestation	Adjusted for site, ITT	0.81 (0.70, 0.94) ***	0.0062
	Adjusted for site, PP	0.80 (0.69, 0.94) ***	0.0054
Delivery	Maternal first adjusted model, ITT§	0.61 (0.46, 0.81) ***	0.0006
	Maternal second adjusted model, ITT¶	0.56 (0.42, 0.75) ***	<0.0001
	Adjusted for site, PP	0.63 (0.47, 0.84) ***	0.0019
	Maternal second adjusted model, PP¶	0.58 (0.43, 0.77) ***	0.0002
	Adjusted for site, multiple imputation (delta -2.0) ¶¶	0.74 (0.59, 0.94) ***	0.013
	Adjusted for site, multiple imputation (delta -1.5) ¶¶	0.71 (0.56, 0.91) ***	0.0076
	Adjusted for site, multiple imputation (delta -1.0) ¶¶	0.68 (0.52, 0.88) ***	0.0036
	Adjusted for site, multiple imputation (delta -0.5) ¶¶	0.65 (0.49, 0.85) ***	0.0018
	Adjusted for site, multiple imputation (delta 0) ¶¶	0.62 (0.46, 0.82) ***	0.0009

	Adjusted for site, multiple imputation (delta +0.5) ¶¶	0.60 (0.45, 0.80) ***	0.0006
	Adjusted for site, multiple imputation (delta +1.0) ¶¶	0.58 (0.43, 0.79) ***	0.0004
	Adjusted for site, multiple imputation (delta +1.5) ¶¶	0.57 (0.42, 0.77) ***	0.0002
	Adjusted for site, multiple imputation (delta +2.0) ¶¶	0.57 (0.42, 0.77) ***	0.0002
1 month post-partum	Maternal first adjusted model, ITT§	0.62 (0.51, 0.75) ***	<0.0001
	Maternal second adjusted model, ITT¶	0.59 (0.48, 0.71) ***	<0.0001
	Adjusted for site, PP	0.62 (0.51, 0.76) ***	<0.0001
	Maternal second adjusted model, PP¶	0.59 (0.49, 0.71) ***	<0.0001
	Adjusted for site, multiple imputation (delta -2.0) ¶¶	0.72 (0.62, 0.84) ***	<0.0001
	Adjusted for site, multiple imputation (delta -1.5) ¶¶	0.70 (0.60, 0.82) ***	<0.0001
	Adjusted for site, multiple imputation (delta -1.0) ¶¶	0.67 (0.57, 0.80) ***	<0.0001
	Adjusted for site, multiple imputation (delta -0.5) ¶¶	0.65 (0.54, 0.78) ***	<0.0001
	Adjusted for site, multiple imputation (delta 0) ¶¶	0.63 (0.51, 0.77) ***	<0.0001
	Adjusted for site, multiple imputation (delta +0.5) ¶¶	0.61 (0.49, 0.75) ***	<0.0001
	Adjusted for site, multiple imputation (delta +1.0) ¶¶	0.59 (0.48, 0.73) ***	<0.0001
	Adjusted for site, multiple imputation (delta +1.5) ¶¶	0.59 (0.47, 0.73) ***	<0.0001
	Adjusted for site, multiple imputation (delta +2.0) ¶¶	0.59 (0.48, 0.73) ***	<0.0001
Moderate/Severe anaemia†			
36 weeks gestation or at delivery (whichever came first)	Maternal first adjusted model, ITT§	0.60 (0.45, 0.80) ***	0.0004
	Maternal second adjusted model, ITT¶	0.56 (0.42, 0.74) ***	<0.0001
	Adjusted for site, PP	0.60 (0.45, 0.80) ***	0.0006
	Maternal second adjusted model, PP¶	0.55 (0.41, 0.73) ***	<0.0001
Delivery	Maternal first adjusted model, ITT§	0.47 (0.28, 0.78) ***	0.0033
	Maternal second adjusted model, ITT¶	0.44 (0.27, 0.72) ***	0.0012
	Adjusted for site, PP	0.50 (0.30, 0.84) ***	0.0087
	Maternal second adjusted model, PP¶	0.46 (0.28, 0.76) ***	0.0025
1 month post-partum	Maternal first adjusted model, ITT§	0.50 (0.33, 0.75) ***	0.0010
	Maternal second adjusted model, ITT¶	0.43 (0.28, 0.65) ***	<0.0001
	Adjusted for site, PP	0.49 (0.32, 0.76) ***	0.0012
	Maternal second adjusted model, PP¶	0.43 (0.28, 0.65) ***	<0.0001
Venous Hb (g/dL) absolute change from baseline			
Delivery	Maternal first adjusted model, ITT§	0.46 (0.19, 0.73) †††	0.0009
	Maternal second adjusted model, ITT‖	0.45 (0.18, 0.73) †††	0.0013
	Adjusted for site, PP	0.44 (0.17, 0.71) †††	0.0017
	Maternal second adjusted model, PP‖	0.50 (0.28, 0.72) †††	<0.0001
	Adjusted for site, multiple imputation (delta -2.0) ¶¶	0.38 (0.09, 0.68) †††	0.011

	Adjusted for site, multiple imputation (delta -1.5) ¶¶	0.40 (0.12, 0.69) †††	0.0060
	Adjusted for site, multiple imputation (delta -1.0) ¶¶	0.42 (0.14, 0.70) †††	0.0032
	Adjusted for site, multiple imputation (delta -0.5) ¶¶	0.44 (0.17, 0.72) †††	0.0017
	Adjusted for site, multiple imputation (delta 0) ¶¶	0.46 (0.19, 0.73) †††	0.0010
	Adjusted for site, multiple imputation (delta +0.5) ¶¶	0.48 (0.20, 0.75) †††	0.0006
	Adjusted for site, multiple imputation (delta +1.0) ¶¶	0.50 (0.22, 0.78) †††	0.0004
	Adjusted for site, multiple imputation (delta +1.5) ¶¶	0.52 (0.23, 0.80) †††	0.0004
	Adjusted for site, multiple imputation (delta +2.0) ¶¶	0.54 (0.25, 0.83) †††	0.0003
Ferritin (µg/L) relative change from baseline			
Delivery	Maternal first adjusted model, ITT§	4.26 (3.65, 4.96) ‡‡‡	<0.0001
	Maternal second adjusted model, ITT¶	4.17 (3.55, 4.90) ‡‡‡	<0.0001
	Adjusted for site, PP	4.27 (3.66, 5.00) ‡‡‡	<0.0001
	Maternal second adjusted model, PP¶	4.23 (3.60, 4.99) ‡‡‡	<0.0001
Iron deficiency ‡			
36 weeks gestation or at delivery (whichever came first)	Maternal first adjusted model, ITT§	0.08 (0.04, 0.17) ***	<0.0001
	Maternal second adjusted model, ITT¶	0.07 (0.03, 0.16) ***	<0.0001
	Adjusted for site, PP	0.08 (0.04, 0.17) ***	<0.0001
	Maternal second adjusted model, PP¶	0.07 (0.03, 0.16) ***	<0.0001
Delivery	Maternal first adjusted model, ITT§	0.20 (0.12, 0.33) ***	<0.0001
	Maternal second adjusted model, ITT¶	0.20 (0.12, 0.32) ***	<0.0001
	Adjusted for site, PP	0.20 (0.12, 0.33) ***	<0.0001
	Maternal second adjusted model, PP¶	0.18 (0.11, 0.31) ***	<0.0001
1 month post-partum	Maternal first adjusted model, ITT§	0.16 (0.08, 0.31) ***	<0.0001
	Maternal second adjusted model, ITT¶	0.14 (0.07, 0.28) ***	<0.0001
	Adjusted for site, PP	0.15 (0.08, 0.30) ***	<0.0001
	Maternal second adjusted model, PP¶	0.14 (0.07, 0.28) ***	<0.0001
Iron deficiency anaemia ‡			
36 weeks gestation or at delivery (whichever came first)	Maternal first adjusted model, ITT§	0.02 (0.00, 0.13) ***	<0.0001
	Maternal second adjusted model, ITT¶	0.02 (0.00, 0.12) ***	<0.0001
	Adjusted for site, PP	0.02 (0.00, 0.13) ***	<0.0001
	Maternal second adjusted model, PP¶	0.02 (0.00, 0.12) ***	<0.0001
Delivery	Maternal first adjusted model, ITT§	0.04 (0.01, 0.23) ***	0.0004
	Maternal second adjusted model, ITT¶	0.04 (0.01, 0.21) ***	0.0002
	Adjusted for site, PP	0.04 (0.01, 0.24) ***	0.0005
	Maternal second adjusted model, PP¶	0.04 (0.01, 0.21) ***	0.0002
1 month post-partum	Maternal first adjusted model§, ITT	0.17 (0.08, 0.35) ***	<0.0001

	Maternal second adjusted model, ITT ¶	0.15 (0.07, 0.33) ***	<0.0001
	Adjusted for site, PP	0.16 (0.07, 0.34) ***	<0.0001
	Maternal second adjusted model, PP¶	0.16 (0.08, 0.34) ***	<0.0001
**Neonate efficacy outcomes**			
**Primary outcome**			
Birthweight (g)	Neonate first adjusted model, ITT **	7.4 (-68.8, 83.5) §§§	0.85
	Neonate second adjusted model, ITT ††	8.1 (-68.2, 84.3) §§§	0.84
	Neonate third adjusted model, ITT	18.4 (-59.1, 95.9) §§§	0.64
	Adjusted for site, ITT	14.4 (-62.1, 91.0) §§§	0.71
	Adjusted for site, PP	30.7 (-43.7, 105.2) §§§	0.42
	Neonate full adjusted model, PP	34.1 (-41.1, 109.2) §§§	0.37
	GEE model||||	14.4 (-65.1, 94.0) §§§	0.72
**Secondary outcomes**			
Venous Hb (g/dL) at 1 month of age	Neonate first adjusted model, ITT **	-0.09 (-0.43, 0.25) §§§	0.61
	Neonate second adjusted model, ITT ††	-0.09 (-0.44, 0.25) §§§	0.60
	Neonate third adjusted model, ITT	-0.11 (-0.46, 0.25) §§§	0.56
	Adjusted for site, PP	-0.05 (-0.39, 0.30) §§§	0.78
	Neonate full adjusted model, PP	-0.05 (-0.41, 0.32) §§§	0.80
Weight (g) at 1 month of age	Neonate first adjusted model, ITT **	14.2 (-105.9, 134.4) §§§	0.82
	Neonate second adjusted model, ITT ††	10.5 (-109.6, 130.6) §§§	0.86
	Neonate third adjusted model, ITT	18.6 (-105.0, 142.2) §§§	0.77
	Adjusted for site, PP	53.8 (-65.3, 173.0) §§§	0.38
	Neonate full adjusted model, PP	28.1 (-92.9, 149.1) §§§	0.65
**Other secondary outcomes**			
Birth length (cm)	Neonate first adjusted model, ITT **	-0.01 (-0.50, 0.48) §§§	0.98
	Neonate second adjusted model††	-0.02 (-0.51, 0.47) §§§	0.94
	Neonate third adjusted model‡‡	-0.02 (-0.52, 0.48) §§§	0.93
	ITT§§	0.01 (-0.48, 0.50) §§§	0.97
	PP§§	0.03 (-0.46, 0.53) §§§	0.89
	Neonate full adjusted model, PP‡‡	0.00 (-0.50, 0.51) §§§	0.99
Length (cm)	Neonate first adjusted model, ITT **	0.31 (-0.25, 0.86) §§§	0.28
	Neonate second adjusted model, ITT ††	0.31 (-0.25, 0.87) §§§	0.27
	Neonate third adjusted model, ITT ‡‡	0.29 (-0.28, 0.87) §§§	0.31
	Adjusted for site, PP	0.42 (-0.14, 0.97) §§§	0.14
	Neonate full adjusted model, PP‡‡	0.35 (-0.22, 0.92) §§§	0.23
Low birthweight (<2,500g)	Neonate first adjusted model, ITT **	0.94 (0.62, 1.43) ¶¶¶	0.77

	Neonate second adjusted model, ITT††<br>Neonate third adjusted model, ITT‡‡<br>ITT§§<br>PP§§<br>Neonate full adjusted model, PP‡‡	0.91 (0.59, 1.39) ¶¶¶<br>0.84 (0.55, 1.30) ¶¶¶<br>0.91 (0.59, 1.39) ¶¶¶<br>0.79 (0.48, 1.29) ¶¶¶<br>0.72 (0.44, 1.19) ¶¶¶	0.66<br>0.45<br>0.65<br>0.35<br>0.20
Stillbirth	PP§§	1.33 (0.30, 5.88) ¶¶¶	0.71
Weight for age z score	Neonate first adjusted model, ITT**<br>Neonate second adjusted model, ITT††<br>Neonate third adjusted model, ITT‡‡<br>Adjusted for site, PP<br>Neonate full adjusted model, PP‡‡	0.04 (-0.18, 0.26) §§§<br>0.03 (-0.19, 0.26) §§§<br>0.05 (-0.18, 0.28) §§§<br>0.06 (-0.15, 0.27) §§§<br>0.06 (-0.16, 0.28) §§§	0.73<br>0.77<br>0.68<br>0.58<br>0.59
Length for age z score	Neonate first adjusted model, ITT**<br>Neonate second adjusted model, ITT††<br>Neonate third adjusted model, ITT‡‡<br>Adjusted for site, PP<br>Neonate full adjusted model, PP‡‡	0.15 (-0.13, 0.43) §§§<br>0.15 (-0.13, 0.44) §§§<br>0.14 (-0.15, 0.44) §§§<br>0.19 (-0.10, 0.47) §§§<br>0.17 (-0.12, 0.46) §§§	0.29<br>0.29<br>0.33<br>0.19<br>0.25
Weight for length z score	Neonate first adjusted model, ITT**<br>Neonate second adjusted model, ITT††<br>Neonate third adjusted model, ITT‡‡<br>Adjusted for site, PP<br>Neonate full adjusted model, PP‡‡	-0.24 (-0.57, 0.09) §§§<br>-0.24 (-0.57, 0.09) §§§<br>-0.20 (-0.54, 0.14) §§§<br>-0.22 (-0.56, 0.13) §§§<br>-0.18 (-0.54, 0.17) §§§	0.16<br>0.16<br>0.25<br>0.22<br>0.31

*One woman who was randomised in the standard-of-care (oral iron) but later found to be not pregnant was excluded. The Intention-to-treat basis indicates maternal and neonatal outcomes analysed according to randomly allocated group of the woman. The Per-protocol population excludes 12 women and 24 neonates due to multiple pregnancy/twins.

CI denotes confidence interval, GEE denotes generalised estimating equation, Hb denotes haemoglobin, ITT denotes intention-to-treat, PP denotes per-protocol, HIV denotes human immunodeficiency virus.

† Anaemia indicates venous Hb <11.0g/dL up to and including delivery and venous Hb <12.0g/dL postpartum. Moderate/Severe anaemia indicates venous Hb <10.0g/dL up to and including delivery and venous Hb <11.0g/dL postpartum.

‡ Iron deficient indicates serum ferritin <15ug/L or serum ferritin <30ug/L if C-reactive protein >5mg/L, and iron deficient anaemia indicates Hb <11.0g/dL and serum ferritin<15 ug/L or serum ferritin <30 ug/L if C-reactive protein >5mg/L.

§ Adjusted for site, and gravidity (primigravid or. multigravid).

¶ Adjusted for site, gravidity (primigravid or multigravid), inflammation status at baseline, iron deficient status at baseline, venous Hb at baseline (continuous), and HIV positive status at baseline.

|| Adjusted for site, gravidity (primigravid or multigravid), inflammation status at baseline, iron deficient status at baseline, and HIV positive status at baseline.

** Neonate first adjusted model: Adjusted for site, sex (female or male)

†† Neonatal second adjusted model: Adjusted for site, sex (female or male) and maternal venous Hb at baseline (continuous).
‡‡ Neonatal third/full adjusted model: Adjusted for site, sex (female or male), maternal venous Hb at baseline (continuous) and maternal iron deficient status at baseline (due to observed imbalance between groups at baseline).
§§ The risk ratio of Ferric carboxymaltose versus standard of care (oral iron) displayed for low birthweight and stillbirth excludes the stratification factor (site) in the log-binomial regression model due to data separation issues.
¶¶ Missing maternal venous Hb data were multiply imputed according to treatment group using chained equations. The imputation model included site, gravidity (primigravid or multigravid), inflammation status at baseline, iron deficient status at baseline, venous Hb at baseline (continuous), and HIV positive status at baseline. A total of 26 imputations were used, as approximately 26% of the participants had missing data in venous Hb values at any one time-point. A delta-adjustment to the imputed values was added before obtaining the multiple imputation estimate, CI and P-value. The shifts were assumed as -2, -1.5, -1, -0.5, 0, +0.5, +1, +1.5, +2 g/dL for all imputed values in both treatment groups.
|||| An analysis consisting of a linear generalised estimating equation (GEE) model with an independence (with robust variance estimation) working correlation structure to account for clustering of twin pairs among those known to be liveborn. Multiple births occurred in ~1% of the maternal ITT population.
*** A prevalence ratio and two-sided 95% confidence interval of Ferric carboxymaltose versus standard-of-care (oral iron) is obtained using a mixed-effects logistic regression model from which the marginal estimate was obtained.
††† An absolute mean difference and two-sided 95% confidence interval of Ferric carboxymaltose and standard-of-care (oral iron) is presented following analyses using a likelihood-based longitudinal data analysis model.
‡‡‡A geometric mean ratio and two-sided 95% confidence interval of Ferric carboxymaltose versus standard-of-care (oral iron) is displayed for ferritin concentration after a log base e transformation due to skewness.
§§§ An absolute mean difference and two-sided 95% confidence interval between ferric carboxymaltose and standard-of-care (oral iron) is displayed following fitting a linear regression model.
¶¶¶ A risk ratio and two-sided 95% confidence interval of Ferric carboxymaltose iron versus standard of care (oral iron) following analyses using a log-binomial regression model.
|||||| The P-values and two-sided 95% CIs presented have not been adjusted for multiple comparisons. The intervals may not be used in place of hypothesis testing.

CONSORT 2010 checklist of information to include when reporting a randomised trial*

Section/Topic	Item No	Checklist item	Reported on page No
**Title and abstract**			
	1a	Identification as a randomised trial in the title	1
	1b	Structured summary of trial design, methods, results, and conclusions (for specific guidance see CONSORT for abstracts)	4
**Introduction**			
Background and objectives	2a	Scientific background and explanation of rationale	5-6
	2b	Specific objectives or hypotheses	6
**Methods**			
Trial design	3a	Description of trial design (such as parallel, factorial) including allocation ratio	32
	3b	Important changes to methods after trial commencement (such as eligibility criteria), with reasons	S384
Participants	4a	Eligibility criteria for participants	32-33
	4b	Settings and locations where the data were collected	33
Interventions	5	The interventions for each group with sufficient details to allow replication, including how and when they were actually administered	34
Outcomes	6a	Completely defined pre-specified primary and secondary outcome measures, including how and when they were assessed	35-36
	6b	Any changes to trial outcomes after the trial commenced, with reasons	S384
Sample size	7a	How sample size was determined	36
	7b	When applicable, explanation of any interim analyses and stopping guidelines	36
Randomisation:			
Sequence generation	8a	Method used to generate the random allocation sequence	33
	8b	Type of randomisation; details of any restriction (such as blocking and block size)	33
Allocation concealment mechanism	9	Mechanism used to implement the random allocation sequence (such as sequentially numbered containers), describing any steps taken to conceal the sequence until interventions were assigned	33
Implementation	10	Who generated the random allocation sequence, who enrolled participants, and who assigned participants to interventions	33
Blinding	11a	If done, who was blinded after assignment to interventions (for example, participants, care providers, those	33-34

		assessing outcomes) and how	
	11b	If relevant, description of the similarity of interventions	N/A
Statistical methods	12a	Statistical methods used to compare groups for primary and secondary outcomes	36-37
	12b	Methods for additional analyses, such as subgroup analyses and adjusted analyses	37
**Results**			
Participant flow (a diagram is strongly recommended)	13a	For each group, the numbers of participants who were randomly assigned, received intended treatment, and were analysed for the primary outcome	7, Figure 1
	13b	For each group, losses and exclusions after randomisation, together with reasons	7, Figure 1
Recruitment	14a	Dates defining the periods of recruitment and follow-up	7
	14b	Why the trial ended or was stopped	N/A
Baseline data	15	A table showing baseline demographic and clinical characteristics for each group	Table 1, Table S1
Numbers analysed	16	For each group, number of participants (denominator) included in each analysis and whether the analysis was by original assigned groups	Table 2
Outcomes and estimation	17a	For each primary and secondary outcome, results for each group, and the estimated effect size and its precision (such as 95% confidence interval)	7-8, Table 2
	17b	For binary outcomes, presentation of both absolute and relative effect sizes is recommended	Relative effect presented
Ancillary analyses	18	Results of any other analyses performed, including subgroup analyses and adjusted analyses, distinguishing pre-specified from exploratory	9-10, Figure 2, Table S2, Table S5
Harms	19	All important harms or unintended effects in each group (for specific guidance see CONSORT for harms)	8-9, Table 3, Table S3, Table S4
**Discussion**			
Limitations	20	Trial limitations, addressing sources of potential bias, imprecision, and, if relevant, multiplicity of analyses	14
Generalisability	21	Generalisability (external validity, applicability) of the trial findings	12-13
Interpretation	22	Interpretation consistent with results, balancing benefits and harms, and considering other relevant evidence	11-15
**Other information**			
Registration	23	Registration number and name of trial registry	4, 32

Protocol	24	Where the full trial protocol can be accessed, if available	Supplement
Funding	25	Sources of funding and other support (such as supply of drugs), role of funders	15

Citation: Schulz KF, Altman DG, Moher D, for the CONSORT Group. CONSORT 2010 Statement: updated guidelines for reporting parallel group randomised trials. BMC Medicine. 2010;8:18.
© 2010 Schulz et al. This is an Open Access article distributed under the terms of the Creative Commons Attribution License (http://creativecommons.org/licenses/by/2.0), which permits unrestricted use, distribution, and reproduction in any medium, provided the original work is properly cited.

*We strongly recommend reading this statement in conjunction with the CONSORT 2010 Explanation and Elaboration for important clarifications on all the items. If relevant, we also recommend reading CONSORT extensions for cluster randomised trials, non-inferiority and equivalence trials, non-pharmacological treatments, herbal interventions, and pragmatic trials. Additional extensions are forthcoming: for those and for up-to-date references relevant to this checklist, see www.consort-statement.org.

REVAMP-TT Protocol version 1.1

Protocol used at trial opening

A randomised controlled trial of the effect of intravenous iron on anaemia in Malawian pregnant women in their third-trimester

Short Title: REVAMP-TT

Study Identifiers:

NHSRC:	PMRA:	ANZCTR
**20/11/2622**	#####	#####

Co-Principal Investigators:

- Professor Kamija Phiri, College of Medicine, University of Malawi and Training and Research Unit of Excellence, P.O Box 30538, Chichiri, Blantyre 3, Malawi; Mobile: +265 999957 048; E-mail: director@true.mw
- Associate Professor Sant-Rayn Pasricha, Walter and Eliza Hall Institute of Medical Research, 1G Royal Parade, Parkville Victoria 3052, Australia; Mobile: +61 407 141 570; E-mail: pasricha.s@wehi.edu.au

Co-Investigators: See Page 13

Funder: Bill & Melinda Gates Foundation

Revision chronology:

Date	Protocol Version	Details of Changes	Authors (see page 13)	Signature Principal Investigators
29 Aug 2020	*1.0 Original*		*SP, KP, RA, MM, LL, SB*	[redacted]
26th Mar 2021	*1.1*	• Trial arms reduced to two after the manufacturer of IIM could not supply the IP<br>• Co-investigator list revised to add Dr Rebecca Harding		[redacted]

Confidentiality Statement: This document contains confidential information that must not be disclosed to anyone other than the sponsor, the investigator team, host institution, relevant ethics committee and regulatory authorities

Table of Contents

1. ABBREVIATIONS 7

1. REVIATIONS 7

2. TITLE OF RESEARCH PROPOSAL 8

3. INVESTIGATORS AND INSTITUTIONAL AFFILIATIONS 8

4. PROTOCOL SUMMARIES 9

4.1. TRIAL REGISTRATION DATA 9
4.2. EXECUTIVE SUMMARY 13
4.3. SCHEDULE OF ACTIVITIES 16

5. INTRODUCTION 20

5.1. BACKGROUND 20
5.1.1. ANAEMIA DURING PREGNANCY REMAINS A CRITICAL GLOBAL HEALTH PROBLEM 20
5.1.2. LOW BIRTH WEIGHT AND PRE-TERM BIRTH HAVE CRITICAL IMPLICATIONS FOR MOTHER AND BABY 20
5.1.3. IRON CAN BENEFIT ANTENATAL ANAEMIA 20
5.1.4. UPTAKE AND ADHERENCE TO ORAL IRON THERAPY DURING PREGNANCY IS INADEQUATE IN THE FIELD 21
5.1.5. FERRIC CARBOXYMALTOSE: A NEW INTRAVENOUS IRON PREPARATIONS 21
5.1.6. INTRAVENOUS IRON AND BENEFITS FOR THE POSTPARTUM PERIOD 30

6. TRIAL OBJECTIVES AND OUTCOMES 31

6.1. OBJECTIVES 31
6.1.1. BROAD OBJECTIVE 31
6.1.2. SPECIFIC OBJECTIVES 31
6.2. OUTCOMES 31
6.2.1. PRIMARY OUTCOME 31
6.2.2. SECONDARY OUTCOME (MATERNAL BENEFITS) 32
6.2.3. SECONDARY OUTCOMES (NEONATE/INFANT BENEFITS) 32
6.2.4. SECONDARY OUTCOME (MATERNAL SAFETY) 32
6.2.5. SECONDARY OUTCOME (CHILD SAFETY) 33

7. STUDY DESIGN 33

8. PARTICIPANT INCLUSION AND EXCLUSION CRITERIA 33

9. STUDY SITES 34

10. TRIAL INTERVENTIONS 36

10.1. ALLOCATION TO TREATMENT 36
10.2. BREAKING THE BLIND 36
10.2.1. PARTICIPANT LEVEL....36
10.2.2. STUDY LEVEL 36

11. STUDY PROCEDURES 36

11.1. SCREENING – VISIT 0 [DAY 0]....37
11.1.1. BLOOD SAMPLING 38
11.1.2. STUDY ARM ALLOCATION 38
11.1.3. STUDY DRUG ADMINISTRATION 38
11.1.4. SOCIO-ECONOMIC DATA COLLECTION....39
11.1.5. OTHER PROCEDURES 39
11.2. 36-WEEK FOLLOW-UP [± 2 DAYS] – VISIT 4 39
11.2.1. BLOOD SAMPLING 39
11.2.2. REPRODUCTIVE TRACT MICROBIOTA 39
11.2.3. ORAL IRON ARM - COMPLIANCE 40
11.2.4. MATERNAL PSYCHOLOGICAL HEALTH 40
11.3. DELIVERY [+1 DAY] – VISIT 7 40
11.3.1. BLOOD SAMPLING 40
11.3.2. PLACENTAL TISSUE - HISTOLOGY AND STORAGE....40
11.3.3. INFANT NEURODEVELOPMENT....41
11.4. 28 DAYS POSTPARTUM [± 2 DAYS] – VISIT 8....41
11.4.1. BLOOD SAMPLING 41
11.4.2. REPRODUCTIVE TRACT MICROBIOTA 41
11.4.3. BREASTMILK....41
11.4.4. CHILD ANTHROPOMETRY....42
11.4.5. CHILD VACCINATION AND VITAMIN A SUPPLEMENTATION STATUS....42
11.4.6. INFANT NEURODEVELOPMENT....42
11.5. 3 MONTHS POSTPARTUM [± 14 DAYS] – VISIT 9 42
11.5.1. BLOOD SAMPLING 42
11.5.2. CHILD ANTHROPOMETRY....42
11.5.3. CHILD VACCINATION AND VITAMIN A SUPPLEMENTATION STATUS....42
11.5.4. MATERNAL PSYCHOLOGICAL HEALTH 43
11.6. 6 MONTHS POSTPARTUM [± 14 DAYS] – VISIT 10 43
11.6.1. BLOOD SAMPLING 43
11.6.2. BREASTMILK....43
11.6.3. CHILD ANTHROPOMETRY....43
11.6.4. CHILD VACCINATION AND VITAMIN A SUPPLEMENTATION STATUS....43
11.6.5. INFANT NEURODEVELOPMENT....43
11.7. 9 MONTHS POSTPARTUM [± 14 DAYS] – VISIT 11 44
11.7.1. BLOOD SAMPLING 44
11.7.2. CHILD ANTHROPOMETRY....44
11.7.3. CHILD VACCINATION AND VITAMIN A SUPPLEMENTATION STATUS....44
11.8. 12 MONTHS POSTPARTUM [± 14 DAYS] – VISIT 12 44
11.8.1. BLOOD SAMPLING 44
11.8.2. REPRODUCTIVE TRACT MICROBIOTA 45
11.8.3. BREASTMILK....45
11.8.4. CHILD ANTHROPOMETRY....45

11.8.5. CHILD VACCINATION AND VITAMIN A SUPPLEMENTATION STATUS 45
11.8.6. INFANT NEURODEVELOPMENT 45
11.9. UNSCHEDULED SICK VISIT [ANYTIME DURING STUDY FOLLOW-UP] 46

12. LABORATORY PROCEDURES 46

12.1. FULL BLOOD COUNT 46
12.2. MALARIA TESTING 46
12.3. PLACENTAL HISTOLOGY 46
12.3.1. MALARIA EVALUATION 47
12.4. BLOOD SAMPLES: SEPARATION AND LONG-TERM STORAGE 47
12.5. VAGINAL AND GUT MICROBIOME ANALYSIS 47
12.6. SUMMARY OF LABORATORY MEASUREMENTS 48
12.7. NEUROCOGNITIVE ASSESSMENTS USED IN INFANT FOLLOW UP 51
12.7.1. PROCEDURES FOR ERPS 51

13. ALLOCATION OF PARTICIPANTS TO TRIAL ARMS 52

14. ASSESSMENT OF SAFETY 53

14.1. PRECAUTIONS IN DELIVERING IRON INTRAVENOUS FORMULATIONS 53
14.2. DEFINITIONS OF ADVERSE EVENTS 55
14.3. RECORDING OF ADVERSE EVENTS 55
14.3.1. THE TIME PERIOD FOR COLLECTING ADVERSE EVENTS 55
14.3.2. METHOD OF CAPTURING ADVERSE EVENTS 55
14.3.3. DOCUMENTATION OF ADVERSE EVENTS DATA 55
14.3.4. ASSESSMENT OF ADVERSE EVENT SEVERITY 56
14.3.5. ASSESSMENT OF CAUSALITY OF ADVERSE EVENTS 56
14.3.6. STUDY ENDPOINTS AND SYMPTOMS ANAEMIA 56
14.3.7. HIV-RELATED DISEASE 56
14.3.8. LACK OF EFFICACY AND DISEASE PROGRESSION 56
14.3.9. ABNORMAL LABORATORY VALUES 56
14.3.10. OVERDOSE 57
14.4. REPORTING OF SERIOUS AES 57
14.4.1. REPORTING BY THE INVESTIGATOR TO THE STUDY SAFETY MONITOR AND SPONSOR 57
14.4.2. REPORTING BY THE SPONSOR 57
14.5. DATA MONITORING COMMITTEE 57

15. DATA HANDLING AND RECORD KEEPING 58

15.1. CASE REPORT FORMS (CRFS) 58
15.2. DATA ENTRY AND VALIDATION 58
15.3. DATABASE LOCK 59

16. STATISTICAL CONSIDERATIONS 59

16.1. SAMPLE SIZE CALCULATIONS 59

DATA ANALYSIS 59
16.1.1. ASSESSMENT OF EFFECTIVENESS 59
16.1.2. ANALYSIS OF ADVERSE EVENTS 60
16.2. ANALYSIS POPULATIONS 60
16.3. MISSING DATA 60
16.4. INTERIM ANALYSES AND CRITERIA FOR TERMINATION OF THE TRIAL 60

17. STUDY MANAGEMENT 60

17.1. STUDY MONITORING 60
17.2. DIRECT ACCESS TO SOURCE DATA/DOCUMENTS 61
17.3. QUALITY ASSURANCE 61
17.4. TRAINING OF STAFF 61
17.5. CHANGES TO THE PROTOCOL 61
17.6. FINANCING AND INSURANCE 62
17.7. STUDY DURATION 62
17.8. RECORD-KEEPING AND ARCHIVING 62
17.9. REPORTING AND PUBLICATION OF DATA 62

18. ETHICAL CONSIDERATIONS 63

18.1. ETHICAL REVIEW 63
18.2. ETHICAL CONDUCT OF THE STUDY 63
18.3. INFORMED CONSENT 63
18.4. RISKS TO THE STUDY PARTICIPANTS 64
18.4.1. BLOOD SAMPLING 64
18.4.2. INTRAVENOUS INFUSIONS 64
18.5. BENEFITS FROM PARTICIPATING IN THE TRIAL 64
18.6. SUBJECT DATA PROTECTION 64
18.7. OTHER ETHICAL CONSIDERATIONS 65
18.7.1. REIMBURSEMENT OF COSTS 65

19. DISSEMINATION OF RESULTS 65

20. CAPACITY BUILDING 65

21. BUDGET 67

22. BUDGET JUSTIFICATION 69

23. BIBLIOGRAPHY 70

24. APPENDIX 76

24.1. CASE REPORT FORMS – CRFS 76
24.1.1. PRE-SCREENING 76

24.1.2. SCREENING POST-MALARIA TREATMENT ... 77
24.1.3. FOETAL BIOMETRY ... 78
24.1.4. SCREENING FORM ... 79
24.1.5. INFORMED CONSENT PROCESS ... 80
24.1.6. DEMOGRAPHICS ... 81
24.1.7. SELF-REPORTING QUESTIONNAIRE ... 82
24.1.8. OBSTETRIC HISTORY ... 84
24.1.9. PHYSICAL EXAMINATION ... 88
24.1.10. RANDOMISATION AND PRODUCT ADMINISTRATION ... 91
24.1.11. PARTICIPANT LOCATOR ... 92
24.1.12. CONCOMITANT MEDICATION ... 94
24.1.13. EPDS FORM ... 95
24.1.14. MIBS FORM ... 98
24.1.15. DASS-21 FORM ... 100
24.1.16. PHYSICAL EXAMINATION – DELIVERY FORM ... 106
24.1.17. PREGNANCY OUTCOME ... 107
24.1.18. NEONATAL OUTCOME ... 109
24.1.19. PLACENTAL HISTOPATHOLOGY FORM ... 110
24.1.20. INFANT PHYSICAL EXAMINATION ... 112
24.1.21. INFANT MORBIDITY ... 114
24.1.22. INFANT VACCINATION HISTORY ... 118
24.1.23. HOUSEHOLD FOOD INSECURITY ... 119
24.1.24. CHILD DIETARY DIVERSITY ... 121
24.1.25. CHILD STIMULATORY CARE ... 121
24.1.26. BAYLEY'S ASSESSMENT ... 123
24.1.27. ADVERSE EVENTS ... 124
24.1.28. REPORTING ADVERSE EVENTS ... 127
24.1.29. END OF STUDY FORM ... 127
24.2. CONSENT FORMS ... 129
24.2.1. ICF CHICHEWA ... 129
24.2.2. ICF ENGLISH VERSION ... 142
24.3. MATERIAL TRANSFER AGREEMENT ... 150

1. Abbreviations

AE	Adverse Event
ANC	Antenatal Care
CoM	College of Medicine
CRF	Case Report Form
CRP	C-Reactive Protein
DASS-21	Depression, Anxiety and Stress Status (short form)
dL	Decilitre
DMC	Data monitoring Committee
FCM	Ferric Carboxymaltose
ID	Iron deficiency
IDA	Iron deficiency anaemia
g	Grams
Hb	Haemoglobin
HC	Health Centre
HIV	Human Immunodeficiency Virus
ICTRP	International Clinical Trials Registry Platform
IPTp	Intermittent Preventive Treatment in pregnancy
ITN	Insecticide-treated net
IV	Intravenous
Kg	Kilograms
L	Litres
LMICs	Low and Medium-Income Countries
LRTI	Lower Respiratory Tract Infection
MCATS	Melbourne Clinical and Translational Science Platform
OR	Odd Ratio
PCR	Polymerase Chain Reaction
RCT	Randomised Controlled Trial
RDT	Rapid Diagnostic Test
SC	Sickle Cell
SHC	Sickle Haemoglobin Cell
SP	Sulfadoxine-Pyrimethamine
TfR	Transferrin Receptor
UK	United Kingdom
USA	United States of America
WHO	World Health Organisation
ZCH	Zomba Central Hospital

2. Title of Research Proposal

A **r**andomised controlled trial of the **e**ffect of intra**v**enous iron on **a**naemia in **M**alawian **p**regnant women- **t**hird-**t**rimester study – **REVAMP-TT**

3. Investigators and Institutional Affiliations

Co-Principal Investigators:

- A/Prof Sant-Rayn Pasricha, Walter and Eliza Hall Institute of Medical Research, Australia
- Professor Kamija Phiri, College of Medicine, Training and Research Unit of Excellence (TRUE), University of Malawi, Malawi.

Co-Investigators:

- Dr Martin Mwangi, College of Medicine, Training and Research Unit of Excellence (TRUE), University of Malawi, Malawi.
- Professor Stephen Rogerson, University of Melbourne, Australia
- Professor Julie Simpson, University of Melbourne, Australia
- Professor William Stones, College of Medicine, University of Malawi, Malawi
- Professor Beverley-Ann Biggs, University of Melbourne, Australia
- Professor Jane Fisher, Monash University
- Ms Sabine Braat, University of Melbourne, Australia
- Dr Leila Larson, University of South Carolina, United States of America
- A/Prof Stefan Bode, University of Melbourne, Australia
- A/Prof Katherine Johnson, University of Melbourne, Australia
- Dr Ricardo Ataide, University of Melbourne, Australia
- Dr Rebecca Harding, Wlater and Eliza Hall Institute of Medical Research, Australia

4. Protocol Summaries

4.1. Trial registration data

Data Category	*Information*
Primary registry and trial identifying number	XXXX
Date of registration in primary registry	XXXX
Secondary identifying numbers	NHSRC: XXXX
Source(s) of monetary or material support	Bill and Melinda Gates Foundation
Primary sponsor	Training and Research Unit of Excellence (TRUE), Malawi.
Secondary sponsor(s)	NA
Contact for public queries	Associate Prof Sant-Rayn Pasricha, E-mail: pasricha.s@wehi.edu.au
Contact for scientific queries	Associate Prof Sant-Rayn Pasricha, Walter and Eliza Hall Institute of Medical Research, 1G Royal Parade, Parkville Victoria 3052, Australia; Mobile: +61 407 141 570; E-mail: pasricha.s@wehi.edu.au
Public title	A randomised controlled trial of the effectiveness of intravenous iron on anaemia in Malawian pregnant women in their third trimester
Scientific title	Effectiveness of intravenous iron administered during the third trimester in Malawian pregnant women in the management of anaemia: A 4-year, multi-centre, parallel-group, two-arm open-label randomized controlled superiority trial
Countries of recruitment	Malawi
Health condition(s) or problem(s) studied	Anaemia, iron deficiency
Intervention(s)	intravenous ferric carboxymaltose (1000 mg for body weight ≥50 kg, or 20 mg/kg for body weight <50 kg) once during the third trimester
	Standard of care: Oral iron 200 mg ferrous sulphate (approx. 65 mg elemental iron) twice daily for 90 days or the duration of pregnancy, whichever is shorter
Study type	Interventional
	Allocation: randomised; intervention model: parallel assignment; arms:2~~3~~; allocation ratio: ~~1:~~1:1; Masking: open-label
	Primary purpose: treatment
	Phase-III

Date of first enrolment	XXXX
Target sample size	590
Recruitment status	Not yet recruiting
Key inclusion criteria	• Confirmed singleton pregnancy in the third trimester (27-35 weeks of gestation, dated by Last Menstrual Period)<br>• Moderate to severe anaemia not requiring an immediate blood transfusion (Hb <10 g/dl)<br>• Negative malaria parasitaemia by mRDT<br>• Currently afebrile with no evidence of septicaemia<br>• Resident in the study catchment area of Zomba district<br>• Able to deliver at health facilities within Zomba district<br>• Written informed consent (including assent if <18 years old)
Exclusion criteria (at enrolment)	• Previous enrolment in REVAMP trial (COMREC P.02/18/2357)<br>• Actively participating in another intervention trial<br>• Known hypersensitivity to the study drugs<br>• Clinical symptoms of malaria or other infection (no fever, no focal symptoms of internal infection i.e. LRTI/ diarrhoea)<br>• Any condition requiring hospitalisation in the next seven days or serious concomitant illness<br>• Known history of sickle cell or sickle-haemoglobin C anaemia<br>• Clinically low haemoglobin level requiring a blood transfusion (usually Hb <5 g/dl)<br>• Pre-eclampsia
Primary outcome(s)	Proportion of women with pre-delivery anaemia (defined as venous Hb <11.0 g/dl at 36 weeks' gestation.
Key<br>Secondary efficacy outcomes	• Mean change from baseline in maternal Hb at 36 weeks' gestation.<br>• Proportion of women with maternal iron deficiency (ferritin<15 mg/L, sTfR/Ferritin index) at 36 weeks' gestation.<br>• Mean levels of maternal iron biomarkers at 36 weeks' gestation.<br>• Proportion of women with maternal inflammation (using C-reactive protein) at 36 weeks' gestation.<br>• Proportion of women with maternal postpartum haemorrhage<br>• Mean change from baseline in maternal Hb at 1, 3, 6, 9 and 12 months postpartum.

	• Proportion of women with maternal anaemia at 1, 3, 6, 9 and 12 months postpartum<br>• Mean levels of maternal iron biomarkers at 1, 3, 6, 9 and 12 months postpartum<br>• Proportion of women with iron deficiency (defined by ferritin<15mg/L) at 1, 3, 6, 9 and 12 months postpartum<br>• Proportion of women with postpartum depression (defined by EDPS > 13) at 3 months postpartum.<br>• Proportion of women with maternal inflammation (defined by C-reactive protein) at 3 months postpartum<br>• Mean gestation duration (in weeks)<br>• Proportion of sub-optimal pregnancy outcomes (defined as a composite outcome: low birth weight (<2500 g); prematurity (birth <37 weeks); small for gestational age (centile score) as defined by International reference standards for gestational age-specific birth weight; stillbirth).<br>• Proportion of neonates born prematurely (defined as birth before 37 week's gestation)<br>• Proportion of infants with low birth weight (defined as a birth weight <2500 g).<br>• Proportion of stillbirth.<br>• Proportion of neonatal mortality.<br>• Mean cord blood Hb and ferritin.<br>• Proportion of neonates with anaemia (with correction for gestational age).<br>• Child development scores measured by Evoked Response Potentials (ERP) at 6 and 12 months of age.<br>• Child development scores measured by the Malawi Developmental Assessment Tool (MDAT) and Bayley Scales of Infant Development at 6 and 12 months of age.<br>• Mean child physical growth as defined by z-scores at 1, 6 and 12 months of age.<br>• Mean infant haemoglobin (capillary) at 6- and 12-months postpartum.<br>• Proportion of infants with anaemia at 6- and 12-months postpartum.<br>• Mean levels of infant iron biomarkers at 6- and 12-months postpartum.<br>• Proportion of infants with iron deficiency at 6- and 12-months postpartum
Safety outcomes	• Proportion of women with at least one treatment related adverse effects (occurring immediately post-infusion, and within 7 days of commencement of treatment in the intravenous group~~s~~).<br>• Incidence of treatment related adverse effects (occurring immediately post-infusion, and within 7 days of commencement of treatment in the intravenous group~~s~~).<br>• Number of unplanned visits to the clinic (cause specific for diarrhea and clinical malaria).

	• Incidence of all-cause maternal sick clinic visits during the antenatal, postpartum and overall participant follow-up period.<br>• Incidence of maternal cause-specific sick clinic visits (in particular malaria, diarrhoea and other infectious conditions) during the antenatal, postpartum and overall participant follow-up period<br>• Proportion of women with placental malaria (past or active infection on histology, parasites on placental blood film).<br>• Proportion of women with malaria parasitaemia (asymptomatic) at 36 weeks' gestation, detected by a) microscopy, b) rapid diagnostic tests, and c) PCR.<br>• Proportion of women with bacteraemia at 36 weeks' gestation.<br>• Proportion of women with hypophosphatemia (clinical and biochemical) at 36 weeks' gestation and 3 months postpartum.<br>• Proportion of infants with hypophosphataemia (biochemical) at 6 weeks and at 6 months of age.<br>• Number of unplanned infant visits to the clinic (cause specific for diarrhea and clinical malaria).<br>• Incidence of all-cause sick clinic visits in infant by 12 months of age.<br>• Incidence of cause-specific sick clinic visits (in particular malaria, diarrhoea and other infectious conditions) in infant by 12 months of age.<br>• Proportion of infants with malaria parasitaemia (asymptomatic) 1, 3, 6, 9 and 12 months postpartum detected by a) microscopy, b) rapid diagnostic tests, and c) PCR

4.2. Executive summary

Type of study: Open-label two-arm parallel-group individual-randomised controlled trial

Problem: Anaemia in pregnancy remains a critical global health problem, affecting 46% of pregnant women in Africa and 49% in Asia. Antenatal anaemia causes significant risks for both mother and child. Anaemia can amplify the risks and consequences of serious complications (including maternal mortality) from maternal haemorrhage and contributes to low birth weight and premature delivery, which both pose critical immediate and long-term risks for the survival, development and wellbeing of the newborn infant.

In pregnancy, anaemia is commonly due to iron deficiency. A recent placebo-controlled double-blind, randomised single-centre field trial in Kenyan pregnant women exemplified the benefits and safety of iron supplementation during pregnancy in the sub-Saharan African context by showing a substantial benefit from iron supplementation on birth weight and the duration of gestation[1]. However, the success of the Kenyan trial resulted from the high adherence demanded of the participants (100%), achieved by daily visits by fieldworkers. The need for such a high adherence emphasises the importance of delivering of a full course of iron supplementation on birth outcomes. Across Africa, only very few women receive the full recommended course of antenatal iron and may present for their initial visit far into the second trimester. This late presentation limits opportunities to treat antenatal anaemia, exposing women and their babies to its consequences.

New intravenous iron products have become available in developed countries and provide a chance to give high doses of iron in a single rapid infusion. Studies comparing older formulations of intravenous iron to oral iron treatment in pregnancy[2–4] found intravenous iron superior for improvements in haemoglobin (e.g. MD 7.4 g/L [3.9, 11.0]) and birth weight (about 58 g) [3,4] and for reducing maternal blood transfusion needs (OR 0.19 [0.05, 0.78]) [3]. Collectively, these data emphasise the critical importance of screening for, preventing and treating ID during pregnancy. There is a real need for the establishment of a parenteral iron formulation in women with moderate or severe IDA in pregnancy, and perhaps in all women with IDA in the third trimester when the foetal iron transfer is highest, and delivery (with the risk of blood loss) is imminent.

Ferric Carboxymaltose is the most commonly used intravenous iron product and provides a single rapid iron infusion suitable for administration in primary care and other non-hospital settings.

Objectives: To determine the effectiveness and safety of intravenous iron administration during the third trimester of pregnancy – given as Ferric Carboxymaltose (FCM) compared with standard of care oral iron in improving maternal (especially anaemia) and infant (growth, birth weight and development up to 12 months postpartum) outcomes.

Methods: This is an open-label two arm parallel-group randomised controlled trial in anaemic pregnant women. The primary maternal outcome is recovery from anaemia at 36-weeks gestation, and the important secondary neonatal outcome is birth weight. Study visits occur over pregnancy, at birth, and follow-up to 12-month postpartum. Other secondary outcomes include (i) maternal iron-deficiency biomarkers; (ii) maternal postpartum depression and wellbeing levels; (iii) infant neurocognitive development, growth, anaemia, and iron status; (iv) adverse events of administering intravenous iron in this setting – including hypophosphatemia; and (v) maternal and infant infection

(e.g. malaria and diarrhoea) events. The study will have two arms: (a) intravenous iron FCM; (b) oral iron. Apart from the source of iron-supplementation, all ANC procedures will be equal between the arms of the trial which may include IPTp, if scheduled. The intravenous iron Intervention group will receive ~~either i)~~ intravenous FCM 1000mg for body weight ≥50 kg, or 20 mg/kg for body weight <50 kg, once during the third trimester. The control group will receive standard of care oral iron 200 mg ferrous sulphate (approx. 65 mg elemental iron) twice daily for the duration of pregnancy. Both arms will receive sulfadoxine-pyrimethamine (SP) as IPTp according to national guidelines if the three doses of recommended IPTp have not been already achieved.

The planned number of randomised participants is 590 pregnant women (295 participants per study arm). The study has 90% power at a two-sided alpha level of 2.5% and incorporating a 10% loss to follow up to detect a difference on the primary maternal outcome between intravenous iron arm and oral iron (49% vs 63%). The sample is also sufficient to have 80% power to detect a 110g difference in birthweight (assuming standard deviation in birth weight of 450g) between the intravenous iron arm and the oral iron arm (two-sided alpha level of 5%) after accounting for a miscarriage and stillbirth rate of 1%. The trial will be based at the TRUE centre at Zomba Central Hospital in Southern Malawi but will operate in health centres across Zomba district. An international team of experts in clinical trials, obstetrics, anaemia, malaria, and implementation sciences based in Malawi and Australia will conduct the trial.

Because anaemia in pregnancy may influence the long-term health of the mother and her baby, we will continue to follow the cohort of women and babies postpartum to assess the longer-term benefits and safety of this treatment. Women and babies will be followed up until 12 months postpartum (infant age 12 months), and will undergo study visits at 4-weeks, 3-, 6-, 9- and 12- months. Mothers and babies will undergo testing for haematologic, iron, nutritional and infection status. Participants will also be consented for use of samples for translational work including immunologic and genetic analyses. Women will undergo testing for wellbeing and depression, and babies will undergo cognitive testing.

Additionally, an implementation study and health system analysis (REVAMP-IS), conducted in parallel, will assess the feasibility of implementing this intervention in routine antenatal care within the mainstream health system in low-income countries.

Expected Findings and Implications: Oral iron supplementation remains cheap while the drug cost of intravenous iron delivery is much higher. Clinical superiority of intravenous iron over oral iron for the recovery of anaemia in the third trimester needs to be demonstrated. Additionally, there is a real need for an in-depth analysis of potential implementation barriers in the setting of antenatal care in LMIC. We hypothesise a clear benefit from intravenous iron on maternal haematologic outcomes and wellbeing, on critical neonatal outcomes such as birth weight and gestation duration, and infant development and wellbeing. We hypothesise that intravenous iron to be safe to administer in ~~this~~ primary health care centres. We hypothesise that FCM will cause hypophosphatemia but that this will not have clinical implications, be asymptomatic and will not affect the child. In conjunction with REVAMP-IS, our study may lead to the implementation of intravenous iron as a treatment recovery from maternal anaemia in the third trimester, which could have long term benefits for maternal and child health, ultimately resulting in benefits for maternal and child survival and child development.

Dissemination: We will present the results from this study at local and international fora. The results will be submitted for publication in peer-reviewed scientific journals, policy briefs to WHO and donors such as the Bill and Melinda Gates Foundation and reported to NHSRC and other relevant ethics committees.

4.3. SCHEDULE OF ACTIVITIES

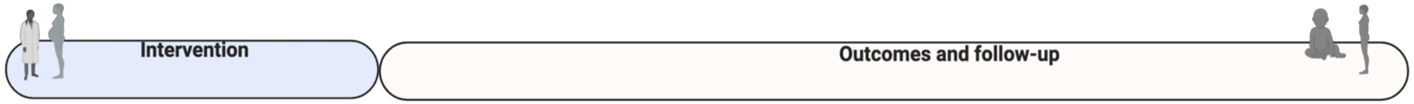

	Visit 0[1]	Visit 1	Visit 4	Visit 7	Visit 8	Visit 9	Visit 10	Visit 11	Visit 12	Sick Visit Unscheduled
**Protocol Activity**	***May occur at visit 1***	***Week 27-35***	***36 weeks ±2d (pre-delivery)***	***Delivery ±2d***	***28 days postpartum ± 2d***	***3 months postpartum ±14 Days***	***6 months postpartum ±14 Days***	***9 months postpartum ±14 Days***	***12 months postpartum ±14 Days***	
**Location of visit**	**HF[j]**	**HFj**	**HF[j]**	**HF[j]**	**HF[j]**	**HF[j]**	**HF[j]**	**HF[j]**	**HF[j]**	**HF[j]**
**Pre-screening form completion**	**X**									
**Screening**	**X**									
**Informed consent process**		**X**								
**Medical & obstetric history**		**X**								
**Household economic data form**		**X**								
**Household food insecurity**			**X**			**X**				
**Complete physical examination[a]**		**X**								
**Limited physical examination[b]**			**X**	**X**	**X**	**X**				
**Maternal psychological health: DASS-21 and MIBS data forms completion**			**X**			**X**				
**Edinburgh Postpartum depression scale (EPDS)**			**X**			**X**				
**Self-reporting questionnaire**		**X**								

[1] Visit numbers are not sequential, but instead are harmonised to match the visit numbers adopted in the ongoing REVAMP-EXT trial (REVAMP-EXT trial – P.02/18/2357). This safeguards the operational success of this trial.

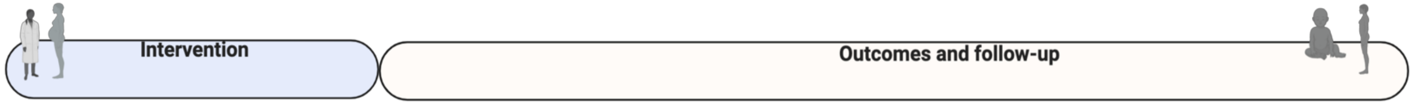

	Visit 0[1]	Visit 1	Visit 4	Visit 7	Visit 8	Visit 9	Visit 10	Visit 11	Visit 12	Sick Visit Unscheduled
**Protocol Activity**	*May occur at visit 1*	*Week 27-35*	*36 weeks ±2d (pre-delivery)*	*Delivery ±2d*	*28 days postpartum ± 2d*	*3 months postpartum ±14 Days*	*6 months postpartum ±14 Days*	*9 months postpartum ±14 Days*	*12 months postpartum ±14 Days*	
**Location of visit**	HF[j]	HFj	HF[j]	HF[j]	HF[j]	HF[j]	HF[j]	HF[j]	HF[j]	HF[j]
**Participant arm allocation**		X								
**Administer treatment**										
**intravenous iron**		X								
**Oral iron**		X								
**Laboratory procedures (Maternal)**										
**Full Blood Count (including Hb)**		X	X	X	X	X	X	X	X	
**Haemoglobin (capillary)**	X									
**Malaria RDT**		X								X
**Malaria microscopy**		X	X	X	X	X	X	X	X	X
**Malaria filter paper for PCR**		X	X	X	X	X	X	X	X	X
**Serum for iron markers tests[c]**		X	X	X	X	X	X	X	X	
**Serum for inflammatory markers tests[d]**		X	X	X	X	X	X	X	X	
**Phosphate**		X	X	X	X		X		X	
**Vaginal swab sample collection[e]**			X		X				X	
**Placenta histology**				X						

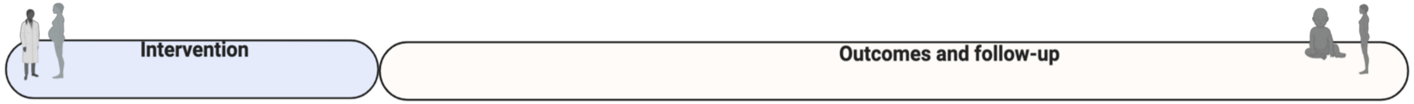

Protocol Activity	Visit 0[1]	Visit 1	Visit 4	Visit 7	Visit 8	Visit 9	Visit 10	Visit 11	Visit 12	Sick Visit Unscheduled
	May occur at visit 1	*Week 27-35*	*36 weeks ±2d (pre-delivery)*	*Delivery ±2d*	*28 days postpartum ± 2d*	*3 months postpartum ±14 Days*	*6 months postpartum ±14 Days*	*9 months postpartum ±14 Days*	*12 months postpartum ±14 Days*	
**Location of visit**	HF[j]	HFj	HF[j]	HF[j]	HF[j]	HF[j]	HF[j]	HF[j]	HF[j]	HF[j]
**Breast milk sample**					X		X[g]		X[g]	
**Laboratory procedures (Infant)**										
**Full Blood Count**				X (cord)	X		X		X	
**Malaria microscopy**				X (cord)	X	X	X	X	X	
**Serum for iron markers tests[c]**				X (cord)	X	X	X	X	X	
**Malaria filter paper for PCR**				X (cord)	X	X	X	X	X	
**Stool sample**					X		X		X	
**Pregnancy outcome**				X	X					
**Birth weight**				X						
**Complete physical examination of baby[h]**				X						
**Limited physical examination of baby[i]**					X	X	X	X	X	
**Maternal anthropometry: weight, height**				X	X	X	X	X	X	
**Child anthropometry: weight, length, head circumference**				X	X	X	X	X	X	
**Child vaccination and Vit A supplementation status**					X	X	X	X	X	

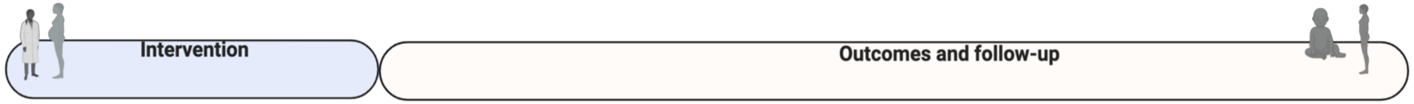

Protocol Activity	Visit 0[1]	Visit 1	Visit 4	Visit 7	Visit 8	Visit 9	Visit 10	Visit 11	Visit 12	Sick Visit Unscheduled
	May occur at visit 1	*Week 27-35*	*36 weeks ±2d (pre-delivery)*	*Delivery ±2d*	*28 days postpartum ± 2d*	*3 months postpartum ±14 Days*	*6 months postpartum ±14 Days*	*9 months postpartum ±14 Days*	*12 months postpartum ±14 Days*	
**Location of visit**	**HF[j]**	**HFj**	**HF[j]**	**HF[j]**	**HF[j]**	**HF[j]**	**HF[j]**	**HF[j]**	**HF[j]**	**HF[j]**
**Child dietary diversity**							**X**		**X**	
**Simulating caregiving**							**X**			
**Infant neurodevelopment using electroencephalography (EEG), Infant and Toddler Development tools (e.g. Baileys and MDAT)**							**X**		**X**	
**Infant neurodevelopment using auditory brainstem responses (ABRs)**					**X**		**X**			

[a] Complete examination: general appearance, throat, neck, thyroid, musculoskeletal, skin, lymph nodes, extremities, pulses, pulmonary, cardiac, abdominal, and neurological examination

[b] Limited examination: general appearance, brief pulmonary, cardiac, abdominal and neurological examination

[c] e.g. Serum ferritin, sTfR

[d] e.g. CRP and alpha-1 glycoprotein

[e] Reproductive tract microbiome analysis: e.g. *Chlamydia trachomatis, Neisseria gonorrhoeae, Trichomonas vaginalis, Lactobacillus spp.* (done only for a sub-set of 50 women)

[f] Stool microbiome analysis

[g] Done only if mothers are still breast feeding

[h] Complete examination: weight, length, head circumference, APGAR score, Ballard score, new-born adiposity, congenital anomaly, and complications at birth

[i] Limited examination: general appearance, brief pulmonary, cardiac, abdominal and neurological examination

[j] Visits will take place at a Health Facility

5. Introduction

5.1. Background

5.1.1. Anaemia during pregnancy remains a critical global health problem

Almost 40% of pregnant women worldwide are anaemic, including 46% of pregnant women in Africa and 49% in Asia, compared with 17% in North America[9]. Anaemia in pregnancy is associated with critical risks for both mother and child. The maternal risks include life-threatening complications of postpartum haemorrhage. On the other hand, the risks for the child include prematurity and low birth weight[10] – which are associated with increased risk of mortality, and reduced iron stores in infancy with increased risk of subsequent anaemia and impaired development[11]. Anaemia in pregnancy is associated with an adjusted 3.6 odds ratio for increased maternal mortality[12], and in the USA is associated with impaired long-term child cognitive development. Control of anaemia in women is, therefore, a key 2025 global nutrition target[13].

5.1.2. Low birth weight and pre-term birth have critical implications for mother and baby

Worldwide, 15%-20% of births worldwide (>20 million annually) (including 13% in sub-Saharan Africa) are low birth weight, while each year, over 1 million children die from complications of pre-term birth. Although the underlying determinants for low birth weight and prematurity are diverse, prevention and treatment of antenatal anaemia with iron is an essential component of the 2025 WHO nutrition target of a 30% reduction in low birth weight[14,15].

5.1.3. Iron can benefit antenatal anaemia

A recent placebo-controlled double-blind, randomised single centre field trial in Kenyan pregnant women exemplified the benefits and safety of iron supplementation during pregnancy in the sub-Saharan African context [1]. In this trial, oral iron supplementation increased birth weight by 150 g and reduced the risk of low birth weight by 58%, lengthened gestation duration by 3.4 days and reduced the risk of premature birth by 7% and reduced the risk of maternal anaemia from 50.4% to 22%. A welcomed observation, in this highly malaria-endemic setting, was that there was no evidence of an increase in clinical malaria, placental malaria or parasitaemia among women randomised to receive iron. This trial is one of the most recent examples of an extensive literature of trials evaluating oral iron in pregnancy. Systematic reviews of these studies confirm likely benefits from iron on maternal outcomes, including anaemia (70% reduction), and trends towards favourable infant outcomes including increased birth weight and extended gestation duration [16]. The success of the Kenyan trial rested on the high adherence demanded of the participants (100%), achieved by daily visits by fieldworkers. This dependency emphasises the importance of delivery of a full course of iron supplementation on birth outcomes.

5.1.4. UPTAKE AND ADHERENCE TO ORAL IRON THERAPY DURING PREGNANCY IS INADEQUATE IN THE FIELD

Global recommendations for the management of anaemia in pregnancy in LMICs are that women be treated with high dose daily oral iron (120 mg of elemental iron) supplementation for three months [17,18]. However, such high amounts of iron are often poorly tolerated due to significant gastrointestinal adverse effects[19] limiting adherence to this vital intervention. Moreover, delivery of iron during pregnancy requires ongoing contacts between the mother and the primary health system. For example, in Malawi, fewer than 25% of pregnant women receive a full course of iron (*Clophat Baleti, Personal Communication*). Across Africa, only very few women get the recommended course of antenatal iron and may present for their initial visit far into the second trimester. This late presentation limits opportunities to treat antenatal anaemia, exposing women and their babies to its consequences. Furthermore, even when delivered, oral iron frequently fails to correct anaemia in routine practice. For example, in our recent study among pregnant women in The Gambia, we provided iron to all women with Hb<10 g/dL at week 20 of gestation; by week 30, 61% still had Hb<10 g/dL[20].

5.1.5. FERRIC CARBOXYMALTOSE: A NEW INTRAVENOUS IRON PREPARATIONS

Over the past decade, there have been dramatic improvements in the safety and convenience of parenteral (intravenous) iron therapies[3–5,21]. Older forms of intravenous iron were associated with severe allergic reactions (iron dextran), required prolonged administration periods (iron polymaltose), or required frequent infusions of small doses of 200 mg (iron sucrose). However, new intravenous agents have entirely changed the landscape of iron therapy.

Ferric carboxymaltose

The most established intravenous therapy is Ferric carboxymaltose (FCM), which overcomes many limitations of previous parenteral iron treatments. Ferric carboxymaltose comprises a colloidal complex of polynuclear iron (III) oxyhydroxide core with carboxymaltose ligands[22]. Following administration, the complex is degraded into simple endogenous molecules (glucose, maltose, maltotriose, maltotetraose and iron), and iron is rapidly taken up by iron transport (transferrin) and storage (ferritin) proteins. Due to this property, FCM can be administered in a short time and at large doses[23]. As currently licensed, a dose of up to 1000 mg of iron (or 15-20 mg/kg body weight), diluted in 250 mL saline, may be administered over a 15-minute infusion. In most cases, this enables a total dose of iron replacement to be achieved in a single visit. FCM has now revolutionised the treatment of iron deficiency anaemia in high-income country settings and is widely used in ambulant/ outpatient settings, emergency departments, in non-specialist clinical wards, preoperative clinics and even in remote settings. FCM has been available in Europe since its approval in 2007 and the USA since 2009 and. Over 50 countries are currently marketing FCM [23].

Safety and efficacy evidence of Ferric carboxymaltose in various patient populations

Rognoni et al. 2015 undertook a systematic review and network meta-analysis of FCM vs both oral iron and other parenteral formulations[21]. The authors identified 21 RCTs comparing iron treatments in anaemic (or non-anaemic) patients requiring therapies for ID published between 2003-2014, although not all of these included FCM.

Regarding safety, the review authors found that overall, FCM was well tolerated and associated with minimal risk of AEs[21]. In trials in which AEs occurred in a more substantial proportion of FCM-treated patients than those receiving oral iron or placebo, the difference seldom reached the statistical significance level. Several studies found that patients who were given FCM experienced fewer drug-related gastrointestinal disorders (e.g. constipation, diarrhoea) than those treated with oral iron. However, patients treated with ferric carboxymaltose iron were more likely to develop rash, dermatitis and pruritus that generally resolved within a few minutes of the infusion. Other frequent AEs associated with ferric carboxymaltose administration were fatigue, headache and dizziness. No true cases of anaphylaxis and no deaths occurred in patients receiving FCM.

Regarding efficacy, the authors undertook a network meta-analysis to compare the efficacy of FCM as compared with oral iron and other parenteral formulations. The authors found that in terms of improving haemoglobin concentrations, FCM was superior to placebo (delta 2.1; 95 % CI 1.2–3.0), oral iron (delta 0.8; 95 % CI 0.6–0.9), intravenous ferric gluconate (delta 0.6; 95 % CI 0.2–0.9), and iron sucrose (but not reaching statistical significance). The authors found that for improvements in ferritin, FCM was superior to oral iron (delta 172.76; 95 % CI 66.7–234.4) and similar to other parenteral formulations (iron sucrose and gluconate)[21].

A previous systematic review and meta-analysis published in September 2011 evaluated published and unpublished studies of efficacy and safety of ferric carboxymaltose[24]. The analysis assessed clinical trial reports and published studies comparing FCM with either other active comparators or with placebo. The authors identified fourteen studies, in which 2,348 patients had been assigned to FCM, compared with, in the control arms, 762 to placebo, 832 to oral iron and 384 to intravenous iron sucrose. Compared with oral iron, ferric carboxymaltose produced superior improvements in haemoglobin, ferritin and transferrin saturation, and was associated with a higher chance of resolution of anaemia or clinically significant increase in haemoglobin. Maximum responses were usually achieved by 4-6 weeks. Adverse events were also evaluated.

The table below, from this manuscript, shows the risk of withdrawals and adverse events in patients receiving ferric carboxymaltose compared with those randomised to receive either control or another iron intervention. As compared with either oral iron or placebo, participants receiving FCM were slightly less likely to withdraw from the study, while there was no difference in the number of participants experiencing at least one adverse event, death, or serious adverse events between subjects receiving FCM and those receiving other interventions. However, there was a slight increase in the risk of hypotension in participants receiving FCM when explicitly compared with oral iron (but not when compared with receipt of other intravenous interventions).

Table 2: Risk of Withdrawals and Adverse Events in patients receiving intravenous Ferric Carboxymaltose

Outcome	Comparator	Number of Trials	Number of Patients	Percent with Ferric carboxymaltose	Percent with Control	RB or RR 95% CI	NNTp 95% CI
**Withdrawals**							
All cause	All	10	3835	6.4	7.5	0.8 (0.6 to 0.9)	93 (37 to 180)
	Oral iron	6	1898	8.1	9.3	0.8 (0.6 to 1.03)	not calculated
Adverse event	All	8	3319	1.0	1.6	0.6 (0.3 to 1.02)	not calculated
	Oral iron	6	1898	1.5	1.9	0.7 (0.3 to 1.4)	not calculated
Lack of efficacy	All	7	2967	0.6	0.8	0.8 (0.4 to 1.7)	not calculated
	Oral iron	5	1546	1.1	1.2	0.8 (0.4 to 1.9)	not calculated
**Adverse events**							**NNH 95% CI**
At least 1 AE	All	8	2951	41	38	1.1 (1.0 to 1.2)	not calculated
	Oral iron	5	1539	48	53	1.0 (0.9 to 1.1)	not calculated
Death	All	10	3762	0.53	0.3	1.3 (0.5 to 3.4)	not calculated
	Oral iron	6	1891	0.38	0.0	1.7 (0.4 to 6.6)	not calculated
Serious AE	All	8	3303	2.5	2.3	1.0 (0.6 to 1.5)	not calculated
	Oral iron	6	1891	3.1	2.3	1.3 (0.7 to 2.2)	not calculated
Hypotension	All	6	2694	1.5	1.0	1.5 (0.8 to 2.7)	not calculated
	Oral iron	4	1339	1.3	0.0	4.7 (1.1 to 21)	79 (44 to 390)

Note: NNT - Number Needed to Treat; NNH - Number Needed to Harm; RB – Relative Benefit; RR – Relative Risk

The table below, adapted from this meta-analysis, compares the incidence of various organ-specific side effects associated with the administration of FCM when compared with i) oral iron as the control, and ii) intravenous saline as the control.

Table 3: Organ-specific side effects of ferric carboxymaltose compared with oral iron or intravenous saline

Outcome	Number of Trials	Number of Patients	Percent with Ferric carboxymaltose	Percent with Control	RB or RR 95% CI	NNTp/H 95% CI
**Comparison with oral iron**						
**Body system and preferred term**						
GI disorder	5	1539	13	32	0.44 (0.36 to 0.54)	5.4 (4.4 to 7.1)
General, administrative site	5	1539	11	4	2.8 (1.9 to 4.2)	**15 (11 to 24)**
Infection, infestation	5	1539	14	12	1.2 (0.9 to 1.6)	not calculated
Metabolism, nutrition, investigation	4	1195	11	5	2.2 (1.4 to 3.4)	**17 (11 to 33)**
Nervous system	5	1539	10	9	1.3 (0.9 to 1.7)	not calculated
**Specific adverse events**						
Constipation	4	1339	3	13	0.3 (0.2 to 0.4)	9.8 (7.6 to 14)
Diarrhoea	3	906	2	5	0.5 (0.2 to 0.9)	33 (18 to 230)
Nausea/vomiting	3	906	3	10	0.4 (0.2 to 0.6)	14 (9.5 to 27)
Headache	5	1539	7	7	1.2 (0.8 to 1.7)	not calculated
**Comparison with IV saline**						
**Body system and preferred term**						
GI disorder	2	1577	8	5	1.6 (1.1 to 2.4)	**34 (19 to 210)**
General, administrative site	2	1577	6	2	2.5 (1.5 to 4.3)	**25 (17 to 49)**
Infection, infestation	2	1577	9	6	1.1 (0.8 to 1.6)	not calculated
Nervous system	2	1577	8	6	1.2 (0.9 to 1.8)	not calculated
Respiratory system	2	1577	2	2	0.8 (0.4 to 1.5)	not calculated

Note NNTp in normal text, NNH when bold

Note: NNTp - number needed to treat to prevent harm; NNH - Number Needed to Harm; RB – Relative Benefit; RR – Relative Risk

As shown in the table above, compared with oral iron, FCM was less likely to cause constipation, diarrhoea, nausea/ vomiting, and gastrointestinal disorders in general, but was more likely to cause adverse events at the administration site. When FCM was compared to intravenous saline, FCM was associated with an increased risk of 'GI disorder' and local site reactions, but not with adverse effects in other organ systems.

Numerous randomised controlled trials have compared FCM with either oral iron or other forms of parenteral iron for treatment of IDA in a variety of clinical situations, including pregnancy[25], postpartum[26] and in a variety of clinical conditions including inflammatory bowel disease[27–33], chronic renal failure[34,35], and heart failure[36]. It has also been studied in the preoperative context for optimising preoperative haemoglobin concentrations as a component of patient blood management[37–41]. Here, we will summarise recent reviews of FCM that address vital safety and efficacy endpoints.

Safety of Ferric carboxymaltose

Several studies, including one randomised controlled trial, have evaluated the efficacy and safety of FCM in pregnancy. These studies are summarised here:

Breymann et al.[25]*:* FER-ASAP was the pivotal, Phase IIIb, open-label randomised controlled trial comparing FCM with oral iron (ferrous sulphate) for treatment of iron deficiency anaemia in pregnancy. This multicentre study was set across eight high and middle-income countries and randomised 252 women in their second or third trimester of pregnancy to FCM (1000-1500 mg iron) or FS (200 mg iron/day for 12 weeks). The trial was designed to identify a more rapid improvement in haemoglobin concentration by three weeks post-infusion compared with baseline. The study showed an advantage of Hb with FCM compared with FS at 6 and 3 weeks (although the difference was not significant at three weeks). According to the SF-36 health survey, FCM treatment led to significant, clinically relevant improvements over FS in vitality and social functioning before delivery.

Safety: The incidence of Treatment-Emergent Adverse Events (TEAEs) was similar between the treatment arms: in the FCM group, 60 women (49%) experienced 165 TEAEs; in the FS group, 50 women (40%) experienced 105 TEAEs. The majority of events were mild in intensity (see table 5 below).

Table 4: Incidence of Treatment-Emergent Adverse Events (TEAEs)

Treatment-related TEAE severity, number of patients (%)	Ferric carboxymaltose (n=123)	Ferrous sulfate (n=124)
Total	60 (49)	50 (40)
Mild	43 (72)	28 (56)
Moderate	17 (28)	20 (40)
Severe	0 (0)	2 (4)

TEAE=treatment-emergent adverse event.

Overall, the most common TEAEs were nausea (6%), headache (5%) and dyspepsia (4%), and the most common TEAEs according to system organ class were "pregnancy, puerperium and perinatal conditions" in the FCM group [32 events in 26 women (21%)] and "gastrointestinal disorders" in the FS group [42 events in 25 women (20%)]. The most common treatment-related TEAEs were headache with FCM [experienced by 4 women (3%)] and nausea with FS [in 6 women (5%)], and markedly higher rates of gastrointestinal disorders were reported with FS treatment (in 16 women) compared with FCM treatment (3 women).

Table 5: TEAEs in FCM vs FS treatment arms

Treatment-related TEAE,[a] number of patients (%)	Ferric carboxymaltose (n=123)	Ferrous sulfate (n=124)
Total	14 (11)	19 (15)
Nervous system disorders	7 (6)	1 (1)
Headache	4 (3)	1 (1)
Dizziness	3 (2)	0 (0)
Dysgeusia	2 (2)	0 (0)
General disorders and administration-site conditions	4 (3)	0 (0)
Vascular disorders	2 (2)	0 (0)
Gastrointestinal disorders	3 (2)	16 (13)
Nausea	2 (2)	6 (5)
Vomiting	0 (0)	2 (2)
Constipation	0 (0)	3 (2)
Diarrhea	0 (0)	4 (3)
Abdominal pain upper	0 (0)	5 (4)
Dyspepsia	0 (0)	3 (2)

[a]According to physician's assessment; a single patient could appear in multiple classes. Percentages calculated according to treatment group.
MedDRA=Medical Dictionary for Regulatory Activities (version 16.1). TEAE=treatment-emergent adverse event.

Serious TEAEs occurred in 23 women treated with FCM (26 events) and in 10 women treated with FS (11 events); all were single events, except for "failed trial of labour", "foetal distress syndrome", "premature delivery", "premature rupture of membranes" and "threatened labour", each of which occurred in two women treated with FCM, and "premature labour", which occurred in three women treated with FS (pre-eclampsia was also reported to have occurred twice in one woman treated with FS). One serious TEAE, "bronchospasm", which was treatment-related, led to discontinuation of FCM; this event was of moderate-intensity and resolved on the same day after the withdrawal of study treatment. Seven women discontinued treatment with FS because of gastrointestinal TEAEs (n=5), syncope (n=1) and rash (n=1). No hypophosphatemia TEAEs were reported during this study. Eleven women (FCM, n=10; ferrous sulphate, n=1) recorded phosphate levels below the lower normal range threshold [0.6 mmol/L (2 mg/dL)]; these decreases were observed at week three and recovered in all the women to within the normal range by the end of the study.

All other trials have been single-arm prospective trials or retrospective comparative studies.

Froessler et al[42]. undertook a prospective observational study in 65 anaemic pregnant women in South Australia, Australia, receiving ferric carboxymaltose (up to 15 mg/kg) between 24- and 40-weeks gestation (median 35 weeks). The authors observed increases in haemoglobin concentrations at 3, 6 and up to 8 weeks post-infusion (see table 6 below), although, in women with the most severe anaemia at baseline, improvements in haemoglobin were not sustained by two months postpartum.

Table 6: Increase in haemoglobin concentration at 3, 6- and 8-weeks post-infusion

	Gestational age at entry	Pre-infusion	3 weeks post infusion	6 weeks post infusion	8 weeks post infusion (post-partum)
**Mild ≥95 g/L**	34 (4)	102.1 (1.0) n = 31	108.3 (3.9)* n = 28	120.6 (2.9)* n = 18	113.1 (4.2)* n = 11
**Moderate 90-94 g/L**	36 (2)	92.6 (0.4) n = 14	105.8 (3.0)* n = 13	108.4 (3.8)* n = 5	92.7 (12.4) n = 3
**Severe <90 g/L**	34 (3)	83.7 (0.9) 20	100.2 (3.3)* n = 17	110.0 (8.1)* n = 5	93.3 (8.1) n = 4

Data are presented as means (SEM). *p < 0.01 compared to pre-infusion haemoglobin levels.

Safety: No serious adverse effects were recorded in any of the 65 women receiving an infusion. Minor side effects occurred in 13 (20%) patients. One patient required medication with Metoclopramide for nausea and vomiting. All other adverse events were self-limiting. Foetal heart rate monitoring did not indicate a drug-related adverse effect on the foetal heart pattern. Red blood cell transfusions were required by three women (4.6%) in the study cohort, all of whom had a significant peripartum haemorrhage. Adverse effects from the cohort are summarised in table 7:

Table 7: Number of women experiencing a drug-related adverse event following infusion with ferric carboxymaltose (total number of women infused n= 65)

Adverse event	n (%)
Any adverse event	13 (20)
Local (injection site irritation)	
Slight burning sensation	5 (8)
Systemic	
Hypotension	1 (1.5)
Headache	4 (6)
Nausea/Vomiting	1 (1.5)
Pruritus	2 (3)

Zeba et al.[43] undertook a prospective cohort study in 260 pregnant women between 28- and 36-weeks' gestation with haemoglobin <10g/dL in a private obstetric clinic in Faridpur, Bangladesh. All women in this study group were treated with intravenous ferric carboxymaltose as a single dose (500-1000mg) in an infusion time of 15-20minutes. *Safety:* No serious adverse events occurred, with 13% experienced minor adverse effects such as injection site irritation, headache, nausea or vomiting etc. *Efficacy:* Compared with the Hb (mean 8.9g/dl) level before infusion, the Hb (mean 10.53g/dl) level after infusion had significantly (p<0.001) increased.

Mishra et al.[44] undertook a single-arm prospective cohort trial of FCM in Ahmedabad, India, in 108 participants. Women received up to 1500mg FCM in total. *Safety*: Three women experienced local reactions, e.g. itching and irritation at the infusion site, while another five women reported systemic reactions of giddiness, headache and nausea. *Efficacy*: FCM produced an average increase of 2.1g/dL haemoglobin in 3 weeks.

Christoph P et al.[45] undertook a retrospective analysis of 206 pregnant women who had received either FCM or iron sucrose during pregnancy to assess maternal safety and tolerability. The authors observed that Mild adverse events occurred in 7.8% for ferric carboxymaltose and in 10.7% for iron sucrose. The authors also concluded that 'no sign for a negative effect on the fetus of iron infusion could be detected.'

There is limited data on safety of FCM in developing countries. However, an ongoing study in Zomba of effectiveness of FCM given in second trimester (COMREC P.02/18/2357) on maternal and neonatal outcome has not recorded any intervention-related Serious Adverse Events, nor specifically anaphylactic shock with > 97% of recruitment completed.

Use of Ferric carboxymaltose in pregnancy

Based on these studies, licensing by international regulators, including the FDA, EMA and TGA, and clinical experience, FCM has rapidly become an established treatment for anaemia in pregnancy in developed countries. For example, the British Committee for Standards in Haematology 2011 guidelines recommend that parenteral iron be considered for women from the second trimester onwards with iron deficiency anaemia who fail or are intolerant of iron[46]. Many UK health services (NHS Trusts) recommend Ferric Carboxymaltose as second-line therapy for iron deficiency anaemia in pregnancy. For example, the Mid Essex NHS Trust recommends FCM for women with persistent anaemia despite four weeks oral iron, or with haemoglobin <80g/L on any occasion, or who are intolerant of or non-compliant with oral iron; the guideline also recommends FCM as first-line therapy in all women who are anaemic beyond 34 weeks pregnancy[47]. In many cases, use is limited not by concerns of efficacy or safety, but by cost and health economic implications.

A 2012 review suggested that as FCM and other novel parenteral iron agents represent a 'milestone' in intravenous iron therapy, they should be considered as increasingly first-line for treatment of anaemia in pregnancy[47]. Here, the authors suggested that women with Hb<10g/dL should probably receive intravenous iron if they have ferritin <30mg/L, and among women with higher ferritin levels, this represents an area of research in establishing whether intravenous or oral iron should be given.

However, since the publication of the FER-ASAP trial, use of intravenous FCM in pregnancy is becoming standard treatment. For example, a recent influential 'How I Treat' review article in the leading haematology journal 'Blood' formalised the new position of parenteral iron as first-line therapy for iron deficiency anaemia in pregnancy[48]. Here, the authors suggest that **all** cases of anaemia in the second or third trimester should receive intravenous iron.

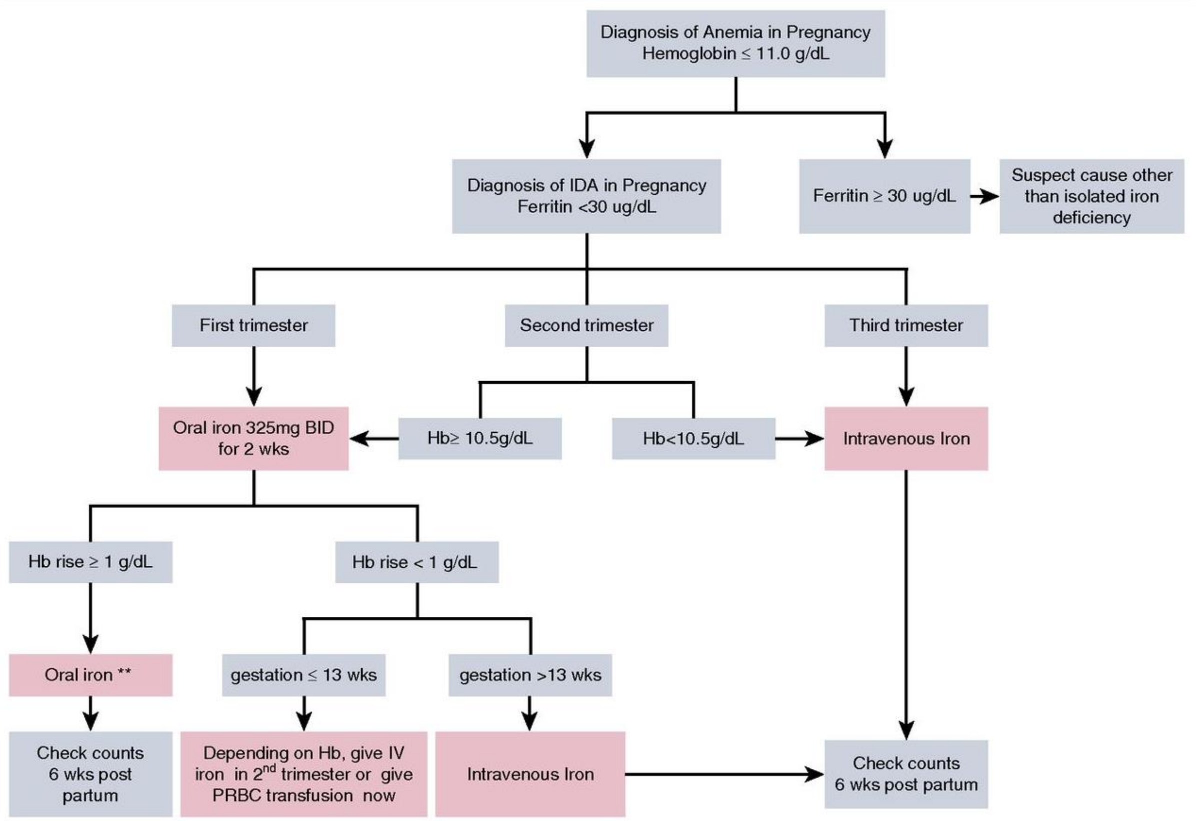

Figure 1: Decision tree on parenteral iron as first-line therapy for iron deficiency anaemia in pregnancy [From Achebe MM et al. How I treat anaemia in pregnancy: iron, cobalamin, and folate. Blood 2017; 129:940-9].

Thus, in high-income countries, FCM is now mainstream, routine therapy for iron deficiency anaemia in pregnancy. Since FCM is already licensed for the treatment of anaemia in pregnancy beyond the first trimester, using European MHRA guidelines, the REVAMP trial would be categorised as Type A = No higher than the risk of standard medical care.

Importantly, although FCM has entered routine care, there remains little data on the effects of antenatal FCM (or intravenous iron) on postpartum health or long-term infant growth and development. A study (COMREC P.02/18/2357) is currently underway in Zomba to determine the effects of FCM given in second trimester on maternal and neonatal outcome. This study will provide evidence on the effect of FCM given in third trimester on maternal and neonatal outcome. We hypothesise that the provision of FCM in third trimester will quickly raise women's haemoglobin levels and thus protect her from anaemia in the intrapartum period and facilitate the recovery postpartum. Furthermore, infants born from women who received third trimester FCM will have more iron stores compared oral iron as new information suggests that much of the iron transfers to the foetus occurs in the third trimester (Whittaker, 1991).

Hypophosphataemia and Ferric Carboxymaltose

Hypophosphatemia is increasingly recognised as a complication of intravenous treatment of FCM and is mediated by increases in intact FGF23 which, in turn, acts on the kidney to inhibit phosphate reabsorption and hence urinary phosphate wasting[72].

Ferric carboxymaltose is designed to be used within a 15-20 min window without the need for dose testing and with the added advantage of being suitable for administration in primary care and other non-hospital settings. Although the clinical replenishment of iron is effective with the use of FCM, there are a few recent studies that show patients presenting with hypophosphataemia when treated with FCM[6–8]. There are two RCTs conducted to evaluate the question of hypophosphataemia in patients with IDA that did not tolerate oral iron[6]. The results demonstrated that the incidence of hypophosphatemia was lower after Isomaltoside (a new IV iron formulation) vs FCM (trial A: 7.9% vs 75.0% [adjusted rate difference, –67.0% (95%CI, –77.4% to –51.5%)], P < .001; trial B: 8.1% vs 73.7% [adjusted rate difference, –65.8% (95%CI, –76.6% to –49.8%)], P < .001)[6] (Figure 4).

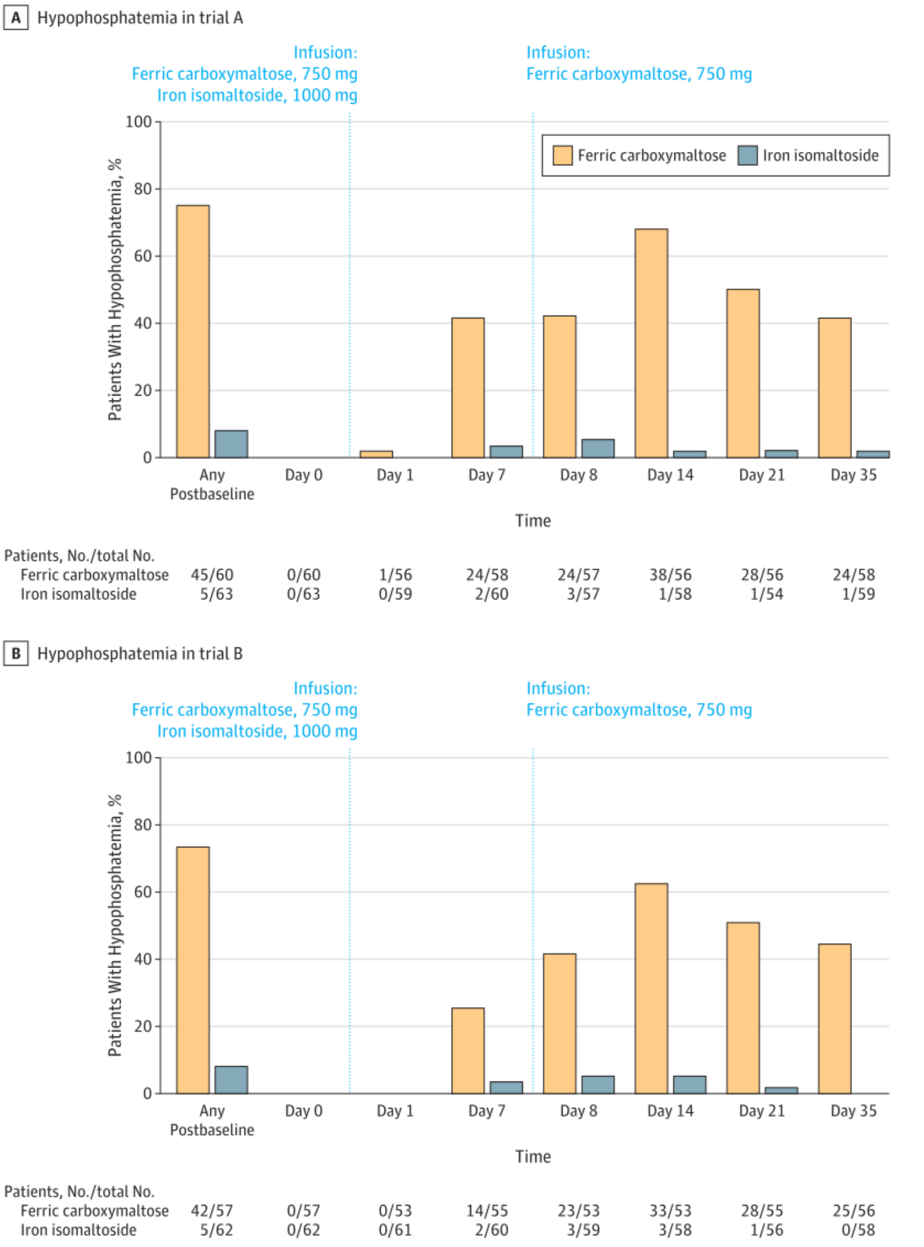

Figure 4: Hypophosphatemia in two RCT comparing FCM and Isomaltoside. The leftmost columns correspond to the primary outcome of incident hypophosphatemia at any time during the trial. The remaining columns correspond to the proportions of patients with serum phosphate level less than

2.0mg/dL at each individual time point in the safety analysis set. [Graph taken from *Wolf M, Rubin J, Achebe M, et al. Effects of Iron Isomaltoside vs Ferric Carboxymaltose on Hypophosphatemia in Iron-Deficiency Anemia: Two Randomised Clinical Trials. JAMA - J Am Med Assoc 2020; 323: 432–43.]*

Although it starts to become evident that the administration of FCM results in hypophosphatemia this is not associated with any significant clinical outcomes. Additionally, the effects of transient maternal hypophosphatemia have not been fully defined in infants; conceivably, hypophosphatemia could promote adverse growth or deficient bone mineralisation[72]. Further, the severity and duration of hypophosphatemia in these women in Africa, where nutritional deficiencies may be more common, may mean there are important variations in the effects of the drug on phosphate levels. Our ongoing REVAMP trial (COMREC P.02/18/2357) in Zomba will determine incidence of hypophosphatemia after FCM treatment. Confirming the safety and avoiding the risk of unexpected adverse events after IV iron administration is a key component of building the case for choosing the appropriate parenteral iron formulation to use in the African context.

5.1.6. *INTRAVENOUS IRON AND BENEFITS FOR THE POSTPARTUM PERIOD*

Postpartum anaemia is a frequent condition, especially in developing countries, and is mainly attributable to prepartum iron deficiency/anaemia together with blood loss during delivery [73,74]. The significance of this observation is that by adequately treating women during pregnancy, we are not only addressing the woman and the baby's health during that period but also, addressing the postpartum period[73]. This becomes even more relevant when there is evidence that demonstrates the impact that postpartum iron deficiency/anaemia has on both maternal physical and psychological health[74]. A recent review by Azami et al. [75] found that both anterpartum and postpartum anaemia increased the risk of maternal postpartum depression. However, there is limited knowledge on the long-term effect of anternatal intravenous iron on maternal physical and psychological health during the postpartum period.

Intravenous iron during pregnancy and postpartum anaemia

There are multiple benefits to the use of intravenous iron for the treatment of iron deficiency anaemia during the postpartum period, however there is very little data regarding the benefits that intravenous iron during pregnancy has on iron deficiency anaemia postpartum[76].

One study, looking retrospectively at levels of haemoglobin in the postpartum period in women who received Isomaltoside during pregnancy compared to control women who were not anaemic during pregnancy and thus did not receive Isomaltoside, showed no difference in the levels of haemoglobin between the two groups of women (mean Hb Isomaltoside group: 104 g/L vs mean Hb non-anaemic group: 107 g/L, n= 213 for both groups)[77].

6. Trial objectives and outcomes

6.1. Objectives

6.1.1. *Broad objective*

To determine the effectiveness of intravenous iron – given as FCM - once during the third trimester (27-35 weeks' gestation) compared to oral iron, as measured by anaemia recovery by 36 weeks' gestation.

6.1.2. *Specific objectives*

Efficacy (Maternal and Neonate/Infant)

- Compare the effectiveness of FCM versus standard of care oral iron on recovery from anaemia by 36 weeks' gestation.
- Compare the effectiveness of FCM versus standard of care oral iron on iron deficiency and iron deficiency anaemia by 36 weeks' gestation.
- Determine the effectiveness of intravenous iron on neonatal outcomes, including birth weight and gestation duration.
- Determine the sustained effects of intravenous iron on child outcomes including neurocognitive development, growth, anaemia, immune status and iron status
- Determine the sustained effects of intravenous iron on postpartum maternal outcomes including wellbeing and depression, at 3, 6, 9 and 12-months postpartum.

Safety (Maternal and Neonate/Infant)

- Assess the safety of intravenous iron in a real-life setting, by trial arm.
- Evaluate the effects of FCM on hypophosphatemia at 36 weeks' gestation
- Assess the frequency of unplanned visits to the clinic resulting from clinical malaria or diarrhoea by trial arm.
- Evaluate the effect of FCM on hypophosphatemia in cord blood at delivery, and in maternal and baby venous blood at three months postpartum.

6.2. Outcomes

The use of FCM for the recovery of anaemia during the late stages of pregnancy requires a strong demonstration of the efficacy and safety of this intervention. Additionally, the feasibility of applying this intervention to the health care settings where the study is being conducted will provide the necessary framework for the deployment of this strategy in other similar settings.

6.2.1. *Primary outcome*

Proportion of women with pre-delivery anaemia (defined as venous blood Hb < 11.0 g/dL at 36 weeks' gestation).

6.2.2. SECONDARY OUTCOME (MATERNAL BENEFITS)

- Mean change from baseline in maternal Hb at 36 weeks' gestation.
- Proportion of women with maternal iron deficiency (ferritin<15mg/L, sTfR/Ferritin index) at 36 weeks' gestation.
- Mean levels of maternal iron biomarkers at 36 weeks' gestation.
- Proportion of women with maternal inflammation (using C-reactive protein) at 36 weeks' gestation.
- Proportion of women with maternal postpartum haemorrhage
- Mean change from baseline in maternal Hb at 1, 3, 6, 9 and 12 months postpartum.
- Proportion of women with maternal anaemia at 1, 3, 6, 9 and 12 months postpartum
- Mean levels of iron biomarkers at 1, 3, 6, 9 and 12 months postpartum
- Proportion of women with iron deficiency (defined by ferritin<15mg/L) at 1, 3, 6, 9 and 12 months postpartum
- Proportion of women with postpartum depression (defined by EDPS > 13) at 3 months postpartum.
- Proportion of women with maternal inflammation (defined by C-reactive protein) at 3 months postpartum

6.2.3. SECONDARY OUTCOMES (NEONATE/INFANT BENEFITS)

- Mean gestation duration (in weeks)
- Proportion of sub-optimal pregnancy outcomes (defined as a composite outcome: low birthweight (<2500g); prematurity (birth <37 weeks); small for gestational age (centile score) as defined by International reference standards for gestational age-specific birthweight; stillbirth).
- Proportion of neonates born prematurely (defined as birth before 37 week's gestation)
- Proportion of infants with low birth weight (defined as a birth weight <2500g).
- Proportion of stillbirth.
- Proportion of neonatal mortality.
- Mean cord blood Hb and ferritin.
- Proportion of neonates with anaemia (with correction for gestational age).
- Child development scores measured by Evoked Response Potentials (ERP) at 6 and 12 months of age.
- Child development scores measured by Bayley Scales of Infant and Toddler development and the Malawi Development Assessment Tool at 6 and 12 months of age
- Mean child physical growth as defined by z-scores at 1, 6 and 12 months of age.
- Mean infant haemoglobin (capillary) at 6- and 12-months postpartum.
- Proportion of infants with anaemia at 6- and 12-months postpartum.
- Mean levels of iron biomarkers at 6- and 12-months postpartum.
- Proportion of infants with iron deficiency at 6- and 12-months postpartum.

6.2.4. SECONDARY OUTCOME (MATERNAL SAFETY)

- Proportion of women with at least one treatment related adverse effects (occurring immediately post-infusion, and within 7 days of commencement of treatment.

- Incidence of treatment related adverse effects (occurring immediately post-infusion, and within 7 days of commencement of treatment).
- Number of unplanned visits to the clinic (cause specific for diarrhoea and clinical malaria).
- Incidence of all-cause sick clinic visits during the antenatal, postpartum and overall participant follow-up period.
- Incidence of cause-specific sick clinic visits (in particular malaria, diarrhoea and other infectious conditions) during the antenatal, postpartum and overall participant follow-up period
- Proportion of women with placental malaria (past or active infection on histology, parasites on placental blood film).
- Proportion of women with malaria parasitaemia (asymptomatic) at 36 weeks' gestation, detected by a) microscopy, b) rapid diagnostic tests, and c) PCR.
- Proportion of women with bacteraemia at 36 weeks' gestation.
- Proportion of women with hypophosphatemia (clinical and biochemical) at 36 weeks' gestation and 3 months postpartum.

6.2.5. SECONDARY OUTCOME (CHILD SAFETY)

- Proportion of infants with hypophosphataemia (biochemical) at 4 weeks and at 6 months of age.
- Number of unplanned infant visits to the clinic (cause specific for diarrhea and clinical malaria).
- Incidence of all-cause sick clinic visits in infant by 12 months of age.
- Incidence of cause-specific sick clinic visits (in particular malaria, diarrhoea and other infectious conditions) in infant by 12 months of age.
- Proportion of infants with malaria parasitaemia (asymptomatic) 1, 3, 6, 9 and 12 months postpartum detected by a) microscopy, b) rapid diagnostic tests, and c) PCR.

7. STUDY DESIGN

This will be a Phase III two-arm open-label individual-randomised controlled trial in women with moderate or severe anaemia (capillary Hb<10g/dL) during their Third Trimester (27-35 weeks gestation) (REVAMP-TT). Participants will be randomised to receive either parenteral iron – in the form of FCM– or standard of care oral iron. The trial will be based at the TRUE centre at Zomba Central Hospital in Southern Malawi but will recruit from health centres across Zomba district. Babies and mothers will be followed up to 12 months (see below).

8. PARTICIPANT INCLUSION AND EXCLUSION CRITERIA

Inclusion Criteria[2]:

- Confirmed singleton pregnancy in the third trimester (27-35 weeks of gestation, dated by Last Menstrual Period).
- Moderate to severe anaemia not requiring an immediate blood transfusion (Hb <10g/dl).
- Negative malaria parasitaemia by RDT.

[2] Participants who are HIV positive will remain eligible to be enrolled.

- Currently afebrile with no evidence of septicaemia.
- Resident in the study catchment area of Zomba district.
- Able to deliver at health facilities within Zomba district.
- Written informed consent (including assent if <18 years old).

Exclusion criteria (at enrolment):

- Previous enrolment in REVAMP trial (REVAMP trial – P.02/18/2357)[3].
- Actively participating in another intervention trial.
- Known hypersensitivity to any of the study drugs.
- Clinical symptoms of malaria or other infection (no fever, no focal symptoms of internal infection i.e. LRTI/ diarrhoea).
- Any condition requiring hospitalisation in the next seven days or serious concomitant illness.
- Known history of sickle cell or sickle-haemoglobin C anaemia.
- Clinically low haemoglobin level requiring a blood transfusion (usually Hb <5g/dl).
- Preeclampsia

9. Study sites

The trial will be based at the TRUE centre at Zomba Central Hospital in Southern Malawi. This is a well-established clinical research site with all resources available to recruit eligible participants, prepare and administer the study drugs, monitor safety, treat adverse effects, and measure trial outcomes (22,230 women above 18 years, 4173 births per year, 13% with Hb <10g/dL respectively). This site recently participated in a large multicentre trial of antenatal anti-malarial treatment (IPTp), and is presently successfully implementing the ongoing REVAMP trial (evaluating intravenous Ferric Carboxymaltose in the second trimester of pregnancy), demonstrating the capability to undertake such studies[78]. Recruitment will be performed from health centres across Zomba district.

In order to directly define and discover aspects of possible field implementation of intravenous iron, *screening* of haemoglobin for eligibility and *administration* of the intervention (i.e. provision of the intravenous iron) will be performed *in the government health centre*. We expect to work across various health centres as part of this process. Our study team will develop procedures and deliver training to ensure that intravenous iron can be safely implemented *within the government system infrastructure.* Trial activities (i.e. enrolment, informed consent, randomisation, outcome measurement and data collection) will be performed by study staff. Still, aspects related to the implementation of the intervention (screening, selection for consideration of the trial, and provision of iron) will be done at least in some cases by government health staff trained and mentored by our team.

Once women are enrolled in the trial, we will carefully monitor them and provide transport to ensure loss to follow up is minimised. This includes the provision of transport for week 36, delivery and postpartum visits.

---

[3] REVAMP is a pregnancy trial, looking to determine whether IV ferric carboxymaltose given once during the second trimester is effective and safe in improving maternal, neonatal and infant outcomes for treatment of moderate to severe maternal anaemia among pregnant women in Blantyre and Zomba districts of Malawi. This trial is led by the same PIs as REVAMP-TT.

We will work at Zomba Central Hospital and in health centres within Zomba district, including the nine health centres described below (Figure 5). The number of new women seen at each of these nine antenatal clinics monthly is shown in brackets.

1. Likangala: (150 per month)

2. Bimbi: (75 per month)

3. Lambulira: (80 per month)

4. Domasi: (100 per month)

5. Naisi: (55 per month)

6. Matawale: (600 per month)

7. City clinic: (90 per month)

8. Sadzi: (200 per month)

9. Zilindo: (90 per month)

The expected total of new women attending these antenatal clinics is ~800 per month. These clinics are within a 30 km radius from Zomba. Based on current data, about 7% of participants screened in Zomba are eligible for the trial. Thus, we expect it will take us 12 months to recruit for the trial.

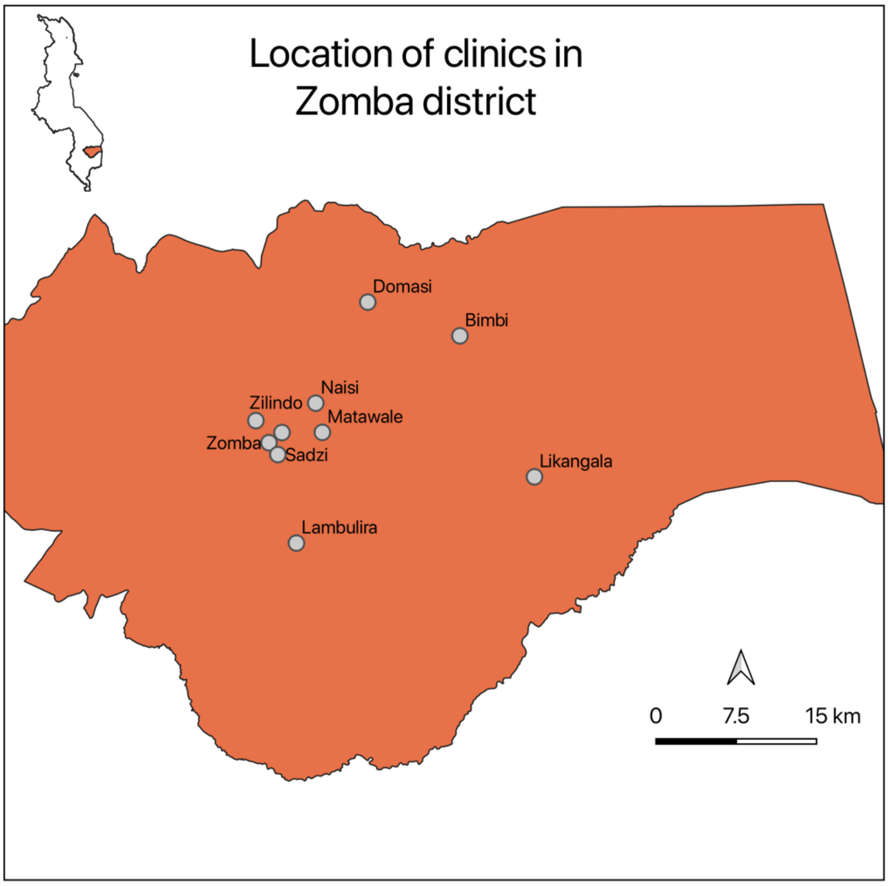

Figure 5: Approximate locations of the health centres where recruitment is going to take place.

10. TRIAL INTERVENTIONS

This is a two-arm parallel-group open-labelled trial, and the intervention groups will receive an intravenous iron given in the form of FCM at recruitment, and the control group will receive oral iron through the routine health care system. Participants will be assigned to the following:

1. **intravenous iron treatment course**: intravenous ferric carboxymaltose (FCM**)** 1000 mg for body weight ≥50 kg, or 20 mg/kg for body weight <50 kg) given over 15 min once at recruitment (Day 0);

OR

2. **Oral iron treatment course**: oral iron- 200 mg ferrous sulphate (approx. 65 mg elemental iron) twice daily for remainder of pregnancy.

If scheduled, participants will receive IPTp with SP, 1500 mg sulfadoxine and 75 mg pyrimethamine (3 fixed tablets of SP strength at 500 mg/25 mg) as recommended in the national guidelines [79]. As part of the safety assessment, IPTp-SP post-randomisation will be directly observed by study staff.

The oral iron will be given under real-life health service delivery conditions where the participant is given three months of oral iron at presentation to the antenatal clinic. This strategy is being employed as it is a key hypothesis in this trial that there is a reduced effect of oral iron on maternal anaemia due to poor adherence to the full course of treatment.

10.1. ALLOCATION TO TREATMENT

The allocation of participants to treatment will not be dictated by the clinical staff's knowledge of treatment nor will the decision to recruit a particular participant affect the order in which participants are recruited (see *section 14*).

10.2. BREAKING THE BLIND

10.2.1. *PARTICIPANT LEVEL*

This is an open-label trial. The participants are not blinded to the type of intervention they are receiving.

10.2.2. *STUDY LEVEL*

Though it is an open label trial, laboratory scientists, midwives, investigators and personnel in Australia (including statisticians in Melbourne) will be blinded to the treatment of the participant until the database has been cleaned for analysis.

11. STUDY PROCEDURES

All the recruitment and follow-up schedule are demonstrated in the figure below (Figure 6):

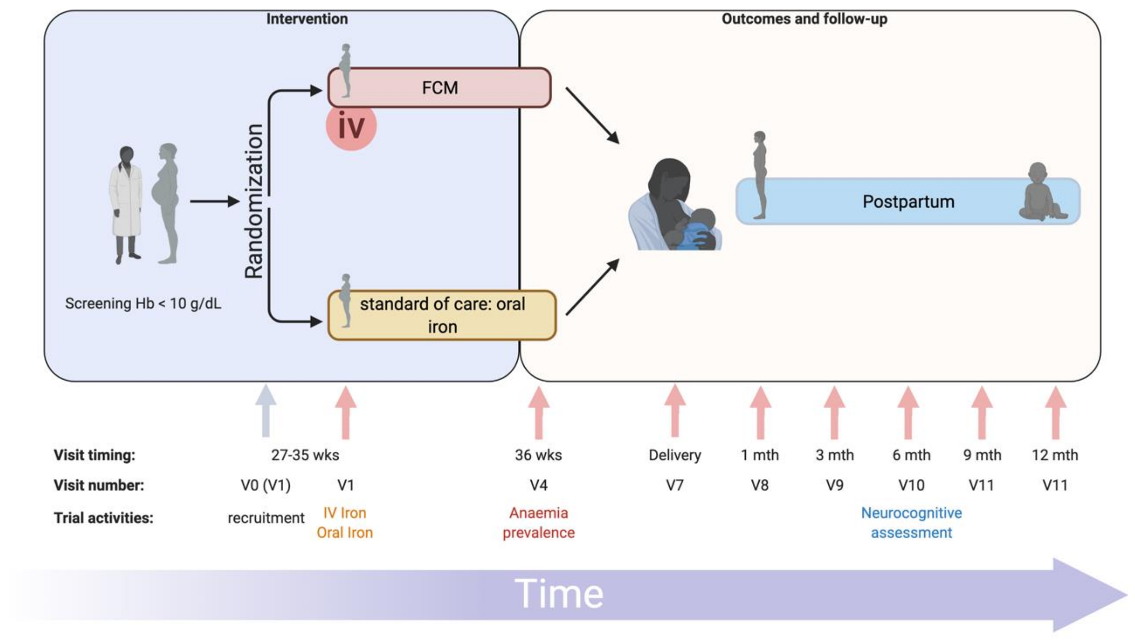

Figure 6 – REVAMP-TT Study schematic
Trial design with visit timings, visit numbers (standardised to match those of REVAMP trial – P.02/18/2357) and main trial activities are represented. FCM – Ferric Carboxymaltose; iv – Intervenous; Hb – Haemoglobin; Wks – weeks

Standard Operating Procedures (SOPs) will be used for all laboratory procedures. These SOPs were developed by senior laboratory technologist and reviewed by principal investigators (refer to Section 14 – Laboratory procedures).

11.1. Screening – visit 0[4] [day 0]

Each day at screening sites, a government-employed nurse trained to screen for study participants will begin with a health talk and include general information about the study (importance, aims and procedures). Health talks are routinely given to ANC attendees before the clinic begins. The screening nurse will then, as she/he processes ANC attendees, screen for potential participants by noting the residency, gestation on clinical examination and Hb and malaria RDT results. The nurse will then communicate to the recruitment site if there are potential participants. Recruitment – visit 1 [day 0]

If all the screening criteria (appendix 1), including giving consent (appendix 2), are met, participants will be administered a questionnaire to collect demographic and medical information before having a full physical examination by a clinician (appendix 1).

[4] Reminder that visit numbers reflect a harmonisation with the existing REVAMP protocol.

11.1.1. BLOOD SAMPLING

Experienced nurses or laboratory personnel will use standard venous blood collection SOPs to obtain the blood specimens. This will be done using aseptic techniques. Up to 3 attempts will be permitted to collect blood, after which we will just collect a capillary sample for haemoglobin estimation.

The field site will have all the equipment necessary for the sample collection and the initial storage (at 2-4°C or -20°C) before transporting to a central lab where trained laboratory personnel will be based. During transportation from the field to the district laboratory, the cold chain will be maintained. At the main laboratory, malaria microscopy (including staining) and processing of samples will be completed by the laboratory personnel.

A 10 ml venous blood sample will be collected for Full blood count (FBC) and reticulocyte count, iron (e.g. ferritin, sTfR) phosphate and inflammatory markers (e.g. C-reactive protein and alpha-1 glycoprotein). Malaria parasitaemia will be sought by Rapid Diagnostic Test. A filter paper sample of blood will be taken and stored for malaria PCR. Samples will be stored in order to assess other biochemical, immunological and metabolic factors associated with maternal health.

11.1.2. STUDY ARM ALLOCATION

A study nurse will assign the participant a Study Number. This will be from a list of consecutive numbers. The study nurse will then retrieve the sealed specific study Number-marked envelope containing the study arm allocation and open it to allocate the participant study arm. This will be done in the presence of the participant. The assigned study number will be recorded in the CRF (Clinical Record Form) and Pharmacy Log.

11.1.3. STUDY DRUG ADMINISTRATION

Those allocated to the intravenous iron arm will be given Ferric carboxymaltose – 20mg/kg up to 1000mg (women 50kg or above) in 250mL normal saline – intravenously over 15 minutes. The iron formulation~~s~~ will be diluted following procedures which shall be additionally outlined in a specific SOP. The study drug will be provided to the health worker for administration. An intravenous cannula will be inserted following standard aseptic procedure. The skin will be cleaned with ethanol, and a sterile cannula will be inserted into the forearm or hand by a skilled clinician, and the cannula will be fixed in place with a sterile Tegaderm or clinical tape. The participant will be monitored over the 15 minutes of the infusion for any adverse events, and if they develop, these will be attended to promptly and treated according to standard clinical management guidelines. The participant will be observed for a further 45 minutes. Following completion, the cannula will be removed and placed in a biohazard container, and a band-aid applied to the arm. We will follow universal precautions whilst working with sharps. Administration of iron will be done in a room equipped with a 'crash' trolley which will contains adrenaline, hydrocortisone, intravenous fluids and antihistamines. The room will also contain airway equipment (laryngoscope and endotracheal tubes) and Oxygen, for emergencies. The contents and expiry of the crash trolley and availability of oxygen will be confirmed each day by the study staff prior to administration of the drug. For those allocated to the oral iron arm, they will be provided with oral iron supplements to be taken twice daily for 90 days (or the duration of pregnancy, whichever is shorter). Women in the oral iron arm will not be encouraged to take the tablets more than the standard routine antenatal advice as we want this arm to reflect current practice as much as possible.

Women in the intravenous iron arm will be instructed not to take the oral iron; this will also be noted in the woman's health passport so as to notify any other healthcare professionals she seeks care from during her pregnancy. Women randomised to oral iron will be administered the tablets and educated according to a standard script that replicates instructions provided in routine clinical practice in Malawi.

11.1.4. *Socio-economic data collection*

All participants will be asked to provide information pertaining to their demographic as well as socioeconomic status. This will help us identify factors associated with trial outcomes, as well as to take into account various socio-economic variables associated with anaemia and general maternal health.

11.1.5. *Other procedures*

We will provide all pregnant women presenting for their first ANC visit an insecticide-treated net (ITN) and if scheduled, an SP-IPTp dose as standard policy in Malawi for the prevention of malaria in pregnancy. SP-IPTp should be given unless it was given within the previous month. Participants will then be dropped home by one of the research assistants to enable him/her to collect a detailed residency map in order to ease tracing of defaulters and allow for location of home visits. Participants will be requested to return to the research clinic (at the recruitment site) at any time that they feel ill during their pregnancy. Otherwise, they will be encouraged to attend all scheduled ANC visits at their local health centre. All participants will be requested to deliver at the research site health facility or notify the research staff if in labour or delivery occurs at any other health facility.

11.2. 36-week follow-up [± 2 days] – Visit 4

All participants will be requested to return to the research clinic at 36-weeks gestation. They will undergo a physical examination as per standard ANC procedures. Adherence to iron therapy will also be determined in the oral iron study arm *(see section 11.2.3 for details)*. This visit includes our primary outcome measure – venous haemoglobin level. They will further have some biological samples collected as follows:

11.2.1. *Blood sampling*

A 10 mL venous blood sample will be collected for Full blood count (FBC) and reticulocyte count, iron (ferritin, sTfR) phosphate and inflammatory markers (C-reactive protein and alpha-1 glycoprotein). A thin and thick smear of blood and a filter paper sample of blood will be taken and stored for malaria PCR. In case the participants present with clinical symptoms consistent with a malaria infection, malaria parasitaemia will be sought by Rapid Diagnostic Test and the participant will be referred for treatment if the results are positive. Samples will be stored in order to assess other biochemical, immunological and metabolic factors associated with maternal health.

11.2.2. *Reproductive tract microbiota*

A vaginal swab will be performed. Samples will be stored in appropriate buffers for non-diagnostic assessment of vaginal and cervical microbiomes (including *Lactobacillus spp.*, *T. vaginalis*, *C.*

trachomatis and *N. gonorrhoea*). In case there are any clinical indications that the participants have an infection or any other condition affecting the urogenital system, women will be referred back to the clinic and treated according to national guidelines.

11.2.3. *Oral iron arm - Compliance*

Women randomised to the oral iron arm of the study will be encouraged to provide any remaining iron tablets so that a pill count can be done to assess their adherence to the treatment.

11.2.4. *Maternal psychological health*

All women will be asked to complete the short version of the Depression, Anxiety and Stress Scale (DASS-21) form and the Edinburgh Postnatal Depression Scale (EPDS) form. These tools are designed to screen women for any symptoms of emotional distress.

11.3. Delivery [+1 day] – visit 7

All participants will be encouraged to deliver at Zomba Central Hospital. Participants delivering at home or at other health facilities will be asked to come within 24 hours to the research site for assessment. If this is impossible, a study field worker will travel to the participant's home to collect all relevant study data. A detailed physical examination will be done on admission and detailed clinical data on the progress of the delivery. A maternal venous blood sample will be taken (for laboratory tests as below). Delayed cord clamping procedures will be standardized. Immediately after delivery, we will record Apgar scores, and the neonate will have a full physical examination, including measurement of birth weight, length, and details of any congenital malformations. We will record whether the birth was vaginal, vaginal but assisted, or caesarean, and the indications for assisted/ caesarean births; we will also record the occurrence of complications such as haemorrhage, and episiotomy.

11.3.1. *Blood sampling*

Placental and cord blood will also be collected for malaria microscopy and PCR. Blood samples will be stored in order to assess other biochemical, immunological and metabolic factors associated with maternal, neonatal or infant/child health. In case the participants present with clinical symptoms consistent with a malaria infection, malaria parasitaemia will be sought by Rapid Diagnostic Test and the participant will be referred for treatment if the results are positive

11.3.2. *Placental tissue - histology and storage*

The placenta will be weighed, and results recorded. A histological full-thickness placental sample about 2 cm long x 1 cm wide will be collected into 40 mL of 10% neutral buffered formalin for malaria histological staining and examination. Sections of the placenta will be stored and may be used for subsequent immunological, metabolic and/or biochemical analyses related to maternal, neonatal or infant/child health. Mothers will be informed these samples may be sent overseas for analysis in cases where the relevant assays are not available in Malawi.

11.3.3. INFANT NEURODEVELOPMENT

We will measure Auditory Brainstem Responses (ABRs) within 48 hours of delivery. ABRs are reflective of maturation of the auditory pathway at various levels (e.g. acoustic nerve level to brainstem level). There is a rapid maturation of the ABR during the perinatal period that is influenced by the degree of myelination, neuronal development, synaptic function, and axonal growth in the auditory nervous system (i.e., changes we expect to be influenced by the level of iron). This assessment does not require any participation by the child; indeed, it is possible when the child is sleeping in their mother's arms. A signal (sound) is provided to the child, and the response is measured. The test is non-invasive, and only requires the fitting of electrodes on the scalp. It does not pose any risk to the child and take less than 20 minutes per child. Drs Larson, Johnson and Bode (co-investigators) are experts in this test.

11.4. 28 DAYS POSTPARTUM [± 2 DAYS] – VISIT 8

All participants will be requested to return to the research clinic together with their infants for a detailed medical examination of both the mother and the baby.

11.4.1. BLOOD SAMPLING

A 10ml maternal venous blood sample will be collected for Full blood count (FBC) and reticulocyte count, iron (e.g. ferritin, sTfR) phosphate and inflammatory markers (e.g. C-reactive protein and alpha-1 glycoprotein). A filter paper sample of blood will be taken and stored for malaria PCR. In case the participants present with clinical symptoms consistent with a malaria infection, malaria parasitaemia will be sought by Rapid Diagnostic Test and the participant will be referred for treatment if the results are positive.

We will also collect a venous sample of up to 3 mL of the infant's blood for malaria RDT, microscopy, filter paper PCR and iron indices. In case the participants present with clinical symptoms consistent with a malaria infection, malaria parasitaemia will be sought by Rapid Diagnostic Test and the participant will be referred for treatment if the results are positive. Samples will be stored in order to assess other biochemical, immunological and metabolic factors associated with maternal, neonatal or infant/child health.

11.4.2. REPRODUCTIVE TRACT MICROBIOTA

A vaginal swab will be performed. Samples will be stored in appropriate buffers for non-diagnostic assessment of vaginal and cervical microbiomes (including *Lactobacillus spp.*, *T. vaginalis*, *C. trachomatis* and *N. gonorrhoea*). In case there are any clinical indications that the participants have an infection or any other condition affecting the urogenital system, women will be referred back to the clinic and treated according to national guidelines.

11.4.3. BREASTMILK

We will request to collect 2 mL of breastmilk. These samples will be stored in order to assess iron content, as well as to assess other molecular, biochemical, immunological and metabolic factors associated with maternal, neonatal or infant/child health.

11.4.4. CHILD ANTHROPOMETRY

Infant weight, length, head circumference and adiposity will be measured using established, calibrated tools.

11.4.5. CHILD VACCINATION AND VITAMIN A SUPPLEMENTATION STATUS

Details of child's vaccination and vitamin A supplementation status will be recorded from medical records.

11.4.6. INFANT NEURODEVELOPMENT

We will measure Auditory Brainstem Responses (ABRs) while the child is sleeping. A signal (sound) is provided to the child, and the response is measured. The test is non-invasive, and only requires the fitting of electrodes on the scalp. It does not pose any risk to the child and take less than 20 minutes per child.

11.5. 3 MONTHS POSTPARTUM [± 14 DAYS] – VISIT 9

All participants will be requested to return to the research clinic together with their infants for a detailed medical examination of both the mother and the baby.

11.5.1. BLOOD SAMPLING

A 10ml maternal venous blood sample will be collected for Full blood count (FBC) and reticulocyte count, iron (e.g. ferritin, sTfR) phosphate and inflammatory markers (e.g. C-reactive protein and alpha-1 glycoprotein). A filter paper sample of blood will be taken and stored for malaria PCR. In case the participants present with clinical symptoms consistent with a malaria infection, malaria parasitaemia will be sought by Rapid Diagnostic Test and the participant will be referred for treatment if the results are positive

We will also collect a venous sample of up to 3 mL of the infant's blood for malaria RDT, microscopy, filter paper PCR and iron indices. In case the participants present with clinical symptoms consistent with a malaria infection, malaria parasitaemia will be sought by Rapid Diagnostic Test and the participant will be referred for treatment if the results are positive. Samples will be stored in order to assess other biochemical, immunological and metabolic factors associated with maternal, neonatal or infant/child health.

11.5.2. CHILD ANTHROPOMETRY

Infant weight, length and head circumference will be measured using established, calibrated tools.

11.5.3. CHILD VACCINATION AND VITAMIN A SUPPLEMENTATION STATUS

Details of child's vaccination and vitamin A supplementation status will be recorded from medical records.

11.5.4. MATERNAL PSYCHOLOGICAL HEALTH

Maternal psychological health will be measured using the short version of Depression, Anxiety and Stress Scale (DASS-21) and EPDS. These tools provide a self-report measure of depression, anxiety and stress. Mother-child interaction will be assessed by Mother-Infant Bonding Scale (MIBS), which is a modified version of the Postpartum Bonding Questionnaire.

11.6. 6 MONTHS POSTPARTUM [± 14 DAYS] – VISIT 10

All participants will be requested to return to the research clinic together with their infants for a detailed medical examination of both the mother and the baby.

11.6.1. BLOOD SAMPLING

A 10ml maternal venous blood sample will be collected for Full blood count (FBC) and reticulocyte count, iron (e.g. ferritin, sTfR) phosphate and inflammatory markers (e.g. C-reactive protein and alpha-1 glycoprotein). A filter paper sample of blood will be taken and stored for malaria PCR. In case the participants present with clinical symptoms consistent with a malaria infection, malaria parasitaemia will be sought by Rapid Diagnostic Test and the participant will be referred for treatment if the results are positive. For consistency with measurement of Hb (as a primary outcome).

We will also collect a venous sample of up to 3 mL of the infant’s blood for malaria RDT, microscopy, filter paper PCR and iron indices. In case the participants present with clinical symptoms consistent with a malaria infection, malaria parasitaemia will be sought by Rapid Diagnostic Test and the participant will be referred for treatment if the results are positive.

Samples will be stored in order to assess other biochemical, immunological and metabolic factors associated with maternal, neonatal or infant/child health.

11.6.2. BREASTMILK

In case mothers are still breastfeeding, we will request to collect 2 mL of breastmilk. These samples will be stored in order to iron content, as well as to assess other molecular, biochemical, immunological and metabolic factors associated with maternal, neonatal or infant/child health.

11.6.3. CHILD ANTHROPOMETRY

Infant weight, length and head circumference will be measured using established, calibrated tools.

11.6.4. CHILD VACCINATION AND VITAMIN A SUPPLEMENTATION STATUS

Details of child’s vaccination and vitamin A supplementation status will be recorded from medical records.

11.6.5. INFANT NEURODEVELOPMENT

We will measure Auditory Brainstem Responses (ABRs) while the child is sleeping. A signal (sound) is provided to the child, and the response is measured. The test is non-invasive, and only requires the

fitting of electrodes on the scalp. It does not pose any risk to the child and take less than 20 minutes per child.

We will measure Event-Related Potentials (ERPs) using electroencephalography (EEG) methods (see Neurocognitive Assessments Used in Infant Follow-Up section below).

We will measure Bayley Scales of Infant Development as well as the MDAT (*see section 13. below for details*).

11.7. 9 MONTHS POSTPARTUM [± 14 DAYS] – VISIT 11

All participants will be requested to return to the research clinic together with their infants for a detailed medical examination of both the mother and the baby.

11.7.1. *BLOOD SAMPLING*

A 10ml maternal venous blood sample will be collected for Full blood count (FBC) and reticulocyte count, iron (e.g. ferritin, sTfR) phosphate and inflammatory markers (e.g. C-reactive protein and alpha-1 glycoprotein). A filter paper sample of blood will be taken and stored for malaria PCR. In case the participants present with clinical symptoms consistent with a malaria infection, malaria parasitaemia will be sought by Rapid Diagnostic Test and the participant will be referred for treatment if the results are positive.

We will also collect a venous sample of up to 3 mL of the infant's blood for malaria RDT, microscopy, filter paper PCR and iron indices. In case the participants present with clinical symptoms consistent with a malaria infection, malaria parasitaemia will be sought by Rapid Diagnostic Test and the participant will be referred for treatment if the results are positive.

Samples will be stored in order to assess other biochemical, immunological and metabolic factors associated with maternal, neonatal or infant/child health.

11.7.2. *CHILD ANTHROPOMETRY*

Infant weight, length and head circumference will be measured using established, calibrated tools.

11.7.3. *CHILD VACCINATION AND VITAMIN A SUPPLEMENTATION STATUS*

Details of child's vaccination and vitamin A supplementation status will be recorded from medical records.

11.8. 12 MONTHS POSTPARTUM [± 14 DAYS] – VISIT 12

All participants will be requested to return to the research clinic together with their infants for a detailed medical examination of both the mother and the baby. This will be the final visit for the study.

11.8.1. *BLOOD SAMPLING*

A 10ml maternal venous blood sample will be collected for Full blood count (FBC) and reticulocyte count, iron (e.g. ferritin, sTfR) phosphate and inflammatory markers (e.g. C-reactive protein and alpha-

1 glycoprotein). A filter paper sample of blood will be taken and stored for malaria PCR. In case the participants present with clinical symptoms consistent with a malaria infection, malaria parasitaemia will be sought by Rapid Diagnostic Test and the participant will be referred for treatment if the results are positive.

We will also collect a venous sample of up to 3 mL of the infant's blood for malaria RDT, microscopy, filter paper PCR and iron indices. In case the participants present with clinical symptoms consistent with a malaria infection, malaria parasitaemia will be sought by Rapid Diagnostic Test and the participant will be referred for treatment if the results are positive.

Samples will be stored in order to assess other biochemical, immunological and metabolic factors associated with maternal, neonatal or infant/child health.

11.8.2. *Reproductive tract microbiota*

A vaginal swab will be performed. Samples will be stored in appropriate buffers for non-diagnostic assessment of vaginal and cervical microbiomes (including *Lactobacillus spp.*, *T. vaginalis*, *C. trachomatis* and *N. gonorrhoea*). In case there are any clinical indications that the participants have an infection or any other condition affecting the urogenital system, women will be referred back to the clinic and treated according to national guidelines.

11.8.3. *Breastmilk*

In case mothers are still breastfeeding, we will request to collect 2 mL of breastmilk. These samples will be stored in order to assess iron content, as well as to assess other molecular, biochemical, immunological and metabolic factors associated with maternal, neonatal or infant/child health.

11.8.4. *Child anthropometry*

Infant weight, length and head circumference will be measured using established, calibrated tools.

11.8.5. *Child vaccination and vitamin A supplementation status*

Details of child's vaccination and vitamin A supplementation status will be recorded from medical records.

11.8.6. *Infant Neurodevelopment*

We will measure Auditory Brainstem Responses (ABRs) while the child is sleeping. A signal (sound) is provided to the child, and the response is measured. The test is non-invasive, and only requires the fitting of electrodes on the scalp. It does not pose any risk to the child and take less than 20 minutes per child.

We will measure Event-Related Potentials (ERPs) using electroencephalography (EEG) methods (see Neurocognitive Assessments Used in Infant Follow-Up section below).

We will measure Bayley Scales of Infant Development as well as the MDAT (*see section 13. below for details*).

11.9. UNSCHEDULED SICK VISIT [ANYTIME DURING STUDY FOLLOW-UP]

Participants will be encouraged to attend the research clinic when sick. The participants will be managed according to standard ANC management guidelines. Blood sample for malaria RDT (and microscopy if RDT positive) will be taken. Some of the blood will be put on filter paper for malaria PCR and for resistance testing for positive samples.

12. LABORATORY PROCEDURES

At Zomba Central Hospital, we have a laboratory with lab technologists and technicians where initial sample processing will be done. The site labs have small laboratory equipment for sample collection, separation and storage (at -20°C). They have a microscope and area for staining of malaria and other slides before reading. Lab technologists and technicians will use SOPs for all their laboratory processing of samples. These SOPs will be developed by senior laboratory technologist and reviewed by principal investigators. Samples will be transferred for long-term storage to the College of Medicine, Blantyre campus where we have -80°C freezers. The temperature of the freezers is monitored daily, and there are backup systems in case they break down. Some samples will be exported out of the country for more advanced assays that are currently not available in Malawi (appendix 3).

12.1. FULL BLOOD COUNT

A Sysmex haematology analyser will be used for full blood counts to determine the Hb level and red cell indices, as determined by the manufacturer.

12.2. MALARIA TESTING

Testing for malaria at screening and at presentation of clinical symptoms will be done using an RDT and following the manufacture's guidelines. Whole blood will be placed on an RDT test strip and read. Positive RDT results will be confirmed by microscopy. The smears will be stained with May-Grunwald-Giemsa using standard techniques. All smears will be double read by certified laboratory technicians (each reading blinded of the other's results). Discrepant results (positive/negative or a 50% difference in the parasite counts) will be settled by a third reader. At all visits, both thin and thick blood smears will be prepared and read at a later time for the presence of asymptomatic parasitaemia. Molecular diagnosis of sub-microscopic parasitaemia and evaluation of markers of resistance to antibiotics will be sought by PCR done of dried-blood spot samples.

Blood from participants in whom parasites are detected can be further analysed for parasite clonality, levels of parasite gene expression and the abundance and characteristics of parasite proteins.

12.3. PLACENTAL HISTOLOGY

Placental histology will be performed using standard approaches. Placental biopsies will be fixed in formalin, embedded in paraffin and 5 mm sections cut onto glass slides. After rehydration, the tissue sections will be stained with Giemsa and/or haematoxylin, and eosin passed through graded alcohols

and sealed with a coverslip. Tissue blocks will be kept for evaluation of metabolic, molecular and immunopathological parameters associated with maternal and child health.

12.3.1. *Malaria evaluation*

Examination, by light microscopy, will note the presence of malaria-infected erythrocytes, malaria pigment in fibrin, and malaria pigment in host leukocytes in the placental blood spaces. Placentas will be classified as uninfected; past infection (malaria pigment, no parasites); or active infections (parasites with or without malaria pigment).

12.4. Blood samples: separation and long-term storage

Blood will be drawn using standard venipuncture techniques and the serum will be separated from the blood cells as soon as possible. Samples will be allowed to clot for one hour at room temperature, centrifuged for 10 minutes at 4 °C, and serum extracted. Inflammatory markers and iron markers such as Serum C-reactive protein and alpha-1 glycoprotein, ferritin, will be analysed on Modular P800 and Modular Analytics E170 systems (Roche).

Blood will also be stored in appropriate preservatives to enable further analysis of genetic, immune, metabolic and molecular aetiologies related to anaemia, infection, developmental and other clinical outcomes in pregnancy and during the first 12 months of life. Residual whole blood (EDTA) samples from participants at baseline will be stored for future extraction of DNA and/or RNA. These DNA samples will be used for non-diagnostic studies which will have no clinical relevance to the participants. We will analyse genetic differences (single nucleotide polymorphisms) which may be linked with anaemia, nutrition, development and growth, or infection status. Samples and results of genetic analysis will not be released to anyone other than the researchers or their colleagues helping with bioinformatic analysis and will be fully deidentified.

These assays will be carried out at the Walter and Eliza Hall Institute of Medical Research, in Melbourne, Australia, or a collaborating laboratory if unavailable at that Institute; all experiments will be undertaken with full consultation and involvement of the Malawian investigators.

12.5. Vaginal and gut microbiome analysis

Vaginal and faecal microbiome analysis will be undertaken at the Walter and Eliza Hall Institute of Medical Research in Melbourne, Australia or partners, using approaches such as 16S rRNA gene sequencing or metagenomics on microbial DNA.

12.6. SUMMARY OF LABORATORY MEASUREMENTS

			Visit 0	Visit 1	Visit 4	Visit 7	Visit 8	Visit 9	Visit 10	Visit 11	Visit 12	Sick Visit Unscheduled	
			May occur at visit 1	***Week 28-34***	***36 weeks***	***Delivery***	***28 days postpartum***	***3 months postpartum***	***6 months postpartum***	***9 months postpartum***	***12 months postpartum***		
	**Sample type**	**Test**	**MEASUREMENTS on the MOTHER**										**Is the test locally available?**
**1**	**Venous blood**	Haemoglobin (Hb)	X										Yes
**2**		Malaria RDTs (mRDT)		X								X	Yes
**3**		Molecular evaluation of resistance genes if Filter paper is mRDT positive										X	No
**4**		FBC (full blood count with Hb)		X	X		X	X	X	X	X		Yes
**5**		Malaria microscopy		X	X	X	X	X	X	X	X	X	Yes
**6**		Hb (by HemoCue)		X	X								Yes
**7**		Malaria PCR		X	X	X	X	X	X	X	X		No
**8**		Parasite gene content and expression analysis (if malaria positive)		X	X	X	X	X	X	X	X		No
**9**		Phosphate levels		X	X	X	X		X		X		No
**10**		Ferritin (serum)		X	X		X	X	X	X	X		No
**11**		sTfR (serum)		X	X		X	X	X	X	X		No

			Visit 0	Visit 1	Visit 4	Visit 7	Visit 8	Visit 9	Visit 10	Visit 11	Visit 12	Sick Visit Unscheduled	
			May occur at visit 1	*Week 28-34*	*36 weeks*	*Delivery*	*28 days postpartum*	*3 months postpartum*	*6 months postpartum*	*9 months postpartum*	*12 months postpartum*		
**12**		Immune/metabolic markers		X	X		X	X	X	X	X		No
**13**		CRP (C-reactive protein)		X	X		X	X	X	X	X		No
**14**		Alpha-1 glycoprotein		X	X		X	X	X	X	X		No
**15**	**Reproductive tract swab**	for non-diagnostic analysis of STIs and microbiome composition			X		X				X		Yes/No
**16**	**Placental biopsy**	for Malaria immunohistological staining and examination				X							Yes/No
**17**		for immune/metabolic markers				X							No
**18**	**Placental blood**	for microscopic malaria test				X							Yes
**19**		for malaria PCR				X							No
**20**		for Immune/metabolic markers				X							No
**21**	**Breastmilk sample**	For iron and nutrition markers					X	X	X		X		No
**22**		MEASUREMENTS on the INFANT											
**23**	**Cord blood**	Malaria microscopy				X							Yes
**24**		Hb by Hemocue®				X							Yes
**25**		for Immune/metabolic markers				X							No
**26**		Full Blood Count				X							Yes
**27**		Malaria PCR				X							No

			**Visit 0**	**Visit 1**	**Visit 4**	**Visit 7**	**Visit 8**	**Visit 9**	**Visit 10**	**Visit 11**	**Visit 12**	**Sick Visit Unscheduled**	
			May occur at visit 1	***Week 28-34***	***36 weeks***	***Delivery***	***28 days postpartum***	***3 months postpartum***	***6 months postpartum***	***9 months postpartum***	***12 months postpartum***		
**28**		Ferritin (serum)				X							No
**29**		sTfR (serum)				X							No
**30**	**Venous blood**	malaria RDT										X	Yes
**31**		Malaria microscopy					X	X	X	X	X	X	Yes
**32**		Malaria PCR					X	X	X	X	X		No
**33**		Hb by Hemocue					X	X	X	X	X		Yes
**34**		Ferritin (serum)					X	X	X	X	X		No
**35**		sTfR (serum)					X	X	X	X	X		No
**36**		Full Blood Count					X	X	X	X	X		Yes
**37**		CRP (C-reactive protein)					X	X	X	X	X		No
**38**		Alpha-1 glycoprotein					X	X	X	X	X		No

FBC, full blood count; HV, home visit; Hb, haemoglobin; mRDTs, malaria rapid diagnostic tests; sTfR, serum transferrin receptor; CRP, C-reactive protein; AGP, alpha 1-acid glycoprotein

12.7. Neurocognitive assessments used in infant follow up

We will use highly sensitive assessments of memory and executive control to examine the effects of iron on children's brain development. Beyond the important effects of iron on the brain, we will also examine the neural mechanisms underlying cognitive development and how infection exposures and environmental adversity in this context may shape neural development. Event-Related Potentials (ERPs) are ideal measures of infant cognitive function because they are sensitive to, and reflective of, brain functions directly affected by iron. They are able to detect subtle yet meaningful delays that have long-term consequences[80–82]. ERPs are neurophysiologic measures that rely on recordings of the brain's electrical activity via electroencephalogram (EEG) in response to stimuli. ERPs can be recorded noninvasively at the scalp surface in minutes. They are used in routine clinical assessments in children in tertiary care hospitals across Australia, America, and Europe, typically in the investigation and diagnosis of epilepsy and seizure disorders[83,84].

Observational studies have reported significant associations between iron status in young children and ERP-derived neural correlates of memory and executive control[85–87]. For instance, Geng et al.[85] reported that two-month-old Chinese children who were iron-sufficient at birth were able to recognize their mother's voice from a stranger's voice whereas iron-deficient children were not, as indicated by the ERP late slow wave component in frontal-central and parietal-occipital locations of the scalp. Another study in American children found that, compared to iron-deficient children, iron-sufficient 9-month-olds showed a larger attentional response to their mother's face compared to a stranger's, indicated by the ERP negative component[87]. They also reported a better updating of memory for the stranger's face, indicated by the positive slow wave component. Children with iron deficiency anaemia showed similar patterns only at 12 months of age, consistent with delayed cognitive development in this group[87]. However, to date, no randomized controlled trial (RCT) of iron interventions has used ERPs.

Differences in cognitive functioning detected using ERPs early in life have been shown to be predictive of later cognition[88,89]. For instance, follow up studies of children with and without iron deficiency anaemia in the first year of life found significant differences using electrophysiology in the auditory and visual systems of the brain at four years of age and inhibitory control at ten years of age[88,89]. Results from longitudinal studies support the hypothesis that iron deficiency in early life causes hypomyelination and has long term detrimental effects on brain function, which can be detected with sensitive measures such as ERPs.

We will use ERPs to measure the impartial and direct effects of iron on brain development, by examining functions and areas of the brain which are especially sensitive to iron, to ensure we capture meaningful effects of iron supplementation on early cognition. Our study intends to build on observational ERP literature, leverage an existing rigorously controlled field RCT, to be the first study to examine the effects of antenatal high dose iron interventions on ERP-derived cognitive functions in young children.

12.7.1. *Procedures for ERPs*

ERPs will be measured in response to auditory and visual stimuli, recorded from 32 scalp electrode sites, using established procedures. The study centres will have a designated ERP testing room,

outfitted with the following equipment: dark curtains, an air conditioning unit, and a screen for visual stimuli.

When caregivers and their children return for the 6-month visit at which ERPs will be measured, they will be walked through each procedure in detail. They will be shown a video detailing the procedure and objectives of the research. Children will be fitted with an ERP cap (resembling a swim cap) and seated on their mothers' laps in front of a screen. Testing will begin after a period of familiarization with the cap, screen, instrument, and with the tester. We intend to spend no more than 30 minutes with each child to avoid poor data collection when a child is tired.

Visual and auditory stimuli for ERP tasks will be culturally appropriate. The order of the ERP tasks will alternate between children to ensure that the order does not bias performance. On a daily or weekly basis, recorded data will be shared with the team in Australia, where it will be processed (to separate adequate from poor EEG data) and averaged for components of interest. We will have regular online meetings between the Malawian and Australian team members to discuss and troubleshoot any issues.

In the cases where a child is not able to complete the ERP session, they will be invited to return the following day for a repeat session.

Feasibility: Electrophysiologic methods have advanced greatly in recent years, and simplifications to equipment and technique mean these methods are now portable and can be used to assess brain development even in remote, low-income settings, like Zomba. ERPs have been used in a field-based study in Pakistan and are currently being used in other resource-limited settings, such as The Gambia and South Africa. We are presently undertaking a large ERP study as part of a major trial of iron interventions in rural Bangladesh.

We will assess children's Auditory Brainstem Responses (ABRs) at birth, one month and six months of age. ABRs are reflective of maturation of the auditory pathway at various levels (e.g. acoustic nerve level to brainstem level). There is a rapid maturation of the ABR during the perinatal period that is influenced by the degree of myelination, neuronal development, synaptic function, and axonal growth in the auditory nervous system (i.e., changes we expect to be influenced by the level of iron). ABRs are already being used in our research sites in Malawi i.e. at Zomba Central Hospital and in Blantyre (Limbe Health Centre).

Behavioural child development measurements: At six and twelve months of age, we will administer the Bayley Scales of Infant and Toddler Development III, the Malawi Developmental Assessment Tool (MDAT) and a behavioural test of memory and attention. The results from the behavioural tests will be used to assess other domains of child development than those gathered using ERPs (i.e., fine and gross motor, cognitive, language, and socio-emotional development), and will be used to corroborate the ERP results. The use of the Bayley Scales and MDAT allows us to describe the findings of the interventions to policymakers, clinicians and other end-users of the trial results.

13. ALLOCATION OF PARTICIPANTS TO TRIAL ARMS

Participants will be randomly allocated to one of the two treatments arms with 1:1 allocation via a computer-generated randomisation schedule of randomly permuted blocks stratified by site to

achieve balance between the arms within each site. The randomisation list will be generated by an independent statistician at the University of Melbourne (Australia) who will not reveal the block size until the database is ready for unblinding.

Individual participant codes will be pre-packed in envelopes, sealed and held securely at research sites. The eligible participants who have met all inclusion/exclusion criteria will receive study medication assigned to the next available randomisation number during the randomisation study visit. The study medication shall be determined after the research staff open the specific participant envelope, which will prescribe the participant's group allocation.

Although the trial is open-label, midwives collecting birth data, laboratory scientists and investigators and personnel in Australia (including statisticians in Melbourne) will be blinded to the allocation code, until the database has been cleaned for analysis.

14. Assessment of Safety

14.1. Precautions in delivering iron intravenous formulations

As discussed above, there remains a theoretical risk of hypersensitivity reactions associated with the infusion of FCM. For this reason, intravenous iron formulations will only be administered in health care centres where appropriate equipment and facilities are available for the treatment of any severe reactions, even though these are rare.

The participants will be monitored for signs and symptoms of hypersensitivity during and after administration of the intravenous infusion for at least 30 min and until clinically stable. The trial teams will carry the necessary equipment to provide urgent medical care.

Protocol for delivering FCM*:*

- Iron formulations will be administered as an infusion in 250 mL of 0.9% sodium chloride
- Infusion rate will be 250 mL over 15 minutes.
- The patency of the intravenous cannula will be confirmed before commencing any infusion to ensure there is no extravasation of iron, which can cause a permanent stain. Connect a 50 mL 0.9% sodium chloride flush and allow this to infuse by gravity. If the flush does not work, do not use the line.
- All women will be observed for signs of adverse reaction which may be acute or delayed (see below).
- A Clinical Officer will be available and able to reach the infusion room within 5 minutes
- Common adverse reactions to intravenous iron include:
    - Headache, dizziness

  - Injection site reactions[5]
  - Hypertension
  - Elevated liver enzymes
  - Hypophosphataemia
- Less common reactions include:
  - Nausea, abdominal pain, constipation, diarrhoea
  - Hypersensitivity including anaphylaxis
  - Paraesthesia and dysguesia
  - Hypotension
  - Tachycardia
  - Flushing
  - Back, joint or muscle pain
  - Pruritis and urticaria
  - Pyrexia, fatigue and malaise
  - Dyspnoea and bronchospasm
  - Syncope
- A member of the study nursing staff will remain in the same room as the patient during the infusion and for the 30 minutes following patient, to ensure any serious adverse reaction is rapidly detected and that the infusion can either be stopped or other appropriate treatments given.
- Vital signs are to be monitored prior to infusion, 5 minutes after the start of the infusion, at the end of infusion, every prior to discharge, and if the participants feel unwell:
  - Heart Rate
  - Blood Pressure
  - Oxygen Saturations
  - Respiratory Rate
  - Conscious State
  - Temperature

Foetal Heart Rate will be monitored prior to infusion, at the end of infusion and prior to discharge.

[5] injection site erythema, -swelling, -burning, -pain, -bruising, -discolouration, -extravasation, -irritation, -reaction

14.2. Definitions of adverse events

According to the latest EU guideline contained in **Detailed guidance on the collection, verification and presentation of adverse event/reaction reports arising from clinical trials on medicinal products for human use ('CT-3')** 2011/C 172/01, an Adverse event is 'Any untoward medical occurrence in a patient or clinical trial subject administered a medicinal product and which does not necessarily have a causal relationship with this treatment'[71].

14.3. Recording of adverse events

14.3.1. *The time period for collecting adverse events*

Adverse events and adverse reactions (non-serious and serious) will be collected from the time consent is given until the participant completes the study (the final visit or withdrawal). All AEs and SAEs will be followed until resolution or stabilisation.

14.3.2. *Method of capturing adverse events*

At scheduled clinic visits, we will record any AE spontaneously reported by the subject and/or observed by the study staff, as well as any AEs identified by means of a standard question to the subject such as "have you experienced any health problems since the last visit/ the last questioning?". In addition, AEs will be documented from physical examination findings, clinically significant lab results or other documents (including correspondence from their primary care physician) that are relevant to participant safety.

14.3.3. *Documentation of adverse events data*

AEs will be recorded in the adverse event section of the CRF. For the purposes of this trial the investigators will record all adverse events, irrespective of their perceived relationship with the trial interventions, with the following exceptions:

- Conditions that are present at screening and do not deteriorate will not be considered adverse events.
- Abnormal laboratory values will not be considered adverse events unless deemed clinically significant by the investigator and documented as such (see section 17.3.9).

For each AE, a description of the event, date of onset and resolution (and if applicable time), its severity, whether it constitutes a SAE or not, any action taken (e.g. other treatment given, change to study treatment, follow-up laboratory tests) and the outcome (continuing or resolved), will be given along with the investigator's assessment of relationship to study drug. Details of changes to the dosage schedule, any other treatment given, or follow-up laboratory tests should be recorded on the appropriate pages of the CRF

All SAEs must be reported according to procedures described in section **17.4 - reporting of serious adverse events**.

If a diagnosis of the subject's condition has been made, then the diagnosis should be recorded as the adverse event. However, if a diagnosis of the subject's condition has not been made then the individual signs or symptoms should be recorded separately.

If an AE changes in frequency or severity during the study period, a new record of the AE should be recorded in the CRF.

14.3.4. *Assessment of adverse event severity*

The investigator will use clinical judgement to make an assessment of the severity of each AE.

14.3.5. *Assessment of causality of adverse events*

The investigator will use clinical judgement to assess the relationship between investigational product and the occurrence of each adverse event. Alternative causes such as natural history of the underlying diseases, concomitant therapy, other risk factors and the temporal relationship of the event to the investigational product will be considered and investigated.

The investigator will make an assessment of the causality of the AE by answering "Yes" or "No" to the question "Is there a reasonable possibility of a causal relationship between the study drug and the AE?" in the CRF.

14.3.6. *Study endpoints and symptoms anaemia*

Adverse events judged to be symptoms of anaemia or meeting the definition of any of the study outcome (severe anaemia) will not be recorded as adverse events unless they fulfil the criteria for an SAE or result in discontinuation of study treatment.

14.3.7. *HIV-related disease*

In HIV infected subjects, adverse events judged to be expected because of HIV-related disease will not be recorded as adverse events unless they fulfil the criteria for a SAE or result in discontinuation of study treatment.

14.3.8. *Lack of efficacy and disease progression*

Any deterioration in the subject's condition after the subject has been enrolled in the study will be discussed with / assessed by the principal investigator. Where the deterioration is considered by the principal investigator to constitute a progression of anaemia or lack of effectiveness this will not be considered an AE unless it fulfils the criteria for an SAE or results in discontinuation of study treatment. Symptoms of unexpected disease progression (as assessed by the investigator) should be recorded as AEs.

14.3.9. *Abnormal laboratory values*

The duplicate reporting of laboratory tests as both laboratory findings and adverse events in the CRF will be avoided. Abnormal laboratory tests results will not be reported as AEs in the CRF unless they fulfil the criteria for a SAE or result in discontinuation of study treatment. They will be evaluated in the overall safety analysis**.** If an abnormal laboratory value is associated with clinical signs and

symptoms, the sign/symptom should be reported as an AE while the associated test result is recorded in the appropriate CRF section.

14.3.10. *Overdose*

Use of study medication in doses in excess of that specified under the section on Trial Interventions should be recorded as an AE. An overdose without associated symptoms should be recorded as an AE of "Overdose".

14.4. Reporting of serious AEs

14.4.1. *Reporting by the investigator to the study safety monitor and sponsor*

The Site Principal Investigator/delegate is responsible for recording all safety events in the source document.

The investigator must report all SAEs that occur during the course of the study to the Sponsor and the ethics committee as soon as possible but within 24 hours of the investigational site becoming aware of it – in accordance with the *'Safety monitoring and reporting in clinical trials involving therapeutic goods' (November 2016)*[90]. All SAEs have to be reported, whether or not considered causally related to the investigational product or study procedure(s). All SAEs will be reported on the SAE form provided. The reporting will be made by research study clinicians.

Follow-up information on SAEs must also be reported by the investigator within the same timeframe. In case of doubt about whether an event fulfils serious criteria, the case should be reported to the safety monitor who will assess whether the event should be reported as an SAE.

At Zomba Central Hospital (ZCH), there will be a Co-investigator assigned to be the Safety Monitor. In Zomba, it will be Dr Kabeya Biselele, a consultant Obstetrician with years of experiencing working in Malawi and other African countries managing pregnant women. He has also been a co-investigator and study physician on a large Pfizer-funded trial investigating the use of azithromycin-chloroquine in the prevention of malaria in pregnancy. The safety monitor shall be available for consultation in an emergency but shall also oversee and advise on the clinical care SOPs and guidelines within the trial. He will also be part of the Trial Steering Committee and hence provide any clinic care-related advice to the trial executive decision-making body.

14.4.2. *Reporting by the sponsor*

The sponsor is responsible for reporting SAEs and other safety issues to the Data Monitoring Committee (DMC) and the Ethics committees in an expedited manner in accordance with the EU guidelines. The sponsor will also inform all other investigators concerned of relevant information about SAEs that could adversely affect the safety of subjects.

14.5. Data monitoring committee

An independent Data Monitoring Committee (DMC) has been set up to review on a regular basis, safety and efficacy data of the ongoing trial. The sponsor will report all SAEs and other relevant safety information to the DMC on an expedited basis. The DMC will review tables of cumulative SAEs, primary

and secondary endpoints at regular intervals. The DMC will remain blinded when presented with any interim analysis results unless the DMC judges that for safety reason the study blind should be broken.

The DMC will comprise of international experts in clinical trials, obstetrics, epidemiology and statistics.

15. DATA HANDLING AND RECORD KEEPING

Data collected from the subjects will be recorded in digital form with REDCap using electronic tablets. REDCap is hosted on infrastructure belonging to the trial's organisational team in Malawi and is subject to the same security and backup regimen as other systems (e.g. the network file servers). Data is backed up nightly to a local backup server, with a de-identified fortnightly backup taken to the servers of the team at the Walter and Eliza Hall Institute, Melbourne, Australia. Monthly backups are also done in physical format and the trial's Data Manager will maintain an audit trail of data create/update/delete events that is accessible to project users who are granted permission to view it. Access to REDCap will be provided via a certified user account or (for external collaborators) via a REDCap user account created by the trial's data manager. The permissions granted to each user within each REDCap project will be controlled by the trial team delegated this task by the Principal Investigator. REDCap has functionality that makes adding and removing users and managing user permissions straightforward. All data transmissions between users and the REDCap server are encrypted. The instructions for data entry to REDCap must be read and the training log signed prior to personnel commencing data entry on REDCap.

15.1. CASE REPORT FORMS (CRFS)

All CRFs (appendix 1) will be developed by the Investigators and implemented in REDCap by the data team. An Instruction manual on the completion of the CRF will be provided to all relevant study site personnel. It will be the responsibility of the investigators to ensure that the data in the CRFs are accurate and complete. If any data are not available, omissions will be indicated on the CRFs. The CRF will automatically sign off the user and lock the record upon completion. Relevant hard copy patient hospital files will be scanned for reference and stored digitally, securely. All CRFs will be checked by the investigator or authorised personnel for accuracy and completeness before uploading to the server. All corrections will be done by the investigator or authorised personnel; revisions will be automatically saved with tracked changes. The data will then be uploaded on the server.

15.2. DATA ENTRY AND VALIDATION

Data will be entered into REDCap database as they become available and exported weekly to the server. Hard copy data will be entered as soon as possible, and no later than 5 days, after collection. Laboratory data will be entered into the database in real time. Data query sheets will be raised and distributed by the data manager to the study team for resolution in a timely manner. Query resolutions will be stored together with the original CRFs.

15.3. Database lock

We will document the data as being clean only once the validation process is concluded. The treatment code will be broken and included in the database – with a subsequent database lock – only after all decisions on the evaluability of the data from each individual subject have been made and documented.

16. Statistical considerations

16.1. Sample size calculations

We plan to recruit 295 women per arm, or 590 women in total when accounting for 10% drop-out by week 36 (primary outcome timepoint).

For our primary outcome of maternal anaemia at 36 weeks gestation, we will be able to detect a reduction in the proportion of women with anaemia (Hb<11g/dL) at 36 weeks gestation from 63% in the oral iron arm to 49% in the IV iron arm with 90% power (two-sided 5%). We assumed that the mean Hb in oral iron treated women is 10.5g/dL and the standard deviation (SD) is 1.5g/dL, thus 63% of women are expected to be anaemic (Hb<11g/dL) at 36 weeks after oral iron. We assumed that the prevalence of anaemia in IV iron treated women would be 14% lower than that of women in the oral iron arm following the pivotal FCM vs oral iron trial. The Fer-ASAP trial demonstrated a 14% reduction in absolute anaemia prevalence compared with oral iron[25].

For our key secondary outcome of birthweight, after accounting for a miscarriage and stillbirth rate of 1%, we will be able to detect a birthweight increase of 100g to 150g in the IV iron arm compared to the oral iron arm with 72% to 97% power (two-sided 5% alpha). A birth weight increase of 100g is conservatively based on achieving an improvement two thirds as large as the 150g improvement seen in women randomized to oral iron versus control with high adherence in a recent Kenyan trial of oral antenatal iron which achieved 100% adherence (and noting that the control arm in our trial still involves giving iron, potentially ameliorating some of the effect). We assume a SD of 450g.

Data analysis

A detailed study statistical analysis plan for the final analysis will be drawn up during the course of the study and finalised before the unblinding of data.

16.1.1. *Assessment of effectiveness*

Descriptive statistics will be presented for all outcomes, by treatment group across the follow-up time points. Anaemia will be analysed using a log-binomial regression model with study participants included as a random intercept. The model will include the standard of care (oral iron) group as the reference group. The primary maternal hypothesis will be evaluated by obtaining the estimate of the risk ratio of IV iron versus standard of care (oral iron) and two-sided 95% confidence interval extracted at 36 weeks' gestation. Birthweight will be analysed by fitting a linear regression model. The primary neonatal hypothesis will be evaluated by estimating the absolute difference in birthweight between IV iron and standard care (oral iron) and two-sided 95% confidence interval. Secondary repeated time

point binary outcomes will be analysed similar to anaemia and secondary single time point continuous outcomes will be analysed similar to birthweight. Secondary, single time point binary outcomes (e.g., low birth weight) will be analysed using a log-binomial regression model and secondary, multiple time point continuous outcomes (e.g., haemoglobin) will be analysed using a likelihood-based longitudinal data analysis model[91]. Appropriate transformations may be applied to the variables before fitting the model if considered skewed (e.g. ferritin). In case of non-convergence of the log-binomial models, a Poisson model with robust standard errors will be fitted instead. Exploratory subgroup analyses (e.g., site, parity, iron deficiency) will be performed for maternal and neonatal outcomes, irrespective of their findings. The analyses models for all study outcomes will adjust for the randomisation stratification variables of site as a main effect.

16.1.2. *ANALYSIS OF ADVERSE EVENTS*

Safety including adverse events, infections and clinic visits will be presented for the mothers and neonates/infants respectively. The proportion of study participants with at least one safety outcome will be compared between arms using a log-binomial regression model. In case of non-convergence, a Poisson model with robust standard errors will be fitted instead.

16.2. ANALYSIS POPULATIONS

Analyses will be undertaken on an intention-to-treat basis.

16.3. MISSING DATA

Every effort will be made to minimise the amount of missing data in the trial. Whenever possible, information on the reason for missing data will be obtained. Additional analyses using multiple imputation will be performed to handle missing data in the primary maternal and neonate outcome. Results will be compared with the main analysis specified in Section 16.1.1 to investigate the robustness of the findings to assumptions about the missing data.

16.4. INTERIM ANALYSES AND CRITERIA FOR TERMINATION OF THE TRIAL

No formal interim analysis is planned. However, the sponsor reserves the right to temporarily suspend or prematurely discontinue this study at any time for reasons including, but not limited to, safety or ethical issues or severe non-compliance. If the sponsor determines such action is needed, it will discuss this with the investigator. When feasible, the sponsor will provide advance notification to the investigator of the impending action prior to it taking effect. The sponsor will promptly inform all the Ethics committee and regulatory bodies and provide the reason for the suspension or termination.

17. STUDY MANAGEMENT

17.1. STUDY MONITORING

A local trial monitor such as the Research Support Centre at the College of Medicine will be appointed to perform independent monitoring of the study on behalf of the sponsor. Prior to subject enrolment,

the monitor will visit the study site to determine the adequacy of facilities, review the protocol and data collection procedures and discuss the responsibilities of the investigator and other study site personnel.

During the study, the monitor will have regular site contacts, including conducting on-site visits to:

- Confirm that the study is being performed according to the protocol, ICH GCP and applicable regulations, data are being accurately recorded in the CRFs, samples are being appropriately collected and stored, and that investigational product accountability is being performed.
- Conduct source data verification
- Confirm facilities remain acceptable
- Provide information and support to the investigators
- Evaluate study progress

Upon completion of the study the monitor will visit the study sites to verify that all CRFs are completed and collected, all data queries have been resolved and filed, conduct final accountability, reconciliation and arrangements for investigational product and verify all study site records are complete. The PI and relevant staff will be available at monitoring visits and agree to allocate sufficient time to the monitor to discuss any issues and address their resolution.

17.2. Direct access to source data/documents

The investigator agrees to allow the sponsor and/or its representatives, including the monitor, the DMC, the IRB/IEC, the regulatory body direct access to source data and other relevant documents.

17.3. Quality assurance

Authorised representatives of the sponsor, an IEC/IRB or regulatory authority may visit the study site to perform audits or inspections, including source data verification.

17.4. Training of staff

The PI is responsible for the conduct of the study at this Study Sites, including delegation of specified study responsibilities, and training of study staff. The PI will maintain a record of all individuals involved in the study (medical, nursing and other staff). The PI will ensure that all persons assisting with the trial receive the appropriate training about the protocol, the investigational product(s) and their trial-related duties and functions. All relevant study nurses and clinical personnel handling the intravenous formulation infusions will receive specific and appropriate training before the start of the trial. During the study regular spot checks will be conducted to assess the performance of study site staff members and re-training provided where necessary.

17.5. Changes to the protocol

No change will be made to the approved protocol without the agreement of the sponsor. If it is necessary for the protocol to be amended, the protocol amendment will be submitted to the IRB/IEC for approval before implementation. Any change to the informed consent form must also be approved

by the sponsor and IRB/IEC, before the revised form is used. The sponsor will distribute amendments to the PI, who in turn is responsible for the distribution of these documents to the staff at his/her study site.

17.6. Financing and insurance

Funding for this study will be provided by Bill & Melinda Gates Foundation through a grant to the Walter and Eliza Hall Institute of Medical Research, Melbourne, Australia. The Training and Research Unit of Excellence (TRUE) will act as sponsor and will purchase a liability insurance policy that covers this study.

17.7. Study duration

The study will start as soon as all pre-study activities and documentation are completed, and the IRB/IEC and Regulatory Authority have approved the protocol. The total duration of the trial is 30 months, which includes 12 months of recruitment, 6 month of additional follow-up phase as per Gantt chart below:

Activity	2020				2021				2022				2023				2024	
	Q1	Q2	Q3	Q4	Q1	Q2	Q3	Q4	Q1	Q2	Q3	Q4	Q1	Q2	Q3	Q4	Q1	Q2
REVAMP-TT (Main Trial)																		
Protocol, Ethics - Malawi/ Melbourne, PMPB, Import drugs																		
Open trial																		
Recruit (aim to complete within 12 months)																		
Babies born (~3 months after recruitment)																		
Babies reach 12 months of age (last outcome measured)																		
Sample analysis																		
Un-blinding and data analysis of RCT																		
Report/ manuscript of RCT																		

17.8. Record-keeping and archiving

During the study, an Investigator Site File will be used to store documentation pertaining to the study and it will be kept in a secure location with access only to authorised individuals. It is the PI's responsibility to continuously update the Investigator file. It must be available to the Monitor during monitoring visits.

Following study closure, the investigator study file, CRFs, medical records and other source documents must be retained at the study site per regulatory obligations (two years after the last marketing application of the study drug in the European Union) and thereafter destroyed only after agreement with the sponsor. Documents with a limited shelf life (e.g. printouts on light/heat sensitive paper) will be copied and verified by signing and dating.

17.9. Reporting and publication of data

The results of the study will be submitted and discussed with the local and national medical authorities. They will then be presented at national and international conferences and submitted for publication in peer-reviewed journals, in accordance with the sponsor' publication policy.

18. Ethical considerations

18.1. Ethical review

This study will be submitted to the following institutional review boards and independent ethics committees (IRB/IEC).

- National Health Science Research Committee (NHSRC), Malawi
- Walter and Eliza Hall Institute of Medical Research (WEHI), Melbourne, Australia

Before initiating the trial, written and dated approval/favourable opinion must be obtained from the IRB/IECs. Protocol amendments will be submitted to the IRB/IEC for approval before implementation. Progress reports, SUSAR reports and safety reports will be submitted to the IRB/IEC in accordance with local requirements.

18.2. Ethical conduct of the study

This study will be conducted in accordance with The International Conference on Harmonisation of Technical Requirements for Registration of Pharmaceuticals for Human Use (ICH) (ICH) guidelines for "good clinical practice" (GCP) and all applicable regulatory requirements, including, where applicable, the 1996 version of the Declaration of Helsinki.

18.3. Informed consent

Informed consent will be obtained from each participant before conducting any study related procedure. The PI will ensure that the participant (and the subject's parent/guardian in case of assent) is given full and adequate oral and written information about the study. Information will be provided in the local language (Chichewa) of the participant (appendix 2). The participant will be given the opportunity to ask questions and allowed time to consider the information provided.

Informed consent will be documented by the use of a written consent form signed by the participant and the person who conducted the informed consent discussion. If the participant is unable to write their signature, then a thumbprint may be used. If the participant is unable to read the information her/himself, full and comprehensive information will be communicated to the participant in the presence of a witness. The witness will be an independent third party i.e. a person not connected with the conduct of the trial. The witness will sign the informed consent form to attest that the information in the consent form was accurately explained to and apparently understood by the participant and that informed consent was freely given. Each original signed informed consent will be kept on file by the investigator. A copy of the informed consent form will be provided to the participant.

Written informed consent obtained at the baseline visit will encompass all study procedures and visits up to and including the 12-month post-partum visit. We will re-discuss with mothers the extension study (mother and infant visits at 3, 6, 9 and 12-months post-partum) during the 1-month postpartum visit, although consent will have already been collected.

We will ask for informed consent for future analysis of samples for nutritional, immune and inflammatory biomarkers, molecular biomarkers, and for non-diagnostic genetic analyses that

provides insights into associations with response to the interventions, and into aspects of placental and child growth, metabolic and immune development and health. Participants prepared to enrol in the trial but who do not wish to provide consent for these extended uses of the samples will be able to decline consent for this aspect of the study.

18.4. Risks to the study participants

All efforts will be made to minimise pain, discomfort, and fear for both pregnant women and the children.

18.4.1. *Blood sampling*

The amount of blood taken for the study is minimal, totalling a maximum of 10 ml per visit over the study period at two timepoints during pregnancy and five timepoints postpartum (an additional 5 ml may be taken during sick visits for clinically-relevant laboratory analyses) for mothers, and <3mL per visit at four time points for infants. Blood will be collected in most cases through a venepuncture and where indicated, a finger prick. A small bruise or mild pain on the finger or the venepuncture site from where the blood is taken may develop. Only well-trained and fully qualified laboratory staff will be hired for this project. Only new disposable needles and lancets are used for the blood taking procedures, and these will be discarded immediately after their use.

18.4.2. *Intravenous infusions*

Participants allocated to the intravenous iron intervention arm~~s~~ will receive an intravenous infusion. A cannula will be inserted by venepuncture to allow the infusion of FCM over a period of 15 minutes. A small bruise or mild pain on the venepuncture site may develop. As discussed above, anaphylaxis is not a major risk for FCM, however there will always be an emergency resuscitation tray available during this procedure. A fully qualified health worker, with specific training in administering and managing intravenous iron infusions, will be available during the entire infusion process (and for a further 45 min after) to monitor the condition of the participant. The cannula will not be removed until the trial participant is certified as being well, with normal blood pressure and heart rate prior to discharge home.

18.5. Benefits from participating in the trial

Participants enrolled in the study will receive close monitoring of their condition for the study period. A trained clinician will see subjects at recruitment and scheduled follow-up visits, for full medical assessments. If a subject has an illness in-between scheduled visits, the subject will be seen by the study clinician and treated at the study clinic if the illness is deemed to be study related or referred to ZCH if other care is required.

18.6. Subject data protection

Participant confidentiality is strictly held in trust by the participating investigators, research staff, and the sponsoring institution and their agents. This confidentiality is extended to cover testing of biological samples and genetic tests in addition to the clinical information relating to participating

participants. The study protocol, documentation, data and all other information generated will be held in strict confidence. No information concerning the study, or the data, will be released to any unauthorised third party, without prior written approval of the sponsoring institution. Authorised representatives of the sponsoring institution may inspect all documents and records required to be maintained by the Investigator, including but not limited to, medical records (office, clinic or hospital) and pharmacy records for the participants in this study. The clinical study site will permit access to such records. All laboratory specimens, evaluation forms, reports and other records that leave the site will be identified only by the Participant's Unique Identification Number (UID) to maintain participant confidentiality. Clinical information will not be released without written permission of the participant, except as necessary for monitoring by HREC, IRB or regulatory agencies.

18.7. Other ethical considerations

18.7.1. *Reimbursement of costs*

The study will provide payment for all the study drugs, study procedures, study-related visits and all reasonable medical expenses that may be incurred as a direct result of the study. This includes compensation for transport for each study visit and will be according to the local ethics committee guidelines. Where applicable the participant will be provided with or reimbursed for, refreshments when a scheduled appointment requires them to be out of their home at mealtimes. In Malawi, in-patient hospital care is free of charge.

19. Dissemination of results

At the end of the study, the results will first be disseminated to national policymakers, government departments, academics from local research institutions and universities, NHSRC, COMREC and professional bodies in Malawi at the national stakeholders' meeting or research dissemination conferences to be held in the country. Subject to the findings of the study and based on consensus emerging at these meetings, we will support national policymakers to develop the necessary tools and guidelines to guide national and district level health providers to implement the strategy within hospital services and the health system more broadly.

Research results will also be disseminated to the global research community, technical agencies, and international government bodies via peer-reviewed journals and at international scientific fora. We will also inform other international organisations and funders of large-scale anaemia control initiatives which aim to improve anaemia at regional and local levels and are instrumental in supporting countries to implement anaemia control policies in low and middle-income countries.

20. Capacity building

Research capacity in Malawi will be enhanced by the provision of training and mentorship for all the research staff involved in the trial. By running this study, capacity in trial management will be enhanced, as will the capacity to deliver intravenous iron infusions at several health centres. The research study will strengthen the clinical skills of health workers in managing pregnant women with

anaemia. There will be 1-2 PhD candidates who will conduct their research as part of this project. Partners from the different institutions forming this research network will jointly supervise them.

In addition, there will be a post-doctoral research scientist who will work closely with the study PIs in the conduct of the study. In the process, the post-doctoral research scientist will be able to carry out various research projects from this study that will be published in peer-reviewed journals. Lastly, all study staff will undertake Good Clinical Practice (GCP) training before starting to work on the research project.

21. Budget

REVAMP TT Budget in US$

PERSONNEL	No.	Cost/month	Cost/year	Year 1	Year 2	Year 3	Total (USD)
Site Coordinator	1	1,200	14,400	7,200	15,120	15,840	38,160
Data Officer	1	836	10,032	5,016	10,534	11,035	26,585
Clinical Officer	1	969	11,628	5,814	12,209	12,791	30,814
Research Nurse	3	850	10,200	15,300	32,130	33,660	81,090
Research Assistants	1	609	7,308	3,654	7,673	8,039	19,366
Lab Technician	1	600	7,200	7,200	7,560	7,920	22,680
**Total - Personnel**				**44,184**	**85,226**	**89,285**	**218,695**

EQUIPMENT	No.	Cost/item					
Desk-top computer	1	1,200		1,200			1,200
Laptops	1	1,542		1,542			1,542
Printer & accessories	1	1,100		1,100			1,100
Hemocue machine	3	300		900			900
Small clinical equipment	1	2,000		2,000		2,000	4,000
**Total - Equipment**				**6,742**	**-**	**2,000**	**8,742**

CONSUMABLES							
Lab consumables				375	1,250	875	2,500
RDT cuvettes				2,000	3,000	1,000	6,000
Fuel - bikes & Car hire				2,340	3,120	3,120	8,580
Maintenance & insurance				700	770	840	2,310

Patient reimbursement	3,120	6,240	6,240	15,600
Consumables - Office				
Communications & internet	1,200	1,260	1,323	3,783
Stationary	1,200	1,320	1,584	4,104
**TOTAL - Consumables**	**10,935**	**16,960**	**14,982**	**42,877**
**Trial Management**				
Monitoring	2,000	2,000	2,000	6,000
Trial Insurance	12,000			12,000
Ethics & regulatory fee	6,150	1,000	1,000	8,150
**TOTAL - Trial Management**	**20,150**	**3,000**	**3,000**	**26,150**
**Overheads (10%)**	**8,201**	**10,519**	**10,927**	**29,646**
**GRAND TOTAL ($)**	**90,212**	**115,705**	**120,193**	**326,111**

22. Budget justification

We requested a total of $326,111 to conduct the study as described above. The majority of the funds ($218,695) will go towards staff renumeration. The staff will be involved in running daily study activities. A total of $8,742 will be directed towards procurement of office equipment which includes: desktop and laptop for data collection and archiving, printers for printing study related materials including CRFs, SOPs and procurement of other clinical equipments such as HemoCue machine for Hb measurements.

A total of $42,877 will be used for the procurement of consumables. This will include lab supplies such as Hb cuvettes, specimen bottles, reagents that will be required for sample processing and analysis. Participants were also given money for transport reimbursement and food as required by the ethics guidelines in all study visits. The study site will also need airtime as well as internet for effective communication locally as well as with international Principal Investigators.

We will need $26,150 for trial management. This money will be used to pay study monitors who undertake important tasks of ensuring that study is implemented in accordance with the declaration of Helsinki thereby ensuring participants'safety. The trail will have two insurances for participants as well as medical malpractice insurance. Payments will also be made to NHSRC for reviewing the protocol and Pharmacy Medicine and Poison Board annually for monitoring the study. Finally, project overheads will be used for study administrative support.

23. BIBLIOGRAPHY

1 Mwangi MN, Roth JM, Smit MR, *et al.* Effect of daily antenatal iron supplementation on plasmodium infection in kenyan women: A randomized clinical trial. *JAMA - J Am Med Assoc* 2015; **314**: 1009–20.

2 Radhika AG, Sharma AK, Perumal V, *et al.* Parenteral Versus Oral Iron for Treatment of Iron Deficiency Anaemia During Pregnancy and post-partum: A Systematic Review. *J Obstet Gynecol India* 2019; **69**: 13–24.

3 Qassim A, Grivell RM, Henry A, Kidson-Gerber G, Shand A, Grzeskowiak LE. Intravenous or oral iron for treating iron deficiency anaemia during pregnancy: systematic review and meta-analysis. *Med J Aust* 2019; **211**: 367–73.

4 Lewkowitz AK, Gupta A, Simon L, *et al.* Intravenous compared with oral iron for the treatment of iron-deficiency anemia in pregnancy: a systematic review and meta-analysis. *J Perinatol* 2019; **39**: 519–32.

5 Pollock RF, Muduma G. A systematic literature review and indirect comparison of iron isomaltoside and ferric carboxymaltose in iron deficiency anemia after failure or intolerance of oral iron treatment. *Expert Rev Hematol* 2019; **12**: 129–36.

6 Wolf M, Rubin J, Achebe M, *et al.* Effects of Iron Isomaltoside vs Ferric Carboxymaltose on Hypophosphatemia in Iron-Deficiency Anemia: Two Randomized Clinical Trials. *JAMA - J Am Med Assoc* 2020; **323**: 432–43.

7 Schaefer B, Würtinger P, Finkenstedt A, *et al.* Choice of high-dose intravenous iron preparation determines hypophosphatemia risk. *PLoS One* 2016; **11**: 1–11.

8 Detlie TE, Lindstrøm JC, Jahnsen ME, *et al.* Incidence of hypophosphatemia in patients with inflammatory bowel disease treated with ferric carboxymaltose or iron isomaltoside. *Aliment Pharmacol Ther* 2019; **50**: 397–406.

9 WHO 2011. The global prevalence of anaemia in 2011. *Who* 2011; : 1–48.

10 Haider BA, Olofin I, Wang M, Spiegelman D, Ezzati M, Fawzi WW. Anaemia, prenatal iron use, and risk of adverse pregnancy outcomes: Systematic review and meta-analysis. *BMJ* 2013; **347**: 1–19.

11 Nair M, Knight M, Kurinczuk J. Risk factors and newborn outcomes associated with maternal deaths in the UK from 2009 to 2013: a national case-control study. *BJOG An Int J Obstet Gynaecol* 2016; **123**: 1654–62.

12 Drassinower D, Lavery JA, Friedman AM, Levin HI, Običan SG, Ananth C V. The effect of maternal haematocrit on offspring iq at 4 and 7 years of age: A secondary analysis. *BJOG An Int J Obstet Gynaecol* 2016; **123**: 2087–93.

13 WHO. Global nutrition targets 2025: Anaemia Policy Brief. 2012; : 1–7.

14 WHO: World Health Organization. Low Birth Weight Policy Brief. *Low Birth Weight Policy Br* 2014; : 1.

15 Beck S, Wojdyla D, Say L, *et al.* The worldwide incidence of preterm birth: a systematic review of maternal mortality and morbidity. *Bull World Health Organ* 2010; **88**: 31–8.

16 Pena-Rosas JP, De-Regil LM, Garcia-Casal MN, Dowswell T. Daily oral iron supplementation

during pregnancy. *Cochrane database Syst Rev* 2015; : CD004736.

17 WHO. Iron and Folate Supplementation: Intergrated Management of Pregnancy and Childbirth (IMPAC). Geneva, 2006 www.who.int/making_pregnancy_safer/publications/en.

18 WHO. Essential Nutrition Actions: improving maternal, newborn, infant and young child health and nutrition. Geneva, 2013.

19 Low MSY, Speedy J, Styles CE, De-Regil LM, Pasricha S-R. Daily iron supplementation for improving anaemia, iron status and health in menstruating women. *Cochrane database Syst Rev* 2016; **4**: CD009747.

20 Bah A, Pasricha S-R, Jallow MW, *et al.* Serum Hepcidin Concentrations Decline during Pregnancy and May Identify Iron Deficiency: Analysis of a Longitudinal Pregnancy Cohort in The Gambia. *J Nutr* 2017; **147**: 1131–7.

21 Rognoni C, Venturini S, Meregaglia M, Marmifero M, Tarricone R. Efficacy and Safety of Ferric Carboxymaltose and Other Formulations in Iron-Deficient Patients: A Systematic Review and Network Meta-analysis of Randomised Controlled Trials. *Clin Drug Investig* 2016; **36**: 177–94.

22 Keating GM. Ferric carboxymaltose: a review of its use in iron deficiency. *Drugs* 2015; **75**: 101–27.

23 Friedrisch JR, Cançado RD. Intravenous ferric carboxymaltose for the treatment of iron deficiency anemia. *Rev Bras Hematol Hemoter* 2015; **37**: 400–5.

24 Moore RA, Gaskell H, Rose P, Allan J. Meta-analysis of efficacy and safety of intravenous ferric carboxymaltose (Ferinject) from clinical trial reports and published trial data. *BMC Blood Disord* 2011; **11**: 4.

25 Breymann C, Milman N, Mezzacasa A, Bernard R, Dudenhausen J. Ferric carboxymaltose vs. oral iron in the treatment of pregnant women with iron deficiency anemia: An international, open-label, randomized controlled trial (FER-ASAP). *J Perinat Med* 2017; **45**: 443–53.

26 Van Wyck DB, Martens MG, Seid MH, Baker JB, Mangione A. Intravenous Ferric Carboxymaltose Compared With Oral Iron in the Treatment of Postpartum Anemia. *Obstet Gynecol* 2007; **110**: 267–78.

27 Kulnigg S, Stoinov S, Simanenkov V, *et al.* A novel intravenous iron formulation for treatment of anemia in inflammatory bowel disease: The ferric carboxymaltose (FERINJECT®) randomized controlled trial. *Am J Gastroenterol* 2008; **103**: 1182–92.

28 Evstatiev R, Marteau P, Iqbal T, *et al.* FERGIcor, a randomized controlled trial on ferric carboxymaltose for iron deficiency anemia in inflammatory bowel disease. *Gastroenterology* 2011; **141**: 846-853.e2.

29 Beigel F, Löhr B, Laubender RP, *et al.* Iron status and analysis of efficacy and safety of ferric carboxymaltose treatment in patients with inflammatory bowel disease. *Digestion* 2012; **85**: 47–54.

30 Befrits R, Wikman O, Blomquist L, *et al.* Anemia and iron deficiency in inflammatory bowel disease: An open, prospective, observational study on diagnosis, treatment with ferric carboxymaltose and quality of life. *Scand J Gastroenterol* 2013; **48**: 1027–32.

31 Evstatiev R, Alexeeva O, Bokemeyer B, *et al.* Ferric carboxymaltose prevents recurrence of anemia in patients with inflammatory bowel disease. *Clin Gastroenterol Hepatol* 2013; **11**: 269–77.

32 Laass MW, Straub S, Chainey S, Virgin G, Cushway T. Effectiveness and safety of ferric carboxymaltose treatment in children and adolescents with inflammatory bowel disease and other gastrointestinal diseases. *BMC Gastroenterol* 2014; **14**: 1–8.

33 Cortes X, Borrás-Blasco J, Molés JR, Boscá M, Cortés E. Safety of ferric carboxymaltose immediately after infliximab administration, in a single session, in inflammatory bowel disease patients with iron deficiency: A pilot study. *PLoS One* 2015; **10**: 1–11.

34 Szczech LA, Bregman DB, Harrington RA, *et al.* Randomized Evaluation of efficacy and safety of ferric carboxymaltose in Patients with iron deficiency Anaemia and Impaired Renal function (REPAIR-IDA): Rationale and study design. *Nephrol Dial Transplant* 2010; **25**: 2368–75.

35 Ponikowski P, Filippatos G, Colet JC, *et al.* The impact of intravenous ferric carboxymaltose on renal function: An analysis of the FAIR-HF study. *Eur J Heart Fail* 2015; **17**: 329–39.

36 Anker SD, Colet JC, Filippatos G, *et al.* Ferric carboxymaltose in patients with heart failure and iron deficiency. *N Engl J Med* 2009; **361**: 2436–48.

37 Bisbe E, Garca-Erce JA, Díez-Lobo AI, Muñoz M. A multicentre comparative study on the efficacy of intravenous ferric carboxymaltose and iron sucrose for correcting preoperative anaemia in patients undergoing major elective surgery. *Br J Anaesth* 2011; **107**: 477–8.

38 Bernabeu-Wittel M, Aparicio R, Romero M, *et al.* Ferric carboxymaltose with or without erythropoietin for the prevention of red-cell transfusions in the perioperative period of osteoporotic hip fractures: A randomized contolled trial. the PAHFRAC-01 project. *BMC Musculoskelet Disord* 2012; **13**: 27.

39 Borstlap WAA, Buskens CJ, Tytgat KMAJ, *et al.* Multicentre randomized controlled trial comparing ferric(III)carboxymaltose infusion with oral iron supplementation in the treatment of preoperative anaemia in colorectal cancer patients. *BMC Surg* 2015; **15**: 1–7.

40 Nores J. The Efficacy of IV Ferric Carboxymaltose in the Perioperative Management of Moderate to Severe Iron Deficiency Anemia. *J Minim Invasive Gynecol* 2015; **22**: S211–2.

41 Khalafallah AA, Yan C, Al-Badri R, *et al.* Intravenous ferric carboxymaltose versus standard care in the management of postoperative anaemia: a prospective, open-label, randomised controlled trial. *Lancet Haematol* 2016; **3**: e415–25.

42 Froessler B, Collingwood J, Hodyl NA, Dekker G. Intravenous ferric carboxymaltose for anaemia in pregnancy. *BMC Pregnancy Childbirth* 2014; **14**: 1–5.

43 Zeba D, Khanam PA, Ahamed M, Khair MA. Intravenous Iron Treatment in Pregnancy: Ferric Carboxymaltose for Correction of Iron Deficiency Anaemia. *Faridpur Med Coll J* 2017; **12**: 54–7.

44 Mishra V, Gandhi K, Roy R, Hokabaj S, Shah KN. Role of Intravenous Ferric Carboxy-maltose in Pregnant Women with Iron Deficiency Anaemia. *J Nepal Health Res Counc* 2017; **15**: 96–9.

45 Christoph P, Schuller C, Studer H, Irion O, De Tejada BM, Surbek D. Intravenous iron treatment in pregnancy: Comparison of high-dose ferric carboxymaltose vs. iron sucrose. *J Perinat Med* 2012; **40**: 469–74.

46 Pavord S, Daru J, Prasannan N, Robinson S, Stanworth S, Girling J. UK guidelines on the management of iron deficiency in pregnancy. *Br J Haematol* 2020; **188**: 819–30.

47 National Health Service. Ferinject in Pregnancy and the Postpartum Period. 2018. https://www.meht.nhs.uk/EasysiteWeb/getresource.axd?AssetID=14692&type=Full&servicetype=Attachment.

48 Achebe MM, Gafter-Gvili A. How I treat anemia in pregnancy: Iron, cobalamin, and folate. *Blood* 2017; **129**: 940–9.

49 Kalra PA. Introducing iron isomaltoside 1000 (Monofer®)development rationale and clinical experience. *NDT Plus* 2011; **4**: 10–3.

50 Food and Drug Administration. Product Quality Review - Monoferric. *Approv Lett* 2018; : 1–45.

51 Administration TG. AusPAR Attachment 2 Extract from the Clinical Evaluation Report for mirabegron Proprietary Product Name : Betmiga. 2013.

52 Monograph P, Patient I, Information M. Monoferric Product Monograph. *Pfizer Canada* 2018; : 1–37.

53 Jahn MR, Andreasen HB, Fütterer S, *et al.* A comparative study of the physicochemical properties of iron isomaltoside 1000 (Monofer®), a new intravenous iron preparation and its clinical implications. *Eur J Pharm Biopharm* 2011; **78**: 480–91.

54 Wikström B, Bhandari S, Barany P, *et al.* Iron isomaltoside 1000: a new intravenous iron for treating iron deficiency in chronic kidney disease. *J Nephrol* 2011; **24**: 589–96.

55 Tomer A, Amir B, Alon G, Hefziba G, Leonard L, Anat GG. The safety of intravenous iron preparations: Systematic review and meta-analysis. *Mayo Clin Proc* 2015; **90**: 12–23.

56 Food and Drug Administration. Ferric Carboxymaltose(Injectafer) - Highlights of prescribing information. 2013; : 1–11.

57 Calvet X, Ruíz MÀ, Dosal A, *et al.* Cost-Minimization Analysis Favours Intravenous Ferric Carboxymaltose over Ferric Sucrose for the Ambulatory Treatment of Severe Iron Deficiency. *PLoS One* 2012; **7**: 7–11.

58 Kalra PA, Bhandari S, Saxena S, *et al.* A randomized trial of iron isomaltoside 1000 versus oral iron in non-dialysis-dependent chronic kidney disease patients with anaemia. *Nephrol Dial Transplant* 2016; **31**: 646–55.

59 Bhandari S, Kalra PA, Kothari J, *et al.* A randomized, open-label trial of iron isomaltoside 1000 (Monofer®) compared with iron sucrose (Venofer®) as maintenance therapy in haemodialysis patients. *Nephrol Dial Transplant* 2015; **30**: 1577–89.

60 Reinisch W, Staun M, Tandon RK, *et al.* A randomized, open-label, non-inferiority study of intravenous iron isomaltoside 1,000 (monofer) compared with oral iron for treatment of anemia in ibd (proceed). *Am J Gastroenterol* 2013; **108**: 1877–88.

61 Reinisch W, Altorjay I, Zsigmond F, *et al.* A 1-year trial of repeated high-dose intravenous iron isomaltoside 1000 to maintain stable hemoglobin levels in inflammatory bowel disease. *Scand J Gastroenterol* 2015; **50**: 1226–33.

62 Dahlerup JF, Jacobsen BA, van der Woude J, Bark LÅ, Thomsen LL, Lindgren S. High-dose fast infusion of parenteral iron isomaltoside is efficacious in inflammatory bowel disease patients with iron-deficiency anaemia without profound changes in phosphate or fibroblast growth factor 23. *Scand J Gastroenterol* 2016; **51**: 1332–8.

63 Frigstad S, Rannem T, Hellstrom P, Hammarlund P, Bonderup O. A Scandinavian prospective observational study of iron isomaltoside 1000 treatment: clinical practice and outcomes in iron deficiency anaemia in patients with IBD [poster P481]. In: 10th Congress of the European Crohn's and Colitis Organisation (ECCO). 2014: 320.

64 Birgegård G, Henry D, Thomsen LAM. (MONOFER®), INTRAVENOUS IRON ISOMALTOSIDE 1000

ORAL, AS MONO THERAPY IN COMPARISON WITH NON-MYELOID, IRON SULPHATE IN PATIENTS WITH CHEMOTHERAPY, MALIGNANCIES ASSOCIATED WITH (CIA), INDUCED ANAEMIA. In: Supportive Care in Cancer. 2015: 1–388.

65 Johansson PI, Rasmussen AS, Thomsen LL. Intravenous iron isomaltoside 1000 (Monofer®) reduces postoperative anaemia in preoperatively non-anaemic patients undergoing elective or subacute coronary artery bypass graft, valve replacement or a combination thereof: A randomized double-blind placebo-. *Vox Sang* 2015; **109**: 257–66.

66 Hildebrandt PR, Bruun NE, Nielsen OW, *et al.* Effects of administration of iron isomaltoside 1000 in patients with chronic heart failure. A pilot study. *Transfus Altern Transfus Med* 2010; **11**: 131–7.

67 Holm C, Thomsen LL, Norgaard A, Langhoff-Roos J. Intravenous iron isomaltoside 1000 administered by high single-dose infusions or standard medical care for the treatment of fatigue in women after postpartum haemorrhage: Study protocol for a randomised controlled trial. *Trials* 2015; **16**: 1–8.

68 Frigstad SO, Haaber A, Bajor A, *et al.* The NIMO Scandinavian Study: A Prospective Observational Study of Iron Isomaltoside Treatment in Patients with Iron Deficiency. *Gastroenterol Res Pract* 2017; **2017**. DOI:10.1155/2017/4585164.

69 Bhandari S, Kalra PA, Berkowitz M, Belo D, Thomsen LL, Wolf M. Safety and efficacy of iron isomaltoside 1000/ferric derisomaltose versus iron sucrose in patients with chronic kidney disease: the FERWON-NEPHRO randomized, open-label, comparative trial. *Nephrol Dial Transplant* 2020; : 1–10.

70 Aksan A, Dignass A, Stein J. Letter: An Economic Evaluation of Iron Isomaltoside 1000 Versus Ferric Carboxymaltose in Patients with Inflammatory Bowel Disease and Iron Deficiency Anemia in Denmark. *Adv Ther* 2019; **36**: 1817–20.

71 European Commission. Communication from the Commission - Detailed guidance on the collection, verification and presentation of adverse event/reaction reports arising from clinical trials on medicinal products for human use (‘CT-3’) (2011/C 172/01). *Online* 2011; **C**: 1–19.

72 Knochel JP. The Pathophysiology and Clinical Characteristics of Severe Hypophosphatemia. *Arch Intern Med* 1977; **137**: 203–20.

73 Milman N. Postpartum anemia II: Prevention and treatment. *Ann Hematol* 2012; **91**: 143–54.

74 Milman N. Postpartum anemia I: Definition, prevalence, causes, and consequences. *Ann Hematol* 2011; **90**: 1247–53.

75 Azami M, Badfar G, Khalighi Z, *et al.* The association between anemia and postpartum depression: A systematic review and meta-analysis. *Casp J Intern Med* 2019; **10**: 115–24.

76 Khalafallah AA, Dennis AE. Iron deficiency anaemia in pregnancy and postpartum: Pathophysiology and effect of oral versus intravenous iron therapy. *J Pregnancy* 2012; **2012**. DOI:10.1155/2012/630519.

77 Wesström J. Safety of intravenous iron isomaltoside for iron deficiency and iron deficiency anemia in pregnancy. *Arch Gynecol Obstet* 2020; **301**: 1127–31.

78 Kimani J, Phiri K, Kamiza S, *et al.* Efficacy and Safety of Azithromycin-Chloroquine versus Sulfadoxine-Pyrimethamine for Intermittent Preventive Treatment of Plasmodium falciparum Malaria Infection in Pregnant Women in Africa: An Open-Label, Randomized Trial. *PLoS One* 2016; **11**: e0157045.

79 Government of Malawi, MOH, NMCP. Guidelines for the treatment of malaria in Malawi, 4th Edition, July 2013. 2013.

80 Rose SA, Feldman JF, Wallace IF, McCarton C. Information Processing at 1 Year: Relation to Birth Status and Developmental Outcome During the First 5 Years. *Dev Psychol* 1991; **27**: 723–37.

81 Bornstein MH, Sigman MD. Continuity in Mental Development from Infancy. *Child Dev* 1986; **57**: 251–74.

82 deRegnier R-A, Nelson CA, Thomas KM, Wewerka S, Georgieff MK. Neurophysiologic evaluation of auditory recognition memory in healthy newborn infants and infants of diabetic mothers. *J Pediatr* 2000; **137**: 777–84.

83 Nuwer MR, Comi G, Emerson R, *et al.* IFCN standards for digital recording of clinical EEG. *Electroencephalogr Clin Neurophysiol* 1998; **106**: 259–61.

84 Monif M, Seneviratne U. Clinical factors associated with the yield of routine outpatient scalp electroencephalograms: A retrospective analysis from a tertiary hospital. *J Clin Neurosci* 2017; **45**: 110–4.

85 Geng F, Mai X, Zhan J, *et al.* Impact of Fetal-Neonatal Iron Deficiency on Recognition Memory at 2 Months of Age. *J Pediatr* 2015; **167**: 1226–32.

86 Monga M, Walia V, Gandhi A, Chandra J, Sharma S. Effect of iron deficiency anemia on visual evoked potential of growing children. *Brain Dev* 2010; **32**: 213–6.

87 Burden MJ, Westerlund AJ, Armony-Sivan R, *et al.* An Event-Related Potential Study of Attention and Recognition Memory in Infants With Iron-Deficiency Anemia. *Pediatrics* 2007; **120**: e336–45.

88 Algarín C, Peirano P, Garrido M, Pizarro F, Lozoff B. Iron deficiency anemia in infancy: Long-lasting effects on auditory and visual system functioning. *Pediatr Res* 2003; **53**: 217–23.

89 Algarín C, Nelson CA, Peirano P, Westerlund A, Reyes S, Lozoff B. Iron-deficiency anemia in infancy and poorer cognitive inhibitory control at age 10 years. *Dev Med Child Neurol* 2013; **55**: 453–8.

90 Council NH and MR, National Health and Medical Research Council. Guidance: Safety monitoring and reporting in clinical trials involving therapeutic goods. 2016; : 27.

91 Zeger SL. Indian Statistical Institute Longitudinal Data Analysis of Continuous and Discrete Responses for Pre-Post Designs Author (s): Kung-Yee Liang and Scott L . Zeger Source : Sankhyā : The Indian Journal of Statistics , Series B (1960-2002), Vol . 62 , No . 2016; **62**: 134–48.

24. APPENDIX

24.1. Case Report Forms – CRFs

24.1.1. *PRE-SCREENING*

PARTICIPANTS INFORMATION

Participant's Name:

Date of Birth (according to health passport): **Age:**

		–				–				

Residential Area:

Gravidity: **Parity:**

Date of Last Normal Menstrual Period (LNMP) if the participant is able to recall

☐☐ / ☐☐☐/ ☐☐☐☐
dd mmm yyyy

24.1.2. SCREENING POST-MALARIA TREATMENT

Prescreening after malaria treatment

(This form should only be filled if potential participant had a positive malaria during the first pre-screening visit, was treated according to national standard treatment guidelines, and accepted to come back after a week for repeat screening)

Does the participant have any of the following?		
01	Clinical symptoms of malaria<br>(If participant has malaria, treat according to national guidelines and ask them to come back in one week for re-screening)<br><br>If yes	☐ (0) No ☐ (1) Yes<br><br>(1) Fever<br>(2) Chills<br>(3) Headache<br>(4) Myalgia (joints pain)<br>(5) Malaise
02	Positive malaria parasitaemia (Microscopy)	☐ (0) No ☐ (1) Yes<br>☐ (2) Not done
03	Capillary Hb <5g/dl or >9.9g/dl	☐ (0) No ☐ (1) Yes<br><br>HB ☐☐.☐ g/dl
If "Yes" to any of the above, the subject is NOT eligible for the study		

STAFF ID ☐☐☐☐

24.1.3. FOETAL BIOMETRY

Gestational age by USS (Weeks + days): ☐☐.☐

Fetal biometry

☐ Not done

Current gestational age (weeks + days)	Head circumference (HC) using the ellipse facility ☐☐☐.☐☐<br>Femur length (FL) ☐☐☐.☐☐<br>**Estimated Gestational Age** ☐☐.☐<br>**Estimated date of delivery** ☐☐ / ☐☐/ ☐☐☐☐<br>dd mm yyyy
Placental localisation	☐ (1) Fundal<br>☐ (2) High anterior<br>☐ (3) High posterior<br>☐ (4) High left lateral<br>☐ (5) High right lateral<br>☐ (6) Low anterior<br>☐ (7) Low posterior<br>☐ (8) Low left lateral<br>☐ (9) Low right lateral
Amniotic fluid volume (Amniotic fluid index)	☐ (1) Normal<br>☐ (2) Moderately increased<br>☐ (3) Polyhydramnios<br>☐ (4) Moderately reduced<br>☐ (5) Oligohydramnios<br>☐ (6) Anhydramnios
Are there any other significant findings?	☐ (1) Yes ☐ (0) No
Comment on significant findings:	

STAFF INITIALS ______________________________________

24.1.4. SCREENING FORM

Study site screening

Inclusion Criteria

01	Viable intrauterine singleton with no evident gross anomaly on Ultrasound Scan?	☐ (0) No ☐ (1) Yes
02	27-35 weeks gestation by Ultrasound Scan	☐ (0) No ☐ (1) Yes<br>Estimated Gestational Age: ☐☐.☐<br>Estimated date of delivery: ☐☐ / ☐☐/ ☐☐☐☐<br>dd mm yyyy
03	[In case malaria test was not done in Pre-screening, perform now]<br>Negative malaria parasitaemia (MRDT)	☐ (0) No<br>☐ (1) Yes<br>☐ (2) NA
If 'Yes' to all of the above, subject is eligible for the study.		
04	Does the participant have any reasons warranting exclusion according to investigator's assessment?	☐ (0) No ☐ (1) Yes<br>If yes state the reason for exclusion
If 'No' subject is eligible for the study		

STAFF ID

24.1.5. INFORMED CONSENT PROCESS

Gestational age by USS (Weeks + days): ☐☐.☐

Informed consent process

(Please ensure the Consent Form used is the most recent version approved by NHSRC)

01. The participant chose to be consented in: ☐ (1) English ☐ (2) Chichewa

02. Husband Present During Consenting, if participant is married?

☐ (0) No ☐ (1) Yes ☐ (2) N/A

If no, the participant should be allowed to discuss with the husband before consenting. Strongly suggest participant contacts key household decision maker.

03. Resides within study catchment area and will be available during the entire study period
☐ (0) No ☐ (1) Yes (If 'NO' subject is NOT eligible for the study)

04. Allows to be followed up at home
☐ (0) No ☐ (1) Yes
(If 'NO' subject is NOT eligible for the study)

05. Accepts study procedures

☐ (0) No ☐ (1) Yes
(If 'NO' subject is NOT eligible for the study)

06. Signed an informed consent form

☐ (0) No ☐ (1) Yes
(If 'NO' subject is NOT eligible for the study)

07. Assigned Study ID

(**Note:** Use assigned Study ID from now on)

STAFF INITIALS

24.1.6. Demographics

Gestational age by USS (Weeks + days): ☐☐.☐

Demography

In this section I am going to ask you information on your background. Please feel free to ask me where you do not understand for clarification.

01.	What is your tribe?	☐ *(1) Chewa*<br>☐ *(2) Yao*<br>☐ *(3) Tumbuka*<br>☐ *(4) Lomwe*<br>☐ *(5) Sena*<br>☐ *(6) Tonga*<br>☐ *(7) Ngonde*<br>☐ *(8) Other Specify_______________*
02.	Have you ever attended formal school?	☐ *(0) No (**if no skip to Q04**)*<br>☐ *(1) Yes*
03.	How far did you go with your education?	☐ *(0) None*<br>☐ *(1) Lower Primary (1-5)*<br>☐ *(2) Upper Primary (6-8)*<br>☐ *(4) Lower Secondary (1-2)*<br>☐ *(5) Upper Secondary (3-4)*<br>☐ *(6) Tertiary*
04.	Are you able to read?	☐ *(0) No*<br>☐ *(1) Yes*
05.	Are you able to write?	☐ *(0) No*<br>☐ *(1) Yes*
06.	What is your current marital status?	☐ *(1) Single*<br>☐ *(2) Married*<br>☐ *(3) Widowed*<br>☐ *(4) Divorced/Separated*<br>☐ *(5) Other Specify__________________*
07.	What religion do you practice?	☐ (0) None<br>☐ (1) Christian<br>☐ (2) Moslem<br>☐ (3) Other Specify_______________
08.	What is the main source of income of your household?	☐ (0) None<br>☐ (1) Subsistence farming<br>☐ (2) Large scale farming<br>☐ (3) Employed<br>☐ (4) Casual work for wages<br>☐ (5) Business<br>☐ (6)Other; specify

STAFF INITIALS

24.1.7. SELF-REPORTING QUESTIONNAIRE

Self Reporting Questionnaire (SRQ) – Modified English version

Now I am going to ask you some questions about the thoughts and feelings that you have experienced over the last 4 weeks. You should answer yes or no to each question. If you are not sure, give the answer that is closest to how you have been feeling. If you do not understand a question, please ask and I can give you an example of what the question means.

1	Do you often have headaches?	Yes	No
2	Is your appetite poor?	Yes	No
3	Do you sleep badly?	Yes	No
4	Do your hands shake?	Yes	No
5	Do you feel nervous tense or worried?	Yes	No
6	Are you easily frightened?	Yes	No
7	Is your digestion poor?	Yes	No
8	Do you have trouble thinking clearly?	Yes	No
9	Do you feel unhappy?	Yes	No
10	Do you cry more than usual?	Yes	No
11	Do you find it difficult to enjoy your daily activities?	Yes	No
12	Do you find it difficult to make decisions?	Yes	No
13	Is your daily work suffering?	Yes	No
14	Are you unable to play a useful part in life?	Yes	No
15	Have you lost interest in things?	Yes	No
16	Do you feel that you are a worthless person?	Yes	No
17	Has the thought of ending your life been on your mind?	Yes	No
18	Do you feel tired all the time?	Yes	No
19	Do you have uncomfortable feelings in your stomach?	Yes	No
20	Are you easily tired?	Yes	No

Self Reporting Questionnaire (SRQ) - Chichewa version

Tsopano ndikufunsani mafunso okhudzana ndi momwe mumamvera mumtima ndi maganizo omwe mwakhala nawo m'sabata zinayi zomwe zapitazi. Muyankhe "eya" kapena "ayi" ku funso lirilonse. Ngati mukukaikira, yankhani mofanizira ndi momwe mwakhala mukumvera. Ngati simukumvetsa funso, chonde funsani ndipo ndikupatsani chitsanzo chotanthauzira funsolo.

1	*M'masabata anayi apitawa,* kodi mumamva kupweteka mutu pafupipafupi?	Eya	Ayi
2	*M'masabata anayi apitawa,* kodi simumakhala ndi chilakolako cha chakudya?	Eya	Ayi
3	*M'masabata anayi apitawa,* kodi mumavutika kugona usiku?	Eya	Ayi
4	*M'masabata anayi apitawa,* kodi manja anu amanjenjemera?	Eya	Ayi
5	*M'masabata anayi apitawa,* kodi mumakhala ndi nkhawa, mantha kapena madandaulo?	Eya	Ayi
6	*M'masabata anayi apitawa,* kodi simumachedwa kututumutsidwa?	Eya	Ayi
7	*M'masabata anayi apitawa,* kodi mumadzimbidwadzimbidwa?	Eya	Ayi
8	*M'masabata anayi apitawa,* kodi mumakhala ndi vuto kuganiza bwinobwino?	Eya	Ayi
9	*M'masabata anayi apitawa,* kodi mumakhala osasangalala kapena osakondwa?	Eya	Ayi
10	*M'masabata anayi apitawa,* kodi mumaliralira pafupipafupi ndipo koposera muyeso?	Eya	Ayi
11	*M'masabata anayi apitawa,* kodi mumaona ngati ndi chinthu chokuvutani kusangalatsidwa ndi zinthu zimene mumapanga tsiku ndi tsiku?	Eya	Ayi
12	*M'masabata anayi apitawa,* kodi mumakhala ndi vuto kupanga maganizo kapena kumanga mfundo?	Eya	Ayi
13	*M'masabata anayi apitawa,* kodi ntchito zanu za tsiku ndi tsiku sizimayenda bwino?	Eya	Ayi
14	*M'masabata anayi apitawa,* kodi mumalephera kupanga zinthu za phindu kapena zofunikira m'moyo wanu?	Eya	Ayi
15	*M'masabata anayi apitawa,* kodi munasiya kukhala ndi chidwi mu zinthu zosiyanasiyana?	Eya	Ayi
16	*M'masabata anayi apitawa,* kodi mumaziona ngati ndinu munthu wopanda ntchito kapena wosafunikira?	Eya	Ayi
17	*M'masabata anayi apitawa,* kodi maganizo odzipha anayamba akubwereranipo?	Eya	Ayi
18	*M'masabata anayi apitawa,* kodi mumamva kapena kukhala otopatopa nthawi zonse?	Eya	Ayi
19	*M'masabata anayi apitawa,* kodi mumakhala ndi vuto losamva bwino m'mimba?	Eya	Ayi
20	*M'masabata anayi apitawa,* kodi simumachedwa kutopa?	Eya	Ayi

24.1.8. OBSTETRIC HISTORY

Gestational age by USS (Weeks + days): ☐☐.☐

Obstetric Assessment

In this section, I am going to ask information concerning your current pregnancy and any other previous pregnancies. Feel free to ask me any question as I am examining you if you feel so.

Gynaecological History		
01.	How many pregnancies have you had including this one (gravidity)? (**If first pregnancy skip to 14**)	☐☐
02.	How many of those pregnancies did you carry for more than 28 weeks (parity)? **(if 0 skip to 06)**	☐☐
03.	At what age were you at delivery of first pregnancy? (how old were you at the delivery of your first pregnancy)	☐☐
04.	How many deliveries resulted in a live baby? (**If 0 skip to Q06**)	☐☐
	Live birth date 1	☐☐ / ☐☐/ ☐☐☐☐<br>dd mm yyyy
	Live birth date 2	☐☐ / ☐☐/ ☐☐☐☐<br>dd mm yyyy
	Live birth date 3	☐☐ / ☐☐/ ☐☐☐☐<br>dd mm yyyy
	Live birth date 4	☐☐ / ☐☐/ ☐☐☐☐<br>dd mm yyyy
	Live birth date 5	☐☐ / ☐☐/ ☐☐☐☐<br>dd mm yyyy
	Live birth date 6	☐☐ / ☐☐/ ☐☐☐☐<br>dd mm yyyy

	Live birth date 7	☐☐ / ☐☐/ ☐☐☐☐<br>dd mm yyyy
	Live birth date 8	☐☐ / ☐☐/ ☐☐☐☐<br>dd mm yyyy
	Stillbirth date 1	☐☐ / ☐☐/ ☐☐☐☐<br>dd mm yyyy
	Stillbirth date 2	☐☐ / ☐☐/ ☐☐☐☐<br>dd mm yyyy
	Stillbirth date 3	☐☐ / ☐☐/ ☐☐☐☐<br>dd mm yyyy
06.	Number of spontaneous abortions/miscarriages	☐☐
07.	Number of elective terminations	☐☐
08.	Number of ectopic pregnancies	☐☐
09.	Number of molar pregnancies	☐☐
10.	Number of vaginal deliveries	☐☐
11.	Number of C-Sections	☐☐
12.	Did you have any problems/complications during any of your deliveries?<br>**If No skip to Q14**	☐ (0) No **Skip to Q14**<br>☐ (1) Yes<br>☐ (99) **N/A Skip to Q14**
13.	If yes, what complications did you have? Please circle whatsoever applies.	☐ (1) Cord prolapse/cord around the neck<br>☐ (2) Perineal lacerations<br>☐ (3) Abnormal foetal heart rate or rhythm<br>☐ (4) Amniotic cavity issues/Premature rupture of membrane<br>☐ (5) Failure to progress/Obstructed labour

		☐ (6) Antepartum or Postpartum haemorrhage<br><br>☐ Others; specify
14.	Do you smoke?	☐ (0) No<br><br>☐ (1) Previous smoker<br><br>☐ (2) Current smoker
15.	Is there a family history of: tick all that applies?	☐ (1) Congenital abnormalities/genetic disease<br><br>☐ (2) Consanguinity<br><br>☐ (0) None applicable

Significant medical history

16.	Does the participant have any of these conditions: tick all that applies	
	**Disease syndrome**	**Status**
	Hypertension	☐ (0) No<br><br>☐ (1) Previous pregnancies<br><br>☐ (2) Current pregnancy
	Diabetes	☐ (0) No<br><br>☐ (1) Previous pregnancies<br><br>☐ (2) Current pregnancy
	HIV Positive	☐ (0) No ☐ (1) Yes
	If HIV positive, are you on ARTs?	☐ (0) No ☐ (1) Yes
	If on ARTs, which type?	☐ (1) 5A<br><br>☐ (2) Others (specify)
	Anaemia in previous pregnancies	☐ (0) No ☐ (1) Yes ☐ (99) NA
	Syphilis (VDRL) positive?	☐ (0) No ☐ (1) Yes<br><br>☐ (99) Unknown/Not done

	If yes treatment given?	☐ (0) No ☐ (1) Yes
	Other Specify<br>____________________________	☐ (0) No ☐ (1) Yes

History of drug allergies

17.	Have you ever experienced an allergic reaction to any drug?	☐ (0) No<br>☐ (1) Yes
	If yes	
	Drug Name/Class	Type of Reaction

STAFF INITIALS ____

24.1.9. PHYSICAL EXAMINATION

Gestational age by USS (Weeks + days): ☐☐.☐

Physical Examination

Vital signs: This time I would like to check your blood pressure, body temperature, pulse rate and respiratory rate

☐ Not done

01.	Actual time of measurement (in 24hrs format)	☐☐:☐☐<br>Hrs : Mins
02.	Sitting position Blood Pressure	________/________mmHg<br>Systolic Diastolic
03.	Pulse Rate	________beats/minute
04.	Respiratory rate	________cycles/minute
05.	Axillary Temperature	______.______ degrees Celsius
06.	Weight 1<br>Weight 2<br>Average	☐☐☐.☐☐Kgs<br>☐☐☐.☐☐Kgs<br>☐☐☐.☐☐Kgs
07.	Height 1<br>Height 2<br>Average	☐☐☐.☐☐cm<br>☐☐☐.☐☐cm<br>☐☐☐.☐☐cm
	Staff ID (Indicate initials for the staff who checked the measurements above)	

			**If abnormal specify**
08.	General Appearance	☐ (1) Normal<br>☐ (2) Abnormal<br>☐ (3) Not done	
09.	Head	☐ (1) Normal	

		☐ (2) Abnormal<br>☐ (3) Not done	
10.	Ears	☐ (1) Normal<br>☐ (2) Abnormal<br>☐ (3) Not done	
11.	Eyes	☐ (1) Normal<br>☐ (2) Abnormal<br>☐ (3) Not done	
12.	Nose	☐ (1) Normal<br>☐ (2) Abnormal<br>☐ (3) Not done	
13.	Mouth	☐ (1) Normal<br>☐ (2) Abnormal<br>☐ (3) Not done	
14.	Throat	☐ (1) Normal<br>☐ (2) Abnormal<br>☐ (3) Not done	
15.	Neck	☐ (1) Normal<br>☐ (2) Abnormal<br>☐ (3) Not done	
16.	Thyroid	☐ (1) Normal<br>☐ (2) Abnormal<br>☐ (3) Not done	
17.	Lungs	☐ (1) Normal<br>☐ (2) Abnormal<br>☐ (3) Not done	
18.	Heart	☐ (1) Normal<br>☐ (2) Abnormal<br>☐ (3) Not done	
19.	Abdomen	☐ (1) Normal<br>☐ (2) Abnormal<br>☐ (3) Not done	
20.	Musculoskeletal	☐ (1) Normal<br>☐ (2) Abnormal<br>☐ (3) Not done	
21.	Extremities	☐ (1) Normal<br>☐ (2) Abnormal<br>☐ (3) Not done	
22	Skin	☐ (1) Normal<br>☐ (2) Abnormal<br>☐ (3) Not done	
23.	Lymph Nodes	☐ (1) Normal<br>☐ (2) Abnormal<br>☐ (3) Not done	
24.	Pulses	☐ (1) Normal<br>☐ (2) Abnormal<br>☐ (3) Not done	
25.	Neurological	☐ (1) Normal<br>☐ (2) Abnormal<br>☐ (3) Not done	
26.	Genitourinary	☐ (1) Normal<br>☐ (2) Abnormal<br>☐ (3) Not done	
27.	Other (specify)	☐ (1) Normal	

		☐ (2) Abnormal<br>☐ (3) Not done	

Comments: ____

STAFF INITIALS ____

24.1.10. RANDOMISATION AND PRODUCT ADMINISTRATION

Gestational age by USS (Weeks + days): ☐☐.☐

Participant Randomization

☐ Not done

01.	Which arm has the participant been randomised?	☐ Oral iron<br>☐ FCM
	Time of randomisation	☐☐:☐☐<br>Hrs : Mins
**Medication Administration**		
	**Study Medications**	**Time of dose**
02.	FCM	Dose________________<br>Start time ☐☐:☐☐<br>Hrs : Mins<br>Finish time ☐☐:☐☐<br>Hrs : Mins
04.	If oral Iron, 90-day supply given?<br>Information to be given to participant:<br>Drug name: F/S<br>Frequency: twice a day and report to facility if experiencing any problem with medication	☐ (0) No ☐ (1) Yes
	If not given, give reasons:	______________________________

Adverse events (This section should not come up if the participant is in the oral arm)

During and after infusion
(Note: Record any adverse events occurring during and after infusion. Prompt for any symptoms of adverse events from the participant by asking the following open ended question during and after infusion)

How are you feeling?

Did the participant experience any of the following?

Tick all that apply:<br>☐ (1) Headache

☐ (2) Dizziness<br>☐ (3) Dysgeusia<br>☐ (4) Discolouration of the skin<br>☐ (5) Nausea<br>☐ (6) Vomiting<br>☐ (8) Upper abdominal pain<br>☐ (9) Dyspepsia<br>☐ (10) Anaphylactic shock<br>☐ (11) Flushing<br>☐ (12) Shortness of breath<br>☐ (13) Chest pains<br>☐ (14) Others: specify<br>☐ (0) None

(If any of these apply, fill the adverse event form)

04.	Has the participant being given insecticide treated bed net (ITN)	☐ (1) Yes<br>☐ (0) No

STAFF INITIALS ____________________

24.1.11. *PARTICIPANT LOCATOR*

PARTICIPANTS INFORMATION

Participant's Name:

Residential Address:

Contact Number (Mobile):

Alternative Contact Number (Mobile):

PHYSICAL ADDRESS

(Record GPS Coordinates

Description of home address

STAFF ID ☐☐☐☐

24.1.12. CONCOMITANT MEDICATION

Gestational age by USS (Weeks + days): ☐☐.☐

CONCOMITTANT MEDICATION

Indicate Yes if given on this visit or at the health centre		
01.	Sulphadoxine Pyrimethamine (SP)	☐ (1) Yes<br>☐ (0) No ***(if no specify ________________________)***<br>☐ N/A
02.	Albendazole	☐ (1) Yes<br>☐ (0) No ***(if no specify ________________________)***<br>☐ N/A

Any other treatment given on this visit

Medication	Start Date<br>DD/MMM/YYY	Stop Date<br>DD/MMM/YYYY	Dose and Route	Diagnosis/Indication

24.1.13. *EPDS FORM*

EDINBURGH POSTNATAL DEPRESSION SCALE (EPDS) CHICHEWA VERSION

(administered at visit 4 and 9)

**Zoyenera Kusata:** Tsopano ndikufunsani mafunso am'mene mwakhala mukuganizira masiku asanu ndi awiri apitawa. Mafunso awiri oyambilira tigwiritsa ntchito mbali imodzi ya kadi. Chithunzi chilichonse chikuimila limodzi mwa mayankho anayi. Ndizilodza zithunzi ndikamawerenga mayankho a funso lililonse. Musankhe yankho logwirilidzana ndimmene mwakhala mukumvera masiku asanu ndi awiri apitawo.<br><br>Onani chitsanzo chiri mmusichi:		
Eg.	Ndakhala ndiri okondwa mmasiku asanu ndi awiri apitawa	☐ (3) Eya, nthawi zonse<br>☐ ***(2) Eya, munthawi zambiri***<br>☐ (1) Ayi, sinthawi zonse<br>☐ (0) Ayi, olo mpangóno komwe<br>*Yankholi likuthandauza kuti mmasiku asanu ndi awiri apitawa mmunthawi zambiri munali okondwa*.
01.	Masiku asanu ndi awiri apitawa, kodi mwakhala mukutha kuseka komanso kuona kusangalatsa kwa zinthu?	☐ (0) Monga mmene mumathera nthawi zonse<br>☐ (1) Osati bwino kwambiri<br>☐ (2) Panopa osati kwambiri<br>☐ (3) Olo mpangóno komwe
02.	Masiku asanu ndi awiri apitawa, kodi mwakhala mukudikira ndi nsangala mu zinthu zozachitika mtsogolo?	☐ (0) Monga mmene mumathera nthawi zonse<br>☐ (1) Osati bwino kwambiri<br>☐ (2) Panopa osati kwambiri<br>☐ (3) Olo mpangóno pomwe
Mafunso otsatirawa tigwilisa ntchito mbali yachiwiri ya kadi. Mobwelenzanso. Chithunzi chilichonse chikuimila limodzi mwa mayankho anayi. Ndiziloza zithunzi ndikamawerenga mayankho a funso lililonse. Musankhe yankho logwilizana ndi mmene mwakhala mukumvela masiku asanu ndi awiri apitawa		
03.	Masiku asanu ndi awiri apitawa, kodi mwakhala mukumazida nokha mosafunikila pamene zithu sizinayende bwino?	☐ (0) Sizinachitikepo<br>☐ (1) Mwakamodzikamodzi

		☐ (2) Kawirikawiri<br>☐ (3) Nthawi zambiri
04.	Masiku asanu ndi awiri apitawa, kodi mwakhala mukumakhumudwa kapena kudela khawa popanda chifukwa chenicheni?	☐ (0) Olo mpangóno pomwe<br>☐ (1) Sizimachitika<br>☐ (2) Eya, nthawi zina<br>☐ (3) Eya, mwapafupipafupi (kwambiri)
05.	Masiku asanu ndi awiri apitawa, kodi mwakhala mukuchita matha kapena kusowa mtendere popanda chifukwa chenicheni?	☐ (3) Eya, kwambiri<br>☐ (2) Eya, nthawi zina<br>☐ (1) Osati kwambiri<br>☐ (0) Ayi, ngakhale pangóno pomwe
06.	Masiku asanu ndi awiri apitawa, kodi mwakhala mukuganiza kapena kumva ngati manali ndi zinthu zambiri Zoyenera kuchita koma simumakwanitsa kuchita?	☐ (3) Eya, nthawi zambiri mwakhala mukulepheleratu<br>☐ (2) Eya, nthawi zina mwakhala mukulepheleratu<br>☐ (1) Nthawi zambiri mwakhala mukutha<br>☐ (0) Mwakhala mukutha ngati mmene mumapangira nthawi zonse
07.	Masiku asanu ndi awiri apitawa, Kodi mwakhala osasangalala mpakana kumakulepheretsani kugona?	☐ (3) Eya, nthawi zambiri<br>☐ (2) Eya, kawirikawiri<br>☐ (1) Osati kawirikawiri<br>☐ (0) Ayi, olo pangóno pomwe
08.	Masiku asanu ndi awiri apitawa, kodi munali okhumudwa kapena kusowa mtendere wa mumtima?	☐ (3) Eya, nthawi zambiri<br>☐ (2) Eya, kawirikawiri<br>☐ (1) Osati kawirikawiri<br>☐ (0) Ayi, olo pangóno pomwe
09.	Masiku asanu ndi awiri apitawa, kodi mwakhala osasangalala moti mwakhala mukulira?	☐ (3) Eya, nthawi zambiri<br>☐ (2) Eya, kawirikawiri

		☐ (1) Mwakamodzikamodzi<br>☐ (0) Ayi, sizinachitikepo
10.	Masiku asanu ndi awiri apitawa, kodi munakhalapo ndi maganizo ofuna kuzipweteka?	☐ (3) Eya, nthawi zambiri<br>☐ (2) Kawirikawiri<br>☐ (1) Mwakamodzikamodzi<br>☐ (0) Sizinachitikepo

STAFF ID: ______________________________ ☐☐☐☐

24.1.14. *MIBS FORM*

MOTHER-TO-INFANT BONDING SCALE (administered at visit 4 and 9)

Malangizo:

Ndikufuna ndidziwe m'mene mwakhala mukumvera zokhudzana ndi mwana wanu cha posachedwapa. Munsimu muli muli zina zimene amai amamva zokhudzana ndi ana awo. Chonde lembani mzere pansi pa yankho limene lukugwirizanako ndi m'mene mukumvera zokhudzana ndi mwana wanu, osangoti m'mene mukumvera pa tsiku la lero. Chonde malidzitsani mfundo ZONSE.

Mwachitsanzo

bh	Ndimanva kukoma kumuchitira zinthu mwana wanga	☐ (0) Ayi, ndi pang'ono pomwe<br>☐ ***(1) <u>Pang'ono kwambiri nthawi zina</u>***<br>☐ (2) Kwambiri, nthawi zina<br>☐ (0) Kwambiri, nthawi zambiri<br>Mau awa akutanthauza kuti: "Ndimamva kukoma pang'ono kwambiri nthawi zina kumuchitira zinthu mwana wanga".
01.	Ndimamva kumukonda mwana wanga	☐ (3) Kwambiri kwake, nthawi zambiri<br>☐ (2) Kambirir kwake, nthawi zina<br>☐ (1) Pang'ono, nthawi zina<br>☐ (0) Ayi ndi pang'ono pomwe
02.	Ndimaopa kapena ndimapanikizika ndikachita china chake kwa mwana wanga	☐ (0) Ayi nkomwe<br>☐ (1) Pang'ono, nthawi zina<br>☐ (2) Kwambiri kwake, nthawi ina<br>☐ (3) Kwambiri wake, nthawi zambiriri
03.	Ndimakhala osakondwa ndi mwana wanga	☐ (3) Kwambiri kwake, nthawi zambiri<br>☐ (2) Kwambiriko kwake, nthawi zina<br>☐ (1) Pang'ono, nthawi zina<br>☐ (0) Ayi ndi pang'ono pomwe
04.	Palibe chimene ndimanva cha mwana wanga	☐ (3) Kwambiri kwake, nthawi zambiri<br>☐ (2) Kwambiri kwake, nthawi zina<br>☐ (1) Pang'ono nthawi zina

		☐ (0) Ayi ndi pang'ono pomwe
05.	Ndimakwiya ndi mwana wanga	☐ (3) Kwambiri kwake, nthawi zambiri<br>☐ (2) Kwambiri kwake, nthawi zina<br>☐ (1) Panag'ono, nthawi zina<br>☐ (0) Ayi ndi pang'ono pomwe
06.	Ndimamva kukoma kumuchitira zinthu mwana wanga	☐ (3) Ayi, ndi pang'ono pomwe<br>☐ (2) Kwambiri kwake, nthawi zina<br>☐ (1) Pang'ono kwambiri nthawi zina<br>☐ (0) Kwambiri kwake, nthawi zambiri
07.	Ndimafuna mwana wanga akanakhala wamtundu wina	☐ (3) Kwambiri kwake, nthawi zambiri<br>☐ (2) Kwambiri kwake, nthawi zina<br>☐ (1) Png'ono, nthawi zina<br>☐ (0) Ayi ndi pang'ono pomwe
08.	Ndimachimva kuti ndimamuteteza mwana wanga	☐ (0) Ayi ndi pang'ono pomwe<br>☐ (1) Pang'ono, nthawi zina<br>☐ (1) Kwambiri kwake, nthawi zina<br>☐ (0) Kwambiri kwake, nthawi zambiri
09.	Ndimalakalaka ndikanakhala opanda mwana	☐ (3) Kwambiri kwake, nthawi zambiri<br>☐ (2) Kwambiri kwake, nthawi zina<br>☐ (1) Pang'ono, nthawi zina<br>☐ (0) Ayi ndi pang'ono pomwe
10.	Ndimamva kuti ndimakhala pafupi ndi mwana wanga	☐ (0) Ayi ndi pang'ono pomwe<br>☐ (1) Pang'ono, nthawi zina<br>☐ (2) Kwambiri kwake, nthawi zina<br>☐ (3) Kwambiri kwake, nthawi zambiri

STAFF ID: ______________________________☐☐☐☐

24.1.15. *DASS-21 FORM*

DEPRESSION ANXIETY STRESS SCALE (DASS-21) CHICHEWA VERSION

(administered at visit 4 and 9)

**Zoyenera Kusata:** Chonde sonyezani kuti chiganizo chili m'munsimu chinagwira bwanji ntchito pa inu mu sabata yapitayi. Palibe mayankho 'olondola' kapena 'olakwika'. Musatenge nthawi yambiri pa chiganizo china chilichonse		
01.	Zimandivuta kubwerera m'chimake ***(kutopa)***	☐ (0) Sizinandichitikire oro ndi pang'ono pomwe<br>☐ (1) Zimandichitikira nthawi zina oro kuti mwaapo ndi apo<br>☐ (2) Zimandichitikira kwambiri panthawi zina<br>☐ (3) Zimandichitikira kwambiri kwa nthawi zambiri
02.	Ndimadziwa za kuuma kwa mkamwa mwanga ***(nkhawa)***	☐ (0) Sizinandichitikire oro ndi pang'ono pomwe<br>☐ (1) Zimandichitikira nthawi zina oro kuti mwa apo ndi apo<br>☐ (2) Zimandichitikira kwambiri panthawi zina<br>☐ (3) Zimandichitikira kwambiri kwa nthawi zambiri
03.	Zikuonetsa kuti sindimatha kumva bwino ***(kukhala okhumudwa)***	☐ (0) Sizinandichitikire oro ndi pang'ono pomwe<br>☐ (1) Zimandichitikira nthawi zina oro kuti mwa apo ndi apo<br>☐ (2) Zimandichitikira kwambiri panthawi zina<br>☐ (3) Zimandichitikira kwambiri kwa nthawi zambiri
04.	Ndinkakhala ndibvuto m'mapumidwe (monga: Kupuma mothamanga, Kubanika ngakhale ndisakugwira ntchito yolemetsa) ***(nkhawa)***	☐ (0) Sizinandichitikire oro ndi pang'ono pomwe<br>☐ (1) Zimandichitikira nthawi zina oro kuti mwa apo ndi apo

		☐ (2) Zimandichitikira kwambiri panthawi zina<br><br>☐ (3) Zimandichitikira kwambiri kwa nthawi zambiri
05.	Ndimakhala ndi ulesi pofuna kugwira ntchito ***(kukhumudwa)***	☐ (0) Sizinandichitikire oro ndi pang'ono pomwe<br><br>☐ (1) Zimandichitikira nthawi zina oro kuti mwa apo ndi apo<br><br>☐ (2) Zimandichitikira kwambiri panthawi zina<br><br>☐ (3) Zimandichitikira kwambiri kwa nthawi zambiri
06.	Ndinali ndi chizolowezi chochita zinthu ndi mkwiyo **(*Kutopa)***	☐ (0) Sizinandichitikire oro ndi pang'ono pomwe<br><br>☐ (1) Zimandichitikira nthawi zina oro kuti mwa apo ndi apo<br><br>☐ (2) Zimandichitikira kwambiri panthawi zina<br><br>☐ (3) Zimandichitikira kwambiri kwa nthawi zambiri
07.	Ndimanjenjemera (e.g. manja) ***(nkhawa)***	☐ (0) Sizinandichitikire oro ndi pang'ono pomwe<br><br>☐ (1) Zimandichitikira nthawi zina oro kuti mwa apo ndi apo<br><br>☐ (2) Zimandichitikira kwambiri panthawi zina<br><br>☐ (3) Zimandichitikira kwambiri kwa nthawi zambiri
08.	Ndimazimva kuti ndimagwiritsa ntchito kwambiri ubongo kapena kuziva kuti ubongo wanu unali wotopa ***(kutopa)***	☐ (0) Sizinandichitikire oro ndi pang'ono pomwe<br><br>☐ (1) Zimandichitikira nthawi zina oro kuti mwa apo ndi apo<br><br>☐ (2) Zimandichitikira kwambiri panthawi zina

		☐ (3) Zimandichitikira kwambiri kwa nthawi zambiri
09.	Ndimadandaula ndi nyengo zochititsa mantha zimene zimkandionetsa ngati ndine chitsiru ***(nkhawa)***	☐ (0) Sizinandichitikire oro ndi pang'ono pomwe<br>☐ (1) Zimandichitikira nthawi zina oro kuti mwa apo ndi apo<br>☐ (2) Zimandichitikira kwambiri panthawi zina<br>☐ (3) Zimandichitikira kwambiri kwa nthawi zambiri
10.	Ndimazimva kupanda chiyembekezo ***(kukhumudwa)***	☐ (0) Sizinandichitikire oro ndi pang'ono pomwe<br>☐ (1) Zimandichitikira nthawi zina oro kuti mwa apo ndi apo<br>☐ (2) Zimandichitikira kwambiri panthawi zina<br>☐ (3) Zimandichitikira kwambiri kwa nthawi zambiri
11.	Ndimazimva kubalalika ***(kutopa)***	☐ (0) Sizinandichitikire oro ndi pang'ono pomwe<br>☐ (1) Zimandichitikira nthawi zina oro kuti mwa apo ndi apo<br>☐ (2) Zimandichitikira kwambiri panthawi zina<br>☐ (3) Zimandichitikira kwambiri kwa nthawi zambiri
12.	Zimandivuta kudekha/kukhala bata ***(kutopa)***	☐ (0) Sizinandichitikire oro ndi pang'ono pomwe<br>☐ (1) Zimandichitikira nthawi zina oro kuti mwa apo ndi apo<br>☐ (2) Zimandichitikira kwambiri panthawi zina<br>☐ (3) Zimandichitikira kwambiri kwa nthawi zambiri

13.	Ndimazimva kudandaula komanso kukhumudwa ***(kukhumudwa)***	☐ (0) Sizinandichitikire oro ndi pang'ono pomwe<br><br>☐ (1) Zimandichitikira nthawi zina oro kuti mwa apo ndi apo<br><br>☐ (2) Zimandichitikira kwambiri panthawi zina<br><br>☐ (3) Zimandichitikira kwambiri kwa nthawi zambiri
14.	Palibe chimene chimandikhudza pa china chilichonse chimene chimandiletsa kupitiliza kuchita zimene ndimachita ***(kutopa)***	☐ (0) Sizinandichitikire oro ndi pang'ono pomwe<br><br>☐ (1) Zimandichitikira nthawi zina oro kuti mwa apo ndi apo<br><br>☐ (2) Zimandichitikira kwambiri panthawi zina<br><br>☐ (3) Zimandichitikira kwambiri kwa nthawi zambiri
15.	Ndimazimva kukhala ndi mantha ***(nkhawa)***	☐ (0) Sizinandichitikire oro ndi pang'ono pomwe<br><br>☐ (1) Zimandichitikira nthawi zina oro kuti mwa apo ndi apo<br><br>☐ (2) Zimandichitikira kwambiri panthawi zina<br><br>☐ (3) Zimandichitikira kwambiri kwa nthawi zambiri
16.	Ndinalibe chikhumbo-khumbo pa china chilichonse ***(nkhawa)***	☐ (0) Sizinandichitikire oro ndi pang'ono pomwe<br><br>☐ (1) Zimandichitikira nthawi zina oro kuti mwa apo ndi apo<br><br>☐ (2) Zimandichitikira kwambiri panthawi zina<br><br>☐ (3) Zimandichitikira kwambiri kwa nthawi zambiri
17.	Ndimazimva kuti ndine munthu osayenera ***(nkhawa)***	☐ (0) Sizinandichitikire oro ndi pang'ono pomwe

		☐ (1) Zimandichitikira nthawi zina oro kuti mwa apo ndi apo<br>☐ (2) Zimandichitikira kwambiri panthawi zina<br>☐ (3) Zimandichitikira kwambiri kwa nthawi zambiri
18.	Ndimazimva kukhala wokwiya-kwiya ***(kutopa)***	☐ (0) Sizinandichitikire oro ndi pang'ono pomwe<br>☐ (1) Zimandichitikira nthawi zina oro kuti mwa apo ndi apo<br>☐ (2) Zimandichitikira kwambiri panthawi zina<br>☐ (3) Zimandichitikira kwambiri kwa nthawi zambiri
19.	Ndimazindikira zimene mtima wanga umachita ngakhale ndisakuchita masewela olimbitsa thupi (e.g. kumva kukwera kwa mulingo wa kagundidwe ka mtima, mtima kusagunda kamphindi kena kake) ***(nkhawa)***	☐ (0) Sizinandichitikire oro ndi pang'ono pomwe<br>☐ (1) Zimandichitikira nthawi zina oro kuti mwa apo ndi apo<br>☐ (2) Zimandichitikira kwambiri panthawi zina<br>☐ (3) Zimandichitikira kwambiri kwa nthawi zambiri
20.	Ndimachita mantha popanda chifukwa chodziwika bwino ***(nkhawa)***	☐ (0) Sizinandichitikire oro ndi pang'ono pomwe<br>☐ (1) Zimandichitikira nthawi zina oro kuti mwa apo ndi apo<br>☐ (2) Zimandichitikira kwambiri panthawi zina<br>☐ (3) Zimandichitikira kwambiri kwa nthawi zambiri
21.	Ndimazimva kuti moyo ulibe tanthauzo ***(kukhumudwa)***	☐ (0) Sizinandichitikire oro ndi pang'ono pomwe<br>☐ (1) Zimandichitikira nthawi zina oro kuti mwa apo ndi apo

		☐ (2) Zimandichitikira kwambiri panthawi zina<br>☐ (3) Zimandichitikira kwambiri kwa nthawi zambiri

STAFF ID: ______________________________☐☐☐☐

24.1.16. PHYSICAL EXAMINATION – DELIVERY FORM

Gestational age (Weeks + days) ☐☐.☐

Section A: Physical Examination

Attach copies of Labour-ward Admission notes and partograph

VITAL SIGNS

☐ Not done

	Actual time of measurement	☐☐ ☐☐<br>Hrs : Min
	Sitting position Blood Pressure	____________/________________mmHg<br>Systolic Diastolic
	Pulse Rate	_________________ beats/minute
	Axillary Temperature	______________.___ degrees Celsius

OBSTETRICAL EXAMINATION

☐ Not done

	Fundal height	☐☐.☐☐ cm
	Fetal heart rate	☐☐☐ bpm
	Has participant felt any fetal movements in the past 24 hours	☐ (1) Yes<br>☐ (2) No
	Investigators assessment of reported fetal movement	☐ (1) Normal<br>☐ (2) Abnormal
	Fetal presentation	(Tick ONE only)<br>☐ (1) Cephalic (head down) position<br>☐ (2) Breech (bottom down) position<br>☐ (3) Transverse<br>☐ (4) Unknown

STAFF ID ____

24.1.17. PREGNANCY OUTCOME

Pregnancy outcome

1. Pregnancy outcome

☐ (1) Full term live birth
☐ (2) Premature Birth
☐ (3) Stillbirth
☐ (4) Spontaneous abortion (**if 4, skip to Question 7**)
☐ (5) Induced/elective abortion (**if 5, skip to Question 7**)

2. **Location of Delivery:** ☐ (1) Research Facility ☐ (2) Other Health Facility ☐ (3) Home

3. **Mode of delivery:** ☐ (1) Vaginal ☐ (2) Cesarean Section ☐ (3) Assisted delivery

4. If Cesarean Section, what was the indication?
____

5. After how many minutes was cord clamping done?
____

   Delivery assisted by: ______________________________
   Cadre: ______________________

6. **Was labour induced?** ☐ (1) Yes ☐ (0) No

If yes Comment: ____

7. **Was there any complication experienced during delivery?** ☐ (1) Yes ☐ (0) No
   (If any provide on the adverse events page)
   ☐ (1) Cord prolapse/cord around the neck
   ☐ (2) Perineal tear (Degree I-IV)
   ☐ (3) Abnormal fetal heart rate or rhythm
   ☐ (4) Amniotic cavity issues/Premature rupture of membrane
   ☐ (5) Failure to progress/Obstructed labour
   ☐ (6) Antepartum Haemorrhage
   ☐ (7) Postpartum Hemorrhage
   ☐ (8) Other, specify______________________________
   Estimated blood loss______________________________

Placenta weight ____________________ grams

8. **Did the mother receive a blood transfusion?**

☐ (1) Yes
☐ (0) No

If yes, how many pints? __________________

9. **If an abortion or stillbirth, was Gross visual inspection of aborted fetus/still birth done?**

☐ (0) Not done
☐ (1) Done, no observed abnormalities
☐ (2) Done, observed abnormalities

If observed abnormalities, specify: ____

10. **Date of Discharge**: ☐☐/☐☐/☐☐☐☐

1.

2. If prolonged admission, give reason ____

STAFF ID ☐☐☐☐

24.1.18. NEONATAL OUTCOME

Neonatal outcome at birth

Date of birth: ☐☐/☐☐/☐☐☐ Time of Birth: ☐☐:☐☐

Sex: ☐ (1) Male ☐ (2) Female

Birth Length: ________cm

Birth Weight: __________g

Head Circumference: _________ cm

Apgar Score_______1 min_____ 5min_____________10min_________________
Skin color_______1 min_____ 5min_____________10min_________________
Pulse Rate_______1 min_____ 5min_____________10min_________________
Reflex irritability grimace_______1 min_____ 5min_____________10min_____________
Activity_______1 min_____ 5min_____________10min_________________
Respiratory effort_______1 min_____ 5min_____________10min_________________

Did the child cry immediately after delivery? ☐ (1) Yes ☐ (0) No
If no immediate cry, what were the resuscitation measures?
☐ (1) Stimulation
☐ (2) Suction
☐ (3) Bag and Mask Ventilation, if this how long? ___________________________ mins
☐ (4) CPR, if this how long, ____ mins
☐ (5) Oxygen therapy

Did the child suckle shortly after birth? ☐ (1) Yes ☐ (0) No

Congenital malformation / anomaly: ☐ (1) Yes ☐ (0) No

If yes specify in the box below:

Was the neonate admitted into nursery? ☐ (1) Yes ☐ (0) No

If yes, what was the primary reason for admission?

Date of Admission: ☐☐/☐☐/☐☐☐☐ Time of Admission: ☐☐:☐☐

Date of Discharge: ☐☐/☐☐/☐☐☐☐ Time of Discharge: ☐☐:☐☐

Did the neonate have to be taken to a local clinic/ outpatient hospital, including emergency room (without having to be admitted) to attend any complications at birth – during the first 24 hours?

☐ (1) Yes
☐ (0) No

If Yes; please provide the number of times and the reason for the visit in the comment section:

Date of Visit: ☐☐/☐☐/☐☐☐☐ **Time of Visit:** ☐☐:☐☐

STAFF ID ☐☐☐☐

24.1.19. *PLACENTAL HISTOPATHOLOGY FORM*

PLACENTAL HISTOPATHOLOGY EVALUATION

**A. Evaluation method, Sample preservation and adequacy**<br>**3.** ☐ **EVAL**<br>4. ☐ **QC**		
01.	Evaluation. Type of light Microscopy used	☐ *(1) Conventional*<br>☐ *(2) Polarized*<br>☐ *(3) Both*
02.	Type of stain used	☐ *(1) H & E*<br>☐ *(2) Giemsa*<br>☐ *(3) Other* ***Specify*****________________**
03.	Formalin pigment	☐ *(0) Absent*<br>☐ *(1) Mild*<br>☐ *(2) Moderate*<br>☐ *(4) Abundant*
04.	Erythrocytes in intervillous space	☐ *(0) Absent*<br>☐ *(1) Mild*

		☐ *(2) Moderate*<br>☐ *(4) Abundant*
05.	Decidual Basalis present	☐ *(0) No*<br>☐ *(1) Yes*
06.	Chorion present	☐ *(0) No*<br>☐ *(1) Yes*
07.	Amnios present	☐ (0) No<br>☐ (1) Yes

**B. Parasites/Malaria pigment**		
01.	Malaria Parasites present	☐ *(0) No*<br>☐ *(1) Yes*<br>***If No skip to Q3***
02.	Percentage of parasitized Maternal erythrocytes	☐ *(1) <1%*<br>☐ *(2) 1-10%*<br>☐ *(3) >10%*
03.	Malaria pigment (excluding parasites)	☐ *(0) Absent*<br>☐ *(1) Mild*<br>☐ *(2) Moderate*<br>☐ *(4) Abundant*
04.	Malaria pigment in free macrophages	☐ *(0) No*<br>☐ *(1) Yes*
05.	Malaria Pigment within Fibrin	☐ *(0) No*<br>☐ *(1) Yes*
06.	Parasitized Fetal Erythrocytes or Pigment villi	☐ *(0) No*<br>☐ *(1) Yes*

**C. Other abnormalities**		
01.	Intervillous inflammation, Leukocytes	☐ *(1) <5*<br>☐ *(2) 6-10*<br>☐ *(3) 11-12*<br>☐ *(4) >25*
02.	Leucocyte Predominance	☐ *(1) Neutrophils*<br>☐ *(2) Monocytes and Macrophages*

03.	Chorionitis	☐ *(0) Absent*<br>☐ *(1) Present*
04.	Amnionitis	☐ *(0) Absent*<br>☐ *(1) Present*

D. Diagnosis/Comments

1.
2.
3.

**Case report completed by**	
**Signature**	
**Date**	☐☐☐☐☐☐☐☐☐

24.1.20. *INFANT PHYSICAL EXAMINATION*

Infant Physical Examination

INFANT VITAL SIGNS

☐ Not done

	Temperature	☐☐.☐ degrees Celsius
	Respiratory rate	☐☐☐ breaths per min
	Heart rate	☐☐☐ beats per min

INFANT ANTHROPOMETRY

☐ (1) Not done

	Weight	☐☐☐☐ gm
	Height	☐☐☐.☐☐cm
	Head Circumference	☐☐☐. ☐☐cm
	MUAC (Mid upper arm circumference)	☐☐☐. ☐☐cm

INFANT PHYSICAL EXAMINATION

☐ Not done

	Head	☐ (1) Normal<br>☐ (2) Abnormal<br>☐ (3) Not done	If abnormal, state any anomalies noted
	Eyes	☐ (1) Normal<br>☐ (2) Abnormal<br>☐ (3) Not done	
	Ears<br><br><br><br>Nose	☐ (1) Normal<br>☐ (2) Abnormal<br>☐ (3) Not done<br><br>(1) Normal<br>(2) Abnormal<br>(3) Not done	
	Mouth	☐ (1) Normal<br>☐ (2) Abnormal<br>☐ (3) Not done	
	Neck	☐ (1) Normal<br>☐ (2) Abnormal<br>☐ (3) Not done	
	Chest	☐ (1) Normal<br>☐ (2) Abnormal<br>☐ (3) Not done	
	Abdomen	☐ (1) Normal<br>☐ (2) Abnormal<br>☐ (3) Not done	
	Back	☐ (1) Normal<br>☐ (2) Abnormal<br>☐ (3) Not done	
	Genital area	☐ (1) Normal<br>☐ (2) Abnormal<br>☐ (3) Not done	
	Extremities	☐ (1) Normal	

		☐ (2) Abnormal<br>☐ (3) Not done	

STAFF ID

24.1.21. *INFANT MORBIDITY*

1.	Did your child fall sick/ill since our last meeting?	☐ (1) Yes<br>☐ (0) No<br><br>If yes how many times was the child sick?<br>(1) ☐ (2) ☐ (3) ☐ (4) ☐ (>4) ☐<br><br>How many days at each occasion?<br>1st episode (1) ☐ (2) ☐ (3) ☐ (4) ☐ (>4) ☐<br>2nd episode (1) ☐ (2) ☐ (3) ☐ (4) ☐ (>4) ☐<br>3rd episode (1) ☐ (2) ☐ (3) ☐ (4) ☐ (>4) ☐<br>4th episode (1) ☐ (2) ☐ (3) ☐ (4) ☐ (>4) ☐<br>5th episode (1) ☐ (2) ☐ (3) ☐ (4) ☐ (>4) ☐<br>6th episode (1) ☐ (2) ☐ (3) ☐ (4) ☐ (>4) ☐
2.	Did your child suffer from fever in the past month?	☐ (1) Yes<br>☐ (0) No<br><br>If yes how many times?<br>(1) ☐ (2) ☐ (3) ☐ (4) ☐ (>4) ☐<br><br>How many days at each occasion?<br>1st episode (1) ☐ (2) ☐ (3) ☐ (4) ☐ (>4) ☐<br>2nd episode (1) ☐ (2) ☐ (3) ☐ (4) ☐ (>4) ☐<br>3rd episode (1) ☐ (2) ☐ (3) ☐ (4) ☐ (>4) ☐<br>4th episode (1) ☐ (2) ☐ (3) ☐ (4) ☐ (>4) ☐

		5th episode (1) ☐ (2) ☐ (3) ☐ (4) ☐ (>4) ☐<br>6th episode (1) ☐ (2) ☐ (3) ☐ (4) ☐ (>4) ☐<br><br>Was a malaria test done?<br>☐ (1) Yes<br>☐ (0) No<br><br>(if yes, indicate outcome i.e. whether positive or negative).<br>☐ (1) Yes<br>☐ (0) No
3.	Did your child suffer from diarrhoea (passage of three or more loose or liquid stools per day) in the past month?	☐ (1) Yes<br>☐ (0) No<br><br>If yes how many times did you child have diarrhea?<br>(1) ☐ (2) ☐ (3) ☐ (4) ☐ (>4) ☐<br><br>How many days at each occasion?<br>1st episode (1) ☐ (2) ☐ (3) ☐ (4) ☐ (>4) ☐<br>2nd episode (1) ☐ (2) ☐ (3) ☐ (4) ☐ (>4) ☐<br>3rd episode (1) ☐ (2) ☐ (3) ☐ (4) ☐ (>4) ☐<br>4th episode (1) ☐ (2) ☐ (3) ☐ (4) ☐ (>4) ☐<br>5th episode (1) ☐ (2) ☐ (3) ☐ (4) ☐ (>4) ☐<br>6th episode (1) ☐ (2) ☐ (3) ☐ (4) ☐ (>4) ☐
4.	Did your child suffer from vomiting in the past month?	☐ (1) Yes<br>☐ (0) No<br><br>If yes how many times?<br>(1) ☐ (2) ☐ (3) ☐ (4) ☐ (>4) ☐

		How many days at each occasion?<br>1st episode (1) ☐ (2) ☐ (3) ☐ (4) ☐ (>4) ☐<br>2nd episode (1) ☐ (2) ☐ (3) ☐ (4) ☐ (>4) ☐<br>3rd episode (1) ☐ (2) ☐ (3) ☐ (4) ☐ (>4) ☐<br>4th episode (1) ☐ (2) ☐ (3) ☐ (4) ☐ (>4) ☐<br>5th episode (1) ☐ (2) ☐ (3) ☐ (4) ☐ (>4) ☐<br>6th episode (1) ☐ (2) ☐ (3) ☐ (4) ☐ (>4) ☐
5.	Did your child suffer from a cough in the past month?	☐ (1) Yes<br>☐ (0) No<br><br>If yes how many times?<br>(1) ☐ (2) ☐ (3) ☐ (4) ☐ (>4) ☐<br><br>How many days at each occasion?<br>1st episode (1) ☐ (2) ☐ (3) ☐ (4) ☐ (>4) ☐<br>2nd episode (1) ☐ (2) ☐ (3) ☐ (4) ☐ (>4) ☐<br>3rd episode (1) ☐ (2) ☐ (3) ☐ (4) ☐ (>4) ☐<br>4th episode (1) ☐ (2) ☐ (3) ☐ (4) ☐ (>4) ☐<br>5th episode (1) ☐ (2) ☐ (3) ☐ (4) ☐ (>4) ☐<br>6th episode (1) ☐ (2) ☐ (3) ☐ (4) ☐ (>4) ☐<br><br>Were there signs of “fast breathing, shortness of breath wheezing, or chest in-drawing”?<br>☐ (1) Yes<br>☐ (0) No
6.	Did your child have any other symptoms?	☐ (1) Yes (**specify**)<br>☐ (0) No

		If yes how many times?<br>(1) ☐ (2) ☐ (3) ☐ (4) ☐ (>4) ☐<br><br>How many days at each occasion?<br>1st episode (1) ☐ (2) ☐ (3) ☐ (4) ☐ (>4) ☐<br>2nd episode (1) ☐ (2) ☐ (3) ☐ (4) ☐ (>4) ☐<br>3rd episode (1) ☐ (2) ☐ (3) ☐ (4) ☐ (>4) ☐<br>4th episode (1) ☐ (2) ☐ (3) ☐ (4) ☐ (>4) ☐<br>5th episode (1) ☐ (2) ☐ (3) ☐ (4) ☐ (>4) ☐<br>6th episode (1) ☐ (2) ☐ (3) ☐ (4) ☐ (>4) ☐
7.	Did you seek treatment on any of the occasions?	☐ (1) Yes<br>☐ (0) No<br><br>Where was treatment sought?<br>☐ (1) Hospital<br>☐ (2) Health Centre<br>☐ (3) Private clinic<br>☐ (4) Drug store/pharmacy<br>☐ (5) Traditional healer/Herbalist<br>☐ (6) Other (**Specify**)
8.	Feeding habits	Tick all that applies<br>☐ (1) Breastfeeding<br>☐ (2) Bottle feeding<br>☐ (3) semi solid food (phala)<br>☐ (4) Normal family meals

STAFF INITIALS________________________________

24.1.22. *INFANT VACCINATION HISTORY*

Age in months and weeks ☐☐.☐

Vaccination history

Has the infant received the following vaccines? (Use documented evidence)

Visit	Vaccine received at	Vaccine	
☐ **Visit 8** (1 month post-partum)	Birth	BCG	☐ (0) No (If No reason)<br>☐ (1) Yes (If Yes date received)<br>☐ (99) Documentation not available
		OPV (Polio 0)	☐ (0) No (If No reason)<br>☐ (1) Yes (If Yes date received)<br>☐ (99) Documentation not available
☐ **Visit 9 (3 months post-partum)**	6 weeks	OPV 1	☐ (0) No (If No reason)<br>☐ (1) Yes (If Yes date received)<br>☐ (99) Documentation not available
		DPT-HepB-Hib1	☐ (0) No (If No reason)<br>☐ (1) Yes (If Yes date received)<br>☐ (99) Documentation not available
		PVC 1	☐ (0) No (If No reason)<br>☐ (1) Yes (If Yes date received)<br>☐ (99) Documentation not available
		Rotavirus vaccine 1	☐ (0) No (If No reason)<br>☐ (1) Yes (If Yes date received)<br>☐ (99) Documentation not available
	10 weeks	OPV 2	☐ (0) No (If No reason)<br>☐ (1) Yes (If Yes date received)<br>☐ (99) Documentation not available
		DPT-HepB-Hib2	☐ (0) No (If No reason)<br>☐ (1) Yes (If Yes date received)<br>☐ (99) Documentation not available
		PCV 2	☐ (0) No (If No reason)<br>☐ (1) Yes (If Yes date received)<br>☐ (99) Documentation not available
		Rotavirus vaccine 2	☐ (0) No (If No reason)<br>☐ (1) Yes (If Yes date received)<br>☐ (99) Documentation not available
☐ **Visit 10 (6 months post-partum)**	14 weeks	OPV 3	☐ (0) No (If No reason)<br>☐ (1) Yes (If Yes date received)<br>☐ (99) Documentation not available
		DPT-HepB-Hib3	☐ (0) No (If No reason)<br>☐ (1) Yes (If Yes date received)<br>☐ (99) Documentation not available
		PCV 3	☐ (0) No (If No reason)

			☐ (1) Yes (If Yes date received)<br>☐ (99) Documentation not available
☐ **Visit 11 (9 months post-partum)**	6 months	Vitamin A	☐ (0) No (If No reason)<br>☐ (1) Yes (If Yes date received)<br>☐ (99) Documentation not available
☐ **Visit 12 (12 months post-partum)**	9 months	Measles 1	☐ (0) No (If No reason)<br>☐ (1) Yes (If Yes date received)<br>☐ (99) Documentation not available

STAFF INITIALS ____________________

24.1.23. *HOUSEHOLD FOOD INSECURITY*

Household Food Insecurity Access Scale (HFIAS)

Instruction: Each of the questions in the following table will be asked with a recall period of four weeks (30 days). The respondent is first asked an occurrence question – that is, whether the condition in the question happened at all in the past four weeks (yes or no). If the respondent answers "yes" to an occurrence question, a frequency-of-occurrence question is asked to determine whether the condition happened rarely (once or twice), sometimes (three to ten times) or often (more than ten times) in the past four weeks.

Example:

1. In the past four weeks, did you worry that your household would not have enough food?

   0 = No (skip to Q2)

   1 = Yes

1.a. How often did this happen?

   1 = Rarely (once or twice in the past four weeks)

   2 = Sometimes (three to ten times in the past four weeks)

   3 = Often (more than ten times in the past four weeks)

No	Question	Response option	Code
1.<br><br>1.a	In the past four weeks, did you worry that your household would not have enough food?<br><br>How often did this happen in the past four weeks? (1=Rarely, 2 =Sometimes, 3 = Often)	0 = No (skip to Q.8)<br>1 = Yes	

2<br><br>2a	In the past four weeks, were you or any household member not able to eat the kinds of foods you preferred because of a lack of resources?<br><br>How often did this happen in the past four weeks? (1=Rarely, 2 =Sometimes, 3 = Often)	0 = No (skip to Q.8)<br><br>1 = Yes	
3<br><br>3a	In the past four weeks, did you or any household member have to eat a limited variety of foods due to a lack of resources?<br><br>How often did this happen in the past four weeks? (1=Rarely, 2 =Sometimes, 3 = Often)	0 = No (skip to Q.8)<br><br>1 = Yes	
4<br><br>4a	In the past four weeks, did you or any household member have to eat some foods that you really did not want to eat because of a lack of resources to obtain other types of food?<br><br>How often did this happen in the past four weeks? (1=Rarely, 2 =Sometimes, 3 = Often)	0 = No (skip to Q.8)<br><br>1 = Yes	
5<br><br>5a	In the past four weeks, did you or any household member have to eat a smaller meal than you felt you needed because there was not enough food?<br><br>How often did this happen in the past four weeks? (1=Rarely, 2 =Sometimes, 3 = Often)	0 = No (skip to Q.8)<br><br>1 = Yes	
6<br><br>6a	In the past four weeks, did you or any household member have to eat fewer meals in a day because there was not enough food?<br><br>How often did this happen in the past four weeks? (1=Rarely, 2=Sometimes, 3 = Often)	0 = No (skip to Q.8)<br><br>1 = Yes	
7<br><br>7a	In the past four weeks, was there ever no food to eat of any kind in your household because of lack of resources to get food?<br><br>How often did this happen in the past four weeks?	0 = No (skip to Q.8)<br><br>1 = Yes	
8<br><br>8a	In the past four weeks, did you or any household member go to sleep at night hungry because there was not enough food?<br><br>How often did this happen in the past four weeks?	0 = No (skip to Q.8)<br><br>1 = Yes	
9<br><br>9a	In the past four weeks, did you or any household member go a whole day and night without eating anything because there was not enough food?<br><br>How often did this happen in the past four weeks?	0 = No (skip to Q.8)<br><br>1 = Yes	

24.1.24. CHILD DIETARY DIVERSITY

A12	Now I would like to ask you about (other) liquids or foods that ***NAME)*** ate yesterday during the day or at night. I am interested in whether your child had the item even if it was combined with other foods. For example, if ***(NAME)*** ate a millet porridge made with a mixed vegetable sauce, you should reply yes to any food I ask about that was an ingredient in the porridge or sauce. Please do not include any food used in a small amount for seasoning or condiments (like chilies, spices, herbs, or fish powder), I will ask you about those foods separately. Yesterday during the day or at night, did ***(NAME)*** drink/eat:		YES	NO	DK
A	Bread, rice, noodles, or other foods made from grains, including thick grain-based porridge?	A..........	1	2	8
B	Pumpkin, carrots, squash, or sweet potatoes that are yellow, or orange inside?	B..........	1	2	8
C	White potatoes, white yams, manioc, cassava, or any other foods made from roots?	C..........	1	2	8
D	Any dark green leafy vegetables?	D..........	1	2	8
E	Ripe mangoes, ripe papayas, or (**insert other local vitamin-A rich fruits**)?	E..........	1	2	8
F	Any other fruits, or vegetables?	F..........	1	2	8
G	Liver, kidney, heart, or other organ meats?	G..........	1	2	8
H	Any meat, such as beef, pork, lamb, goat, chicken, or duck?	H..........	1	2	8
I	Eggs?	I..........	1	2	8
J	Fresh or dried fish, shellfish, or seafood?	J..........	1	2	8
K	Any foods made from beans, peas, lentils, or nuts?	K..........	1	2	8
L	Cheese, yogurt, or other milk products?	L..........	1	2	8
M	Any oil, fats, or butter, or foods made with any of these?	M..........	1	2	8
N	Any sugary foods such as chocolates, sweets, candies, pastries, cakes, or biscuits?	N..........	1	2	8
O	Condiments for flavor, such as chilies, spices, herbs, or fish powder?	O..........	1	2	8
P	Grubs, snails, or insects?	P..........	1	2	8
Q	Foods made with red palm oil, red palm nut, red palm nut pulp sauce	Q..........	1	2	8
R	Any other solid or semi-solid food	R..........	1	2	8

24.1.25. CHILD STIMULATORY CARE

Family care indicator questionnaire

4. *If the information giver (respondent) is other than mother, then mention her name:*
5.
6. *I want to know about those things with which the child plays at home. Please show me those things. These may be home made e.g. home made clay-built toy, doll made up of cloths or toy which is bought & household materials etc. The question should be coded. When mother will show these toys, the question will help her to recall other toys present at home. Code only those toys which the mother can show. Not only the presence of the toys will do, but also these toys should be used for specific play or work mentioned in the following questions.*
   1. *In the last 30 days (Child name) did the child play with any toy that can make music or can be played as musical instrument (e.g. musical instrument or the toys that produce musical sound e.g. plastic mobile as toy, radio as toy, singing doll, tom-tom, pipe etc.)?*

7. *1= Yes 2= No*

2. *In the last 30 days did the child (name) play with any toy that can be used for drawing or writing purpose (e.g. picture book for coloring, pencil, pen, chalk, slate or marking / writing with stick in the floor or courtyard etc.*

8. *1= Yes 2= No*

3. *Is there any picture book suitable for the child (except school book)?*

9. *1= Yes 2= No*

10.

4. *In the last 30 days did the child (name) play with anything that disguise himself or take the role of mother, doctor, teacher, actor, doll, plate & cup for acting purpose?*

11. *1= Yes 2= No*

5. *In the last 30 days (Child name) did the child play with any toy with which he ran about (e.g. ball& bat, rope for jumping, rocking cradle made of rope, a car that can be pulled or pushed etc)?*

12. *1= Yes 2= No*

6. *Does the child have any toy with which he can get idea regarding shape (triangular, rectangular, round) & color?*

13. *1= Yes 2= No*

7. *Does the child have any toy (globular shaped, logo, block) with which tower; house, car etc can be made by placing them one over another or side by side.*

14. *1= Yes 2= No*

Instruction: Answer to the question number 8 & 9 should be in number. If the number is 10 or >10, then the answer should be 10.

8. *What is the number of book in your house including school book (except picture book for children) ……?*
9. *What is the number of paper & magazine at your house?*

15.

16. *Now I want to know from you regarding some work or play which has been played with the child by you or his father or any senior member of the family, in the last three days.*

17.

10. *Book was read or picture book/picture/poster was shown to your child by-*
    a. *Mother b. Father c. other family member who is above 15 years of old d. Not done*
11. *Story was told to the child (name) by-*
    a. *Mother b. Father c. other family member who is above 15 years of old d. Not done*

18.

12. *Song, rhyme, religious song was sung to the child (name) by-*
    a. *Mother b. Father c. other family member who is above 15 years of old d. Not done*

19.

13. *Game was played with the child (name) using toys by-*
    a. *Mother b. Father c. other family member who is above 15 years of old d. Not done*

20.

14. *Name of something, counting number & drawing was taught to the child (name) by allocating time for him by-*

21. *a. Mother b. Father c. other family member who is above 15 years of old d. Not done*

24.1.26. *BAYLEY'S ASSESSMENT*

Bayley
Scales of Infant and Toddler Development™
THIRD EDITION

Record Form

Child's name: ______________________
Sex: ☐ M ☐ F ID #: ______________________
Examiner's name: ______________________
School/Child care program: ______________________
Reason for referral: ______________________

Subtest Summary Scores

Subtest	Total Raw Score	Scaled Score	Composite Score	Percentile Rank	Conf. Interval (___%)
**Cognitive** (Cog)					
			Use Table A.5		
**Language** (Lang)					
Receptive Communication (RC)					
Expressive Communication (EC)					
Sum					
			Use Table A.4		
**Motor** (Mot)					
Fine Motor (FM)					
Gross Motor (GM)					
Sum					
			Use Table A.4		
**Social-Emotional** (SE)					
			Use Table A.5		
**Adaptive Behavior**					
*Communication (Com)					
Community Use (CU)					
Functional Pre-Academics (FA)					
Home Living (HL)					
*Health and Safety (HS)					
*Leisure (LS)					
*Self-Care (SC)					
*Self-Direction (SD)					
*Social (Soc)					
*Motor (MO)			(GAC)		
Sum					
			Use Table A.6		

*For children younger than one year, the GAC is calculated using only those skill areas indicated by an asterisk.

Calculate Age and Start Point

	Years	Months	Days
Date Tested			
Date of Birth			
Age			
Age in Months and Days	Years × 12 + months		
Adjustment for Prematurity	Adjust through 24 months		
Adjusted Age			
Start Point	Calculate start point according to chart below		

Age	Start Point
16 days–1 month 15 days	A
1 month 16 days–2 months 15 days	B
2 months 16 days–3 months 15 days	C
3 months 16 days–4 months 15 days	D
4 months 16 days–5 months 15 days	E
5 months 16 days–6 months 15 days	F
6 months 16 days–8 months 30 days	G
9 months 0 days–10 months 30 days	H
11 months 0 days–13 months 15 days	I
13 months 16 days–16 months 15 days	J
16 months 16 days–19 months 15 days	K
19 months 16 days–22 months 15 days	L
22 months 16 days–25 months 15 days	M
25 months 16 days–28 months 15 days	N
28 months 16 days–32 months 30 days	O
33 months 0 days–38 months 30 days	P
39 months 0 days–42 months 15 days	Q

ISBN 015402723-5

PEARSON

Copyright © 2006, 1993, 1984, 1969 by NCS Pearson, Inc.
All rights reserved. Printed in the United States of America.
8 9 10 11 12 A B C D E

PsychCorp

9780154027238

24.1.27. ADVERSE EVENTS

Gestational age by USS (Weeks + days):

Age: Gravidity: Parity:

Adverse events

Note: A clinical condition which is diagnosed prior to enrolment (at baseline) is a pre-existing condition and should be documented as part of a participant's medical history and not as an adverse event.

Please record all adverse events observed or reported regardless of suspected treatment causality.

DESCRIPTION OF ADVERSE EVENT

	Description of adverse event	
	Time of onset	☐☐ :☐☐<br>Hrs Mins

(Allow room to record more than one adverse event and time of onset of each)

SEVERITY (Check 1 only)

	☐ (1) Grade 1<br>☐ (2) Grade 2<br>☐ (3) Grade 3<br>☐ (4) Grade 4<br>☐ (5) Grade 5
	Do serious criteria apply? ☐ (1) Yes, **If Yes, Notify PI immediately**<br>☐ (2) No
	If yes, please specify:<br>☐ Death<br>☐ life-threatening<br>☐ In-patient hospitalization, prolongation of existing hospitalization<br>☐ Persistent or significant disability/incapacity<br>☐ Congenital anomaly/birth defect<br>☐ Important medical event (i.e. may jeopardize participant and may require medical/surgical interventions to prevent above outcomes)

ACTION (check all relevant actions)

**Study treatment arm: Control (Oral Ferrous Sulphate)**

**If IV arm, event occurred:** ☐ During drug administration ☐ After drug administration<br><br>**Action taken on study treatment:** (Tick one only and enter details in study treatment section)		
	☐ (0) Continued<br>☐ (1) Increased<br>☐ (2) Reduced<br>☐ (3) Stopped temporarily<br>☐ (4) Permanently discontinued	
**Participant:** (Tick all relevant options and enter details in concomitant treatment section if applicable		
	☐ (1) Withdrawn from study<br>☐ (2) Treatment given (specify details in concomitant treatment section)<br>☐ (3) Other Specify ______________________<br>☐ (4) No action	

OUTCOME OF AE

	Still present/ongoing?	☐ (1) Yes<br>☐ (0) No, resolved
	Date resolved:<br>☐☐ / ☐☐☐/ ☐☐☐☐<br>dd mmm yyyy	Time resolved: (24-hour clock)<br>☐☐:☐☐<br>Hrs : min
Complete the following section when causality is determined, when the event resolves, or at the end of the study, whichever comes first		

Is the participant taking any concomitant Medication? (List all medications taken up to 1 month prior to event) ☐ (1) Yes ☐ (0) No

Medication	Start Date DD/MMM/YYYY	Stop Date DD/MMM/YYYY	Total Daily Dose	Indication	Suspect
					ϒ Yes<br>ϒ No
					ϒ Yes<br>ϒ No

List Relevant Lab/Diagnostic results below OR attach copies of the results.

Relevant Laboratory Tests o No relevant laboratory tests done

Test	Collection Date (DD/MMM/YYYY)	Result	Site Normal Range	Collection Date of test previous to this SAE	Result of test previous to this SAE

Relevant Diagnostic Tests (EX: MRI, CT Scan, Ultrasound) No relevant diagnostic tests

**Test**	**Date Performed** (DD/MMM/YYYY)	**Results/Comments**

Treatment or medication given

**Medication**	**Start Date DD/MMM/YYYY**	**Stop Date DD/MMM/YYYY**	**Total Daily Dose**	**Indication**

Did participant require the following?

☐ Blood transfusion

☐ ICU admission

Any other non-medical or surgical treatment given? ☐ Yes ☐ No
If Yes, specify: ________________________________

CAUSALITY

	Is there a reasonable possibility the adverse event is related to the study drug?	☐ (1) Yes<br>☐ (0) No<br>☐ (2) Unknown
	If No, what was the most likely cause of the AE? (Tick ONE only)	☐ (1) Disease under study<br>☐ (2) Other illness, specify<br>☐ (3) Concomitant treatment (drug or non-drug, specify)<br>☐ (4) Other, Specify

STAFF INITIALS:

24.1.28. REPORTING ADVERSE EVENTS

REPORTING & ASSESSMENT OF ADVERSE EVENTS & MISSED UNSCHEDULED VISITS

Note: A clinical condition which is diagnosed prior to enrolment (at baseline) is a pre-existing condition and should be documented as part of a participant's medical history and not as an adverse event.

1. Have you experienced any health problems since our last meeting?

☐ (1) Yes
☐ (0) No (**if No skip all questions below)**

2. If Yes, did you report to the study clinic?

☐ (1) Yes (**If Yes, skip all questions below**)
☐ (0) No

3. If No, did you report to any other health facility?
☐ (1) Yes (**If Yes, fill missed unscheduled visit form and adverse event form if applicable**)
☐ (0) No (**Fill adverse event form)**

Please record all adverse events reported regardless of suspected treatment causality.

STAFF ID ☐☐☐☐

24.1.29. END OF STUDY FORM

END OF STUDY VISIT

Visit number: ☐☐☐ Visit Date: ☐☐/☐☐/☐☐☐☐

**A. STUDY OUTCOME**	
1.	Outcome of participant in this study:

	☐ (1) Completed Study<br><br>☐ (2) Withdrawn consent<br><br>☐ (3) Withdrawn for safety reason<br><br>☐ (4) Lost to follow up<br><br>☐ (5) Death (maternal)<br><br>☐ (6) Death (infant)<br><br>☐ (7) Miscarriage<br><br>☐ (8) Did not reconsent
2.	Date of last visit: ☐☐/☐☐/☐☐☐☐
3.	Last contact visit:<br><br>☐ (1) Enrolment (visit 1)<br><br>☐ (2) Pre-delivery visit: 36 weeks GA (visit 4)<br><br>☐ (3) Delivery (visit 7)<br><br>☐ (4) 1 month postpartum (Visit 8)<br><br>☐ (5) 3 months postpartum (Visit 9)<br><br>☐ (6) 6 months postpartum (Visit 10)<br><br>☐ (6) 9 months postpartum (Visit 11)<br><br>☐ (8) 12 months postpartum (Visit 12)
4	Which scheduled visits did the participant attend? (reason if missed)<br><br>☐ (1) Enrolment (visit 1)<br><br>☐ (2) Pre-delivery visit: 36 weeks GA (visit 4)<br><br>☐ (3) Delivery (visit 7)<br><br>☐ (4) 1 month postpartum (Visit 8)<br><br>☐ (5) 3 months postpartum (Visit 9)<br><br>☐ (6) 6 months postpartum (Visit 10)<br><br>☐ (7) 9 months postpartum (Visit 11)

	☐ (8) 12 months postpartum (Visit 12)

B. END OF STUDY COMPLETION INFORMATION

Completed by: ☐☐☐ Signature: ………………….. Date: ☐☐/☐☐/☐☐☐☐

Verified by: ☐☐☐ Signature: ………………….. Date: ☐☐/☐☐/☐☐☐☐

STAFF ID ☐☐☐☐

24.2. Consent forms

24.2.1. *ICF Chichewa*

Mutu wa Kafukufuku	Kafukufuku owunikira ubwino olandilira mu msempha chithandizo cha mankhwala olimbana ndi nthenda ya kuchepa kwa magazi kwa amayi aku Malawi omwe ali ndi pakati popotsera miyezi isanu ndi umodzi kapena kuti REVAMP-TT mu chingerezi
Amene akuthandiza ndi ndalama pa kafukufuku ameneyu	Training and Research Unit of Excellence (TRUE), College of Medicine, University of Malawi
Akuluakulu a zofufuzafufuza mu kafukufuku ameneyu	1. Professor Kamija Phiri<br>College of Medicine, Blantyre<br>Malawi<br>Phone: +265999957048<br><br>2. Dr Sant-Rayn Pasricha<br>Walter and Eliza Hall Institute of Medical Research, Melbourne Australia<br>Phone: +61393452618
Ma nambala a *lamya* kapena kuti foni a komiti yoyang`anira za malamulo a kafukufuku (*NHSRC*)	Ngati mukufuna kudziwa zambiri za ufulu wanu potenga nawo mbali mu kafukufukuyu mukhonza kuyimbira foni kwa mlembi wamuofesi ya NHSRC ku unduna wa za umoyo pa nambala iyi: +2651726422/418 kapena

	kutumiza kalata kudzela pa makina a intaneti ku: mohdoccenter@gmail.com
Malo ochitikira kafukufukuyu	Zipatala zazing`ono mu boma la Zomba kuno ku Malawi

Choyambilira

Mwayitanidwa kuti mutenge nawo mbali mu kafukufuku owunikira njira zopewela matenda a kuchepa kwa magazi kwa amayi a pakati. Kalatayi ikufotokozera ndondomeko ya kafukufukuyu. Ikufotokozanso za kuyeza mthupi komanso chithandizo cha mankhwala chimene chidzakhalepo. Kudziwa za mmene kafukufukuyu akhalire kudzakuthandizani kupanga chisankho ngati mukufuna kutenga nawo mbali mu kafukufukuyu. Ngati musankha kutenga nawo mbali mu kafukufukuyu mudzafunsidwa kuti musayine kalata yovomeleza kumapeto kwake. Pamene mwasayina kalata yovomelezayi ndiye kuti mukutiuza kuti:

1. Mwamvetsetsa zimene mwawerenga kapena kufotokozeledwa
2. Mwavomeleza kutenga nawo mbali mu kafukufukuyu
3. Mwavomeleza zoyezedwa komanso chithandizo cha mankhwala chimene chafotokozedwa
4. Mwavomeleza kuti tidzagwiritsa ntchito uthenga okhuza inu komanso umoyo wanu monga mmene zafotokozedwela.

Mudzapatsidwa ina mwa kalata iyi yomwe iri ndi uthenga wa kafukufukuyu komanso kuvomeleza kwanu potenga nawo mbali mukafukufukuyu kuti musunge.

Mudzakhala ndi ufulu osiya kutenga nawo mbali mu kafukufukuyu nthawi ina ili yonse ngakhale osapereka zifukwa zimene mwasiyila. Ngati panopa simukufuna kutenga nawo mbali, kapena mtsogolomu, ndipo ngati mudzasiyila pa njira kutenga nawo mbali pa kafukufuku ameneyu, izi sidzidzakhuza kalandiridwe ka chithandizo chimene mudzalandira pamane mwapita ku chipatala inu ndi mwana wanu.

Cholinga cha Kafukufuku ameneyu

Amayi a pakati a ku Malawi ali ndi kuthekela kwakukulu kuti angathe kudwala matenda ochepa magazi pamene ali oyembekezela. Nthenda ya kuchepa kwa magazi kwa mayi oyembekezela ingathe kupeleka chiopsezo kwa mayiyo pamodzi ndi mwana amene akuyembekezelayo. Njira imene ikugwiritsidwa ntchito pothana ndi vuto la kuchepa kwa magazi mthupi ndi kumwa mankhwala amene ali ngati mchere othandidzila kuonjezela magazi mthupi dzina lake ayironi. Ku mayiko a ku *Ulaya* kapena kuti ku mayiko a azungu tsopano kukupezeka mankhwala operekedwa podzera musempha/diripi amene amathandidza kuthetsa vutoli otchedwa ferric carboxymaltose. Mankhwalawa amapeleka mwayi kuti munthu alandire mchere wambiri wa ayironi mu mphindi khumi ndi zisanu zokha pamene akulandilira mankhwalawa mu njira ya jakisoni kudzela mu msempha. Mankhwalawa ndi okwera mtengo kwambiri; komabe, tikukhulupilira kuti amagwira ntchito bwino pochepetsa bvuto la kuchepa kwa magazi mthupi kuposa makhwala a aironi akumwa makamaka panthawi imene mzimai woyembekedzera wasala pang'ono kuchila/kubeleka.

Cholinga cha kafukufukuyu ndichofuna kuwunikira ubwino komanso njira yotetezeka yopelekera mankhwala amenewa kudzela mu msempha a ferric carboxymaltose kwazimayi yemwe ali ndi pakati/mimba yomwe yapitiri miyezi isanu ndi umodzi poyerekedza ndi makhwala akumwa a aironi. Mukuchita izi ndiye kuti tidzakhala tikusiyanitsa kagwiridwe ka ntchito poyerekeza ndi mankhwala wokumwawa a *aironi* makamaka polimbana ndi mavuto obwera kamba ka kupelewera kwa magazi kwa amayi oyembekezera komanso zotsatira za umoyo wa mwana kuyambira tsiku limene anabadwa mpaka kufikira atakwanitsa miyezi khumi ndi iwiri, kapena kuti chaka.

Mmene Kafukufukuyu adzayendere

Tidzakhala tikufunsa mafunso kwa amayi oyembekezela ngati inuyo amene muli ndipakati popotsera miyezi isanu ndi umodzi komanso omwe magazi awo ali ochepa kuti atenge nawo mbali mu kafukufuku ameneyu. Iwo adzayikidwa mwa kasakaniza/mwamwayi mugulu limodzi lamagulu awiri a kafukufukuyu. Gulu loyamba lidzidzalandira mankhwala a mchere owonjezela magazi otchedwa *Ferric Carboxymaltose* kapena kuti *FCM* kudzela mu msempha wa magazi ndipo gulu lachiwiri lidzidzalandila mankhwala a mchere owonjezela magazi otchedwa kuti *aironi* koma kuzela njira yakumwa. Amayi onse amene adzatenge nawo mbali mu kafukufukuyu adzidzalandila chithandizo mu nthawi imene idzakhazikitsidwe, kuphatikizaponso mankhwala oteteza ku matenda a malungo ngati mayi ali oyenela kuyikidwa pa ndandanda wa mankhwala oteteza ku malungo. Amayi amene azakhale mu gulu lolandira mankhwala a mchere owonjezela magazi mthupi kuzela mu njira ya jakisoni wa mmisempha adzalandila mankhwala otchedwa *Ferric Carboxymaltose* kapena kuti *FCM* ma miligalamu 1000 kwa mayi olemela thupi ma kiligalamu 50 kapena kuposela apo, kapena adzalandira mankhwala a mlingo okwana ma milugalamu 20 pa 1 kilogalamu ina iliyonse ngati kulemela kwa thupi lawo sikunafike ma kiligalamu 50 omwe adzapelekedwa kamodzi basi panthawi yomwe pathupi pawo pali popotsera miyezi isanu ndi umodzi. Gulu lachiwiri lidzalandila mankhwala a mchere owonjezela magazi mthupi otchedwa kuti *Ferrous Sulphate* (kapena kuti *ayironi)* omwe azidzamwa kawiri pa tsiku mpaka adzabeleke, ndipo mlingo wake udzakhala ma miligalamu 200 omwe uli pafupifupi ma miligalamu 65 a mchere wotchedwa *elemental iron*. Onse otenga nawo mbali mu kafukufukuyu adzalandila mankhwala owatetedza ku malungo otchedwa *Sulfadoxine-Pyrimethamine (SP)* malingana ndi ndondomeko ya dziko.

Ngati musankha kutenga nawo mbali mu kafukufukuyu, tidzakufunsani mafunso ena okhuza za umoyo wanu, tidzakuyezani mthupi, tidzidzatengako magazi anu pang`ono okwana masupuni aang`ono awiri paulendo uliwonse mwabwera kuno komanso tizatenga zina ndi zina zokayeza zamthupi mwanu. Tidzakufunsani kuti mubwele ku chipatala kasanu ndi kamodzi (ma ulendo sikisi). Ulendo oyamba mudzabwela pamene mimba yanu ili ndi ma sabata okwana 36 kapena kuti miyezi 9, ulendo wachiwiri pakatha mwezi umodzi kuyambira tsiku limene mwana wanu anabadwa, ulendo wachitatu patatha miyezi itatu, ulendo wachinayi patatha miyezi isanu ndi umodzi, ulendo wachisanu patatha miyezi isanu ndi inayi ndipo ulendo omaliza (wa nambala sikisi) patatha miyezi khumi ndi iwiri (kapena kuti chaka) chibadwire mwana wanu. Pamene mudzabwera pa sabata ya nambala 36 kapena kuti mwezi wa nambala 9, tizakuyezani zinthu zosiyana siyana pa thupi lanu, tidzakufunsani mafunso okhuza umoyo wanu komanso thanzi la kaganizidwe kanu ndiponso tidzakutengani magazi. Pa ulendo

omweu, tidzayezanso njira yanu ya chibelekero pogwiritsa ntchito ka chipangizo kakang`ono kamene kamakhala ndi thonje kutsogolo kwake kuti tione mitundu ya tizilombo toyambisa matenda timene tingapezeke mu njirayi. Dziwani kuti padzafunika kuti inu komanso ukondedwa anu mulandire chithandizo chamakhwala ngati pangapezeke kuti zosatira zakuyedza zasocheza kuti njira yachibelekero chanu muli tizilombo (zina mwa izo ndi monga matenda opatsirana pogonana). Chomalizira pa ulendo umenewu tidzakufunsani kuti titenge nawo pang`ono chimbudzi chanu. Ndipo mukadzabeleka, munthu mmodzi wa gulu lathu adzabwera kudzatengako pang`ono magazi kuchokera ku thumbo la pa mchombo la mwana komanso kuchokera ku mbali imodzi ya msengwa lachibelekero. Kuphatikiza apo, adzatenganso ka chiduswa kakang`ono kwambiri ka mbali imodzi ya msengwa la chibelekero kamene ka matuluka limodzi pa nthawi yobeleka. Kupima zithu zimenezi kudzatipatsa ife uthenga odziwa umoyo komanso thanzi la mwana wanu. Kuonjezela apo tidzapimanso mmene mwana wanu akuonekera mmene magazi ake akuyendera, kutakataka, mmene akupumira komanso kumupima zina ndi zina monga kumukweza sikelo kuti tione mmene akulemelela pa nthawi imene wabadwa, kutalika kwake, kutambalala kwa mutu, mmene akumvera ndi makutu ake komanso kupima kapena kumuona ngati ali ndi chilema chilli chonse. Tidzatenganso chimbudzi cha mwana wanu kuti tikachipime. Ndipo tikatero tidzakufunsaninso kuti mudzabwere pakatha mwezi umodzi, kenako pakatha miyezi itatu, ulendo wina pakatha miyezi isanu ndi umodzi, pakatha miyezi isanu ndi inayi komanso ulendo omaliza pakatha miyezi isanu ndi iwiri (chaka). Mu maulendo amenewa tidzidzakupimani pofuna kuona mmene mukupezela mthupi, tidzidzatengakonso magazi pang`ono komanso tidzidzayeza ndikukweza mwana wanu sikelo. Kuonjezela apo, mu ulendo umene mudzabwera mu mwezi oyamba mwana atabadwa, komanso ulendo umene mudzabwera patatha miyezi isanu ndi umodzi kapena kuti 6 ndi ulendo umene mudzabwera patatha miyezi khumi ndi iwiri kapena chaka, tidzidzatengako mkaka wa mmaere anu kuti tikaupime pofuna kuona mmene michere yowonjezera magazi mthupi ilili komanso thanzi lanu ndi la mwana wanu. Mumaulendo onsewa tidzidzatengakonso chimbudzi chanu komanso cha mwana wanu kuti zikapimidwe. Mukufunsidwanso kuti mudzibwera ku chipatala chino chimene kukuchitikira kafukufukuyu nthawi ina iliyonse pamene mwadwala kudzapimidwa mthupi komanso kuyezetsa magazi.

Kayikidwe ka gulu la mankhwala

Ndi zofunikira kuti mudziwe komanso kumvetsetsa kuti mudzayikidwa mu gulu limodzi mwa la mankhwala mwa mwayi chabe. Dziwani kuti palibe angakhale mmodzi mwa ife amene akudziwa zagulu lomwe mungaikidwe. Muli ndimwayi wofanana kugwera gulu limodzi mwamagulu awiriwa. Muzapatsidwa uthenga onse ofunikira kutsatira molingana ndi gulu limene mwapatsidwa/mwagwera.

Ngati zapezeka kuti muli mu gulu la anthu olandira chithandizo cha mankhwala a mchere owonjezela magazi mthupi a *FCM*, ndiye kuti mudzalandira mankhwalawa kudzela mu diripi yaying`ono imene adzalowesera mankhwalawa kudzela mu jakisoni mu msempha wa pa nkono wanu. Ndipo ngati zapezeka kuti muli mu gulu la anthu olandila chithandizo cha mankhwala a mchere owonjezela magazi lake *iron*, ndiye kuti mudzapatsidwa mpukutu wa mankhwala

kuchokera kwa adotolo anu komanso adzakupatsani ndondomeko ya kamwedwe kake ka mankhwalawo.

Chidzachitike ndi chiyani pa mapeto a chithandizo cha mankhwala amene mudzalandira

Pamene takupatsani chithandizo cha mankhwala ulendo oyamba, tidzakufunsani kuti mudzibwera ku chipatala chino chimene kafukufukuyu akuchitikira mu nthawi kapena miyezi imene tidzakupatsani kuti mudzidzalandira zithandizo zosiyanasiyana. Mabweredwe a ku chipatala chino kumene kafukufukuyu akuchitikira agawidwa motere; Ulendo oyamba mudzafika pamene mimba yanu ili ndi miyezi 36 kapena kuti yakwanisa miyezi isanu ndi inayi (9). Ulendo wachiwiri mudzabwera patatha mwezi umodzi kuyambira tsiku limene munabeleka, ulendo wachitatu mudzabwera patatha miyezi itatu, ulendo wachinayi mudzabwera patatha miyezi isanu ndi umodzi (6), ulendo wachisanu mudzabwera patatha miyezi isanu ndi inayi ndipo ulendo omaliza mudzabwera patatha miyezi khumi ndi iwiri (kapena kuti chaka). Kuonjezera apo, tidzakulimbikitsaninso kuti mudzibwera ku chipatala chino kumene kafukufukuyu akuchitikira nthawi zonse pamene mwadwala. Kuonjezela apo tikukulimbikitsaninso kuti mudzapite kukachilira ku chipatala chomwe mwauzidwa. Dziwani kuti simukuyenera kugawana mankhwalawa ndi mayi mzanu wapakati kapena munthu wina aliyense.

Kodi pali zovuta zina zimene zingathe kubwera chifukwa cha mankhwalawa?

Mankhwala ena ali wonse ali ndi zovuta zina zimene amabweretsa pamene munthu akulandira. Pali mpata ochepa kwambiri kuti mutha kumva zinthu zina mthupi monga, chizungulire pang`ono, kuphwanya malo olumikizana mafupa, komanso kumva kuwawa kwa mutu. Koma izi nthawi zambiri zimasiya zokha popanda kumwera mankhwala. Pamene mankhwala amchere owonjezera magazi mthupi kudzera pokumwa otchedwa *iron* nthawi zina amatha kupangitsa munthu kuzimbidwa, kutsekula mmimba komanso kuwawa kwa mmimba. Amathanso kupangitsa chimbuzi kusandulika chakuda. Koma izi sizoopsa ayi pa moyo wa munthu ngati zitachitika. Zigawo zili mmusimu zikuthandizani kuti mumvetsetse zovuta zina zimene mankhwala angathe kubweretsa komanso kuti kodi zimenezi zimabwera mowilikiza motani:

Zovuta Zina	Kodi Zingachitike mochuluka bwanji?	Kukula kwa vutoli kungakhale kotani?	Kodi vutoli limatenga nthawi yayitali bwanji?
Kupweteka kwa mmimba, kumva nseru, kudzimbidwa kapena kutsekula mmimba (pamene tamwa mankhwala otchedwa *Iron*)	Mwina zingachitike kwa munthu mmodzi mwa anthu anayi aliwonse	Pang`ono basi	Kwa masiku ochepa pamene mukumwa mankhwalawa, koma nthawi zambiri munthu amantha kukhala bwinobwino ngakhale osamwa mankhwala

Matenda ena obwera chifukwa cha tidzirombo toyambitsa matenda (monga kutsekula mmimba kapena malungo)	Zimachitika mofanana ndi anthu amene sakumwa mankhwala owonjezela magazi, kapena nthawi zina mochulukilako pang`ono	Mofanana ndi anthu amene sakumwa mankhwala owonjezela magazi kapena nthawi zina mochulukilapo pang'ono	Kwa masiku ochepa
Chimbudzi kukhala chakuda (pamene munthu akumwa mankhwala a Ayironi)	Zimachitika kwa anthu ambiri	Sizoopsa nkomwe	Limakhala pa nthawi imene mankhwalawa akumwedwa basi
Kuphwanya kwa thupi makamaka mu malo olumikizana mafupa (pamene munthu akulandila chithandizo kuzela mu msepha)	Mwina munthu mmodzi mwa anthu makumi awiri (20) kapena munthu mmodzi mwa anthu anthu asanu ndi atatu (8)	Kuphwanya thupi pang`ono mwa patali patali koma nthawi zambiri kumasiya okha	Masiku awiri kapena atatu
Kuchepa kwa mlingo wa mchere umene umapezekanso mmagazi dzina lake fosifeti (kwa amene akulandilira chithandizo cha mankhwala kudzela mu msempha)	Mwina kwa munthu mmodzi mwa anthu khumi (aliwonse)	Pang`ono chabe – mwinanso simudzamva kalikonse mthupi mwanu. Ndikusintha chabe kumene kumachitika mu magazi anu ndipo sizoopsa ayi. Komabe tidzakuyezaninso zimenezi.	Ma sabata anayi mwina mpaka masaba asanu ndi limodzi

Kodi kafukufukuyu adzakhuzana bwanji ndi mwana wanga?

Ngati musankha kutenga nawo mbali mu kafukufuku ameneyu ndiye kuti tidzachita monga mmene zimakhalira ku sikelo ya ana, monga kuyeza mwana wanu mmene akulemelera, usinkhu, kutambalala kwa mutu wake komanso kumupima ngati ali ndi zilema kapena matenda otengela pobadwa. Komanso tidzatengako pang`ono chimbuzi choyamba cha mwana wanu. Kuonjezera apo, tidzayeza ngati mwana wanu ali ndi vuto lina lina lili lonse la kamvedwe pogwiritsa ntchito makina a kompyuta otchedwa *Auditory Brain Response* mu chingerezi. Izi

zidzatheka pomuveka mwana wanu makina opangidwa mowoneka ngati timawaya tating'ono komanso tofewa tomwe timagwiritsidwa ntchito kumvetsera nyimbo m`makutu. Kuyeza izi sikumafuna kuti mwana wanu achitepo chili chonse, makamaka zimakhala bwino kuchita izi pamene mwanayo ali kugona mmanja mwa amayi ake. Ka nyimbo ka pansipansi kadzaseweredwa, ndiye mmene mwanayo akuchitira pamene nyimboyi ikuseweredwa zidzaoneka komanso kusungidwa mu chipangizo cha magesi dzina lake *Computer*. Kukula kwa phokoso la nyimboyo kudzakhala kolingana ndi msinkhu wa mwanayo ndipo sizidzakhala zoopsa kwa mwanayo. Ndipo izi sizidzakhala zoopsa kwa mwanayo, komanso chifukwa zimangotenga mphindi zosakwana khumi (10) basi kuti zitheke.

Pakazatha mwezi umodzi tidzakufunsani kuti mudzabwerenso inu ndi mwana wanu kuti tidzakupimeni. Pa nthawi imeneyi, tidzakwezanso mwana wanu sikelo pofuna kuona kulemela kwa mwana wanu komanso kumupima zina ndi zina. Tidzatenganso magazi pang'ono kudzela pa phazi lake kapena kudzela pa nkono wa mwana wanu kuti tiyedze zizindikilo za kusowa kwa magazi kapenanso ngati akupelewera mchere otchedwa *Iron* umene umathandiza kuwonjezela magazi mthupi. Pa tsiku lomweli tidzatengakonso pang`ono chimbuzi cha mwana wanu kuti tikachipime. Kuonjezela apo, pa ulendo umene mudzabwera patangotha mwezi umodzi pamene mwana anabadwa tidzayezanso mamvedwe a mwana wanu pogwiritsa ntchito chipangizo chimene mu chingerezi chimatchedwa kuti *Auditory Brain Response*. Tidzakufunsaninso kuti mudzabwerenso inu ndi mwana mu mwezi wa chitatu, kenako pakatha miyezi isanu ndi umodzi, pakathanso miyezi isanu ndi inayi ndipo ulendo omaliza mudzabwere pakatha miyezi khumi ndi iwiri (12) kapena kuti chaka. Mu ma ulendo onsewa tidzidzakweza mwana wanu sikelo pofuna kuona kulemela kwake, kumupima mthupi komanso kutengako pang`ono kagazi. Tidzamupimanso kamvedwe kake mmakutu mu ulendo umene mudzabwere patatha miyezi itatu komanso ulendo umene mudzabwera patatha miyezi isanu ndi umodzi. Mu ma ulendo amene mudzabwere patatha miyezi itatu, miyezi isanu ndi umodzi komanso ulendo umene mudzabwere patatha miyezi khumi ndi iwiri (12) kapena kuti chaka, tidzidzatengako pang`ono chimbuzi cha mwana wanu. Kuonjezela pa zopima zimene tafotokozazi, mu ulendo umene mudzabwera patatha miyezi isanu andi umodzi komanso ulendo umene mudzabwere patatha miyezi khumi ndi iwiri (12) kapena kuti chaka, tidzayeza mmene ubongo wa mwana wanu ukugwilira ntchito. Makamaka pofuna kuona monga mmene ubongo wa mwana wanu akuchitila pamene mwana wanu wamva phokoso kapena nyimbo imene, pamene wapatsidwa kapena kuona zithunzi, kuonela kanema kapena masewero osiyanasiyana. Zinthu izi zidzayezedwa pogwiritsa ntchito makina otchedwa *EEG* komanso njira ina yotchedwa kuti *Bayley tests*. Izi zimatheka pomuveka mwana wanu ku mutu makina amenewa omwe anapangidwa mooneka ngati chipewa. Kenako timamusewelera nyimbo, kumuonetsa chithunzi kapena kanema. Zikatero makina athu amajambula kapena kuonesa changu chimene ubongo wa mwana wanu ukuchitila, kapena kugwirira ntchito pamene wamva nyimbo kapena phokoso, waona kapena kupatsidwa chithunzi komanso pamene waonera kanema. Izi zidzatenga ka mphindi kochepa kokha koma zidzatipatsa ife uthenga okwanira pofuna kuona kuti kodi mankhwala amene tinakupatsani pamene munali oyembekezela athandizapo motani kumbali ya kaganizidwe komanso mmene ubongo wa mwana wanu ukugwirira ntchito. Monga mmene tafotokozera, poyeza izi mwana wanu tidzamuveka makina owoneka ngati chipewa koma chokhala ndi mawaya opangidwa ndi pulasitiki panja pake omwe adzathandizira kutionetsa

mmene ubongo wake ukugwilira ntchito pamene wapatsidwa kapena kuwona chithunzi kapenanso pamene wamva nyimbo kapena phokoso. Pa nthawi imene tizidzayeza izi, mwanayu adzakhala pa miyendo panu mu chipinda mmene tidzayikamo kanema komanso wayilesi yomwe idzidayimba nyimbo. Pa nthawiyi, mwana wanu sadzafunika kuchita chilichonse koma makina amene adzavale kumutuwo ndi amene adzidzayeza kapena kuonesa mmene ubongo wake ukuchitila kapena kugwirira ntchito pamene waona zithunzi kapena wamva nyimbo kapena phokoso. Njira iyi imayeza mmene ubongo wa mwana wanu ukugwirira ntchito powonesa pa makina a kompyuta zinthu zimene zikuchitika mu ubongo wa mwanayo. Inu monga mayi a mwamayu mudzakhala muli mu chipinda chomwecho pamene ntchito yopimayi ikuchitika. Zinthuzi kapena nyimbo zimene zidzagwiritsidwe ntchito popima mwana wanu zaunikilidwa kuti ndi zolingana ndi msinkhu wa mwanayo monga mwa chikhalidwe chathu ku Malawi, ndipo mwana wanu adzasangalala kwambiri powona zithunzizo komanso kumvela nyimbo zimene zidzaseweredwa! Ngati mwana wanu adzayamba kuvuta kapena kulira pa nthawi yomwe tikumuyeza, tidzaimitsa kaye zoyezayezazi ndipo tidzapitiliza pamene mwana wanu watonthola ndi kukhazikika. Mwana wanu sadzamva kupweteka kwina kulikonse panthawi imeneyi ndipo sipadzafunikira kumubaya kapena kutenga magazi. Ndipo chipewa chomwe mwana wanu azavale sichidzamupweteka mu njira ina ili yonse. Monga mmene tafotokozela, palibe chiopsezo china chili chonse pamene mwana wanu wavala chipewa chimenechi komanso ntchitoyi imangotenga mphindi zosakwana makumi awiri basi pa mwana aliyense.

Pali chiopsezo chanji pamene mwana wanga wavala chipewa chimenechi?

Monga mmene tafotokozela, palibe chiopsezo china chili chonse pamene mwana wanu wavala chipewa chimenechi, kungoti kuyezaku kumafunikira kuti mwana wanu avale makina owoneka ngati chipewawa kumutu kwake basi. Mwina nkutheka kuti mwana wanu adzakhala omangika pang`ono pa nthawi imene tikugwira ntchito yoyezayi, komabe madotolo athu amene adzayeza mwana wanu ndi akadaulo omwe akhala akugwira ntchitoyi kwa zaka zambiri ndipo adzaonetsetsa kuti chipewachi chidzakhale chaukhondo pa nthawi yomwe mwana wanu akuvala komanso kuonetsetsa kuti mwana wanu adzakhale okondwa pa nthawi yonse yomwe akuyezedwa.

Kuonjezela apo tidzayezanso kapena kumuona mwana wanu mmene akukulira komanso kuchitila mmakhalidwe ake pogwiritsa ntchito njira yotchedwa kuti *Bayley tests*. Izi zidzachitika pamene mudzabwera ku chipatala kuno mu mwezi wa nambala sikisi komanso mwezi wa khumi ndi iwiri kapena kuti chaka. Njirayi makamaka imayeza kagwiridwe ntchito ka ubongo wa mwana pa zinthu zina monga, kukumbukira zinthu, chidwi, kayankhula, kukwiya kapena kusangalala komanso kuchitachita ndi zinthu zosiyana siyana monga zidole, pamene wamva nyimbo kapena phokoso komanso kudzindikira malo komanso anthu. Kuyeza izi kumachitika poyika zidole ku tsogolo kwa mwana wanu kuti tione ngati iye angathe kuzizindikira komanso kutha kusewera nazo kapena kuchita nazo china chilichonse. Kuonjezera apo tidzaseweranso masewero osiyanasiyana ndi mwana wanu uku tikuona mmene akuchitira. Zonsezi sizoopsa kwa mwana wanu ayi.

Kodi chidzachitike ndi chiyani kwa madzi kapena magazi a mthupi mwanga amene adzatengedwa kukapimidwawo?

Mudzafunsidwa kuti mupereke chilolezo kuti tingathe kutenga nawo magazi anu, madzi kapena zina mu thupi lanu kuti zikapimidwe mu nthawi ya kafukufukuyu. Kutengedwa kwa magazi anu ndi mbali imodzi yofunika komanso yosonyeza kuti inu muli kutenga nawo mbali mu kafukufuku ameneyu. Magazi amene azatengedwa adzapimidwa kufiira kwake komanso kupezeka kwa nchere otchedwa iron kuti tione ngati chithandizo cha mankhwala chimene munalandira chagwira ntchito. Komanso tizawasungabe ndikuwagwiritsa ntchito popima za mmene chitetezo chanu cha mthupi chilili komanso kagwiridwe ntchito ka thupi lanu pofuna kuona kuti zimenezi zingakudze bwanji umoyo wa inu mayi oyembekela komanso umoyo wa mwanayo maka maka polimbana ndi matenda, thanzi komanso kakulidwe. Magazi kapena zina zimene tidzakutengani mthupi zidzasungidwa mowonetsetsa kuti chinsinsi chanu chasungidwa. Izi zidzatheka pogwitsa ntchito nambala ya chinsinsi.

Magazi kapena zina zimene tidzakhala tikutenga mthupi mwanu tidzagwiritsa tchito pofuna kupima chibadwa mtsogolo muno. Koma sitidzayeza matenda ochokera ku makolo ayi, choncho zotsatirazi sidzidzakhuza umoyo wa inu, kapena mwana wanu angakhale umoyo wa anthu a ku banja kwanu. Komanso kuyezaku sikudzalosela kapena kupeza matenda a ku makolo amene agathe kudzabwera mtsogolo. Komano tikuyembekezera kudzayeza kusiyana kwa chibadwa kumene kumakhuzana ndi kupelewera kwa magazi mthupi, thanzi la munthu, kakulidwe ka nsinkhu kapena ziwalo kapena mmene thupi lake limalimbanirana, kapena kugwidwa ndi matenda. Magazi kapena zina zimene tidzakhala tikutenga mthupi lanu ndi kuziyeza komanso zotsatira zake sidzidzapatsidwa kapena kuonetsedwa kwa wina aliyense kupatula madotolo kapena akaswiri okhawo ndi anzawo ena amene akuthandizila nawo kufukufukuyu.

Kodi phindu lotenga nawo mbali mu kafukufuku ameneyu ndi lotani?

Ngati musankha kutenga nawo mbali mu kafukufuku ameneyu ndiye kuti mudzalandila chithandizo cha mankhwala chimene nthawi zonse chimaperekedwa kwa amayi ndi ana. Komanso kuonjezela apo tidzakulimbikitsani kuti mudzibwera ku chipatala chathu chino kumene kafukufukuyu achichitikira kudzatenga chithandizo cha mankhwala ngati mukudwala makamaka pa nthawi imene kafukufukuyu ali kuchitika. Tikumvetsetsa kuti kubwera ku chipatala kuno ma ulendo ochulukirapo kungathe kukhala kotopetsa. Kotero tidzakupasani ndalama yokwanira MK 7,000.00. (~US$10)

Pamene mukutenga nawo mbali mu kafukufuku ameneyu dziwani kuti mukuthandizira kuti akaswiri a za chipatala apeze njira yabwino komanso yodalilika yothana kapena kuchiza vuto la kuchepa kwa magazi nthupi kumene thawi zina kumathanso kukhala vuto lalikulu makamaka kwa amayi mu nthawi imene ali ndi pakati. Kafukufuku ameneyu atha kubweretsa ubwino waukulu pa nkhondo yolimbana ndi kuchepa kwa magazi mthupi kuno ku Malawi komanso kuthandidza kukonza bwino zotsatira za amayi a pakati ndi ana mtsogolo muno makamaka dela lino la chigawo cha kummwera kwa dziko la Malawi komanso koposaposa dela la kuno kwanu.

Kodi pali chiopsezo cha mtundu wanji pamene ndikutenga nawo mbali mu kafukufuku ameneyu?

Ngati mupanga chisankho chotenga nawo mbali mu kafukufuku ameneyu, chiopsezo kapena zovuta zina ndi zochepa kwambiri. Mwachisanzo, ngati mwayikidwa mu gulu lolandila chithandizo cha makhwala kudzela mu msempha wa pa nkono, ndiye kuti mutha kukhala ndi ka chilonda kakang`ono kapena kumva kuwawa pang`ono pa malo pamene anakubayani jakisoni nkuyika diripi ya mankhwala, kapena pamene anabaya potengako magazi pang`ono. Palinso kuthekera kochepa zedi kuti pa malo pamene anakubayani pangathe kusanduka chilonda. Izi monga mmene tafotokozela zilibe chiopsezo pa thupi lanu chifukwa nthawi zonse timagwiritsa ntchito zipangizo zotetezedwa bwino ndi mankhwala opha ma jelemusi, komanso anthu amene akugwira ntchito mu kafukufuku ameneyu ndi akaswiri komanso ophunzitsidwa bwino ntchito yawo. Tikudziwa kuti kafukufukuyu adzidzafuna kuti mudzabwere kuno ma ulendo ochulukirapo kuposa nthawi zonse. Tikudziwa kuti izi zidzatha kukupangisani kuti nthawi zina mudzikhala otangwanika, komabe tidzidzakubwezelani ndalama ya mayendedwe kapena kuti thiransipoti yanu. Ulendo uliwonse mwabwera kuno tidzidzakupasani ndalama yokwana MK 7,000.00. Cholinga chake ndi chakuti mubwezeletse pa ndalama yanu ya mayendedwe kapena kuti thiransipoti komanso kugulira chakudya cha masana.

Komanso dziwani kuti chiopsezo kapena kuti zovuta zina pa mwana wanu mu kafukufuku ameneyu ndi chochepa kwambiri. Njira zonse za kupima inu kapena mwana wanu sizidzafuna kuti kubowola kapena kubaya khungu la inu kapena mwana wanu pokha pokha pamene tikufuna kutengako magazi kuti tikawapime. Koma dziwani kuti izi sizidzavulaza mwana wanu. Monga mmene zidzakhalira kwa inu mayi, nayenso mwana adzatha kukhala ndi kachilonda kakang`ono kapena kumva kuwawa pang`ono pa malo pamene tidzabaya ndi kitengako magazi.

Zokhuza Chinsinsi mu Kafukufukuyu

Ngati muvomeleza kutenga nawo mbali mu kafukufukuyu, ife tidzasunga mwachinsinsi dzina lanu komanso la mwana wanu kuphatikizapo uthenga umene tidzapeza kuchokera kwa inu ndi mwana wanu pamene kafukufukuyu ali kuchitika. Malamulo a dziko amafotokoza za kusungira anthu chinsinsi. Komanso chilango chake ngati munthu waphwanya lamuloli. Choncho ifenso tidzagwira ntchito potsatira malamulowo. Anthu amene angaone kapena kugwiritsa ntchito uthenga wa inu kapena mwana wanu komanso dzina lanu kapena la mwana wanu ndi okhawo amene avomelezedwa kugwira ntchito mu kafukufuku ameneyu, komanso anthu amene akugwira ntchito yoteteza anzawo mu kafukufuku ameneyu komanso kuphatikizapo akulu akulu a boma ogwira ntchito za chipatala basi. Tidzagwiritsa ntchito uthenga umene mudzatipatsa mu kafukufukuyu basi. Uthenga kapena zotsatira zimene tidzapeza mu kafukufuku ameneyu tingathe kugawana ndi anzathu a ma ofesi ena kapenanso mayika ena koma dziwani kuti sipadzakhala dzina lanu pa mwamba pa uthenga umenewu kapena zolembedwa ku mapeto a kafukufuku ameneyu.

Kodi ndi ndani waunikira kapena kulondoloza mmene kafukufukuyu adzayendere

Malamulo kapena zoyenera kutsatira pamene kafukufukuyu akuchitika zavomerezedwa ndi akomiti kapena gulu la anthu lowona za malamulo a kafukufuku lotchedwa *College of Medicine Ethics Committee kapena* kuti *COMREC* ndi a kafukufuku lotchedwa *National Health Sciences Research Committee* kapena kuti *NHSRC*. Komiti imeneyi imapezeka ku

unduna wa za umoyo. Komanso iwowa akuthandidzana ndi komiti yowona za umoyo komanso za malamulo a kafukufuku yotchedwa *Health and Research Ethics Committee* kapena kuti *HREC* imene ili nthambi ya bungwe lotchedwa kuti *Walter and Eliza Hall Institute of Medical Research* imene imapezeka mu mzinda wotchedwa *Melbourne* mu dziko la *Australia*.

Anthu ofunika kuwadziwa mu kafukufuku ameneyu

Kodi ndi ndani amene akupereka thandizo la ndalama za kafukufuku ameneyu?

Amene akupangitsa kapena kuyendesa kafukufuku ameneyu ndi mphunzitsi wankulu dzina lawo ndi a Kamija Phiri amene amagwira ntchito ku kusukulu ya ukachenjede imene maphunzitsa madotolo komanso anamwino kapena kuti ma nesi. Dzina la sukulu imeneyi ndi *College of Medicin*e. Komanso iwowa akugwira limodzi ntchitoyi ndi anzawo a Sant-Rayn Pasricha amene ndi dotolo wankulu ku sukulu yochita kafukufuku wa za umoyo yotchedwa *Walter and Eliza Hall Institute of Medical Research*. Koma chithandizo cha ndalama choyendetsera kafukufukuyu chikuchokera ku bungwe lotchedwa kuti *Bill and Melinda Gates Foundation* limene limapezeka mu dziko la America.

Uthenga owonjezera komanso amene mungathe kulumikizana nawo

Ngati mukufuna uthenga wina uliwonse owonjezera okhuza kafukufukuyu, kapena mwina ngati mmodzi wa otenga nawo mbali mu kafukufuku ameneyu wadwala mwina chifukwa chakuti mankhwala amene wamwa abweletsa zovuta zina mthupi monga mmene tinafotokozera poyamba paja, mukhoknza kutifunsa lero pompano, kapena kudzatifunsa nthawi imene tabwera kuno mtsogolomu. Koma ngati mudzakhala ndi mafunso ena mtsogolomu mukhonza kulumikizana ndi mphunzitsi wankulu a Kamija Phiri amenenso ndi wankulu wa kufukufuku ameneyu. Iwowa amapezeka ku sukulu yotchedwa *College of Medicine* ku Blantyre. Mutha kuwayimbira foni pa nambala iyi; +265999957048. Kapena muthanso kulumikizana ndi anzawo amene ndi dotolo wankulu, dzina lawo ndi a Sant-Rayn Pasricha poyimba foni pa nambala iyi; +61393452618. Iwowanso ndi nkulu wa kafukufuku koma amachokera ku sukulu yochita za kafukufuku wa za umoyo yotchedwa *Walter and Eliza Hall Institute of Medical Research* imene imapezeka mu dziko la Australia.

Kumbali yokhuza chidandaulo chilichonse makamaka chokhuzana ndi kafukufukuyu mutha kukaonana ndi amene ayikidwa kuti aziona za chidandaulo ku chipatala chimene mwana wanu akutenga nawo gawo mu kafukufukuyu. Ndipo pezani mmunsimu udindo, dzina komanso nambala ya foni ya amene mungathe kulumikizana nawo kuti mupereke chidandaulo chanu.

Kulumikizana ndi munthu wa za umoyo ndi munthu owona za chidandaulo ku malo amene kafukufuku akuchitikira

Dzina	*Zinenani Truwah*
Udindo	*Olondoloza ntchito za kafukufuku, Training and Research Unit of Excellence*

Nambala ya lamya kapena foni	*+265999413775 kapena +2650882091578*
Njira kapena adiresi yotumizira kalata pa compyuta kapena kuti intaneti	*zinenanitruwah@gmail.com*

Ngati muli ndi madandaulo a zina ndi zina zokhuza kafukufukuyu, monga mmene ntchitoyi ikuyendera, kapena mafunso aliwonse ochokera kwa inu monga mmodzi wa anthu otenga nawo mbali mutha kulumikizana ndi anthu a ku ofesi kudzela mu ma nambala a foni ali mmunsiwa:

Dzina la owunikira za umoyo komanso za malamulo a kafukufuku	**National Health Sciences Research Committee**
Oyimirira komiti yowunikira za umoyo komanso za malamulo a kafukufuku	*Dr. Collins Mitambo*
Nambala ya lamya kapena kuti foni	*+265999397913*
Njira kapena adiresi yotumizira kalata pa compyuta kapena kuti intaneti	*cmitambo@gmail.com*

Kuvomera kwa otenga nawo mbali mu kafukufukuyu kapena kuvomera kudzela kwa owayimilira

➢ Ndikuvomera kuti ndawerenga kalata ya chilolezo kapena kuti munthu wina wandiwerengera mu Chichewa

➢ Ndikuvomera kuti ndamvetsetsa zolinga za kafukufukuy, njira zimene zidzatsatidwa, komanso zovuta zina zimene zingathe kubwera pamene munthu watenga nawo mbali, komanso monga mmene zafotokozeledwa ku kalatayi yokhuza kafukufukuyi

➢ Ndikuvomera kuti ndipereka chilolezo kwa ma dotolo anga komanso ma dotolo a mwana wanga kuphatikiza akaswiri ena a za umoyo, zipatala kapena nyumba zopimila matenda kupatula chipatala chino kuti angathe kutulutsa kapena kupeleka zotsatira za kafukufukuyu, monga umoyo wa mwana wanga komanso zokhuza chithandizo cha

mankhwala kwa akaswiri ochokera ku sukulu ya ukachenjede yotchedwa College of Medicine komanso akaswiri a za chipatala ochokera ku sukulu yotchedwa *Walter and Eliza Hall Institute of Medical Research*. Izi zidzachitika makamaka pofuna kukwanilitsa cholinga cha kafukufukuyu. Ndipo ndikudziwa kuti uthenga wa ine komanso okhuza umoyo wa mwana wanga udzakhala wa chinsinsi ndipo kuti sudzapatsidwa kwa munthu wamba.

- ➢ Ndikuvomera kuti ndinali ndi mpata ofunsa mafunso ndipo ndakhutira ndi mayankho amene ndalandira
- ➢ Mwakufuna kwanga ndikuvomereza kutenga nawo mbali komanso kuti mwana wanga athanso kutenga nawo mbali mu kafukufuku ameneyu monga mmene zafotokozeledwa, komanso ndikumvetsa kuti ndine omasuka kusiya kutenga nawo mbali chimodzimodzinso mwana wanga nthawi ina iliyonse kafukufukuyu ali mkati popanda kuopa kwina kuli konse pa zokhuza chithandizo cha ku chipatala kwa ine mayi ngakhalenso mwana wanga mtsogolomu.
- ➢ Ndikumvetsa kuti ndidzapatsidwa kalata iyi ya chilolezo komanso yokhuza za kafukufukuyu kuti ndisunge ine mwini.

____ ______________________

Saini kapena chidindo cha chala Tsiku/mwezi/chaka

chachikulu cha ku manzere cha otenga nawo mbali

kapena omuyang`anira kapena omuthandizila

____ ______________________

Saini kapena chidindo cha chala chachikulu cha Tsiku/mwezi/chaka

Kumanzere cha mboni

(pokhapokha ngati otenga nawo mbali

samatha kulemba ndi kuwerenga)

____ ______________________

Saini ya *PI* kapena omuyimirira Tsiku/mwezi/chaka

24.2.2. *ICF English version*

Title	Randomized controlled trial of the effect of intravenous iron on anaemia in Malawian pregnant women in the third trimester (REVAMP-TT)
Project Sponsor	Training and Research Unit of Excellence (TRUE), College of Medicine, University of Malawi
Co - Principal Investigators	1. Professor Kamija Phiri<br>College of Medicine, Blantyre<br>Malawi<br>Phone: +265999957048<br><br>2. Dr Sant-Rayn Pasricha<br>Walter and Eliza Hall Institute of Medical Research, Melbourne, Australia<br>Phone: +61393452618
NHSRC Contacts	If you want to know more about your rights as a research participant you can contact the NHSRC Secretariat, at the ministry of health on the following details, Phone: +2651 726422/418. Email: mohdoccenter@gmail.com.
Locations	Health centres in Zomba District, Malawi

Introduction

This is an invitation for you to take part in this research project evaluating methods for preventing anaemia in pregnant women. This *Participant Information Sheet/Consent Form* tells you about the research project. It explains the tests and treatments involved. Knowing what is involved will help you decide if you want to take part in the research. If you choose to take part in the research project, you will be asked to sign the consent section at the end. By signing this consent form, you are telling us that you:

1. Understand what you have read and/or heard
2. Consent to taking part in the research project
3. Consent for the tests and treatments that are described
4. Consent to the use of your personal and health information as described.

You will be given a copy of this Participant Information and Consent Form to keep.

You will be free to withdraw from participating in this study at any time during the study without giving a reason. If you do not want to take part now, or in the future, and if you withdraw at any point from the study, this will not affect the standard of care you will receive at the hospital.

Purpose of this study

Pregnant women living in Malawi have a high chance of developing anaemia during pregnancy. Antenatal anaemia may contribute to risks for both mother and child. The standard approach for treating anaemia is oral iron. An intravenous-iron drugs, Ferric Carboxymaltose is now available in Western countries. This drug provides the opportunity to give high doses of iron in a single 15-minute infusion. This drug is currently costly; however, we believe it works better than oral iron for improving anaemia when we are trying to rescue anaemia late in pregnancy. The aim of this study is to determine the effectiveness and safety of intravenous iron administration during the third trimester of pregnancy – given as Ferric Carboxymaltose (FCM) – when compared with oral iron in improving maternal (especially anaemia) and infant (growth, birth weight and development up to 12 months postpartum) outcomes.

Study procedure

We will be asking pregnant women, such as yourself, in the third trimester of their pregnancies and with signs of having anaemia to participate in this study. They will be randomly allocated to one of two study arms: (a) intravenous iron FCM; (b) oral iron. All the women enrolled in this study will receive routine care, which may include malaria prevention drugs, if scheduled. Women in the intravenous iron intervention group will receive intravenous FCM 1000mg for body weight ≥ 50kg, or 20mg/kg for bodyweight <50kg, once during the third trimester-once during the third trimester. The control group will receive oral iron 200mg Ferrous Sulphate (approx. 65 mg elemental iron) twice daily for the duration of pregnancy. All participants will receive Sulfadoxine-Pyrimethamine (SP) as IPTp according to national guidelines if the three doses of recommended IPTp have not been already achieved.

If you choose to participate in this study, we will ask you some questions about your psychological wellbeing; we will perform medical examinations; and we will collect a small amount of blood (2 teaspoons/visit) and other biological samples. We will ask you to return for visits at 6 scheduled times: at 36 weeks' gestation, and 1 month, 3 months, 6 months, 9 months and 12 months post-delivery. At the 36-week gestation visit, we will perform check-ups on you, ask some questions on your health and psychological wellbeing, and collect some blood. At this same visit, we will do a vaginal swab to test for the types of bacteria that may be present in your reproductive tract. There may be a need for you and your sexual partner(s) to be treated if any inadvertent clinical findings are detected that could have health implications for you and your partner(s) (i.e. STDs). Lastly, on this visit, we will ask to collect a sample of your stool. After your delivery, a member of our team will come to collect blood samples from the cord and placenta, as well as collect a small piece of the placenta for further tests that are meant to provide us with information with regards to the health and well-being of your baby. We will also record Apgar scores, and the baby will have a full physical examination, including measurement of birth weight, length, head circumference, auditory brainstem responses, and details of any congenital malformations. We will also take a small sample of your baby's first stool for analysis. We will ask you to come back at 1 month, 3 months, 6 months, 9 months and 12 months after the birth, when we will check to see how you are feeling, collect blood samples, and measure and weigh your baby. In addition, during the 1-month, 6-month and 12-month post-delivery visits, we will take a small amount of expressed breast milk (2ml) for iron

indices testing and other general well-being and nutritional parameters. We will also collect stool samples from both you and your child for analysis on these visits.

You are also asked to come to the study clinic anytime you are sick, for a check-up and blood test.

Allocating you to a treatment

It is essential that you understand that you will be assigned to one of the treatment arms totally by chance. Before this assignment, neither you nor the study staff will know to which group you will be assigned. You have equal chances of being assigned to either of the treatments described above. Once assigned, you will receive detailed information about the treatment you will receive.

If you are assigned to the intravenous iron intervention arm, you will receive the drug through a small drip inserted into your arm. If you receive oral iron, you will be given a course of tablets from your health worker, plus information on how to take the drug.

What happens after receiving treatment?

After we give you the treatment on that first visit, we will ask you to come to the study clinic at every scheduled study visit for study-specific procedures. Visits are scheduled for 36-weeks gestation, 1-month, 3-month, 6-month, 9-months, and 12-month post-delivery. In addition, we would encourage you to come to the study clinic whenever you are sick. You are also encouraged to go for delivery at the facility that has been allocated to you. You must never share the medicine with your fellow pregnant women or anyone.

Are there any side effects of the treatment?

All medicines have side effects. There is a rare chance that you may experience mild dizziness, joint aches and headache, which usually subsidise without medications. Oral Iron may cause constipation, diarrhoea and stomach aches, and is likely to turn your faeces black – but this is not dangerous. The following table will help you understand the side effects that might happen as well as their frequencies:

Side Effects	How often is it likely to occur?	How severe is it likely to be?	How long might it last?
Stomach aches, nausea, constipation or diarrhoea (from oral iron)	Perhaps up to 1 in 4 people	Mild	A few days of the duration of the treatment; but it is usually tolerable
Infection (e.g. diarrhoea, malaria)	As often as in people not receiving iron, or perhaps slightly more commonly	About as severe as in women not receiving iron, or perhaps slightly more severe.	A few days.

Darkening of stools (from oral iron)	Common	Not harmful at all.	It lasts for the duration of the treatment.
Aches and pains in the joints, headaches (from intervenous iron)	Perhaps 1 in 20 to 1 in 8 people	Mild aches which will go away by themselves	2-3 days
Low blood phosphate levels (from intravenous iron)	Maybe 1 in 10 people	Mild – you will not notice this. It is just a change that occurs in your blood and is not usually harmful. We will test for this condition.	4-6 weeks

What will this study involve for my baby?

If you decide to participate in this study, we will perform all the usual clinical checks on your baby which will include assessing the baby's weight, length, head circumference and examining the baby for any congenital anomalies. We will also collect a small sample of your baby's first stool. In addition, we will check your baby' shearing response to sounds using a machine that measures auditory brain response. This will bed done by placing small soft electrodes that look like headsets into your baby's ears. This assessment does not require any participation by the child. It is best done when the child is sleeping in their mother's arms. A small soft sound will be played, and the baby's response will be recorded using a computer. The sound played will be appropriate for the baby's age and will not be harmful. This will not pose any risk to the child and takes less than 10 minutes to perform.

After 1 month, we will ask you and the baby to return for a check-up, at which point we will weigh and measure the baby again. We will also take a heel-prick or a venous blood test for anaemia and iron deficiency in your baby. On this same visit, we will also collect a stool sample from your child for analysis. In addition, on the 1-month visit, we will also check your baby's hearing using auditory brain response. We will ask you to come back at 3, 6, 9 and 12 months after birth, when we will measure and weigh your baby and collect blood samples. Auditory brain response will also be rechecked on the 3 and 6 months visits. On the 3, 6 and 12 months visits, we will collect stool samples from your child. In addition to the procedures stated above, on the 6 and 12 months visit, will assess your child cognitive development and behavioural response. This involves measuring your child's reaction to sounds, pictures, videos and games using the EEG method and Bayley tests. The EEG method consists in placing a cap on your child's head and playing them a sound or a picture or a video, and then recording how quickly your child's brain responds to the sound, image or video. It will only take a few minutes but provides us with robust information on whether the medicines we gave you in pregnancy have

influenced your child's brain development. The test requires the fitting of the cap and connection of electrodes. Your child will be fitted with a cap(resembling a baby's hat) that measures the brain's response to image and sounds. During testing, the child will be sitting on your lap in a room with a screen and speakers. Your child does not have to do anything but the cap will measure your child's brain response to the images and sounds played. The technique measures brain development by providing a computer image of what is happening in the brain. You will always be present while we conduct these procedures. Pictures and sound played for your child are culturally sensitive and appropriate for his/her age, and your child will enjoy looking at and listening to them. If your child begins to fuss during testing, we will stop the test and resume once the child is feeling better. The technique is completely non-invasive, so it does not require blood or any samples from inside the body. It will not hurt the child. It does not pose any risk to the child and take less than 20 minutes per child. What are the possible risks and disadvantages for my child wearing this cap? There is no risk for your child wearing this cap. The test involves the cap that needs to be worn on the head. The child may experience some discomfort with the cap during testing, but this will not last long. Our experienced testers will ensure complete hygienic precautions and minimum distress.

Additionally, your baby will undergo behavioural child development measurements on the 6 and 12-months visits using Bayley Scales of Infant and Toddler Development III and behavioural test of memory and attention. Bayley tests mainly assess fine and gross motor, cognitive, language and socio-emotional development by evaluating how the child interacts with the environment, i.e. toys, response to sounds and recognising places and persons. This is done by placing toys in front of your baby to check if they recognise and respond to the toy and playing different games with your child and recording how they respond. These tests will not harm your child in any way.

What will happen to my test samples?

You will be asked to provide consent for the collection of blood and other biological samples during the research project. Collection of blood is a required part of involvement in this project. Samples will be analysed for haemoglobin and iron levels in the blood, so we can see if the interventions have worked. We will also store the samples and use them to understand other immune, metabolic and molecular factors that may influence maternal and child health, their response to infections, nutritional status and development. Samples will be stored in such a way that your identity is kept private by use of a code.

Some of the samples we are collecting may also be used for future testing of DNA. We will not be testing for any genetic diseases, and so the results will not have any implications for yours nor your child's nor the rest of your family's health. These tests will not diagnose a genetic disease in the future. Instead, we anticipate testing for genetic differences that may be linked with anaemia, nutrition, development, health and growth, or immune and infection status. Samples and results of genetic testing will not be released to anyone other than the researchers or their colleagues helping with analysis of the samples.

What are the benefits of participating in this study?

If you decide to participate in this study, you will receive all the medical attention usually provided to both mothers and babies, plus you will be encouraged to visit our study site if you get sick during this study. We understand that the excessive travel to the clinics may be a burden and so we will compensate with the equivalent of 10 USD (MK 7000.00) for each visit.

By participating in this study, you will be contributing to identifying a better and effective treatment for moderate and severe anaemia during pregnancy. This can have a significant impact on the way anaemia is managed in Malawi and in the future pregnancy outcomes for women and babies in this region, thus benefiting your community as a whole.

What are the risks of participating in this study?

If you decide to join the study, the risks are minor. If you are enrolled in the intravenous iron arm, a small bruise or mild pain on the arm where the injection is given, and blood is taken may develop. There is also a minimal chance of infection at the site where blood is drawn from. This is almost negligible, however, because we always use sterile materials and our study personnel are highly skilled and trained phlebotomists. We understand the study will require you to make more visits to the hospital than usual. This may be inconvenient, but we will reimburse your transport costs. On each visit to the site, you will compensate you with the equivalent of 10 USD (~MK 7000.00) to cover for your lunch and transport costs.

The risks for your child are also minimal. All procedures (with the exception of the sampling of blood) are non-invasive and will not harm your child. As will happen with you, a small bruise or mild pain on the site where blood is taken may develop.

Privacy and confidentiality concerns

If you consent to take part in the research study, we will keep your name and that of your child and all the information that we get from you as part of this study confidential to the extent that is required by the law. Only members of the study staff and people from the safety committee and government authorities can review the records with your name and that of your child on it. We will use the information you give to us only for research. The information that we collect may be shared with other people in other institutes and countries, but your name and that of your child will not appear on any reports.

Who has reviewed the research project?

The ethical aspects of this research project have been approved by the Malawian Ethics Authorities (the College of Medicine Ethics Committee (COMREC) and or the National Health Sciences Research Committee (NHSRC) at the Ministry of Health) and; Health and Research Ethics Committee (HREC) of the Walter and Eliza Hall Institute of Medical Research, Melbourne, Australia.

Important contacts

Who is organising and funding the research?

This research project is being conducted by Professor Kamija Phiri of the College of Medicine and Dr Sant-Rayn Pasricha of Walter and Eliza Hall Institute of Medical Research. This project is funded by the Bill and Melinda Gates Foundation.

Further information and who to contact

If you want any additional information concerning this project or if the participant has any medical problems which may be related to their involvement in the project (for example, any side effects), you can ask us today or during our regular visits or subsequent visits. If you have other questions later, you can contact: Prof Kamija Phiri, Principal Investigator, College of Medicine, Blantyre, +265999957048 or Dr Sant-Rayn Pasricha, Principal Investigator from Walter and Eliza Hall Institute of Medical Research Tel: +61393452618

For matters relating to research at the site at which the child is participating, the details of the local site complaints person are:

Clinical and Complaints contact person

Name	*Zinenani Truwah*
Position	*Study Coordinator, Training and Research Unit of Excellence*
Telephone	*+265999413775/+2650882091578*
Email	*zinenanitruwah@gmail.com*

If you have any complaints about any aspect of the project, the way it is being conducted or any questions about being a research participant in general, then you may contact:

Reviewing HREC name	**National Health Sciences Research Committee**
HREC Contact Person:	*Dr. Collins Mitambo*
Telephone	*+265999397913*
Email	*cmitambo@gmail.com*

<u>Declaration by participant/ Guardian</u>

- I have read the consent, or someone has read it to me in Chichewa.
- I understand the purposes, procedures and risks of the research described in the project.
- I give permission for my doctors and my child's doctors, as well as other health professionals, hospitals or laboratories outside this hospital to release information to the College of Medicine and the Walter and Eliza Hall Institute of Medical Research

concerning mine and my child's health and treatment for the purposes of this project. I understand that such information will remain confidential.

- ➢ I have had the opportunity to ask questions, and I am satisfied with the answers I have received.
- ➢ I freely agree to participate and to have my child participating in this research project as described and I understand that I am free to withdraw my participation and my child's participation at any time during the research project without affecting mine or my child's future health care.
- ➢ I understand that I will be given a signed copy of this document to keep.

____ ____________________

Signature or left thumb impression of Participant/ Guardian/ Attendant — Date

____ ____________________

Signature or left thumb impression of the witness (only when the participant is illiterate) — Date

____ ____________________

Signature of the PI or his/her representative — Date

24.3. MATERIAL TRANSFER AGREEMENT

NATIONAL HEALTH SCIENCES RESEARCH COMMITTEE

MATERIAL TRANSFER AGREEMENT (MTA) FORM

Protocol Number:

Title of protocol:

Randomized controlled trial of the Effect of intraVenous iron on Anaemia in Malawian Pregnant women – Third Trimester (REVAMP-TT Study)

Intention and Justification of transfer:

Blood, placental tissue, vaginal swabs and stool samples will be shipped to the Walter and Eliza Hall Institute of Medical Research, in Melbourne, Australia where one of the Study Principal Investigators is based for advanced tests that are currently not available in Malawi. Blood samples include whole blood as well as cellular, serum and plasma fractions.

All samples are required to in order to conduct the immune, metabolic and molecular laboratory analyses which will be required to derive some of the study's secondary outcomes, as clearly stated in the protocol.

Duration of storage:

All exported samples will be stored for a period of not more than 5 years and any remaining samples will be destroyed soon after the laboratory analyses have been completed.

Responsible Party:

- Prof Kamija Phiri, Department of Public Health, College of Medicine, University of Malawi, Private Bag 360, Chichiri, Blantyre 3, Malawi. Mobile +265 999957 048; E-mail: kphiri@medcol.mw
- Dr Sant-Rayn Pasricha, Walter and Eliza Hall Institute of Medical Research, 1G Royal Parade, Parkville, Melbourne 3052, Australia. Mobile: +61407141570; E-mail: Pasricha.s@wehi.edu.au

Location of stored samples:

- In Malawi prior to exporting the samples will be stored at the College of Medicine, University of Malawi, Private Bag 360, Chichiri, Blantyre 3, Malawi. Mobile +265 999957 048
- In Australia samples will be stored at Walter and Eliza Hall Institute of Medical Research, 1G Royal Parade, Parkville, Melbourne 3052, Australia. Mobile: +61407141570

Transportation of samples:

Packaging and transportation of the samples will be as per requirements by the IATA guidelines and regulations on shipment of biological materials.

Ownership of samples:

The Government of Malawi will maintain ownership of all samples through the College of Medicine, University of Malawi.

After all laboratory testing has been completed: Describe what will happen to the samples

As stated in this Material Agreement Transfer form, all samples will be kept for a maximum of 10 years after which the samples will be destroyed.

**Appropriate informed consent authorising the exportation and importation of samples**
The trial's Informed Consent Form clearly seeks authorization from the participants with regards to the export of all necessary samples, as well as provides information concerning the reasons for that sample export as well as contact numbers that can be reached if more information is required.
**To whom will the samples be accessible**
The samples will only be accessible to assigned laboratory staff at the College of Medicine, University of Malawi (during preparation and transfer) and the laboratory staff directly involved in sample analyses for the Clinical Trial at the Walter and Eliza Hall Institute of Medical Research, in Melbourne, Australia. In case of any analyses that might require a third party involvement, this will only be done with the full agreement of the trial's Principal Investigators.
**Who will be the controlling officers of the samples**
**Dr Sant-Rayn Pasricha**, Walter and Eliza Hall Institute of Medical Research, 1G Royal Parade, Parkville, Melbourne 3052, Australia. Mobile: +61407141570; E-mail: Pasricha.s@wehi.edu.au

Samples collected in Malawi may not be sold without prior permission from the collaborating or controlling institutions and the NHSRC in Malawi.

Signed by

**Name of the PI:**	**Name of Co – PI**
Prof. Kamija Phiri	Dr. Sant-Rayn Pasricha
**Name of Institution:**	**Name of Institution:**

College of Medicine, University of Malawi

Signature:

Date Signed:

WEHI, Melbourne, Australia

Signature: [redacted]

Date Signed: 30/10/2020

NHSRC APPROVAL

Name of the Chairperson:

Signature:

Date Signed:

Name of Secretary

Signature:

Date Signed:

NHSRC STAMP OF APPROVAL:

Executive Committee: *Dr B. Chilima (Chairperson), Dr Bagrey Ngwira (Vice-Chairperson)*
Registered with the USA Office for Human Research Protections (OHRP) as an International RB
IRB Number IRB00003905 FWA00005976
Email: ***mohdoccentre@nhsrc-mw.com***

REVAMP-TT Protocol version 3.1

Protocol used at trial close

Training & Research Unit of Excellence
(TRUE)

To Dr. M. Joshua
Chairperson NHSRC
Ministry of Health
P.O. Box 30377
Lilongwe 3 Malawi
Telephone: + 265 726 422
Facsimile: + 265 726 418
Email: ***mohdoccentre@gmail.com***

January 30, 2023

OUR REF: NHSRC: 20/11/2622

Dear Sir,

RE: Application for an amendment to the protocol entitled "Randomized controlled trial ofthe Effect of intravenous iron on Anaemia in Malawian Pregnant women – Third Trimester (REVAMP-TT Study)" – NHSRC NO: 20/11/2622

Thank you for reviewing our application for an amendment of the REVAMP-TT Study protocol version 3.0 dated 2nd November, 2022. We received the feedback dated 6th December, 2022 that *"the committee reviewed and did not approve the proposed amendment as we did not provide the updated budget for the increase in sample size"*.

Initially, we did not provide the updated budget as funds could only be given to us if the sample size re-evaluation supported our proposal to increase the sample size. However, the chairperson of the study DSMB recommended maintaining the current sample size. The recommendation was based on the findings of the sample size re-evaluation as below.

We have submitted version 3.1 with track changes for review and subsequent approval by NHSRC. We have submitted the following documents:

1. Cover letter
2. NHSRC Amendment form 10-01
3. Summary of proposed changes to REVAMP-TT study
4. REVAMP TT study protocol with tracked changes

We request that the NHSRC secretariat stamps the approved and signed study documents with the NHSRC stamp so that the study monitors can verify that these are the approved versions. We look forward to a favorable response from your office.

Thank you.

Yours truly,

[redacted]

Prof Kamija Phiri Principal Investigator

Professor David Lissauer, Professor Peter J Diggle and Professor Michael J Dibley
The REVAMP-TT DSMB members

12th January 2023

Prof Kamija Phiri
Department of Public Health, School of Global and Public Health
Kamuzu University of Health Sciences
Training and Research Unit of Excellence (TRUE)
Blantyre, Malawi

Prof Sant-Rayn Pasricha
Population Health and Immunity Division
Walter and Eliza Hall Institute of Medical Research
The University of Melbourne, Melbourne, Australia

Dear Sirs,

The sample size re-evaluation of REVAMP-TT was conducted on 11th January 2023. A report outlining the statstical details supporting our recommendation was provided to us by the independent unblinded statistician Dr Anurika De Silva.

Please find below the outcome of the sample size re-evaluation conducted as detailed in the interim statistical plan (document version date 09 January 2023).

The recommendation is for the trial to continue as planned and recruit the planned sample size of 590, which accounts for 10% attrition.

Yours sincerely,

[redacted]

Professor David Lissauer, DSMB chair
On behalf of all DSMB members

NHSRC FORM 10-01

REQUEST FOR AMENDMENT/MODIFICATION

Please complete the following:

**NHSRC REF. Number: 20/11/2622** (NHSRC will not process requests without this number.)	**Date of Request:**
**Principal Investigator Name:** **Professor Kamija Phiri,** Kamuzu University of Health Sciences (KUHES) Malawi and Training and Research Unit of Excellence (TRUE), P.O Box 30538, Chichiri, Blantyre 3, Malawi; Mobile: +265 999957 048. E-mail: director@true.mw	**Contact Person** (if other than PI) **Ernest Moya, MPH, PhD Student**, Kamuzu University of Health Sciences (KUHES) Malawi and Training and Research Unit of Excellence (TRUE), P.O Box 30538, Chichiri, Blantyre 3, Malawi; Mobile: +265 999 639 917; E-mail: moyaernest@gmail.com
**Title of Study** **A randomised controlled trial of the effect of intravenous iron on anaemia in Malawian pregnant women in their third trimester (REVAMP – TT)**	

1. **Description of proposed changes:** (Note: ***Changes will not be implemented before NHSRC approval. Attach the original document with highlighted changes/modifications***)
   - We have described the proposed changes to the protocol.
   - See attached documents namely:
     - Table of changes by section (summary of proposed changes)
     - Edited protocol with track changes (and a clean version as well)

   Use attachments and additional pages, as needed.

2. **Reason for Amendment/Modification:**
   - Addition of interim analyses for adaptive sample size recalculation (including results of analysis).
   - Refine the definition of the primary outcome.
   - Addition of continuous birth weight and birth length outcomes
   - Addition of time point 1 month to infant hemoglobin, inflammation and iron outcomes.

Executive Committee: *Dr M. Joshua (Chairperson), Dr E. Chitsa Banda (Vice-Chairperson)*
Registered with the USA Office for Human Research Protections (OHRP) as an International IRB
IRB Number IRB00003905 FWA00005976
Email: ***mohdoccentre@gmail.com***

3. Changes to Consent Form: Are changes required? **No** ________ Yes _______ (If Yes, attach new consent form and highlight the changes)

[redacted]
Signature of Principal Investigator Date: 30th January, 2023

NHSRC Office Use only:
Approval date:______________________
Approved by:

Approval of Changes /Modifications by NHSRC

Recommended :__________
Not recommended :_______
Full committee Review_______

Signature **NHSRC Chairperson or Authorised Signatory** **Date**

Executive Committee: *Dr M. Joshua (Chairperson), Dr E. Chitsa Banda (Vice-Chairperson)*
Registered with the USA Office for Human Research Protections (OHRP) as an International IRB
IRB Number IRB00003905 FWA00005976
Email: ***mohdoccentre@gmail.com***

SUMMARY OF PROPOSED CHANGES TO REVAMP-TT PROTOCOL VERSION 2.1 to VERSION 3.0

1. Summary of main adjustments to protocol by section:

Section	Version 2.1 text	Changes made for Version 3.0	Reason	Main justification for protocol amendment
Executive summary: Methods	< add a new sentence after the sentence "The study has … (49% vs 63%).>	A sample size re-estimation was conducted while the trial was ongoing and confirmed the planned number of 590 randomised participants.	Addition of interim analyses for adaptive sample size recalculation	The REVAMP trial, which compared FCM to standard of care to women with moderate or severe anaemia in the second trimester of pregnancy, was unblinded in April 2022. This trial showed a mean difference of 4.3% (52.5% vs 56.8%) at the primary endpoint of anaemia at 36 weeks' gestation, and a mean difference of 7.7% (76.7% vs 84.4%) for anaemia at 4 weeks post infusion. There as a substantial reduction in iron deficiency anaemia (prevalence ratio 0.30 (0.20, 0.44). The REVAMP trial was conducted in the same setting as REVAMP-TT and provides important new external information on the assumed treatment effect underlying the ongoing REVAMP-TT trial. Our initial analyses indicated a higher prevalence of baseline iron deficiency in the REVAMP-TT cohort, likely because it is being undertaken in the third, rather than second trimester, and also because all participants have received IPTp previously. For all these reasons, we feel it is appropriate to undertake a sample size re-estimation based on a conditional power calculation based on the promising zone methodology, as outlined, which will ensure the participation of study participants is of
Section 16.1. Sample Size Calculations	< add a new paragraph at the end>	Whilst the pre-planned sample size is 590 participants, an adaptive sample size re-estimation procedure will be undertaken as per the 'promising zone' methodology of Mehta and Pocock with a potential increase of up to 260 participants to the prespecified maximum of 850 participants total (including loss to follow up) once the outcomes of at least 50% of the recruited participants are obtained (Mehta and Pocock, 2011, p. 3267-84). Details will be outlined in a stand-alone interim statistical analysis plan. In short, this procedure will involve evaluation of the conditional power during an interim analysis conducted by an independent unblinded statistician. If the conditional power falls in a pre-specified 'promising zone', then the sample size may be increased with the aim to achieve a conditional power of 90%.	Addition of interim analyses for adaptive sample size recalculation	

		Otherwise, if the conditional power does not fall within this 'promising zone', then the planned sample size of 590 participants will be maintained. There will be no impact on the Type I error rate and thus no adjustment will be needed to the two-sided significance level of 5%.		maximal value. The sample size re-estimation was conducted on January 11, 2023 while the trial was ongoing and confirmed the planned number of 590 randomised participants.
Executive summary: Primary outcome<br><br>All locations throughout the document where the primary outcome is mentioned.	Proportion of women with pre-delivery anaemia (defined as venous Hb <11.0 g/dl at 36 weeks' gestation	Proportion of women with anaemia (defined as venous Hb <11.0 g/dl at 36 weeks' gestation or at delivery, whichever comes first	Clarification on the definition of the timepoint for assessment of the primary outcome	The REVAMP trial demonstrated that a significant proportion of women delivered their babies before the defined visit for the primary outcome (visit 4 - week 36). This resulted in loss of data at the primary endpoint and a decrease in the statistical power. We would like to clarify our timepoint of primary outcome assessment to avoid this.

2. Minor editing changes/corrections have also been made to the protocol and have been highlighted (or struck out) in the attached documents.

A randomised controlled trial of the effect of intravenous iron on anaemia in Malawian pregnant women in their third trimester

Short Title: REVAMP-TT

Study Identifiers:		
NHSRC: **20/11/2622**	PMRA: PMRA/CTRC/III/08062021130	ANZCTR 12621001239853

Co-Principal Investigators:

- Professor Kamija Phiri, Kamuzu University of Health Sciences (KUHES) Malawi and Training and Research Unit of Excellence (TRUE), P.O Box 30538, Chichiri,Blantyre 3, Malawi; Mobile: +265 999957 048; E-mail: director@true.mw
- Associate Professor Sant-Rayn Pasricha, Walter and Eliza Hall Institute of Medical Research, 1G Royal Parade, Parkville Victoria 3052, Australia; Mobile: +61 407 141 570; E-mail: pasricha.s@wehi.edu.au

Co-Investigators: See Page 8

Funder: Bill & Melinda Gates Foundation (BMGF)

Revision chronology:

Date	Protocol Version	Details ofChanges	Authors (see pg 8)	Signature Principal Investigators
29 Aug 2020	*1.0 Original*		*SP, KP, RA, MM, LL, SB*	
26th Mar 2021	*1.1*	• Trial arms reduced to two after themanufacturer of IIM could not supply the IP<br>• Co-investigator list revised to add Dr RebeccaHarding		
16th Jan 2022	*2.0*	• Added measures of the proportion of women with hypophosphatemia (clinical and biochemical) at various timepoints<br>• Added assessment of child neurodevelopment scores measured by low field magnetic resonance imaging (MRI)<br>• Added assessment of the proportion of infants with radiological Rickets at 3 and 12 months of age.<br>• See table of summaries by section		

	2.1	• Budget amendment<br>• MTA form amendment<br>• Amendment of ICF		
1th Nov 2022	*3.0*	• Added provision for and results of interim analysis for sample size recalculation<br>• Primary oucome definition was clarified<br>• Addition of time point 1 month to infant hemoglobin, inflammation and iron outcomes	*SP, KP*	
30th Jan 2022	*3.1*	• Withdrawing the proposal to increase the sample size to 850 based on sample size re-evalution and DSMB recommendation	*SP, KP*	

Confidentiality Statement: This document contains confidential information that must not be disclosed to anyone other than the sponsor, the investigator team, host institution, relevant ethics committee and regulatory authorities

Table of Contents

1. ABBREVIATIONS 7

1. ABBREVIATIONS 7

2. TITLE OF RESEARCH PROPOSAL 8

3. INVESTIGATORS AND INSTITUTIONAL AFFILIATIONS 8

4. PROTOCOL SUMMARIES 9

4.1. TRIAL REGISTRATION DATA 9
4.2. EXECUTIVE SUMMARY 13
4.3. SCHEDULE OF ACTIVITIES 16

5. INTRODUCTION 20

5.1. BACKGROUND 20
5.1.1. *ANAEMIA DURING PREGNANCY REMAINS A CRITICAL GLOBAL HEALTH PROBLEM* 20
5.1.2. *LOW BIRTH WEIGHT AND PRE-TERM BIRTH HAVE CRITICAL IMPLICATIONS FOR MOTHER AND BABY* 20
5.1.3. *IRON CAN BENEFIT ANTENATAL ANAEMIA* 20
5.1.4. *UPTAKE AND ADHERENCE TO ORAL IRON THERAPY DURING PREGNANCY IS INADEQUATE IN THE FIELD* 21
5.1.5. *FERRIC CARBOXYMALTOSE: A NEW INTRAVENOUS IRON PREPARATIONS* 21
5.1.6. *INTRAVENOUS IRON AND BENEFITS FOR THE POSTPARTUM PERIOD* 30

6. TRIAL OBJECTIVES AND OUTCOMES 31

6.1. OBJECTIVES 31
6.1.1. *BROAD OBJECTIVE* 31
6.1.2. *SPECIFIC OBJECTIVES* 31
6.2. OUTCOMES 31
6.2.1. *PRIMARY OUTCOME* 31
6.2.2. *SECONDARY OUTCOME (MATERNAL BENEFITS)* 32
6.2.3. *SECONDARY OUTCOMES (NEONATE/INFANT BENEFITS)* 32
6.2.4. *SECONDARY OUTCOME (MATERNAL SAFETY)* 32
6.2.5. *SECONDARY OUTCOME (CHILD SAFETY)* 33

7. STUDY DESIGN 33

8. PARTICIPANT INCLUSION AND EXCLUSION CRITERIA 33

9. STUDY SITES 34

10. TRIAL INTERVENTIONS 36

10.1. ALLOCATION TO TREATMENT 36
10.2. BREAKING THE BLIND 36
10.2.1. PARTICIPANT LEVEL 36
10.2.2. STUDY LEVEL 36

11. STUDY PROCEDURES 36

11.1. SCREENING – VISIT 0 [DAY 0] 37
11.1.1. BLOOD SAMPLING 38
11.1.2. STUDY ARM ALLOCATION 38
11.1.3. STUDY DRUG ADMINISTRATION 38
11.1.4. SOCIO-ECONOMIC DATA COLLECTION 39
11.1.5. OTHER PROCEDURES 39
11.2. 36-WEEK FOLLOW-UP [± 2 DAYS] – VISIT 4 39
11.2.1. BLOOD SAMPLING 39
11.2.2. REPRODUCTIVE TRACT MICROBIOTA 39
11.2.3. ORAL IRON ARM - COMPLIANCE 40
11.2.4. MATERNAL PSYCHOLOGICAL HEALTH 40
11.3. DELIVERY [+1 DAY] – VISIT 7 40
11.3.1. BLOOD SAMPLING 40
11.3.2. PLACENTAL TISSUE - HISTOLOGY AND STORAGE 40
11.3.3. INFANT NEURODEVELOPMENT 41
11.4. 28 DAYS POSTPARTUM [± 2 DAYS] – VISIT 8 41
11.4.1. BLOOD SAMPLING 41
11.4.2. REPRODUCTIVE TRACT MICROBIOTA 41
11.4.3. BREASTMILK 41
11.4.4. CHILD ANTHROPOMETRY 42
11.4.5. CHILD VACCINATION AND VITAMIN A SUPPLEMENTATION STATUS 42
11.4.6. INFANT NEURODEVELOPMENT 42
11.5. 3 MONTHS POSTPARTUM [± 14 DAYS] – VISIT 9 42
11.5.1. BLOOD SAMPLING 42
11.5.2. CHILD ANTHROPOMETRY 42
11.5.3. CHILD VACCINATION AND VITAMIN A SUPPLEMENTATION STATUS 42
11.5.4. MATERNAL PSYCHOLOGICAL HEALTH 43
11.6. 6 MONTHS POSTPARTUM [± 14 DAYS] – VISIT 10 43
11.6.1. BLOOD SAMPLING 43
11.6.2. BREASTMILK 43
11.6.3. CHILD ANTHROPOMETRY 43
11.6.4. CHILD VACCINATION AND VITAMIN A SUPPLEMENTATION STATUS 43
11.6.5. INFANT NEURODEVELOPMENT 43
11.7. 9 MONTHS POSTPARTUM [± 14 DAYS] – VISIT 11 44
11.7.1. BLOOD SAMPLING 44
11.7.2. CHILD ANTHROPOMETRY 44
11.7.3. CHILD VACCINATION AND VITAMIN A SUPPLEMENTATION STATUS 44
11.8. 12 MONTHS POSTPARTUM [± 14 DAYS] – VISIT 12 44
11.8.1. BLOOD SAMPLING 44
11.8.2. REPRODUCTIVE TRACT MICROBIOTA 45
11.8.3. BREASTMILK 45
11.8.4. CHILD ANTHROPOMETRY 45

11.8.5. CHILD VACCINATION AND VITAMIN A SUPPLEMENTATION STATUS 45
11.8.6. INFANT NEURODEVELOPMENT 45
11.9. UNSCHEDULED SICK VISIT [ANYTIME DURING STUDY FOLLOW-UP] 46

12. LABORATORY PROCEDURES 46

12.1. FULL BLOOD COUNT 46
12.2. MALARIA TESTING 46
12.3. PLACENTAL HISTOLOGY 46
12.3.1. MALARIA EVALUATION 47
12.4. BLOOD SAMPLES: SEPARATION AND LONG-TERM STORAGE 47
12.5. VAGINAL AND GUT MICROBIOME ANALYSIS 47
12.6. SUMMARY OF LABORATORY MEASUREMENTS 48
12.7. NEUROCOGNITIVE ASSESSMENTS USED IN INFANT FOLLOW UP 51
12.7.1. PROCEDURES FOR ERPS 51

13. ALLOCATION OF PARTICIPANTS TO TRIAL ARMS 52

14. ASSESSMENT OF SAFETY 53

14.1. PRECAUTIONS IN DELIVERING IRON INTRAVENOUS FORMULATIONS 53
14.2. DEFINITIONS OF ADVERSE EVENTS 55
14.3. RECORDING OF ADVERSE EVENTS 55
14.3.1. THE TIME PERIOD FOR COLLECTING ADVERSE EVENTS 55
14.3.2. METHOD OF CAPTURING ADVERSE EVENTS 55
14.3.3. DOCUMENTATION OF ADVERSE EVENTS DATA 55
14.3.4. ASSESSMENT OF ADVERSE EVENT SEVERITY 56
14.3.5. ASSESSMENT OF CAUSALITY OF ADVERSE EVENTS 56
14.3.6. STUDY ENDPOINTS AND SYMPTOMS ANAEMIA 56
14.3.7. HIV-RELATED DISEASE 56
14.3.8. LACK OF EFFICACY AND DISEASE PROGRESSION 56
14.3.9. ABNORMAL LABORATORY VALUES 56
14.3.10. OVERDOSE 57
14.4. REPORTING OF SERIOUS AES 57
14.4.1. REPORTING BY THE INVESTIGATOR TO THE STUDY SAFETY MONITOR AND SPONSOR 57
14.4.2. REPORTING BY THE SPONSOR 57
14.5. DATA MONITORING COMMITTEE 57

15. DATA HANDLING AND RECORD KEEPING 58

15.1. CASE REPORT FORMS (CRFS) 58
15.2. DATA ENTRY AND VALIDATION 58
15.3. DATABASE LOCK 59

16. STATISTICAL CONSIDERATIONS 59

16.1. SAMPLE SIZE CALCULATIONS 59

DATA ANALYSIS 59
16.1.1. ASSESSMENT OF EFFECTIVENESS 59
16.1.2. ANALYSIS OF ADVERSE EVENTS 60
16.2. ANALYSIS POPULATIONS 60
16.3. MISSING DATA 60
16.4. INTERIM ANALYSES AND CRITERIA FOR TERMINATION OF THE TRIAL 60

17. STUDY MANAGEMENT 60

17.1. STUDY MONITORING 60
17.2. DIRECT ACCESS TO SOURCE DATA/DOCUMENTS 61
17.3. QUALITY ASSURANCE 61
17.4. TRAINING OF STAFF 61
17.5. CHANGES TO THE PROTOCOL 61
17.6. FINANCING AND INSURANCE 62
17.7. STUDY DURATION 62
17.8. RECORD-KEEPING AND ARCHIVING 62
17.9. REPORTING AND PUBLICATION OF DATA 62

18. ETHICAL CONSIDERATIONS 63

18.1. ETHICAL REVIEW 63
18.2. ETHICAL CONDUCT OF THE STUDY 63
18.3. INFORMED CONSENT 63
18.4. RISKS TO THE STUDY PARTICIPANTS 64
18.4.1. BLOOD SAMPLING 64
18.4.2. INTRAVENOUS INFUSIONS 64
18.5. BENEFITS FROM PARTICIPATING IN THE TRIAL 64
18.6. SUBJECT DATA PROTECTION 64
18.7. OTHER ETHICAL CONSIDERATIONS 65
18.7.1. REIMBURSEMENT OF COSTS 65

19. DISSEMINATION OF RESULTS 65

20. CAPACITY BUILDING 65

21. BUDGET 67

22. BUDGET JUSTIFICATION 69

23. BIBLIOGRAPHY 70

24. APPENDIX 76

1. Abbreviations

AE	Adverse Event
ANC	Antenatal Care
CoM	College of Medicine
CRF	Case Report Form
CRP	C-Reactive Protein
DASS-21	Depression, Anxiety and Stress Status (short form)
dL	Deciliter
DMC	Data Monitoring Committee
FCM	Ferric Carboxymaltose
ID	Iron deficiency
IDA	Iron deficiency anaemia
g	Grams
Hb	Haemoglobin
HC	Health Centre
HIV	Human Immunodeficiency Virus
ICTRP	International Clinical Trials Registry Platform
IPTp	Intermittent Preventive Treatment in pregnancy
ITN	Insecticide-treated net
IV	Intravenous
Kg	Kilograms
L	Litres
LMICs	Low and Medium-Income Countries
LRTI	Lower Respiratory Tract Infection
MCATS	Melbourne Clinical and Translational Science Platform
MRI	Magnetic Resonance Imaging
OR	Odd Ratio
PCR	Polymerase Chain Reaction
RCT	Randomised Controlled Trial
RDT	Rapid Diagnostic Test
SC	Sickle Cell
SHC	Sickle Haemoglobin Cell
SP	Sulfadoxine-Pyrimethamine
TfR	Transferrin Receptor
UK	United Kingdom
USA	United States of America
WHO	World Health Organisation
ZCH	Zomba Central Hospital

2. TITLE OF RESEARCH PROPOSAL

A randomised controlled trial of the effect of intravenous iron on anaemia in Malawian pregnant women- third-trimester study – **REVAMP-TT**

3. INVESTIGATORS AND INSTITUTIONAL AFFILIATIONS

Co-Principal Investigators:

- A/Prof Sant-Rayn Pasricha, Walter and Eliza Hall Institute of Medical Research, Australia
- Professor Kamija Phiri, Training and Research Unit of Excellence (TRUE), College of Medicine, Kamuzu University of Health Sciences (KUHES), Blantyre, Malawi.

Co-Investigators:

- Dr Martin Mwangi, Training and Research Unit of Excellence (TRUE), College of Medicine, Kamuzu University of Health Sciences (KUHES), Blantyre, Malawi.
- Professor Stephen Rogerson, University of Melbourne, Australia
- Professor Julie Simpson, University of Melbourne, Australia
- Professor William Stones, Training and Research Unit of Excellence (TRUE), College of Medicine, Kamuzu University of Health Sciences (KUHES), Blantyre, Malawi.
- Professor Beverley-Ann Biggs, University of Melbourne, Australia
- Professor Jane Fisher, Monash University
- Ms Sabine Braat, University of Melbourne, Australia
- Dr Leila Larson, University of South Carolina, United States of America
- A/Prof Stefan Bode, University of Melbourne, Australia
- A/Prof Katherine Johnson, University of Melbourne, Australia
- Dr Ricardo Ataide, University of Melbourne, Australia
- Dr Rebecca Harding, Walter and Eliza Hall Institute of Medical Research, Australia
- Dr Louise Randall, Walter and Eliza Hall Institute of Medical Research, Australia
- A/Prof Marc Seal, Murdoch Children's Research Institute and University of Melbourne, Australia
- Dr Peter Simm, Murdoch Children's Research Institute, Australia
- Ernest Moya, Training and Research Unit of Excellence (TRUE), Kamuzu University of Health Sciences (KUHES), Blantyre, Malawi.
- Glory Mzembe, MD, Training and Research Unit of Excellence (TRUE) Kamuzu University of Health Sciences (KUHES), Blantyre,

4. Protocol Summaries

4.1. Trial registration data

Data Category	*Information*
Primary registry and trial identifying number	ANZCTR Trial number ACTRN12621001239853
Date of registration in primary registry	14 September 2020
Secondary identifying numbers	NHSRC: 20/11/2622 **PMRA:** PMRA/CTRC/III/08062021130 **ANZCTR:** 12621001239853
Source(s) of monetary or material support	Bill and Melinda Gates Foundation (BMGF)
Primary sponsor	Training and Research Unit of Excellence (TRUE), Malawi.
Secondary sponsor(s)	NA
Contact for public queries	Associate Prof Sant-Rayn Pasricha, E-mail: pasricha.s@wehi.edu.au
Contact for scientific queries	Associate Prof Sant-Rayn Pasricha, Walter and Eliza Hall Institute of Medical Research, 1G Royal Parade, Parkville Victoria 3052, Australia; Mobile: +61 407 141 570; E-mail: pasricha.s@wehi.edu.au
Public title	A randomised controlled trial of the effectiveness of intravenous iron on anaemia in Malawian pregnant women in their third trimester
Scientific title	Effectiveness of intravenous iron administered during the third trimester in Malawian pregnant women in the management of anaemia: A 4-year, multi-centre, parallel-group, two-arm open-label randomized controlled superiority trial
Countries of recruitment	Malawi
Health condition(s) or problem(s) studied	Anaemia, iron deficiency
Intervention(s)	intravenous ferric carboxymaltose (1000 mg for body weight ≥50 kg, or 20 mg/kg for body weight <50 kg) once during the third trimester
	Standard of care: Oral iron 200 mg ferrous sulphate (approx. 65 mg elemental iron) twice daily for 90 days or the duration of pregnancy, whichever is shorter
Study type	Interventional
	Allocation: randomised; intervention model: parallel assignment; arms:2~~3~~; allocation ratio: ~~1:~~1:1; Masking: open-label
	Primary purpose: treatment
	Phase-III

Date of first enrolment	24 November 2021
Target sample size	590
Recruitment status	Recruiting
Key inclusion criteria	• Confirmed singleton pregnancy in the third trimester (27-35 weeks of gestation, dated by Last Menstrual Period and/or Fundal Height)<br>• Moderate to severe anaemia not requiring an immediate blood transfusion (Hb <10 g/dl)<br>• Negative malaria parasitaemia by mRDT<br>• Currently afebrile with no evidence of septicaemia<br>• Resident in the study catchment area of Zomba district<br>• Able to deliver at health facilities within Zomba district<br>• Written informed consent (including assent if <18 years old)
Exclusion criteria (at enrolment)	• Previous enrolment in REVAMP trial (COMREC P.02/18/2357)<br>• Actively participating in another intervention trial<br>• Known hypersensitivity to the study drugs<br>• Clinical symptoms of malaria or other infection (no fever, no focal symptoms of internal infection i.e. LRTI/ diarrhoea)<br>• Any condition requiring hospitalisation in the next seven days or serious concomitant illness<br>• Known history of sickle cell or sickle-haemoglobin C anaemia<br>• Clinically low haemoglobin level requiring a blood transfusion (usually Hb <5 g/dl)<br>• Pre-eclampsia
Primary outcome(s)	Proportion of women with anaemia (defined as venous Hb <11.0 g/dl at 36 weeks' gestation or at delivery, whichever comes first)
Key Secondary efficacy outcomes	• Mean change from baseline in maternal Hb at 36 weeks' gestation or at delivery, whichever comes first.<br>• Proportion of women with maternal iron deficiency (ferritin<15 mg/L, sTfR/Ferritin index) at 36 weeks' gestation or at delivery, whichever comes first..<br>• Mean levels of maternal iron biomarkers at 36 weeks' gestation or at delivery, whichever comes first..<br>• Proportion of women with maternal inflammation (using C-reactive protein) at 36 weeks' gestation or at delivery, whichever comes first.<br>• Proportion of women with maternal postpartum haemorrhage.<br>• Mean change from baseline in maternal Hb at 1, 3, 6, 9 and 12 months postpartum.

	• Proportion of women with maternal anaemia at 1, 3, 6, 9 and 12 months postpartum<br>• Mean levels of maternal iron biomarkers at 1, 3, 6, 9 and 12 months postpartum<br>• Proportion of women with iron deficiency (defined by ferritin<15mg/L) at 1, 3, 6, 9 and 12 months postpartum<br>• Proportion of women with postpartum depression (defined by EDPS > 13) at 3 months postpartum.<br>• Proportion of women with maternal inflammation (defined by C-reactive protein) at 3 months postpartum.<br>• Mean gestation duration (in weeks).<br>• Mean birth weight and birth length (in grams and centimetres, respectively, within 24 hours of delivery).<br>• Proportion of sub-optimal pregnancy outcomes (defined as a composite outcome: low birth weight (<2500 g); prematurity (birth <37 weeks); small for gestational age (centile score) as defined by international reference standards for gestational age-specific birth weight; stillbirth).<br>• Proportion of neonates born prematurely (defined as birth before 37 week's gestation)<br>• Proportion of infants with low birth weight (defined as a birth weight <2500 g).<br>• Proportion of stillbirth.<br>• Proportion of neonatal mortality.<br>• Mean cord blood Hb and ferritin.<br>• Proportion of neonates with anaemia (with correction for gestational age).<br>• Child development scores measured by Evoked Response Potentials (ERP) at 6 and 12 months of age.<br>• Child development scores measured by the Malawi Developmental Assessment Tool (MDAT) and Bayley Scales of Infant Development at 6 and 12 months of age.<br>• Child neurodevelopment scores measured by low field magnetic resonance imaging (MRI) at 3 and 12 months of age.<br>• Mean child physical growth as defined by z-scores at 1, 6 and 12 months of age.<br>• Mean infant haemoglobin (capillary) at 1-, 6- and 12-months postpartum.<br>• Proportion of infants with anaemia at 1-, 6- and 12-months postpartum.<br>• Mean levels of infant iron biomarkers at 1-, 6- and 12-months postpartum.<br>• Proportion of infants with iron deficiency at 1-, 6- and 12-months postpartum

Safety outcomes	• Proportion of women with at least one treatment related adverse effects (occurring immediately post-infusion, and within 7 days of commencement of treatment in the intravenous groups).<br>• Incidence of treatment related adverse effects (occurring immediately post-infusion, and within 7 days of commencement of treatment in the intravenous groups).<br>• Number of unplanned visits to the clinic (cause specific for diarrhea and clinical malaria).<br>• Incidence of all-cause maternal sick clinic visits during the antenatal, postpartum and overall participant follow-up period.<br>• Incidence of maternal cause-specific sick clinic visits (in particular malaria, diarrhoea and other infectious conditions) during the antenatal, postpartum and overall participant follow-up period<br>• Proportion of women with placental malaria (past or active infection on histology, parasites on placental blood film).<br>• Proportion of women with malaria parasitaemia (asymptomatic) at 36 weeks' gestation (or at delivery, whichever comes first), detected by a) microscopy, b) rapid diagnostic tests, and c) PCR.<br>• Proportion of women with bacteraemia at 36 weeks' gestation or at delivery, whichever comes first.<br>• Proportion of women with hypophosphatemia (clinical and biochemical) at baseline, 36 weeks' gestation, delivery, 28 days postpartum, 3 months postpartum, 6 months postpartum and 12 months postpartum.<br>• Proportion of infants with hypophosphataemia (biochemical) at 6 weeks and at 6 months of age.<br>• Number of unplanned infant visits to the clinic (cause specific for diarrhea and clinical malaria).<br>• Incidence of all-cause sick clinic visits in infant by 12 months of age.<br>• Incidence of cause-specific sick clinic visits (in particular malaria, diarrhoea and other infectious conditions) ininfant by 12 months of age.<br>• Proportion of infants with malaria parasitaemia (asymptomatic) 1, 3, 6, 9 and 12 months postpartum detected by a) microscopy, b) rapid diagnostic tests, and c) PCR<br>• Proportion of infants with radiological Rickets at 3 months and 12 months of age.

4.2. EXECUTIVE SUMMARY

Type of study: Open-label two-arm parallel-group individual-randomised controlled trial

Problem: Anaemia in pregnancy remains a critical global health problem, affecting 46% of pregnant women in Africa and 49% in Asia. Antenatal anaemia causes significant risks for both mother and child. Anaemia can amplify the risks and consequences of serious complications (including maternal mortality) from maternal haemorrhage and contributes to low birth weight and premature delivery, which both pose critical immediate and long-term risks for the survival, development, and wellbeing of the newborn infant.

In pregnancy, anaemia is commonly due to iron deficiency. A recent placebo-controlled double-blind, randomised single-centre field trial in Kenyan pregnant women exemplified the benefits and safety of iron supplementation during pregnancy in the sub-Saharan African context by showing a substantial benefit from iron supplementation on birth weight and the duration of gestation[1]. However, the success of the Kenyan trial resulted from the high adherence demanded of the participants (100%), achieved by daily visits by fieldworkers. The need for such a high adherence emphasises the importance of delivering of a full course of iron supplementation on birth outcomes. Across Africa, only very few women receive the full recommended course of antenatal iron and may present for their initial visit far into the second trimester. This late presentation limits opportunities to treat antenatal anaemia, exposing women and their babies to its consequences.

New intravenous iron products have become available in developed countries and provide a chance to give high doses of iron in a single rapid infusion. Studies comparing older formulations of intravenous iron to oral iron treatment in pregnancy[2–4] found intravenous iron superior for improvements in haemoglobin (e.g., MD 7.4 g/L [3.9, 11.0]) and birth weight (about 58 g) [3,4] and for reducing maternal blood transfusion needs (OR 0.19 [0.05, 0.78]) [3]. Collectively, these data emphasise the critical importance of screening for, preventing and treating ID during pregnancy. There is a real need for the establishment of a parenteral iron formulation in women with moderate or severe IDA in pregnancy, and perhaps in all women with IDA in the third trimester when the foetal iron transfer is highest, and delivery (with the risk of blood loss) is imminent.

Ferric Carboxymaltose is the most commonly used intravenous iron product and provides a single rapid iron infusion suitable for administration in primary care and other non-hospital settings.

Objectives: To determine the effectiveness and safety of intravenous iron administration during the third trimester of pregnancy – given as Ferric Carboxymaltose (FCM) compared with standard of care oral iron in improving maternal (especially anaemia) and infant (growth, birth weight and development up to 12 months postpartum) outcomes.

Methods: This is an open-label two arm parallel-group randomised controlled trial in anaemic pregnant women. The primary maternal outcome is recovery from anaemia at 36-weeks gestation or at delivery, whichever comes first, and the important secondary neonatal outcome is birth weight. Study visits occur over pregnancy, atbirth, and follow-up to 12-month postpartum. Other secondary outcomes include (i) maternal iron- deficiency biomarkers; (ii) maternal postpartum depression and wellbeing levels; (iii) infant neurocognitive development, growth, anaemia, and iron status; (iv) adverse events of administering intravenous iron in this setting – including hypophosphatemia;

and (v) maternal and infant infection (e.g. malaria and diarrhoea) events. The study will have two arms: (a) intravenous iron FCM; (b) oral iron. Apart from the source of iron-supplementation, all ANC procedures will be equal between the arms of the trial which may include IPTp, if scheduled. The intravenous iron Intervention group will receive either i) intravenous FCM 1000mg for body weight 50 kg, or 20 mg/kg for body weight <50 kg, once during the third trimester. The control group will receive standard of care oral iron 200 mg ferrous sulphate (approx. 65 mg elemental iron) twice daily for the duration of pregnancy. Both arms will receive sulfadoxine-pyrimethamine (SP) as IPTp according to national guidelines if the three doses of recommended IPTp have not been already achieved.

The planned number of randomised participants is 590 pregnant women (295 participants per study arm). The study has 90% power at a two-sided alpha level of 2.5% and incorporating a 10% loss to follow up to detect a difference on the primary maternal outcome between intravenous iron arm and oral iron (49% vs 63%). A sample size re-estimation was conducted while the trial was ongoing and confirmed the planned number of 590 randomised participants. The sample is also sufficient to have 80% power to detect a 110g difference in birthweight (assuming standard deviation in birth weight of 450g) between the intravenous iron armand the oral iron arm (two-sided alpha level of 5%) after accounting for a miscarriage and stillbirth rate of 1%. The trial will be based at the TRUE centre at Zomba Central Hospital in Southern Malawi but will operate in health centres across Zomba district. An international team of experts in clinical trials, obstetrics, anaemia, malaria, and implementation sciences based in Malawi and Australia will conduct the trial.

Because anaemia in pregnancy may influence the long-term health of the mother and her baby, we will continue to follow the cohort of women and babies postpartum to assess the longer-term benefits and safety of this treatment. Women and babies will be followed up until 12 months postpartum (infant age 12 months), and will undergo study visits at 4-weeks, 3-, 6-, 9- and 12- months. Mothers and babies will undergo testing for haematologic, iron, nutritional and infection status. Participants will also be consented for use of samples for translational work including immunologic and genetic analyses. Women will undergo testing for wellbeing and depression, and babies will undergo cognitive testing.

Additionally, an implementation study and health system analysis (REVAMP-IS), conducted in parallel, will assess the feasibility of implementing this intervention in routine antenatal care within the mainstream health system in low-income countries.

Expected Findings and Implications: Oral iron supplementation remains cheap while the drug cost of intravenous iron delivery is much higher. Clinical superiority of intravenous iron over oral iron for the recovery of anaemia in the third trimester needs to be demonstrated. Additionally, there is a real need for an in-depth analysis of potential implementation barriers in the setting of antenatal care in LMIC. We hypothesise a clear benefit from intravenous iron on maternal haematologic outcomes and wellbeing, on critical neonatal outcomes such as birth weight and gestation duration, and infant development and wellbeing. We hypothesise that intravenous iron to be safe to administer in primary health care centres. We hypothesise that FCM will cause hypophosphatemia but that this willnot have clinical implications, be asymptomatic and will not affect the child**.** In conjunction with REVAMP-IS, our study may lead to the implementation of intravenous iron as a treatment recovery from maternal anaemia in the third trimester, which could have long term benefits for maternal and child health, ultimately resulting in benefits for maternal and child survival and child development.

Dissemination: We will present the results from this study at local and international fora. The results will be submitted for publication in peer-reviewed scientific journals, policy briefs to WHO and donors such as the Bill and Melinda Gates Foundation and reported to NHSRC and other relevant ethics committees.

4.3. SCHEDULE OF ACTIVITIES

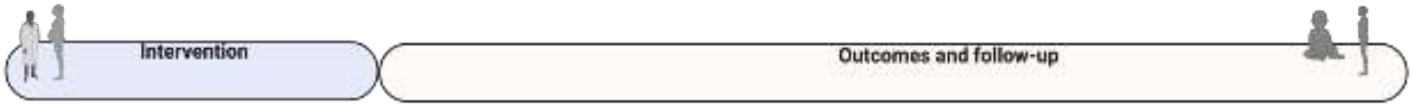

	Visit 0[1]	Visit 1	Visit 4	Visit 7	Visit 8	Visit 9	Visit 10	Visit 11	Visit 12	Sick Visit Unscheduled
**Protocol Activity**	*May occur at visit 1*	*Week 27-35*	*36 weeks ±2d (pre-delivery)*	*Delivery ±2d*	*28 days postpartum ± 2d*	*3 months postpartum ± 14 Days*	*6 months postpartum ± 14 Days*	*9 months postpartum ± 14 Days*	*12 months postpartum ± 14 Days*	
**Location of visit**	**HF[j]**	**HFj**	**HF[j]**	**HF[j]**	**HF[j]**	**HF[j]**	**HF[j]**	**HF[j]**	**HF[j]**	**HF[j]**
**Pre-screening form completion**	X									
**Screening**	X									
**Informed consent process**		X								
**Medical & obstetric history**		X								
**Household economic data form**		X								
**Household food insecurity**			X			X				
**Complete physical examination [a]**		X								
**Limited physical examination [b]**			X	X	X	X				
**Mother Infant Bonding Scale data forms completion**						X	x		x	
**Edinburgh Postpartum depression scale (EPDS)**		x				X	x		x	
**Self-reporting questionnaire**										

[1] Visit numbers are not sequential, but instead are harmonised to match the visit numbers adopted in the ongoing REVAMP-EXT trial (REVAMP-EXT trial – P.02/18/2357). This safeguards the operational success of this trial.

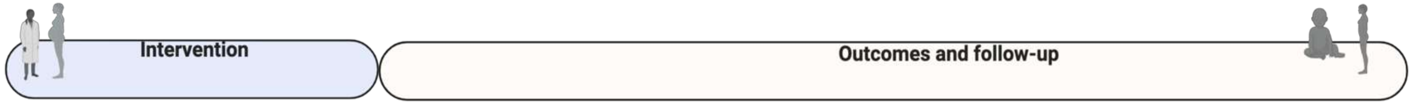

Protocol Activity	Visit 0[1]	Visit 1	Visit 4	Visit 7	Visit 8	Visit 9	Visit 10	Visit 11	Visit 12	Sick Visit Unscheduled
	May occur at visit 1	*Week 27-35*	*36 weeks ±2d (pre-delivery)*	*Delivery ±2d*	*28 days postpartum ± 2d*	*3 months postpartum ± 14 Days*	*6 months postpartum ± 14 Days*	*9 months postpartum ± 14 Days*	*12 months postpartum ± 14 Days*	
**Location of visit**	HF[j]	HFj	HF[j]	HF[j]	HF[j]	HF[j]	HF[j]	HF[j]	HF[j]	HF[j]
**Participant arm allocation**		X								
**Administer treatment**										
**intravenous iron**		X								
**Oral iron**		X								
**Laboratory procedures (Maternal)**										
**Full Blood Count (including Hb)**		X	X	X	X	X	X	X	X	
**Haemoglobin (capillary)**	X									
**Malaria RDT**		X								X
**Malaria microscopy**		X	X	X	X	X	X	X	X	X
**Malaria filter paper for PCR**		X	X	X	X	X	X	X	X	X
**Serum for iron markers tests[c]**		X	X	X	X	X	X	X	X	
**Serum for inflammatory markers tests[d]**		X	X	X	X	X	X	X	X	
**Phosphate**		X	X	X	X		X		X	
**Vaginal swab sample collection[e]**			X		X				X	
**Placenta histology**				X						

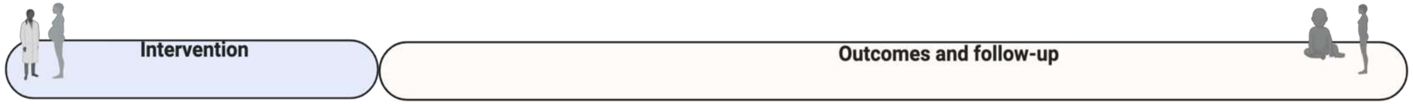

Protocol Activity	Visit 0[1] May occur at visit 1	Visit 1 Week 27-35	Visit 4 36 weeks ±2d (pre-delivery)	Visit 7 Delivery ±2d	Visit 8 28 days postpartum ± 2d	Visit 9 3 months postpartum ± 14 Days	Visit 10 6 months postpartum ± 14 Days	Visit 11 9 months postpartum ± 14 Days	Visit 12 12 months postpartum ± 14 Days	Sick Visit Unscheduled
Location of visit	HF[j]	HFj	HF[j]	HF[j]	HF[j]	HF[j]	HF[j]	HF[j]	HF[j]	HF[j]
Breast milk sample					X		X[g]		X[g]	
Laboratory procedures (Infant)										
Full Blood Count				X (cord)	X		X		X	
Malaria microscopy				X (cord)	X	X	X	X	X	
Serum for iron markers tests[c]				X (cord)	X	X	X	X	X	
Malaria filter paper for PCR				X (cord)	X	X	X	X	X	
Stool sample					X		X		X	
Pregnancy outcome				X	X					
Birth weight				X						
Complete physical examination of baby[h]				X						
Limited physical examination of baby[i]					X	X	X	X	X	
Maternal anthropometry: weight, height				X	X	X	X	X	X	
Child anthropometry: weight, length, head circumference				X	X	X	X	X	X	
Child vaccination and Vit A supplementation status					X	X	X	X	X	

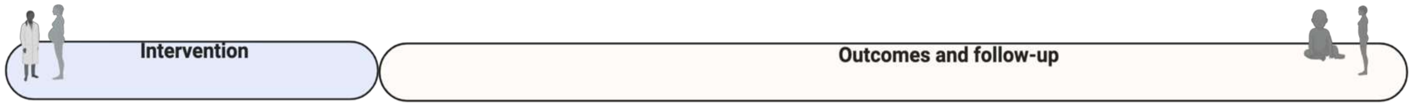

Protocol Activity	Visit 0[1]	Visit 1	Visit 4	Visit 7	Visit 8	Visit 9	Visit 10	Visit 11	Visit 12	Sick Visit Unscheduled
	May occur at visit 1	*Week 27-35*	*36 weeks ±2d (pre-delivery)*	*Delivery ±2d*	*28 days postpartum ± 2d*	*3 months postpartum ± 14 Days*	*6 months postpartum ± 14 Days*	*9 months postpartum ± 14 Days*	*12 months postpartum ± 14 Days*	
**Location of visit**	HF[j]	HFj	HF[j]	HF[j]	HF[j]	HF[j]	HF[j]	HF[j]	HF[j]	HF[j]
**Child dietary diversity**							X		X	
**Simulating caregiving**							X			
**Radiological examination of wrist and knee (x-rays)**										
**Infant neurodevelopment using electroencephalography (EEG), Infant and Toddler Development tools (e.g. Baileys and MDAT)**							X		X	
**Infant neurodevelopment using auditory brainstem responses (ABRs)**					X		X			
**Infant neurodevelopment using low field magnetic resonance imaging (MRI) technology**										

[a] Complete examination: general appearance, throat, neck, thyroid, musculoskeletal, skin, lymph nodes, extremities, pulses, pulmonary, cardiac, abdominal, and neurological examination
[b] Limited examination: general appearance, brief pulmonary, cardiac, abdominal, and neurological examination
[c] e.g., Serum ferritin, sTfR
[d] e.g., CRP and alpha-1 glycoprotein
[e] Reproductive tract microbiome analysis: e.g., *Chlamydia trachomatis, Neisseria gonorrhoeae, Trichomonas vaginalis, Lactobacillus spp.* (done only for a sub-set of 50 women)
[f] Stool microbiome analysis
[g] Done only if mothers are still breast feeding
[h] Complete examination: weight, length, head circumference, APGAR score, Ballard score, new-born adiposity, congenital anomaly, and complications at birth
[i] Limited examination: general appearance, brief pulmonary, cardiac, abdominal, and neurological examination
[j] Visits will take place at a Health Facility

5. Introduction

5.1. Background

5.1.1. Anaemia during pregnancy remains a critical global health problem

Almost 40% of pregnant women worldwide are anaemic, including 46% of pregnant women in Africa and 49% in Asia, compared with 17% in North America[9]. Anaemia in pregnancy is associated with critical risks for both mother and child. The maternal risks include life-threatening complications of postpartum haemorrhage. On the other hand, the risks for the child include prematurity and low birth weight[10] – which are associated with increased risk of mortality, and reduced iron stores in infancy with increased risk of subsequent anaemia and impaired development[11]. Anaemia in pregnancy is associated with an adjusted 3.6 odds ratio for increased maternal mortality[12], and in the USA is associated with impaired long-term child cognitive development. Control of anaemia in women is, therefore, a key 2025 global nutrition target[13].

5.1.2 Low birth weight and pre-term birth have critical implications for mother andbaby

Worldwide, 15%-20% of births worldwide (>20 million annually) (including 13% in sub-Saharan Africa) are low birth weight, while each year, over 1 million children die from complications of pre-term birth. Although the underlying determinants for low birth weight and prematurity are diverse, prevention and treatment of antenatal anaemia with iron is an essential component of the 2025 WHO nutrition target of a 30% reduction in low birth weight[14,15].

5.1.2. Iron can benefit antenatal anaemia

A recent placebo-controlled double-blind, randomised single centre field trial in Kenyan pregnant women exemplified the benefits and safety of iron supplementation during pregnancy in the sub-Saharan African context [1]. In this trial, oral iron supplementation increased birth weight by 150 g and reduced the risk of low birth weight by 58%, lengthened gestation duration by 3.4 days and reduced the risk of premature birth by 7% and reduced the risk of maternal anaemia from 50.4% to 22%. A welcomed observation, in this highly malaria-endemic setting, was that there was no evidence of an increase in clinical malaria, placental malaria or parasitaemia among women randomised to receive iron. This trial is one of the most recent examples of an extensive literature of trials evaluating oral iron in pregnancy. Systematic reviews of these studies confirm likely benefits from iron on maternal outcomes, including anaemia (70% reduction), and trends towards favourable infant outcomes including increased birth weight and extended gestation duration [16]. The success of the Kenyan trial rested on the high adherence demanded of the participants (100%), achieved by daily visits by fieldworkers. This dependency emphasises the importance of delivery of a full course of iron supplementation on birth outcomes.

5.1.3. Uptake and adherence to oral iron therapy during pregnancy is inadequate in thefield

Global recommendations for the management of anaemia in pregnancy in LMICs are that women be treated with high dose daily oral iron (120 mg of elemental iron) supplementation for three months [17,18]. However, such high amounts of iron are often poorly tolerated due to significant gastrointestinal adverse effects[19] limiting adherence to this vital intervention. Moreover, delivery of iron during pregnancy requires ongoing contacts between the mother and the primary health system. For example, in Malawi, fewer than 25% of pregnant women receive a full course of iron (*Clophat Baleti,*

Personal Communication). Across Africa, only very few women get the recommended course of antenatal iron and may present for their initial visit far into the second trimester. This late presentation limits opportunities to treat antenatal anaemia, exposing women and their babies to its consequences. Furthermore, even when delivered, oral iron frequently fails to correct anaemia in routine practice. For example, in our recent study among pregnant women in The Gambia, we provided iron to all women with Hb<10 g/dL at week 20 of gestation; by week 30, 61% still had Hb<10 g/dL[20].

5.1.4. FERRIC CARBOXYMALTOSE: A NEW INTRAVENOUS IRON PREPARATIONS

Over the past decade, there have been dramatic improvements in the safety and convenience of parenteral (intravenous) iron therapies[3–5,21]. Older forms of intravenous iron were associated with severe allergic reactions (iron dextran), required prolonged administration periods (iron polymaltose), or required frequent infusions of small doses of 200 mg (iron sucrose). However, new intravenous agents have entirely changed the landscape of iron therapy.

Ferric carboxymaltose

The most established intravenous therapy is Ferric carboxymaltose (FCM), which overcomes many limitations of previous parenteral iron treatments. Ferric carboxymaltose comprises a colloidal complex of polynuclear iron (III) oxyhydroxide core with carboxymaltose ligands[22]. Following administration, the complex is degraded into simple endogenous molecules (glucose, maltose, maltotriose, maltotetraose and iron), and iron is rapidly taken up by iron transport (transferrin) and storage (ferritin) proteins. Due to this property, FCM can be administered in a short time and at large doses[23]. As currently licensed, a dose of up to 1000 mg of iron (or 15-20 mg/kg body weight), diluted in 250 mL saline, may be administered over a 15-minute infusion. In most cases, this enables a total dose of iron replacement to be achieved in a single visit. FCM has now revolutionised the treatment of iron deficiency anaemia in high-income country settings and is widely used in ambulant/ outpatient settings, emergency departments, in non-specialist clinical wards, preoperative clinics and even in remote settings. FCM has been available in Europe since its approval in 2007 and the USA since 2009 and. Over 50 countries are currently marketing FCM [23].

<u>*Safety and efficacy evidence of Ferric carboxymaltose in various patient populations*</u>

Rognoni et al. 2015 undertook a systematic review and network meta-analysis of FCM vs both oral iron and other parenteral formulations[21]. The authors identified 21 RCTs comparing iron treatments in anaemic (or non-anaemic) patients requiring therapies for ID published between 2003-2014, although not all of these included FCM.

Regarding safety, the review authors found that overall, FCM was well tolerated and associated with minimal risk of AEs[21]. In trials in which AEs occurred in a more substantial proportion of FCM-treated patients than those receiving oral iron or placebo, the difference seldom reached the statistical significance level. Several studies found that patients who were given FCM experienced fewer drug-related gastrointestinal disorders (e.g. constipation, diarrhoea) than those treated with oral iron. However, patients treated with ferric carboxymaltose iron were more likely to develop rash, dermatitis and pruritus that generally resolved within a few minutes of the infusion. Other frequent AEs associated with ferric carboxymaltose administration were fatigue, headache and dizziness. No true cases of anaphylaxis and no deaths occurred in patients receiving FCM.

Regarding efficacy, the authors undertook a network meta-analysis to compare the efficacy of FCM as compared with oral iron and other parenteral formulations. The authors found that in terms of improving haemoglobin concentrations, FCM was superior to placebo (delta 2.1; 95 % CI 1.2–3.0), oral iron (delta 0.8; 95 % CI 0.6–0.9), intravenous ferric gluconate (delta 0.6; 95 % CI 0.2–0.9), and iron sucrose (but not reaching statistical significance). The authors found that for improvements in ferritin, FCM was superior to oral iron (delta 172.76; 95 % CI 66.7–234.4) and similar to other parenteral formulations (iron sucrose and gluconate)[21].

A previous systematic review and meta-analysis published in September 2011 evaluated published and unpublished studies of efficacy and safety of ferric carboxymaltose[24]. The analysis assessed clinical trial reports and published studies comparing FCM with either other active comparators or with placebo. The authors identified fourteen studies, in which 2,348 patients had been assigned to FCM, compared with, in the control arms, 762 to placebo, 832 to oral iron and 384 to intravenous iron sucrose. Compared with oral iron, ferric carboxymaltose produced superior improvements in haemoglobin, ferritin and transferrin saturation, and was associated with a higher chance of resolution of anaemia or clinically significant increase in haemoglobin. Maximum responses were usually achieved by 4-6 weeks. Adverse events were also evaluated.

The table below, from this manuscript, shows the risk of withdrawals and adverse events in patients receiving ferric carboxymaltose compared with those randomised to receive either control or another iron intervention. As compared with either oral iron or placebo, participants receiving FCM were slightly less likely to withdraw from the study, while there was no difference in the number of participants experiencing at least one adverse event, death, or serious adverse events between subjects receiving FCM and those receiving other interventions. However, there was a slight increase in the risk of hypotension in participants receiving FCM when explicitly compared with oral iron (but not when compared with receipt of other intravenous interventions).

Table 2: Risk of Withdrawals and Adverse Events in patients receiving intravenous Ferric Carboxymaltose

Outcome	Comparator	Number of Trials	Number of Patients	Percent with Ferric carboxymaltose	Percent with Control	RB or RR 95% CI	NNTp 95% CI
**Withdrawals**							
All cause	All	10	3835	6.4	7.5	0.8 (0.6 to 0.9)	93 (37 to 180)
	Oral iron	6	1898	8.1	9.3	0.8 (0.6 to 1.03)	not calculated
Adverse event	All	8	3319	1.0	1.6	0.6 (0.3 to 1.02)	not calculated
	Oral iron	6	1898	1.5	1.9	0.7 (0.3 to 1.4)	not calculated
Lack of efficacy	All	7	2967	0.6	0.8	0.8 (0.4 to 1.7)	not calculated
	Oral iron	5	1546	1.1	1.2	0.8 (0.4 to 1.9)	not calculated
**Adverse events**							**NNH 95% CI**
At least 1 AE	All	8	2951	41	38	1.1 (1.0 to 1.2)	not calculated
	Oral iron	5	1539	48	53	1.0 (0.9 to 1.1)	not calculated
Death	All	10	3762	0.53	0.3	1.3 (0.5 to 3.4)	not calculated
	Oral iron	6	1891	0.38	0.0	1.7 (0.4 to 6.6)	not calculated
Serious AE	All	8	3303	2.5	2.3	1.0 (0.6 to 1.5)	not calculated
	Oral iron	6	1891	3.1	2.3	1.3 (0.7 to 2.2)	not calculated
Hypotension	All	6	2694	1.5	1.0	1.5 (0.8 to 2.7)	not calculated
	Oral iron	4	1339	1.3	0.0	4.7 (1.1 to 21)	79 (44 to 390)

Note: NNT - Number Needed to Treat; NNH - Number Needed to Harm; RB – Relative Benefit; RR – Relative Risk

The table below, adapted from this meta-analysis, compares the incidence of various organ-specific side effects associated with the administration of FCM when compared with i) oral iron as the control, and ii) intravenous saline as the control.

Table 3: Organ-specific side effects of ferric carboxymaltose compared with oral iron or intravenous saline

Outcome	Number of Trials	Number of Patients	Percent with Ferric carboxymaltose	Percent with Control	RB or RR 95% CI	NNTp/H 95% CI
			**Comparison with oral iron**			
**Body system and preferred term**						
GI disorder	5	1539	13	32	0.44 (0.36 to 0.54)	5.4 (4.4 to 7.1)
General, administrative site	5	1539	11	4	2.8 (1.9 to 4.2)	**15 (11 to 24)**
Infection, infestation	5	1539	14	12	1.2 (0.9 to 1.6)	not calculated
Metabolism, nutrition, investigation	4	1195	11	5	2.2 (1.4 to 3.4)	**17 (11 to 33)**
Nervous system	5	1539	10	9	1.3 (0.9 to 1.7)	not calculated
**Specific adverse events**						
Constipation	4	1339	3	13	0.3 (0.2 to 0.4)	9.8 (7.6 to 14)
Diarrhoea	3	906	2	5	0.5 (0.2 to 0.9)	33 (18 to 230)
Nausea/vomiting	3	906	3	10	0.4 (0.2 to 0.6)	14 (9.5 to 27)
Headache	5	1539	7	7	1.2 (0.8 to 1.7)	not calculated
			**Comparison with IV saline**			
**Body system and preferred term**						
GI disorder	2	1577	8	5	1.6 (1.1 to 2.4)	**34 (19 to 210)**
General, administrative site	2	1577	6	2	2.5 (1.5 to 4.3)	**25 (17 to 49)**
Infection, infestation	2	1577	9	6	1.1 (0.8 to 1.6)	not calculated
Nervous system	2	1577	8	6	1.2 (0.9 to 1.8)	not calculated
Respiratory system	2	1577	2	2	0.8 (0.4 to 1.5)	not calculated

Note NNTp in normal text, NNH when bold

Note: NNTp - number needed to treat to prevent harm; NNH - Number Needed to Harm; RB – Relative Benefit; RR – Relative Risk

As shown in the table above, compared with oral iron, FCM was less likely to cause constipation, diarrhoea, nausea/ vomiting, and gastrointestinal disorders in general, but was more likely to cause adverse events at the administration site. When FCM was compared to intravenous saline, FCM was associated with an increased risk of 'GI disorder' and local site reactions, but not with adverse effects in other organ systems.

Numerous randomised controlled trials have compared FCM with either oral iron or other forms of parenteral iron for treatment of IDA in a variety of clinical situations, including pregnancy[25], postpartum[26] and in a variety of clinical conditions including inflammatory bowel disease[27–33], chronic renal failure[34,35], and heart failure[36]. It has also been studied in the preoperative context for optimising preoperative haemoglobin concentrations as a component of patient blood management[37–41]. Here, we will summarise recent reviews of FCM that address vital safety and efficacy endpoints.

Safety of Ferric carboxymaltose

Several studies, including one randomised controlled trial, have evaluated the efficacy and safety of FCM in pregnancy. These studies are summarised here:

Breymann et al.[25]*:* FER-ASAP was the pivotal, Phase IIIb, open-label randomised controlled trial comparing FCM with oral iron (ferrous sulphate) for treatment of iron deficiency anaemia in pregnancy. This multicentre study was set across eight high and middle-income countries and randomised 252 women in their second or third trimester of pregnancy to FCM (1000-1500 mg iron) or FS (200 mg iron/day for 12 weeks). The trial was designed to identify a more rapid improvement in haemoglobin concentration by three weeks post-infusion compared with baseline. The study showed an advantage of Hb with FCM compared with FS at 6 and 3 weeks (although the difference was not significant at three weeks). According to the SF-36 health survey, FCM treatment led to significant, clinically relevant improvements over FS in vitality and social functioning before delivery.

Safety: The incidence of Treatment-Emergent Adverse Events (TEAEs) was similar between the treatment arms: in the FCM group, 60 women (49%) experienced 165 TEAEs; in the FS group, 50 women (40%) experienced 105 TEAEs. The majority of events were mild in intensity (see table 5 below).

Table 4: Incidence of Treatment-Emergent Adverse Events (TEAEs)

Treatment-related TEAE severity, number of patients (%)	Ferric carboxymaltose (n=123)	Ferrous sulfate (n=124)
Total	60 (49)	50 (40)
Mild	43 (72)	28 (56)
Moderate	17 (28)	20 (40)
Severe	0 (0)	2 (4)

TEAE=treatment-emergent adverse event.

Overall, the most common TEAEs were nausea (6%), headache (5%) and dyspepsia (4%), and the most common TEAEs according to system organ class were “pregnancy, puerperium and perinatal conditions” in the FCM group [32 events in 26 women (21%)] and “gastrointestinal disorders” in the FS group [42 events in 25 women (20%)]. The most common treatment-related TEAEs were headache with FCM [experienced by 4 women (3%)] and nausea with FS [in 6 women (5%)], and markedly higher rates of gastrointestinal disorders were reported with FS treatment (in 16 women) compared with FCM treatment (3 women).

Table 5: TEAEs in FCM vs FS treatment arms

Treatment-related TEAE,[a] number of patients (%)	Ferric carboxymaltose (n=123)	Ferrous sulfate (n=124)
Total	14 (11)	19 (15)
Nervous system disorders	7 (6)	1 (1)
Headache	4 (3)	1 (1)
Dizziness	3 (2)	0 (0)
Dysgeusia	2 (2)	0 (0)
General disorders and administration-site conditions	4 (3)	0 (0)
Vascular disorders	2 (2)	0 (0)
Gastrointestinal disorders	3 (2)	16 (13)
Nausea	2 (2)	6 (5)
Vomiting	0 (0)	2 (2)
Constipation	0 (0)	3 (2)
Diarrhea	0 (0)	4 (3)
Abdominal pain upper	0 (0)	5 (4)
Dyspepsia	0 (0)	3 (2)

[a]According to physician's assessment; a single patient could appear in multiple classes. Percentages calculated according to treatment group.
MedDRA=Medical Dictionary for Regulatory Activities (version 16.1), TEAE=treatment-emergent adverse event.

Serious TEAEs occurred in 23 women treated with FCM (26 events) and in 10 women treated with FS (11 events); all were single events, except for “failed trial of labour”, “foetal distress syndrome”, “premature delivery”, “premature rupture of membranes” and “threatened labour”, each of which occurred in two women treated with FCM, and “premature labour”, which occurred in three women treated with FS (pre-eclampsia was also reported to have occurred twice in one woman treated with FS). One serious TEAE, “bronchospasm”, which was treatment-related, led to discontinuation of FCM; this event was of moderate-intensity and resolved on the same day after the withdrawal of study treatment. Seven women discontinued treatment with FS because of gastrointestinal TEAEs (n=5), syncope (n=1) and rash (n=1). No hypophosphatemia TEAEs were reported during this study. Eleven women (FCM, n=10; ferrous sulphate, n=1) recorded phosphate levels below the lower normal range threshold [0.6 mmol/L (2 mg/dL)]; these decreases were observed at week three and recovered in all the women to within the normal range by the end of the study.

All other trials have been single-arm prospective trials or retrospective comparative studies.

Froessler et al[42]. undertook a prospective observational study in 65 anaemic pregnant women in South Australia, Australia, receiving ferric carboxymaltose (up to 15 mg/kg) between 24- and 40-weeks gestation (median 35 weeks). The authors observed increases in haemoglobin concentrations at 3, 6 and up to 8 weeks post-infusion (see table 6 below), although, in women with the most severe anaemia at baseline, improvements in haemoglobin were not sustained by two months postpartum.

Table 6: Increase in haemoglobin concentration at 3, 6- and 8-weeks post-infusion

	Gestational age at entry	Pre-infusion	3 weeks post infusion	6 weeks post infusion	8 weeks post infusion (post-partum)
Mild ≥95 g/L	34 (4)	102.1 (1.0) n = 31	108.3 (3.9)* n = 28	120.6 (2.9)* n = 18	113.1 (4.2)* n = 11
Moderate 90-94 g/L	36 (2)	92.6 (0.4) n = 14	105.8 (3.0)* n = 13	108.4 (3.8)* n = 5	92.7 (12.4) n = 3
Severe <90 g/L	34 (3)	83.7 (0.9) 20	100.2 (3.3)* n = 17	110.0 (8.1)* n = 5	93.3 (8.1) n = 4

Data are presented as means (SEM). *p < 0.01 compared to pre-infusion haemoglobin levels.

Safety: No serious adverse effects were recorded in any of the 65 women receiving an infusion. Minor side effects occurred in 13 (20%) patients. One patient required medication with Metoclopramide for nausea and vomiting. All other adverse events were self-limiting. Foetal heart rate monitoring did not indicate a drug-related adverse effect on the foetal heart pattern. Red blood cell transfusions were required by three women (4.6%) in the study cohort, all of whom had a significant peripartum haemorrhage. Adverse effects from the cohort are summarised in table 7:

Table 7: Number of women experiencing a drug-related adverse event following infusion with ferric carboxymaltose (total number of women infused n= 65)

Adverse event	n (%)
Any adverse event	13 (20)
Local (injection site irritation)	
Slight burning sensation	5 (8)
Systemic	
Hypotension	1 (1.5)
Headache	4 (6)
Nausea/Vomiting	1 (1.5)
Pruritus	2 (3)

Zeba et al.[43] undertook a prospective cohort study in 260 pregnant women between 28- and 36-weeks' gestation with haemoglobin <10g/dL in a private obstetric clinic in Faridpur, Bangladesh. All women in this study group were treated with intravenous ferric carboxymaltose as a single dose (500-1000mg) in an infusion time of 15-20minutes. *Safety:* No serious adverse events occurred, with 13% experienced minor adverse effects such as injection site irritation, headache, nausea or vomiting etc. *Efficacy:* Compared with the Hb (mean 8.9g/dl) level before infusion, the Hb (mean 10.53g/dl) level after infusion had significantly (p<0.001) increased.

Mishra et al.[44] undertook a single-arm prospective cohort trial of FCM in Ahmedabad, India, in 108 participants. Women received up to 1500mg FCM in total. *Safety*: Three women experienced local reactions, e.g. itching and irritation at the infusion site, while another five women reported systemic reactions of giddiness, headache and nausea. *Efficacy*: FCM produced an average increase of 2.1g/dL haemoglobin in 3 weeks.

Christoph P et al.[45] undertook a retrospective analysis of 206 pregnant women who had received either FCM or iron sucrose during pregnancy to assess maternal safety and tolerability. The authors observed that Mild adverse events occurred in 7.8% for ferric carboxymaltose and in 10.7% for iron sucrose. The authors also concluded that 'no sign for a negative effect on the fetus of iron infusion could be detected.'

There is limited data on safety of FCM in developing countries. However, an ongoing study in Zomba of effectiveness of FCM given in second trimester (COMREC P.02/18/2357) on maternal and neonatal outcome has not recorded any intervention-related Serious Adverse Events, nor specifically anaphylactic shock with > 97% of recruitment completed.

Use of Ferric carboxymaltose in pregnancy

Based on these studies, licensing by international regulators, including the FDA, EMA and TGA, and clinical experience, FCM has rapidly become an established treatment for anaemia in pregnancy in developed countries. For example, the British Committee for Standards in Haematology 2011 guidelines recommend that parenteral iron be considered for women from the second trimester onwards with iron deficiency anaemia who fail or are intolerant of iron[46]. Many UK health services (NHS Trusts) recommend Ferric Carboxymaltose as second-line therapy for iron deficiency anaemia in pregnancy. For example, the Mid Essex NHS Trust recommends FCM for women with persistent anaemia despite four weeks oral iron, or with haemoglobin <80g/L on any occasion, or who are intolerant of or non-compliant with oral iron; the guideline also recommends FCM as first-line therapy in all women who are anaemic beyond 34 weeks pregnancy[47]. In many cases, use is limited not by concerns of efficacy or safety, but by cost and health economic implications.

A 2012 review suggested that as FCM and other novel parenteral iron agents represent a 'milestone' in intravenous iron therapy, they should be considered as increasingly first-line for treatment of anaemia in pregnancy[47]. Here, the authors suggested that women with Hb<10g/dL should probably receive intravenous iron if they have ferritin <30mg/L, and among women with higher ferritin levels, this represents an area of research in establishing whether intravenous or oral iron should be given.

However, since the publication of the FER-ASAP trial, use of intravenous FCM in pregnancy is becoming standard treatment. For example, a recent influential 'How I Treat' review article in the leading haematology journal 'Blood' formalised the new position of parenteral iron as first-line therapy for iron deficiency anaemia in pregnancy[48]. Here, the authors suggest that **all** cases of anaemia in the second or third trimester should receive intravenous iron.

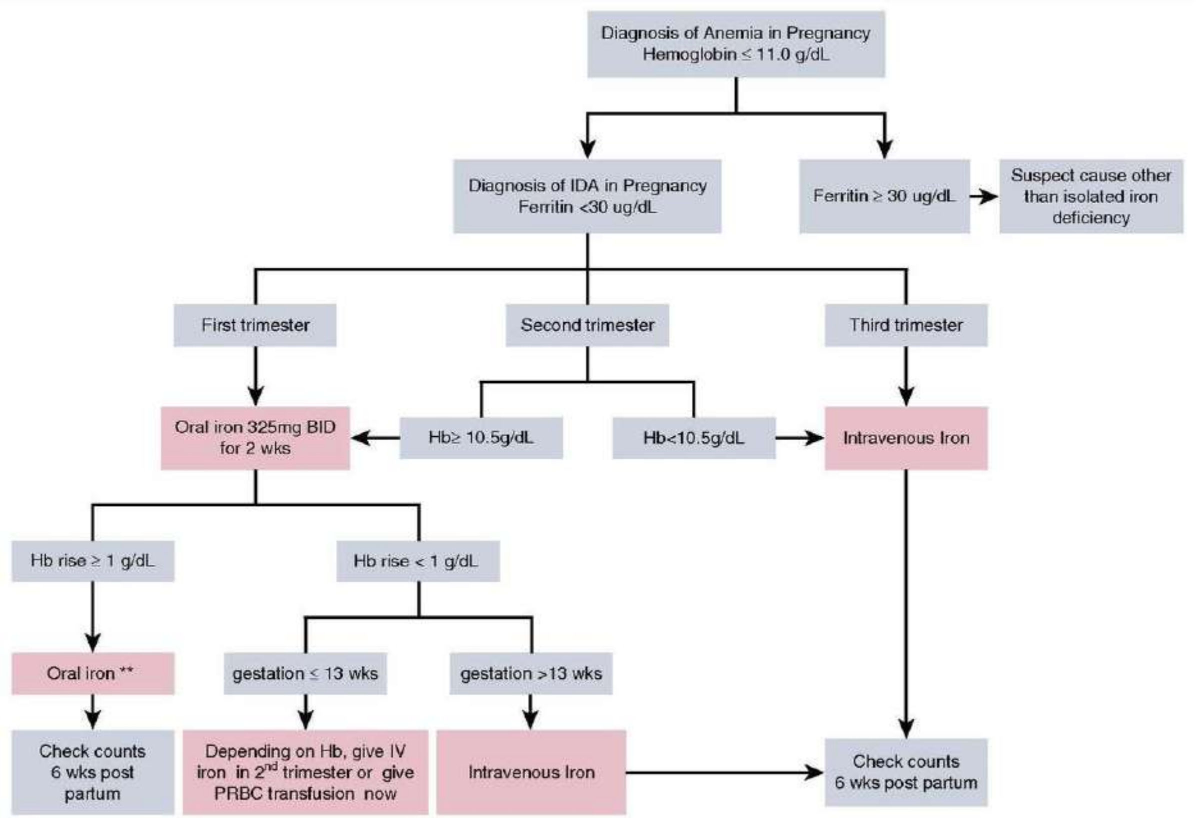

Figure 1: Decision tree on parenteral iron as first-line therapy for iron deficiency anaemia in pregnancy [From Achebe MM et al. How I treat anaemia in pregnancy: iron, cobalamin, and folate. Blood 2017; 129:940-9].

Thus, in high-income countries, FCM is now mainstream, routine therapy for iron deficiency anaemiain pregnancy. Since FCM is already licensed for the treatment of anaemia in pregnancy beyond the first trimester, using European MHRA guidelines, the REVAMP trial would be categorised as Type A = No higher than the risk of standard medical care.

Importantly, although FCM has entered routine care, there remains little data on the effects of antenatal FCM (or intravenous iron) on postpartum health or long-term infant growth and development. A study (COMREC P.02/18/2357) is currently underway in Zomba to determine the effectsof FCM given in second trimester on maternal and neonatal outcome. This study will provide evidence on the effect of FCM given in third trimester on maternal and neonatal outcome. We hypothesise that the provision of FCM in third trimester will quickly raise women’s haemoglobin levelsand thus protect her from anaemia in the intrapartum period and facilitate the recovery postpartum. Furthermore, infants born from women who received third trimester FCM will have more iron storescompared oral iron as new information suggests that much of the iron transfers to the foetus occurs in the third trimester (Whittaker, 1991).

Hypophosphataemia and Ferric Carboxymaltose

Hypophosphatemia is increasingly recognised as a complication of intravenous treatment of FCM and is mediated by increases in intact FGF23 which, in turn, acts on the kidney to inhibit phosphate reabsorption and hence urinary phosphate wasting[72].

Ferric carboxymaltose is designed to be used within a 15-20 min window without the need for dose testing and with the added advantage of being suitable for administration in primary care and other non-hospital settings. Although the clinical replenishment of iron is effective with the use of FCM, there are a few recent studies that show patients presenting with hypophosphataemia when treated with FCM[6–8]. There are two RCTs conducted to evaluate the question of hypophosphataemia in patients with IDA that did not tolerate oral iron[6]. The results demonstrated that the incidence of hypophosphatemia was lower after Isomaltoside (a new IV iron formulation) vs FCM (trial A: 7.9% vs 75.0% [adjusted rate difference, –67.0% (95%CI, –77.4% to –51.5%)], P < .001; trial B: 8.1% vs 73.7% [adjusted rate difference, –65.8% (95%CI, –76.6% to –49.8%)], P < .001)[6] (Figure 4).

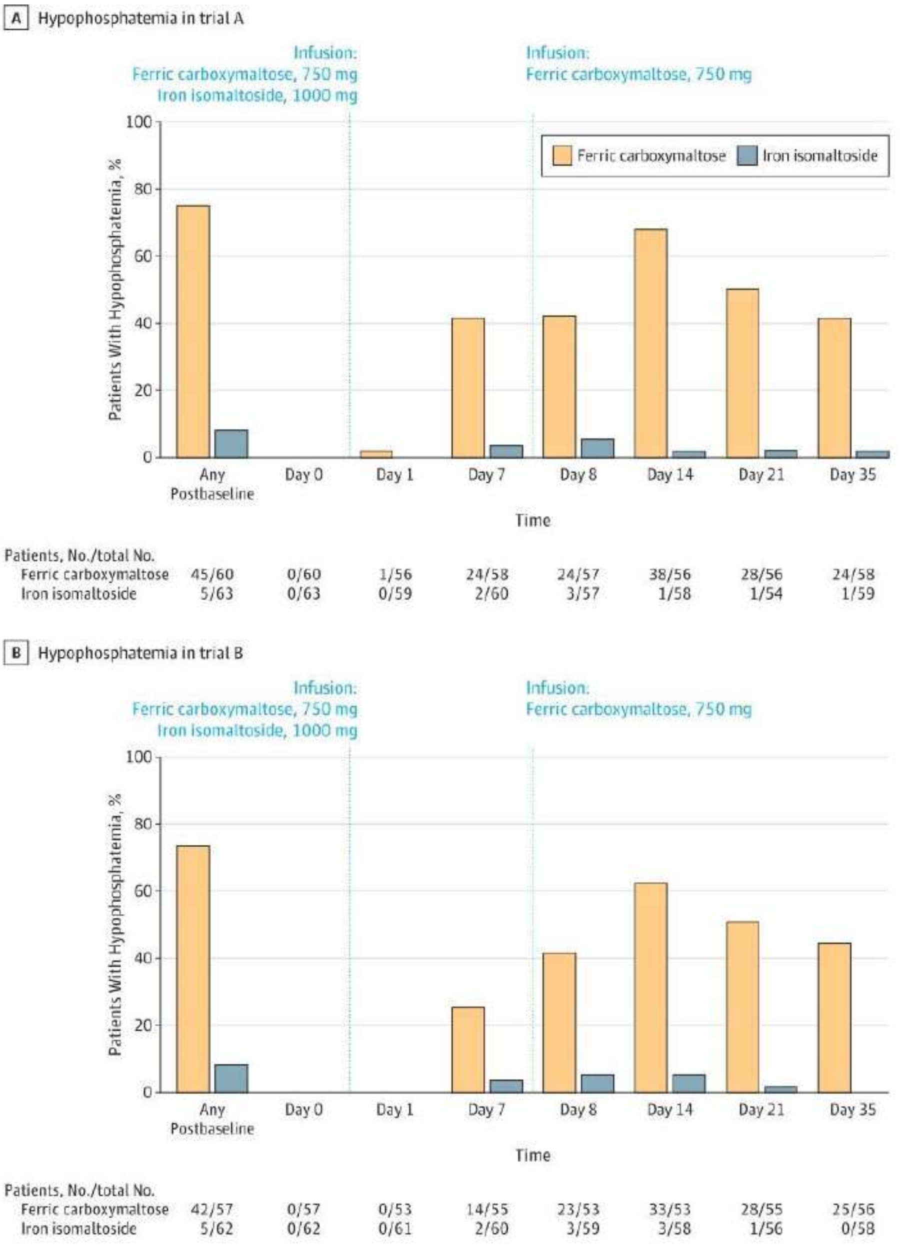

Figure 4: Hypophosphatemia in two RCT comparing FCM and Isomaltoside. The leftmost columns correspond to the primary outcome of incident hypophosphatemia at any time during the trial. The remaining columns correspond to the proportions of patients with serum phosphate level less than

2.0mg/dL at each individual time point in the safety analysis set. [Graph taken from *Wolf M, Rubin J, Achebe M, et al. Effects of Iron Isomaltoside vs Ferric Carboxymaltose on Hypophosphatemia in Iron-Deficiency Anemia: Two Randomised Clinical Trials. JAMA - J Am Med Assoc 2020; 323: 432–43.]*

Although it starts to become evident that the administration of FCM results in hypophosphatemia this is not associated with any significant clinical outcomes. Additionally, the effects of transient maternal hypophosphatemia have not been fully defined in infants; conceivably, hypophosphatemia could promote adverse growth or deficient bone mineralisation[72]. Further, the severity and duration of hypophosphatemia in these women in Africa, where nutritional deficiencies may be more common, may mean there are important variations in the effects of the drug on phosphate levels. Our ongoing REVAMP trial (COMREC P.02/18/2357) in Zomba will determine incidence of hypophosphatemia after FCM treatment. Confirming the safety and avoiding the risk of unexpected adverse events after IV iron administration is a key component of building the case for choosing the appropriate parenteral iron formulation to use in the African context.

5.1.5. INTRAVENOUS IRON AND BENEFITS FOR THE POSTPARTUM PERIOD

Postpartum anaemia is a frequent condition, especially in developing countries, and is mainly attributable to prepartum iron deficiency/anaemia together with blood loss during delivery [73,74]. The significance of this observation is that by adequately treating women during pregnancy, we are not only addressing the woman and the baby’s health during that period but also, addressing the postpartum period[73]. This becomes even more relevant when there is evidence that demonstrates the impact that postpartum iron deficiency/anaemia has on both maternal physical and psychological health[74]. A recent review by Azami et al. [75] found that both antepartum and postpartum anaemia increased the risk of maternal postpartum depression. However, there is limited knowledge on the long-term effect of antenatal intravenous iron on maternal physical and psychological health during the postpartum period.

Intravenous iron during pregnancy and postpartum anaemia

There are multiple benefits to the use of intravenous iron for the treatment of iron deficiency anaemia during the postpartum period, however there is very little data regarding the benefits that intravenous iron during pregnancy has on iron deficiency anaemia postpartum[76].

One study, looking retrospectively at levels of haemoglobin in the postpartum period in women who received Isomaltoside during pregnancy compared to control women who were not anaemic during pregnancy and thus did not receive Isomaltoside, showed no difference in the levels of haemoglobin between the two groups of women (mean Hb Isomaltoside group: 104 g/L vs mean Hb non-anaemic group: 107 g/L, n= 213 for both groups)[77].

6. Trial objectives and outcomes

6.1. Objectives

6.1.1. Broad objective

To determine the effectiveness of intravenous iron – given as FCM - once during the third trimester (27-35 weeks' gestation) compared to oral iron, as measured by anaemia recovery by 36 weeks' gestation or at delivery, whichever comes first.

6.1.2. Specific objectives

Efficacy (Maternal and Neonate/Infant)

- Compare the effectiveness of FCM versus standard of care oral iron on recovery from anaemia by 36 weeks' gestation or at delivery, whichever comes first.
- Compare the effectiveness of FCM versus standard of care oral iron on iron deficiency and iron deficiency anaemia by 36 weeks' gestation or at delivery, whichever comes first..
- Determine the effectiveness of intravenous iron on neonatal outcomes, including birth weight and gestation duration.
- Determine the sustained effects of intravenous iron on child outcomes including neurocognitive development, growth, anaemia, immune status and iron status
- Determine the sustained effects of intravenous iron on postpartum maternal outcomes including wellbeing and depression, at 3, 6, 9 and 12-months postpartum.

Safety (Maternal and Neonate/Infant)

- Assess the safety of intravenous iron in a real-life setting, by trial arm.
- Evaluate the effects of FCM on hypophosphatemia at 36 weeks' gestation or at delivery, whichever comes first.
- Assess the frequency of unplanned visits to the clinic resulting from clinical malaria or diarrhoea by trial arm.
- Evaluate the effect of FCM on hypophosphatemia in cord blood at delivery, and in maternal and baby venous blood at three months postpartum.

6.2. Outcomes

The use of FCM for the recovery of anaemia during the late stages of pregnancy requires a strong demonstration of the efficacy and safety of this intervention. Additionally, the feasibility of applying this intervention to the health care settings where the study is being conducted will provide the necessary framework for the deployment of this strategy in other similar settings.

6.2.1. Primary outcome

Proportion of women with anaemia (defined as venous blood Hb < 11.0 g/dL) at 36 weeks' gestation or at delivery, whichever comes first.

6.2.2. Secondary outcome (maternal benefits)

- Mean change from baseline in maternal Hb at 36 weeks' gestation or at delivery, whichever comes first.
- Proportion of women with maternal iron deficiency (ferritin<15mg/L, sTfR/Ferritin index) at 36

weeks' gestation or at delivery, whichever comes first..

- Mean levels of maternal iron biomarkers at 36 weeks' gestation or at delivery, whichever comes first..
- Proportion of women with maternal inflammation (using C-reactive protein) at 36 weeks' gestation or at delivery, whichever comes first..
- Proportion of women with maternal postpartum haemorrhage.
- Mean change from baseline in maternal Hb at 1, 3, 6, 9 and 12 months postpartum.
- Proportion of women with maternal anaemia at 1, 3, 6, 9 and 12 months postpartum
- Mean levels of iron biomarkers at 1, 3, 6, 9 and 12 months postpartum
- Proportion of women with iron deficiency (defined by ferritin<15mg/L) at 1, 3, 6, 9 and 12 months postpartum
- Proportion of women with postpartum depression (defined by EDPS > 13) at 3 months postpartum.
- Proportion of women with maternal inflammation (defined by C-reactive protein) at 3 months postpartum

6.2.3. Secondary outcomes (neonate/Infant benefits)

- Mean gestation duration (in weeks)
- Mean birth weight and birth length (in grams and centimetres, respectively, within 24 hours of delivery).
- Proportion of sub-optimal pregnancy outcomes (defined as a composite outcome: low birthweight (<2500g); prematurity (birth <37 weeks); small for gestational age (centile score) as defined by International reference standards for gestational age-specific birthweight; stillbirth).
- Proportion of neonates born prematurely (defined as birth before 37 week's gestation)
- Proportion of infants with low birth weight (defined as a birth weight <2500g).
- Proportion of stillbirth.
- Proportion of neonatal mortality.
- Mean cord blood Hb and ferritin.
- Proportion of neonates with anaemia (with correction for gestational age).
- Child development scores measured by Evoked Response Potentials (ERP) at 6 and 12 months of age.
- Child development scores measured by Bayley Scales of Infant and Toddler development and the Malawi Development Assessment Tool at 6 and 12 months of age
- Child neurodevelopment scores measured by low field Magnetic Resonance Imaging (MRI) at 3 and 12 months of age.
- Mean child physical growth as defined by z-scores at 1, 6 and 12 months of age.
- Mean infant haemoglobin (capillary) at 1-, 6- and 12-months postpartum.
- Proportion of infants with anaemia at 1-, 6- and 12-months postpartum.
- Mean levels of iron biomarkers at 1-, 6- and 12-months postpartum.
- Proportion of infants with iron deficiency at 1-, 6- and 12-months postpartum.

6.2.4. Secondary outcome (maternal safety)

- Proportion of women with at least one treatment related adverse effects (occurring immediately post-infusion, and within 7 days of commencement of treatment.

- Incidence of treatment related adverse effects (occurring immediately post-infusion, and within 7 days of commencement of treatment).

- Number of unplanned visits to the clinic (cause specific for diarrhoea and clinical malaria).
- Incidence of all-cause sick clinic visits during the antenatal, postpartum and overall participant follow-up period.
- Incidence of cause-specific sick clinic visits (in particular malaria, diarrhoea and other infectious conditions) during the antenatal, postpartum and overall participant follow-up period
- Proportion of women with placental malaria (past or active infection on histology, parasites on placental blood film).
- Proportion of women with malaria parasitaemia (asymptomatic) at 36 weeks' gestation (or at delivery, whichever comes first), detected by a) microscopy, b) rapid diagnostic tests, and c) PCR.
- Proportion of women with bacteraemia at 36 weeks' gestation or at delivery, whichever comes first.
- Proportion of women with hypophosphatemia (clinical and biochemical) at 36 weeks' gestation, baseline, delivery, 28 days postpartum, 3 months postpartum, 6 months postpartum and 12 months postpartum.

6.2.5. *SECONDARY OUTCOME (CHILD SAFETY)*

- Proportion of infants with hypophosphataemia (biochemical) at 1- and at 6 months of age.
- Number of unplanned infant visits to the clinic (cause specific for diarrhea and clinical malaria).
- Incidence of all-cause sick clinic visits in infant by 12 months of age.
- Incidence of cause-specific sick clinic visits (in particular malaria, diarrhoea and other infectious conditions) in infant by 12 months of age.
- Proportion of infants with malaria parasitaemia (asymptomatic) 1, 3, 6, 9 and 12 months postpartum detected by a) microscopy, b) rapid diagnostic tests, and c) PCR.
- Proportion of infants with radiological Rickets at 3 and 12 months of age.

7. STUDY DESIGN

This will be a Phase III two-arm open-label individual-randomised controlled trial in women with moderate or severe anaemia (capillary Hb<10g/dL) during their Third Trimester (27-35) weeks gestation) (REVAMP-TT). Participants will be randomised to receive either parenteral iron – in the form of FCM– or standard of care oral iron. The trial will be based at the TRUE centre at Zomba Central Hospital in Southern Malawi but will recruit from health centres across Zomba district. Babies and mothers will be followed up to 12 months (see below).

8. PARTICIPANT INCLUSION AND EXCLUSION CRITERIA

Inclusion Criteria[2]*:*

- Confirmed singleton pregnancy in the third trimester (27-35 weeks of gestation, dated by Last Menstrual Period and fundal height).
- Moderate to severe anaemia not requiring an immediate blood transfusion (Hb <10g/dl).
- Negative malaria parasitaemia by RDT.
- Currently afebrile with no evidence of septicaemia.
- Resident in the study catchment area of Zomba district.
- Able to deliver at health facilities within Zomba district.
- Written informed consent (including assent if <18 years old).

Exclusion criteria (at enrolment):

- Previous enrolment in REVAMP trial (REVAMP trial – P.02/18/2357)[3].
- Actively participating in another intervention trial.
- Known hypersensitivity to any of the study drugs.
- Clinical symptoms of malaria or other infection (no fever, no focal symptoms of internal infection i.e. LRTI/ diarrhoea).
- Any condition requiring hospitalisation in the next seven days or serious concomitant illness.
- Known history of sickle cell or sickle-haemoglobin C anaemia.
- Clinically low haemoglobin level requiring a blood transfusion (usually Hb <5g/dl).
- Preeclampsia

[2] Participants who are HIV positive will remain eligible to be enrolled.

9. STUDY SITES

The trial will be based at the TRUE centre at Zomba Central Hospital in Southern Malawi. This is a well-established clinical research site with all resources available to recruit eligible participants, prepare and administer the study drugs, monitor safety, treat adverse effects, and measure trial outcomes (22,230 women above 18 years, 4173 births per year, 13% with Hb <10g/dL respectively). This site recently participated in a large multicentre trial of antenatal anti-malarial treatment (IPTp), and is presently successfully implementing the ongoing REVAMP trial (evaluating intravenous Ferric Carboxymaltose in the second trimester of pregnancy), demonstrating the capability to undertake such studies[78]. Recruitment will be performed from health centres across Zomba district.

In order to directly define and discover aspects of possible field implementation of intravenous iron, *screening* of haemoglobin for eligibility and *administration* of the intervention (i.e. provision of the intravenous iron) will be performed *in the government health centre*. We expect to work across various health centres as part of this process. Our study team will develop procedures and deliver training to ensure that intravenous iron can be safely implemented *within the government system infrastructure.* Trial activities (i.e. enrolment, informed consent, randomisation, outcome measurement and data collection) will be performed by study staff. Still, aspects related to the implementation of the intervention (screening, selection for consideration of the trial, and provision of iron) will be done at least in some cases by government health staff trained and mentored by our team.

Once women are enrolled in the trial, we will carefully monitor them and provide transport to ensure loss to follow up is minimised. This includes the provision of transport for week 36, delivery and postpartum visits.

We will work at Zomba Central Hospital and in health centres within Zomba district, including the nine health centres described below (Figure 5). The number of new women seen at each of these nine antenatal clinics monthly is shown in brackets.

1. Likangala: (150 per month)

2. Bimbi: (75 per month)

3. Lambulira: (80 per month)

4. Domasi: (100 per month)

5. Naisi: (55 per month)

6. Matawale: (600 per month)

7. City clinic: (90 per month)

[3] REVAMP is a pregnancy trial, looking to determine whether IV ferric carboxymaltose given once during the second trimester is effective and safe in improving maternal, neonatal and infant outcomes for treatment of moderate to severe maternal anaemia among pregnant women in Blantyre and Zomba districts of Malawi. This trial is led by the same PIs as REVAMP-TT.

8. Sadzi: (200 per month)

9. Zilindo: (90 per month)

The expected total of new women attending these antenatal clinics is ~800 per month. These clinics are within a 30 km radius from Zomba. Based on current data, about 7% of participants screened in Zomba are eligible for the trial. Thus, we expect it will take us 12 months to recruit for the trial.

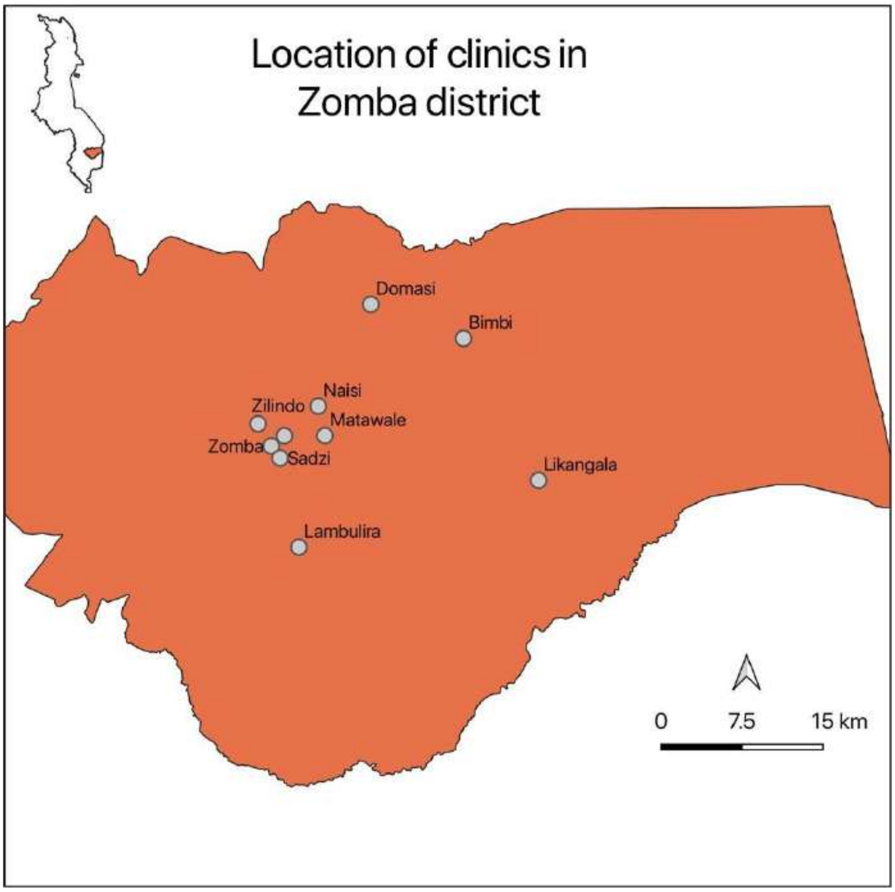

Figure 5: Approximate locations of the health centres where recruitment is going to take place.

10. TRIAL INTERVENTIONS

This is a two-arm parallel-group open-labelled trial, and the intervention groups will receive an intravenous iron given in the form of FCM at recruitment, and the control group will receive oral iron through the routine health care system. Participants will be assigned to the following:

1. **intravenous iron treatment course**: intravenous ferric carboxymaltose (FCM**)** 1000 mg for body weight ≥50 kg, or 20 mg/kg for body weight <50 kg) given over 15 min once at recruitment (Day 0);

OR

2. **Oral iron treatment course**: oral iron- 200 mg ferrous sulphate (approx. 65 mg elemental iron) twice daily for remainder of pregnancy.

If scheduled, participants will receive IPTp with SP, 1500 mg sulfadoxine and 75 mg pyrimethamine (3 fixed tablets of SP strength at 500 mg/25 mg) as recommended in the national guidelines [79]. As part of the safety assessment, IPTp-SP post-randomisation will be directly observed by study staff.

The oral iron will be given under real-life health service delivery conditions where the participant is given three months of oral iron at presentation to the antenatal clinic. This strategy is being employed as it is a key hypothesis in this trial that there is a reduced effect of oral iron on maternal anaemia due to poor adherence to the full course of treatment.

10.1. ALLOCATION TO TREATMENT

The allocation of participants to treatment will not be dictated by the clinical staff's knowledge of treatment nor will the decision to recruit a particular participant affect the order in which participants are recruited (see *section 14*).

10.2. BREAKING THE BLIND

10.2.1. PARTICIPANT LEVEL

This is an open-label trial. The participants are not blinded to the type of intervention they are receiving.

10.2.2. STUDY LEVEL

Though it is an open label trial, laboratory scientists, midwives, investigators and personnel in Australia (including statisticians in Melbourne) will be blinded to the treatment of the participant until the database has been cleaned for analysis.

11. STUDY PROCEDURES

All the recruitment and follow-up schedule are demonstrated in the figure below (Figure 6):

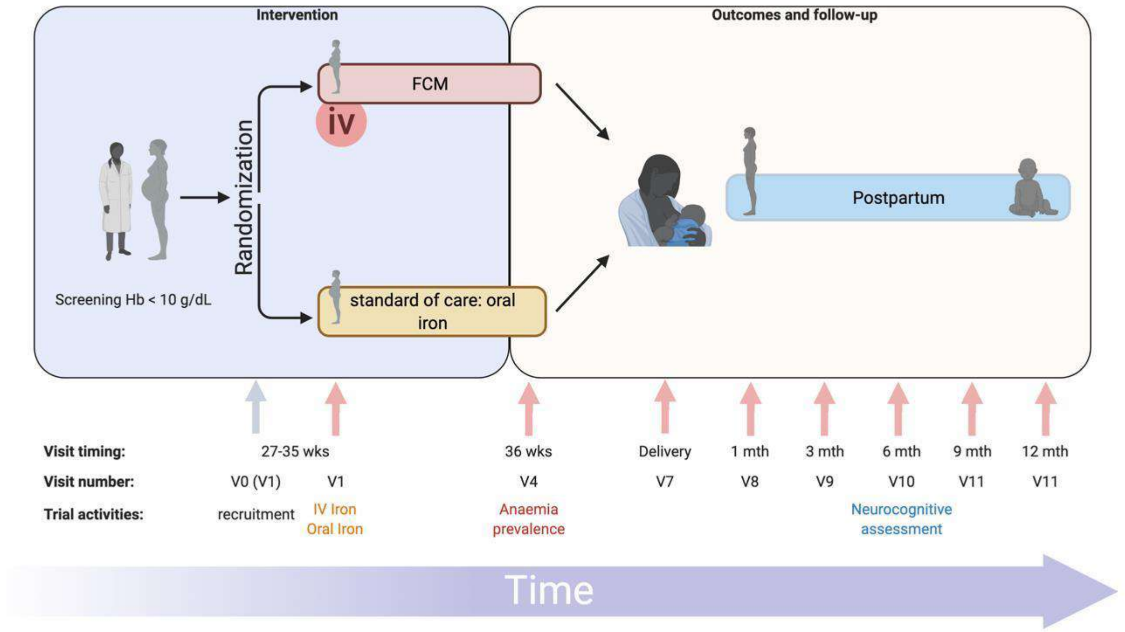

Figure 6 – REVAMP-TT Study schematic
Trial design with visit timings, visit numbers (standardised to match those of REVAMP trial – P.02/18/2357) and main trial activities are represented. FCM – Ferric Carboxymaltose; iv – Intravenous; Hb – Haemoglobin; Wks – weeks

Standard Operating Procedures (SOPs) will be used for all laboratory procedures. These SOPs were developed by senior laboratory technologist and reviewed by principal investigators (refer to Section 14 – Laboratory procedures).

11.1. SCREENING – VISIT 0[4] [DAY 0]

Each day at screening sites, a government-employed nurse trained to screen for study participants will begin with a health talk and include general information about the study (importance, aims and procedures). Health talks are routinely given to ANC attendees before the clinic begins. The screening nurse will then, as she/he processes ANC attendees, screen for potential participants by noting the residency, gestation on clinical examination and Hb and malaria RDT results. The nurse will then communicate to the recruitment site if there are potential participants. Recruitment – visit 1 [day 0]

If all the screening criteria (appendix 1), including giving consent (appendix 2), are met, participants will be administered a questionnaire to collect demographic and medical information before having a full physical examination by a clinician (appendix 1).

[4] Reminder that visit numbers reflect a harmonisation with the existing REVAMP protocol.

11.1.1. Blood sampling

Experienced nurses or laboratory personnel will use standard venous blood collection SOPs to obtain the blood specimens. This will be done using aseptic techniques. Up to 3 attempts will be permitted to collect blood, after which we will just collect a capillary sample for haemoglobin estimation.

The field site will have all the equipment necessary for the sample collection and the initial storage (at 2-4°C or -20°C) before transporting to a central lab where trained laboratory personnel will be based. During transportation from the field to the district laboratory, the cold chain will be maintained. At the main laboratory, malaria microscopy (including staining) and processing of samples will be completed by the laboratory personnel.

A 10 ml venous blood sample will be collected for Full blood count (FBC) and reticulocyte count, iron (e.g. ferritin, sTfR) phosphate and inflammatory markers (e.g. C-reactive protein and alpha-1 glycoprotein). Malaria parasitaemia will be sought by Rapid Diagnostic Test. A filter paper sample of blood will be taken and stored for malaria PCR. Samples will be stored in order to assess other biochemical, immunological and metabolic factors associated with maternal health.

11.1.2. Study arm allocation

A study nurse will assign the participant a Study Number. This will be from a list of consecutive numbers. The study nurse will then retrieve the sealed specific study Number-marked envelope containing the study arm allocation and open it to allocate the participant study arm. This will be done in the presence of the participant. The assigned study number will be recorded in the CRF (Clinical Record Form) and Pharmacy Log.

11.1.3. Study drug administration

Those allocated to the intravenous iron arm will be given Ferric carboxymaltose – 20mg/kg up to 1000mg (women 50kg or above) in 250mL normal saline – intravenously over 15 minutes. The iron formulations will be diluted following procedures which shall be additionally outlined in a specific SOP. The study drug will be provided to the health worker for administration. An intravenous cannula will be inserted following standard aseptic procedure. The skin will be cleaned with ethanol, and a sterile cannula will be inserted into the forearm or hand by a skilled clinician, and the cannula will be fixed in place with a sterile Tegaderm or clinical tape. The participant will be monitored over the 15 minutes of the infusion for any adverse events, and if they develop, these will be attended to promptly and treated according to standard clinical management guidelines. The participant will be observed for a further 30 minutes. Following completion, the cannula will be removed and placed in a biohazard container, and a band-aid applied to the arm. We will follow universal precautions whilst working with sharps. Administration of iron will be done in a room equipped with a 'crash' trolley which will contains adrenaline, hydrocortisone, intravenous fluids and antihistamines. The room will also contain airway equipment (laryngoscope and endotracheal tubes) and Oxygen, for emergencies. The contents and expiry of the crash trolley and availability of oxygen will be confirmed each day by the study staff prior to administration of the drug. For those allocated to the oral iron arm, they will be provided with oral iron supplements to be taken twice daily for 90 days (or the duration of pregnancy, whichever is shorter). Women in the oral iron arm will not be encouraged to take the tablets more than the standard routine antenatal advice as we want this arm to reflect current practice as much as possible.

Women in the intravenous iron arm will be instructed not to take the oral iron; this will also be noted in the woman's health passport so as to notify any other healthcare professionals she seeks care from during her pregnancy. Women randomised to oral iron will be administered the tablets and educated according to a standard script that replicates instructions provided in routine clinical practice in Malawi.

11.1.4. Socio-economic data collection

All participants will be asked to provide information pertaining to their demographic as well as socioeconomic status. This will help us identify factors associated with trial outcomes, as well as to take into account various socio-economic variables associated with anaemia and general maternal health.

11.1.5. Other procedures

We will provide all pregnant women presenting for their first ANC visit an insecticide-treated net (ITN) and if scheduled, an SP-IPTp dose as standard policy in Malawi for the prevention of malaria in pregnancy. SP-IPTp should be given unless it was given within the previous month. Participants will then be dropped home by one of the research assistants to enable him/her to collect a detailed residency map in order to ease tracing of defaulters and allow for location of home visits. Participants will be requested to return to the research clinic (at the recruitment site) at any time that they feel ill during their pregnancy. Otherwise, they will be encouraged to attend all scheduled ANC visits at their local health centre. All participants will be requested to deliver at the research site health facility or notify the research staff if in labour or delivery occurs at any other health facility.

11.2. 36-week follow-up [± 2 days] – Visit 4

All participants will be requested to return to the research clinic at 36-weeks gestation. They will undergo a physical examination as per standard ANC procedures. Adherence to iron therapy will also be determined in the oral iron study arm *(see section 11.2.3 for details)*. This visit includes our primary outcome measure – venous haemoglobin level. They will further have some biological samples collected as follows:

11.2.1. Blood sampling

A 10 mL venous blood sample will be collected for Full blood count (FBC) and reticulocyte count, iron (ferritin, sTfR) phosphate and inflammatory markers (C-reactive protein and alpha-1 glycoprotein). A thin and thick smear of blood and a filter paper sample of blood will be taken and stored for malaria PCR. In case the participants present with clinical symptoms consistent with a malaria infection, malaria parasitaemia will be sought by Rapid Diagnostic Test and the participant will be referred for treatment if the results are positive. Samples will be stored in order to assess other biochemical, immunological and metabolic factors associated with maternal health.

11.2.2. Reproductive tract microbiota

A vaginal swab will be performed. Samples will be stored in appropriate buffers for non-diagnostic assessment of vaginal and cervical microbiomes (including *Lactobacillus spp.*, *T. vaginalis*, *C.*

trachomatis and *N. gonorrhoea*). In case there are any clinical indications that the participants have an infection or any other condition affecting the urogenital system, women will be referred back to the clinic and treated according to national guidelines.

11.2.3. Oral iron arm - Compliance

Women randomised to the oral iron arm of the study will be encouraged to provide any remaining iron tablets so that a pill count can be done to assess their adherence to the treatment.

11.2.4. Maternal psychological health

All women will be asked to complete the short version of the Depression, Anxiety and Stress Scale (DASS-21) form and the Edinburgh Postnatal Depression Scale (EPDS) form. These tools are designed to screen women for any symptoms of emotional distress.

11.3. Delivery [+1 day] – visit 7

All participants will be encouraged to deliver at Zomba Central Hospital. Participants delivering at home or at other health facilities will be asked to come within 24 hours to the research site for assessment. If this is impossible, a study field worker will travel to the participant's home to collect all relevant study data. A detailed physical examination will be done on admission and detailed clinical data on the progress of the delivery. A maternal venous blood sample will be taken (for laboratory tests as below). Delayed cord clamping procedures will be standardized. Immediately after delivery, we will record Apgar scores, and the neonate will have a full physical examination, including measurement of birth weight, length, and details of any congenital malformations. We will record whether the birth was vaginal, vaginal but assisted, or caesarean, and the indications for assisted/ caesarean births; we will also record the occurrence of complications such as haemorrhage, and episiotomy.

11.3.1. Blood sampling

Placental and cord blood will also be collected for malaria microscopy and PCR. Blood samples will be stored in order to assess other biochemical, immunological and metabolic factors associated with maternal, neonatal or infant/child health. In case the participants present with clinical symptoms consistent with a malaria infection, malaria parasitaemia will be sought by Rapid Diagnostic Test and the participant will be referred for treatment if the results are positive

11.3.2. Placental tissue - histology and storage

The placenta will be weighed, and results recorded. A histological full-thickness placental sample about 2 cm long x 1 cm wide will be collected into 40 mL of 10% neutral buffered formalin for malaria histological staining and examination. Sections of the placenta will be stored and may be used for subsequent immunological, metabolic and/or biochemical analyses related to maternal, neonatal or infant/child health. Mothers will be informed these samples may be sent overseas for analysis in cases where the relevant assays are not available in Malawi.

11.3.3. Infant neurodevelopment

We will measure Auditory Brainstem Responses (ABRs) within 48 hours of delivery. ABRs are reflective of maturation of the auditory pathway at various levels (e.g. acoustic nerve level to brainstem level). There is a rapid maturation of the ABR during the perinatal period that is influenced by the degree of myelination, neuronal development, synaptic function, and axonal growth in the auditory nervous system (i.e., changes we expect to be influenced by the level of iron). This assessment does not require any participation by the child; indeed, it is possible when the child is sleeping in their mother's arms. A signal (sound) is provided to the child, and the response is measured. The test is non-invasive, and only requires the fitting of electrodes on the scalp. It does not pose any risk to the child and take less than 20 minutes per child. Drs Larson, Johnson and Bode (co-investigators) are experts in this test.

11.4. 28 days Postpartum [± 2 days] – visit 8

All participants will be requested to return to the research clinic together with their infants for a detailed medical examination of both the mother and the baby.

11.4.1. Blood sampling

A 10ml maternal venous blood sample will be collected for Full blood count (FBC) and reticulocyte count, iron (e.g. ferritin, sTfR) phosphate and inflammatory markers (e.g. C-reactive protein and alpha-1 glycoprotein). A filter paper sample of blood will be taken and stored for malaria PCR. In case the participants present with clinical symptoms consistent with a malaria infection, malaria parasitaemia will be sought by Rapid Diagnostic Test and the participant will be referred for treatment if the results are positive.

We will also collect a venous sample of up to 3 mL of the infant's blood for malaria RDT, microscopy, filter paper PCR and iron indices. In case the participants present with clinical symptoms consistent with a malaria infection, malaria parasitaemia will be sought by Rapid Diagnostic Test and the participant will be referred for treatment if the results are positive. Samples will be stored in order to assess other biochemical, immunological and metabolic factors associated with maternal, neonatal or infant/child health.

11.4.2. Reproductive tract microbiota

A vaginal swab will be performed. Samples will be stored in appropriate buffers for non-diagnostic assessment of vaginal and cervical microbiomes (including *Lactobacillus spp.*, *T. vaginalis*, *C. trachomatis* and *N. gonorrhoea*). In case there are any clinical indications that the participants have an infection or any other condition affecting the urogenital system, women will be referred back to the clinic and treated according to national guidelines.

11.4.3. Breastmilk

We will request to collect 2 mL of breastmilk. These samples will be stored in order to assess iron content, as well as to assess other molecular, biochemical, immunological and metabolic factors associated with maternal, neonatal or infant/child health.

11.4.4. Child anthropometry

Infant weight, length, head circumference and adiposity will be measured using established, calibrated tools.

11.4.5. Child vaccination and vitamin A supplementation status

Details of child's vaccination and vitamin A supplementation status will be recorded from medical records.

11.4.6. Infant neurodevelopment

We will measure Auditory Brainstem Responses (ABRs) while the child is sleeping. A signal (sound) is provided to the child, and the response is measured. The test is non-invasive, and only requires the fitting of electrodes on the scalp. It does not pose any risk to the child and take less than 20 minutes per child.

11.5. 3 months postpartum [± 14 days] – visit 9

All participants will be requested to return to the research clinic together with their infants for a detailed medical examination of both the mother and the baby.

11.5.1. Blood sampling

A 10ml maternal venous blood sample will be collected for Full blood count (FBC) and reticulocyte count, iron (e.g. ferritin, sTfR) phosphate and inflammatory markers (e.g. C-reactive protein and alpha-1 glycoprotein). A filter paper sample of blood will be taken and stored for malaria PCR. In case the participants present with clinical symptoms consistent with a malaria infection, malaria parasitaemia will be sought by Rapid Diagnostic Test and the participant will be referred for treatment if the results are positive

We will also collect a venous sample of up to 3 mL of the infant's blood for malaria RDT, microscopy, filter paper PCR and iron indices. In case the participants present with clinical symptoms consistent with a malaria infection, malaria parasitaemia will be sought by Rapid Diagnostic Test and the participant will be referred for treatment if the results are positive. Samples will be stored in order to assess other biochemical, immunological and metabolic factors associated with maternal, neonatal or infant/child health.

11.5.2. Child anthropometry

Infant weight, length and head circumference will be measured using established, calibrated tools.

11.5.3. Child vaccination and vitamin A supplementation status

Details of child's vaccination and vitamin A supplementation status will be recorded from medical records.

11.5.4. MATERNAL PSYCHOLOGICAL HEALTH

Maternal psychological health will be measured using the short version of Depression, Anxiety and Stress Scale (DASS-21) and EPDS. These tools provide a self-report measure of depression, anxiety and stress. Mother-child interaction will be assessed by Mother-Infant Bonding Scale (MIBS), which is a modified version of the Postpartum Bonding Questionnaire.

11.5.5. CHILD BONE HEALTH

We will take x-rays of the child's wrist and knee to identify the presence of radiological Ricketts using standard techniques. The use of x-rays is a safe and non-invasive method for the assessment of bones.

11.5.6. INFANT NEURODEVELOPMENT

We will measure structural neurodevelopment using portable low field MRI technology (see Neurocognitive Assessments Used in Infant Follow-Up section below). This technique is safe and non-invasive, and imaging will usually be performed when the infant is asleep.

11.6. 6 MONTHS POSTPARTUM [± 14 DAYS] – VISIT 10

All participants will be requested to return to the research clinic together with their infants for a detailed medical examination of both the mother and the baby.

11.6.1. BLOOD SAMPLING

A 10ml maternal venous blood sample will be collected for Full blood count (FBC) and reticulocyte count, iron (e.g. ferritin, sTfR) phosphate and inflammatory markers (e.g. C-reactive protein and alpha-1 glycoprotein). A filter paper sample of blood will be taken and stored for malaria PCR. In case the participants present with clinical symptoms consistent with a malaria infection, malaria parasitaemia will be sought by Rapid Diagnostic Test and the participant will be referred for treatment if the results are positive. For consistency with measurement of Hb (as a primary outcome).

We will also collect a venous sample of up to 3 mL of the infant's blood for malaria RDT, microscopy, filter paper PCR and iron indices. In case the participants present with clinical symptoms consistent with a malaria infection, malaria parasitaemia will be sought by Rapid Diagnostic Test and the participant will be referred for treatment if the results are positive.

Samples will be stored in order to assess other biochemical, immunological and metabolic factors associated with maternal, neonatal or infant/child health.

11.6.2. BREASTMILK

In case mothers are still breastfeeding, we will request to collect 2 mL of breastmilk. These samples will be stored in order to iron content, as well as to assess other molecular, biochemical, immunological and metabolic factors associated with maternal, neonatal or infant/child health.

11.6.3. CHILD ANTHROPOMETRY

Infant weight, length and head circumference will be measured using established, calibrated tools.

11.6.4. CHILD VACCINATION AND VITAMIN A SUPPLEMENTATION STATUS

Details of child's vaccination and vitamin A supplementation status will be recorded from medical records.

11.6.5. Infant neurodevelopment

We will measure Auditory Brainstem Responses (ABRs) while the child is sleeping. A signal (sound) is provided to the child, and the response is measured. The test is non-invasive, and only requires the fitting of electrodes on the scalp. It does not pose any risk to the child and take less than 20 minutes per child.

We will measure Event-Related Potentials (ERPs) using electroencephalography (EEG) methods (see Neurocognitive Assessments Used in Infant Follow-Up section below).

We will measure Bayley Scales of Infant Development as well as the MDAT (*see section 13. below for details*).

11.7. 9 months postpartum [± 14 days] – visit 11

All participants will be requested to return to the research clinic together with their infants for a detailed medical examination of both the mother and the baby.

11.7.1. Blood sampling

A 10ml maternal venous blood sample will be collected for Full blood count (FBC) and reticulocyte count, iron (e.g. ferritin, sTfR) phosphate and inflammatory markers (e.g. C-reactive protein and alpha- 1 glycoprotein). A filter paper sample of blood will be taken and stored for malaria PCR. In case the participants present with clinical symptoms consistent with a malaria infection, malaria parasitaemiawill be sought by Rapid Diagnostic Test and the participant will be referred for treatment if the resultsare positive.

We will also collect a venous sample of up to 3 mL of the infant's blood for malaria RDT, microscopy, filter paper PCR and iron indices. In case the participants present with clinical symptoms consistent with a malaria infection, malaria parasitaemia will be sought by Rapid Diagnostic Test and the participant will be referred for treatment if the results are positive.

Samples will be stored in order to assess other biochemical, immunological and metabolic factors associated with maternal, neonatal or infant/child health.

11.7.2. Child anthropometry

Infant weight, length and head circumference will be measured using established, calibrated tools.

11.7.3. Child vaccination and vitamin A supplementation status

Details of child's vaccination and vitamin A supplementation status will be recorded from medical records

11.8. 12 months postpartum [± 14 days] – visit 12

All participants will be requested to return to the research clinic together with their infants for a detailed medical examination of both the mother and the baby. This will be the final visit for the study.

11.8.1. Blood sampling

A 10ml maternal venous blood sample will be collected for Full blood count (FBC) and reticulocyte count, iron (e.g. ferritin, sTfR) phosphate and inflammatory markers (e.g. C-reactive protein and alpha-1 glycoprotein). A filter paper sample of blood will be taken and stored for malaria PCR. In case the participants present with clinical symptoms consistent with a malaria infection, malaria parasitaemia will be sought by Rapid Diagnostic Test and the participant will be referred for treatment if the results are positive.

We will also collect a venous sample of up to 3 mL of the infant's blood for malaria RDT, microscopy, filter paper PCR and iron indices. In case the participants present with clinical symptoms consistent with a malaria infection, malaria parasitaemia will be sought by Rapid Diagnostic Test and the participant will be referred for treatment if the results are positive.

Samples will be stored in order to assess other biochemical, immunological and metabolic factors associated with maternal, neonatal or infant/child health.

11.8.2. Reproductive tract microbiota

A vaginal swab will be performed. Samples will be stored in appropriate buffers for non-diagnostic assessment of vaginal and cervical microbiomes (including *Lactobacillus spp.*, *T. vaginalis*, *C. trachomatis* and *N. gonorrhoea*). In case there are any clinical indications that the participants have an infection or any other condition affecting the urogenital system, women will be referred back to the clinic and treated according to national guidelines.

11.8.3. Breastmilk

In case mothers are still breastfeeding, we will request to collect 2 mL of breastmilk. These samples will be stored in order to assess iron content, as well as to assess other molecular, biochemical, immunological and metabolic factors associated with maternal, neonatal or infant/child health.

11.8.4. Child anthropometry

Infant weight, length and head circumference will be measured using established, calibrated tools.

11.8.5. Child vaccination and vitamin A supplementation status

Details of child's vaccination and vitamin A supplementation status will be recorded from medical records.

11.8.6. Infant Neurodevelopment

We will measure Auditory Brainstem Responses (ABRs) while the child is sleeping. A signal (sound) is provided to the child, and the response is measured. The test is non-invasive, and only requires the fitting of electrodes on the scalp. It does not pose any risk to the child and take less than 20 minutes per child.

We will measure Event-Related Potentials (ERPs) using electroencephalography (EEG) methods (see Neurocognitive Assessments Used in Infant Follow-Up section below).

We will measure Bayley Scales of Infant Development as well as the MDAT (*see section 13. below for details*).

We will measure structural neurodevelopment using portable low field MRI technology (Hyperfine; see Neurocognitive Assessments Used in Infant Follow-Up section below). This technique is safe and non-invasive, and imaging will usually be performed when the child is asleep

11.8.7 CHILD BONE HEALTH

We will take x-rays of the child's wrist and knee to identify the presence of radiological Ricketts using standard techniques. The use of x-rays is a safe and non-invasive method for the assessment of bones.

11.9. UNSCHEDULED SICK VISIT [ANYTIME DURING STUDY FOLLOW-UP]

Participants will be encouraged to attend the research clinic when sick. The participants will be managed according to standard ANC management guidelines. Blood sample for malaria RDT (and microscopy if RDT positive) will be taken. Some of the blood will be put on filter paper for malaria PCR and for resistance testing for positive samples.

12. LABORATORY PROCEDURES

At Zomba Central Hospital, we have a laboratory with lab technologists and technicians where initial sample processing will be done. The site labs have small laboratory equipment for sample collection, separation, and storage (at -20°C). They have a microscope and area for staining of malaria and other slides before reading. Lab technologists and technicians will use SOPs for all their laboratory processing of samples. These SOPs will be developed by senior laboratory technologist and reviewed by principal investigators. Samples will be transferred for long-term storage to the College of Medicine, Blantyre campus where we have -80°C freezers. The temperature of the freezers is monitored daily, and there are backup systems in case they break down. Some samples will be exported out of the country for more advanced assays that are currently not available in Malawi (appendix 3).

12.1. FULL BLOOD COUNT

A Sysmex haematology analyser will be used for full blood counts to determine the Hb level and red cell indices, as determined by the manufacturer.

12.2. MALARIA TESTING

Testing for malaria at screening and at presentation of clinical symptoms will be done using an RDT and following the manufacture's guidelines. Whole blood will be placed on an RDT test strip and read. Positive RDT results will be confirmed by microscopy. The smears will be stained with May-Grunwald-Giemsa using standard techniques. All smears will be double read by certified laboratory technicians (each reading blinded of the other's results). Discrepant results (positive/negative or a 50% difference in the parasite counts) will be settled by a third reader. At all visits, both thin and thick blood smears will be prepared and read at a later time for the presence of asymptomatic parasitaemia. Molecular diagnosis of sub-microscopic parasitaemia and evaluation of markers of resistance to antibiotics will be sought by PCR done of dried-blood spot samples.

Blood from participants in whom parasites are detected can be further analysed for parasite clonality, levels of parasite gene expression and the abundance and characteristics of parasite proteins.

12.3. Placental histology

Placental histology will be performed using standard approaches. Placental biopsies will be fixed in formalin, embedded in paraffin and 5 mm sections cut onto glass slides. After rehydration, the tissue sections will be stained with Giemsa and/or haematoxylin, and eosin passed through graded alcohols and sealed with a coverslip. Tissue blocks will be kept for evaluation of metabolic, molecular and immunopathological parameters associated with maternal and child health.

12.3.1. Malaria evaluation

Examination, by light microscopy, will note the presence of malaria-infected erythrocytes, malaria pigment in fibrin, and malaria pigment in host leukocytes in the placental blood spaces. Placentas will be classified as uninfected; past infection (malaria pigment, no parasites); or active infections (parasites with or without malaria pigment).

12.4. Blood samples: separation and long-term storage

Blood will be drawn using standard venipuncture techniques and the serum will be separated from the blood cells as soon as possible. Samples will be allowed to clot for one hour at room temperature, centrifuged for 10 minutes at 4 °C, and serum extracted. Inflammatory markers and iron markers such as Serum C-reactive protein and alpha-1 glycoprotein, ferritin, will be analysed on Modular P800 and Modular Analytics E170 systems (Roche).

Blood will also be stored in appropriate preservatives to enable further analysis of genetic, immune, metabolic and molecular aetiologies related to anaemia, infection, developmental and other clinical outcomes in pregnancy and during the first 12 months of life. Residual whole blood (EDTA) samples from participants at baseline will be stored for future extraction of DNA and/or RNA. These DNA samples will be used for non-diagnostic studies which will have no clinical relevance to the participants. We will analyse genetic differences (single nucleotide polymorphisms) which may be linked with anaemia, nutrition, development and growth, or infection status. Samples and results of genetic analysis will not be released to anyone other than the researchers or their colleagues helping with bioinformatics analysis and will be fully de-identified.

These assays will be carried out at the Walter and Eliza Hall Institute of Medical Research, in Melbourne, Australia, or a collaborating laboratory if unavailable at that Institute; all experiments will be undertaken with full consultation and involvement of the Malawian investigators.

12.5. Vaginal and gut microbiome analysis

Vaginal and faecal microbiome analysis will be undertaken at the Walter and Eliza Hall Institute of Medical Research in Melbourne, Australia or partners, using approaches such as 16S rRNA gene sequencing or metagenomics on microbial DNA.

12.6. SUMMARY OF LABORATORY MEASUREMENTS

			Visit 0	Visit 1	Visit 4	Visit 7	Visit 8	Visit 9	Visit 10	Visit 11	Visit 12	Sick Visit Unscheduled	
			May occur at visit 1	*Week 28-34*	*36 weeks*	*Delivery*	*28 days postpartum*	*3 months postpartum*	*6 months postpartum*	*9 months postpartum*	*12 months postpartum*		
	**Sample type**	**Test**	**MEASUREMENTS on the MOTHER**										**Is the test locally available?**
**1**	**Venous blood**	Haemoglobin (Hb)	X										Yes
**2**		Malaria RDTs (mRDT)		X								X	Yes
**3**		Molecular evaluation of resistance genes if Filter paper is mRDT positive										X	No
**4**		FBC (full blood count with Hb)		X	X		X	X	X	X	X		Yes
**5**		Malaria microscopy		X	X	X	X	X	X	X	X	X	Yes
**6**		Hb (by HemoCue)		X	X								Yes
**7**		Malaria PCR		X	X	X	X	X	X	X	X		No
**8**		Parasite gene content and expression analysis (if malaria positive)		X	X	X	X	X	X	X	X		No
**9**		Phosphate levels		X	X	X	X	X	X		X		No
**10**		Ferritin (serum)		X	X		X	X	X	X	X		No
**11**		sTfR (serum)		X	X		X	X	X	X	X		No

			Visit 0	Visit 1	Visit 4	Visit 7	Visit 8	Visit 9	Visit 10	Visit 11	Visit 12	Sick Visit Unscheduled	
			May occur at visit 1	*Week 28-34*	*36 weeks*	*Delivery*	*28 days postpartum*	*3 months postpartum*	*6 months postpartum*	*9 months postpartum*	*12 months postpartum*		
**12**		Immune/metabolic markers		X	X		X	X	X	X	X		No
**13**		CRP (C-reactive protein)		X	X		X	X	X	X	X		No
**14**		Alpha-1 glycoprotein		X	X		X	X	X	X	X		No
**15**	**Reproductive tract swab**	for non-diagnostic analysis of STIs and microbiome composition			X		X				X		Yes/No
**16**	**Placental biopsy**	for Malaria immunohistological staining and examination				X							Yes/No
**17**		for immune/metabolic markers				X							No
**18**	**Placental blood**	for microscopic malaria test				X							Yes
**19**		for malaria PCR				X							No
**20**		for Immune/metabolic markers				X							No
**21**	**Breastmilk sample**	For iron and nutrition markers					X	X	X		X		No
**22**		MEASUREMENTS on the INFANT											
**23**	**Cord blood**	Malaria microscopy				X							Yes
**24**		Hb by Hemocue®				X							Yes
**25**		for Immune/metabolic markers				X							No
**26**		Full Blood Count				X							Yes
**27**		Malaria PCR				X							No

			**Visit 0**<br>***May occur at visit 1***	**Visit 1**<br>***Week 28-34***	**Visit 4**<br>***36 weeks***	**Visit 7**<br>***Delivery***	**Visit 8**<br>***28 days postpartum***	**Visit 9**<br>***3 months postpartum***	**Visit 10**<br>***6 months postpartum***	**Visit 11**<br>***9 months postpartum***	**Visit 12**<br>***12 months postpartum***	**Sick Visit Unscheduled**	
**28**		Ferritin (serum)				X							No
**29**		sTfR (serum)				X							No
**30**	**Venous blood**	malaria RDT										X	Yes
**31**		Malaria microscopy					X	X	X	X	X	X	Yes
**32**		Malaria PCR					X	X	X	X	X		No
**33**		Hb by Hemocue					X	X	X	X	X		Yes
**34**		Ferritin (serum)					X	X	X	X	X		No
**35**		sTfR (serum)					X	X	X	X	X		No
**36**		Full Blood Count					X	X	X	X	X		Yes
**37**		CRP (C-reactive protein)					X	X	X	X	X		No
**38**		Alpha-1 glycoprotein					X	X	X	X	X		No
**39**		Radiological examination of wrist and knee (x-rays)						X			X		Yes
**40**		Low field MRI (magnetic resonance imaging) of brain						X			X		Yes

FBC, full blood count; HV, home visit; Hb, haemoglobin; mRDTs, malaria rapid diagnostic tests; sTfR, serum transferrin receptor; CRP, C-reactive protein; AGP, alpha 1-acid glycoprotein; MRI, magnetic resonance imaging.

12.7. Neurocognitive assessments used in infant follow up

We will use highly sensitive assessments of memory and executive control to examine the effects of iron on children's brain development. Beyond the important effects of iron on the brain, we will also examine the neural mechanisms underlying cognitive development and how infection exposures and environmental adversity in this context may shape neural development. Event-Related Potentials (ERPs) are ideal measures of infant cognitive function because they are sensitive to, and reflective of, brain functions directly affected by iron. They are able to detect subtle yet meaningful delays that have long-term consequences[80–82]. ERPs are neurophysiologic measures that rely on recordings of the brain's electrical activity via electroencephalogram (EEG) in response to stimuli. ERPs can be recorded noninvasively at the scalp surface in minutes. They are used in routine clinical assessments in children in tertiary care hospitals across Australia, America, and Europe, typically in the investigation and diagnosis of epilepsy and seizure disorders[83,84].

Observational studies have reported significant associations between iron status in young children and ERP-derived neural correlates of memory and executive control[85–87]. For instance, Geng et al.[85] reported that two-month-old Chinese children who were iron-sufficient at birth were able to recognize their mother's voice from a stranger's voice whereas iron-deficient children were not, as indicated by the ERP late slow wave component in frontal-central and parietal-occipital locations of the scalp. Another study in American children found that, compared to iron-deficient children, iron-sufficient 9-month-olds showed a larger attentional response to their mother's face compared to a stranger's, indicated by the ERP negative component[87]. They also reported a better updating of memory for the stranger's face, indicated by the positive slow wave component. Children with iron deficiency anaemia showed similar patterns only at 12 months of age, consistent with delayed cognitive development in this group[87]. However, to date, no randomized controlled trial (RCT) of iron interventions has used ERPs.

Differences in cognitive functioning detected using ERPs early in life have been shown to be predictive of later cognition[88,89]. For instance, follow up studies of children with and without iron deficiency anaemia in the first year of life found significant differences using electrophysiology in the auditory and visual systems of the brain at four years of age and inhibitory control at ten years of age[88,89]. Results from longitudinal studies support the hypothesis that iron deficiency in early life causes hypomyelination and has long term detrimental effects on brain function, which can be detected with sensitive measures such as ERPs.

We will use ERPs to measure the impartial and direct effects of iron on brain development, by examining functions and areas of the brain which are especially sensitive to iron, to ensure we capture meaningful effects of iron supplementation on early cognition. Our study intends to build on observational ERP literature, leverage an existing rigorously controlled field RCT, to be the first study to examine the effects of antenatal high dose iron interventions on ERP-derived cognitive functions in young children.

12.7.1. Procedures for ERPs

ERPs will be measured in response to auditory and visual stimuli, recorded from 32 scalp electrode sites, using established procedures. The study centres will have a designated ERP testing room,

outfitted with the following equipment: dark curtains, an air conditioning unit, and a screen for visual stimuli.

When caregivers and their children return for the 6-month visit at which ERPs will be measured, they will be walked through each procedure in detail. They will be shown a video detailing the procedure and objectives of the research. Children will be fitted with an ERP cap (resembling a swim cap) and seated on their mothers' laps in front of a screen. Testing will begin after a period of familiarization with the cap, screen, instrument, and with the tester. We intend to spend no more than 30 minutes with each child to avoid poor data collection when a child is tired.

Visual and auditory stimuli for ERP tasks will be culturally appropriate. The order of the ERP tasks will alternate between children to ensure that the order does not bias performance. On a daily or weekly basis, recorded data will be shared with the team in Australia, where it will be processed (to separate adequate from poor EEG data) and averaged for components of interest. We will have regular online meetings between the Malawian and Australian team members to discuss and troubleshoot any issues.

In the cases where a child is not able to complete the ERP session, they will be invited to return the following day for a repeat session.

Feasibility: Electrophysiologic methods have advanced greatly in recent years, and simplifications to equipment and technique mean these methods are now portable and can be used to assess brain development even in remote, low-income settings, like Zomba. ERPs have been used in a field-based study in Pakistan and are currently being used in other resource-limited settings, such as The Gambia and South Africa. We are presently undertaking a large ERP study as part of a major trial of iron interventions in rural Bangladesh.

We will assess children's Auditory Brainstem Responses (ABRs) at birth, one month and six months of age. ABRs are reflective of maturation of the auditory pathway at various levels (e.g. acoustic nerve level to brainstem level). There is a rapid maturation of the ABR during the perinatal period that is influenced by the degree of myelination, neuronal development, synaptic function, and axonal growth in the auditory nervous system (i.e., changes we expect to be influenced by the level of iron). ABRs are already being used in our research sites in Malawi i.e. at Zomba Central Hospital and in Blantyre (Limbe Health Centre).

Behavioural child development measurements: At six and twelve months of age, we will administer the Bayley Scales of Infant and Toddler Development III, the Malawi Developmental Assessment Tool (MDAT) and a behavioural test of memory and attention. The results from the behavioural tests will be used to assess other domains of child development than those gathered using ERPs (i.e., fine and gross motor, cognitive, language, and socio-emotional development), and will be used to corroborate the ERP results. The use of the Bayley Scales and MDAT allows us to describe the findings of the interventions to policymakers, clinicians and other end-users of the trial results.

12.7.2 PORTABLE LOW FIELD MRI (HYPERFINE)

Neuroimaging offers an opportunity to assess the impact of high dose iron during gestation on child neurodevelopment. At the 3- and 12-months-of-age visits, we will obtain structural images of the brain using portable low field MRI technology (Hyperfine). Low field MRI technology for neuroimaging has been established by Hyperfine and has been adapted for low-income settings. This technique is already being used in Malawian research settings. This technique is safe and non-invasive and uses a very low

strength magnetic field compared with conventional high field MRI. The imaging will usually occur while the child is sleeping. Images will be taken in our Zomba Central Hospital research site, Malawi. Data will be uploaded and shared with our team in Melbourne, Australia and analysed by the Developmental Imaging team at the Murdoch Children's Research Institute, Melbourne. All analyses will be undertaken by staff blinded to the intervention arm and other exposures of the participant. In addition, experienced paediatric radiologists from the Royal Children's Hospital, Melbourne, will review every MRI. Children found to have an unexpected finding on MRI will be notified and referred for further evaluation in Malawi.

13. ALLOCATION OF PARTICIPANTS TO TRIAL ARMS

Participants will be randomly allocated to one of the two treatments arms with 1:1 allocation via a computer-generated randomisation schedule of randomly permuted blocks stratified by site to achieve balance between the arms within each site. The randomisation list will be generated by an independent statistician at the University of Melbourne (Australia) who will not reveal the block size until the database is ready for unblinding.

Individual participant codes will be pre-packed in envelopes, sealed and held securely at research sites. The eligible participants who have met all inclusion/exclusion criteria will receive study medication assigned to the next available randomisation number during the randomisation study visit. The study medication shall be determined after the research staff open the specific participant envelope, which will prescribe the participant's group allocation. Although the trial is open-label, midwives collecting birth data, laboratory scientists and investigators and personnel in Australia (including statisticians in Melbourne) will be blinded to the allocation code, until the database has been cleaned for analysis.

14. ASSESSMENT OF SAFETY

14.1. PRECAUTIONS IN DELIVERING IRON INTRAVENOUS FORMULATIONS

As discussed above, there remains a theoretical risk of hypersensitivity reactions associated with the infusion of FCM. For this reason, intravenous iron formulations will only be administered in health care centres where appropriate equipment and facilities are available for the treatment of any severe reactions, even though these are rare.

The participants will be monitored for signs and symptoms of hypersensitivity during and after administration of the intravenous infusion for at least 30 min and until clinically stable. The trial teams will carry the necessary equipment to provide urgent medical care.

Protocol for delivering FCM*:*

- Iron formulations will be administered as an infusion in 250 mL of 0.9% sodium chloride
- Infusion rate will be 250 mL over 15 minutes.
- The patency of the intravenous cannula will be confirmed before commencing any infusion to ensure there is no extravasation of iron, which can cause a permanent stain. Connect a 50 mL 0.9% sodium chloride flush and allow this to infuse by gravity. If the flush does not work, do not use the line.
- All women will be observed for signs of adverse reaction which may be acute or delayed (see below).
- A Clinical Officer will be available and able to reach the infusion room within 5 minutes
- Common adverse reactions to intravenous iron include:
    - Headache, dizziness

- Injection site reactions[5]
- Hypertension
- Elevated liver enzymes
- Hypophosphataemia

- Less common reactions include:
    - Nausea, abdominal pain, constipation, diarrhoea
    - Hypersensitivity including anaphylaxis
    - Paraesthesia and dysgeusia
    - Hypotension
    - Tachycardia
    - Flushing
    - Back, joint or muscle pain
    - Pruritis and urticaria
    - Pyrexia, fatigue and malaise
    - Dyspnoea and bronchospasm
    - Syncope
- A member of the study nursing staff will remain in the same room as the patient during the infusion and for the 30 minutes following patient, to ensure any serious adverse reaction is rapidly detected and that the infusion can either be stopped or other appropriate treatments given.
- Vital signs are to be monitored prior to infusion, 5 minutes after the start of the infusion, at the end of infusion, every prior to discharge, and if the participants feel unwell:
    - Heart Rate
    - Blood Pressure
    - Oxygen Saturations
    - Respiratory Rate
    - Conscious State
    - Temperature

Foetal Heart Rate will be monitored prior to infusion, at the end of infusion and prior to discharge.

[5] injection site erythema, -swelling, -burning, -pain, -bruising, -discolouration, -extravasation, -irritation, -reaction

14.2. Definitions of adverse events

According to the latest EU guideline contained in **Detailed guidance on the collection, verification and presentation of adverse event/reaction reports arising from clinical trials on medicinal products for human use ('CT-3')** 2011/C 172/01, an Adverse event is 'Any untoward medical occurrence in a patient or clinical trial subject administered a medicinal product and which does not necessarily havea causal relationship with this treatment'[71].

14.3. Recording of adverse events

14.3.1. The time period for collecting adverse events

Adverse events and adverse reactions (non-serious and serious) will be collected from the time consent is given until the participant completes the study (the final visit or withdrawal). All AEs and SAEs will be followed until resolution or stabilisation.

14.3.2. Method of capturing adverse events

At scheduled clinic visits, we will record any AE spontaneously reported by the subject and/or observed by the study staff, as well as any AEs identified by means of a standard question to the subject such as "have you experienced any health problems since the last visit/ the last questioning?". In addition, AEs will be documented from physical examination findings, clinically significant lab results or other documents (including correspondence from their primary care physician) that are relevant to participant safety.

14.3.3. Documentation of adverse events data

AEs will be recorded in the adverse event section of the CRF. For the purposes of this trial the investigators will record all adverse events, irrespective of their perceived relationship with the trial interventions, with the following exceptions:

- Conditions that are present at screening and do not deteriorate will not be considered adverse events.
- Abnormal laboratory values will not be considered adverse events unless deemed clinically significant by the investigator and documented as such (see section 17.3.9).

For each AE, a description of the event, date of onset and resolution (and if applicable time), its severity, whether it constitutes a SAE or not, any action taken (e.g. other treatment given, change to study treatment, follow-up laboratory tests) and the outcome (continuing or resolved), will be given along with the investigator's assessment of relationship to study drug. Details of changes to the dosage schedule, any other treatment given, or follow-up laboratory tests should be recorded on the appropriate pages of the CRF

All SAEs must be reported according to procedures described in section **17.4 - reporting of serious adverse events**.

If a diagnosis of the subject's condition has been made, then the diagnosis should be recorded as the adverse event. However, if a diagnosis of the subject's condition has not been made then the individual signs or symptoms should be recorded separately.

If an AE changes in frequency or severity during the study period, a new record of the AE should be recorded in the CRF.

14.3.4. Assessment of adverse event severity

The investigator will use clinical judgement to make an assessment of the severity of each AE.

14.3.5. Assessment of causality of adverse events

The investigator will use clinical judgement to assess the relationship between investigational product and the occurrence of each adverse event. Alternative causes such as natural history of the underlying diseases, concomitant therapy, other risk factors and the temporal relationship of the event to the investigational product will be considered and investigated.

The investigator will make an assessment of the causality of the AE by answering "Yes" or "No" to the question "Is there a reasonable possibility of a causal relationship between the study drug and the AE?" in the CRF.

14.3.6. Study endpoints and symptoms anaemia

Adverse events judged to be symptoms of anaemia or meeting the definition of any of the study outcome (severe anaemia) will not be recorded as adverse events unless they fulfil the criteria for an SAE or result in discontinuation of study treatment.

14.3.7. HIV-related disease

In HIV infected subjects, adverse events judged to be expected because of HIV-related disease will not be recorded as adverse events unless they fulfil the criteria for a SAE or result in discontinuation of study treatment.

14.3.8. Lack of efficacy and disease progression

Any deterioration in the subject's condition after the subject has been enrolled in the study will be discussed with / assessed by the principal investigator. Where the deterioration is considered by the principal investigator to constitute a progression of anaemia or lack of effectiveness this will not be considered an AE unless it fulfils the criteria for an SAE or results in discontinuation of study treatment. Symptoms of unexpected disease progression (as assessed by the investigator) should be recorded as AEs.

14.3.9. Abnormal laboratory values

The duplicate reporting of laboratory tests as both laboratory findings and adverse events in the CRF will be avoided. Abnormal laboratory tests results will not be reported as AEs in the CRF unless they fulfil the criteria for a SAE or result in discontinuation of study treatment. They will be evaluated in the overall safety analysis**.** If an abnormal laboratory value is associated with clinical signs and

symptoms, the sign/symptom should be reported as an AE while the associated test result is recorded in the appropriate CRF section.

14.3.10. Overdose

Use of study medication in doses in excess of that specified under the section on Trial Interventions should be recorded as an AE. An overdose without associated symptoms should be recorded as an AE of "Overdose".

14.4. Reporting of serious AEs

14.4.1. Reporting by the investigator to the study safety monitor and sponsor

The Site Principal Investigator/delegate is responsible for recording all safety events in the source document.

The investigator must report all SAEs that occur during the course of the study to the Sponsor and the ethics committee as soon as possible but within 24 hours of the investigational site becoming aware of it – in accordance with the *'Safety monitoring and reporting in clinical trials involving therapeutic goods' (November 2016)*[90]. All SAEs have to be reported, whether or not considered causally related to the investigational product or study procedure(s). All SAEs will be reported on the SAE form provided. The reporting will be made by research study clinicians.

Follow-up information on SAEs must also be reported by the investigator within the same timeframe. In case of doubt about whether an event fulfils serious criteria, the case should be reported to the safety monitor who will assess whether the event should be reported as an SAE.

At Zomba Central Hospital (ZCH), there will be a Co-investigator assigned to be the Safety Monitor. In Zomba, it will be Dr Kabeya Biselele, a consultant Obstetrician with years of experiencing working in Malawi and other African countries managing pregnant women. He has also been a co-investigator and study physician on a large Pfizer-funded trial investigating the use of azithromycin-chloroquine in the prevention of malaria in pregnancy. The safety monitor shall be available for consultation in an emergency but shall also oversee and advise on the clinical care SOPs and guidelines within the trial. He will also be part of the Trial Steering Committee and hence provide any clinic care-related advice to the trial executive decision-making body.

14.4.2. Reporting by the sponsor

The sponsor is responsible for reporting SAEs and other safety issues to the Data Monitoring Committee (DMC) and the Ethics committees in an expedited manner in accordance with the EU guidelines. The sponsor will also inform all other investigators concerned of relevant information about SAEs that could adversely affect the safety of subjects.

14.5. Data monitoring committee

An independent Data Monitoring Committee (DMC) has been set up to review on a regular basis, safety and efficacy data of the ongoing trial. The sponsor will report all SAEs and other relevant safety information to the DMC on an expedited basis. The DMC will review tables of cumulative SAEs, primary

and secondary endpoints at regular intervals. The DMC will remain blinded when presented with any interim analysis results unless the DMC judges that for safety reason the study blind should be broken.

The DMC will comprise of international experts in clinical trials, obstetrics, epidemiology and statistics.

15. DATA HANDLING AND RECORD KEEPING

Data collected from the subjects will be recorded in digital form with REDCap using electronic tablets. REDCap is hosted on infrastructure belonging to the trial's organisational team in Malawi and is subject to the same security and backup regimen as other systems (e.g. the network file servers). Datais backed up nightly to a local backup server, with a de-identified fortnightly backup taken to the servers of the team at the Walter and Eliza Hall Institute, Melbourne, Australia. Monthly backups arealso done in physical format and the trial's Data Manager will maintain an audit trail of datacreate/update/delete events that is accessible to project users who are granted permission to view it. Access to REDCap will be provided via a certified user account or (for external collaborators) via a REDCap user account created by the trial's data manager. The permissions granted to each user withineach REDCap project will be controlled by the trial team delegated this task by the Principal Investigator. REDCap has functionality that makes adding and removing users and managing user permissions straightforward. All data transmissions between users and the REDCap server are encrypted. The instructions for data entry to REDCap must be read and the training log signed prior to personnel commencing data entry on REDCap.

15.1. CASE REPORT FORMS (CRFS)

All CRFs (appendix 1) will be developed by the Investigators and implemented in REDCap by the data team. An Instruction manual on the completion of the CRF will be provided to all relevant study site personnel. It will be the responsibility of the investigators to ensure that the data in the CRFs are accurate and complete. If any data are not available, omissions will be indicated on the CRFs. The CRF will automatically sign off the user and lock the record upon completion. Relevant hard copy patient hospital files will be scanned for reference and stored digitally, securely. All CRFs will be checked by the investigator or authorised personnel for accuracy and completeness before uploading to the server. All corrections will be done by the investigator or authorised personnel; revisions will be automatically saved with tracked changes. The data will then be uploaded on the server.

15.2. DATA ENTRY AND VALIDATION

Data will be entered into REDCap database as they become available and exported weekly to the server. Hard copy data will be entered as soon as possible, and no later than 5 days, after collection. Laboratory data will be entered into the database in real time. Data query sheets will be raised and distributed by the data manager to the study team for resolution in a timely manner. Query resolutions will be stored together with the original CRFs.

15.3. Database lock

We will document the data as being clean only once the validation process is concluded. Thetreatment code will be broken and included in the database – with a subsequent database lock – onlyafter all decisions on the evaluability of the data from each individual subject have been made and documented.

16. Statistical considerations

16.1. Sample size calculations

We plan to recruit 295 women per arm, or 590 women in total when accounting for 10% drop-out at the primary outcome (at week 36 or at delivery, whichever comes first).

For our primary outcome of maternal anaemia at 36 weeks gestation or at delivery, whichever comes first, we will be able to detect a reduction in the proportion of women with anaemia (Hb<11g/dL) at 36 weeks gestation from 63% in the oral iron arm to 49% in the IV iron arm with 90% power (two-sided 5%). We assumed that the mean Hb in oral iron treated women is 10.5g/dL and the standard deviation (SD) is 1.5g/dL, thus 63%of women are expected to be anaemic (Hb<11g/dL) at 36 weeks after oral iron. We assumed that theprevalence of anaemia in IV iron treated women would be 14% lower than that of women in the oraliron arm following the pivotal FCM vs oral iron trial. The Fer-ASAP trial demonstrated a 14% reduction in absolute anaemia prevalence compared with oral iron[25].

For our key secondary outcome of birthweight, after accounting for a miscarriage and stillbirth rate of 1%, we will be able to detect a birthweight increase of 100g to 150g in the IV iron arm compared to the oral iron arm with 72% to 97% power (two-sided 5% alpha). A birth weight increase of 100g is conservatively based on achieving an improvement two thirds as large as the 150g improvement seen in women randomized to oral iron versus control with high adherence in a recent Kenyan trial of oral antenatal iron which achieved 100% adherence (and noting that the control arm in our trial still involves giving iron, potentially ameliorating some of the effect). We assume a SD of 450g.

Whilst the pre-planned sample size is 590 participants, an adaptive sample size re-estimation procedure will be undertaken as per the ‘promising zone’ methodology of Mehta and Pocock with a potential increase of up to 260 participants to the prespecified maximum of 850 participants total (including loss to follow up) once the outcomes of at least 50% of the recruited participants are obtained[90]. Details will be outlined in a stand-alone interim statistical analysis plan. In short, this procedure will involve evaluation of the conditional power during an interim analysis conducted by an independent unblinded statistician. If the conditional power falls in a pre-specified ‘promising zone’, then the sample size may be increased with the aim to achieve a conditional power of 90%. Otherwise, if the conditional power does not fall within this ‘promising zone’, then the planned sample size of 590 participants will be maintained. There will be no impact on the Type I error rate and thus no adjustment will be needed to the two-sided significance level of 5%.

Data analysis

A detailed study statistical analysis plan for the final analysis will be drawn up during the course of the

study and finalised before the unblinding of data.

16.1.1. Assessment of effectiveness

Descriptive statistics will be presented for all outcomes, by treatment group across the follow-up time points. Anaemia will be analysed using a log-binomial regression model with study participants included as a random intercept. The model will include the standard of care (oral iron) group as the reference group. The primary maternal hypothesis will be evaluated by obtaining the estimate of the risk ratio of IV iron versus standard of care (oral iron) and two-sided 95% confidence interval extracted at 36 weeks' gestation. Birthweight will be analysed by fitting a linear regression model. The primary neonatal hypothesis will be evaluated by estimating the absolute difference in birthweight between IV iron and standard care (oral iron) and two-sided 95% confidence interval.

Secondary repeated time point binary outcomes will be analysed similar to anaemia and secondary single time point continuous outcomes will be analysed similar to birthweight. Secondary, single time point binary outcomes (e.g.,low birth weight) will be analysed using a log-binomial regression model and secondary, multiple time point continuous outcomes (e.g., haemoglobin) will be analysed using a likelihood-based longitudinaldata analysis model[91]. Appropriate transformations may be applied to the variables before fitting themodel if considered skewed (e.g. ferritin). In case of non-convergence of the log-binomial models, a Poisson model with robust standard errors will be fitted instead. Exploratory subgroup analyses (e.g.,site, parity, iron deficiency) will be performed for maternal and neonatal outcomes, irrespective of their findings. The analyses models for all study outcomes will adjust for the randomisation stratification variables of site as a main effect.

16.1.2. Analysis of adverse events

Safety including adverse events, infections and clinic visits will be presented for the mothers and neonates/infants respectively. The proportion of study participants with at least one safety outcome will be compared between arms using a log-binomial regression model. In case of non-convergence, a Poisson model with robust standard errors will be fitted instead.

16.2. Analysis populations

Analyses will be undertaken on an intention-to-treat basis.

16.3. Missing data

Every effort will be made to minimise the amount of missing data in the trial. Whenever possible, information on the reason for missing data will be obtained. Additional analyses using multiple imputation will be performed to handle missing data in the primary maternal and neonate outcome. Results will be compared with the main analysis specified in Section 16.1.1 to investigate the robustness of the findings to assumptions about the missing data.

16.4. Interim analyses and criteria for termination of the trial

No formal interim analysis is planned. However, the sponsor reserves the right to temporarily suspend or prematurely discontinue this study at any time for reasons including, but not limited to, safety or ethical issues or severe non-compliance. If the sponsor determines such action is needed, it will discuss this with the investigator. When feasible, the sponsor will provide advance notification to the investigator of the impending action prior to it taking effect. The sponsor will promptly inform all the Ethics committee and regulatory bodies and provide the reason for the suspension or termination.

17. Study Management

17.1. Study monitoring

A local trial monitor such as the Research Support Centre at the College of Medicine will be appointed to perform independent monitoring of the study on behalf of the sponsor. Prior to subject enrolment,

the monitor will visit the study site to determine the adequacy of facilities, review the protocol and data collection procedures and discuss the responsibilities of the investigator and other study site personnel.

During the study, the monitor will have regular site contacts, including conducting on-site visits to:

- Confirm that the study is being performed according to the protocol, ICH GCP and applicable regulations, data are being accurately recorded in the CRFs, samples are being appropriately collected and stored, and that investigational product accountability is being performed.
- Conduct source data verification
- Confirm facilities remain acceptable
- Provide information and support to the investigators
- Evaluate study progress

Upon completion of the study the monitor will visit the study sites to verify that all CRFs are completed and collected, all data queries have been resolved and filed, conduct final accountability, reconciliation and arrangements for investigational product and verify all study site records are complete. The PI and relevant staff will be available at monitoring visits and agree to allocate sufficient time to the monitor to discuss any issues and address their resolution.

17.2. Direct access to source data/documents

The investigator agrees to allow the sponsor and/or its representatives, including the monitor, the DMC, the IRB/IEC, the regulatory body direct access to source data and other relevant documents.

17.3. Quality assurance

Authorised representatives of the sponsor, an IEC/IRB or regulatory authority may visit the study site to perform audits or inspections, including source data verification.

17.4. Training of staff

The PI is responsible for the conduct of the study at this Study Sites, including delegation of specified study responsibilities, and training of study staff. The PI will maintain a record of all individuals involved in the study (medical, nursing and other staff). The PI will ensure that all persons assisting with the trial receive the appropriate training about the protocol, the investigational product(s) and their trial-related duties and functions. All relevant study nurses and clinical personnel handling the intravenous formulation infusions will receive specific and appropriate training before the start of the trial. During the study regular spot checks will be conducted to assess the performance of study site staff members and re-training provided where necessary.

17.5. Changes to the protocol

No change will be made to the approved protocol without the agreement of the sponsor. If it is necessary for the protocol to be amended, the protocol amendment will be submitted to the IRB/IEC for approval before implementation. Any change to the informed consent form must also be approved

by the sponsor and IRB/IEC, before the revised form is used. The sponsor will distribute amendments to the PI, who in turn is responsible for the distribution of these documents to the staff at his/her study site.

17.6. FINANCING AND INSURANCE

Funding for this study will be provided by Bill & Melinda Gates Foundation through a grant to the Walter and Eliza Hall Institute of Medical Research, Melbourne, Australia. The Training and Research Unit of Excellence (TRUE) will act as sponsor and will purchase a liability insurance policy that covers this study.

17.7. STUDY DURATION

The study will start as soon as all pre-study activities and documentation are completed, and the IRB/IEC and Regulatory Authority have approved the protocol. The total duration of the trial is 30 months, which includes 12 months of recruitment, 6 month of additional follow-up phase as per Gantt chart below:

Activity	2020				2021				2022				2023				2024	
	Q1	Q2	Q3	Q4	Q1	Q2	Q3	Q4	Q1	Q2	Q3	Q4	Q1	Q2	Q3	Q4	Q1	Q2
REVAMP-TT (Main Trial)																		
Protocol, Ethics - Malawi/ Melbourne, PMPB, Import drugs																		
Open trial																		
Recruit (aim to complete within 12 months)																		
Babies born (~3 months after recruitment)																		
Babies reach 12 months of age (last outcome measured)																		
Sample analysis																		
Un-blinding and data analysis of RCT																		
Report/ manuscript of RCT																		

17.8. RECORD-KEEPING AND ARCHIVING

During the study, an Investigator Site File will be used to store documentation pertaining to the study and it will be kept in a secure location with access only to authorised individuals. It is the PI's responsibility to continuously update the Investigator file. It must be available to the Monitor during monitoring visits.

Following study closure, the investigator study file, CRFs, medical records and other source documents must be retained at the study site per regulatory obligations (two years after the last marketing application of the study drug in the European Union) and thereafter destroyed only after agreement with the sponsor. Documents with a limited shelf life (e.g. printouts on light/heat sensitive paper) will be copied and verified by signing and dating.

17.9. REPORTING AND PUBLICATION OF DATA

The results of the study will be submitted and discussed with the local and national medical authorities. They will then be presented at national and international conferences and submitted for publication in peer-reviewed journals, in accordance with the sponsor' publication policy.

18. ETHICAL CONSIDERATIONS

18.1. ETHICAL REVIEW

This study will be submitted to the following institutional review boards and independent ethics committees (IRB/IEC).

- National Health Science Research Committee (NHSRC), Malawi
- Walter and Eliza Hall Institute of Medical Research (WEHI), Melbourne, Australia

Before initiating the trial, written and dated approval/favourable opinion must be obtained from the IRB/IECs. Protocol amendments will be submitted to the IRB/IEC for approval before implementation. Progress reports, SUSAR reports and safety reports will be submitted to the IRB/IEC in accordance with local requirements.

18.2. ETHICAL CONDUCT OF THE STUDY

This study will be conducted in accordance with The International Conference on Harmonisation of Technical Requirements for Registration of Pharmaceuticals for Human Use (ICH) (ICH) guidelines for "good clinical practice" (GCP) and all applicable regulatory requirements, including, where applicable, the 1996 version of the Declaration of Helsinki.

18.3. INFORMED CONSENT

Informed consent will be obtained from each participant before conducting any study related procedure. The PI will ensure that the participant (and the subject's parent/guardian in case of assent) is given full and adequate oral and written information about the study. Information will be provided in the local language (Chichewa) of the participant (appendix 2). The participant will be given the opportunity to ask questions and allowed time to consider the information provided.

Informed consent will be documented by the use of a written consent form signed by the participant and the person who conducted the informed consent discussion. If the participant is unable to write their signature, then a thumbprint may be used. If the participant is unable to read the information her/himself, full and comprehensive information will be communicated to the participant in the presence of a witness. The witness will be an independent third party i.e. a person not connected with the conduct of the trial. The witness will sign the informed consent form to attest that the information in the consent form was accurately explained to and apparently understood by the participant and that informed consent was freely given. Each original signed informed consent will be kept on file by the investigator. A copy of the informed consent form will be provided to the participant.

Written informed consent obtained at the baseline visit will encompass all study procedures and visits up to and including the 12-month post-partum visit. We will re-discuss with mothers the extension study (mother and infant visits at 3, 6, 9 and 12-months post-partum) during the 1-month postpartum visit, although consent will have already been collected.

We will ask for informed consent for future analysis of samples for nutritional, immune and inflammatory biomarkers, molecular biomarkers, and for non-diagnostic genetic analyses that

provides insights into associations with response to the interventions, and into aspects of placental and child growth, metabolic and immune development and health. Participants prepared to enrol in the trial but who do not wish to provide consent for these extended uses of the samples will be able to decline consent for this aspect of the study.

18.4. Risks to the study participants

All efforts will be made to minimise pain, discomfort, and fear for both pregnant women and the children.

18.4.1. Blood sampling

The amount of blood taken for the study is minimal, totaling a maximum of 10 ml per visit over the study period at two timepoints during pregnancy and five timepoints postpartum (an additional 5 ml may be taken during sick visits for clinically-relevant laboratory analyses) for mothers, and <3mL per visit at four time points for infants. Blood will be collected in most cases through a venipuncture and where indicated, a finger prick. A small bruise or mild pain on the finger or the venipuncture site from where the blood is taken may develop. Only well-trained and fully qualified laboratory staff will be hired for this project. Only new disposable needles and lancets are used for the blood taking procedures, and these will be discarded immediately after their use.

18.4.2. Intravenous infusions

Participants allocated to the intravenous iron intervention arm~~s~~ will receive an intravenous infusion. A cannula will be inserted by venipuncture to allow the infusion of FCM over a period of 15 minutes. A small bruise or mild pain on the venipuncture site may develop. As discussed above, anaphylaxis is not a major risk for FCM, however there will always be an emergency resuscitation tray available during this procedure. A fully qualified health worker, with specific training in administering and managing intravenous iron infusions, will be available during the entire infusion process (and for a further 45 min after) to monitor the condition of the participant. The cannula will not be removed until the trial participant is certified as being well, with normal blood pressure and heart rate prior to discharge home.

18.5. Benefits from participating in the trial

Participants enrolled in the study will receive close monitoring of their condition for the study period. A trained clinician will see subjects at recruitment and scheduled follow-up visits, for full medical assessments. If a subject has an illness in-between scheduled visits, the subject will be seen by the study clinician and treated at the study clinic if the illness is deemed to be study related or referred to ZCH if other care is required.

18.6. Subject data protection

Participant confidentiality is strictly held in trust by the participating investigators, research staff, and the sponsoring institution and their agents. This confidentiality is extended to cover testing of biological samples and genetic tests in addition to the clinical information relating to participating

participants. The study protocol, documentation, data, and all other information generated will be held in strict confidence. No information concerning the study, or the data, will be released to any unauthorized third party, without prior written approval of the sponsoring institution. Authorised representatives of the sponsoring institution may inspect all documents and records required to be maintained by the Investigator, including but not limited to, medical records (office, clinic, or hospital) and pharmacy records for the participants in this study. The clinical study site will permit access to such records. All laboratory specimens, evaluation forms, reports and other records that leave the site will be identified only by the Participant's Unique Identification Number (UID) to maintain participant confidentiality. Clinical information will not be released without written permission of the participant, except as necessary for monitoring by HREC, IRB or regulatory agencies.

18.7. Other ethical considerations

18.7.1. Reimbursement of costs

The study will provide payment for all the study drugs, study procedures, study-related visits and all reasonable medical expenses that may be incurred as a direct result of the study. This includes compensation for transport for each study visit and will be according to the local ethics committee guidelines. Where applicable the participant will be provided with or reimbursed for, refreshments when a scheduled appointment requires them to be out of their home at mealtimes. In Malawi, in-patient hospital care is free of charge.

19. Dissemination of results

At the end of the study, the results will first be disseminated to national policymakers, government departments, academics from local research institutions and universities, NHSRC, COMREC and professional bodies in Malawi at the national stakeholders' meeting or research dissemination conferences to be held in the country. Subject to the findings of the study and based on consensus emerging at these meetings, we will support national policymakers to develop the necessary tools and guidelines to guide national and district level health providers to implement the strategy within hospital services and the health system more broadly.

Research results will also be disseminated to the global research community, technical agencies, and international government bodies via peer-reviewed journals and at international scientific fora. We will also inform other international organisations and funders of large-scale anaemia control initiatives which aim to improve anaemia at regional and local levels and are instrumental in supporting countries to implement anaemia control policies in low and middle-income countries.

20. Capacity building

Research capacity in Malawi will be enhanced by the provision of training and mentorship for all the research staff involved in the trial. By running this study, capacity in trial management will be enhanced, as will the capacity to deliver intravenous iron infusions at several health centres. The research study will strengthen the clinical skills of health workers in managing pregnant women with

anaemia. There will be 1-2 PhD candidates who will conduct their research as part of this project. Partners from the different institutions forming this research network will jointly supervise them.

In addition, there will be a post-doctoral research scientist who will work closely with the study PIs in the conduct of the study. In the process, the post-doctoral research scientist will be able to carry out various research projects from this study that will be published in peer-reviewed journals. Lastly, all study staff will undertake Good Clinical Practice (GCP) training before starting to work on the research project.

21. BUDGET

REVAMP TT Budget in US$

PERSONNEL	No.	Cost/month	Cost/year	Year 1	Year 2	Year 3	Additional Budget	Total (USD)
Site Coordinator	1	1,200	14,400	7,200	15,120	15,840	-	38,160
Data Officer	1	836	10,032	5,016	10,534	11,035	-	26,585
Clinical Officer	1	969	11,628	5,814	12,209	12,791	14,306.85	45,120.85
Research Nurse	3	850	10,200	15,300	32,130	33,660	9,543.75	90,633.75
Research Assistants	1	609	7,308	3,654	7,673	8,039	4,312.50	23,678.50
Lab Technician	1	600	7,200	7,200	7,560	7,920	-	22,680
**Total - Personnel**				**44,184**	**85,226**	**89,285**	**28,163.10**	**246,858.10**

EQUIPMENT	No.	Cost/item		Year 1	Year 2	Year 3	Additional Budget	Total (USD)
Desk-top computer	1	1,200		1,200				1,200
Laptops	1	1,542		1,542				1,542
Printer & accessories	1	1,100		1,100				1,100
Hemocue machine	3	300		900				900
Small clinical equipment	1	2,000		2,000		2,000		4,000
**Total - Equipment**				**6,742**	**-**	**2,000**		**8,742**

CONSUMABLES				Year 1	Year 2	Year 3	Additional Budget	Total (USD)
Lab consumables				375	1,250	875	4,833.45	7,333.45
RDT cuvettes				2,000	3,000	1,000	2,328.75	8,328.75
Fuel - bikes & Car hire				2,340	3,120	3,120	1,638.75	10,218.75
Maintenance & insurance				700	770	840	1,794.00	4,104.00

Patient reimbursement		6,240	6,240	1,897.50	17,497.50
Consumables - Office					
Communications & internet	1,200	1,260	1,323	1,983.75	17,497.50
Stationary	1,200	1,320	1,584	1,207.50	5,311.5
**TOTAL - Consumables**	**10,935**	**16,960**	**14,982**	**15,683.70**	**58,560.70**
**Trial Management**					
Monitoring	2,000	2,000	2,000	1,035.00	7,035.00
Trial Insurance	12,000			2,156.25	14,156.25
Ethics & regulatory fee	6,150	1,000	1,000	172.50	8,322.50
Training and Results Dissemination				1,354.13	1,354.13
**TOTAL - Trial Management**	**20,150**	**3,000**	**3,000**	**4,717.88**	**30,867.88**
**Overheads (10%)**	**8,201**	**10,519**	**10,927**	**4,856.47**	**34,502.47**
**GRAND TOTAL ($)**	**90,212**	**115,705**	**120,193**	**53,421.15**	**379,532.15**

Additional budget notes:

- **Budget is for a sub-set of 200 participants (mother-infant pairs) who will participate in the REVAMP-Bone Substudy.**
- **Infant neurodevelopment at 12 months of age (brain structure parameters measured by low field portable MRI technology) in a sub-set of 60 infants (REVAMP-Neuro Sub study).**
- **Prevalence of radiological rickets will be done at 6 and 12 months of age**
- **Equipment for MRI (Hyperfine) are a donation thus cost not included in the budget**

22. BUDGET JUSTIFICATION

We requested a total of $326,111 to conduct the study as described above. The majority of the funds ($218,695) will go towards staff renumeration. The staff will be involved in running daily study activities. A total of $8,742 will be directed towards procurement of office equipment which includes: desktop and laptop for data collection and archiving, printers for printing study related materials including CRFs, SOPs and procurement of other clinical equipments such as HemoCue machine for Hb measurements.

A total of $42,877 will be used for the procurement of consumables. This will include lab supplies such as Hb cuvettes, specimen bottles, reagents that will be required for sample processing and analysis. Participants were also given money for transport reimbursement and food as required by the ethics guidelines in all study visits. The study site will also need airtime as well as internet for effective communication locally as well as with international Principal Investigators.

We will need $26,150 for trial management. This money will be used to pay study monitors who undertake important tasks of ensuring that study is implemented in accordance with the declaration of Helsinki thereby ensuring participants'safety. The trail will have two insurances for participants as well as medical malpractice insurance. Payments will also be made to NHSRC for reviewing the protocol and Pharmacy Medicine and Poison Board annually for monitoring the study. Finally, project overheads will be used for study administrative support

23. Bibliography

1 Mwangi MN, Roth JM, Smit MR, *et al.* Effect of daily antenatal iron supplementation on plasmodium infection in kenyan women: A randomized clinical trial. *JAMA - J Am Med Assoc* 2015; **314**: 1009–20.

2 Radhika AG, Sharma AK, Perumal V, *et al.* Parenteral Versus Oral Iron for Treatment of Iron Deficiency Anaemia During Pregnancy and post-partum: A Systematic Review. *J Obstet Gynecol India* 2019; **69**: 13–24.

3 Qassim A, Grivell RM, Henry A, Kidson-Gerber G, Shand A, Grzeskowiak LE. Intravenous or oral iron for treating iron deficiency anaemia during pregnancy: systematic review and meta-analysis. *Med J Aust* 2019; **211**: 367–73.

4 Lewkowitz AK, Gupta A, Simon L, *et al.* Intravenous compared with oral iron for the treatment of iron-deficiency anemia in pregnancy: a systematic review and meta-analysis. *J Perinatol* 2019; **39**: 519–32.

5 Pollock RF, Muduma G. A systematic literature review and indirect comparison of iron isomaltoside and ferric carboxymaltose in iron deficiency anemia after failure or intolerance of oral iron treatment. *Expert Rev Hematol* 2019; **12**: 129–36.

6 Wolf M, Rubin J, Achebe M, *et al.* Effects of Iron Isomaltoside vs Ferric Carboxymaltose on Hypophosphatemia in Iron-Deficiency Anemia: Two Randomized Clinical Trials. *JAMA - J Am Med Assoc* 2020; **323**: 432–43.

7 Schaefer B, Würtinger P, Finkenstedt A, *et al.* Choice of high-dose intravenous iron preparation determines hypophosphatemia risk. *PLoS One* 2016; **11**: 1–11.

8 Detlie TE, Lindstrøm JC, Jahnsen ME, *et al.* Incidence of hypophosphatemia in patients with inflammatory bowel disease treated with ferric carboxymaltose or iron isomaltoside. *Aliment Pharmacol Ther* 2019; **50**: 397–406.

9 WHO 2011. The global prevalence of anaemia in 2011. *Who* 2011: 1–48.

10 Haider BA, Olofin I, Wang M, Spiegelman D, Ezzati M, Fawzi WW. Anaemia, prenatal iron use, and risk of adverse pregnancy outcomes: Systematic review and meta-analysis. *BMJ* 2013; **347**: 1–19.

11 Nair M, Knight M, Kurinczuk J. Risk factors and newborn outcomes associated with maternal deaths in the UK from 2009 to 2013: a national case-control study. *BJOG An Int J Obstet Gynaecol* 2016; **123**: 1654–62.

12 Drassinower D, Lavery JA, Friedman AM, Levin HI, Običan SG, Ananth C V. The effect of maternal haematocrit on offspring iq at 4 and 7 years of age: A secondary analysis. *BJOG An Int J Obstet Gynaecol* 2016; **123**: 2087–93.

13 WHO. Global nutrition targets 2025: Anaemia Policy Brief. 2012: 1–7.

14 WHO: World Health Organization. Low Birth Weight Policy Brief. *Low Birth Weight Policy Br* 2014: 1.

15 Beck S, Wojdyla D, Say L, *et al.* The worldwide incidence of preterm birth: a systematic review of maternal mortality and morbidity. *Bull World Health Organ* 2010; **88**: 31–8.

16 Pena-Rosas JP, De-Regil LM, Garcia-Casal MN, Dowswell T. Daily oral iron supplementation

during pregnancy. *Cochrane database Syst Rev* 2015: CD004736.

17 WHO. Iron and Folate Supplementation: Intergrated Management of Pregnancy and Childbirth (IMPAC). Geneva, 2006 www.who.int/making_pregnancy_safer/publications/en.

18 WHO. Essential Nutrition Actions: improving maternal, newborn, infant and young child health and nutrition. Geneva, 2013.

19 Low MSY, Speedy J, Styles CE, De-Regil LM, Pasricha S-R. Daily iron supplementation for improving anaemia, iron status and health in menstruating women. *Cochrane database Syst Rev* 2016; **4**: CD009747.

20 Bah A, Pasricha S-R, Jallow MW, *et al.* Serum Hepcidin Concentrations Decline during Pregnancy and May Identify Iron Deficiency: Analysis of a Longitudinal Pregnancy Cohort in The Gambia. *J Nutr* 2017; **147**: 1131–7.

21 Rognoni C, Venturini S, Meregaglia M, Marmifero M, Tarricone R. Efficacy and Safety of Ferric Carboxymaltose and Other Formulations in Iron-Deficient Patients: A Systematic Review and Network Meta-analysis of Randomised Controlled Trials. *Clin Drug Investig* 2016; **36**: 177–94.

22 Keating GM. Ferric carboxymaltose: a review of its use in iron deficiency. *Drugs* 2015; **75**: 101–27.

23 Friedrisch JR, Cançado RD. Intravenous ferric carboxymaltose for the treatment of iron deficiency anemia. *Rev Bras Hematol Hemoter* 2015; **37**: 400–5.

24 Moore RA, Gaskell H, Rose P, Allan J. Meta-analysis of efficacy and safety of intravenous ferric carboxymaltose (Ferinject) from clinical trial reports and published trial data. *BMC Blood Disord* 2011; **11**: 4.

25 Breymann C, Milman N, Mezzacasa A, Bernard R, Dudenhausen J. Ferric carboxymaltose vs. oral iron in the treatment of pregnant women with iron deficiency anemia: An international, open-label, randomized controlled trial (FER-ASAP). *J Perinat Med* 2017; **45**: 443–53.

26 Van Wyck DB, Martens MG, Seid MH, Baker JB, Mangione A. Intravenous Ferric Carboxymaltose Compared with Oral Iron in the Treatment of Postpartum Anemia. *Obstet Gynecol* 2007; **110**: 267–78.

27 Kulnigg S, Stoinov S, Simanenkov V, *et al.* A novel intravenous iron formulation for treatment of anemia in inflammatory bowel disease: The ferric carboxymaltose (FERINJECT®) randomized controlled trial. *Am J Gastroenterol* 2008; **103**: 1182–92.

28 Evstatiev R, Marteau P, Iqbal T, *et al.* FERGIcor, a randomized controlled trial on ferric carboxymaltose for iron deficiency anemia in inflammatory bowel disease. *Gastroenterology* 2011; **141**: 846-853.e2.

29 Beigel F, Löhr B, Laubender RP, *et al.* Iron status and analysis of efficacy and safety of ferric carboxymaltose treatment in patients with inflammatory bowel disease. *Digestion* 2012; **85**: 47–54.

30 Befrits R, Wikman O, Blomquist L, *et al.* Anemia and iron deficiency in inflammatory bowel disease: An open, prospective, observational study on diagnosis, treatment with ferric carboxymaltose and quality of life. *Scand J Gastroenterol* 2013; **48**: 1027–32.

31 Evstatiev R, Alexeeva O, Bokemeyer B, *et al.* Ferric carboxymaltose prevents recurrence of anemia in patients with inflammatory bowel disease. *Clin Gastroenterol Hepatol* 2013; **11**: 269–77.

32 Laass MW, Straub S, Chainey S, Virgin G, Cushway T. Effectiveness and safety of ferric carboxymaltose treatment in children and adolescents with inflammatory bowel disease and other gastrointestinal diseases. *BMC Gastroenterol* 2014; **14**: 1–8.

33 Cortes X, Borrás-Blasco J, Molés JR, Boscá M, Cortés E. Safety of ferric carboxymaltose immediately after infliximab administration, in a single session, in inflammatory bowel disease patients with iron deficiency: A pilot study. *PLoS One* 2015; **10**: 1–11.

34 Szczech LA, Bregman DB, Harrington RA, *et al.* Randomized Evaluation of efficacy and safety of ferric carboxymaltose in Patients with iron deficiency Anaemia and Impaired Renal function (REPAIR-IDA): Rationale and study design. *Nephrol Dial Transplant* 2010; **25**: 2368–75.

35 Ponikowski P, Filippatos G, Colet JC, *et al.* The impact of intravenous ferric carboxymaltose on renal function: An analysis of the FAIR-HF study. *Eur J Heart Fail* 2015; **17**: 329–39.

36 Anker SD, Colet JC, Filippatos G, *et al.* Ferric carboxymaltose in patients with heart failure and iron deficiency. *N Engl J Med* 2009; **361**: 2436–48.

37 Bisbe E, Garca-Erce JA, Díez-Lobo AI, Muñoz M. A multicentre comparative study on the efficacy of intravenous ferric carboxymaltose and iron sucrose for correcting preoperative anaemia in patients undergoing major elective surgery. *Br J Anaesth* 2011; **107**: 477–8.

38 Bernabeu-Wittel M, Aparicio R, Romero M, *et al.* Ferric carboxymaltose with or without erythropoietin for the prevention of red-cell transfusions in the perioperative period of osteoporotic hip fractures: A randomized contolled trial. the PAHFRAC-01 project. *BMC Musculoskelet Disord* 2012; **13**: 27.

39 Borstlap WAA, Buskens CJ, Tytgat KMAJ, *et al.* Multicentre randomized controlled trial comparing ferric(III)carboxymaltose infusion with oral iron supplementation in the treatment of preoperative anaemia in colorectal cancer patients. *BMC Surg* 2015; **15**: 1–7.

40 Nores J. The Efficacy of IV Ferric Carboxymaltose in the Perioperative Management of Moderate to Severe Iron Deficiency Anemia. *J Minim Invasive Gynecol* 2015; **22**: S211–2.

41 Khalafallah AA, Yan C, Al-Badri R, *et al.* Intravenous ferric carboxymaltose versus standard care in the management of postoperative anaemia: a prospective, open-label, randomised controlled trial. *Lancet Haematol* 2016; **3**: e415–25.

42 Froessler B, Collingwood J, Hodyl NA, Dekker G. Intravenous ferric carboxymaltose for anaemia in pregnancy. *BMC Pregnancy Childbirth* 2014; **14**: 1–5.

43 Zeba D, Khanam PA, Ahamed M, Khair MA. Intravenous Iron Treatment in Pregnancy: Ferric Carboxymaltose for Correction of Iron Deficiency Anaemia. *Faridpur Med Coll J* 2017; **12**: 54–7.

44 Mishra V, Gandhi K, Roy R, Hokabaj S, Shah KN. Role of Intravenous Ferric Carboxy-maltose in Pregnant Women with Iron Deficiency Anaemia. *J Nepal Health Res Counc* 2017; **15**: 96–9.

45 Christoph P, Schuller C, Studer H, Irion O, De Tejada BM, Surbek D. Intravenous iron treatment in pregnancy: Comparison of high-dose ferric carboxymaltose vs. iron sucrose. *J Perinat Med* 2012; **40**: 469–74.

46 Pavord S, Daru J, Prasannan N, Robinson S, Stanworth S, Girling J. UK guidelines on the management of iron deficiency in pregnancy. *Br J Haematol* 2020; **188**: 819–30.

47 National Health Service. Ferinject in Pregnancy and the Postpartum Period. 2018. https://www.meht.nhs.uk/EasysiteWeb/getresource.axd?AssetID=14692&type=Full&servicetype=Attachment.

48 Achebe MM, Gafter-Gvili A. How I treat anemia in pregnancy: Iron, cobalamin, and folate. *Blood* 2017; **129**: 940–9.

49 Kalra PA. Introducing iron isomaltoside 1000 (Monofer®) development rationale and clinical experience. *NDT Plus* 2011; **4**: 10–3.

50 Food and Drug Administration. Product Quality Review - Monoferric. *Approv Lett* 2018: 1–45.

51 Administration TG. AusPAR Attachment 2 Extract from the Clinical Evaluation Report for mirabegron Proprietary Product Name: Betmiga. 2013.

52 Monograph P, Patient I, Information M. Monoferric Product Monograph. *Pfizer Canada* 2018; : 1–37.

53 Jahn MR, Andreasen HB, Fütterer S, *et al.* A comparative study of the physicochemical properties of iron isomaltoside 1000 (Monofer®), a new intravenous iron preparation and its clinical implications. *Eur J Pharm Biopharm* 2011; **78**: 480–91.

54 Wikström B, Bhandari S, Barany P, *et al.* Iron isomaltoside 1000: a new intravenous iron for treating iron deficiency in chronic kidney disease. *J Nephrol* 2011; **24**: 589–96.

55 Tomer A, Amir B, Alon G, Hefziba G, Leonard L, Anat GG. The safety of intravenous iron preparations: Systematic review and meta-analysis. *Mayo Clin Proc* 2015; **90**: 12–23.

56 Food and Drug Administration. Ferric Carboxymaltose(Injectafer) - Highlights of prescribing information. 2013: 1–11.

57 Calvet X, Ruíz MÀ, Dosal A, *et al.* Cost-Minimization Analysis Favours Intravenous Ferric Carboxymaltose over Ferric Sucrose for the Ambulatory Treatment of Severe Iron Deficiency. *PLoS One* 2012; **7**: 7–11.

58 Kalra PA, Bhandari S, Saxena S, *et al.* A randomized trial of iron isomaltoside 1000 versus oral iron in non-dialysis-dependent chronic kidney disease patients with anaemia. *Nephrol Dial Transplant* 2016; **31**: 646–55.

59 Bhandari S, Kalra PA, Kothari J, *et al.* A randomized, open-label trial of iron isomaltoside 1000 (Monofer®) compared with iron sucrose (Venofer®) as maintenance therapy in haemodialysis patients. *Nephrol Dial Transplant* 2015; **30**: 1577–89.

60 Reinisch W, Staun M, Tandon RK, *et al.* A randomized, open-label, non-inferiority study of intravenous iron isomaltoside 1,000 (monofer) compared with oral iron for treatment of anemia in ibd (proceed). *Am J Gastroenterol* 2013; **108**: 1877–88.

61 Reinisch W, Altorjay I, Zsigmond F, *et al.* A 1-year trial of repeated high-dose intravenous iron isomaltoside 1000 to maintain stable hemoglobin levels in inflammatory bowel disease. *Scand J Gastroenterol* 2015; **50**: 1226–33.

62 Dahlerup JF, Jacobsen BA, van der Woude J, Bark LÅ, Thomsen LL, Lindgren S. High-dose fast infusion of parenteral iron isomaltoside is efficacious in inflammatory bowel disease patients with iron-deficiency anaemia without profound changes in phosphate or fibroblast growth factor 23. *Scand J Gastroenterol* 2016; **51**: 1332–8.

63 Frigstad S, Rannem T, Hellstrom P, Hammarlund P, Bonderup O. A Scandinavian prospective observational study of iron isomaltoside 1000 treatment: clinical practice and outcomes in iron deficiency anaemia in patients with IBD [poster P481]. In: 10th Congress of the European Crohn's and Colitis Organisation (ECCO). 2014: 320.

64 Birgegård G, Henry D, Thomsen LAM. (MONOFER®), Intravenous Iron Isomaltoside 1000

Oral, As Mono Therapy in Comparison with Non-Myeloid, Iron Sulphate in Patients with Chemotherapy, Malignancies Associated with (Cia), Induced Anaemia. In: Supportive Care in Cancer. 2015: 1–388.

65 Johansson PI, Rasmussen AS, Thomsen LL. Intravenous iron isomaltoside 1000 (Monofer®) reduces postoperative anaemia in preoperatively non-anaemic patients undergoing elective or subacute coronary artery bypass graft, valve replacement or a combination thereof: A randomized double-blind placebo-. *Vox Sang* 2015; **109**: 257–66.

66 Hildebrandt PR, Bruun NE, Nielsen OW, *et al.* Effects of administration of iron isomaltoside 1000 in patients with chronic heart failure. A pilot study. *Transfus Altern Transfus Med* 2010; **11**: 131–7.

67 Holm C, Thomsen LL, Norgaard A, Langhoff-Roos J. Intravenous iron isomaltoside 1000 administered by high single-dose infusions or standard medical care for the treatment of fatigue in women after postpartum haemorrhage: Study protocol for a randomised controlled trial. *Trials* 2015; **16**: 1–8.

68 Frigstad SO, Haaber A, Bajor A, *et al.* The NIMO Scandinavian Study: A Prospective Observational Study of Iron Isomaltoside Treatment in Patients with Iron Deficiency. *Gastroenterol Res Pract* 2017; **2017**. DOI:10.1155/2017/4585164.

69 Bhandari S, Kalra PA, Berkowitz M, Belo D, Thomsen LL, Wolf M. Safety and efficacy of iron isomaltoside 1000/ferric derisomaltose versus iron sucrose in patients with chronic kidney disease: the FERWON-NEPHRO randomized, open-label, comparative trial. *Nephrol Dial Transplant* 2020: 1–10.

70 Aksan A, Dignass A, Stein J. Letter: An Economic Evaluation of Iron Isomaltoside 1000 Versus Ferric Carboxymaltose in Patients with Inflammatory Bowel Disease and Iron Deficiency Anemia in Denmark. *Adv Ther* 2019; **36**: 1817–20.

71 European Commission. Communication from the Commission - Detailed guidance on the collection, verification and presentation of adverse event/reaction reports arising from clinical trials on medicinal products for human use (‘CT-3’) (2011/C 172/01). *Online* 2011; **C**: 1–19.

72 Knochel JP. The Pathophysiology and Clinical Characteristics of Severe Hypophosphatemia. *Arch Intern Med* 1977; **137**: 203–20.

73 Milman N. Postpartum anemia II: Prevention and treatment. *Ann Hematol* 2012; **91**: 143–54.

74 Milman N. Postpartum anemia I: Definition, prevalence, causes, and consequences. *Ann Hematol* 2011; **90**: 1247–53.

75 Azami M, Badfar G, Khalighi Z, *et al.* The association between anemia and postpartum depression: A systematic review and meta-analysis. *Casp J Intern Med* 2019; **10**: 115–24.

76 Khalafallah AA, Dennis AE. Iron deficiency anaemia in pregnancy and postpartum: Pathophysiology and effect of oral versus intravenous iron therapy. *J Pregnancy* 2012; **2012**. DOI:10.1155/2012/630519.

77 Wesström J. Safety of intravenous iron isomaltoside for iron deficiency and iron deficiency anemia in pregnancy. *Arch Gynecol Obstet* 2020; **301**: 1127–31.

78 Kimani J, Phiri K, Kamiza S, *et al.* Efficacy and Safety of Azithromycin-Chloroquine versus Sulfadoxine-Pyrimethamine for Intermittent Preventive Treatment of Plasmodium Falciparum Malaria Infection in Pregnant Women in Africa: An Open-Label, Randomized Trial. *PLoS One* 2016; **11**: e0157045.

79 Government of Malawi, MOH, NMCP. Guidelines for the treatment of malaria in Malawi, 4th Edition, July 2013. 2013.

80 Rose SA, Feldman JF, Wallace IF, McCarton C. Information Processing at 1 Year: Relation to Birth Status and Developmental Outcome During the First 5 Years. *Dev Psychol* 1991; **27**: 723–37.

81 Bornstein MH, Sigman MD. Continuity in Mental Development from Infancy. *Child Dev* 1986; **57**: 251–74.

82 deRegnier R-A, Nelson CA, Thomas KM, Wewerka S, Georgieff MK. Neurophysiologic evaluation of auditory recognition memory in healthy newborn infants and infants of diabetic mothers. *J Pediatr* 2000; **137**: 777–84.

83 Nuwer MR, Comi G, Emerson R, *et al.* IFCN standards for digital recording of clinical EEG. *Electroencephalogr Clin Neurophysiol* 1998; **106**: 259–61.

84 Monif M, Seneviratne U. Clinical factors associated with the yield of routine outpatient scalp electroencephalograms: A retrospective analysis from a tertiary hospital. *J Clin Neurosci* 2017; **45**: 110–4.

85 Geng F, Mai X, Zhan J, *et al.* Impact of Fetal-Neonatal Iron Deficiency on Recognition Memory at 2 Months of Age. *J Pediatr* 2015; **167**: 1226–32.

86 Monga M, Walia V, Gandhi A, Chandra J, Sharma S. Effect of iron deficiency anemia on visual evoked potential of growing children. *Brain Dev* 2010; **32**: 213–6.

87 Burden MJ, Westerlund AJ, Armony-Sivan R, *et al.* An Event-Related Potential Study of Attention and Recognition Memory in Infants with Iron-Deficiency Anemia. *Pediatrics* 2007; **120**: e336–45.

88 Algarín C, Peirano P, Garrido M, Pizarro F, Lozoff B. Iron deficiency anemia in infancy: Long-lasting effects on auditory and visual system functioning. *Pediatr Res* 2003; **53**: 217–23.

89 Algarín C, Nelson CA, Peirano P, Westerlund A, Reyes S, Lozoff B. Iron-deficiency anemia in infancy and poorer cognitive inhibitory control at age 10 years. *Dev Med Child Neurol* 2013; **55**: 453–8.

90 Mehta CR, Pocock SJ. Adaptive increase in sample size when interim results are promising: a practical guide with examples. Stat Med. 2011;30(28):3267-84.

91 Council NH and MR, National Health and Medical Research Council. Guidance: Safety monitoring and reporting in clinical trials involving therapeutic goods. 2016: 27.

92 Zeger SL. Indian Statistical Institute Longitudinal Data Analysis of Continuous and Discrete Responses for Pre-Post Designs Author (s): Kung-Yee Liang and Scott L. Zeger Source: Sankhyā: The Indian Journal of Statistics, Series B (1960-2002), Vol . 62, No. 2016; **62**: 134–48.

24. APPENDIX (ICFs and Case Report Forms (CRFs))

24.1.1. ICF – English version (with track changes)

24.1.2. ICF – English version (clean)

24.1.3. ICF – Chichewa version (with track changes)

24.1.4. ICF – Chichewa version (clean)

24.1.5. Screening form

24.1.6. Screening (post-malaria treatment)

24.1.7. PICF process for enrolment

24.1.8. Demographics

24.1.9. Obstetric history

24.1.10. Maternal physical examination

24.1.11. Randomisation and product administration

24.1.12. Locator details (for participant)

24.1.13. Concomitant medication during pregnancy

24.1.14. Physical examination at delivery

24.1.15. Pregnancy outcome

24.1.16. Neonatal outcome

24.1.17. Infant physical examination

24.1.18. EPDS form

24.1.19. MIBS form

24.1.20. Infant vaccination history

24.1.21. Infant morbidity questionnaire

24.1.22. Adverse events form

24.1.23. End of study form

24.1.24. Informed consent cover page

24.1.25. Placental histopathology form

24.1.26. Household food insecurity form

24.1.27. Child dietary diversity

24.1.28. Child stimulatory care

24.1.29. Bayley's assessment

24.1.30. Material Transfer Agreement (MTA)

Screening ID									

Study ID									

REVAMP-TT STUDY
PARTICIPANT INFORMATION SHEET AND INFORMED CONSENT FORM (ICF)
ENGLISH VERSION

COVER SHEET

**Participant Name:**	
**Participants age at time of informed consent:**	
**Name of husband or guardian present during the consenting process (optional):**	
**Date of informed consent discussion:**	
**Date informed consent obtained:**	
**Time (24 hrs clock format):**	
**Name of study staff/ person completing informed consent discussion (and this coversheet):**	
**Is the potential volunteer literate?:**	Yes
	☐ No (If no, an impartial witness must be present during the entire informed consent discussion. Refer to informed consent SOP for specific instructions.
**Was a copy of ICF given to the participant?:**	☐ Yes
	☐ No, refused to accept

Screening ID									

Study ID									

**Title**	Randomized controlled trial of the effect of intravenous iron on anaemia in Malawian pregnant women in the third trimester (REVAMP-TT)
**Project Sponsor**	Training and Research Unit of Excellence (TRUE),College of Medicine, University of Malawi
**Co - Principal Investigators**	1. Professor Kamija Phiri College of Medicine, BlantyreMalawi Phone: +265999957048<br>2. Dr. Sant-Rayn Pasricha Walter and Eliza Hall Institute of MedicalResearch, Melbourne, Australia Phone: +61393452618
**NHSRC Contacts**	If you want to know more about your rights as a research participant you can contact the NHSRC Secretariat, at the ministry of health on the following details, Phone: +2651 726422/418. Email: mohdoccenter@gmail.com.
**Locations**	Health centres in Zomba District, Malawi

Introduction

This is an invitation for you to take part in this research project evaluating methods for preventing anaemia in pregnant women. This *Participant Information Sheet/Consent Form* tells you about the research project. It explains the tests and treatments involved. Knowing what is involved will help you decide if you want to take part in the research. If you choose totake part in the research project, you will be asked to sign the consent section at the end. By signing this consent form, you are telling us that you:

1. Understand what you have read and/or heard
2. Consent to taking part in the research project
3. Consent for the tests and treatments that are described
4. Consent to the use of your personal and health information as described. You will be given a copy of this Participant Information and Consent Form to keep.

You will be free to withdraw from participating in this study at any time during the study without

Screening ID									

Study ID									

giving a reason. If you do not want to take part now, or in the future, and if you withdraw at any point from the study, this will not affect the standard of care you will receiveat the hospital.

Purpose of this study

Pregnant women living in Malawi have a high chance of developing anaemia during pregnancy. Antenatal anaemia may contribute to risks for both mother and child. The standardapproach for treating anaemia is oral iron. An intravenous-iron drugs, Ferric Carboxymaltose is now available in Western countries. This drug provides the opportunity to give high doses of iron in a single 15-minute infusion. This drug is currently costly; however, we believe it works better than oral iron for improving anaemia when we are trying to rescue anaemia late in pregnancy. The aim of this study is to determine the effectiveness and safety of intravenousiron administration during the third trimester of pregnancy – given as Ferric Carboxymaltose (FCM) – when compared with oral iron in improving maternal (especially anaemia) and infant (growth, birth weight and development up to 12 months postpartum) outcomes.

Study procedure

We will be asking pregnant women, such as yourself, in the third trimester of their pregnanciesand with signs of having anaemia to participate in this study. They will be randomly allocatedto one of two study arms: (a) intravenous iron FCM; (b) oral iron. All the women enrolled in this study will receive routine care, which may include malaria prevention drugs, if scheduled. Women in the intravenous iron intervention group will receive intravenous FCM 1000mg for body weight ≥ 50kg, or 20mg/kg for bodyweight <50kg, once during the third trimester once during the third trimester. The control group will receive oral iron 200mg Ferrous Sulphate (approx. 65 mg elemental iron) twice daily for the duration of pregnancy. All participants willreceive Sulfadoxine-Pyrimethamine (SP) as IPTp according to national guidelines if the threedoses of recommended IPTp have not been already achieved.

If you choose to participate in this study, we will ask you some questions about yourpsychological wellbeing; we will perform medical examinations; and we will collect a small amount of blood (2 teaspoons/visit) and other biological samples. We will ask you to return forvisits at 6 scheduled times: at 36 weeks' gestation, and 1 month, 3 months, 6 months, 9 months, and 12 months post-delivery. At the 36-week gestation visit, we will perform check-ups on you, ask some questions on your health and psychological wellbeing, and collect some blood. At this same visit, we will do a vaginal swab to test for the types of bacteria that may be presentin your reproductive tract. This may cause you some discomfort. There may be a need for you and your sexual partner(s) to be treatedif any inadvertent clinical findings are detected that could have health implications for you andyour partner(s) (i.e., STDs). Lastly, on this visit, we will ask to collect a sample of your stool. After your delivery, a member of our team will come to collect blood samples from the cord and placenta, as well as collect

Screening ID									

Study ID									

a small piece of the placenta for further tests that are meant to provide us with information with regards to the health and well-being of your baby. We will also record Apgar scores, and the baby will have a full physical examination, including measurement of birth weight, length, head circumference, auditory brainstem responses, and details of any congenital malformations. We will also take a small sample of your baby's firststool for analysis. We will ask you to come back at 1 month, 3 months, 6 months, 9 months, and 12 months after the birth, when we will check to see how you are feeling, collect blood samples, and measure and weigh your baby. In addition, during the 1-month, 6-month and 12-month post-delivery visits, we will take a small amount of expressed breast milk (2ml) for iron indices testing and other general well-being and nutritional parameters. We will also collect stool samples from both you and your child for analysis on these visits.

You are also asked to come to the study clinic anytime you are sick, for a check-up and blood test.

Allocating you to a treatment

It is essential that you understand that you will be assigned to one of the treatment arms totally by chance. Before this assignment, neither you nor the study staff will know to which group you will be assigned. You have equal chances of being assigned to either of the treatments described above. Once assigned, you will receive detailed information about the treatment you will receive.

If you are assigned to the intravenous iron intervention arm, you will receive the drug through a small drip inserted into your arm. If you receive oral iron, you will be given a course of tablets from your health worker, plus information on how to take the drug.

What happens after receiving treatment?

After we give you the treatment on that first visit, we will ask you to come to the study clinic at every scheduled study visit for study-specific procedures. Visits are scheduled for 36-weeks gestation, 1-month, 3-month, 6-month, 9-months, and 12-month post-delivery. In addition, we would encourage you to come to the study clinic whenever you are sick. You are also encouraged to go for delivery at the facility that has been allocated to you. You must never share the medicine with your fellow pregnant women or anyone.

Are there any side effects of the treatment?

All medicines have side effects. There is a rare chance that you may experience mild dizziness, joint aches, and headache, which usually subsidise without medications. Oral Iron may cause constipation, diarrhoea, and stomach aches, and is likely to turn your faeces black – but this is not dangerous. Chances are also very rare that one might experience potentially life-threatening allergic reactions immediately after given intravenous iron which might include symptoms like

Screening ID									

Study ID									

skin rash, nausea, low blood pressure and difficulty breathing. However, our clinical team is well trained to handle those conditions if happens. There is no established adverse effect of this intravenous iron on the foetus. The following table will help you understand the side effects that might happen as well as their frequencies:

**Side Effects**	**How often is it likely to occur?**	**How severe is itlikely to be?**	**How long might it last?**
Stomach aches, nausea, constipation,or diarrhoea (fromoral iron)	Perhaps up to 1 in 4 people	Mild	A few days of the duration of the treatment; but it is usually tolerable
Infection (e.g., diarrhoea, malaria)	As often as in peoplenot receiving iron, or perhaps slightly more commonly	About as severe as inwomen not receivingiron, or perhaps slightly more severe.	A few days.
Darkening of stools (from oral iron)	Common	Not harmful at all.	It lasts for the duration of the treatment.
Aches and pains in the joints, headaches (from intravenous iron)	Perhaps 1 in 20 to 1 in 8 people	Mild aches which will go away by themselves	2-3 days
Low blood phosphate levels (from intravenous iron)	Maybe 1 in 10 people	Mild – you will not notice this. It is just a change that occurs in your blood and is not usually harmful. We will test for this condition.	4-6 weeks

What will this study involve for my baby?

If you decide to participate in this study, we will perform all the usual clinical checks on yourbaby which will include assessing the baby's weight, length, head circumference and examining the baby for any congenital anomalies. We will also collect a small sample of yourbaby's first stool. In addition, we will check your baby' shearing response to sounds using a machine that measures auditory brain response. This will be done by placing small soft electrodes that look like headsets into your baby's ears. This assessment does not require any participation by the child. It is best

Screening ID									

Study ID									

done when the child is sleeping in their mother's arms. A small soft sound will be played, and the baby's response will be recorded using a computer. The sound played will be appropriate for the baby's age and will not be harmful. This will notpose any risk to the child and takes less than 10 minutes to perform.

After 1 month, we will ask you and the baby to return for a check-up, at which point we will weigh and measure the baby again. We will also take a heel-prick or a venous blood test for anaemia and iron deficiency in your baby. On this same visit, we will also collect a stool sample from your child for analysis. In addition, on the 1-month visit, we will also check your baby'shearing using auditory brain response. We will ask you to come back at 3, 6, 9 and 12 monthsafter birth, when we will measure and weigh your baby and collect blood samples.

At 3 and 12 months after birth, we will take x-ray images of your baby's knee and wrist to assess your child for the presence of radiological Rickets. The use of x-rays is a safe and non-invasive way to assess bones and your child will only need to be still for a short time.

Auditory brain response will also be rechecked on the 3 and 6 months visits. On the 3, 6 and 12 months visits, we will collect stool samples from your child. In addition to the procedures stated above,on the 6 and 12 months visit, will assess your child cognitive development and behavioural response. This involves measuring your child's reaction to sounds, pictures, videos and gamesusing the EEG method and Bayley tests. The EEG method consists in placing a cap on your child's head and playing them a sound or a picture or a video, and then recording how quicklyyour child's brain responds to the sound, image or video. It will only take a few minutes but provides us with robust information on whether the medicines we gave you in pregnancy have influenced your child's brain development. The test requires the fitting of the cap and connection of electrodes. Your child will be fitted with a cap (resembling a baby's hat) that measures the brain's response to image and sounds. During testing, the child will be sitting on your lap in a room with a screen and speakers. Your child does not have to do anything but the cap will measure your child's brain response to the images and sounds played. The technique measures brain development by providing acomputer image of what is happening in the brain. You will always be present while we conduct these procedures. Pictures and sound played for your child are culturally sensitive and appropriatefor his/her age, and your child will enjoy looking at and listening to them. If your child begins to fuss during testing, we will stop the test and resume once the child is feeling better. The techniqueis completely non-invasive, so it does not require blood or any samples from inside the body. It will not hurt the child. It does not pose any risk to the child and take less than 20 minutes per child.What are the possible risks and disadvantages for my child wearing this cap? There is no risk for your child wearing this cap. The test involves the cap that needs to be worn on the head. The childmay experience some discomfort with the cap during testing, but this will not last long. Our experienced testers will ensure complete hygienic precautions and minimum distress.

Screening ID									

Study ID									

Additionally, your baby will undergo behavioural child development measurements on the 6 and 12-months visits using Bayley Scales of Infant and Toddler Development III and behavioural test of memory and attention. Bayley tests mainly assess fine and gross motor, cognitive, language and socio-emotional development by evaluating how the child interacts with the environment, i.e. toys, response to sounds and recognising places and persons. This is done by placing toys in front of your baby to check if they recognise and respond to the toy and playing different games with your child and recording how they respond.

These tests will not harm your child in any way. Furthermore, at the 3 and 12 months visits, your child will have a picture of their brain taken using low field magnetic resonance imaging (MRI) on the Hyperfine machine. This procedure takes only 20 minutes for a picture and usually happens when the child is sleeping. The procedure is safe and non-invasive. This will help us understand how your child's brain is developing.

What will happen to my test samples?

You will be asked to provide consent for the collection of blood and other biological samples during the research project. Collection of blood is a required part of involvement in this project. Samples will be analysed for haemoglobin and iron levels in the blood, so we can see if the interventions have worked. We will also store the samples and use them to understand other immune, metabolic and molecular factors that may influence maternal and child health, their response to infections, nutritional status and development. Samples will be stored in such a way that your identity is kept private by use of a code.

Some of the samples we are collecting may also be used for future testing of DNA. We will not be testing for any genetic diseases, and so the results will not have any implications for yours nor your child's nor the rest of your family's health. These tests will not diagnose a genetic disease in the future. Instead, we anticipate testing for genetic differences that may be linked with anaemia, nutrition, development, health and growth, or immune and infection status. Samples and results of genetic testing will not be released to anyone other than the researchers or their colleagues helping with analysis of the samples.

What are the benefits of participating in this study?

If you decide to participate in this study, you will receive all the medical attention usually provided to both mothers and babies, plus you will be encouraged to visit our study site if you get sick during this study. We understand that the excessive travel to the clinics may be a burden and so we will compensate with the equivalent of 10 USD (MK 7000.00) for each visit to cover for yourlunch and transport costs.

By participating in this study, you will be contributing to identifying a better and effective

Screening ID									

Study ID									

treatment for moderate and severe anaemia during pregnancy. This can have a significant impact on the way anaemia is managed in Malawi and in the future pregnancy outcomes for women and babies in this region, thus benefiting your community as a whole.

What are the risks of participating in this study?

If you decide to join the study, the risks are minor. If you are enrolled in the intravenous iron arm, a small bruise or mild pain on the arm where the injection is given, and blood is taken may develop. There is also a minimal chance of infection at the site where blood is drawn from. This is almost negligible, however, because we always use sterile materials and our study personnel are highly skilled and trained phlebotomists. We understand the study will require you to make more visits to the hospital than usual. This may be inconvenient, but we will reimburse your transport costs.

The risks for your child are also minimal. All procedures (with the exception of the sampling of blood) are non-invasive and will not harm your child. As will happen with you, a small bruise or mild pain on the site where blood is taken may develop.

Privacy and confidentiality concerns

If you consent to take part in the research study, we will keep your name and that of your childand all the information that we get from you as part of this study confidential to the extent thatis required by the law. Only members of the study staff and people from the safety committeeand government authorities can review the records with your name and that of your child on it.We will use the information you give to us only for research. The information that we collect may be shared with other people in other institutes and countries, but your name and that of your child will not appear on any reports.

Who has reviewed the research project?

The ethical aspects of this research project have been approved by the Malawian Ethics Authorities (the College of Medicine Ethics Committee (COMREC) and or the National Health Sciences Research Committee (NHSRC) at the Ministry of Health) and; Health and Research Ethics Committee (HREC) of the Walter and Eliza Hall Institute of Medical Research, Melbourne, Australia.

Important contacts

Who is organising and funding the research?

This research project is being conducted by Professor Kamija Phiri of the College of Medicineand Dr. Sant-Rayn Pasricha of Walter and Eliza Hall Institute of Medical Research. This project is funded by the Bill and Melinda Gates Foundation.

Screening ID									

Study ID									

Further information and who to contact

If you want any additional information concerning this project or if the participant has any medical problems which may be related to their involvement in the project (for example, any side effects), you can ask us today or during our regular visits or subsequent visits. If you haveother questions later, you can contact: Prof Kamija Phiri, Principal Investigator, College of Medicine, Blantyre, +265999957048 or Dr. Sant-Rayn Pasricha, Principal Investigator from Walter and Eliza Hall Institute of Medical Research Tel: +61393452618

For matters relating to research at the site at which the child is participating, the details of the local site complaints person are:

Clinical and Complaints contact person

Name	*Zinenani Truwah*
Position	*Study Coordinator, Training and Research Unit of Excellence*
Telephone	*+265999413775/+2650882091578*
Email	*zinenanitruwah@gmail.com*

<u>Declaration by participant/ Guardian</u>

- I have read the consent, or someone has read it to me in Chichewa.
- I understand the purposes, procedures and risks of the research described in the project.
- I give permission for my doctors and my child's doctors, as well as other health professionals, hospitals or laboratories outside this hospital to release information to the College of Medicine and the Walter and Eliza Hall Institute of Medical Research concerning mine and my child's health and treatment for the purposes of this project. I understand that such information will remain confidential
- I have had the opportunity to ask questions, and I am satisfied with the answers I have received.
- I freely agree to participate and to have my child participating in this research project as described and I understand that I am free to withdraw my participation and my child's participation at any time during the research project without affecting mine or my child's future health care.
- I understand that I will be given a signed copy of this document to keep

Screening ID									

Study ID									

Certificate of Consent:

A. For all participants >18years of age, able to read and write

I have been invited to participate in a study of a medicine used to treat anaemia during pregnancy.

I have read the above information, or it has been read to me. I have had the opportunity to ask questions, and any questions that I have asked have been answered to my satisfaction. I consent voluntarily to my participation in this study.

Print name of participant: ____

Signature of participant: ____

Date (dd/mm/yyyy): ____

B. For participants>18years of age, unable to read or write

Witness' signature

(A witness' signature and the thumb-print of the participant are required only if the participant is illiterate. In this case, a literate witness must sign. If possible, this person should be selected by the participant and should have no connection with the study team.)

I have witnessed the accurate reading of the consent form to the potential participant, who has had the opportunity to ask questions. I confirm that the participant has given consent freely.

Print name of witness:
____

Signature of witness: ____

Date (dd/mmm/yyyy): ____

Right Thumbprint of participant:

Screening ID									

Study ID									

C. For participants<18years of age

1. Legal guardian signature

My daughter/ward has been invited to participate in a study of a medicine used to treat anaemia during pregnancy.

I have read the above information, or it has been read to me. I have had the opportunity to ask questions, and any questions that I have asked have been answered to my satisfaction. I consent voluntarily for my daughter or ward to participate in this study.

Print name of legal guardian: ____

Signature of legal guardian: ____

Date (dd/mm/yyyy): ____

Thumbprint of legal guardian (if unable to write):

If legal guardian is unable to write

I have witnessed the accurate reading of the consent form to:

____
*(Legal guardian's name should be written by witness **NOT** study staff)*

I confirm that she was given an opportunity to ask questions and she has given consent

freely. Name of witness

____

Signature of witness:

Screening ID									

Study ID									

Date (dd/mmm/yyyy): ____

Thumbprint of legal guardian (if unable to write):

1. Participant signature

I have been invited to participate in a study of a medicine used to treat anaemia during pregnancy.

I have read the above information, or it has been read to me. I have had the opportunity to ask questions, and any questions that I have asked have been answered to my satisfaction. I consent voluntarily to participate in this study. I understand that I have I can choose not to participate in this study even if my legal guardian consents for my participation.

Print name of participant: ____

Signature of participant: ____

Date (dd/mmm/yyyy): ____

Thumbprint of participant (if unable to write)

Screening ID									

Study ID									

D. Participants who do not wish to provided consent for extended use of samples.

Signature or Right thumb print

____

dd/mmm/yyyy

E. Investigator's signature:

I have accurately read or witnessed the accurate reading of the consent form to the potential participant, who has had the opportunity to ask questions. I confirm that the participant has given consent freely.

Print name of investigator/delegatee: ____

Signature of investigator/delegatee: ____

Date (dd/mm/yyyy): ____

NB: Give a copy of this informed consent form has been provided to participant

Screening ID									

Study ID									

REVAMP-TT STUDY
PARTICIPANT INFORMATION SHEET AND INFORMED CONSENT FORM (ICF)
ENGLISH VERSION

COVER SHEET

**Participant Name:**	
**Participants age at time of informed consent:**	
**Name of husband or guardian present during the consenting process (optional):**	
**Date of informed consent discussion:**	
**Date informed consent obtained:**	
**Time (24 hrs clock format):**	
**Name of study staff/ person completing informed consent discussion (and this coversheet):**	
**Is the potential volunteer literate?:**	☐ Yes
	☐ No (If no, an impartial witness must be present during the entire informed consent discussion. Refer to informed consent SOP for specific instructions.
**Was a copy of ICF given to the participant?:**	☐ Yes
	☐ No, refused to accept

Screening ID									

Study ID									

**Title**	Randomized controlled trial of the effect of intravenous iron on anaemia in Malawian pregnant women in the third trimester (REVAMP-TT)
**Project Sponsor**	Training and Research Unit of Excellence (TRUE),College of Medicine, University of Malawi
**Co - Principal Investigators**	1. Professor Kamija Phiri College of Medicine, BlantyreMalawi Phone: +265999957048<br>2. Dr. Sant-Rayn Pasricha Walter and Eliza Hall Institute of MedicalResearch, Melbourne, Australia Phone: +61393452618
**NHSRC Contacts**	If you want to know more about your rights as a research participant you can contact the NHSRC Secretariat, at the ministry of health on the following details, Phone: +2651 726422/418. Email: mohdoccenter@gmail.com.
**Locations**	Health centres in Zomba District, Malawi

Introduction

This is an invitation for you to take part in this research project evaluating methods for preventing anaemia in pregnant women. This *Participant Information Sheet/Consent Form* tells you about the research project. It explains the tests and treatments involved. Knowing what is involved will help you decide if you want to take part in the research. If you choose totake part in the research project, you will be asked to sign the consent section at the end. By signing this consent form, you are telling us that you:

1. Understand what you have read and/or heard
2. Consent to taking part in the research project
3. Consent for the tests and treatments that are described
4. Consent to the use of your personal and health information as described. You will be given a copy of this Participant Information and Consent Form to keep.

You will be free to withdraw from participating in this study at any time during the study without

Screening ID									

Study ID									

giving a reason. If you do not want to take part now, or in the future, and if you withdraw at any point from the study, this will not affect the standard of care you will receiveat the hospital.

Purpose of this study

Pregnant women living in Malawi have a high chance of developing anaemia during pregnancy. Antenatal anaemia may contribute to risks for both mother and child. The standardapproach for treating anaemia is oral iron. An intravenous-iron drugs, Ferric Carboxymaltose is now available in Western countries. This drug provides the opportunity to give high doses of iron in a single 15-minute infusion. This drug is currently costly; however, we believe it works better than oral iron for improving anaemia when we are trying to rescue anaemia late in pregnancy. The aim of this study is to determine the effectiveness and safety of intravenousiron administration during the third trimester of pregnancy – given as Ferric Carboxymaltose (FCM) – when compared with oral iron in improving maternal (especially anaemia) and infant(growth, birth weight and development up to 12 months postpartum) outcomes.

Study procedure

We will be asking pregnant women, such as yourself, in the third trimester of their pregnanciesand with signs of having anaemia to participate in this study. They will be randomly allocatedto one of two study arms: (a) intravenous iron FCM; (b) oral iron. All the women enrolled in this study will receive routine care, which may include malaria prevention drugs, if scheduled. Women in the intravenous iron intervention group will receive intravenous FCM 1000mg for body weight ≥ 50kg, or 20mg/kg for bodyweight <50kg, once during the third trimester once during the third trimester. The control group will receive oral iron 200mg Ferrous Sulphate (approx. 65 mg elemental iron) twice daily for the duration of pregnancy. All participants willreceive Sulfadoxine-Pyrimethamine (SP) as IPTp according to national guidelines if the threedoses of recommended IPTp have not been already achieved.

If you choose to participate in this study, we will ask you some questions about yourpsychological wellbeing; we will perform medical examinations; and we will collect a small amount of blood (2 teaspoons/visit) and other biological samples. We will ask you to return forvisits at 6 scheduled times: at 36 weeks' gestation, and 1 month, 3 months, 6 months, 9 months,and 12 months post-delivery. At the 36-week gestation visit, we will perform check-ups on you, ask some questions on your health and psychological wellbeing, and collect some blood.At this same visit, we will do a vaginal swab to test for the types of bacteria that may be presentin your reproductive tract. This may cause you some discomfort. There may be a need for you and your sexual partner(s) to be treatedif any inadvertent clinical findings are detected that could have health implications for you andyour partner(s) (i.e., STDs). Lastly, on this visit, we will ask to collect a sample of your stool. After your delivery, a member of our team will come to collect blood samples from the cord and placenta, as well as collect

Screening ID									
Study ID									

a small piece of the placenta for further tests that are meant to provide us with information with regards to the health and well-being of your baby. We will also record Apgar scores, and the baby will have a full physical examination, including measurement of birth weight, length, head circumference, auditory brainstem responses, and details of any congenital malformations. We will also take a small sample of your baby's firststool for analysis. We will ask you to come back at 1 month, 3 months, 6 months, 9 months, and 12 months after the birth, when we will check to see how you are feeling, collect blood samples, and measure and weigh your baby. In addition, during the 1-month, 6-month and 12-month post-delivery visits, we will take a small amount of expressed breast milk (2ml) for iron indices testing and other general well-being and nutritional parameters. We will also collect stool samples from both you and your child for analysis on these visits.

You are also asked to come to the study clinic anytime you are sick, for a check-up and blood test.

Allocating you to a treatment

It is essential that you understand that you will be assigned to one of the treatment arms totally by chance. Before this assignment, neither you nor the study staff will know to which group you will be assigned. You have equal chances of being assigned to either of the treatments described above. Once assigned, you will receive detailed information about the treatment you will receive.

If you are assigned to the intravenous iron intervention arm, you will receive the drug through a small drip inserted into your arm. If you receive oral iron, you will be given a course of tablets from your health worker, plus information on how to take the drug.

What happens after receiving treatment?

After we give you the treatment on that first visit, we will ask you to come to the study clinic at every scheduled study visit for study-specific procedures. Visits are scheduled for 36-weeks gestation, 1-month, 3-month, 6-month, 9-months, and 12-month post-delivery. In addition, we would encourage you to come to the study clinic whenever you are sick. You are also encouraged to go for delivery at the facility that has been allocated to you. You must never share the medicine with your fellow pregnant women or anyone.

Are there any side effects of the treatment?

All medicines have side effects. There is a rare chance that you may experience mild dizziness, joint aches, and headache, which usually subsidise without medications. Oral Iron may cause constipation, diarrhoea, and stomach aches, and is likely to turn your faeces black – but this is not dangerous. Chances are also very rare that one might experience potentially life-threatening allergic reactions immediately after given intravenous iron which might include symptoms like

Screening ID									

Study ID									

skin rash, nausea, low blood pressure and difficulty breathing. However, our clinical team is well trained to handle those conditions if happens. There is no established adverse effect of this intravenous iron on the foetus. The following table will help you understand the side effects that might happen as well as their frequencies:

**Side Effects**	**How often is it likely to occur?**	**How severe is itlikely to be?**	**How long might it last?**
Stomach aches, nausea, constipation,or diarrhoea (fromoral iron)	Perhaps up to 1 in 4 people	Mild	A few days of the duration of the treatment; but it is usually tolerable
Infection (e.g., diarrhoea, malaria)	As often as in peoplenot receiving iron, or perhaps slightly more commonly	About as severe as inwomen not receivingiron, or perhaps slightly more severe.	A few days.
Darkening of stools (from oral iron)	Common	Not harmful at all.	It lasts for the duration of the treatment.
Aches and pains in the joints, headaches (from intravenous iron)	Perhaps 1 in 20 to 1 in 8 people	Mild aches which will go away by themselves	2-3 days
Low blood phosphate levels (from intravenous iron)	Maybe 1 in 10 people	Mild – you will not notice this. It is just a change that occurs in your blood and is not usually harmful. We will test for this condition.	4-6 weeks

What will this study involve for my baby?

If you decide to participate in this study, we will perform all the usual clinical checks on yourbaby which will include assessing the baby's weight, length, head circumference and examining the baby for any congenital anomalies. We will also collect a small sample of yourbaby's first stool. In addition, we will check your baby' shearing response to sounds using a machine that measures auditory brain response. This will be done by placing small soft electrodes that look like headsets into your baby's ears. This assessment does not require any participation by the child. It is best

Screening ID									

Study ID									

done when the child is sleeping in their mother's arms. A small soft sound will be played, and the baby's response will be recorded using a computer. The sound played will be appropriate for the baby's age and will not be harmful. This will notpose any risk to the child and takes less than 10 minutes to perform.

After 1 month, we will ask you and the baby to return for a check-up, at which point we will weigh and measure the baby again. We will also take a heel-prick or a venous blood test for anaemia and iron deficiency in your baby. On this same visit, we will also collect a stool sample from your child for analysis. In addition, on the 1-month visit, we will also check your baby'shearing using auditory brain response. We will ask you to come back at 3, 6, 9 and 12 monthsafter birth, when we will measure and weigh your baby and collect blood samples.

At 3 and 12 months after birth, we will take x-ray images of your baby's knee and wrist to assess your child for the presence of radiological Rickets. The use of x-rays is a safe and non-invasive way to assess bones and your child will only need to be still for a short time.

Auditory brain response will also be rechecked on the 3 and 6 months visits. On the 3, 6 and 12 months visits, we will collect stool samples from your child. In addition to the procedures stated above,on the 6 and 12 months visit, will assess your child cognitive development and behavioural response. This involves measuring your child's reaction to sounds, pictures, videos and gamesusing the EEG method and Bayley tests. The EEG method consists in placing a cap on your child's head and playing them a sound or a picture or a video, and then recording how quicklyyour child's brain responds to the sound, image or video. It will only take a few minutes but provides us with robust information on whether the medicines we gave you in pregnancy have influenced your child's brain development. The test requires the fitting of the cap and connection of electrodes. Your child will be fitted with a cap (resembling a baby's hat) that measures the brain's response to image and sounds. During testing, the child will be sitting on your lap in a room with a screen and speakers. Your child does not have to do anything but the cap will measure your child's brain response to the images and sounds played. The technique measures brain development by providing acomputer image of what is happening in the brain. You will always be present while we conduct these procedures. Pictures and sound played for your child are culturally sensitive and appropriatefor his/her age, and your child will enjoy looking at and listening to them. If your child begins to fuss during testing, we will stop the test and resume once the child is feeling better. The techniqueis completely non-invasive, so it does not require blood or any samples from inside the body. It will not hurt the child. It does not pose any risk to the child and take less than 20 minutes per child.What are the possible risks and disadvantages for my child wearing this cap? There is no risk for your child wearing this cap. The test involves the cap that needs to be worn on the head. The childmay experience some discomfort with the cap during testing, but this will not last long. Our experienced testers will ensure complete hygienic precautions and minimum distress.

Screening ID									

Study ID									

Additionally, your baby will undergo behavioural child development measurements on the 6 and 12-months visits using Bayley Scales of Infant and Toddler Development III and behavioural test of memory and attention. Bayley tests mainly assess fine and gross motor, cognitive, language and socio-emotional development by evaluating how the child interacts with the environment, i.e. toys, response to sounds and recognising places and persons. This is done by placing toys in front of your baby to check if they recognise and respond to the toy and playing different games with your child and recording how they respond.

These tests will not harm your child in any way. Furthermore, at the 3 and 12 months visits, your child will have a picture of their brain taken using low field magnetic resonance imaging (MRI) on the Hyperfine machine. This procedure takes only 20 minutes for a picture and usually happens when the child is sleeping. The procedure is safe and non-invasive. This will help us understand how your child's brain is developing.

What will happen to my test samples?

You will be asked to provide consent for the collection of blood and other biological samples during the research project. Collection of blood is a required part of involvement in this project. Samples will be analysed for haemoglobin and iron levels in the blood, so we can see if the interventions have worked. We will also store the samples and use them to understand other immune, metabolic and molecular factors that may influence maternal and child health, their response to infections, nutritional status and development. Samples will be stored in such a way that your identity is kept private by use of a code.

Some of the samples we are collecting may also be used for future testing of DNA. We will not be testing for any genetic diseases, and so the results will not have any implications for yours nor your child's nor the rest of your family's health. These tests will not diagnose a genetic disease in the future. Instead, we anticipate testing for genetic differences that may be linked with anaemia, nutrition, development, health and growth, or immune and infection status. Samples and results of genetic testing will not be released to anyone other than the researchers or their colleagues helping with analysis of the samples.

What are the benefits of participating in this study?

If you decide to participate in this study, you will receive all the medical attention usually provided to both mothers and babies, plus you will be encouraged to visit our study site if you get sick during this study. We understand that the excessive travel to the clinics may be a burden and so we will compensate with the equivalent of 10 USD (MK 7000.00) for each visit to cover for yourlunch and transport costs.

By participating in this study, you will be contributing to identifying a better and effective

Screening ID									

Study ID									

treatment for moderate and severe anaemia during pregnancy. This can have a significant impact on the way anaemia is managed in Malawi and in the future pregnancy outcomes for women and babies in this region, thus benefiting your community as a whole.

What are the risks of participating in this study?

If you decide to join the study, the risks are minor. If you are enrolled in the intravenous iron arm, a small bruise or mild pain on the arm where the injection is given, and blood is taken may develop. There is also a minimal chance of infection at the site where blood is drawn from. This is almost negligible, however, because we always use sterile materials and our study personnel are highly skilled and trained phlebotomists. We understand the study will require you to make more visits to the hospital than usual. This may be inconvenient, but we will reimburse your transport costs.

The risks for your child are also minimal. All procedures (with the exception of the sampling of blood) are non-invasive and will not harm your child. As will happen with you, a small bruise or mild pain on the site where blood is taken may develop.

Privacy and confidentiality concerns

If you consent to take part in the research study, we will keep your name and that of your childand all the information that we get from you as part of this study confidential to the extent thatis required by the law. Only members of the study staff and people from the safety committeeand government authorities can review the records with your name and that of your child on it.We will use the information you give to us only for research. The information that we collect may be shared with other people in other institutes and countries, but your name and that of your child will not appear on any reports.

Who has reviewed the research project?

The ethical aspects of this research project have been approved by the Malawian Ethics Authorities (the College of Medicine Ethics Committee (COMREC) and or the National Health Sciences Research Committee (NHSRC) at the Ministry of Health) and; Health and Research Ethics Committee (HREC) of the Walter and Eliza Hall Institute of Medical Research, Melbourne, Australia.

Important contacts

Who is organising and funding the research?

This research project is being conducted by Professor Kamija Phiri of the College of Medicineand Dr. Sant-Rayn Pasricha of Walter and Eliza Hall Institute of Medical Research. This project is funded by the Bill and Melinda Gates Foundation.

Screening ID									

Study ID									

Further information and who to contact

If you want any additional information concerning this project or if the participant has any medical problems which may be related to their involvement in the project (for example, any side effects), you can ask us today or during our regular visits or subsequent visits. If you haveother questions later, you can contact: Prof Kamija Phiri, Principal Investigator, College of Medicine, Blantyre, +265999957048 or Dr. Sant-Rayn Pasricha, Principal Investigator from Walter and Eliza Hall Institute of Medical Research Tel: +61393452618

For matters relating to research at the site at which the child is participating, the details of the local site complaints person are:

Clinical and Complaints contact person

Name	*Zinenani Truwah*
Position	*Study Coordinator, Training and Research Unit of Excellence*
Telephone	*+265999413775/+2650882091578*
Email	*zinenanitruwah@gmail.com*

<u>Declaration by participant/ Guardian</u>

- I have read the consent, or someone has read it to me in Chichewa.
- I understand the purposes, procedures and risks of the research described in the project.
- I give permission for my doctors and my child's doctors, as well as other health professionals, hospitals or laboratories outside this hospital to release information to the College of Medicine and the Walter and Eliza Hall Institute of Medical Research concerning mine and my child's health and treatment for the purposes of this project. I understand that such information will remain confidential
- I have had the opportunity to ask questions, and I am satisfied with the answers I have received.
- I freely agree to participate and to have my child participating in this research project as described and I understand that I am free to withdraw my participation and my child's participation at any time during the research project without affecting mine or my child's future health care.
- I understand that I will be given a signed copy of this document to keep

Screening ID									

Study ID									

Certificate of Consent:

A. For all participants >18years of age, able to read and write

I have been invited to participate in a study of a medicine used to treat anaemia during pregnancy.

I have read the above information, or it has been read to me. I have had the opportunity to ask questions, and any questions that I have asked have been answered to my satisfaction. I consent voluntarily to my participation in this study.

Print name of participant: ____

Signature of participant: ____

Date (dd/mm/yyyy): ____

B. For participants>18years of age, unable to read or write

Witness' signature

(A witness' signature and the thumb-print of the participant are required only if the participant is illiterate. In this case, a literate witness must sign. If possible, this person should be selected by the participant and should have no connection with the study team.)

I have witnessed the accurate reading of the consent form to the potential participant, who has had the opportunity to ask questions. I confirm that the participant has given consent freely.

Print name of witness:
____

Signature of witness: ____

Date (dd/mmm/yyyy): ____

Right Thumbprint of participant:

Screening ID									

Study ID									

C. For participants<18years of age

1. Legal guardian signature

My daughter/ward has been invited to participate in a study of a medicine used to treat anaemia during pregnancy.

I have read the above information, or it has been read to me. I have had the opportunity to ask questions, and any questions that I have asked have been answered to my satisfaction. I consent voluntarily for my daughter or ward to participate in this study.

Print name of legal guardian: ____

Signature of legal guardian: ____

Date (dd/mm/yyyy): ____

Thumbprint of legal guardian (if unable to write):

If legal guardian is unable to write

I have witnessed the accurate reading of the consent form to:

____

*(Legal guardian's name should be written by witness **NOT** study staff)*

I confirm that she was given an opportunity to ask questions and she has given consent freely. Name of witness

____

Signature of witness:

____

Screening ID									

Study ID									

Date (dd/mmm/yyyy): ______________________________

Thumbprint of legal guardian (if unable to write):

1. Participant signature

I have been invited to participate in a study of a medicine used to treat anaemia during pregnancy.

I have read the above information, or it has been read to me. I have had the opportunity to ask questions, and any questions that I have asked have been answered to my satisfaction. I consent voluntarily to participate in this study. I understand that I have I can choose not to participate in this study even if my legal guardian consents for my participation.

Print name of participant: ______________________________

Signature of participant: ______________________________

Date (dd/mmm/yyyy): ______________________________

Thumbprint of participant (if unable to write)

Screening ID									

Study ID									

D. Participants who do not wish to provided consent for extended use of samples.

Signature or Right thumb print

____

dd/mmm/yyyy

E. Investigator's signature:

I have accurately read or witnessed the accurate reading of the consent form to the potential participant, who has had the opportunity to ask questions. I confirm that the participant has given consent freely.

Print name of investigator/delegatee: ______________________________

Signature of investigator/delegatee: ______________________________

Date (dd/mm/yyyy): ______________________________

NB: Give a copy of this informed consent form has been provided to participant

Screening ID:									

Study ID:									

REVAMP-TT STUDY
PARTICIPANT INFORMATION SHEET AND INFORMED CONSENT FORM (ICF)
CHICHEWA VERSION

COVER SHEET

**Participant Name:**	
**Participants age at time of informed consent:**	
**Name of husband or guardian present during the consenting process (optional):**	
**Date of informed consent discussion:**	
**Date informed consent obtained:**	
**Time (24 hrs clock format):**	
**Name of study staff/ person completing informed consent discussion (and this coversheet):**	
**Is the potential volunteer literate?:**	Yes
	No (If no, an impartial witness must be present during the entire informed consent discussion. Refer to informed consent SOP for specific instructions.
**Was a copy of ICF given to the participant?:**	Yes
	No, refused to accept

Screening ID:									

Study ID:									

Mutu wa Kafukufuku	Kafukufuku owunikira ubwino olandilira mu msempha chithandizo cha mankhwala olimbana ndi nthenda ya kuchepa kwa magazi kwa amayi aku Malawi omwe ali ndipakati popotsera miyezi isanu ndi umodzi kapena kuti REVAMP-TT mu chingerezi
Amene akuthandiza ndi ndalama pa kafukufukuameneyu	Training and Research Unit of Excellence (TRUE),College of Medicine, University of Malawi
Akuluakulu a zofufuzafufuzamu kafukufuku ameneyu	1. Professor Kamija<br>Phiri College of<br>Medicine, Blantyre<br>Malawi<br>Phone: +265999957048<br><br>2. Dr Sant-Rayn Pasricha<br>Walter and Eliza Hall Institute of Medical Research, Melbourne Australia<br>Phone: +61393452618
Ma nambala a *lamya* kapenakuti foni a komiti yoyang`anira za malamulo a kafukufuku (*NHSRC*)	Ngati mukufuna kudziwa zambiri za ufulu wanu potenga nawo mbali mu kafukufukuyu mukhonza kuyimbira foni kwa mlembi wamuofesi ya NHSRC ku unduna wa za umoyo pa nambala iyi: +2651726422/418 kapena kutumiza kalata kudzela pa makina a intaneti ku: mohdoccenter@gmail.com
Malo ochitikira kafukufukuyu	Zipatala zazing`ono mu boma la Zomba kuno ku Malawi

Choyambilira

Mwayitanidwa kuti mutenge nawo mbali mu kafukufuku owunikira njira zopewela matenda a kuchepa kwa magazi kwa amayi a pakati. Kalatayi ikufotokozera ndondomeko ya kafukufukuyu. Ikufotokozanso za kuyeza mthupi komanso chithandizo cha mankhwala chimene chidzakhalepo. Kudziwa za mmene kafukufukuyu akhalire kudzakuthandizani kupanga chisankho ngati mukufuna kutenga nawo mbali mu kafukufukuyu. Ngati musankha kutenga nawo mbali mu kafukufukuyu mudzafunsidwa kuti musayine kalata yovomeleza kumapeto kwake. Pamene mwasayina kalata yovomelezayi ndiye kuti mukutiuza kuti:

1. Mwamvetsetsa zimene mwawerenga kapena kufotokozeledwa
2. Mwavomeleza kutenga nawo mbali mu kafukufukuyu
3. Mwavomeleza zoyezedwa komanso chithandizo cha mankhwala chimene

Screening ID:										

Study ID:										

chafotokozedwa

4. Mwavomeleza kuti tidzagwiritsa ntchito uthenga okhuza inu komanso umoyo wanu monga mmene zafotokozedwela

Mudzapatsidwa ina mwa kalata iyi yomwe iri ndi uthenga wa kafukufukuyu komanso kuvomeleza kwanu potenga nawo mbali mukafukufukuyu kuti musunge.
Mudzakhala ndi ufulu osiya kutenga nawo mbali mu kafukufukuyu nthawi ina ili yonse ngakhale osapereka zifukwa zimene mwasiyila. Ngati panopa simukufuna kutenga nawo mbali, kapena mtsogolomu, ndipo ngati mudzasiyila pa njira kutenga nawo mbali pa kafukufuku ameneyu, izi sidzidzakhuza kalandiridwe ka chithandizo chimene mudzalandira pamane mwapita ku chipatala inu ndi mwana wanu.

Cholinga cha Kafukufuku ameneyu
Amayi a pakati a ku Malawi ali ndi kuthekela kwakukulu kuti angathe kudwala matenda ochepa magazi pamene ali oyembekezela. Nthenda ya kuchepa kwa magazi kwa mayi oyembekezela ingathe kupeleka chiopsezo kwa mayiyo pamodzi ndi mwana amene akuyembekezelayo. Njira imene ikugwiritsidwa ntchito pothana ndi vuto la kuchepa kwa magazi mthupi ndi kumwa mankhwala amene ali ngati mchere othandidzila kuonjezela magazi mthupi dzina lake ayironi. Ku mayiko a ku *Ulaya* kapena kuti ku mayiko a azungu tsopano kukupezeka mankhwala operekedwa podzera musempha/diripi amene amathandidza kuthetsa vutoli otchedwa ferric carboxymaltose. Mankhwalawa amapeleka mwayi kuti munthu alandire mchere wambiri wa ayironi mu mphindi khumi ndi zisanu zokha pamene akulandilira mankhwalawa mu njira ya jakisoni kudzela mu msempha. Mankhwalawa ndi okwera mtengo kwambiri; komabe, tikukhulupilira kuti amagwira ntchito bwino pochepetsa bvuto la kuchepa kwa magazi mthupi kuposa makhwala a aironi akumwa makamaka panthawi imene mzimai woyembekedzera wasala pang'ono kuchila/kubeleka.

Cholinga cha kafukufukuyu ndichofuna kuwunikira ubwino komanso njira yotetezeka yopelekera mankhwala amenewa kudzela mu msempha a ferric carboxymaltose kwazimayi yemwe ali ndi pakati/mimba yomwe yapitiri miyezi isanu ndi umodzi poyerekedza ndi makhwala akumwa a aironi. Mukuchita izi ndiye kuti tidzakhala tikusiyanitsa kagwiridwe ka ntchito poyerekeza ndi mankhwala wokumwawa a *aironi* makamaka polimbana ndi mavuto obwera kamba ka kupelewera kwa magazi kwa amayi oyembekezera komanso zotsatira za umoyo wa mwana kuyambira tsiku limene anabadwa mpaka kufikira atakwanitsa miyezi khumi ndi iwiri, kapena kuti chaka

Mmene Kafukufukuyu adzayendere

Tidzakhala tikufunsa mafunso kwa amayi oyembekezela ngati inuyo amene muli ndipakati popotsera miyezi isanu ndi umodzi komanso omwe magazi awo ali ochepa kuti atenge nawo mbali mu kafukufuku ameneyu. Iwo adzayikidwa mwa kasakaniza/mwamwayi mugulu limodzi lamagulu awiri a kafukufukuyu. Gulu loyamba lidzidzalandira mankhwala a mchere owonjezela magazi otchedwa *Ferric Carboxymaltose* kapena kuti *FCM* kudzela mu msempha wa magazi ndipo gulu lachiwiri lidzidzalandila mankhwala a mchere owonjezela magazi otchedwa kuti *aironi* koma kuzela njira yakumwa. Amayi onse amene adzatenge nawo mbali mu kafukufukuyu adzidzalandila chithandizo mu nthawi imene idzakhazikitsidwe, kuphatikizaponso mankhwala oteteza ku matenda a malungo ngati mayi ali oyenela kuyikidwa pa ndandanda wa mankhwala oteteza ku malungo.

Screening ID:									

Study ID:									

Amayi amene azakhale mu gulu lolandira mankhwala a mchere owonjezela magazi mthupi kuzela mu njira ya jakisoni wa mmisempha adzalandila mankhwala otchedwa *Ferric Carboxymaltose* kapena kuti *FCM* ma miligalamu 1000 kwa mayi olemela thupi ma kiligalamu 50 kapena kuposela apo, kapena adzalandira mankhwala a mlingo okwana ma milugalamu 20 pa 1 kilogalamu ina iliyonse ngati kulemela kwa thupi lawo sikunafike ma kiligalamu 50 omwe adzapelekedwa kamodzi basi panthawi yomwe pathupi pawo pali popotsera miyezi isanu ndi umodzi. Gulu lachiwiri lidzalandila mankhwala a mchere owonjezela magazi mthupi otchedwa kuti *Ferrous Sulphate* (kapena kuti *ayironi)* omwe azidzamwa kawiri pa tsiku mpaka adzabeleke,ndipo mlingo wake udzakhala ma miligalamu 200 omwe uli pafupifupi ma miligalamu 65 a mchere wotchedwa *elemental iron*. Onse otenga nawo mbali mu kafukufukuyu adzalandila mankhwala owatetedza ku malungo otchedwa *Sulfadoxine-Pyrimethamine (SP)* malingana ndi ndondomeko ya dziko.

Ngati musankha kutenga nawo mbali mu kafukufukuyu, tidzakufunsani mafunso ena okhuza za umoyo wanu, tidzakuyezani mthupi, tidzidzatengako magazi anu pang`ono okwana masupuni aang`ono awiri paulendo uliwonse mwabwera kuno komanso tizatenga zina ndi zina zokayeza zamthupi mwanu. Tidzakufunsani kuti mubwele ku chipatala kasanu ndi kamodzi (ma ulendo sikisi). Ulendo oyamba mudzabwela pamene mimba yanu ili ndi ma sabata okwana 36 kapena kuti miyezi 9, ulendo wachiwiri pakatha mwezi umodzi kuyambira tsiku limene mwana wanu anabadwa, ulendo wachitatu patatha miyezi itatu, ulendo wachinayi patatha miyezi isanu ndi umodzi, ulendo wachisanu patatha miyezi isanu ndi inayi ndipo ulendo omaliza (wa nambala sikisi) patatha miyezi khumi ndi iwiri (kapena kuti chaka) chibadwire mwana wanu.

Pamene mudzabwera pa sabata ya nambala 36 kapena kuti mwezi wa nambala 9, tizakuyezani zinthu zosiyana siyana pa thupi lanu, tidzakufunsani mafunso okhuza umoyo wanu komanso thanzi la kaganizidwe kanu ndiponso tidzakutengani magazi. Pa ulendo omweu, tidzayezanso njira yanu ya chibelekero pogwiritsa ntchito ka chipangizo kakang`ono kamene kamakhala ndi thonje kutsogolo kwake kuti tione mitundu ya tizilombo toyambisa matenda timene tingapezeke mu njirayi. Dziwani kuti padzafunika kuti inu komanso ukondedwa anu mulandire chithandizo chamakhwala ngati pangapezeke kuti zosatira zakuyedza zasocheza kuti njira yachibelekero chanu muli tizilombo (zina mwa izo ndi monga matenda opatsirana pogonana). Chomalizira pa ulendo umenewu tidzakufunsani kuti titenge nawo pang`ono chimbudzi chanu. Ndipo mukadzabeleka, munthu mmodzi wa gulu lathu adzabwera kudzatengako pang`ono magazi kuchokera ku thumbo la pa mchombo la mwana komanso kuchokera ku mbali imodzi ya msengwa lachibelekero.

Kuphatikiza apo, adzatenganso ka chiduswa kakang`ono kwambiri ka mbali imodzi ya msengwa la chibelekero kamene ka matuluka limodzi pa nthawi yobeleka. Kupima zithu zimenezi kudzatipatsa ife uthenga odziwa umoyo komanso thanzi la mwana wanu. Kuonjezela apo tidzapimanso mmene mwana wanu akuonekera mmene magazi ake akuyendera, kutakataka, mmene akupumira komanso kumupima zina ndi zina monga kumukweza sikelo kuti tione mmene akulemelela pa nthawi imene wabadwa, kutalika kwake, kutambalala kwa mutu, mmene akumvera ndi makutu ake komanso kupima kapena kumuona ngati ali ndi chilema chilli chonse.

Screening ID:									

Study ID:									

Tidzatenganso chimbudzi cha mwana wanu kuti tikachipime. Ndipo tikatero tidzakufunsaninso kuti mudzabwere pakatha mwezi umodzi, kenako pakatha miyezi itatu, ulendo wina pakatha miyezi isanu ndi umodzi, pakatha miyezi isanu ndi inayi komanso ulendo omaliza pakatha miyezi isanu ndi iwiri (chaka). Mu maulendo amenewa tidzidzakupimani pofuna kuona mmene mukupezela mthupi, tidzidzatengakonso magazi pang`ono komanso tidzidzayeza ndikukweza mwana wanu sikelo. Kuonjezela apo, mu ulendo umene mudzabwera mu mwezi oyamba mwana atabadwa, komanso ulendo umene mudzabwera patatha miyezi isanu ndi umodzi kapena kuti 6 ndi ulendo umene mudzabwera patatha miyezi khumi ndi iwiri kapena chaka, tidzidzatengako mkaka wa mmaere anu kuti tikaupime pofuna kuona mmene michere yowonjezera magazi mthupi ilili komanso thanzi lanu ndi la mwana wanu. Mumaulendo onsewa tidzidzatengakonso chimbudzi chanu komanso cha mwana wanu kuti zikapimidwe. Mukufunsidwanso kuti mudzibwera ku chipatala chino chimene kukuchitikira kafukufukuyu nthawi ina iliyonse pamene mwadwala kudzapimidwa mthupi komanso kuyezetsa magazi.

Kayikidwe ka gulu la mankhwala

Ndi zofunikira kuti mudziwe komanso kumvetsetsa kuti mudzayikidwa mu gulu limodzi mwa la mankhwala mwa mwayi chabe. Dziwani kuti palibe angakhale mmodzi mwa ife amene akudziwa zagulu lomwe mungaikidwe. Muli ndimwayi wofanana kugwera gulu limodzi mwamagulu awiriwa. Muzapatsidwa uthenga onse ofunikira kutsatira molingana ndi gulu limene mwapatsidwa/mwagwera.

Ngati zapezeka kuti muli mu gulu la anthu olandira chithandizo cha mankhwala a mchere owonjezela magazi mthupi a *FCM*, ndiye kuti mudzalandira mankhwalawa kudzela mu diripi yaying`ono imene adzalowesera mankhwalawa kudzela mu jakisoni mu msempha wa pa nkono wanu. Ndipo ngati zapezeka kuti muli mu gulu la anthu olandila chithandizo cha mankhwala a mchere owonjezela magazi lake ***iron***, ndiye kuti mudzapatsidwa mpukutu wa mankhwala kuchokera kwa adotolo anu komanso adzakupatsani ndondomeko ya kamwedwe kake ka mankhwalawo.

Chidzachitike ndi chiyani pa mapeto a chithandizo cha mankhwala amene mudzalandira

Pamene takupatsani chithandizo cha mankhwala ulendo oyamba, tidzakufunsani kuti mudzibwera ku chipatala chino chimene kafukufukuyu akuchitikira mu nthawi kapena miyezi imene tidzakupatsani kuti mudzidzalandira zithandizo zosiyanasiyana. Mabweredwe a ku chipatala chino kumene kafukufukuyu akuchitikira agawidwa motere; Ulendo oyamba mudzafika pamene mimba yanu ili ndi miyezi 36 kapena kuti yakwanisa miyezi isanu ndi inayi (9). Ulendo wachiwiri mudzabwera patatha mwezi umodzi kuyambira tsiku limene munabeleka, ulendo wachitatu mudzabwera patatha miyezi itatu, ulendo wachinayi mudzabwera patatha miyezi isanu ndi umodzi (6), ulendo wachisanu mudzabwera patatha miyezi isanu ndi inayi ndipo ulendo omaliza mudzabwera patatha miyezi khumi ndi iwiri (kapena kuti chaka).

Screening ID:									

Study ID:									

Kuonjezera apo, tidzakulimbikitsaninso kuti mudzibwera ku chipatala chino kumene kafukufukuyu akuchitikira nthawi zonse pamene mwadwala. Kuonjezela apo tikukulimbikitsaninso kuti mudzapite kukachilira ku chipatala chomwe mwauzidwa. Dziwani kuti simukuyenera kugawana mankhwalawa ndi mayi mzanu wapakati kapena munthu wina aliyense.

Kodi pali zovuta zina zimene zingathe kubwera chifukwa cha mankhwalawa?

Mankhwala ena ali wonse ali ndi zovuta zina zimene amabweretsa pamene munthu akulandira. Pali mpata ochepa kwambiri kuti mutha kumva zinthu zina mthupi monga, chizungulire pang`ono, kuphwanya malo olumikizana mafupa, komanso kumva kuwawa kwa mutu. Koma izi nthawi zambiri zimasiya zokha popanda kumwera mankhwala. Pamene mankhwala amchere owonjezera magazi mthupi kudzera pokumwa otchedwa *iron* nthawi zina amatha kupangitsa munthu kuzimbidwa, kutsekula mmimba komanso kuwawa kwa mmimba. Amathanso kupangitsa chimbuzi kusandulika chakuda. Koma izi sizoopsa ayi pa moyo wa munthu ngati zitachitika. Pali mwayi wochepa kwambiri kwa munthu yemwe walandira makhwala owonjezera magazi kudzera musepha kuti thupi lake silingayanjane nawo ndipo akhoza kutulaka tizironda mthupi, kuchita nseru, kusika kwakathamangidwe kamagazi mthupi komanso kubanika kupuma. Koma tikufuna kukusimikizirani kuti madokotala athu adaphuzitsidwa bwino mmene angakuthandizireni izi zitachita. Makhwalawa alibe chiopsezo china chirichonse pa mwana yemwe mukuyembekezerayi. Zigawo zili mmusimu zikuthandizani kuti mumvetsetse zovuta zina zimene mankhwala angathe kubweretsa komanso kuti kodi zimenezi zimabwera mowilikiza motani:

Zovuta Zina	Kodi Zingachitike mochuluka bwanji?	Kukula kwa vutoli kungakhale kotani?	Kodi bvutoli limatenga nthawi yayitali bwanji?
Kupweteka kwa mmimba, kumva nseru, kudzimbidwa kapena kutsekula mmimba (pamene tamwa mankhwala otchedwa *Iron*)	Mwina zingachitike kwa munthu mmodzi mwa anthu anayi aliwonse	Pang`ono basi	Kwa masiku ochepa pamene mukumwa mankhwalawa, koma nthawi zambiri munthu amantha kukhala bwinobwino ngakhale osamwa mankhwala

Screening ID:										

Study ID:										

Matenda ena obwera chifukwa cha tidzirombo toyambitsa matenda (monga kutsekula mmimba kapena malungo)	Zimachitika mofanana ndi anthu amene sakumwa mankhwala owonjezela magazi, kapena nthawi zina mochulukilako pang`ono	Mofanana ndi anthu amene sakumwa mankhwala owonjezela magazi kapena nthawi zina mochulukilapo pang'ono	Kwa masiku ochepa
Chimbudzikukhala chakuda (pamene umwa mankhwala	Zimachitika kwa anthu ambiri	Sizoopsa nkomwe	Limakhala pa nthawi imene mankhwalawa akumwedwa basi
Kuphwanya kwa thupi makamaka mu malo olumikizana mafupa (pamene munthu akulandila chithandizo kuzela mu msepha)	Mwina munthu mmodzi mwa anthu makumi awiri (20) kapena munthu mmodzi mwa anthu anthu asanu ndi atatu (8)	Kuphwanya thupi pang`ono mwa patali patali koma nthawi zambiri kumasiya okha	Masiku awiri kapena atatu
Kuchepa kwa mlingo wa mchere umene umapezekanso mmagazi dzina lake fosifeti (kwa amene akulandilira chithandizo cha mankhwala kudzela mu msempha)	Mwina kwa munthu mmodzi mwa anthu khumi (aliwonse)	Pang`onochabe – mwinanso simudzamva kalikonse mthupi mwanu. Ndikusintha chabe kumene kumachitika mu magazi anu ndipo sizoopsa ayi. Komabe tidzakuyezaninso zimenezi.	Ma sabata anayi mwina mpaka masaba asanu ndi limodzi

Kodi kafukufukuyu adzakhuzana bwanji ndi mwana wanga?

Ngati musankha kutenga nawo mbali mu kafukufuku ameneyu ndiye kuti tidzachita monga mmene zimakhalira ku sikelo ya ana, monga kuyeza mwana wanu mmene akulemelera, usinkhu, kutambalala kwa mutu wake komanso kumupima ngati ali ndi zilema kapena matendaotengela pobadwa. Komanso tidzatengako pang`ono chimbuzi choyamba cha mwana wanu. Kuonjezera

Screening ID:									

Study ID:									

apo, tidzayeza ngati mwana wanu ali ndi vuto lina lina lili lonse la kamvedwe pogwiritsa ntchito makina a kompyuta otchedwa *Auditory Brain Response* mu chingerezi. Izi zidzatheka pomuveka mwana wanu makina opangidwa mowoneka ngati timawaya tating′ono komanso tofewa tomwe timagwiritsidwa ntchito kumvetsera nyimbo m`makutu. Kuyeza izi sikumafuna kuti mwana wanu achitepo chili chonse, makamaka zimakhala bwino kuchita izi pamene mwanayo ali kugona mmanja mwa amayi ake. Ka nyimbo ka pansipansi kadzaseweredwa, ndiye mmene mwanayo akuchitira pamene nyimboyi ikuseweredwa zidzaoneka komanso kusungidwa mu chipangizo cha magesi dzina lake *Computer*. Kukula kwa phokoso la nyimboyo kudzakhala kolingana ndi msinkhu wa mwanayo ndipo sizidzakhala zoopsa kwa mwanayo. Ndipo izi sizidzakhala zoopsa kwa mwanayo, komanso chifukwa zimangotenga mphindi zosakwana khumi (10) basi kuti zitheke. Mwana wanu akakwanitsa miyezi itatu (3) komanso khumi ndi masiku awiri (12) tidzamujambula ndi makina a x-ray pachigong′otho ndi mmawondo ndicholinga chofuna kuwunika ngati ali ndi matewe. Izi zizatenga nthawi yochepa chabe basi ndipo sidzimafuna kubaya mwana wanu.

Pakazatha mwezi umodzi tidzakufunsani kuti mudzabwerenso inu ndi mwana wanu kuti tidzakupimeni. Pa nthawi imeneyi, tidzakwezanso mwana wanu sikelo pofuna kuona kulemela kwa mwana wanu komanso kumupima zina ndi zina. Tidzatenganso magazi pang′ono kudzela pa phazi lake kapena kudzela pa nkono wa mwana wanu kuti tiyedze zizindikilo za kusowa kwa magazi kapenanso ngati akupelewera mchere otchedwa *Iron* umene umathandiza kuwonjezela magazi mthupi. Pa tsiku lomweli tidzatengakonso pang`ono chimbuzi cha mwana wanu kuti tikachipime. Kuonjezela apo, pa ulendo umene mudzabwera patangotha mwezi umodzi pamene mwana anabadwa tidzayezanso mamvedwe a mwana wanu pogwiritsa ntchito chipangizo chimene mu chingerezi chimatchedwa kuti *Auditory Brain Response*.

Tidzakufunsaninso kuti mudzabwerenso inu ndi mwana mu mwezi wa chitatu, kenako pakatha miyezi isanu ndi umodzi, pakathanso miyezi isanu ndi inayi ndipo ulendo omaliza mudzabwere pakatha miyezi khumi ndi iwiri (12) kapena kuti chaka. Mu ma ulendo onsewa tidzidzakweza mwana wanu sikelo pofuna kuona kulemela kwake, kumupima mthupi komanso kutengako pang`ono kagazi. Tidzamupimanso kamvedwe kake mmakutu mu ulendo umene mudzabwere patatha miyezi itatu komanso ulendo umene mudzabwera patatha miyezi isanu ndi umodzi. Mu ma ulendo amene mudzabwere patatha miyezi itatu, miyezi isanu ndi umodzi komanso ulendo umene mudzabwere patatha miyezi khumi ndi iwiri (12) kapena kuti chaka, tidzidzatengako pang`ono chimbuzi cha mwana wanu. Kuonjezela pa zopima zimene tafotokozazi, mu ulendo umene mudzabwera patatha miyezi isanu andi umodzi komanso ulendo umene mudzabwere patatha miyezi khumi ndi iwiri (12) kapena kuti chaka, tidzayeza mmene ubongo wa mwana wanu ukugwilira ntchito. Makamaka pofuna kuona monga mmene ubongo wa mwana wanu akuchitila pamene mwana wanu wamva phokoso kapena nyimbo imene, pamene wapatsidwa kapena kuona zithunzi, kuonela kanema kapena masewero osiyanasiyana. Zinthu izi zidzayezedwa pogwiritsa ntchito makina otchedwa *EEG* komanso njira ina yotchedwa kuti *Bayley tests*. Izi zimatheka pomuveka mwana wanu ku mutu makina amenewa omwe anapangidwa mooneka ngati chipewa.

Screening ID:									

Study ID:									

Kenako timamusewelera nyimbo, kumuonetsa chithunzi kapena kanema. Zikatero makina athu amajambula kapena kuonesa changu chimene ubongo wa mwana wanu ukuchitila, kapena kugwirira ntchito pamene wamva nyimbo kapena phokoso, waona kapena kupatsidwa chithunzi komanso pamene waonera kanema. Izi zidzatenga ka mphindi kochepa kokha koma zidzatipatsa ife uthenga okwanira pofuna kuona kuti kodi mankhwala amene tinakupatsani pamene munali oyembekezela athandizapo motani kumbali ya kaganizidwe komanso mmene ubongo wa mwana wanu ukugwirira ntchito. Monga mmene tafotokozera, poyeza izi mwana wanu tidzamuveka makina owoneka ngati chipewa koma chokhala ndi mawaya opangidwa ndi pulasitiki panja pake omwe adzathandizira kutionetsammene ubongo wake ukugwilira ntchito pamene wapatsidwa kapena kuwona chithunzi kapenanso pamene wamva nyimbo kapena phokoso. Pa nthawi imene tizidzayeza izi, mwanayu adzakhala pa miyendo panu mu chipinda mmene tidzayikamo kanema komanso wayilesi yomwe idzidayimba nyimbo. Pa nthawiyi, mwana wanu sadzafunika kuchita chilichonse koma makina amene adzavale kumutuwo ndi amene adzidzayeza kapena kuonesa mmene ubongo wake ukuchitila kapena kugwirira ntchito pamene waona zithunzi kapena wamva nyimbo kapena phokoso. Njira iyi imayeza mmene ubongo wa mwana wanu ukugwirira ntchito powonesa pa makina a kompyuta zinthu zimene zikuchitika mu ubongo wa mwanayo. Inu monga mayi a mwamayu mudzakhala muli mu chipinda chomwecho pamene ntchito yopimayi ikuchitika. Zinthuzi kapena nyimbo zimene zidzagwiritsidwe ntchito popima mwana wanu zaunikilidwa kuti ndi zolingana ndi msinkhu wa mwanayo monga mwa chikhalidwe chathu ku Malawi, ndipo mwana wanu adzasangalala kwambiri powona zithunzizo komanso kumvela nyimbo zimene zidzaseweredwa! Ngati mwana wanu adzayamba kuvuta kapena kulira pa nthawi yomwe tikumuyeza, tidzaimitsa kaye zoyezayezazi ndipo tidzapitiliza pamene mwana wanu watonthola ndi kukhazikika. Mwana wanu sadzamva kupweteka kwina kulikonse panthawi imeneyi ndipo sipadzafunikira kumubaya kapena kutenga magazi. Ndipo chipewa chomwe mwana wanu azavale sichidzamupweteka mu njira ina ili yonse. Monga mmene tafotokozela, palibe chiopsezo china chili chonse pamene mwana wanu wavala chipewa chimenechi komanso ntchitoyi imangotenga mphindi zosakwana makumi awiri basi pa mwana aliyense.

Pali chiopsezo chanji pamene mwana wanga wavala chipewa chimenechi?

Monga mmene tafotokozela, palibe chiopsezo china chili chonse pamene mwana wanu wavala chipewa chimenechi, kungoti kuyezaku kumafunikira kuti mwana wanu avale makina owoneka ngati chipewawa kumutu kwake basi. Mwina nkutheka kuti mwana wanu adzakhala omangika pang`ono pa nthawi imene tikugwira ntchito yoyezayi, komabe madotolo athu amene adzayeza mwana wanu ndi akadaulo omwe akhala akugwira ntchitoyi kwa zaka zambiri ndipo adzaonetsetsa kuti chipewachi chidzakhale chaukhondo pa nthawi yomwe mwana wanu akuvala komanso kuonetsetsa kuti mwana wanu adzakhale okondwa pa nthawi yonse yomwe akuyezedwa. Kuonjezela apo tidzayezanso kapena kumuona mwana wanu mmene akukulira komanso kuchitila mmakhalidwe ake pogwiritsa ntchito njira yotchedwa kuti *Bayley tests.*

Izi zidzachitika pamene mudzabwera ku chipatala kuno mu mwezi wa nambala sikisi komanso mwezi wa khumi ndi iwiri kapena kuti chaka. Njirayi makamaka imayeza kagwiridwe ntchito ka

Screening ID:									
Study ID:									

ubongo wa mwana pa zinthu zina monga, kukumbukira zinthu, chidwi, kayankhula, kukwiya kapena kusangalala komanso kuchitachita ndi zinthu zosiyana siyana monga zidole, pamene wamva nyimbo kapena phokoso komanso kudzindikira malo komanso anthu. Kuyeza izi kumachitika poyika zidole ku tsogolo kwa mwana wanu kuti tione ngati iye angathe kuzizindikira komanso kutha kusewera nazo kapena kuchita nazo china chilichonse. Kuonjezera apo tidzaseweranso masewero osiyanasiyana ndi mwana wanu uku tikuona mmene akuchitira. Zonsezi sizoopsa kwa mwana wanu ayi. Kuwonjezera apo, mwana wanu akatha miyezi itatu (3) komanso isanu ndi umodzi (6) tidzamujambura ubongo wake ndimakina a low field magnetic resonance (MRI; awonetseni makinawo). Izi zidzachitika mwaphindi khumi ziwiri (20 minitsi) ndipo tidzachita izi pamene mwana wanu akugona. Palibe bvuto lina lirilonse limene mwana wanu angakumane nalo chifukwa chakujambulako komanso mwana wanu sazabayidwa. Kujambulaku kudzatithandiza kuti tidziwe mmene ubongo wamwana wanu ukukulira.

Kodi chidzachitike ndi chiyani kwa madzi kapena magazi a mthupi mwanga amene adzatengedwa kukapimidwawo?

Mudzafunsidwa kuti mupereke chilolezo kuti tingathe kutenga nawo magazi anu, madzi kapena zina mu thupi lanu kuti zikapimidwe mu nthawi ya kafukufukuyu. Kutengedwa kwa magazi anu ndi mbali imodzi yofunika komanso yosonyeza kuti inu muli kutenga nawo mbali mu kafukufuku ameneyu. Magazi amene azatengedwa adzapimidwa kufiira kwake komanso kupezeka kwa nchere otchedwa iron kuti tione ngati chithandizo cha mankhwala chimene munalandira chagwira ntchito. Komanso tizawasungabe ndikuwagwiritsa ntchito popima za mmene chitetezo chanu cha mthupi chilili komanso kagwiridwe ntchito ka thupi lanu pofuna kuona kuti zimenezi zingakudze bwanji umoyo wa inu mayi oyembekela komanso umoyo wa mwanayo maka maka polimbana ndi matenda, thanzi komanso kakulidwe. Magazi kapena zina zimene tidzakutengani mthupi zidzasungidwa mowonetsetsa kuti chinsinsi chanu chasungidwa. Izi zidzatheka pogwitsa ntchito nambala ya chinsinsi.

Magazi kapena zina zimene tidzakhala tikutenga mthupi mwanu tidzagwiritsa tchito pofuna kupima chibadwa mtsogolo muno. Koma sitidzayeza matenda ochokera ku makolo ayi, choncho zotsatirazi sidzidzakhuza umoyo wa inu, kapena mwana wanu angakhale umoyo wa anthu a ku banja kwanu. Komanso kuyezaku sikudzalosela kapena kupeza matenda a ku makolo amene agathe kudzabwera mtsogolo. Komano tikuyembekezera kudzayeza kusiyana kwa chibadwa kumene kumakhuzana ndi kupelewera kwa magazi mthupi, thanzi la munthu, kakulidwe ka nsinkhu kapena ziwalo kapena mmene thupi lake limalimbanirana, kapena kugwidwa ndi matenda. Magazi kapena zina zimene tidzakhala tikutenga mthupi lanu ndi kuziyeza komanso zotsatira zake sidzidzapatsidwa kapena kuonetsedwa kwa wina aliyense kupatula madotolo kapena akaswiri okhawo ndi anzawo ena amene akuthandizila nawo kafukufukuyu.

Kodi phindu lotenga nawo mbali mu kafukufuku ameneyu ndi lotani?

Ngati musankha kutenga nawo mbali mu kafukufuku ameneyu ndiye kuti mudzalandila chithandizo cha mankhwala chimene nthawi zonse chimaperekedwa kwa amayi ndi ana. Komanso kuonjezela apo tidzakulimbikitsani kuti mudzibwera ku chipatala chathu chino

Screening ID:									

Study ID:									

kumene kafukufukuyu achichitikira kudzatenga chithandizo cha mankhwala ngati mukudwala makamaka pa nthawi imene kafukufukuyu ali kuchitika. Tikumvetsetsa kuti kubwera ku chipatala kuno ma ulendo ochulukirapo kungathe kukhala kotopetsa. Kotero tidzakupasani ndalama yokwanira MK 7,000.00. (~US$10). Cholinga chake ndi chakuti mubwezeletse pa ndalama yanu ya mayendedwe kapena kuti thiransipoti komanso kugulira chakudya cha masana.

Pamene mukutenga nawo mbali mu kafukufuku ameneyu dziwani kuti mukuthandizira kuti akaswiri a za chipatala apeze njira yabwino komanso yodalilika yothana kapena kuchiza vuto la kuchepa kwa magazi nthupi kumene thawi zina kumathanso kukhala vuto lalikulu makamaka kwa amayi mu nthawi imene ali ndi pakati. Kafukufuku ameneyu atha kubweretsa ubwino waukulu pa nkhondo yolimbana ndi kuchepa kwa magazi mthupi kuno ku Malawi komanso kuthandidza kukonza bwino zotsatira za amayi a pakati ndi ana mtsogolo muno makamaka dela lino la chigawo cha kummwera kwa dziko la Malawi komanso koposaposa dela la kuno kwanu.

Kodi pali chiopsezo cha mtundu wanji pamene ndikutenga nawo mbali mu kafukufuku ameneyu?

Ngati mupanga chisankho chotenga nawo mbali mu kafukufuku ameneyu, chiopsezo kapena zovuta zina ndi zochepa kwambiri. Mwachisanzo, ngati mwayikidwa mu gulu lolandila chithandizo cha makhwala kudzela mu msempha wa pa nkono, ndiye kuti mutha kukhala ndi ka chilonda kakang`ono kapena kumva kuwawa pang`ono pa malo pamene anakubayani jakisoni nkuyika diripi ya mankhwala, kapena pamene anabaya potengako magazi pang`ono. Palinso kuthekera kochepa zedi kuti pa malo pamene anakubayani pangathe kusanduka chilonda. Izi monga mmene tafotokozela zilibe chiopsezo pa thupi lanu chifukwa nthawi zonse timagwiritsa ntchito zipangizo zotetezedwa bwino ndi mankhwala opha ma jelemusi, komanso anthu amene akugwira ntchito mu kafukufuku ameneyu ndi akaswiri komanso ophunzitsidwa bwino ntchito yawo. Tikudziwa kuti kafukufukuyu adzidzafuna kuti mudzabwere kuno ma ulendo ochulukirapo kuposa nthawi zonse. Tikudziwa kuti izi zidzatha kukupangisani kuti nthawi zina mudzikhala otangwanika, komabe tidzidzakubwezelani ndalama ya mayendedwe kapena kuti thiransipoti yanu.

Komanso dziwani kuti chiopsezo kapena kuti zovuta zina pa mwana wanu mu kafukufuku ameneyu ndi chochepa kwambiri. Njira zonse za kupima inu kapena mwana wanu sizidzafuna kuti kubowola kapena kubaya khungu la inu kapena mwana wanu pokha pokha pamene tikufuna kutengako magazi kuti tikawapime. Koma dziwani kuti izi sizidzavulaza mwana wanu. Monga mmene zidzakhalira kwa inu mayi, nayenso mwana adzatha kukhala ndi kachilonda kakang`ono kapena kumva kuwawa pang`ono pa malo pamene tidzabaya ndi kutengako magazi.

Zokhuza Chinsinsi mu Kafukufukuyu

Ngati muvomeleza kutenga nawo mbali mu kafukufukuyu, ife tidzasunga mwachinsinsi dzina lanu komanso la mwana wanu kuphatikizapo uthenga umene tidzapeza kuchokera kwa inu ndi mwana wanu pamene kafukufukuyu ali kuchitika. Malamulo a dziko amafotokoza za kusungira anthu chinsinsi. Komanso chilango chake ngati munthu waphwanya lamuloli. Choncho ifenso

Screening ID:									

Study ID:									

tidzagwira ntchito potsatira malamulowo.

Anthu amene angaone kapena kugwiritsa ntchito uthenga wa inu kapena mwana wanu komanso dzina lanu kapena la mwana wanu ndi okhawo amene avomelezedwa kugwira ntchito mu kafukufuku ameneyu, komanso anthu amene akugwira ntchito yoteteza anzawo mu kafukufuku ameneyu komanso kuphatikizapo akulu akulu a boma ogwira ntchito za chipatala basi. Tidzagwiritsa ntchito uthenga umene mudzatipatsa mu kafukufukuyu basi. Uthenga kapena zotsatira zimene tidzapeza mu kafukufuku ameneyu tingathe kugawana ndi anzathu a ma ofesi ena kapenanso mayika ena koma dziwani kuti sipadzakhala dzina lanu pa mwamba pa uthenga umenewu kapena zolembedwa ku mapeto a kafukufuku ameneyu.

Kodi ndi ndani waunikira kapena kulondoloza mmene kafukufukuyu adzayendere

Malamulo kapena zoyenera kutsatira pamene kafukufukuyu akuchitika zavomerezedwa ndi akomiti kapena gulu la anthu lowona za malamulo a kafukufuku lotchedwa *College of Medicine Ethics Committee kapena* kuti *COMREC* ndi a kafukufuku lotchedwa *National Health Sciences Research Committee* kapena kuti *NHSRC*. Komiti imeneyi imapezeka ku unduna wa za umoyo. Komanso iwowa akuthandidzana ndi komiti yowona za umoyo komanso za malamulo a kafukufuku yotchedwa *Health and Research Ethics Committee* kapena kuti *HREC* imene ili nthambi ya bungwe lotchedwa kuti *Walter and Eliza Hall Institute of Medical Research* imene imapezeka mu mzinda wotchedwa *Melbourne* mu dziko la *Australia*.

Anthu ofunika kuwadziwa mu kafukufuku ameneyu

Kodi ndi ndani amene akupereka thandizo la ndalama za kafukufuku ameneyu?

Amene akupangitsa kapena kuyendesa kafukufuku ameneyu ndi mphunzitsi wankulu dzina lawo ndi a Kamija Phiri amene amagwira ntchito ku kusukulu ya ukachenjede imene maphunzitsa madotolo komanso anamwino kapena kuti ma nesi. Dzina la sukulu imeneyi ndi *College of Medicin*e. Komanso iwowa akugwira limodzi ntchitoyi ndi anzawo a Sant-Rayn Pasricha amene ndi dotolo wankulu ku sukulu yochita kafukufuku wa za umoyo yotchedwa *Walter and Eliza Hall Institute of Medical Research*. Koma chithandizo cha ndalama choyendetsera kafukufukuyu chikuchokera ku bungwe lotchedwa kuti *Bill and Melinda Gates Foundation* limene limapezeka mu dziko la America.

Uthenga owonjezera komanso amene mungathe kulumikizana nawo

Ngati mukufuna uthenga wina uliwonse owonjezera okhuza kafukufukuyu, kapena mwina ngati mmodzi wa otenga nawo mbali mu kafukufuku ameneyu wadwala mwina chifukwa chakuti mankhwala amene wamwa abweletsa zovuta zina mthupi monga mmene tinafotokozera poyamba paja, mukhoknza kutifunsa lero pompano, kapena kudzatifunsa nthawi imene tabwera kuno mtsogolomu. Koma ngati mudzakhala ndi mafunso ena mtsogolomu mukhonza kulumikizana ndi mphunzitsi wankulu a Kamija Phiri amenenso ndi wankulu wa kufukufuku ameneyu. Iwowa amapezeka ku sukulu yotchedwa *College of Medicine* ku Blantyre. Mutha kuwayimbira foni pa nambala iyi; +265999957048. Kapena muthanso kulumikizana ndi anzawo

Screening ID:									

Study ID:									

amene ndi dotolo wankulu, dzina lawo ndi a Sant-Rayn Pasricha poyimba foni pa nambala iyi; +61393452618

Iwowanso ndi nkulu wa kafukufuku koma amachokera ku sukulu yochita za kafukufuku wa za umoyo yotchedwa *Walter and Eliza Hall Institute of Medical Research* imene imapezeka mu dziko la Australia.

Kumbali yokhuza chidandaulo chilichonse makamaka chokhuzana ndi kafukufukuyu mutha kukaonana ndi amene ayikidwa kuti aziona za chidandaulo ku chipatala chimene mwana wanu akutenga nawo gawo mu kafukufukuyu. Ndipo pezani mmunsimu udindo, dzina komanso nambala ya foni ya amene mungathe kulumikizana nawo kuti mupereke chidandaulo chanu.

Kulumikizana ndi munthu wa za umoyo ndi munthu owona za chidandaulo kumalo amene kafukufuku akuchitikira

Dzina	*Zinenani Truwah*
Udindo	*Olondoloza ntchito za kafukufuku, Training and Research Unit ofExcellence*
Nambala ya lamya kapena foni	*+265999413775 kapena +2650882091578*
Njira kapena adiresi yotumizira kalata pa compyuta kapena kuti intaneti	*zinenanitruwah@gmail.com*

Ngati muli ndi madandaulo a zina ndi zina zokhuza kafukufukuyu, monga mmene ntchitoyi ikuyendera, kapena mafunso aliwonse ochokera kwa inu monga mmodzi wa anthu otenga nawo mbali mutha kulumikizana ndi anthu a ku ofesi kudzela mu ma nambala a foni ali mmunsiwa.

Dzina la owunikira za umoyo komanso za malamulo a kafukufuku	**National Health Sciences Research Committee**
Oyimirira komiti yowunikira za umoyokomanso za malamulo a kafukufuku	*Dr. Collins Mitambo*
Nambala ya lamya kapenakuti foni	*+265999397913*
Njira kapena adiresi yotumizira kalata pa compyuta kapena kuti intaneti	*cmitambo@gmail.com*

Screening ID:									

Study ID:									

Kuvomera kwa otenga nawo mbali mu kafukufukuyu kapena kuvomera kudzela kwa owayimilira

- Ndikuvomera kuti ndawerenga kalata ya chilolezo kapena kuti munthu wina wandiwerengera mu Chichewa
- Ndikuvomera kuti ndamvetsetsa zolinga za kafukufukuy, njira zimene zidzatsatidwa, komanso zovuta zina zimene zingathe kubwera pamene munthu watenga nawo mbali, komanso monga mmene zafotokozeledwa ku kalatayi yokhuza kafukufukuyu
- Ndikuvomera kuti ndipereka chilolezo kwa ma dotolo anga komanso ma dotolo a mwana wanga kuphatikiza akaswiri ena a za umoyo, zipatala kapena nyumba zopimila matenda kupatula chipatala chino kuti angathe kutulutsa kapena kupeleka zotsatira za kafukufukuyu, monga umoyo wa mwana wanga komanso zokhuza chithandizo chamankhwala kwa akaswiri ochokera ku sukulu ya ukachenjede yotchedwa College of Medicine komanso akaswiri a za chipatala ochokera ku sukulu yotchedwa *Walter and Eliza Hall Institute of Medical Research*. Izi zidzachitika makamaka pofuna kukwanilitsa cholinga cha kafukufukuyu. Ndipo ndikudziwa kuti uthenga wa ine komanso okhuza umoyo wa mwana wanga udzakhala wa chinsinsi ndipo kuti sudzapatsidwa kwa munthu wamba
- Ndikuvomera kuti ndinali ndi mpata ofunsa mafunso ndipo ndakhutira ndi mayankho amene ndalandira Mwakufuna kwanga ndikuvomereza kutenga nawo mbali komanso kuti mwana wanga athanso kutenga nawo mbali mu kafukufuku ameneyu monga mmene zafotokozeledwa, komanso ndikumvetsa kuti ndine omasuka kusiya kutenga nawo mbali chimodzimodzinso mwana wanga nthawi ina iliyonse kafukufukuyu ali mkati popanda kuopa kwina kuli konse pa zokhuza chithandizo cha ku chipatala kwa ine mayi ngakhalenso mwana wanga mtsogolomu.
- Ndikumvetsa kuti ndidzapatsidwa kalata iyi ya chilolezo komanso yokhuza za kafukufukuyu kuti ndisunge ine mwini.

Chiphaso chachilolezo:

A. Wotenga nao mbali amene ali opitilira zaka 18 zakubadwa amene amadziwa kuwerenga:

Lembani dzina lawotenga nawo mbali:

____

__________________________ _______________

Saini ya otenga nao mbali Tsiku/mwezi/chaka

Screening ID:									

Study ID:									

B. Wotenga nawo mbali amene ali opitilira zaka 18 zakubadwa amene samadziwa kuwerenga:

Saini yamboni

(Saini ya mboni ndi chidindo chawotenga nawo mbali ndizofunika pokhapokha ngati otenga nawo mbali ndiwosadziwa kulemba ndi kuwerenga. Izi zikakhala chomwechi, mboni yodziwa kulemba ndikuwerenga ikuyenera kusaina. Ngati nkotheka, munthu ameneyu akuyenera kusankhidwa ndiwotenga nawo mbali ndipo sakuyenera kukhala pa ubale ndiwogwira ntchito mukafukufuku).

Ndawonelera kuwerenga bwino kwa kalata yachilolezo kwa amene atenge nawo mbali, amene anapatsidwa nthawi yofunsa mafunso. Ndikutsimikiza kuti otenga nawo mbali wavomera mwa kufuna kwake.

Lembani dzina la mboni ______________________________

Saini la mboni: ______________________________

Tsiku (Tsiku/mwezi/chaka) ______________________________

Ndi chidindo cha chala chachikulu cha ku manja cha wotenga nawo mbali

C. Wotenga nawo mbali wosapitilira zaka 18 zakubadwa

1. Saini ya kholo kapena womuyang'anira wovomerezeka:

Mwana wanga wayitanidwa kuti atenge nawo mbali mukafukufuku wamankhwala ogwiritsidwa ntchito poteteza kuchepa kwa magazi panthawi yomwe ali woyembekezera.

Ndawerenga/ndawerengeredwa uthenga omwe uli pamwambapa. Ndinapatsidwa nthawi yofunsa mafunso, ndipo mafunso ena aliwonse omwe ndinafunsa ndayankhidwa ndipo ndakhutitsidwa. Ndikuvomera mwakufuna kwanga kuti mwana wanga atenge nawo mbali mukafukufukuyu.

Dzina la kholo ______________________________

Saini la kholo: ______________________________

Screening ID:										
Study ID:										

Tsiku (Tsiku/mwezi/chaka) ____

Ngati kholo kapena womuyang'anira wovomerezeka sadziwa kulemba
Ndawonelera kuwerenga bwino kwa kalata yachilolezo kwa:

____

(Lembani dzina la kholo kapena womuyang'anira wovomerezeka. Dzinali lilembedwe ndi mboni osati ogwira mukafufukufuku)

Ndikutsimikiza kuti anapatsidwa nthawi yofunsa mafunso ndipo wavomera kutenga nawo mbali mwa kufuna kwake.

Dzina la mboni ____

Saini la mboni: ____

Tsiku (Tsiku/mwezi/chaka) ____

Chidindo cha chala chachikulu cha ku manja (makolo amene sadziwa kulemba)

2. Saini ya wotenga nawo mbali:

Ndayitanidwa kuti nditenge nawo mbali mukafukufuku wamankhwala ogwiritsidwa ntchito poteteza kuchepa kwa magazi panthawi yomwe ndili woyembekezera.

Ndawerenga/ndawerengeredwa uthenga omwe uli pamwambapa, Ndinapatsidwa nthawi yofunsa mafunso, ndipo mafunso ena aliwonse omwe ndinafunsa ndayankhidwa ndipo ndakhutitsidwa. Ndikuvomera mwakufuna kwanga kuti kutenga nawo mbali mukafukufukuyu. Ndikuzindikira kuti nditha kusankha kusatenga nawo mbali mukafukufuku ngakhale kholo litavomera kutero.

Lembani dzina la wotenga nao mbali:

____

Screening ID:									

Study ID:									

Saini la wotenga nao mbali:

Tsiku (Tsiku/mwezi/chaka)

Chidindo cha chala chachikulu cha ku manja (amene sadziwa kulemba)

D. Wotenga nawo mbali yemwe sadapereke chilolezo chogwiritsa uthenga komanso zomwe adayezedwa ngati magazi mukafukufuku watsogolo.

Saini kapena chidindo

Tsiku/mwezi/chaka

Saini ya *PI* kapena omuyimirira   Tsiku/mwezi/haka

NB: Chikalata china chachilolezo chaperekedwa kwa wotenga nawo mbali mukafukufukuyu

Screening ID:									

Study ID:									

REVAMP-TT STUDY
PARTICIPANT INFORMATION SHEET AND INFORMED CONSENT FORM (ICF) CHICHEWA VERSION

COVER SHEET

**Participant Name:**	
**Participants age at time of informed consent:**	
**Name of husband or guardian present during the consenting process (optional):**	
**Date of informed consent discussion:**	
**Date informed consent obtained:**	
**Time (24 hrs clock format):**	
**Name of study staff/ person completing informed consent discussion (and this coversheet):**	
**Is the potential volunteer literate?:**	☐ Yes
	☐ No (If no, an impartial witness must be present during the entire informed consent discussion. Refer to informed consent SOP for specific instructions.
**Was a copy of ICF given to the participant?:**	☐ Yes
	☐ No, refused to accept

Screening ID:									

Study ID:									

Mutu wa Kafukufuku	Kafukufuku owunikira ubwino olandilira mu msempha chithandizo cha mankhwala olimbana ndi nthenda ya kuchepa kwa magazi kwa amayi aku Malawi omwe ali ndipakati popotsera miyezi isanu ndi umodzi kapena kuti REVAMP-TT mu chingerezi
Amene akuthandiza ndi ndalama pa kafukufukuameneyu	Training and Research Unit of Excellence (TRUE),College of Medicine, University of Malawi
Akuluakulu a zofufuzafufuzamu kafukufuku ameneyu	1. Professor Kamija Phiri College of Medicine, Blantyre Malawi Phone: +265999957048<br><br>2. Dr Sant-Rayn Pasricha Walter and Eliza Hall Institute of Medical Research, Melbourne Australia Phone: +61393452618
Ma nambala a *lamya* kapenakuti foni a komiti yoyang`anira za malamulo a kafukufuku (*NHSRC*)	Ngati mukufuna kudziwa zambiri za ufulu wanu potenga nawo mbali mu kafukufukuyu mukhonza kuyimbira foni kwa mlembi wamuofesi ya NHSRC ku unduna wa za umoyo pa nambala iyi: +2651726422/418 kapena kutumiza kalata kudzela pa makina a intaneti ku: mohdoccenter@gmail.com
Malo ochitikira kafukufukuyu	Zipatala zazing`ono mu boma la Zomba kuno ku Malawi

Choyambilira

Mwayitanidwa kuti mutenge nawo mbali mu kafukufuku owunikira njira zopewela matenda a kuchepa kwa magazi kwa amayi a pakati. Kalatayi ikufotokozera ndondomeko ya kafukufukuyu. Ikufotokozanso za kuyeza mthupi komanso chithandizo cha mankhwala chimene chidzakhalepo. Kudziwa za mmene kafukufukuyu akhalire kudzakuthandizani kupanga chisankho ngati mukufuna kutenga nawo mbali mu kafukufukuyu. Ngati musankha kutenga nawo mbali mu kafukufukuyu mudzafunsidwa kuti musayine kalata yovomeleza kumapeto kwake. Pamene mwasayina kalata yovomelezayi ndiye kuti mukutiuza kuti:

1. Mwamvetsetsa zimene mwawerenga kapena kufotokozeledwa
2. Mwavomeleza kutenga nawo mbali mu kafukufukuyu
3. Mwavomeleza zoyezedwa komanso chithandizo cha mankhwala chimene

Screening ID:									

Study ID:									

chafotokozedwa

4. Mwavomeleza kuti tidzagwiritsa ntchito uthenga okhuza inu komanso umoyo wanu monga mmene zafotokozedwela

Mudzapatsidwa ina mwa kalata iyi yomwe iri ndi uthenga wa kafukufukuyu komanso kuvomeleza kwanu potenga nawo mbali mukafukufukuyu kuti musunge.
Mudzakhala ndi ufulu osiya kutenga nawo mbali mu kafukufukuyu nthawi ina ili yonse ngakhale osapereka zifukwa zimene mwasiyila. Ngati panopa simukufuna kutenga nawo mbali, kapena mtsogolomu, ndipo ngati mudzasiyila pa njira kutenga nawo mbali pa kafukufuku ameneyu, izi sidzidzakhuza kalandiridwe ka chithandizo chimene mudzalandira pamane mwapita ku chipatala inu ndi mwana wanu.

Cholinga cha Kafukufuku ameneyu

Amayi a pakati a ku Malawi ali ndi kuthekela kwakukulu kuti angathe kudwala matenda ochepa magazi pamene ali oyembekezela. Nthenda ya kuchepa kwa magazi kwa mayi oyembekezela ingathe kupeleka chiopsezo kwa mayiyo pamodzi ndi mwana amene akuyembekezelayo. Njira imene ikugwiritsidwa ntchito pothana ndi vuto la kuchepa kwa magazi mthupi ndi kumwa mankhwala amene ali ngati mchere othandidzila kuonjezela magazi mthupi dzina lake ayironi. Ku mayiko a ku *Ulaya* kapena kuti ku mayiko a azungu tsopano kukupezeka mankhwala operekedwa podzera musempha/diripi amene amathandidza kuthetsa vutoli otchedwa ferric carboxymaltose. Mankhwalawa amapeleka mwayi kuti munthu alandire mchere wambiri wa ayironi mu mphindi khumi ndi zisanu zokha pamene akulandilira mankhwalawa mu njira ya jakisoni kudzela mu msempha. Mankhwalawa ndi okwera mtengo kwambiri; komabe, tikukhulupilira kuti amagwira ntchito bwino pochepetsa bvuto la kuchepa kwa magazi mthupi kuposa makhwala a aironi akumwa makamaka panthawi imene mzimai woyembekedzera wasala pang'ono kuchila/kubeleka.

Cholinga cha kafukufukuyu ndichofuna kuwunikira ubwino komanso njira yotetezeka yopelekera mankhwala amenewa kudzela mu msempha a ferric carboxymaltose kwazimayi yemwe ali ndi pakati/mimba yomwe yapitiri miyezi isanu ndi umodzi poyerekedza ndi makhwala akumwa a aironi. Mukuchita izi ndiye kuti tidzakhala tikusiyanitsa kagwiridwe ka ntchito poyerekeza ndi mankhwala wokumwawa a *aironi* makamaka polimbana ndi mavuto obwera kamba ka kupelewera kwa magazi kwa amayi oyembekezera komanso zotsatira za umoyo wa mwana kuyambira tsiku limene anabadwa mpaka kufikira atakwanitsa miyezi khumi ndi iwiri, kapena kuti chaka

Mmene Kafukufukuyu adzayendere

Tidzakhala tikufunsa mafunso kwa amayi oyembekezela ngati inuyo amene muli ndipakati popotsera miyezi isanu ndi umodzi komanso omwe magazi awo ali ochepa kuti atenge nawo mbali mu kafukufuku ameneyu. Iwo adzayikidwa mwa kasakaniza/mwamwayi mugulu limodzi lamagulu awiri a kafukufukuyu. Gulu loyamba lidzidzalandira mankhwala a mchere owonjezela magazi otchedwa *Ferric Carboxymaltose* kapena kuti *FCM* kudzela mu msempha wa magazi ndipo gulu lachiwiri lidzidzalandila mankhwala a mchere owonjezela magazi otchedwa kuti *aironi* koma kuzela njira yakumwa. Amayi onse amene adzatenge nawo mbali mu kafukufukuyu adzidzalandila chithandizo mu nthawi imene idzakhazikitsidwe, kuphatikizaponso mankhwala oteteza ku matenda a malungo ngati mayi ali oyenela kuyikidwa pa ndandanda wa mankhwala oteteza ku malungo.

Screening ID:									

Study ID:									

Amayi amene azakhale mu gulu lolandira mankhwala a mchere owonjezela magazi mthupi kuzela mu njira ya jakisoni wa mmisempha adzalandila mankhwala otchedwa *Ferric Carboxymaltose* kapena kuti *FCM* ma miligalamu 1000 kwa mayi olemela thupi ma kiligalamu 50 kapena kuposela apo, kapena adzalandira mankhwala a mlingo okwana ma milugalamu 20 pa 1 kilogalamu ina iliyonse ngati kulemela kwa thupi lawo sikunafike ma kiligalamu 50 omwe adzapelekedwa kamodzi basi panthawi yomwe pathupi pawo pali popotsera miyezi isanu ndi umodzi. Gulu lachiwiri lidzalandila mankhwala a mchere owonjezela magazi mthupi otchedwa kuti *Ferrous Sulphate* (kapena kuti *ayironi)* omwe azidzamwa kawiri pa tsiku mpaka adzabeleke,ndipo mlingo wake udzakhala ma miligalamu 200 omwe uli pafupifupi ma miligalamu 65 a mchere wotchedwa *elemental iron*. Onse otenga nawo mbali mu kafukufukuyu adzalandila mankhwala owatetedza ku malungo otchedwa *Sulfadoxine-Pyrimethamine (SP)* malingana ndi ndondomeko ya dziko.

Ngati musankha kutenga nawo mbali mu kafukufukuyu, tidzakufunsani mafunso ena okhuza za umoyo wanu, tidzakuyezani mthupi, tidzidzatengako magazi anu pang`ono okwana masupuni aang`ono awiri paulendo uliwonse mwabwera kuno komanso tizatenga zina ndi zina zokayeza zamthupi mwanu. Tidzakufunsani kuti mubwele ku chipatala kasanu ndi kamodzi (ma ulendo sikisi). Ulendo oyamba mudzabwela pamene mimba yanu ili ndi ma sabata okwana 36 kapena kuti miyezi 9, ulendo wachiwiri pakatha mwezi umodzi kuyambira tsiku limene mwana wanu anabadwa, ulendo wachitatu patatha miyezi itatu, ulendo wachinayi patatha miyezi isanu ndi umodzi, ulendo wachisanu patatha miyezi isanu ndi inayi ndipo ulendo omaliza (wa nambala sikisi) patatha miyezi khumi ndi iwiri (kapena kuti chaka) chibadwire mwana wanu.

Pamene mudzabwera pa sabata ya nambala 36 kapena kuti mwezi wa nambala 9, tizakuyezani zinthu zosiyana siyana pa thupi lanu, tidzakufunsani mafunso okhuza umoyo wanu komanso thanzi la kaganizidwe kanu ndiponso tidzakutengani magazi. Pa ulendo omweu, tidzayezanso njira yanu ya chibelekero pogwiritsa ntchito ka chipangizo kakang`ono kamene kamakhala ndi thonje kutsogolo kwake kuti tione mitundu ya tizilombo toyambisa matenda timene tingapezeke mu njirayi. Dziwani kuti padzafunika kuti inu komanso ukondedwa anu mulandire chithandizo chamakhwala ngati pangapezeke kuti zosatira zakuyedza zasocheza kuti njira yachibelekero chanu muli tizilombo (zina mwa izo ndi monga matenda opatsirana pogonana). Chomalizira pa ulendo umenewu tidzakufunsani kuti titenge nawo pang`ono chimbudzi chanu. Ndipo mukadzabeleka, munthu mmodzi wa gulu lathu adzabwera kudzatengako pang`ono magazi kuchokera ku thumbo la pa mchombo la mwana komanso kuchokera ku mbali imodzi ya msengwa lachibelekero.

Kuphatikiza apo, adzatenganso ka chiduswa kakang`ono kwambiri ka mbali imodzi ya msengwa la chibelekero kamene ka matuluka limodzi pa nthawi yobeleka. Kupima zithu zimenezi kudzatipatsa ife uthenga odziwa umoyo komanso thanzi la mwana wanu. Kuonjezela apo tidzapimanso mmene mwana wanu akuonekera mmene magazi ake akuyendera, kutakataka, mmene akupumira komanso kumupima zina ndi zina monga kumukweza sikelo kuti tione mmene akulemelela pa nthawi imene wabadwa, kutalika kwake, kutambalala kwa mutu, mmene akumvera ndi makutu ake komanso kupima kapena kumuona ngati ali ndi chilema chilli chonse.

Screening ID:									

Study ID:									

Tidzatenganso chimbudzi cha mwana wanu kuti tikachipime. Ndipo tikatero tidzakufunsaninso kuti mudzabwere pakatha mwezi umodzi, kenako pakatha miyezi itatu, ulendo wina pakatha miyezi isanu ndi umodzi, pakatha miyezi isanu ndi inayi komanso ulendo omaliza pakatha miyezi isanu ndi iwiri (chaka). Mu maulendo amenewa tidzidzakupimani pofuna kuona mmene mukupezela mthupi, tidzidzatengakonso magazi pang`ono komanso tidzidzayeza ndikukweza mwana wanu sikelo. Kuonjezela apo, mu ulendo umene mudzabwera mu mwezi oyamba mwana atabadwa, komanso ulendo umene mudzabwera patatha miyezi isanu ndi umodzi kapena kuti 6 ndi ulendo umene mudzabwera patatha miyezi khumi ndi iwiri kapena chaka, tidzidzatengako mkaka wa mmaere anu kuti tikaupime pofuna kuona mmene michere yowonjezera magazi mthupi ilili komanso thanzi lanu ndi la mwana wanu. Mumaulendo onsewa tidzidzatengakonso chimbudzi chanu komanso cha mwana wanu kuti zikapimidwe. Mukufunsidwanso kuti mudzibwera ku chipatala chino chimene kukuchitikira kafukufukuyu nthawi ina iliyonse pamene mwadwala kudzapimidwa mthupi komanso kuyezetsa magazi.

Kayikidwe ka gulu la mankhwala

Ndi zofunikira kuti mudziwe komanso kumvetsetsa kuti mudzayikidwa mu gulu limodzi mwa la mankhwala mwa mwayi chabe. Dziwani kuti palibe angakhale mmodzi mwa ife amene akudziwa zagulu lomwe mungaikidwe. Muli ndimwayi wofanana kugwera gulu limodzi mwamagulu awiriwa. Muzapatsidwa uthenga onse ofunikira kutsatira molingana ndi gulu limene mwapatsidwa/mwagwera.

Ngati zapezeka kuti muli mu gulu la anthu olandira chithandizo cha mankhwala a mchere owonjezela magazi mthupi a *FCM*, ndiye kuti mudzalandira mankhwalawa kudzela mu diripi yaying`ono imene adzalowesera mankhwalawa kudzela mu jakisoni mu msempha wa pa nkono wanu. Ndipo ngati zapezeka kuti muli mu gulu la anthu olandila chithandizo cha mankhwala a mchere owonjezela magazi lake *iron*, ndiye kuti mudzapatsidwa mpukutu wa mankhwala kuchokera kwa adotolo anu komanso adzakupatsani ndondomeko ya kamwedwe kake ka mankhwalawo.

Chidzachitike ndi chiyani pa mapeto a chithandizo cha mankhwala amene mudzalandira

Pamene takupatsani chithandizo cha mankhwala ulendo oyamba, tidzakufunsani kuti mudzibwera ku chipatala chino chimene kafukufukuyu akuchitikira mu nthawi kapena miyezi imene tidzakupatsani kuti mudzidzalandira zithandizo zosiyanasiyana. Mabweredwe a ku chipatala chino kumene kafukufukuyu akuchitikira agawidwa motere; Ulendo oyamba mudzafika pamene mimba yanu ili ndi miyezi 36 kapena kuti yakwanisa miyezi isanu ndi inayi (9). Ulendo wachiwiri mudzabwera patatha mwezi umodzi kuyambira tsiku limene munabeleka, ulendo wachitatu mudzabwera patatha miyezi itatu, ulendo wachinayi mudzabwera patatha miyezi isanu ndi umodzi (6), ulendo wachisanu mudzabwera patatha miyezi isanu ndi inayi ndipo ulendo omaliza mudzabwera patatha miyezi khumi ndi iwiri (kapena kuti chaka).

Screening ID:									

Study ID:									

Kuonjezera apo, tidzakulimbikitsaninso kuti mudzibwera ku chipatala chino kumene kafukufukuyu akuchitikira nthawi zonse pamene mwadwala. Kuonjezela apo tikukulimbikitsaninso kuti mudzapite kukachilira ku chipatala chomwe mwauzidwa. Dziwani kuti simukuyenera kugawana mankhwalawa ndi mayi mzanu wapakati kapena munthu wina aliyense.

Kodi pali zovuta zina zimene zingathe kubwera chifukwa cha mankhwalawa?

Mankhwala ena ali wonse ali ndi zovuta zina zimene amabweretsa pamene munthu akulandira. Pali mpata ochepa kwambiri kuti mutha kumva zinthu zina mthupi monga, chizungulire pang`ono, kuphwanya malo olumikizana mafupa, komanso kumva kuwawa kwa mutu. Koma izi nthawi zambiri zimasiya zokha popanda kumwera mankhwala. Pamene mankhwala amchere owonjezera magazi mthupi kudzera pokumwa otchedwa *iron* nthawi zina amatha kupangitsa munthu kuzimbidwa, kutsekula mmimba komanso kuwawa kwa mmimba. Amathanso kupangitsa chimbuzi kusandulika chakuda. Koma izi sizoopsa ayi pa moyo wa munthu ngati zitachitika. Pali mwayi wochepa kwambiri kwa munthu yemwe walandira makhwala owonjezera magazi kudzera musepha kuti thupi lake silingayanjane nawo ndipo akhoza kutulaka tizironda mthupi, kuchita nseru, kusika kwakathamangidwe kamagazi mthupi komanso kubanika kupuma. Koma tikufuna kukusimikizirani kuti madokotala athu adaphuzitsidwa bwino mmene angakuthandizireni izi zitachita. Makhwalawa alibe chiopsezo china chirichonse pa mwana yemwe mukuyembekezerayi. Zigawo zili mmusimu zikuthandizani kuti mumvetsetse zovuta zina zimene mankhwala angathe kubweretsa komanso kuti kodi zimenezi zimabwera mowilikiza motani:

Zovuta Zina	Kodi Zingachitike mochuluka bwanji?	Kukula kwa vutoli kungakhale kotani?	Kodi bvutoli limatenga nthawi yayitali bwanji?
Kupweteka kwa mmimba, kumva nseru, kudzimbidwa kapena kutsekula mmimba (pamene tamwa mankhwala otchedwa *Iron*)	Mwina zingachitike kwa munthu mmodzi mwa anthu anayi aliwonse	Pang`ono basi	Kwa masiku ochepa pamene mukumwa mankhwalawa, koma nthawi zambiri munthu amantha kukhala bwinobwino ngakhale osamwa mankhwala

Screening ID:										

Study ID:										

Matenda ena obwera chifukwa cha tidzirombo toyambitsa matenda (monga kutsekula mmimba kapena malungo)	Zimachitika mofanana ndi anthu amene sakumwa mankhwala owonjezela magazi, kapena nthawi zina mochulukilako pang`ono	Mofanana ndi anthu amene sakumwa mankhwala owonjezela magazi kapena nthawi zina mochulukilapo pang'ono	Kwa masiku ochepa
Chimbudzikukhala chakuda (pamene umwa mankhwala	Zimachitika kwa anthu ambiri	Sizoopsa nkomwe	Limakhala pa nthawi imene mankhwalawa akumwedwa basi
Kuphwanya kwa thupi makamaka mu malo olumikizana mafupa (pamene munthu akulandila chithandizo kuzela mu msepha)	Mwina munthu mmodzi mwa anthu makumi awiri (20) kapena munthu mmodzi mwa anthu anthu asanu ndi atatu (8)	Kuphwanya thupi pang`ono mwa patali patali koma nthawi zambiri kumasiya okha	Masiku awiri kapena atatu
Kuchepa kwa mlingo wa mchere umene umapezekanso mmagazi dzina lake fosifeti (kwa amene akulandilira chithandizo cha mankhwala kudzela mu msempha)	Mwina kwa munthu mmodzi mwa anthu khumi (aliwonse)	Pang`onochabe – mwinanso simudzamva kalikonse mthupi mwanu. Ndikusintha chabe kumene kumachitika mu magazi anu ndipo sizoopsa ayi. Komabe tidzakuyezaninso zimenezi.	Ma sabata anayi mwina mpaka masaba asanu ndi limodzi

Kodi kafukufukuyu adzakhuzana bwanji ndi mwana wanga?

Ngati musankha kutenga nawo mbali mu kafukufuku ameneyu ndiye kuti tidzachita monga mmene zimakhalira ku sikelo ya ana, monga kuyeza mwana wanu mmene akulemelera, usinkhu, kutambalala kwa mutu wake komanso kumupima ngati ali ndi zilema kapena matendaotengela pobadwa. Komanso tidzatengako pang`ono chimbuzi choyamba cha mwana wanu. Kuonjezera

Screening ID:									

Study ID:									

apo, tidzayeza ngati mwana wanu ali ndi vuto lina lina lili lonse la kamvedwe pogwiritsa ntchito makina a kompyuta otchedwa *Auditory Brain Response* mu chingerezi. Izi zidzatheka pomuveka mwana wanu makina opangidwa mowoneka ngati timawaya tating′ono komanso tofewa tomwe timagwiritsidwa ntchito kumvetsera nyimbo m`makutu. Kuyeza izi sikumafuna kuti mwana wanu achitepo chili chonse, makamaka zimakhala bwino kuchita izi pamene mwanayo ali kugona mmanja mwa amayi ake. Ka nyimbo ka pansipansi kadzaseweredwa, ndiye mmene mwanayo akuchitira pamene nyimboyi ikuseweredwa zidzaoneka komanso kusungidwa mu chipangizo cha magesi dzina lake *Computer*. Kukula kwa phokoso la nyimboyo kudzakhala kolingana ndi msinkhu wa mwanayo ndipo sizidzakhala zoopsa kwa mwanayo. Ndipo izi sizidzakhala zoopsa kwa mwanayo, komanso chifukwa zimangotenga mphindi zosakwana khumi (10) basi kuti zitheke. Mwana wanu akakwanitsa miyezi itatu (3) komanso khumi ndi masiku awiri (12) tidzamujambula ndi makina a x-ray pachigong′otho ndi mmawondo ndicholinga chofuna kuwunika ngati ali ndi matewe. Izi zizatenga nthawi yochepa chabe basi ndipo sidzimafuna kubaya mwana wanu.

Pakazatha mwezi umodzi tidzakufunsani kuti mudzabwerenso inu ndi mwana wanu kuti tidzakupimeni. Pa nthawi imeneyi, tidzakwezanso mwana wanu sikelo pofuna kuona kulemela kwa mwana wanu komanso kumupima zina ndi zina. Tidzatenganso magazi pang′ono kudzela pa phazi lake kapena kudzela pa nkono wa mwana wanu kuti tiyedze zizindikilo za kusowa kwa magazi kapenanso ngati akupelewera mchere otchedwa *Iron* umene umathandiza kuwonjezela magazi mthupi. Pa tsiku lomweli tidzatengakonso pang`ono chimbuzi cha mwana wanu kuti tikachipime. Kuonjezela apo, pa ulendo umene mudzabwera patangotha mwezi umodzi pamene mwana anabadwa tidzayezanso mamvedwe a mwana wanu pogwiritsa ntchito chipangizo chimene mu chingerezi chimatchedwa kuti *Auditory Brain Response*.

Tidzakufunsaninso kuti mudzabwerenso inu ndi mwana mu mwezi wa chitatu, kenako pakatha miyezi isanu ndi umodzi, pakathanso miyezi isanu ndi inayi ndipo ulendo omaliza mudzabwere pakatha miyezi khumi ndi iwiri (12) kapena kuti chaka. Mu ma ulendo onsewa tidzidzakweza mwana wanu sikelo pofuna kuona kulemela kwake, kumupima mthupi komanso kutengako pang`ono kagazi. Tidzamupimanso kamvedwe kake mmakutu mu ulendo umene mudzabwere patatha miyezi itatu komanso ulendo umene mudzabwera patatha miyezi isanu ndi umodzi. Mu ma ulendo amene mudzabwere patatha miyezi itatu, miyezi isanu ndi umodzi komanso ulendo umene mudzabwere patatha miyezi khumi ndi iwiri (12) kapena kuti chaka, tidzidzatengako pang`ono chimbuzi cha mwana wanu. Kuonjezela pa zopima zimene tafotokozazi, mu ulendo umene mudzabwera patatha miyezi isanu andi umodzi komanso ulendo umene mudzabwere patatha miyezi khumi ndi iwiri (12) kapena kuti chaka, tidzayeza mmene ubongo wa mwana wanu ukugwilira ntchito. Makamaka pofuna kuona monga mmene ubongo wa mwana wanu akuchitila pamene mwana wanu wamva phokoso kapena nyimbo imene, pamene wapatsidwa kapena kuona zithunzi, kuonela kanema kapena masewero osiyanasiyana. Zinthu izi zidzayezedwa pogwiritsa ntchito makina otchedwa *EEG* komanso njira ina yotchedwa kuti *Bayley tests*. Izi zimatheka pomuveka mwana wanu ku mutu makina amenewa omwe anapangidwa mooneka ngati chipewa.

Screening ID:										

Study ID:										

Kenako timamusewelera nyimbo, kumuonetsa chithunzi kapena kanema. Zikatero makina athu amajambula kapena kuonesa changu chimene ubongo wa mwana wanu ukuchitila, kapena kugwirira ntchito pamene wamva nyimbo kapena phokoso, waona kapena kupatsidwa chithunzi komanso pamene waonera kanema. Izi zidzatenga ka mphindi kochepa kokha koma zidzatipatsa ife uthenga okwanira pofuna kuona kuti kodi mankhwala amene tinakupatsani pamene munali oyembekezela athandizapo motani kumbali ya kaganizidwe komanso mmene ubongo wa mwana wanu ukugwirira ntchito. Monga mmene tafotokozera, poyeza izi mwana wanu tidzamuveka makina owoneka ngati chipewa koma chokhala ndi mawaya opangidwa ndi pulasitiki panja pake omwe adzathandizira kutionetsammene ubongo wake ukugwilira ntchito pamene wapatsidwa kapena kuwona chithunzi kapenanso pamene wamva nyimbo kapena phokoso. Pa nthawi imene tizidzayeza izi, mwanayu adzakhala pa miyendo panu mu chipinda mmene tidzayikamo kanema komanso wayilesi yomwe idzidayimba nyimbo. Pa nthawiyi, mwana wanu sadzafunika kuchita chilichonse koma makina amene adzavale kumutuwo ndi amene adzidzayeza kapena kuonesa mmene ubongo wake ukuchitila kapena kugwirira ntchito pamene waona zithunzi kapena wamva nyimbo kapena phokoso. Njira iyi imayeza mmene ubongo wa mwana wanu ukugwirira ntchito powonesa pa makina a kompyuta zinthu zimene zikuchitika mu ubongo wa mwanayo. Inu monga mayi a mwamayu mudzakhala muli mu chipinda chomwecho pamene ntchito yopimayi ikuchitika. Zinthuzi kapena nyimbo zimene zidzagwiritsidwe ntchito popima mwana wanu zaunikilidwa kuti ndi zolingana ndi msinkhu wa mwanayo monga mwa chikhalidwe chathu ku Malawi, ndipo mwana wanu adzasangalala kwambiri powona zithunzizo komanso kumvela nyimbo zimene zidzaseweredwa! Ngati mwana wanu adzayamba kuvuta kapena kulira pa nthawi yomwe tikumuyeza, tidzaimitsa kaye zoyezayezazi ndipo tidzapitiliza pamene mwana wanu watonthola ndi kukhazikika. Mwana wanu sadzamva kupweteka kwina kulikonse panthawi imeneyi ndipo sipadzafunikira kumubaya kapena kutenga magazi. Ndipo chipewa chomwe mwana wanu azavale sichidzamupweteka mu njira ina ili yonse. Monga mmene tafotokozela, palibe chiopsezo china chili chonse pamene mwana wanu wavala chipewa chimenechi komanso ntchitoyi imangotenga mphindi zosakwana makumi awiri basi pa mwana aliyense.

Pali chiopsezo chanji pamene mwana wanga wavala chipewa chimenechi?

Monga mmene tafotokozela, palibe chiopsezo china chili chonse pamene mwana wanu wavala chipewa chimenechi, kungoti kuyezaku kumafunikira kuti mwana wanu avale makina owoneka ngati chipewawa kumutu kwake basi. Mwina nkutheka kuti mwana wanu adzakhala omangika pang`ono pa nthawi imene tikugwira ntchito yoyezayi, komabe madotolo athu amene adzayeza mwana wanu ndi akadaulo omwe akhala akugwira ntchitoyi kwa zaka zambiri ndipo adzaonetsetsa kuti chipewachi chidzakhale chaukhondo pa nthawi yomwe mwana wanu akuvala komanso kuonetsetsa kuti mwana wanu adzakhale okondwa pa nthawi yonse yomwe akuyezedwa. Kuonjezela apo tidzayezanso kapena kumuona mwana wanu mmene akukulira komanso kuchitila mmakhalidwe ake pogwiritsa ntchito njira yotchedwa kuti *Bayley tests.*

Izi zidzachitika pamene mudzabwera ku chipatala kuno mu mwezi wa nambala sikisi komanso mwezi wa khumi ndi iwiri kapena kuti chaka. Njirayi makamaka imayeza kagwiridwe ntchito ka

Screening ID:										
Study ID:										

ubongo wa mwana pa zinthu zina monga, kukumbukira zinthu, chidwi, kayankhula, kukwiya kapena kusangalala komanso kuchitachita ndi zinthu zosiyana siyana monga zidole, pamene wamva nyimbo kapena phokoso komanso kudzindikira malo komanso anthu. Kuyeza izi kumachitika poyika zidole ku tsogolo kwa mwana wanu kuti tione ngati iye angathe kuzizindikira komanso kutha kusewera nazo kapena kuchita nazo china chilichonse. Kuonjezera apo tidzaseweranso masewero osiyanasiyana ndi mwana wanu uku tikuona mmene akuchitira. Zonsezi sizoopsa kwa mwana wanu ayi. Kuwonjezera apo, mwana wanu akatha miyezi itatu (3) komanso isanu ndi umodzi (6) tidzamujambura ubongo wake ndimakina a low field magnetic resonance (MRI; awonetseni makinawo). Izi zidzachitika mwaphindi khumi ziwiri (20 minitsi) ndipo tidzachita izi pamene mwana wanu akugona. Palibe bvuto lina lirilonse limene mwana wanu angakumane nalo chifukwa chakujambulako komanso mwana wanu sazabayidwa. Kujambulaku kudzatithandiza kuti tidziwe mmene ubongo wamwana wanu ukukulira.

Kodi chidzachitike ndi chiyani kwa madzi kapena magazi a mthupi mwanga amene adzatengedwa kukapimidwawo?

Mudzafunsidwa kuti mupereke chilolezo kuti tingathe kutenga nawo magazi anu, madzi kapena zina mu thupi lanu kuti zikapimidwe mu nthawi ya kafukufukuyu. Kutengedwa kwa magazi anu ndi mbali imodzi yofunika komanso yosonyeza kuti inu muli kutenga nawo mbali mu kafukufuku ameneyu. Magazi amene azatengedwa adzapimidwa kufiira kwake komanso kupezeka kwa nchere otchedwa iron kuti tione ngati chithandizo cha mankhwala chimene munalandira chagwira ntchito. Komanso tizawasungabe ndikuwagwiritsa ntchito popima za mmene chitetezo chanu cha mthupi chilili komanso kagwiridwe ntchito ka thupi lanu pofuna kuona kuti zimenezi zingakudze bwanji umoyo wa inu mayi oyembekela komanso umoyo wa mwanayo maka maka polimbana ndi matenda, thanzi komanso kakulidwe. Magazi kapena zina zimene tidzakutengani mthupi zidzasungidwa mowonetsetsa kuti chinsinsi chanu chasungidwa. Izi zidzatheka pogwitsa ntchito nambala ya chinsinsi.

Magazi kapena zina zimene tidzakhala tikutenga mthupi mwanu tidzagwiritsa tchito pofuna kupima chibadwa mtsogolo muno. Koma sitidzayeza matenda ochokera ku makolo ayi, choncho zotsatirazi sidzidzakhuza umoyo wa inu, kapena mwana wanu angakhale umoyo wa anthu a ku banja kwanu. Komanso kuyezaku sikudzalosela kapena kupeza matenda a ku makolo amene agathe kudzabwera mtsogolo. Komano tikuyembekezera kudzayeza kusiyana kwa chibadwa kumene kumakhuzana ndi kupelewera kwa magazi mthupi, thanzi la munthu, kakulidwe ka nsinkhu kapena ziwalo kapena mmene thupi lake limalimbanirana, kapena kugwidwa ndi matenda. Magazi kapena zina zimene tidzakhala tikutenga mthupi lanu ndi kuziyeza komanso zotsatira zake sidzidzapatsidwa kapena kuonetsedwa kwa wina aliyense kupatula madotolo kapena akaswiri okhawo ndi anzawo ena amene akuthandizila nawo kafukufukuyu.

Kodi phindu lotenga nawo mbali mu kafukufuku ameneyu ndi lotani?

Ngati musankha kutenga nawo mbali mu kafukufuku ameneyu ndiye kuti mudzalandila chithandizo cha mankhwala chimene nthawi zonse chimaperekedwa kwa amayi ndi ana. Komanso kuonjezela apo tidzakulimbikitsani kuti mudzibwera ku chipatala chathu chino

Screening ID:										

Study ID:										

kumene kafukufukuyu achichitikira kudzatenga chithandizo cha mankhwala ngati mukudwala makamaka pa nthawi imene kafukufukuyu ali kuchitika. Tikumvetsetsa kuti kubwera ku chipatala kuno ma ulendo ochulukirapo kungathe kukhala kotopetsa. Kotero tidzakupasani ndalama yokwanira MK 7,000.00. (~US$10). Cholinga chake ndi chakuti mubwezeletse pa ndalama yanu ya mayendedwe kapena kuti thiransipoti komanso kugulira chakudya cha masana.

Pamene mukutenga nawo mbali mu kafukufuku ameneyu dziwani kuti mukuthandizira kuti akaswiri a za chipatala apeze njira yabwino komanso yodalilika yothana kapena kuchiza vuto la kuchepa kwa magazi nthupi kumene thawi zina kumathanso kukhala vuto lalikulu makamaka kwa amayi mu nthawi imene ali ndi pakati. Kafukufuku ameneyu atha kubweretsa ubwino waukulu pa nkhondo yolimbana ndi kuchepa kwa magazi mthupi kuno ku Malawi komanso kuthandidza kukonza bwino zotsatira za amayi a pakati ndi ana mtsogolo muno makamaka dela lino la chigawo cha kummwera kwa dziko la Malawi komanso koposaposa dela la kuno kwanu.

Kodi pali chiopsezo cha mtundu wanji pamene ndikutenga nawo mbali mu kafukufuku ameneyu?

Ngati mupanga chisankho chotenga nawo mbali mu kafukufuku ameneyu, chiopsezo kapena zovuta zina ndi zochepa kwambiri. Mwachisanzo, ngati mwayikidwa mu gulu lolandila chithandizo cha makhwala kudzela mu msempha wa pa nkono, ndiye kuti mutha kukhala ndi ka chilonda kakang`ono kapena kumva kuwawa pang`ono pa malo pamene anakubayani jakisoni nkuyika diripi ya mankhwala, kapena pamene anabaya potengako magazi pang`ono. Palinso kuthekera kochepa zedi kuti pa malo pamene anakubayani pangathe kusanduka chilonda. Izi monga mmene tafotokozela zilibe chiopsezo pa thupi lanu chifukwa nthawi zonse timagwiritsa ntchito zipangizo zotetezedwa bwino ndi mankhwala opha ma jelemusi, komanso anthu amene akugwira ntchito mu kafukufuku ameneyu ndi akaswiri komanso ophunzitsidwa bwino ntchito yawo. Tikudziwa kuti kafukufukuyu adzidzafuna kuti mudzabwere kuno ma ulendo ochulukirapo kuposa nthawi zonse. Tikudziwa kuti izi zidzatha kukupangisani kuti nthawi zina mudzikhala otangwanika, komabe tidzidzakubwezelani ndalama ya mayendedwe kapena kuti thiransipoti yanu.

Komanso dziwani kuti chiopsezo kapena kuti zovuta zina pa mwana wanu mu kafukufuku ameneyu ndi chochepa kwambiri. Njira zonse za kupima inu kapena mwana wanu sizidzafuna kuti kubowola kapena kubaya khungu la inu kapena mwana wanu pokha pokha pamene tikufuna kutengako magazi kuti tikawapime. Koma dziwani kuti izi sizidzavulaza mwana wanu. Monga mmene zidzakhalira kwa inu mayi, nayenso mwana adzatha kukhala ndi kachilonda kakang`ono kapena kumva kuwawa pang`ono pa malo pamene tidzabaya ndi kutengako magazi.

Zokhuza Chinsinsi mu Kafukufukuyu

Ngati muvomeleza kutenga nawo mbali mu kafukufukuyu, ife tidzasunga mwachinsinsi dzina lanu komanso la mwana wanu kuphatikizapo uthenga umene tidzapeza kuchokera kwa inu ndi mwana wanu pamene kafukufukuyu ali kuchitika. Malamulo a dziko amafotokoza za kusungira anthu chinsinsi. Komanso chilango chake ngati munthu waphwanya lamuloli. Choncho ifenso

Screening ID:										

Study ID:										

tidzagwira ntchito potsatira malamulowo.

Anthu amene angaone kapena kugwiritsa ntchito uthenga wa inu kapena mwana wanu komanso dzina lanu kapena la mwana wanu ndi okhawo amene avomelezedwa kugwira ntchito mu kafukufuku ameneyu, komanso anthu amene akugwira ntchito yoteteza anzawo mu kafukufuku ameneyu komanso kuphatikizapo akulu akulu a boma ogwira ntchito za chipatala basi. Tidzagwiritsa ntchito uthenga umene mudzatipatsa mu kafukufukuyu basi. Uthenga kapena zotsatira zimene tidzapeza mu kafukufuku ameneyu tingathe kugawana ndi anzathu a ma ofesi ena kapenanso mayika ena koma dziwani kuti sipadzakhala dzina lanu pa mwamba pa uthenga umenewu kapena zolembedwa ku mapeto a kafukufuku ameneyu.

Kodi ndi ndani waunikira kapena kulondoloza mmene kafukufukuyu adzayendere

Malamulo kapena zoyenera kutsatira pamene kafukufukuyu akuchitika zavomerezedwa ndi akomiti kapena gulu la anthu lowona za malamulo a kafukufuku lotchedwa *College of Medicine Ethics Committee kapena* kuti *COMREC* ndi a kafukufuku lotchedwa *National Health Sciences Research Committee* kapena kuti *NHSRC*. Komiti imeneyi imapezeka ku unduna wa za umoyo. Komanso iwowa akuthandidzana ndi komiti yowona za umoyo komanso za malamulo a kafukufuku yotchedwa *Health and Research Ethics Committee* kapena kuti *HREC* imene ili nthambi ya bungwe lotchedwa kuti *Walter and Eliza Hall Institute of Medical Research* imene imapezeka mu mzinda wotchedwa *Melbourne* mu dziko la *Australia*.

Anthu ofunika kuwadziwa mu kafukufuku ameneyu

Kodi ndi ndani amene akupereka thandizo la ndalama za kafukufuku ameneyu?

Amene akupangitsa kapena kuyendesa kafukufuku ameneyu ndi mphunzitsi wankulu dzina lawo ndi a Kamija Phiri amene amagwira ntchito ku kusukulu ya ukachenjede imene maphunzitsa madotolo komanso anamwino kapena kuti ma nesi. Dzina la sukulu imeneyi ndi *College of Medicin*e. Komanso iwowa akugwira limodzi ntchitoyi ndi anzawo a Sant-Rayn Pasricha amene ndi dotolo wankulu ku sukulu yochita kafukufuku wa za umoyo yotchedwa *Walter and Eliza Hall Institute of Medical Research*. Koma chithandizo cha ndalama choyendetsera kafukufukuyu chikuchokera ku bungwe lotchedwa kuti *Bill and Melinda Gates Foundation* limene limapezeka mu dziko la America.

Uthenga owonjezera komanso amene mungathe kulumikizana nawo

Ngati mukufuna uthenga wina uliwonse owonjezera okhuza kafukufukuyu, kapena mwina ngati mmodzi wa otenga nawo mbali mu kafukufuku ameneyu wadwala mwina chifukwa chakuti mankhwala amene wamwa abweletsa zovuta zina mthupi monga mmene tinafotokozera poyamba paja, mukhoknza kutifunsa lero pompano, kapena kudzatifunsa nthawi imene tabwera kuno mtsogolomu. Koma ngati mudzakhala ndi mafunso ena mtsogolomu mukhonza kulumikizana ndi mphunzitsi wankulu a Kamija Phiri amenenso ndi wankulu wa kufukufuku ameneyu. Iwowa amapezeka ku sukulu yotchedwa *College of Medicine* ku Blantyre. Mutha kuwayimbira foni pa nambala iyi; +265999957048. Kapena muthanso kulumikizana ndi anzawo

Screening ID:									

Study ID:									

amene ndi dotolo wankulu, dzina lawo ndi a Sant-Rayn Pasricha poyimba foni pa nambala iyi; +61393452618

Iwowanso ndi nkulu wa kafukufuku koma amachokera ku sukulu yochita za kafukufuku wa za umoyo yotchedwa *Walter and Eliza Hall Institute of Medical Research* imene imapezeka mu dziko la Australia.

Kumbali yokhuza chidandaulo chilichonse makamaka chokhuzana ndi kafukufukuyu mutha kukaonana ndi amene ayikidwa kuti aziona za chidandaulo ku chipatala chimene mwana wanu akutenga nawo gawo mu kafukufukuyu. Ndipo pezani mmunsimu udindo, dzina komanso nambala ya foni ya amene mungathe kulumikizana nawo kuti mupereke chidandaulo chanu.

Kulumikizana ndi munthu wa za umoyo ndi munthu owona za chidandaulo kumalo amene kafukufuku akuchitikira

Dzina	*Zinenani Truwah*
Udindo	*Olondoloza ntchito za kafukufuku, Training and Research Unit ofExcellence*
Nambala ya lamya kapena foni	*+265999413775 kapena +2650882091578*
Njira kapena adiresi yotumizira kalata pa compyuta kapena kuti intaneti	*zinenanitruwah@gmail.com*

Ngati muli ndi madandaulo a zina ndi zina zokhuza kafukufukuyu, monga mmene ntchitoyi ikuyendera, kapena mafunso aliwonse ochokera kwa inu monga mmodzi wa anthu otenga nawo mbali mutha kulumikizana ndi anthu a ku ofesi kudzela mu ma nambala a foni ali mmunsiwa.

Dzina la owunikira za umoyo komanso za malamulo a kafukufuku	**National Health Sciences Research Committee**
Oyimirira komiti yowunikira za umoyokomanso za malamulo a kafukufuku	*Dr. Collins Mitambo*
Nambala ya lamya kapenakuti foni	*+265999397913*
Njira kapena adiresi yotumizira kalata pa compyuta kapena kuti intaneti	*cmitambo@gmail.com*

Screening ID:										

Study ID:										

Kuvomera kwa otenga nawo mbali mu kafukufukuyu kapena kuvomera kudzela kwa owayimilira

- ➢ Ndikuvomera kuti ndawerenga kalata ya chilolezo kapena kuti munthu wina wandiwerengera mu Chichewa
- ➢ Ndikuvomera kuti ndamvetsetsa zolinga za kafukufukuy, njira zimene zidzatsatidwa, komanso zovuta zina zimene zingathe kubwera pamene munthu watenga nawo mbali, komanso monga mmene zafotokozeledwa ku kalatayi yokhuza kafukufukuyu
- ➢ Ndikuvomera kuti ndipereka chilolezo kwa ma dotolo anga komanso ma dotolo a mwana wanga kuphatikiza akaswiri ena a za umoyo, zipatala kapena nyumba zopimila matenda kupatula chipatala chino kuti angathe kutulutsa kapena kupeleka zotsatira za kafukufukuyu, monga umoyo wa mwana wanga komanso zokhuza chithandizo chamankhwala kwa akaswiri ochokera ku sukulu ya ukachenjede yotchedwa College of Medicine komanso akaswiri a za chipatala ochokera ku sukulu yotchedwa *Walter and Eliza Hall Institute of Medical Research*. Izi zidzachitika makamaka pofuna kukwanilitsa cholinga cha kafukufukuyu. Ndipo ndikudziwa kuti uthenga wa ine komanso okhuza umoyo wa mwana wanga udzakhala wa chinsinsi ndipo kuti sudzapatsidwa kwa munthu wamba
- ➢ Ndikuvomera kuti ndinali ndi mpata ofunsa mafunso ndipo ndakhutira ndi mayankho amene ndalandira Mwakufuna kwanga ndikuvomereza kutenga nawo mbali komanso kuti mwana wanga athanso kutenga nawo mbali mu kafukufuku ameneyu monga mmene zafotokozeledwa, komanso ndikumvetsa kuti ndine omasuka kusiya kutenga nawo mbali chimodzimodzinso mwana wanga nthawi ina iliyonse kafukufukuyu ali mkati popanda kuopa kwina kuli konse pa zokhuza chithandizo cha ku chipatala kwa ine mayi ngakhalenso mwana wanga mtsogolomu.
- ➢ Ndikumvetsa kuti ndidzapatsidwa kalata iyi ya chilolezo komanso yokhuza za kafukufukuyu kuti ndisunge ine mwini.

Chiphaso chachilolezo:

A. Wotenga nao mbali amene ali opitilira zaka 18 zakubadwa amene amadziwa kuwerenga:

Lembani dzina lawotenga nawo mbali: ____

____ ______________________

Saini ya otenga nao mbali Tsiku/mwezi/chaka

Screening ID:									

Study ID:									

B. Wotenga nawo mbali amene ali opitilira zaka 18 zakubadwa amene samadziwa kuwerenga:

Saini yamboni

(Saini ya mboni ndi chidindo chawotenga nawo mbali ndizofunika pokhapokha ngati otenga nawo mbali ndiwosadziwa kulemba ndi kuwerenga. Izi zikakhala chomwechi, mboni yodziwa kulemba ndikuwerenga ikuyenera kusaina. Ngati nkotheka, munthu ameneyu akuyenera kusankhidwa ndiwotenga nawo mbali ndipo sakuyenera kukhala pa ubale ndiwogwira ntchito mukafufukufuku).

Ndawonelera kuwerenga bwino kwa kalata yachilolezo kwa amene atenge nawo mbali, amene anapatsidwa nthawi yofunsa mafunso. Ndikutsimikiza kuti otenga nawo mbali wavomera mwa kufuna kwake.

Lembani dzina la mboni ______________________________

Saini la mboni: ______________________________

Tsiku (Tsiku/mwezi/chaka) ______________________________

Ndi chidindo cha chala chachikulu cha ku manja cha wotenga nawo mbali

C. Wotenga nawo mbali wosapitilira zaka 18 zakubadwa

1. Saini ya kholo kapena womuyang'anira wovomerezeka:

Mwana wanga wayitanidwa kuti atenge nawo mbali mukafukufuku wamankhwala ogwiritsidwa ntchito poteteza kuchepa kwa magazi panthawi yomwe ali woyembekezera.

Ndawerenga/ndawerengeredwa uthenga omwe uli pamwambapa. Ndinapatsidwa nthawi yofunsa mafunso, ndipo mafunso ena aliwonse omwe ndinafunsa ndayankhidwa ndipo ndakhutitsidwa. Ndikuvomera mwakufuna kwanga kuti mwana wanga atenge nawo mbali mukafukufukuyu.

Dzina la kholo ______________________________

Saini la kholo: ______________________________

Screening ID:									

Study ID:									

Tsiku (Tsiku/mwezi/chaka) ____

Ngati kholo kapena womuyang'anira wovomerezeka sadziwa kulemba
Ndawonelera kuwerenga bwino kwa kalata yachilolezo kwa:

____

(Lembani dzina la kholo kapena womuyang'anira wovomerezeka. Dzinali lilembedwe ndi mboni osati ogwira mukafukufuku)

Ndikutsimikiza kuti anapatsidwa nthawi yofunsa mafunso ndipo wavomera kutenga nawo mbali mwa kufuna kwake.

Dzina la mboni ____

Saini la mboni: ____

Tsiku (Tsiku/mwezi/chaka) ____

Chidindo cha chala chachikulu cha ku manja (makolo amene sadziwa kulemba)

2. Saini ya wotenga nawo mbali:

Ndayitanidwa kuti nditenge nawo mbali mukafukufuku wamankhwala ogwiritsidwa ntchito poteteza kuchepa kwa magazi panthawi yomwe ndili woyembekezera.

Ndawerenga/ndawerengeredwa uthenga omwe uli pamwambapa, Ndinapatsidwa nthawi yofunsa mafunso, ndipo mafunso ena aliwonse omwe ndinafunsa ndayankhidwa ndipo ndakhutitsidwa. Ndikuvomera mwakufuna kwanga kuti kutenga nawo mbali mukafukufukuyu. Ndikuzindikira kuti nditha kusankha kusatenga nawo mbali mukafukufuku ngakhale kholo litavomera kutero.

Lembani dzina la wotenga nao mbali:

____

Screening ID:									

Study ID:									

Saini la wotenga nao mbali:

____

Tsiku (Tsiku/mwezi/chaka)

____

Chidindo cha chala chachikulu cha ku manja (amene sadziwa kulemba)

D. Wotenga nawo mbali yemwe sadapereke chilolezo chogwiritsa uthenga komanso zomwe adayezedwa ngati magazi mukafukufuku watsogolo.

Saini kapena chidindo

____
Tsiku/mwezi/chaka

______________________________________ ______________________

Saini ya *PI* kapena omuyimirira Tsiku/mwezi/chaka

NB: Chikalata china chachilolezo chaperekedwa kwa wotenga nawo mbali mukafukufukuyu

PARTICIPANT SCREENING ID | | | | | | | | | | |

Date | | | _ | | | _ | | | | |

Visit | 0 | SCREENING |

Participant's Name:

Date of Birth: *dd/mmm/yyyy*

Residential Area:

1.	Does the participant remember their last normal menstrual period (LMNP)?	☐ (0) No<br>☐ (1) Yes	If yes insert date *dd/mmm/yyyy*
2.	Is the gestation age between 27-35 weeks by Last Normal Menstrual Period (LNMP) or physical examination (e.g., fundal height)?	☐ (0) No<br>☐<br>(1) Yes	If yes, insert gestation age<br>Weeks ☐☐<br>Days ☐
3.	Is it singleton pregnancy by physical examination?	☐ (0) No<br>☐ (1) Yes	
4.	Capillary Haemoglobin (Hb) g/dl	_____.__	
5.	Negative malaria test (MRDT)	☐ (0) No<br>☐<br>(1) Yes	(If participant has malaria, treat according to national guidelines and ask them to come back in one week for re-screening)
6.	Any clinical symptoms of acute infection?	☐ (0) No<br>☐ (1) Yes	
7.	Any Condition requiring immediate hospitalisation?	☐ (0) No<br>☐(1) Yes	
8.	Evidence of Pre-Eclampsia?	☐ (0) No<br>(1) Yes	
9.	History of diabetes?	☐ (0) No<br>☐<br>(1) Yes	
10.	Known sickle cell (SC) disease or sickle-haemoglobin C (SHC) anaemia?	☐ (0) No<br>(1) Yes	
11.	Hypersensitivity to study drugs?	☐ (0) No<br>(1) Yes	
12.	Disagrees to study procedures?	☐ (0) No<br>☐<br>(1) Yes	

**PARTICIPANT SCREENING ID**											
**Date**			_				_				
**Visit**	**0**	**SCREENING**									

13.	Does the participant have any reasons warranting exclusion according to investigator's assessment?	☐ (0) No<br>☐<br>(1) Yes	If Yes, indicate reason

STAFF ID:

VISIT 0 (DAY 0): Post-Malaria SCREENING

**PARTICIPANT SCREENING ID**	☐☐☐☐☐☐☐☐
**Date:**	☐☐-☐☐☐-☐☐☐☐
**Visit**	0 SCREENING

Prescreening after malaria treatment
(*This form should only be filled if potential participant had a positive malaria during the first pre-screening visit, was treated according to national standard treatment guidelines, and accepted to come back after a week for repeat screening)*

Does the participant have any of the following?		
01	Clinical symptoms of malaria<br>(If participant has malaria, treat according to national guidelines and ask them to come back in one week for re-screening)<br><br>If yes	☐ (0) No ☐ (1) Yes<br><br>(1) Fever<br>(2) Chills<br>(3) Headache<br>(4) Myalgia (joint pain)<br>(5) Malaise
02	Positive malaria parasitaemia (Microscopy)	☐ (0) No<br>(1) Yes<br>☐ (2) Not done
03	Capillary Hb <5g/dl or >9.9g/dl	☐ (0) No<br>(1) Yes<br><br>HB ☐☐.☐ g/dl
If "Yes" to any of the above, the subject is NOT eligible for the study		

STAFF ID ☐☐☐☐

PARTICIPANT SCREENING ID | | | | | | | | | | |

Date | | | - | | | | | | | | |

Visit | 1 | **ENROLMENT** |

Informed consent process

(Please ensure the Consent Form used is the most recent version approved by NHMRC)

01. The participant chose to be consented in: ☐ (1) English ☐ (2) Chichewa

02. Husband Present During Consenting, if participant is married?

☐ (0) No ☐ (1) Yes ☐ (2) N/A

If no, the participant should be allowed to discuss with the husband before consenting. Strongly suggest participant contacts key household decision maker.

03. Resides within study catchment area and will be available during the entire study period
☐ (0) No ☐ (1) Yes (If 'NO' subject is NOT eligible for the study)

04. Accepts study procedures

☐ (0) No ☐ (1) Yes
(If 'NO' subject is NOT eligible for the study)

05. Accepts to stop taking other iron tablets or supplements that may have been provided before?

☐ (0) No ☐ (1) Yes
(If 'NO' subject is NOT eligible for the study)

06. Signed an informed consent form

☐ (0) No ☐ (1) Yes
(If 'NO' subject is NOT eligible for the study)

07. Assigned Study ID | | | | | | | | | | |

(**Note:** Use assigned Study ID from now on)

STAFF INITIALS ______________________________

**PARTICIPANT SCREENING ID**										

**Date**			-								

**Visit**	**1**	**ENROLMENT**

**STUDY ID**												
**Date**				-				-					
**Visit**	1 ENROLMENT

Demography

In this section I am going to ask you information on your background. Please feel free to ask me where you do not understand for clarification.

01.	What is your tribe?	☐ *(1) Chewa*<br>☐ *(2) Yao*<br>☐ *(3) Tumbuka*<br>☐ *(4) Lomwe*<br>☐ *(5) Sena*<br>☐ *(6) Tonga*<br>☐ *(7) Ngonde*<br>☐ *(8) Nyanja*<br>☐ *(9) Ngoni*<br>☐ *(10) Mang'anya*<br>☐ *(11) Other, Specify________________*
02.	Have you ever attended formal school?	☐ *(0) No (**if no skip to Q04**)*<br>☐ *(1) Yes*
03.	How far did you go with your education?	☐ *(0) None*<br>☐ *(1) Lower Primary (1-5)*<br>☐ *(2) Upper Primary (6-8)*<br>☐ *(4) Lower Secondary (1-2)*<br>☐ *(5) Upper Secondary (3-4)*<br>☐ *(6) Tertiary*
04.	Are you able to read?	☐ *(0) No*<br>☐ *(1) Yes*
05.	Are you able to write?	☐ *(0) No*<br>☐ *(1) Yes*
06.	What is your current marital status?	☐ *(1) Single/Never married*<br>☐ *(2) Married*<br>☐ *(3) Widowed*<br>☐ *(4) Divorced/Separated*<br>☐ *(5) Other Specify__________________*
07.	What religion do you practice?	☐ (1) Catholic<br>☐ (2) CCAP<br>☐ (3) Anglican<br>☐ (4) Seventh Day Adventist<br>☐ (5) Muslim<br>☐ (6) No Religion<br>☐ (7) Others, specify:

**STUDY ID**	| | | | | | | | | | |
**Date**	| | | - | | | - | | | | |
**Visit**	1 **ENROLMENT**

08.	What is the main source of income of your household?	☐ (0) None<br>☐ (1) Subsistence farming<br>☐ (2) Large scale farming<br>☐ (3) Employed<br>☐ (4) Casual work for wages<br>☐ (5) Business<br>☐ (6) Other; specify
09.	Has the participant being given insecticide treated bed net (ITN)	☐ (1) Yes<br>☐ (0) No

STAFF INITIALS ______________________________

**STUDY ID**												
**Date**				-				-					
**Visit**	1 **ENROLMENT**

Obstetric Assessment

In this section, I am going to ask information concerning your current pregnancy and any other previous pregnancies. Feel free to ask me any question as I am examining you if you feel so.

Gynaecological History		
01.	Gravidity - How many pregnancies have you had including this one? (**If first pregnancy skip to 11**)	☐☐
02.	Parity - How many of those pregnancies did you carry for more than 28 weeks? **(if 0 skip to 06)**	☐☐
03.	At what age were you at delivery of first pregnancy? (how old were you at the delivery of your first pregnancy)	☐☐
04.	How many deliveries resulted in a live baby?	☐☐
05	How many deliveries resulted in a stillbirth?	☐☐
06.	Number of spontaneous abortions/miscarriages	☐☐
07.	Number of vaginal deliveries	☐☐
08.	Number of C-Sections	☐☐
09.	Did you have any problems/complications during any of your deliveries?<br><br>**If No skip to Q11**	☐ **(0)** No **Skip to Q11**<br>☐ (1) Yes<br>☐ (99) **N/A Skip to Q11**

**STUDY ID**											
**Date**			-				-				
**Visit**	1	ENROLMENT									

10.	If yes, what complications did you have? Please circle whatsoever applies.	☐ (1) Cord prolapse/cord around the neck<br>☐ (2) Perineal lacerations<br>☐ (3) Abnormal foetal heart rate or rhythm<br>☐ (4) Amniotic cavity issues/Premature rupture of membrane<br>☐ (5) Failure to progress/Obstructed labour<br>☐ (6) Antepartum or Postpartum haemorrhage<br>☐ Others; specify
11.	Is there a family history of: tick all that applies?	☐ (1) Congenital abnormalities/genetic disease<br>☐ (2) Consanguinity<br>☐ (0) None applicable

Significant medical history

12.	Does the participant have any of these conditions: tick all that applies	
	**Disease syndrome**	**Status**
	Hypertension	☐ (0) No<br>☐ (1) Previous pregnancies<br>☐ (2) Current pregnancy
	Diabetes	☐ (0) No

**STUDY ID**	
**Date**	- -
**Visit**	1 **ENROLMENT**

		☐ (1) Previous pregnancies<br>☐ (2) Current pregnancy
	HIV Positive	☐ (0) No ☐ (1) Yes
	If HIV positive, are you on ARTs?	☐ (0) No ☐ (1) Yes
	If on ARTs, which type?	☐ (1) 5A<br>☐ (2) Others (specify)
	Anaemia in previous pregnancies	☐ (0) No ☐ (1) Yes ☐ (99) NA
	Syphilis (VDRL) positive?	☐ (0) No ☐ (1) Yes<br>☐ (99) Unknown/Not done
	If yes treatment given?	☐ (0) No ☐ (1) Yes
	Other Specify<br>_____________________________	☐ (0) No ☐ (1) Yes
13.	Has the participant received their vaccinations?	
	**Vaccination History**	**Status**
	TTV: Pregnancy vaccines during current pregnancy	☐ (0) None<br>☐ (1) (To be completed)<br>☐ (2) (To be completed)
	TTV: Pregnancy vaccines during previous pregnancy	☐ (0) None<br>☐ (1) (To be completed)<br>☐ (2) (To be completed)

**STUDY ID**										

**Date**			-				-				

**Visit**	**1**	**ENROLMENT**

	COVID-19 vaccine: Have you received COVID-19 vaccination	(0) No (1) Yes
	If yes, which one?	
	If yes, how many doses?	(0) 1<br>(1) 2<br>(2) Don't know

History of drug allergies

17.	Have you ever experienced an allergic reaction to any drug?	☐ (0) No<br>☐ (1) Yes
	If yes	
	Drug Name/Class	Type of Reaction

STAFF INITIALS ____

**STUDY ID**	☐☐☐☐☐☐☐☐☐☐
**Date**	☐☐-☐☐☐-☐☐☐☐
**Visit**	☐

Physical Examination

Vital signs: This time I would like to check your blood pressure, body temperature, pulse rate and respiratory rate

☐ Not done

01.	Sitting position Blood Pressure	____________/____________mmHg<br>Systolic Diastolic
02.	Pulse Rate	____________beats/minute
03.	Respiratory rate	____________cycles/minute
04.	Axillary Temperature	________.______degrees Celsius
05.	Weight 1<br>Weight 2<br>Weight 3	☐☐☐.☐☐Kgs<br>☐☐☐.☐☐Kgs<br>☐☐☐.☐☐Kgs
06.	Height 1<br>Height 2<br>Height 3	☐☐☐.☐☐cm<br>☐☐☐.☐☐cm<br>☐☐☐.☐☐cm
	Staff ID (Indicate initials for the staff who checked the measurements above)	

			**If abnormal specify**
07.	General Appearance	☐ (1) Normal<br>☐ (2) Abnormal	

STUDY ID | | | | | | | | | | |

Date | | | - | | | - | | | | |

Visit | | |

		☐ (3) Not done	
08.	Eyes	☐ (1) Normal<br>☐ (2) Abnormal<br>☐ (3) Not done	
09.	Mouth	☐ (1) Normal<br>☐ (2) Abnormal<br>☐ (3) Not done	
10.	Neck	☐ (1) Normal<br>☐ (2) Abnormal<br>☐ (3) Not done	
11.	Thyroid	☐ (1) Normal<br>☐ (2) Abnormal<br>☐ (3) Not done	
12.	Musculoskeletal	☐ (1) Normal<br>☐ (2) Abnormal<br>☐ (3) Not done	
13.	Extremities	☐ (1) Normal<br>☐ (2) Abnormal<br>☐ (3) Not done	
14	Skin	☐ (1) Normal<br>☐ (2) Abnormal<br>☐ (3) Not done	
15.	Other (specify)	☐ (1) Normal<br>☐ (2) Abnormal<br>☐ (3) Not done	

Comments:________________________

STAFF INITIALS____

**STUDY ID**	☐☐☐☐☐☐☐☐☐☐
**Date**	☐☐-☐☐☐☐-☐☐☐☐
**Visit**	☐

Physical Examination

Vital signs: This time I would like to check your blood pressure, body temperature, pulse rate and respiratory rate

☐ Not done

01.	Sitting position Blood Pressure	______/______mmHg<br>Systolic Diastolic
02.	Pulse Rate	______beats/minute
03.	Respiratory rate	______cycles/minute
04.	Axillary Temperature	______.______degrees Celsius
05.	Weight 1<br>Weight 2<br>Weight 3	☐☐☐.☐☐Kgs<br>☐☐☐.☐☐Kgs<br>☐☐☐.☐☐Kgs
06.	Height 1<br>Height 2<br>Height 3	☐☐☐.☐☐cm<br>☐☐☐.☐☐cm<br>☐☐☐.☐☐cm
	Staff ID (Indicate initials for the staff who checked the measurements above)	

			**If abnormal specify**
07.	General Appearance	☐ (1) Normal<br>☐ (2) Abnormal	

**STUDY ID**	☐☐☐☐☐☐☐☐☐☐
**Date**	☐☐ - ☐☐ - ☐☐☐☐
**Visit**	☐ ☐

		☐ (3) Not done	
08.	Eyes	☐ (1) Normal<br>☐ (2) Abnormal<br>☐ (3) Not done	
09.	Mouth	☐ (1) Normal<br>☐ (2) Abnormal<br>☐ (3) Not done	
10.	Neck	☐ (1) Normal<br>☐ (2) Abnormal<br>☐ (3) Not done	
11.	Thyroid	☐ (1) Normal<br>☐ (2) Abnormal<br>☐ (3) Not done	
12.	Musculoskeletal	☐ (1) Normal<br>☐ (2) Abnormal<br>☐ (3) Not done	
13.	Extremities	☐ (1) Normal<br>☐ (2) Abnormal<br>☐ (3) Not done	
14	Skin	☐ (1) Normal<br>☐ (2) Abnormal<br>☐ (3) Not done	
15.	Other (specify)	☐ (1) Normal<br>☐ (2) Abnormal<br>☐ (3) Not done	

Comments:________________________

STAFF INITIALS____

STUDY ID ☐☐☐☐☐☐☐☐☐☐

Date ☐☐-☐☐☐-☐☐☐☐

Visit ☐ ☐

Physical Examination

Vital signs: This time I would like to check your blood pressure, body temperature, pulse rate and respiratory rate

☐ Not done

01.	Sitting position Blood Pressure	________/________mmHg<br>Systolic Diastolic
02.	Pulse Rate	__________beats/minute
03.	Respiratory rate	__________cycles/minute
04.	Axillary Temperature	________.______degrees Celsius
05.	Weight 1<br>Weight 2<br>Weight 3	☐☐☐.☐☐Kgs<br>☐☐☐.☐☐Kgs<br>☐☐☐.☐☐Kgs
06.	Height 1<br>Height 2<br>Height 3	☐☐☐.☐☐cm<br>☐☐☐.☐☐cm<br>☐☐☐.☐☐cm
	Staff ID (Indicate initials for the staff who checked the measurements above)	

			**If abnormal specify**
07.	General Appearance	☐ (1) Normal<br>☐ (2) Abnormal	

STUDY ID | | | | | | | | | | |

Date | | | - | | | - | | | | |

Visit | | |

		☐ (3) Not done	
08.	Eyes	☐ (1) Normal<br>☐ (2) Abnormal<br>☐ (3) Not done	
09.	Mouth	☐ (1) Normal<br>☐ (2) Abnormal<br>☐ (3) Not done	
10.	Neck	☐ (1) Normal<br>☐ (2) Abnormal<br>☐ (3) Not done	
11.	Thyroid	☐ (1) Normal<br>☐ (2) Abnormal<br>☐ (3) Not done	
12.	Musculoskeletal	☐ (1) Normal<br>☐ (2) Abnormal<br>☐ (3) Not done	
13.	Extremities	☐ (1) Normal<br>☐ (2) Abnormal<br>☐ (3) Not done	
14	Skin	☐ (1) Normal<br>☐ (2) Abnormal<br>☐ (3) Not done	
15.	Other (specify)	☐ (1) Normal<br>☐ (2) Abnormal<br>☐ (3) Not done	

Comments:________________________

STAFF INITIALS____

**STUDY ID**	☐☐☐☐☐☐☐☐☐☐
**Date**	☐☐ - ☐☐ - ☐☐☐☐
**Visit**	1 **ENROLMENT**

Participant Randomization

☐ Not done

01.	Which arm has the participant been randomised?	**1.** ☐ **Oral iron**<br>**2.** ☐ **FCM**
	Time of randomisation	☐☐:☐☐<br>Hrs : Mins
**Medication Administration**		
	**Study Medications**	**Time of dose**
02.	FCM	Dose____________________<br>Start time ☐☐:☐☐ Hrs : Mins<br>Finish time ☐☐:☐☐ Hrs : Mins
	IV drug given by	☐ (1) Government staff<br>☐ (1) Research staff
	Name of staff giving IV iron: ____________________<br>Cadre: ____________________	
03.	If oral Iron, 90-day supply given?<br>Information to be given to participant:<br>Drug name: F/S<br>Frequency: twice a day and report to facility if experiencing any problem with medication	☐ (0) No ☐ (1) Yes
	If not given, give reasons:	____________________

Adverse events (This section should not come up if the participant is in the oral arm)

During and after infusion

**STUDY ID**											
**Date**			-				-				
**Visit**	1	ENROLMENT									

(Note: Record any adverse events occurring during and after infusion. Prompt for any symptoms of adverse events from the participant by asking the following open ended question during and after infusion)

How are you feeling?

Did the participant experience any of the following?

Tick all that apply:
- ☐ (0) None
- ☐ (1) Headache
- ☐ (2) Dizziness
- ☐ (3) Discolouration of the skin
- ☐ (4) Nausea
- ☐ (5) Vomiting
- ☐ (6) Upper abdominal pain
- ☐ (8) Dyspepsia
- ☐ (9) Flushing
- ☐ (10) Shortness of breath
- ☐ (11) Chest pains
- ☐ (12) Anaphylactic shock
- ☐ (13) Others: specify

(If any of these apply, fill the adverse event form) If any of these is ticked than there should be an option to move to the AE form.

STAFF INITIALS ______________________________

**STUDY ID**										

**Date**								

**Visit**	1	**ENROLMENT**

PARTICIPANTS INFORMATION

Participant's Name:

Residential Address:

Contact Number (Mobile):

Alternative Contact Number (Mobile):

PHYSICAL ADDRESS
(Record GPS Coordinates – CRF can be closed without this information)

Village/Neighbourhood:

Residential address:

Description of home address

STAFF ID ☐☐☐☐

**STUDY ID**	_ _ _ _ _ _ _ _ _ _
**Date**	_ _ - _ _ _ - _ _ _ _
**Visit**	_ _______

MEDICATION DURING PREGNANCY

Record medication given on this visit or at health centre during pregnancy		
01.	Sulphadoxine Pyrimethamine (SP)	☐ ***(0)*** No ***(if no specify__________________)Skip to Q.03***<br>☐ **(1)** Yes **move to Q.02**<br>☐ (99999) N/A
02	How many doses have you received in total?	☐ (1) 1<br>☐ (2) 2<br>☐ (3) 3<br>☐ (4) 4 or more
03.	Are you taking any pregnancy supplements? (Tick all that apply)	☐ (0) No<br>☐ (1) Folic Acid + Iron tablets<br>☐ (2) Micronutrient powders<br>☐ (2) Vitamin A
04.	Albendazole	☐ ***(0)*** No ***(if no specify__________________________)***<br>☐ (1) Yes<br>☐ (99999) N/A

STUDY ID

Date

		-				-				

Visit

Any other treatment given on this visit

Medication	Start Date DD/MMM/YYYY	Stop Date DD/MMM/YYYY	Dose and route	Diagnosis/Indication

**STUDY ID**	☐☐☐☐☐☐☐☐☐☐
**Date**	☐☐ - ☐☐☐ - ☐☐☐☐
**Visit**	☐ ☐

MEDICATION DURING PREGNANCY

Record medication given on this visit or at health centre during pregnancy		
01.	Sulphadoxine Pyrimethamine (SP)	☐ ***(0)*** No ***(if no specify_________________)Skip to Q.03***<br>☐ **(1)** Yes **move to Q.02**<br>☐ (99999) N/A
02	How many doses?	☐ (1) 1<br>☐ (2) 2<br>☐ (3) 3<br>☐ (4) 4 or more
03.	Are you taking any pregnancy supplements? (Tick all that apply)	☐ (0) No<br>☐ (1) Folic Acid + Iron tablets<br>☐ (2) Micronutrient powders<br>☐ (2) Vitamin A
04.	Albendazole	☐ ***(0)*** No ***(if no specify________________________)***<br>☐ (1) Yes<br>☐ (99999) N/A

STUDY ID

Date

		-				-				

Visit

Any other treatment given on this visit

Medication	Start Date DD/MMM/YYYY	Stop Date DD/MMM/YYYY	Dose and route	Diagnosis/Indication

**STUDY ID**												
**Date**			-			-					
**Visit**	7 **DELIVERY**

Section A: Physical Examination (consider merging with 07. TT-maternal physical examination_v1.1)

Attach copies of Labour-ward Admission notes and partograph

VITAL SIGNS

☐ Not done

	Sitting position Blood Pressure	____________/______________mmHg<br>Systolic Diastolic
	Pulse Rate	_________________ beats/minute
	Respiratory rate	
	Axillary Temperature	_______________.___degrees Celsius

OBSTETRICAL EXAMINATION

☐ Not done

	Fetal heart rate	☐☐☐ bpm
	Has participant felt any fetal movements in the past 24 hours	☐ (1) Yes<br>☐ (2) No
	Investigators assessment of reported fetal movement	☐ (1) Normal<br>☐ (2) Abnormal
	Fetal presentation	(Tick ONE only)<br>☐ (1) Cephalic (head down) position<br>☐ (2) Breech (bottom down) position<br>☐ (3) Transverse<br>☐ (4) Unknown

STAFF ID ____

**STUDY ID**										
**Date**			-			-				
**Visit**	7	**DELIVERY**								

Pregnancy outcome

1. Pregnancy outcome

☐ (1) Full term live birth
☐ (2) Premature Birth
☐ (3) Stillbirth (**if 4, skip to Question 14**)
☐ (4) Spontaneous abortion (**if 4, skip to Question 14**)
☐ **(5)** Induced/elective abortion (**if 5, skip to Question 14**

2. **Location of Delivery:**
   ☐ (1) Zomba Central Hospital
   ☐ (2) Likangala Health Centre
   ☐ (3) Bimbi Health Centre
   ☐ (4) Lambulira Health Centre
   ☐ (5) Naisi Health Centre
   ☐ (6) Domasi Health Centre
   ☐ (7) Home
   ☐ (8) Others, Specify

3. **Mode of delivery:** ☐ (1) Vaginal ☐ (2) Cesarean Section ☐ (3) Assisted delivery

4. **Date of Delivery:** ☐☐/☐☐/☐☐☐☐ **dd/mm/yyyy**

5. If Cesarean Section, what was the indication?

   ____
   ____
   ____

6. After how many minutes was cord clamping done?

   ____

   Delivery assisted by: ______________________________
   Cadre: ______________________________

7. **Was labour induced?** ☐ (1) Yes ☐ (0) No

If yes Comment:

____
____
____

8. **Was there any complication experienced during delivery?** ☐ (1) Yes ☐ (0) No

**STUDY ID**										

**Date**			-				-				

**Visit**	7	**DELIVERY**

(If any provide on the adverse events page)
☐ (1) Cord prolapse/cord around the neck
☐ (2) Perineal tear (Degree I-IV)
☐ (3) Abnormal fetal heart rate or rhythm
☐ (4) Amniotic cavity issues/Premature rupture of membrane
☐ (5) Failure to progress/Obstructed labour
☐ (6) Antepartum Haemorrhage
☐ (7) Postpartum Hemorrhage
☐ (8) Other, specify____

9. Estimated blood loss____________________. Not done ☐ (99999)

10. Placenta weight______________________grams. Not done ☐ (99999)
11. Placental sample in RNA later done? (0) No (1) Yes.
    If No, why? ________________________
12. Placental sample in Buffered formalin done? (0) No (1) Yes

    If No, why?________________________(There is a Placental Histology CRF that needs to be completed once tissues processed and read)

13. Did the mother receive a blood transfusion?

☐ (1) Yes
☐ (0) No

If yes, how many pints? ___________________

14. If an abortion or stillbirth, was Gross visual inspection of aborted fetus/still birth done?

☐ (0) Not done
☐ (1) Done, no observed abnormalities
☐ (2) Done, observed abnormalities

If observed abnormalities, specify:

____
____
____
____

**STUDY ID**										
**Date**			-				-			
**Visit**	7	**DELIVERY**								

15. **Date of Discharge**: ☐☐/☐☐/☐☐☐☐

If prolonged admission, give reason

____

____

STAFF ID ☐☐☐☐

STUDY ID ☐☐☐☐☐☐☐☐☐☐

Date ☐☐-☐☐☐-☐☐☐☐

Visit | 7 | **DELIVERY** |

Neonatal outcome at birth

Date of birth: ☐☐/☐☐/☐☐☐ Time of Birth: ☐☐:☐☐

Sex: ☐ (1) Male ☐ (2) Female

1. Birth Length:________cm
2. Birth Length:________cm
3. Birth Length:________cm

1. Birth Weight:__________g
2. Birth Weight:__________g
3. Birth Weight:__________g

1. Head Circumference:_________cm
2. Head Circumference:_________cm
3. Head Circumference:_________cm

Apgar Score______1 min_____ 5min____________10min_________________

Did the child cry immediately after delivery? ☐ (1) Yes ☐ (0) No

If no immediate cry, what were the resuscitation measures?
☐ (1) Stimulation
☐ (2) Suction
☐ (3) Bag and Mask Ventilation, if this how long?_________________________mins
☐ (4) CPR, if this how long,_____________________________________mins
☐ (5) Oxygen therapy

Did the child suckle shortly after birth? ☐ (1) Yes ☐ (0) No

Congenital malformation / anomaly: ☐ (1) Yes ☐ (0) No

If yes specify in the box below:

Was the neonate admitted into nursery? (1) Yes (0) No

**STUDY ID**	☐☐☐☐☐☐☐☐☐☐
**Date**	☐☐-☐☐☐-☐☐☐☐
**Visit**	7 **DELIVERY**

If yes, what was the primary reason for admission?

Date of Admission: ☐☐/☐☐/☐☐☐☐ Time of Admission: ☐☐:☐☐

Date of Discharge: ☐☐/☐☐/☐☐☐☐ Time of Discharge: ☐☐:☐☐

Did the neonate have to be taken to a local clinic/ outpatient hospital, including emergency room (without having to be admitted) to attend any complications at birth – during the first 24 hours?

☐ (1) Yes
☐ (0) No

If Yes; please provide the number of times and the reason for the visit in the comment section:

Date of Visit: ☐☐/☐☐/☐☐☐☐ **Time of Visit:** ☐☐:☐☐

STAFF ID ☐☐☐☐

STUDY ID ☐☐☐☐☐☐☐☐☐☐

Date ☐☐-☐☐☐-☐☐☐☐

Visit ☐☐

Infant Physical Examination

INFANT VITAL SIGNS

☐ Not done

	Temperature	☐☐.☐ degrees Celsius
	Respiratory rate	☐☐☐ breaths per min
	Heart rate	☐☐☐ beats per min

INFANT ANTHROPOMETRY

☐ (1) Not done

	Weight	☐☐☐☐ gm<br>☐☐☐☐ gm<br>☐☐☐☐ gm
	Height	☐☐☐.☐☐ cm<br>☐☐☐.☐☐ cm<br>☐☐☐.☐☐ cm
	Head Circumference	☐☐☐.☐☐ cm<br>☐☐☐.☐☐ cm<br>☐☐☐.☐☐ cm
	MUAC (Mid upper arm circumference)	☐☐☐.☐☐ cm<br>☐☐☐.☐☐ cm<br>☐☐☐.☐☐ cm

STUDY ID | | | | | | | | | | |

Date | | | - | | | - | | | | |

Visit | | |

INFANT PHYSICAL EXAMINATION

☐ Not done

	Head	☐ (1) Normal<br>☐ (2) Abnormal<br>☐ (3) Not done	If abnormal, state any anomalies noted
	Eyes	☐ (1) Normal<br>☐ (2) Abnormal<br>☐ (3) Not done	
	Ears<br><br><br><br>Nose	☐ (1) Normal<br>☐ (2) Abnormal<br>☐ (3) Not done<br><br>(1) Normal<br>(2) Abnormal<br>(3) Not done	
	Mouth	☐ (1) Normal<br>☐ (2) Abnormal<br>☐ (3) Not done	
	Neck	☐ (1) Normal<br>☐ (2) Abnormal<br>☐ (3) Not done	
	Chest	☐ (1) Normal<br>☐ (2) Abnormal<br>☐ (3) Not done	
	Abdomen	☐ (1) Normal<br>☐ (2) Abnormal<br>☐ (3) Not done	
	Back	☐ (1) Normal<br>☐ (2) Abnormal<br>☐ (3) Not done	
	Genital area	☐ (1) Normal<br>☐ (2) Abnormal<br>☐ (3) Not done	
	Extremities	☐ (1) Normal<br>☐ (2) Abnormal<br>☐ (3) Not done	

STUDY ID

Date

		-				-				

Visit

STAFF ID

STUDY ID:

Date:

		–				–				

Visit:

EDINBURGH POSTNATAL DEPRESSION SCALE (EPDS) English VERSION

(administered at baseline and 9)

Please **UNDERLINE** the answer which comes closest to how you have felt **IN THE PAST 7 DAYS**, not just how you feel today.<br>Here is an example, already completed.		
Eg.	I have felt happy:	☐ (0) No, not at all<br>☐ (1) No, not very often<br>☐ (2) Yes, most of the time *<br>☐ (3) Yes, all the time<br><br>*This would mean: ***"I have felt happy most of the time" during the past week***. Please complete the other questions in the same way.
In the past 7 days:		
01.	In the past 7 days: I have been able to laugh and see the funny side of things	☐ (0) As much as I always could<br>☐ (1) Not quite so much now<br>☐ (2) Definitely not so much now<br>☐ (3) Not at all
02.	In the past 7 days: I have looked forward with enjoyment to things	☐ (0) As much as I ever did<br>☐ (1) Rather less than I used to<br>☐ (2) Definitely less than I used to<br>☐ (3) Hardly at all
03.	In the past 7 days: I have blamed myself unnecessarily when things went wrong	☐ (3) Yes, most of the time<br>☐ (2) Yes, some of the time<br>☐ (1) Not very often<br>☐ (0) No, never
04.	In the past 7 days: I have been anxious or worried for no good reason	☐ (0) No, not at all<br>☐ (1) Hardly ever<br>☐ (2) Yes, sometimes<br>☐ (3) Yes, very often
05.	In the past 7 days: I have felt scared or panicky for no very good reason	☐ (3) Yes, quite a lot<br>☐ (2) Yes, sometimes<br>☐ (1) No, not much<br>☐ (0) No, not at all

STUDY ID: | | | | | | | | | | |

Date: | | | – | | | | – | | | | |

Visit: | | |

06.	In the past 7 days: Things have been getting on top of me	☐ (3) Yes, most of the time I haven't been able to cope at all<br>☐ (2) Yes, sometimes I haven't been coping as well as usual<br>☐ (1) No, most of the time I have coped quite well<br>☐ (0) No, have been coping as well as ever
07.	In the past 7 days: I have been so unhappy that I have had difficulty sleeping	☐ (3) Yes, most of the time<br>☐ (2) Yes, sometimes<br>☐ (1) Not very often<br>☐ (0) No, not at all
08.	In the past 7 days: I have felt sad or miserable	☐ (3) Yes, most of the time<br>☐ (2) Yes, quite often<br>☐ (1) Not very often<br>☐ (0) No, not at all
09.	In the past 7 days: I have been so unhappy that I have been crying	☐ (3) Yes, most of the time<br>☐ (2) Yes, quite often<br>☐ (1) Only occasionally<br>☐ (0) No, never
10.	In the past 7 days: The thought of harming myself has occurred to me	☐ (3) Yes, quite often<br>☐ (2) Sometimes<br>☐ (1) Hardly ever<br>☐ (0) Never

STAFF ID: ______________________________ ☐☐☐☐

STUDY ID: | | | | | | | | | | |

Date: | | | – | | | | – | | | |

Visit: | | |

EDINBURGH POSTNATAL DEPRESSION SCALE (EPDS) English VERSION

(administered at baseline and 9)

Please **UNDERLINE** the answer which comes closest to how you have felt **IN THE PAST 7 DAYS**, not just how you feel today.<br>Here is an example, already completed.		
Eg.	I have felt happy:	☐ (0) No, not at all<br>☐ (1) No, not very often<br>☐ (2) Yes, most of the time *<br>☐ (3) Yes, all the time<br><br>*This would mean: ***"I have felt happy most of the time" during the past week***. Please complete the other questions in the same way.
In the past 7 days:		
01.	In the past 7 days: I have been able to laugh and see the funny side of things	☐ (0) As much as I always could<br>☐ (1) Not quite so much now<br>☐ (2) Definitely not so much now<br>☐ (3) Not at all
02.	In the past 7 days: I have looked forward with enjoyment to things	☐ (0) As much as I ever did<br>☐ (1) Rather less than I used to<br>☐ (2) Definitely less than I used to<br>☐ (3) Hardly at all
03.	In the past 7 days: I have blamed myself unnecessarily when things went wrong	☐ (3) Yes, most of the time<br>☐ (2) Yes, some of the time<br>☐ (1) Not very often<br>☐ (0) No, never
04.	In the past 7 days: I have been anxious or worried for no good reason	☐ (0) No, not at all<br>☐ (1) Hardly ever<br>☐ (2) Yes, sometimes<br>☐ (3) Yes, very often
05.	In the past 7 days: I have felt scared or panicky for no very good reason	☐ (3) Yes, quite a lot<br>☐ (2) Yes, sometimes<br>☐ (1) No, not much<br>☐ (0) No, not at all

STUDY ID: | | | | | | | | | | |

Date: | | | – | | | | – | | | | |

Visit: | | |

06.	In the past 7 days: Things have been getting on top of me	☐ (3) Yes, most of the time I haven't been able to cope at all<br>☐ (2) Yes, sometimes I haven't been coping as well as usual<br>☐ (1) No, most of the time I have coped quite well<br>☐ (0) No, have been coping as well as ever
07.	In the past 7 days: I have been so unhappy that I have had difficulty sleeping	☐ (3) Yes, most of the time<br>☐ (2) Yes, sometimes<br>☐ (1) Not very often<br>☐ (0) No, not at all
08.	In the past 7 days: I have felt sad or miserable	☐ (3) Yes, most of the time<br>☐ (2) Yes, quite often<br>☐ (1) Not very often<br>☐ (0) No, not at all
09.	In the past 7 days: I have been so unhappy that I have been crying	☐ (3) Yes, most of the time<br>☐ (2) Yes, quite often<br>☐ (1) Only occasionally<br>☐ (0) No, never
10.	In the past 7 days: The thought of harming myself has occurred to me	☐ (3) Yes, quite often<br>☐ (2) Sometimes<br>☐ (1) Hardly ever<br>☐ (0) Never

STAFF ID: ______________________________ ☐☐☐☐

24.1.14: MIBS Form

MOTHER-TO-INFANT BONDING SCALE (administered at visit 9, 10 and 12)

Malangizo:

Ndikufuna ndidziwe m'mene mwakhala mukumvera zokhudzana ndi mwana wanu cha posachedwapa. Munsimu muli muli zina zimene amai amamva zokhudzana ndi ana awo. Chonde lembani mzere pansi pa yankho limene lukugwirizanako ndi m'mene mukumvera zokhudzana ndi mwana wanu, osangoti m'mene mukumvera pa tsiku la lero. Chonde malidzitsani mfundo ZONSE.

Mwachitsanzo

bh	Ndimanva kukoma kumuchitira zinthu mwana wanga	(0) Ayi, ndi pang'ono pomwe<br>***(1) <u>Pang'ono kwambiri nthawi zina</u>***<br>(2) Kwambiri, nthawi zina<br>(0) Kwambiri, nthawi zambiri<br>Mau awa akutanthauza kuti: "Ndimamva kukoma pang'ono kwambiri nthawi zina kumuchitira zinthu mwana wanga".
01.	Ndimamva kumukonda mwana wanga	(3) Kwambiri kwake, nthawi zambiri<br>(2) Kambirir kwake, nthawi zina<br>(1) Pang'ono, nthawi zina<br>(0) Ayi ndi pang'ono pomwe
02.	Ndimaopa kapena ndimapanikizika ndikachita china chake kwa mwana wanga	(0) Ayi nkomwe<br>(1) Pang'ono, nthawi zina<br>(2) Kwambiri kwake, nthawi ina<br>(3) Kwambiri wake, nthawi zambiriri
03.	Ndimakhala osakondwa ndi mwana wanga	(3) Kwambiri kwake, nthawi zambiri<br>(2) Kwambiriko kwake, nthawi zina<br>(1) Pang'ono, nthawi zina<br>(0) Ayi ndi pang'ono pomwe
04.	Palibe chimene ndimanva cha mwana wanga	(3) Kwambiri kwake, nthawi zambiri<br>(2) Kwambiri kwake, nthawi zina<br>(1) Pang'ono nthawi zina

		☐ (0) Ayi ndi pang'ono pomwe
05.	Ndimakwiya ndi mwana wanga	☐ (3) Kwambiri kwake, nthawi zambiri<br>☐ (2) Kwambiri kwake, nthawi zina<br>☐ (1) Panag'ono, nthawi zina<br>☐ (0) Ayi ndi pang'ono pomwe
06.	Ndimamva kukoma kumuchitira zinthu mwana wanga	☐ (3) Ayi, ndi pang'ono pomwe<br>☐ (2) Kwambiri kwake, nthawi zina<br>☐ (1) Pang'ono kwambiri nthawi zina<br>☐ (0) Kwambiri kwake, nthawi zambiri
07.	Ndimafuna mwana wanga akanakhala wamtundu wina	☐ (3) Kwambiri kwake, nthawi zambiri<br>☐ (2) Kwambiri kwake, nthawi zina<br>☐ (1) Png'ono, nthawi zina<br>☐ (0) Ayi ndi pang'ono pomwe
08.	Ndimachimva kuti ndimamuteteza mwana wanga	☐ (0) Ayi ndi pang'ono pomwe<br>☐ (1) Pang'ono, nthawi zina<br>☐ (1) Kwambiri kwake, nthawi zina<br>☐ (0) Kwambiri kwake, nthawi zambiri
09.	Ndimalakalaka ndikanakhala opanda mwana	☐ (3) Kwambiri kwake, nthawi zambiri<br>☐ (2) Kwambiri kwake, nthawi zina<br>☐ (1) Pang'ono, nthawi zina<br>☐ (0) Ayi ndi pang'ono pomwe
10.	Ndimamva kuti ndimakhala pafupi ndi mwana wanga	☐ (0) Ayi ndi pang'ono pomwe<br>☐ (1) Pang'ono, nthawi zina<br>☐ (2) Kwambiri kwake, nthawi zina<br>☐ (3) Kwambiri kwake, nthawi zambiri

STAFF ID: ☐☐☐☐

**SCREENING ID**	☐☐☐☐☐☐☐☐☐☐
**Date**	☐☐-☐☐☐-☐☐☐☐
**Visit**	☐

Vaccination history

Has the infant received the following vaccines? (Use documented evidence)

**Visit**	**Vaccine received at**	**Vaccine**	
☐ **Visit 8** (1 month post-partum)	Birth	BCG	☐ (0) No (If No reason)<br>☐ (1) Yes (If Yes date received)<br>☐ (99) Documentation not available
		OPV (Polio 0)	☐ (0) No (If No reason)<br>☐ (1) Yes (If Yes date received)<br>☐ (99) Documentation not available
☐ **Visit 9 (3 months post-partum)**	6 weeks	OPV 1	☐ (0) No (If No reason)<br>☐ (1) Yes (If Yes date received)<br>☐ (99) Documentation not available
		DPT-HepB-Hib1	☐ (0) No (If No reason)<br>☐ (1) Yes (If Yes date received)<br>☐ (99) Documentation not available
		PVC 1	☐ (0) No (If No reason)<br>☐ (1) Yes (If Yes date received)<br>☐ (99) Documentation not available
		Rotavirus vaccine 1	☐ (0) No (If No reason)<br>☐ (1) Yes (If Yes date received)<br>☐ (99) Documentation not available
	10 weeks	OPV 2	☐ (0) No (If No reason)<br>☐ (1) Yes (If Yes date received)<br>☐ (99) Documentation not available
		DPT-HepB-Hib2	☐ (0) No (If No reason)<br>☐ (1) Yes (If Yes date received)<br>☐ (99) Documentation not available
		PCV 2	☐ (0) No (If No reason)<br>☐ (1) Yes (If Yes date received)<br>☐ (99) Documentation not available
		Rotavirus vaccine 2	☐ (0) No (If No reason)<br>☐ (1) Yes (If Yes date received)<br>☐ (99) Documentation not available
☐ **Visit 10 (6 months post-partum)**	14 weeks	OPV 3	☐ (0) No (If No reason)<br>☐ (1) Yes (If Yes date received)<br>☐ (99) Documentation not available
		DPT-HepB-Hib3	☐ (0) No (If No reason)

SCREENING ID ☐☐☐☐☐☐☐☐☐☐

Date ☐☐ - ☐☐☐ - ☐☐☐☐

Visit ☐ ☐

			☐ (1) Yes (If Yes date received)<br>☐ (99) Documentation not available
		PCV 3	☐ (0) No (If No reason)<br>☐ (1) Yes (If Yes date received)<br>☐ (99) Documentation not available
☐ **Visit 11 (9 months post-partum)**	6 months	Vitamin A	☐ (0) No (If No reason)<br>☐ (1) Yes (If Yes date received)<br>☐ (99) Documentation not available
☐ **Visit 12 (12 months post-partum)**	9 months	Measles 1	☐ (0) No (If No reason)<br>☐ (1) Yes (If Yes date received)<br>☐ (99) Documentation not available

STAFF INITIALS ____________________

PARTICIPANT ID | | | | | | | | | | | |

DATE | | | - | | | - | | | | |

VISIT | | |

1.	Did your child fall sick/ill since our last meeting?	☐ (1) Yes<br>☐ (0) No<br><br>If yes how many times was the child sick?<br>(1) ☐ (2) ☐ (3) ☐ (4) ☐ (>4) ☐
2.	Did your child suffer from fever in the past month?	☐ (1) Yes<br>☐ (0) No<br><br>If yes how many times?<br>(1) ☐ (2) ☐ (3) ☐ (4) ☐ (>4) ☐<br><br>Was a malaria test done?<br>☐ (1) Yes<br>☐ (0) No<br><br>(if yes, indicate outcome i.e. whether positive or negative).<br>☐ (1) Yes<br>☐ (0) No
3.	Did your child suffer from diarrhoea (passage of three or more loose or liquid stools per day) in the past month?	☐ (1) Yes<br>☐ (0) No<br><br>If yes how many times did you child have diarrhea?<br>(1) ☐ (2) ☐ (3) ☐ (4) ☐ (>4) ☐
4.	Did your child suffer from vomiting in the past month?	☐ (1) Yes<br>☐ (0) No<br><br>If yes how many times?<br>(1) ☐ (2) ☐ (3) ☐ (4) ☐ (>4) ☐
5.	Did your child suffer from a cough in the past month?	☐ (1) Yes<br>☐ (0) No<br><br>If yes how many times?<br>(1) ☐ (2) ☐ (3) ☐ (4) ☐ (>4) ☐<br><br>Were there signs of “fast breathing, shortness of breath wheezing, or chest in-drawing”?

**PARTICIPANT ID**											

**DATE**			-				-				

**VISIT**		

		☐ (1) Yes<br>☐ (0) No
6.	Did your child have any other symptoms?	☐ (1) Yes (**specify**)<br>☐ (0) No<br><br>If yes how many times?<br>(1) ☐ (2) ☐ (3) ☐ (4) ☐ (>4) ☐
7.	Did you seek treatment on any of the occasions?	☐ (1) Yes<br>☐ (0) No<br><br>Where was treatment sought?<br>☐ (1) Hospital<br>☐ (2) Health Centre<br>☐ (3) Private clinic<br>☐ (4) Drug store/pharmacy<br>☐ (5) Traditional healer/Herbalist<br>☐ (6) Other (**Specify**)
8.	Feeding habits	Tick all that applies<br>☐ (1) Breastfeeding<br>☐ (2) Bottle feeding<br>☐ (3) semi solid food (phala)<br>☐ (4) Normal family meals

STAFF INITIALS______________________________

**Participant ID**	☐☐☐☐☐☐☐☐☐☐
**Date**	☐☐☐☐☐☐☐☐☐
**Visit**	☐ ☐

Age: Gravidity: Parity:

Adverse events

Note: A clinical condition which is diagnosed prior to enrolment (at baseline) is a pre-existing condition and should be documented as part of a participant's medical history and not as an adverse event.

Please record all adverse events observed or reported regardless of suspected treatment causality.

DESCRIPTION OF ADVERSE EVENT

	Description of adverse event	
	Time of onset	☐☐ :☐☐<br>Hrs Mins

(Allow room to record more than one adverse event and time of onset of each)

SEVERITY (Check 1 only)

	☐ (1) Grade 1<br>☐ (2) Grade 2<br>☐ (3) Grade 3<br>☐ (4) Grade 4<br>☐ (5) Grade 5
	Do serious criteria apply? ☐ (1) Yes, **If Yes, Notify PI immediately**<br>☐ (2) No
	If yes please specify:<br>☐ Death<br>☐ life-threatening<br>☐ In-patient hospitalization, prolongation of existing hospitalization<br>☐ Persistent or significant disability/incapacity<br>☐ Congenital anomaly/birth defect<br>☐ Important medical event (i.e. may jeopardize participant and may require medical/surgical interventions to prevent above outcomes)

**Participant ID**										
**Date**										
**Visit**										

ACTION (check all relevant actions)

**Study treatment arm: Control (Oral Ferrous Sulphate)**<br><br>**If IV arm, event occurred:** ☐ During drug administration ☐ After drug administration<br><br>**Action taken on study treatment:** (Tick one only and enter details in study treatment section)		
	☐ (0) Continued<br>☐ (1) Increased<br>☐ (2) Reduced<br>☐ (3) Stopped temporarily<br>☐ (4) Permanently discontinued	
**Participant:** (Tick all relevant options and enter details in concomitant treatment section if applicable		
	☐ (1) Withdrawn from study<br>☐ (2) Treatment given (specify details in concomitant treatment section)<br>☐ (3) Other Specify ___________________________________<br>☐ (4) No action	

OUTCOME OF AE

	Still present/ongoing?	☐ (1) Yes<br>☐ (0) No, resolved
	Date resolved:<br>☐☐ / ☐☐☐ / ☐☐☐☐<br>dd mmm yyyy	Time resolved: (24-hour clock)<br>☐☐:☐☐<br>Hrs : min
Complete the following section when causality is determined, when the event resolves, or at the end of the study, whichever comes first		

Is the participant taking any concomitant Medication? (List all medications taken up to 1 month prior to event) ☐ (1) Yes ☐ (0) No

Medication	Start Date DD/MMM/YYYY	Stop Date DD/MMM/YYYY	Total Daily Dose	Indication	Suspect
					☐ Yes<br>☐ No

Participant ID

Date

Visit

					ϒ Yes<br>ϒ No

List Relevant Lab/Diagnostic results below OR attach copies of the results.
Relevant Laboratory Tests ! No relevant laboratory tests done

**Test**	**Collection Date** (DD/MMM/YYYY)	**Result**	**Site Normal Range**	**Collection Date of test previous to this SAE**	**Result of test previous to this SAE**

Relevant Diagnostic Tests (EX: MRI, CT Scan, Ultrasound) No relevant diagnostic tests

**Test**	**Date Performed** (DD/MMM/YYYY)	**Results/Comments**

Treatment or medication given

**Medication**	**Start Date DD/MMM/YYYY**	**Stop Date DD/MMM/YYYY**	**Total Daily Dose**	**Indication**

Participant ID

Date

Visit

Did participant require the following?

☐ Blood transfusion

☐ ICU admission

Any other non-medical or surgical treatment given? ☐ Yes ☐ No
If Yes, specify: ________________________________

CAUSALITY

	Is there a reasonable possibility the adverse event is related to the study drug?	☐ (1) Yes<br>☐ (0) No<br>☐ (2) Unknown
	If No, what was the most likely cause of the AE? (Tick ONE only)	☐ (1) Disease under study<br>☐ (2) Other illness, specify<br>☐ (3) Concomitant treatment (drug or non-drug, specify)<br>☐ (4) Other, Specify

STAFF INITIALS: ________________________________

**Participant ID**										
**Date**										
**Visit**										

Age: Gravidity: Parity:

Adverse events

Note: A clinical condition which is diagnosed prior to enrolment (at baseline) is a pre-existing condition and should be documented as part of a participant's medical history and not as an adverse event.

Please record all adverse events observed or reported regardless of suspected treatment causality.

DESCRIPTION OF ADVERSE EVENT

	Description of adverse event	
	Time of onset	☐☐ :☐☐<br>Hrs Mins

(Allow room to record more than one adverse event and time of onset of each)

SEVERITY (Check 1 only)

	☐ (1) Grade 1<br>☐ (2) Grade 2<br>☐ (3) Grade 3<br>☐ (4) Grade 4<br>☐ (5) Grade 5
	Do serious criteria apply? ☐ (1) Yes, **If Yes, Notify PI immediately**<br>☐ (2) No
	If yes please specify:<br>☐ Death<br>☐ life-threatening<br>☐ In-patient hospitalization, prolongation of existing hospitalization<br>☐ Persistent or significant disability/incapacity<br>☐ Congenital anomaly/birth defect<br>☐ Important medical event (i.e. may jeopardize participant and may require medical/surgical interventions to prevent above outcomes)

Participant ID ☐☐☐☐☐☐☐☐☐☐

Date ☐☐☐☐☐☐☐☐☐

Visit ☐ ☐

ACTION (check all relevant actions)

**Study treatment arm: Control (Oral Ferrous Sulphate)**<br><br>**If IV arm, event occurred:** ☐ During drug administration ☐ After drug administration<br><br>**Action taken on study treatment:** (Tick one only and enter details in study treatment section)	
	☐ (0) Continued<br>☐ (1) Increased<br>☐ (2) Reduced<br>☐ (3) Stopped temporarily<br>☐ (4) Permanently discontinued
**Participant:** (Tick all relevant options and enter details in concomitant treatment section if applicable	
	☐ (1) Withdrawn from study<br>☐ (2) Treatment given (specify details in concomitant treatment section)<br>☐ (3) Other Specify ___________________________________<br>☐ (4) No action

OUTCOME OF AE

	Still present/ongoing?	☐ (1) Yes<br>☐ (0) No, resolved
	Date resolved:<br>☐☐ / ☐☐☐ / ☐☐☐☐<br>dd mmm yyyy	Time resolved: (24-hour clock)<br>☐☐:☐☐<br>Hrs : min
Complete the following section when causality is determined, when the event resolves, or at the end of the study, whichever comes first		

Is the participant taking any concomitant Medication? (List all medications taken up to 1 month prior to event) ☐ (1) Yes ☐ (0) No

Medication	Start Date DD/MMM/YYYY	Stop Date DD/MMM/YYYY	Total Daily Dose	Indication	Suspect
					☐ Yes<br>☐ No

**Participant ID**										

**Date**									

**Visit**		

					ϒ Yes<br>ϒ No

List Relevant Lab/Diagnostic results below OR attach copies of the results.
Relevant Laboratory Tests ! No relevant laboratory tests done

**Test**	**Collection Date** (DD/MMM/YYYY)	**Result**	**Site Normal Range**	**Collection Date of test previous to this SAE**	**Result of test previous to this SAE**

Relevant Diagnostic Tests (EX: MRI, CT Scan, Ultrasound) No relevant diagnostic tests

**Test**	**Date Performed** (DD/MMM/YYYY)	**Results/Comments**

Treatment or medication given

**Medication**	**Start Date DD/MMM/YYYY**	**Stop Date DD/MMM/YYYY**	**Total Daily Dose**	**Indication**

Participant ID | | | | | | | | | | |

Date | | | | | | | | | |

Visit | | |

Did participant require the following?

☐ Blood transfusion

☐ ICU admission

Any other non-medical or surgical treatment given? ☐ Yes ☐ No
If Yes, specify: ________________________________

CAUSALITY

	Is there a reasonable possibility the adverse event is related to the study drug?	☐ (1) Yes<br>☐ (0) No<br>☐ (2) Unknown
	If No, what was the most likely cause of the AE? (Tick ONE only)	☐ (1) Disease under study<br>☐ (2) Other illness, specify<br>☐ (3) Concomitant treatment (drug or non-drug, specify)<br>☐ (4) Other, Specify

STAFF INITIALS: ________________________________

**Participant ID**										
**Date**										
**Visit**										

Age: Gravidity: Parity:

Adverse events

Note: A clinical condition which is diagnosed prior to enrolment (at baseline) is a pre-existing condition and should be documented as part of a participant's medical history and not as an adverse event.

Please record all adverse events observed or reported regardless of suspected treatment causality.

DESCRIPTION OF ADVERSE EVENT

	Description of adverse event	
	Time of onset	☐☐ :☐☐<br>Hrs Mins

(Allow room to record more than one adverse event and time of onset of each)

SEVERITY (Check 1 only)

	☐ (1) Grade 1<br>☐ (2) Grade 2<br>☐ (3) Grade 3<br>☐ (4) Grade 4<br>☐ (5) Grade 5
	Do serious criteria apply? ☐ (1) Yes, **If Yes, Notify PI immediately**<br>☐ (2) No
	If yes please specify:<br>☐ Death<br>☐ life-threatening<br>☐ In-patient hospitalization, prolongation of existing hospitalization<br>☐ Persistent or significant disability/incapacity<br>☐ Congenital anomaly/birth defect<br>☐ Important medical event (i.e. may jeopardize participant and may require medical/surgical interventions to prevent above outcomes)

**Participant ID**	☐☐☐☐☐☐☐☐☐☐
**Date**	☐☐☐☐☐☐☐☐☐
**Visit**	☐

ACTION (check all relevant actions)

**Study treatment arm: Control (Oral Ferrous Sulphate)**<br><br>**If IV arm, event occurred:** ☐ During drug administration ☐ After drug administration<br><br>**Action taken on study treatment:** (Tick one only and enter details in study treatment section)	
	☐ (0) Continued<br>☐ (1) Increased<br>☐ (2) Reduced<br>☐ (3) Stopped temporarily<br>☐ (4) Permanently discontinued
**Participant:** (Tick all relevant options and enter details in concomitant treatment section if applicable	
	☐ (1) Withdrawn from study<br>☐ (2) Treatment given (specify details in concomitant treatment section)<br>☐ (3) Other Specify ___________________________________<br>☐ (4) No action

OUTCOME OF AE

	Still present/ongoing?	☐ (1) Yes<br>☐ (0) No, resolved
	Date resolved:<br>☐☐ / ☐☐☐/ ☐☐☐☐<br>dd mmm yyyy	Time resolved: (24-hour clock)<br>☐☐:☐☐<br>Hrs : min
Complete the following section when causality is determined, when the event resolves, or at the end of the study, whichever comes first		

Is the participant taking any concomitant Medication? (List all medications taken up to 1 month prior to event) ☐ (1) Yes ☐ (0) No

Medication	Start Date DD/MMM/YYYY	Stop Date DD/MMM/YYYY	Total Daily Dose	Indication	Suspect
					☐ Yes<br>☐ No

Participant ID | | | | | | | | | | |

Date | | | | | | | | | |

Visit | | |

					ϒ Yes<br>ϒ No

List Relevant Lab/Diagnostic results below OR attach copies of the results.
Relevant Laboratory Tests ! No relevant laboratory tests done

**Test**	**Collection Date** (DD/MMM/YYYY)	**Result**	**Site Normal Range**	**Collection Date of test previous to this SAE**	**Result of test previous to this SAE**

Relevant Diagnostic Tests (EX: MRI, CT Scan, Ultrasound) No relevant diagnostic tests

**Test**	**Date Performed** (DD/MMM/YYYY)	**Results/Comments**

Treatment or medication given

**Medication**	**Start Date DD/MMM/YYYY**	**Stop Date DD/MMM/YYYY**	**Total Daily Dose**	**Indication**

Participant ID ☐☐☐☐☐☐☐☐☐☐

Date ☐☐☐☐☐☐☐☐☐

Visit ☐ ☐

Did participant require the following?

☐ Blood transfusion

☐ ICU admission

Any other non-medical or surgical treatment given? ☐ Yes ☐ No
If Yes, specify: ________________________________

CAUSALITY

	Is there a reasonable possibility the adverse event is related to the study drug?	☐ (1) Yes<br>☐ (0) No<br>☐ (2) Unknown
	If No, what was the most likely cause of the AE? (Tick ONE only)	☐ (1) Disease under study<br>☐ (2) Other illness, specify<br>☐ (3) Concomitant treatment (drug or non-drug, specify)<br>☐ (4) Other, Specify

STAFF INITIALS: ________________________________

PARTICIPANT ID ☐☐☐☐☐☐☐☐☐

Date ☐☐-☐☐☐-☐☐☐☐

Visit ☐☐

END OF STUDY VISIT

Visit number: ☐☐☐ Visit Date: ☐☐/☐☐/☐☐☐☐

A. STUDY OUTCOME	
1.	Outcome of participant in this study:<br>☐ (1) Completed Study<br>☐ (2) Withdrawn consent<br>☐ (3) Withdrawn for safety reason<br>☐ (4) Lost to follow up<br>☐ (5) Death (maternal)<br>☐ (6) Death (infant)<br>☐ (7) Miscarriage<br>☐ (8) Did not reconsent
2.	Date of last visit: ☐☐/☐☐/☐☐☐☐
3.	Last contact visit:<br>☐ (1) Enrolment (visit 1)<br>☐ (2) Pre-delivery visit: 36 weeks GA (visit 4)<br>☐ (3) Delivery (visit 7)<br>☐ (4) 1 month postpartum (Visit 8)<br>☐ (5) 3 months postpartum (Visit 9)<br>☐ (6) 6 months postpartum (Visit 10)<br>☐ (6) 9 months postpartum (Visit 11)<br>☐ (8) 12 months postpartum (Visit 12)
4	Which scheduled visits did the participant attend? (reason if missed)<br>☐ (1) Enrolment (visit 1)<br>☐ (2) Pre-delivery visit: 36 weeks GA (visit 4). Missed reason____________<br>☐ (3) Delivery (visit 7) Missed reason____________<br>☐ (4) 1 month postpartum (Visit 8) Missed reason____________<br>☐ (5) 3 months postpartum (Visit 9) Missed reason____________<br>☐ (6) 6 months postpartum (Visit 10) Missed reason____________

PARTICIPANT ID | | | | | | | | | |

Date | | | - | | | | - | | | | |

Visit | | |

	☐ (7) 9 months postpartum (Visit 11) Missed reason____________<br>☐ (8) 12 months postpartum (Visit 12) Missed reason____________
**B. END OF STUDY COMPLETION INFORMATION**	
Completed by: ☐☐☐ Signature: ………………….. Date: ☐☐/☐☐/☐☐☐☐<br>Verified by: ☐☐☐ Signature: ………………….. Date: ☐☐/☐☐/☐☐☐☐	

STAFF ID ☐☐☐☐

PARTICIPANT ID ☐☐☐☐☐☐☐☐☐

Date ☐☐-☐☐☐-☐☐☐☐

Visit ☐☐

END OF STUDY VISIT

Visit number: ☐☐☐ Visit Date: ☐☐/☐☐/☐☐☐☐

A. STUDY OUTCOME	
1.	Outcome of participant in this study:<br>☐ (1) Completed Study<br>☐ (2) Withdrawn consent<br>☐ (3) Withdrawn for safety reason<br>☐ (4) Lost to follow up<br>☐ (5) Death (maternal)<br>☐ (6) Death (infant)<br>☐ (7) Miscarriage<br>☐ (8) Did not reconsent
2.	Date of last visit: ☐☐/☐☐/☐☐☐☐
3.	Last contact visit:<br>☐ (1) Enrolment (visit 1)<br>☐ (2) Pre-delivery visit: 36 weeks GA (visit 4)<br>☐ (3) Delivery (visit 7)<br>☐ (4) 1 month postpartum (Visit 8)<br>☐ (5) 3 months postpartum (Visit 9)<br>☐ (6) 6 months postpartum (Visit 10)<br>☐ (6) 9 months postpartum (Visit 11)<br>☐ (8) 12 months postpartum (Visit 12)
4	Which scheduled visits did the participant attend? (reason if missed)<br>☐ (1) Enrolment (visit 1)<br>☐ (2) Pre-delivery visit: 36 weeks GA (visit 4). Missed reason____________<br>☐ (3) Delivery (visit 7) Missed reason____________<br>☐ (4) 1 month postpartum (Visit 8) Missed reason____________<br>☐ (5) 3 months postpartum (Visit 9) Missed reason____________<br>☐ (6) 6 months postpartum (Visit 10) Missed reason____________

PARTICIPANT ID | | | | | | | | |

Date | | - | | | | - | | | |

Visit | |

	☐ (7) 9 months postpartum (Visit 11) Missed reason____________<br>☐ (8) 12 months postpartum (Visit 12) Missed reason____________
**B. END OF STUDY COMPLETION INFORMATION**	
Completed by: ☐☐☐ Signature: ………………….. Date: ☐☐/☐☐/☐☐☐☐<br>Verified by: ☐☐☐ Signature: ………………….. Date: ☐☐/☐☐/☐☐☐☐	

STAFF ID ☐☐☐☐

**SCREENING ID**	
**STUDY ID**	

ATTACHMENT: CONSENT COVER SHEET

Participant Name:	
Participants age at time of informed consent	
Name of husband or guardian present during consenting process (optional)	
Date of informed consent discussion:	
Date informed consent obtained	
Time (24 hours clock)	
Name of study staff/person completing informed consent discussion (and this coversheet):	
Is the potential volunteer literate?	☐ Yes<br>☐ No<br>If no, an impartial witness must be present during the entire informed consent discussion. Refer to informed consent SOP for specific instructions.
Was a copy of the informed consent form given to the participant?	☐ Yes<br>☐ No, refused to accept

**SCREENING ID**										
**STUDY ID**										

ATTACHMENT: CONSENT COVER SHEET

Notes/Comments: Document questions or concerns woman/man has about study participation.

STAFF ID: ________________

PARTICIPANT ID | | | | | | | | | |

Date | | | | | | | | | |

Visit | | |

PLACENTAL HISTOPATHOLOGY EVALUATION

**A. Evaluation method, Sample preservation and adequacy**<br>☐ **EVAL**<br>☐ **QC**		
01.	Evaluation. Type of light Microscopy used	☐ *(1) Conventional*<br>☐ *(2) Polarized*<br>☐ *(3) Both*
02.	Type of stain used	☐ *(1) H & E*<br>☐ *(2) Giemsa*<br>☐ *(3) Other* ***Specify*_________________**
03.	Formalin pigment	☐ *(0) Absent*<br>☐ *(1) Mild*<br>☐ *(2) Moderate*<br>☐ *(4) Abundant*
04.	Erythrocytes in intervillous space	☐ *(0) Absent*<br>☐ *(1) Mild*<br>☐ *(2) Moderate*<br>☐ *(4) Abundant*
05.	Decidual Basalis present	☐ *(0) No*<br>☐ *(1) Yes*
06.	Chorion present	☐ *(0) No*<br>☐ *(1) Yes*
07.	Amnios present	☐ (0) No<br>☐ (1) Yes

**B. Parasites/Malaria pigment**		
01.	Malaria Parasites present	☐ *(0) No*<br>☐ *(1) Yes*<br>***If No skip to Q3***

**PARTICIPANT ID**	
**Date**	
**Visit**	

02.	Percentage of parasitized Maternal erythrocytes	☐ *(1) <1%*<br>☐ *(2) 1-10%*<br>☐ *(3) >10%*
03.	Malaria pigment (excluding parasites)	☐ *(0) Absent*<br>☐ *(1) Mild (seen at High power)*<br>☐ *(2) Moderate (seen at medium power*<br>☐ *(4) Abundant (see at low power)*
04.	Malaria pigment in free macrophages	☐ *(0) No*<br>☐ *(1) Yes*
05.	Malaria Pigment within Fibrin	☐ *(0) No*<br>☐ *(1) Yes*
06.	Parasitized Fetal Erythrocytes or Pigment villi	☐ *(0) No*<br>☐ *(1) Yes*

**C. Other abnormalities**		
01.	Intervillous inflammation, Leukocytes	☐ *(1) <5*<br>☐ *(2) 6-10*<br>☐ *(3) 11-25*<br>☐ *(4) >25*
02.	Leucocyte Predominance	☐ *(1) Neutrophils*<br>☐ *(2) Monocytes and Macrophages*
03.	Chorionitis	☐ *(0) Absent*<br>☐ *(1) Present*<br>☐ *(3) Chorion not present*
04	Severity of chorionitis (if Present)	*(1) Grade I*<br>*(2) Grade II*<br>*(3) Grade III*

PARTICIPANT ID

Date

Visit

05	Amnionitis	*(0) Absent*<br>*(1) Present*<br>*(3) Amnion not present*
06.	Severity of Amnionitis	☐ *(1) Grade I*<br>☐ *(2) Grade II*<br>☐ *(3) Grade III*

D. Diagnosis/Comments

Case report completed by

Signature

PARTICIPANT ID

Date

Visit

Date

Household Food Insecurity Access Scale (HFIAS)

Instruction: Each of the questions in the following table will be asked with a recall period of four weeks (30 days). The respondent is first asked an occurrence question – that is, whether the condition in the question happened at all in the past four weeks (yes or no). If the respondent answers "yes" to an occurrence question, a frequency-of-occurrence question is asked to determine whether the condition happened rarely (once or twice), sometimes (three to ten times) or often (more than ten times) in the past four weeks.

Example:

1. In the past four weeks, did you worry that your household would not have enough food?

    0 = No (skip to Q2)

    1 = Yes

1.a. How often did this happen?

    1 = Rarely (once or twice in the past four weeks)

    2 = Sometimes (three to ten times in the past four weeks)

    3 = Often (more than ten times in the past four weeks)

No	Question	Response option	Code
1.<br><br>1.a	In the past four weeks, did you worry that your household would not have enough food?<br><br>How often did this happen in the past four weeks? (1=Rarely, 2 =Sometimes, 3 = Often)	0 = No (skip to<br>Q.8)1 = Yes	

2<br><br>2a	In the past four weeks, were you or any household member not able to eat the kinds of foods you preferred because of a lack of resources?<br><br>How often did this happen in the past four weeks? (1=Rarely, 2 =Sometimes, 3 = Often)	0 = No (skip to Q.8)<br><br>1 = Yes	
3<br><br>3a	In the past four weeks, did you or any household member have to eat a limited variety of foods due to a lack of resources?<br><br>How often did this happen in the past four weeks? (1=Rarely, 2 =Sometimes, 3 = Often)	0 = No (skip to Q.8)<br><br>1 = Yes	
4<br><br>4a	In the past four weeks, did you or any household member have to eat some foods that you really did not want to eat because of a lack of resources to obtain other types of food?<br><br>How often did this happen in the past four weeks? (1=Rarely, 2 =Sometimes, 3 = Often)	0 = No (skip to Q.8)<br><br>1 = Yes	
5<br><br>5a	In the past four weeks, did you or any household member have to eat a smaller meal than you felt you needed because there was not enough food?<br><br>How often did this happen in the past four weeks? (1=Rarely, 2 =Sometimes, 3 = Often)	0 = No (skip to Q.8)<br><br>1 = Yes	
6<br><br>6a	In the past four weeks, did you or any household member have to eat fewer meals in a day because there was not enough food?<br><br>How often did this happen in the past four weeks? (1=Rarely, 2=Sometimes, 3 = Often)	0 = No (skip to Q.8)<br><br>1 = Yes	
7<br><br>7a	In the past four weeks, was there ever no food to eat of any kind in your household because of lack of resources to get food?<br><br>How often did this happen in the past four weeks?	0 = No (skip to Q.8)<br><br>1 = Yes	
8<br><br>8a	In the past four weeks, did you or any household member go to sleep at night hungry because there was not enough food?<br><br>How often did this happen in the past four weeks?	0 = No (skip to Q.8)<br><br>1 = Yes	
9<br><br>9a	In the past four weeks, did you or any household member go a whole day and night without eating anything because there was not enough food?<br><br>How often did this happen in the past four weeks?	0 = No (skip to Q.8)<br><br>1 = Yes	

Child Dietary Diversity

A12	Now I would like to ask you about (other) liquids or foods that ***(NAME)*** ate yesterday during the day or at night. I am interested in whether your child had the item even if it was combined with other foods. For example, if ***(NAME)*** ate a millet porridge made with a mixed vegetable sauce, you should reply yes to any food I ask about that was an ingredient in the porridge or sauce. Please do not include any food used in a small amount for seasoning or condiments (like chilies, spices, herbs, or fish powder), I will ask you about those foods separately. Yesterday during the day or at night, did ***(NAME)*** drink/eat:		YES	NO	DK
A	Bread, rice, noodles, or other foods made from grains, including thick grain-based porridge?	A..........	1	2	8
B	Pumpkin, carrots, squash, or sweet potatoes that are yellow, or orange inside?	B..........	1	2	8
C	White potatoes, white yams, manioc, cassava, or any other foods made from roots?	C..........	1	2	8
D	Any dark green leafy vegetables?	D..........	1	2	8
E	Ripe mangoes, ripe papayas, or (**insert other local vitamin-A rich fruits**)?	E..........	1	2	8
F	Any other fruits, or vegetables?	F..........	1	2	8
G	Liver, kidney, heart, or other organ meats?	G..........	1	2	8
H	Any meat, such as beef, pork, lamb, goat, chicken, or duck?	H..........	1	2	8
I	Eggs?	I..........	1	2	8
J	Fresh or dried fish, shellfish, or seafood?	J..........	1	2	8
K	Any foods made from beans, peas, lentils, or nuts?	K..........	1	2	8
L	Cheese, yogurt, or other milk products?	L..........	1	2	8
M	Any oil, fats, or butter, or foods made with any of these?	M..........	1	2	8
N	Any sugary foods such as chocolates, sweets, candies, pastries, cakes, or biscuits?	N..........	1	2	8
O	Condiments for flavor, such as chilies, spices, herbs, or fish powder?	O..........	1	2	8
P	Grubs, snails, or insects?	P..........	1	2	8
Q	Foods made with red palm oil, red palm nut, red palm nut pulp sauce	Q..........	1	2	8
R	Any other solid or semi-solid food	R..........	1	2	8

Child Stimulatory Care

Family care indicator questionnaire

If the information giver (respondent) is other than mother, then mention his/her name:

………………………………………. …………………………………………..

I want to know about those things with which the child plays at home. Please show me those things. These may be home-made e.g. home-made clay-built toy, doll made up of cloths or toy which is bought & household materials etc. The question should be coded. Whenmother shows these toys, the question will help her to recall other toys present at home. Code only those toys which the mother can show. Not only the presence of the toys will do,but also these toys should be used for specific play or work mentioned in the following questions.

1. *In the last 30 days (Child name) did the child play with any toy that can make music or canbe played as musical instrument (e.g., musical instrument or the toys that produce musicalsound e.g., plastic mobile as toy, radio as toy, singing doll, tom-tom, pipe etc.)?*

   1= Yes 2= No

2. *In the last 30 days did the child (name) play with any toy that can be used for drawing or writing purpose (e.g., picture book for coloring, pencil, pen, chalk, slate or marking / writing withstick in the floor or courtyard etc.*

   1= Yes 2= No

3. *Is there any picture book suitable for the child (except schoolbook)?*

   1= Yes 2= No

4. *In the last 30 days did the child (name) play with anything that disguise himself or take the role of mother, doctor, teacher, actor, doll, plate & cup for acting purpose?*

   1= Yes 2= No

5. *In the last 30 days (Child name) did the child play with any toy with which he ran about (e.g., ball& bat, rope for jumping, rocking cradle made of rope, a car that can be pulled or pushed etc.)?*

   1= Yes 2= No

6. *Does the child have any toy with which he can get idea regarding shape (triangular, rectangular, round) & color?*

   1= Yes 2= No

7. *Does the child have any toy (globular shaped, logo, block) with which tower; house, car etc. can be made by placing them one over another or side by side.*

   1= Yes 2= No

   Instruction: Answer to the question number 8 & 9 should be in number. If the number is 10 or >10, thenthe answer should be 10.

8. *What is the number of books in your house including schoolbook (except picture book for children) ……?*

9. *What is the number of paper & magazine at your house?*

Now I want to know from you regarding some work or play which has been played with the child byyou or his father or any senior member of the family, in the last three days.

10. *Book was read or picture book/picture/poster was shown to your child by-*
    a. *Mother*
    b. *Father*
    c. *other family member who is above 15 years of old*
    d. *Notdone*

11. *Story was told to the child (name) by-*
    a. *Mother*
    b. *Father*
    c. *other family member who is above 15 years of old*
    d. *Notdone*

12. *Song, rhyme, religious song was sung to the child (name) by-*
    a. *Mother*
    b. *Father*
    c. *other family member who is above 15 years of old*
    d. *Notdone*

13. *Game was played with the child (name) using toys by-*
    a. *Mother*
    b. *Father*
    c. *other family member who is above 15 years of old*
    d. *Notdone*

14. *Name of something, counting number & drawing was taught to the child (name) by allocating time for him by-*
    a. *Mother*
    b. *Father*
    c. *other family member who is above 15 years of old*
    d. *Not done*

Bayley's Assessment

Bayley
Scales of Infant and Toddler Development™
THIRD EDITION

Record Form

Child's name: ______
Sex: ☐ M ☐ F ID #: ______
Examiner's name: ______
School/Child care program: ______
Reason for referral: ______

Subtest Summary Scores

Subtest	Total Raw Score	Scaled Score	Composite Score	Percentile Rank	Conf. Interval (___%)
**Cognitive** (Cog)					
			Use Table A.5		
**Language** (Lang)					
Receptive Communication (RC)					
Expressive Communication (EC)					
**Sum**					
			Use Table A.4		
**Motor** (Mot)					
Fine Motor (FM)					
Gross Motor (GM)					
**Sum**					
			Use Table A.4		
**Social-Emotional** (SE)					
			Use Table A.5		
**Adaptive Behavior**					
*Communication (Com)					
Community Use (CU)					
Functional Pre-Academics (FA)					
Home Living (HL)					
*Health and Safety (HS)					
*Leisure (LS)					
*Self-Care (SC)					
*Self-Direction (SD)					
*Social (Soc)					
*Motor (MO)			(GAC)		
**Sum**					
			Use Table A.6		

*For children younger than one year, the GAC is calculated using only those skill areas indicated by an asterisk.

Calculate Age and Start Point

	Years	Months	Days
Date Tested			
Date of Birth			
Age			
Age in Months and Days	Years × 12 + months		
Adjustment for Prematurity	Adjust through 24 months		
Adjusted Age			
Start Point	Calculate start point according to chart below		

Age	Start Point
16 days–1 month 15 days	A
1 month 16 days–2 months 15 days	B
2 months 16 days–3 months 15 days	C
3 months 16 days–4 months 15 days	D
4 months 16 days–5 months 15 days	E
5 months 16 days–6 months 15 days	F
6 months 16 days–8 months 30 days	G
9 months 0 days–10 months 30 days	H
11 months 0 days–13 months 15 days	I
13 months 16 days–16 months 15 days	J
16 months 16 days–19 months 15 days	K
19 months 16 days–22 months 15 days	L
22 months 16 days–25 months 15 days	M
25 months 16 days–28 months 15 days	N
28 months 16 days–32 months 30 days	O
33 months 0 days–38 months 30 days	P
39 months 0 days–42 months 15 days	Q

ISBN 015402723-5

PEARSON

Copyright © 2006, 1993, 1984, 1969 by NCS Pearson, Inc.
All rights reserved. Printed in the United States of America.
8 9 10 11 12 A B C D E

PsychCorp

9780154027238

Telephone: + 265 726 422
Facsimile: + 265 726 418
E-mail: mohdoccentre@gmail.com

All Communications should be addressed to:
The Chairperson

In reply please quote No. MED/4/36c

MINISTRY OF HEALTH
P.O. BOX 30377
LILONGWE 3
MALAWI

NATIONAL HEALTH SCIENCES RESEARCH COMMITTEE
MATERIAL TRANSFER AGREEMENT (MTA) FORM

Protocol Number(s):

NHSRC: 20/11/2622 **PMRA:** PMRA/CTRC/III/08062021130

Title of protocol:

Randomized controlled trial of the Effect of intraVenous iron on Anaemia in Malawian Pregnant women – Third Trimester (REVAMP-TT Study)

Intention and Justification of transfer:

Blood, placental tissue, vaginal swabs and stool samples will be shipped to the Walter and Eliza Hall Institute of Medical Research, in Melbourne, Australia where one of the Study Principal Investigators is based for advanced tests that are currently not available in Malawi. Blood samples include whole blood as well as cellular, serum and plasma fractions.

All samples are required to in order to conduct the immune, metabolic and molecular laboratory analyses which will be required to derive some of the study's secondary outcomes, as clearly stated in the protocol.

Duration of storage:

All exported samples will be stored for a period of not more than 5 years and any remaining samples will be destroyed soon after the laboratory analyses have been completed.

Responsible Party:

- Prof Kamija Phiri, Department of Public Health, College of Medicine, University of Malawi, Private Bag 360, Chichiri, Blantyre 3, Malawi. Mobile +265 999957 048; E-mail: kphiri@medcol.mw
- Dr Sant-Rayn Pasricha, Walter and Eliza Hall Institute of Medical Research, 1G Royal Parade, Parkville, Melbourne 3052, Australia. Mobile: +61407141570; E-mail: Pasricha.s@wehi.edu.au

Location of stored samples:

- In Malawi prior to exporting the samples will be stored at the College of Medicine, University of Malawi, Private Bag 360, Chichiri, Blantyre 3, Malawi. Mobile +265 999957 048
- Second location in Malawi prior to exporting will be at Zomba Central Hospital, Training and Research Unit of Excellence (TRUE) Research laboratory. P.O. Box 30538 Chichiri BT 3, Blantyre, Malawi. Mobile +265 88 122 2672

In Australia samples will be stored at Walter and Eliza Hall Institute of Medical Research, 1GRoyal

Telephone: + 265 726 422
Facsimile: + 265 726 418
E-mail: mohdoccentre@gmail.com

All Communications should be addressed to:
The Chairperson

In reply please quote No. MED/4/36c
MINISTRY OF HEALTH
P.O. BOX 30377
LILONGWE 3
MALAWI

Parade, Parkville, Melbourne 3052, Australia. Mobile: +61407141570
**Transportation of samples:**
Packaging and transportation of the samples will be as per requirements by the IATA guidelines andregulations on shipment of biological materials.
**Ownership of samples:**
The Government of Malawi will maintain ownership of all samples through the College of Medicine,University of Malawi and the Training and Research Unit of Excellence (TRUE).
**After all laboratory testing has been completed: Describe what will happen to the samples**
As stated in this Material Agreement Transfer form, all samples will be kept for a maximum of 10 yearsafter which the samples will be destroyed.
**Appropriate informed consent authorising the exportation and importation of samples**
The trial's Informed Consent Form clearly seeks authorization from the participants with regards to the export of all necessary samples, as well as provides information concerning the reasons for that sample export as well as contact numbers that can be reached if more information is required.
**To whom will the samples be accessible**
The samples will only be accessible to assigned laboratory staff at the College of Medicine, University of Malawi (during preparation and transfer) and the laboratory staff directly involved in sample analyses forthe Clinical Trial at the Walter and Eliza Hall Institute of Medical Research, in Melbourne, Australia. In caseof any analyses that might require a third party involvement, this will only be done with the full agreementof the trial's Principal Investigators.
**Who will be the controlling officers of the samples**
**Dr Sant-Rayn Pasricha**, Walter and Eliza Hall Institute of Medical Research, 1G Royal Parade,Parkville, Melbourne 3052, Australia. Mobile: +61407141570; E-mail: Pasricha.s@wehi.edu.au

Samples collected in Malawi may not be sold without prior permission from the collaboratingor controlling institutions and the NHSRC in Malawi.

Telephone: + 265 726 422
Facsimile: + 265 726 418
E-mail: mohdoccentre@gmail.com

All Communications should be addressed to:
The Chairperson

In reply please quote No. MED/4/36c

MINISTRY OF HEALTH
P.O. BOX 30377
LILONGWE 3
MALAWI

Signed by

Name of the PI:
Prof. Kamija Phiri

Name of Institution:

Training and Research Unit of Excellence (TRUE)

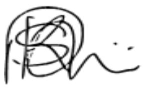

Australia
Signature:

Date Signed: 16/01/2022

Name of Co – PI
Dr. Sant-Rayn Pasricha,

Name of Institution:

WEHI, Melbourne,

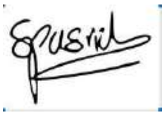

Signature:

Date Signed: 16/01/2022

NHSRC APPROVAL

Name of the Chairperson:

Signature:

Date Signed:

Name of Secretary

Signature:

Date Signed:

NHSRC STAMP OF APPROVAL:

Changes to the Protocol (between version 1.0 and version 3.1)

Changes to Protocol (between Version 1.0 and Version 3.1)

These amendments comprised:

from the protocol v.1.0 to v.1.1:

- Reduced the number of intervention arms from three to two after the manufacturer of Iron Isomaltoside (IIM) could not supply the Investigational Product nor the necessary documentation for use in the trial. Since the primary goal of the trial did not include a direct comparison between intravenous (IV) iron drugs, but rather evaluating the efficacy of IV iron drugs in comparison to standard of care oral iron in reducing anaemia in pregnant women in their third trimester, the trial remained relevant without the inclusion of IIM

from the protocol v1.1 to 2.1

- Added measures of the proportion of women with hypophosphatemia (clinical and biochemical) at baseline, 36 weeks gestation, delivery, 28 days postpartum, 3 months postpartum, 6 months postpartum and 12 months postpartum. The additional timepoints were to give a more complete measurement of hypophosphatemia as a maternal safety outcome
- Added assessment of child neurodevelopment scores measured by low field magnetic resonance imaging at 3 and 12 months of age as a key secondary efficacy outcome during their follow-up beyond the main trial
- Added assessment of the proportion of infants of trial participants with radiological Rickets at 3 and 12 months of age as a safety outcome during their follow-up beyond the main trial

from the protocol 2.1 to 3.1

- Added provision for interim analyses for adaptive sample size recalculation with the potential to increase the sample size, based on new data from the REVAMP trial on the assumed treatment effect underlying the ongoing REVAMP-TT trial. A sample size re-estimation was conducted and the proposal to increase the sample size was withdrawn, based on sample size re-evaluation and a data safety monitoring committee recommendation
- Clarified the definition of the primary timepoint of the primary outcome (anaemia defined as venous hemoglobin<11.0g/dl) from 36 week's gestation to 36 week's gestation or delivery, whichever comes first. This allowed to take into account data from women who delivered prior to week 36 of gestation, reflecting of our primary interest in the woman's anaemia status around 36 week's of gestation
- Addition of continuous birth weight and birth length outcomes to complement the outcomes low birth weight and growth specified in protocol v1.0
- Addition of time point of 1 month to infant haemoglobin, inflammation and iron outcomes to align with trial registry

REVAMP-TT Statistical Analysis Plan version 2.0

Statistical Analysis Plan

TRIAL FULL TITLE	A Randomized controlled trial of the Effect of intraVenous iron on Anaemia in Malawian Pregnant women in their Third Trimester
TRIAL SHORT TITLE	REVAMP-TT
TRIAL REGISTRATION	Australian New Zealand Clinical Trials Registry Number: ACTRN12621001239853
PROTOCOL VERSION	3.1
TRIAL CHIEF INVESTIGATORS	Professor Kamija Phiri, University of Malawi<br>Professor Sant-Rayn Pasricha, Walter and Eliza Hall Institute of Medical Research
TRIAL STATISTICIAN	Dr Rebecca Harding, Walter and Eliza Hall Institute of Medical Research
SAP AUTHORS	Dr Rebecca Harding, Walter and Eliza Hall Institute of Medical Research<br>Ms Sabine Braat, University of Melbourne/Walter and Eliza Hall Institute of Medical Research<br>Dr Ricardo Ataide, Walter and Eliza Hall Institute of Medical Research
SAP VERSION	2
SAP VERSION DATE	11 June 2024

SAP Revision History

Version	Reason(s) for change	Date
1.0	Initial version for Protocol and SAP Publication	13 April 2023
1.1	Version before database unblinding incorporating reviewers' comments from the Gates Open Research Publication and review by the Data Monitoring Committee	19 Dec 2023
2.0	Version after database lock and unblinding	11 June 2024

SAP Signatures

I give my approval for the attached SAP entitled REVAMP dated 11 June 2024

Chief Investigators

Name / Affiliation	Signature	Date / time / time zone
Prof Kamija Phiri, University of Malawi	[redacted]	11 June 2024
Prof Sant-Rayn Pasricha, Walter and Eliza Hall Institute of Medical Research	[redacted]	11 June 2024

Trial Coordinator

Name / Affiliation	Signature	Date / time / time zone
Dr Ricardo Ataide, Walter and Eliza Hall Institute of Medical Research	[redacted]	11 June 2024

Trial Statistician

Name / Affiliation	Signature	Date / time / time zone
Dr Rebecca Harding, Walter and Eliza Hall Institute of Medical Research	[redacted]	11 June 2024

Senior Statistician

Name / Affiliation	Signature	Date / time / time zone
Ms Sabine Braat, University of Melbourne/Walter and Eliza Hall Institute of Medical Research	[redacted]	11 June 2024

Table of Contents

Introduction 7
1.1 Preface 7
1.2 Purpose of the analyses 8
2 Study Objectives and Endpoints 8
2.1 Study Objectives 8
2.2 Endpoints 9
3 Study Methods 10
3.1 General Study Design and Plan 11
3.2 Inclusion-Exclusion Criteria and General Study Population 12
3.3 Randomisation and Blinding 13
3.4 Study Visit Schedule 13
3.5 Study Variables 17
3.5.1 Maternal Study Variables 17
3.5.2 Neonate/Infant Study Variables 17
4 Sample Size 18
5 General Considerations 19
5.1 Timing of Analyses 19
5.2 Analysis Populations 21
5.2.1 Intention-to-Treat Population 21
5.2.1.1 Mothers 21
5.2.1.2 Neonates/infants 21
5.2.2 Per Protocol Population 21
5.2.2.1 Mothers 21
5.2.2.2 Neonates/infants 22
5.2.3 Safety Population 22
5.2.3.1 Mothers 22
5.2.3.2 Neonates/infants 22

5.2.4 Modified Safety Population 22
5.2.4.1 Mothers 22
5.2.4.2 Neonates/infants 22
5.3 Covariates and Subgroups 22
5.3.1 Covariates 23
5.3.1.1 Maternal Covariates 23
5.3.1.2 Neonate/Infant Covariates 23
5.3.2 Subgroups 24
5.4 Missing Data 24
5.4.1 Maternal outcomes 24
5.4.2 Neonate/Infant Outcomes 26
5.5 Interim Analyses and Data Monitoring 27
5.6 Multi-centre Studies 28
5.7 Multiple Testing 28
6 Summary of Study Data 30
6.1 Subject Disposition 30
6.2 Demographic and Baseline Variables 31
6.3 Treatment Compliance 32
7 Effectiveness Analyses 32
7.1 Primary Estimand 33
7.2 Maternal Study Variables 36
7.2.1 Maternal Anaemia 36
7.2.2 Secondary Maternal Analyses 37
7.3 Neonate/Infant Study Variables 38
7.3.1 Birth weight 38
7.3.2 Secondary Neonate/Infant Analyses 39
8 Safety Analyses 40
8.1 (Serious) Adverse Events 40

8.1.1 Maternal 41
8.1.2 Neonates/Infants 42
8.2 Safety biomarkers/Clinical Laboratory Evaluations 42
8.2.1 Maternal 42
8.2.2 Neonates/infants 43
9 Technical Details 43
10 Validation of Output 43
11 Summary of Changes to the Registry and Protocol 43
12 Summary of Changes to the Statistical Analysis Plan 44
13 References 46
14 Listing of Key Tables, Listings and Figures 49

Abbreviations and Definitions

ABBREVIATION	DEFINITION
AE	Adverse Event
ANC	Antenatal Care
BMI	Body Mass Index
CI	Confidence Interval
CONSORT	Consolidated Standards of Reporting Trials
CRF	Case Report Form
CRP	C-Reactive Protein
dL	Decilitre
DMC	Data Monitoring Committee
FCM	Ferric Carboxymaltose
GEE	Generalised Estimating Equations
Hb	Haemoglobin
HC	Health Centre
HIV	Human Immunodeficiency Virus
ICU	Intensive Care Unit
ID	Iron Deficiency
IDA	Iron Deficiency Anaemia
IPTp	Intermittent Preventive Treatment in pregnancy
ITT	Intention-To-Treat
IV	Intravenous
LMICs	Low- and Middle-Income Countries
MAR	Missing At Random
MCAR	Missing Completely At Random
MNAR	Missing Not At Random
NNT	Number-Needed-to-Treat
PO4	Phosphate
PP	Per Protocol
RCT	Randomised Controlled Trial
RDT	Rapid Diagnostic Test
SAE	Serious Adverse Event
SAP	Statistical Analysis Plan
SOC	Standard-of-Care
SP	Sulfadoxine-Pyrimethamine
TRUE	Training & Research Unit of Excellence
WEHI	The Walter and Eliza Hall Institute of Medical Research
WHO	World Health Organization

Introduction

1.1 Preface

Anaemia during pregnancy remains a critical global health problem. Almost 40% of pregnant women worldwide are anaemic, including 46% of pregnant women in Africa and 49% in Asia (2). Anaemia in pregnancy is very common and mostly results from iron deficiency and is associated with critical risks for both mother (e.g., life-threatening complications of postpartum haemorrhage) and child (especially prematurity and low birth weight, which are associated with increased risk of neonatal and infant mortality, and reduced iron stores in infancy with increased risk of subsequent anaemia and impaired development). (3-6)

Control of maternal anaemia, an important contributor to maternal mortality, is a key World Health Organization (WHO) nutrition target and a component of the third Sustainable Development Goal (7). Treatment with oral iron is poorly tolerated (8) and requires extended contact with a well-functioning health system; this is difficult to achieve, and few women receive full courses of iron. A new intravenous (IV) iron formulation, ferric carboxymaltose (FCM), now routinely available and used in developed countries, is safe and provides the opportunity to give high doses of iron in a single rapid infusion (9). In low and middle-income countries (LMICs), IV iron could present a novel, innovative opportunity to rapidly cure moderate and severe antenatal anaemia, thereby improving crucial maternal and neonatal outcomes.

REVAMP-TT (Randomized controlled trial of the effect of intravenous iron on anaemia in Malawian Pregnant women in their third trimester) is a multicentre, open-label, two-arm, parallel-group randomised control trial (RCT) in health centres across the Zomba district in Malawi (10) (ACTRN12621001239853). This is the sister trial to the second-trimester **REVAMP** trial, funded by the Bill and Melinda Gates Foundation (trial registration ACTRN12618001268235, Gates Grant number INV-010612), which was published 2023(11). Women are eligible if they are in the third trimester of pregnancy and have a capillary haemoglobin concentration below 10.0g/dL. The study comprises two arms: (a) Intravenous Ferric Carboxymaltose (1000mg for women with weight >50kg, and 20mg/kg for women with weight <50kg) given once after randomisation (N=295), and (b) oral iron (65mg elemental iron twice daily) for the remainder of pregnancy, provided according to local health care practices (N=295). In addition, both arms receive sulfadoxine-pyrimethamine (SP) as IPTp according to

national guidelines. Participants are followed up until one month postpartum, after which we will report on the primary and secondary objectives.

1.2 Purpose of the analyses

This Statistical Analysis Plan (SAP) aims to outline the pre-planned analyses to be completed to support the main publication of the REVAMP-TT study. Versions of the SAP will be tracked pre and post-unblinding, with a clear distinction between the changes before and after unblinding. Any analyses not identified in the SAP after unblinding of the randomised study treatment will be clearly identified as such in the main publication and will be considered post-hoc. In addition, follow-up of mothers and infants from one-month postpartum to 12 months postpartum consists of a range of exploratory economic, biological, cognitive, and clinical outcomes. These analyses are beyond the scope of this SAP, as are the exploratory outcomes collected up to 12-months postpartum and will be reported separately.

2 Study Objectives and Endpoints

2.1 Study Objectives

The **primary objective** is to determine whether a single dose of FCM up to 1000mg is superior to standard-of-care (i.e., oral iron provided through local health services) in reducing maternal anaemia at 36 weeks' gestation or at delivery, whichever comes first in Malawian women in the third trimester of pregnancy with moderate or severe anaemia.

The **secondary objectives** are to determine the effects of FCM (compared with standard-of-care) during pregnancy and up to one month postpartum on:

Effectiveness:

- Maternal haemoglobin concentration and iron status (measured through iron biomarkers), and
- Critical neonatal outcomes including birth weight (low birth weight), and other perinatal outcomes,

Safety:

- Maternal and neonatal adverse events, including infection episodes, serious maternal complications, and hypophosphatemia.

2.2 Endpoints

To address the primary objective, we use the **primary endpoint** of maternal anaemia, defined as a venous haemoglobin concentration less than 11.0g/dL at 36 weeks' gestation or at delivery, whichever comes first (12).

To address the secondary objectives, we use the **secondary endpoints** as per below:

Effectiveness:

Maternal key secondary:

- haemoglobin concentration at 36 weeks' gestation or at delivery, whichever comes first
- serum ferritin concentration at 36 weeks' gestation or at delivery, whichever comes first
- haemoglobin concentration at 1 month postpartum
- serum ferritin concentration at 1 month postpartum

Maternal other secondary:

- anaemia (Hb <11.0g/dL) at delivery
- anaemia (Hb <12.0g/dL) at 1 month postpartum
- moderate/severe anaemia (Hb <10.0g/dL) at 36 weeks' gestation or at delivery, whichever comes first
- moderate/severe anaemia (Hb <10.0g/dL) at delivery
- moderate/severe anaemia (Hb <11.0g/dL) at 1 month postpartum
- haemoglobin concentration at delivery
- serum ferritin concentration at delivery
- iron deficiency (serum ferritin<15ug/L or ferritin<30ug/L if C-reactive protein>5mg/L) at 36 weeks' gestation or at delivery, whichever comes first
- iron deficiency (serum ferritin<15ug/L or ferritin<30ug/L if C-reactive protein>5mg/L) at delivery
- iron deficiency (serum ferritin<15ug/L or ferritin<30ug/L if C-reactive protein>5mg/L) at 1 month postpartum
- iron-deficiency anaemia (Hb<11g/dL and serum ferritin<15ug/L or ferritin<30ug/L if C-reactive protein>5mg/L) at 36 weeks' gestation or at delivery, whichever comes first
- iron-deficiency anaemia (Hb<11g/dL and serum ferritin<15ug/L or ferritin<30ug/L if C-reactive protein>5mg/L) at delivery

- iron-deficiency anaemia (Hb<11g/dL and serum ferritin<15ug/L or ferritin<30ug/L if C-reactive protein>5mg/L) at 1 month postpartum

Neonate key:

- birth weight within 24 hours of birth

Neonate secondary:

- haemoglobin concentration (venous) at 1 month of age
- ~~serum ferritin concentration at 1 month of age~~
- neonate weight at 1 month of age

Neonate other:

- birth length within 24 hours of birth
- low birth weight (<2500grams) within 24 hours of birth
- stillbirth
- neonate length at 1 month of age
- infant growth (weight for age, length for age, weight for length) at 1-month postpartum.

Safety:

Maternal:

- (serious) adverse events related to administration of FCM (events occurring immediately post-infusion)
- (serious) adverse events
- hypophosphatemia (biochemical; defined as Phosphate (PO4)<0.80 mmol/L) by study visit
- inflammation (elevated C-reactive protein) by study visit
- severe medical events including haemorrhage, need for transfusion, ICU admission, or mortality.

Neonate:

- (serious) adverse events
- ~~hypophosphatemia (biochemical; defined as Phosphate (PO4)<0.80 mmol/L)~~
- severe medical events including mortality.

3 Study Methods

3.1 General Study Design and Plan

This study is a multicentre, open label, two-arm, parallel-group individually randomized controlled effectiveness and safety trial carried out in real-life settings in health centres across the Zomba district of Southern Malawi. An open-label trial was chosen as it was considered unfeasible to deliver dark-coloured placebo intravenous infusions to Malawian pregnant women in this field context.

The trial randomized 590 pregnant women in their third trimester equally to (a) IV ferric carboxymaltose 1000mg (for women with weight >50kg), or 20mg/kg (for women with weight <50kg) once during the third trimester; or (b) oral iron 200mg ferrous sulphate (approx. 65mg elemental iron) twice daily for remainder of pregnancy. Women received interventions and then were followed up until one-month postpartum. The database will be locked, and the trial unblinded to report prespecified outcomes once all participants reach one-month postpartum. Planned extended follow-up of mothers and infants to 12-months postpartum will be reported in subsequent documents. The trial design is summarised in **Figure 1**.

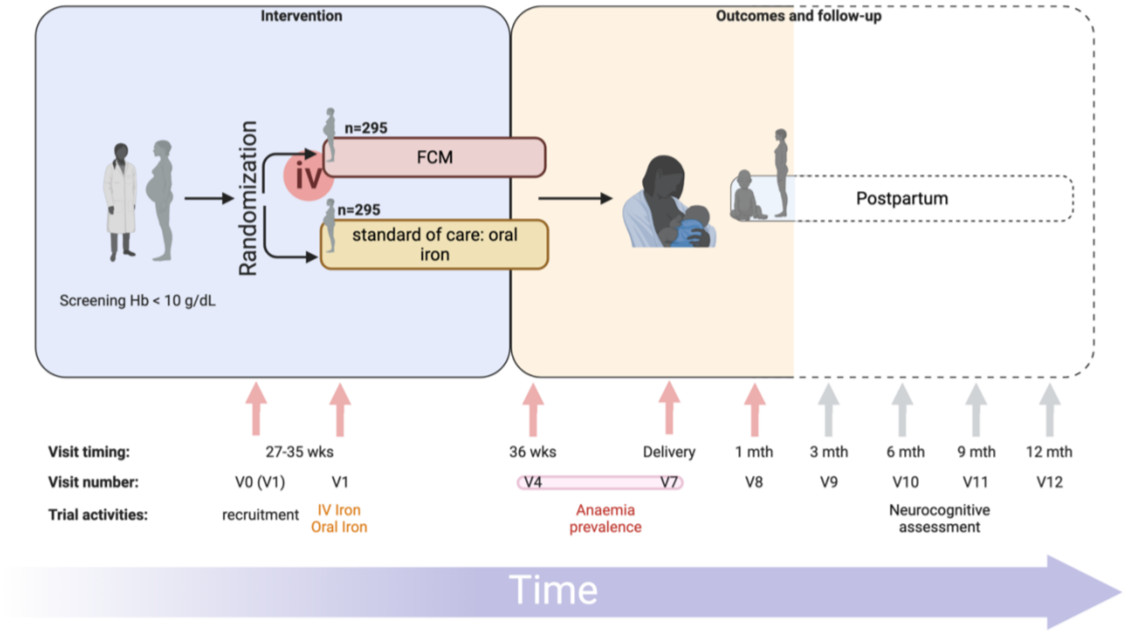

Figure 1 REVAMP-TT Trial Schema

Trial design with visit timings, visit numbers (standardized to match those of REVAMP trial – P.02/18/2357) and main trial activities are represented. The study was designed as a two-arm trial (intravenous ferric carboxymaltose versus standard-of-

care (oral iron)) where women were randomized in their third trimester of pregnancy. Study visits occurred over pregnancy, at birth, initial follow-up to one-month postpartum and extended follow-up to 12-months postpartum. Primary outcome measured at V4 or V7, indicated by the red bar connecting both visits. Dotted lines represent follow-up visits not included in the main trial. Abbreviations: FCM, Ferric Carboxymaltose; iv, intravenous; mth, months; REVAMP-TT, Randomized controlled trial of the Effect of intraVenous iron on Anemia in Malawian Pregnant women – Third Trimester; V0, visit 0; V1, visit 1; V4, visit 4; V7, visit 7; V8, visit 8; V9, visit 9; V10, visit 10; V11, visit 11; V12, visit 12; wks, weeks. Figure prepared using BioRender.

3.2 Inclusion-Exclusion Criteria and General Study Population

Women were randomized only if they fulfilled all the inclusion criteria and none of the exclusion criteria.

Inclusion criteria:

1. Confirmed singleton pregnancy in the third trimester (27-35 weeks of gestation, dated by Last Menstrual Period and/or fundal height).
2. Moderate to severe anaemia not requiring an immediate blood transfusion (Hb <10g/dL).
3. Negative malaria parasitaemia by RDT.
4. Currently afebrile with no evidence of septicaemia.
5. Resident in the study catchment area of Zomba district.
6. Able to deliver at health facilities within Zomba district.
7. Written informed consent (including assent if <18 years old).

Exclusion criteria:

1. Previous enrolment in REVAMP trial (REVAMP trial – P.02/18/2357)
2. Actively participating in another intervention trial.
3. Known hypersensitivity to any of the study drugs.
4. Clinical symptoms of malaria or other infection (no fever, no focal symptoms of internal infection i.e. LRTI/ diarrhoea).
5. Any condition requiring hospitalisation in the next seven days or serious concomitant illness.
6. Known history of sickle cell or sickle-haemoglobin C anaemia.
7. Clinically low haemoglobin level requiring a blood transfusion (usually Hb <5g/dl).

8. Preeclampsia.

3.3 Randomisation and Blinding

Participants are randomly allocated to one of the two treatments arms with 1:1 allocation via a computer-generated randomisation schedule of randomly permuted blocks stratified by site to achieve balance between the arms within each site. The randomisation list was generated by an independent statistician at the University of Melbourne (Australia) who will not reveal the block size until the database is ready for unblinding.

Individual participant codes are pre-packed in opaque envelopes by an independent researcher not associated with the study and held securely at research sites. Eligible participants who meet all inclusion/exclusion criteria are sequentially allocated participant identification numbers within the research site, and their allocation to study group is revealed after opening the corresponding sealed envelope.

Although the trial is open-label, laboratory scientists measuring haemoglobin concentration, midwives collecting birth outcome data, investigators and researchers in Australia (including data managers and statisticians in Melbourne) are blinded to the allocation during the conduct of the trial until the database is locked and ready for unblinding.

An open-label (rather than blinded) trial was chosen as it was deemed that it would be ethically and operationally challenging to provide placebo infusions to women during pregnancy. In particular, FCM is black in colour, and thus would require either use of opaque tubing, a curtain and veil to conceal the tubing or a blindfold for the participant – none of which are likely to be feasible in practice.

3.4 Study Visit Schedule

Table 1 shows the schedule of activities per visit for the mother, and Table 2 shows the schedule of activities per visit for the neonate/infant.

Table 1: Maternal Study Visit Schedule

Protocol Activity	Prescreening (Visit 0)	Enrolment (Visit 1)	36 weeks gestation (Visit 4)	Delivery (Visit 7)	28 days postpartum (Visit 8)	Unscheduled visits
Pre-screening form	X					

completion						
Screening	X					
Informed consent process		X				
Medical & obstetric history		X				
Household economic data form		X				
Household food insecurity			X			
Complete physical examination [a]		X				
Limited physical examination [b]			X	X	X	
Edinburgh Postpartum depression scale (EPDS)		X				
Participant arm allocation		X				
Administer treatment						
intravenous iron		X				
Oral iron		X				
Laboratory procedures (Maternal)						
Full Blood Count (including Hb)		X	X	X	X	
Haemoglobin (capillary)	X					
Malaria RDT		X				X
Malaria microscopy		X	X	X	X	X
Malaria filter paper for PCR		X	X	X	X	X
Serum for iron markers tests[c]		X	X	X	X	
Serum for inflammatory markers tests[d]		X	X	X	X	
Phosphate		X	X	X	X	
Vaginal swab sample collection[e]			X		X	
Placenta histology				X		
Breast milk sample[f]					X	

[a] Complete examination: general appearance, throat, neck, thyroid, musculoskeletal, skin, lymph nodes, extremities, pulses, pulmonary, cardiac, abdominal, and neurological examination

[b] Limited examination: general appearance, brief pulmonary, cardiac, abdominal, and neurological examination

[c] e.g., Serum ferritin

[d] e.g., CRP

[e] Reproductive tract microbiome analysis: e.g., Chlamydia trachomatis, Neisseria gonorrhoeae, Trichomonas vaginalis, Lactobacillus spp. (done only for a sub-set of 50 women)

[f] Done only if mothers are still breast feeding

Table 2 Neonate Study Visit Schedule

Protocol Activity	Prescreening (Visit 0)	Enrolment (Visit 1)	36 weeks gestation (Visit 4)	Delivery (Visit 7)	28 days postpartum (Visit 8)	Unscheduled visits
Pregnancy outcome				X	X	
Birth weight				X		
Complete physical examination of baby [a]				X		
Limited physical examination of baby [b]					X	
Maternal anthropometry: weight, height				X	X	
Child anthropometry: weight, length, head circumference				X	X	
Child vaccination and Vit A supplementation status					X	
Infant neurodevelopment using auditory brainstem responses (ABRs)					X	
Laboratory procedures (Infant)						

Full Blood Count				X (cord)	X	
Malaria microscopy				X (cord)	X	
Serum for iron markers tests[c]				X (cord)	X	
Phosphate				X (cord)	X	
Malaria filter paper for PCR				X (cord)	X	
Stool sample					X	
Infant neurodevelopment using auditory brainstem responses (ABRs)					X	

[a] Complete examination: weight, length, head circumference, APGAR score, Ballard score, new-born adiposity, congenital anomaly, and complications at birth

[b] Limited examination: general appearance, brief pulmonary, cardiac, abdominal, and neurological examination

[c] e.g., Serum ferritin

In accordance with the study visit schedule, we will use time-windows to convert visit dates into visit numbers. Relative days will be derived as the visit date minus the randomisation date. The visit windows in Table 3 will be applied to the relative days.

Table 3 Visit windows

Visit	Target Day	Lower limit	Upper limit
Visit 1 (Clinic)<br>Day 0 – Randomisation date	Day 0	-7 days	0 days
Visit 4 (Clinic)<br>36 weeks' gestation	36 weeks + 0 days gestation	32 weeks + 0 days	40 weeks + 0 days
Visit 7 (Clinic)*<br>Delivery	Day of delivery	-2 days **	2 days post-delivery
Visit 8 (Clinic)*<br>28 days postpartum	28 days post-delivery	21 days post-delivery	42 days post-delivery

*This visit window applies to both mother and neonates
**This lower limit only applies to mothers, the lower limit of delivery for neonates is the day of delivery.

Assessments performed outside these visit windows will not be included in the analyses. If a study participant has more than one assessment done within the same visit window, then the assessment closest to the target date will be used. The visit windows will be applied to the study variables described below in Section 3.5.

3.5 Study Variables

A detailed description of the effectiveness study variables, grouped according to domain, is presented in Sections 3.5.1 to 3.5.2. We define the domains as maternal (including women who experienced still birth) and neonate/infants. All safety study variables are detailed in Section 8. Note that while C-reactive protein is discussed in Section 3.5.1, it is considered a safety study variable.

3.5.1 Maternal Study Variables

At baseline, 36 weeks' gestation, delivery, and 1-month postpartum, venous blood samples are collected from the mother to obtain the mothers haemoglobin (g/dL), ferritin (µg/L), and C-reactive protein (µg/L). Values of C-reactive protein below the lower limit of detection will be replaced by the half this value. In the case of haemolysis (rare occurrence), ~~C-reactive protein~~ phosphate will be set to missing. We will then derive the following variables:

- anaemia (haemoglobin<11.0 g/dL) or not up to and including delivery,
- anaemia (haemoglobin<12.0 g/dL/0 or not postpartum,
- mild anaemia (10.0 g/dL≤haemoglobin<11.0 g/dL), moderate anaemia (7.0 g/dL≤haemoglobin<10.0 g/dL), severe anaemia (haemoglobin<7.0 g/dL), or not up to and including delivery,
- mild anaemia (11.0 g/dL≤haemoglobin<12.0 g/dL), moderate anaemia (8.0 g/dL≤haemoglobin<11.0 g/dL), severe anaemia (haemoglobin<8.0 g/dL), or not postpartum,
- iron deficient (ID) (ferritin<15 ug/L or ferritin <30 ug/L if C-reactive protein >5 µg/L) or not,
- iron deficient anaemia (IDA) (iron deficient and anaemia) or not,
- inflammation (C-reactive protein >5µg/L) or not, and
- anaemia and inflammation (anaemia and inflammation).

3.5.2 Neonate/Infant Study Variables

Stillbirth (pregnancy loss after 22 weeks and prior to delivery with no evidence of life at delivery) and neonatal death (death in the first 28 days of life of a child with

evidence of life following delivery) will be recorded. Within 24 hours of delivery, live-born neonates will have a full physical examination including measurement of birth weight (grams) and birth length (cm). Using these physical examination indices, the study variable low birth weight (birth weight < 2500g) or not will be derived.

At 1-month of age, venous blood samples are collected from the child to obtain the child's haemoglobin (g/dL) ~~and ferritin (µg/L)~~. Also, the child's weight and length will be collected. Together with the age and sex of the child, z-scores will be derived for length-for-age, weight-for-age, and weight-for-length according to age and sex-specific WHO international reference growth standards (13). We will be using the WHO Anthro Software on the WHO website using the web-links available on the webpage https://www.who.int/childgrowth/software/en/.

4 Sample Size

The sample size estimation was 295 women per arm, or 590 women in total when accounting for a 10% drop out at the primary outcome; proportion of women with anaemia at 36 weeks' gestation or delivery, whichever comes first (defined as venous Hb<11.0g/dL). We will be able to detect a reduction in the proportion of women with anaemia from 63% in the oral iron arm to 49% in the IV iron arm with 90% power (two-sided 5%). We assumed that the mean Hb in oral iron treated women is 10.5g/dL and the standard deviation (SD) is 1.5g/dL, thus 63% of women are expected to be anaemic (Hb<11g/dL) at 36 weeks after oral iron. We assumed that the prevalence of anaemia in IV iron treated women would be 14% lower than that of women in the oral iron arm following the pivotal FCM vs oral iron trial. The Fer-ASAP trial demonstrated a 14% reduction in absolute anaemia prevalence compared with oral iron(14).

For our key secondary outcome of birth weight, after accounting for a miscarriage and stillbirth rate of 1%, we will be able to detect a birth weight increase of 100g to 150g in the IV iron arm compared to the oral iron arm with 72% to 97% power (two-sided 5% alpha). A birth weight increase of 100g is conservatively based on achieving an improvement two thirds as large as the 150g improvement seen in women randomized to oral iron versus control with high adherence in a recent Kenyan trial of oral antenatal iron which achieved 100% adherence (and noting that the control arm in our trial still involves giving iron, potentially ameliorating some of the effect). We assume a SD of 450g(15).

Sample size calculations were performed using Stata/SE (StataCorp. 2019. College Station, TX: StataCorp LLC).

Whilst the pre-planned sample size was 590 participants, the RCT design included an adaptive sample size re-estimation procedure as per the 'promising zone' methodology of Mehta and Pocock(16) once the outcomes of at least 50% of the recruited participants were obtained. Details are outlined in a stand-alone interim statistical analysis plan. In short, this procedure involves the evaluation of the conditional power during an interim analysis conducted by an independent unblinded statistician. If the conditional power falls in a pre-specified 'promising zone', then the sample size should be increased, with a potential increase of up to 260 participants to the prespecified maximum of 850 participants total (including loss to follow up), with the aim to achieve a conditional power of 90%. Otherwise, if the conditional power does not fall within this 'promising zone', then the planned sample size of 590 participants will be maintained. The 'promising zone' for conditional power was defined as between 0.388 and 0.9, as per Mehta and Pocock(16) methodology using a one-tailed value of alpha of 0.025 and power of 0.9. There will be no impact on the Type I error rate and thus no adjustment will be needed to the two-sided significance level of 5%.

The pre-planned sample size of 590 participants was confirmed as the final sample size after execution or the method by an independent unblinded statistician using the data from 77.8% (459/590) of the participants and recommendation from the Data Monitoring Committee. Sample size re-estimation was performed using a user-written program (Excel version 16.71).

5 General Considerations

5.1 Timing of Analyses

Study data are entered and stored in REDCap at the Training and Research Unit of Excellence (TRUE), Malawi. Laboratory indices are analysed at TRUE and Meander Medical Centre laboratory, accreditation number M040, EN ISO 15189:2012 (Amersfoort, The Netherlands) (for ferritin, phosphate, C-reactive protein). After all data supporting the main results are available and have been cleaned (except ferritin, phosphate, and C-reactive protein due to its potential unblinding nature) a blinded data review meeting will be held prior to database lock (defined as its state is kept

constant). During this blinded data review meeting, at least the following topics will be discussed and decided upon without knowledge of the underlying treatment code:

1. Study participants (mothers or neonates/infants) who have withdrawn consent, in relation to the use of the study participant's data (or part of it) in any of the analysis populations (Section 5.2). Data will be used up to the withdrawal of consent.
2. Study participants (mothers or neonates/infants) with protocol violations as defined in Section 5.2.2, in relation to the use of the study participant's data (or part of it) in the per protocol population (Section 5.2.2).
3. Study participant's (mothers and neonates/infants) inclusion or exclusion status with regard to each analysis population (Section 5.2) guided by items 1-2 listed earlier.
4. Study participant's (mothers and neonates/infants) as-randomised vs as-treated (e.g. randomised to standard-of-care but received IV iron).
5. Auxiliary variables to be included in the additional analyses outlined in Section 5.4.

After database lock, the randomized treatment allocation will be obtained from the independent statistician who generated the randomisation code. No database may be locked, randomised treatment unblinded, or analyses completed until the SAP has been approved (in case of updates since its initial approval). The planned analyses in this SAP will be conducted after unblinding of the database and any changes to this SAP after unblinding will be documented in an amendment of this SAP and considered post-hoc analyses. The trial statistician will add the laboratory data of maternal ferritin, phosphate, C-reactive protein from Meander Medical Centre laboratory, accreditation number M040, EN ISO 15189:2012 (Amersfoort, The Netherlands) and Melbourne Health Shared Pathology Service, Royal Melbourne Hospital, accreditation number 2448, ISO 15189:2022 (Melbourne, Victoria, Australia) to the study database after unblinding.

5.2 Analysis Populations

Participating mothers and their neonates/infants will be reported and analysed according to their randomised study treatment ("as-randomised") for 5.2.1 and 5.2.2 and according to their actual study treatment ("as-treated") for 5.2.3. One randomised woman was found to have never been pregnant and was immediately withdrawn from the trial upon discovery during the first post-randomisation visit (i.e., Visit 4 at 36 weeks gestation) as further data collection was deemed not applicable. Following guidance on handling randomisation errors (17), the participant will be excluded from all analyses given the reasons for exclusion are independent of treatment group assignment and primary study outcome. The participant's information regarding the intervention administered or received between randomisation and Visit 4 together with related safety data will be reported. The following analysis populations are planned.

5.2.1 Intention-to-Treat Population

5.2.1.1 Mothers

This will consist of all women who were randomised, excluding those who have withdrawn informed consent for the use of all their data. This population will be used in the analysis of the maternal effectiveness study variables.

5.2.1.2 Neonates/infants

This will consist of all neonates/infants born to mothers who were randomised, excluding those who were stillborn (except for the stillbirth outcome) and excluding those who have withdrawn informed consent for the use of all their data. This population will be used in the analysis of the infant effectiveness study variables.

5.2.2 Per Protocol Population

5.2.2.1 Mothers

This will consist of all women who were randomised, and without protocol violations. A protocol violation is defined as those who have withdrawn informed consent for the use of all their data or violating in/exclusion criteria (e.g., twin pregnancy). These protocol violations are based on pre-randomisation characteristics only and expected to be balanced between treatment groups. Mothers who are found to be non-adherent to treatment (e.g., refused or not provided treatment after randomisation) will be excluded from the per-protocol (PP) population analysis. This population will be used in the analysis of the maternal effectiveness study variables.

5.2.2.2 Neonates/infants

This will consist of all neonates/infants born to mothers who were randomised, excluding those who were stillborn (except for the stillbirth outcome) and without protocol violations. A protocol violation is defined as those who have withdrawn informed consent for the use of all their data or the mother being a protocol violation. Neonates/infants of mothers who are found to be non-adherent to treatment (e.g., refused or not provided treatment after randomisation) will be excluded from the per-protocol (PP) population analysis. This population will be used in the analysis of the infant effectiveness study variables.

5.2.3 Safety Population

5.2.3.1 Mothers

This will consist of all women who received at least one study treatment (either IV iron or standard-of-care), excluding study participants who have withdrawn informed consent for use of all their data. This population will be used in the analysis of the maternal safety study variables.

5.2.3.2 Neonates/infants

This will consist of all neonates/infants born to mothers who received at least one study treatment (either IV iron or standard-of-care), excluding study participants who have withdrawn informed consent for use of all their data. This population will be used in the analysis of the infant safety study variables.

5.2.4 Modified Safety Population

5.2.4.1 Mothers

This will consist of all women who received at least one study treatment (either IV iron or standard-of-care), and without a protocol violation, excluding study participants who have withdrawn informed consent for use of all their data. This population will be used in the analysis of the maternal safety study variables.

5.2.4.2 Neonates/infants

This will consist of all neonates/infants born to mothers who received at least one study treatment (either IV iron or standard-of-care), and without a protocol violation, excluding study participants who have withdrawn informed consent for use of all their data. This population will be used in the analysis of the infant safety study variables.

5.3 Covariates and Subgroups

5.3.1 Covariates

The analysis model for all maternal and neonate/infant outcomes will be adjusted for the randomisation stratification variable of site as a main effect. This model will be referred to as the unadjusted model.

In addition, adjusted analysis models will be fitted to some of the effectiveness outcomes as specified in Section 7, separately for mothers and neonates/infants.

Of note, participants with missing data on one or more covariates in the adjusted model will be excluded from the adjusted analysis.

5.3.1.1 Maternal Covariates

1. First adjusted model: Adding to the unadjusted model demographic and/or baseline covariates as main affects assumed to be (strongly) prognostic or predictive of maternal anaemia. These covariates are: gravidity (primigravida vs. multigravida).
2. Second adjusted model: Adding to the first adjusted model laboratory biomarkers as main effects assumed to be (strongly) prognostic or predictive of maternal anaemia. These covariates are: inflammation status at baseline, iron deficient status at baseline, haemoglobin at baseline (continuous), and HIV positive status at baseline.
3. Third adjusted model: Given the large sample size, imbalance in demographic and/or baseline characteristics are not expected. However, in case of unexpected imbalance a third adjusted model will be fitted by adding those covariates identified after unblinding as main effects to the second adjusted model. After unblinding, no imbalances not yet accounted for were present.

5.3.1.2 Neonate/Infant Covariates

1. First adjusted model: Adding to the unadjusted model demographic and/or baseline covariates as main effects assumed to be (strongly) prognostic or predictive of birth weight. These covariates are: sex (female or male).
2. Second adjusted model: Adding to the first adjusted model laboratory biomarkers as main effects assume to be (strongly) prognostic or predictive of birth weight. These covariates are: maternal haemoglobin at baseline (continuous).
3. Third adjusted model: Given the large sample size, we do not expect an imbalance in demographic and/or baseline characteristics. However, a third adjusted model will be fitted in case of unexpected imbalance by adding those covariates

identified after unblinding as main effects to the second adjusted model. After unblinding, baseline maternal iron deficient status was imbalanced between groups and accounted for in the third adjusted model.

5.3.2 Subgroups

Exploratory subgroup analyses will be performed for the outcomes of maternal anaemia at 36 weeks' gestation or delivery (whichever comes first), haemoglobin concentration at 36 weeks' gestation or delivery (whichever comes first), birth weight, and low birth weight for the ITT population. The six subgroups' analyses listed below will be performed irrespective of whether the study's primary objective based on maternal anaemia at 36 weeks' gestation or delivery (whichever comes first) was achieved. Site was not considered a subgroup of clinical interest.

1. Gravidity (primigravida vs. multigravida)
2. Baseline HIV status (positive vs negative)
3. ~~Baseline severe anaemia status (yes vs no severe anaemia)~~
4. Baseline iron deficient status (yes vs no ID)
5. Baseline iron deficient anaemia status (yes vs no IDA)
6. Baseline inflammation status (yes vs no elevated CRP)

We hypothesise that mothers with severe anaemia, ID, or IDA at baseline will have a larger treatment effect compared with those who are non-severe anaemic, non-iron deficient, or non-iron deficient anaemic, respectively.

Subgroup (main effect) and the subgroup-by-treatment interactions term will be added to the unadjusted model to evaluate whether the treatment effect (IV iron versus standard-of-care) differs between subgroup categories. Results of the subgroup analyses will be displayed using Forest plots.

Of note, participants with missing data on a subgroup will be excluded from the subgroup analysis.

5.4 Missing Data

5.4.1 Maternal outcomes

To describe the missing data, the frequency and percentage of study participants with missing data at baseline, 36 weeks' gestation or delivery (whichever comes first),

delivery and one month postpartum will be summarised by treatment group using the ITT population. In addition, baseline and demographic characteristics will be summarised by those with baseline only, incomplete data at any visit, and complete data at all visits for anaemia (mothers) to explore the missing data assumption(s) and identify any study variables not included in the target analyses that are potentially associated with missing/not missing of these study variables (known as auxiliary variables).

As the primary strategy to handle missing data for the primary maternal outcome, the analysis of maternal anaemia will use a likelihood-based approach; detailed in Section 7.2.1. This approach relies on the underlying assumption that the probability of missing outcome data is not related to the missing data after conditioning on observed data in the model (Missing at Random [MAR]). An additional analysis will be performed separately by treatment group whereby missing maternal haemoglobin data will be multiply imputed using chained equations. The imputation model will include site, visit (categorical), and all variables listed in the second adjusted model described in Section 5.3.1.1. During the blinded data review, no auxiliary variables were identified in addition to the variables already part of the imputation model. Maternal haemoglobin will be imputed using a linear regression model. The missing outcome data at baseline, 36 weeks' gestation or delivery (whichever comes first), delivery and one month postpartum will be imputed using the "just another variable" approach (also known as imputing in wide format) which requires a separate imputation model for imputing the variable at each assessment time (18). The number of imputed data sets will be greater than or equal to the percentage of missing data in the available case analyses. Using these imputed data sets, an analysis based on a pattern-mixture model (19) consisting of applying a delta-adjustment to the imputed values by treatment group will be conducted. Delta-adjustment values represent the average difference in the mean maternal haemoglobin value for those with and without missing data on haemoglobin, conditional on all analysis variables. Within the standard-of-care group participants with missing data will be assumed having both a poorer and better response than those with observed data. No *a priori* difference is anticipated in the mean response for the IV iron group. Differences in baseline participant characteristics between those with and without data will inform delta-values to explore in both treatment groups. Based on the baseline participant characteristics, a delta-adjustment of -2g/dL to +2g/dL (in steps of 0.5g/dL) will be applied to the imputed values of both the standard-of-care (oral iron) and IV iron

groups. Using these values, the delta-adjusted approach will shift imputed haemoglobin values by 'delta'. After deriving maternal anaemia from the delta-adjusted imputed haemoglobin values, the imputed data sets will be analyzed using the model described in Section 7.2.1. The estimates from the analyses of the imputed data sets will be combined to obtain a pooled common estimate and corresponding confidence interval for the effect of the iron intervention on maternal anaemia using Rubin's rules. The delta-adjustment method within the multiple imputation framework assumes a Missing Not At Random (MNAR) assumption for the outcome, maternal anaemia.

Key secondary and other secondary maternal outcomes will use a likelihood-based approach for handling missing data; detailed in Section 7.2.2.

5.4.2 Neonate/Infant Outcomes

To describe the missing data, the frequency and percentage of study participants with missing data at birth will be summarised by treatment group using the ITT population (excluding those undefined due to stillbirth). In addition, baseline and demographic characteristics will be summarised by those with data at birth to explore the missing data assumption(s) and identify any study variables not included in the target analyses that are potentially associated with missing/not missing of these study variables (known as auxiliary variables).

As the primary strategy to handle missing data for the key neonatal outcome, the analysis of birth weight will use a multiple imputation approach. This approach relies on the MAR assumption.

For cases where birth weight is undetermined (e.g., drop-out or lost-to-follow-up prior to Visit 4 [36 weeks' gestation] and with unknown delivery outcome, or known live-born infant but missing birth weight), missing birth weight data will be multiply imputed, separately by treatment group. The imputation model will include site and maternal haemoglobin at baseline (continuous). Infant sex (female or male) will not be included because it is missing for those with unknown delivery outcome. During the blinded data review, gravidity (primigravida vs. multigravida) and maternal HIV positive status at baseline were identified as auxiliary variables. For the imputed cases, singleton birth will be assumed (given confirmed singleton pregnancy in the third trimester is an inclusion criteria) and the imputation model will be fitted by restricting the data to singleton births only as recommended by Sullivan et al. (20).

Of note, all anthropometry measures at birth are collected for infants from multiple births. Birth weight will be imputed using a linear regression model. The number of imputed data sets will be greater than or equal to the percentage of missing data in the complete case analyses. The estimates from the analyses of the imputed data sets will be combined to obtain a pooled common estimate and corresponding confidence interval for the effect of the iron intervention on birth weight using Rubin's rules. Outcome data known to be undefined because of stillbirth will not be imputed. Analysis of birth weight is further detailed in Section 7.3.1.

A similar approach will be used for birth length. Low birth weight will be derived from the multiple imputed birth weight values, after which the imputed data sets will be analysed and combined as described above. The outcome stillbirth will not be imputed and thus will be reported among those with a known delivery outcome (i.e., complete cases). Key secondary and other neonatal outcomes collected at one month of age will not be imputed. Analyses for key secondary and other neonatal outcomes are further detailed in Section 7.3.2.

5.5 Interim Analyses and Data Monitoring

An independent Data Monitoring Committee (DMC) was constituted and provided oversight of the study. The DMC is comprised of three international experts in clinical trials, obstetrics, epidemiology and statistics. The DMC recommends to the sponsor and investigators whether to continue, modify or terminate the trial on ethical grounds. Interim analyses to stop the trial early were neither planned nor conducted. A sample size re-estimation was conducted (see section 4).

A total of 3 DMC meetings were held before unblinding the randomised treatment allocation. The recommendation was to continue the study as planned without any modification.

Meeting	Date	Outcome
1st meeting (open+closed)	17 August 2021	Initial meeting
2nd meeting (open+closed)	29 November 2022	Continue as planned
3rd meeting (closed only)*	11 January 2023	Continue as planned

* During this meeting, the DMC evaluated the results of the sample size re-estimation (see sample size estimation and power calculation section 4) and recommended that no sample size change was necessary.

5.6 Multi-centre Studies

The trial is based at the Training and Research Unit of Excellence (TRUE) center at Zomba Central Hospital in Southern Malawi. The trial has eight government health centers across Zomba district available for screening, recruitment and provision of the intravenous drug as part of REVAMP-TT, namely: Likangala, Bimbi, Lambulira, Domasi, Naisi, Matawale, Sadzi and City clinic. These health centres are within a 30km radius from Zomba.

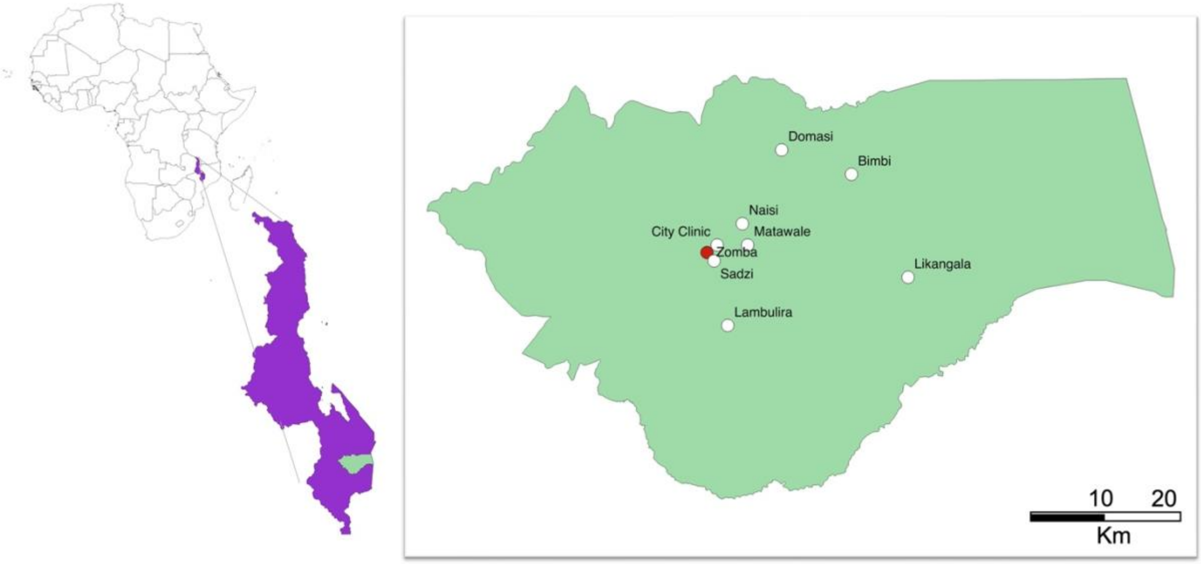

Figure 2 Map of Malawi with location of Zomba district, the coordinating trial site in Zomba (red) and the recruiting government primary health centres (white). Figure prepared in QGIS2.10.

5.7 Multiple Testing

We ordered study variables by degree of importance. The following comparison and hypothesis is defined for the effectiveness study variables:

H0: There is no difference between IV iron and Standard-of-care

H1: There is a difference between IV iron and Standard-of-care

For the primary maternal endpoint (anaemia at 36 weeks' gestation or delivery, whichever comes first) and key neonate endpoint (birth weight), no adjustment for

multiplicity is planned. The above hypothesis will be tested at a two-sided 5% significance level for each endpoint separately.

The above hypothesis will be tested using the Holm procedure (21) to control the Type I error rate for following set of maternal and neonate/infant outcomes separately:

Maternal:

- Haemoglobin concentration at 36 weeks' gestation or delivery (whichever comes first)
- Serum ferritin concentration at 36 weeks' gestation or delivery (whichever comes first)
- Haemoglobin concentration at 1 month postpartum
- Serum ferritin concentration at 1 month postpartum

Neonate/infant:

- Haemoglobin concentration at 1 month of age
- Weight at 1 month of age
- ~~Serum ferritin concentration at 1 month of age~~

We will report multiplicity unadjusted P-values along with the estimate and 95% confidence intervals and footnote the comparisons meeting the statistical significance threshold according to the Holm procedure. Specifically, the Holm procedure is a stepwise multiple test method that controls the probability of one or more type 1 errors to be at most 5%, whereby null hypotheses are tested step-down using adjusted individual significance levels until no further rejections can be made.

For effectiveness endpoints/study variables not listed above, we will present the estimate and two-sided 95% confidence intervals (no P-Values). No adjustment is planned. This includes the components of the composite several medical event and adverse birth outcome.

No multiplicity adjustment is planned for the safety endpoints/study variables, P-Values will be presented.

No multiplicity adjustment is planned for the subgroup analyses (Section 5.3.2) because they are considered exploratory, P-Values will be presented.

6 Summary of Study Data

6.1 Subject Disposition

The flow of study participants will be presented in a Consolidated Standards of Reporting Trials (CONSORT) diagram. The diagram will include the number of study participants within the following categories:

Pre-Screening	• Pregnant women assessed for eligibility<br>• Women excluded<br>◦ Did not meet eligibility criteria (reasons)*
Screening & Enrolment	• Women excluded<br>◦ Did not meet eligibility criteria (reasons)*<br>◦ Declined to participate†<br>◦ Other reasons
Allocation	• Randomised IV iron (plus IPTp) or oral iron (plus IPTp)<br>• Received allocated intervention‡<br>• Did not receive allocated intervention (reasons)<br>• Did not receive IPTp<br>• For IV iron only: Discontinued IV
36 weeks' gestation	• Assessed for maternal Hb at 36 weeks' gestation, if not:<br>◦ Lost-to follow-up (reasons)<br>◦ Discontinued (reasons)<br>• Attended the visit<br>• Not attended the visit
Delivery	• Assessed for maternal Hb at delivery, if not:<br>◦ Lost-to follow-up (reasons)<br>◦ Discontinued (reasons)<br>• Assessed for birth weight at delivery, if not:<br>◦ Lost-to follow-up (reasons)<br>◦ Discontinued (reasons)<br>• Mothers:<br>◦ Attended the visit<br>◦ Not attended the visit<br>• No. of deliveries:

	• Stillbirth<br>• Neonatal deaths
Day 28 postpartum	• Assessed for maternal Hb at 28 days postpartum, if not:<br>• Lost-to follow-up (reasons)<br>• Discontinued (reasons)<br>• Mothers:<br>• Attended the visit<br>• Not attended the visit<br>• Assessed for child's Hb at 28 days postpartum, if not:<br>• Lost-to follow-up (reasons)<br>• Discontinued (reasons)<br>• Children:<br>• Attended the visit<br>• Not attended the visit
Analysis	• Mothers analysed<br>• Excluded from analysis (reasons)<br>• Neonates/infants analysed<br>• Excluded from analysis (reasons)

*Reasons for not meeting eligibility were assessed on the questions on the eligibility data collection form.

†Defined as those who answered No to the question "Accepts study procedures?" at enrolment.

‡Reasons for not receiving or not provided with the treatment were collected on the participant randomisation form.

6.2 Demographic and Baseline Variables

Demographic and baseline variables will be summarised for the ITT and PP population and presented by treatment group in the order of (IV iron, standard-of-care) using frequencies and percentages (based on the non-missing sample size) for categorical variables, mean and standard deviation for continuous variables, or median and quartiles (25th and 75th percentile) for non-symmetrical continuous variables. If variables have missing values, we will report the non-missing sample size either in

the table or in the footnote of the table. No P-Values will be reported to compare treatment arms.

We will present the following variables:

- General: site, age (years) at enrolment*, primigravid, gestational age (in weeks)**, religion, maternal education (education level), marital status, income source.
- Maternal anthropometry: maternal height (cm), maternal weight (kg), maternal BMI (kg/m^2).
- Maternal laboratory indices: HIV positive, malaria RDT positive at enrolment, capillary haemoglobin < 10.0 g/dL, venous haemoglobin, (venous) anaemia (mild, moderate, severe), ferritin (ug/L), iron deficient, iron deficient anaemia, C-reactive protein (µg/L), inflammation, (venous) anaemia and inflammation.

*derived using the date of randomisation visit and maternal date of birth
**dated either using Last Menstrual Period or fundal height, the authors note this limits the accuracy of gestational age assessment

6.3 Treatment Compliance

All randomised women were planned to be provided with SP as IPTp according to national guidelines, those who received at least one dose or with contraindications will be reported as part of the CONSORT diagram.

In addition:

- FCM/IV iron group: After randomisation, all participants in the IV iron arm had FCM administered by the field team unless the participant refused the treatment. Discontinuation of IV iron during administration was recorded. Information on IV administration, refusal, and discontinuation will be reported as part of CONSORT diagram.
- Standard-of-care/oral iron group: After randomisation, all participants in the oral iron arm were planned to be provided with oral iron to be taken twice daily for the reminder of pregnancy. Information on distribution of oral iron will be reported as part of the CONSORT diagram.

7 Effectiveness Analyses

For all effectiveness analyses, we will use the following principles:

- We will use the ITT population for all effectiveness analyses. In addition, we will use the PP population for selected study variables.
- Comparisons of interest are IV iron versus standard-of-care at 36 weeks' gestation or delivery (whichever comes first), delivery, and 1-month postpartum, whereby the maternal primary endpoint is 36 weeks' gestation or delivery (whichever comes first), and the neonate key endpoint is delivery.
- Confidence intervals and P-Values will be presented following the multiple testing strategies described in Section 5.6.
- All analyses will be adjusted for the stratification variable used during the randomisation (site), referred to as the unadjusted analyses.
- Missing data will be handled as described in Section 5.4.

7.1 Primary Estimand

The estimands for REVAMP-TT have been defined according to the addendum to the ICH E9 on estimands and sensitivity analyses in clinical trials (22). Each primary maternal and key neonate objective has a corresponding primary estimand for the treatment comparison of interest. In total, two primary estimands are defined and presented below in estimand-to-analysis tables (Table 4 and Table 5).

Table 4 Estimand-to-analysis table for the primary maternal estimand

**Objective**: To determine whether a single dose of FCM up to 1000mg is superior to standard-of-care (i.e., oral iron provided through local health services) in reducing maternal anaemia (defined as a venous haemoglobin concentration less than 11.0g/dL) at 36 weeks' gestation or at delivery, whichever comes first in Malawian women in the third trimester of pregnancy with moderate or severe anaemia.
**Estimand**: To demonstrate superiority of a single dose of FCM up to 1000mg compared with standard-of-care (i.e., oral iron provided through local health services), based on the prevalence of anaemia (defined as a venous haemoglobin concentration less than 11.0g/dL) at 36 weeks' gestation or at delivery, whichever comes first in Malawian women in the third trimester of pregnancy with moderate or severe anaemia, regardless of stillbirth, (serious) adverse events, hospitalisation, ICU admission, or blood transfusion.

**Treatment:** IV Iron (FCM)	
**Comparator:** Standard-of-care (oral iron)	
**Estimand**	**Analysis**
**Target population:** Malawian women in the third trimester of pregnancy with moderate or severe anaemia. Full details of the inclusion and exclusion criteria are provided in section 3.2.	**Analysis set:** All randomized participants excluding those randomised in error
**Variable:** Maternal anaemia, defined as a venous haemoglobin concentration less than 11.0g/dL~~, assessed~~ at 36 weeks' gestation or delivery~~, whichever came first~~, less than 12.0g/dL at 28 days postpartum.	**Outcome measure:** prevalence ~~ratio~~ of anaemia at 36 weeks' gestation or delivery, whichever came first.
**Handling of intercurrent events:**<br><br>Birth/delivery: composite strategy<br><br>Stillbirth*: treatment policy strategy<br><br>(Serious) adverse event: treatment policy strategy<br><br>Hospitalization and/or ICU admission: treatment policy strategy<br><br>Blood transfusion: treatment policy strategy	**Handling of missing data:** Missing data (e.g., as a result of withdrawal or loss to follow up for reasons not related to intercurrent events) will be assumed to be Missing at Random (MAR).
**Population-level summary measure:** prevalence ratio of IV iron versus standard-of-care (oral iron)	**Analysis approach:** longitudinal regression model. Full details are provided in section 7.2.1.

*Stillbirth occurred in ~1% of the study population.

Table 5 Estimand-to-analysis table for the key neonatal estimand

**Objective:** To determine the effects of a single dose of FCM up to 1000mg compared to standard-of-care (i.e., oral iron provided through local health services) on neonatal birth weight within 24 hours of birth in live-born children born of Malawian women in the third trimester of pregnancy with moderate or severe anaemia.	
**Estimand:** To demonstrate superiority of a single dose of FCM up to 1000mg compared with standard-of-care (i.e., oral iron provided through local health services), based on the average birth weight within 24 hours of birth, in live-born children born of Malawian women in the third trimester of pregnancy with moderate or severe anaemia, regardless of (serious) adverse events in mother or child, maternal hospitalisation, maternal ICU admission, or maternal blood transfusion..	
**Treatment:** IV Iron (FCM)	
**Comparator:** Standard-of-care (oral iron)	
**Estimand**	**Analysis**
**Target population:** Live-born children born of Malawian women in the third trimester of pregnancy with moderate or severe anaemia. Full details of the inclusion and exclusion criteria are provided in section 3.2.	**Analysis set:** Children of all randomized mothers (except those stillborn*)
**Variable:** birth weight (in grams) within 24 hours of birth	**Outcome measure:** ~~absolute difference in~~ mean birth weight
**Handling of intercurrent events:**<br><br>Neonate (Serious) adverse event: treatment policy strategy<br><br>Maternal (Serious) adverse event: treatment policy strategy<br><br>Maternal hospitalization and/or ICU admission: treatment policy strategy	**Handling of missing data:** Missing data (e.g., as a result of withdrawal or loss to follow up for reasons not related to intercurrent events will be assumed to be Missing at Random (MAR).

Maternal blood transfusion: treatment policy strategy	
**Population-level summary measure:** Mean difference in birth weight between IV iron and stand-of-care (oral iron)	**Analysis approach**: linear regression model. Full details are provided in section 7.3.1.

*Stillbirth occurred in ~1% of the study population.

7.2 Maternal Study Variables

7.2.1 Maternal Anaemia

Maternal anaemia (primary outcome) at 36 weeks' gestation or delivery, whichever comes first (primary timepoint), delivery, and 28 days postpartum will be analysed for the ITT population using a log-binomial regression model (i.e., log-link function and binomial distribution), including study participants (mothers) as a random intercept to account for the multiple time points. The model will include a treatment and treatment by timepoint interaction and adjust for the stratification variable used during the randomization (site). The model will include the standard-of-care (oral iron) group as the reference group. We will report the estimate of the prevalence ratio of IV iron versus standard-of-care (oral iron), two-sided 95% confidence interval extracted at the primary time point and P-Value. From this same model we will also extract the treatment effect at delivery and 28 days postpartum.

$$\log\{\pi_{ij}\} = \beta_0 + \beta_1 treatment_i + \beta_2 time_j + \beta_3 treatment_i \times time_j + \beta_4 site_i + \mu_i$$

Where, $\pi_{ij} = P(y_{ij} = 1|treatment_i, time_j, site_i, \mu_i)$ represents the probability of maternal anaemia for participant $i = 1, \dots n$ at time j, $y_{ij}|\pi_{ij} \sim Binomial(1, \pi_{ij}), treatment_i$ has values IV iron and standard-of-care (i.e., reference level), $time_j$ has values 36 weeks' gestation or delivery, delivery, and 28 days postpartum, and random intercept $\mu_i \sim N(0, \sigma_u^2)$.

The treatment effect will be estimated from this model as the prevalence ratio of IV iron versus standard-of-care (oral iron). We have selected the prevalence ratio as an effect measure because its interpretation as the ratio change in prevalence is easier to understand than the interpretation of the odds ratio for clinical researchers.

In case of non-convergence (23), we will fit a binomial regression model with logit or complementary log-log (if appropriate) link, including study participants (mothers) as a random intercept to account for the multiple time points. An estimate of the prevalence ratio will be obtained using margins with confidence intervals calculated by the delta-method.

In addition to the analysis described above, we will perform:

- The three adjusted analyses on the ITT population described in Section 5.3.1.1. Of note, the models will include only those participants with non-missing covariate information.
- The missing data additional analyses on the ITT population described in Section 5.4.1
- The model described above on the PP population
- The model described above on the PP population adjusted for baseline characteristics considered not balanced between the arms for the PP population
- The model described above fitted to 36 weeks' gestation, delivery, and 28 days postpartum to obtain the treatment effect at 36 weeks' gestation on the ITT and PP population.

Furthermore, we will report the number-needed-to-treat (NNT) and 95% confidence interval for maternal anaemia at 36 weeks' gestation or delivery (whichever comes first), with the confidence interval only if the treatment effect is statistically significant (24).

7.2.2 Secondary Maternal Analyses

The repeated time point continuous outcomes haemoglobin concentration and ferritin concentration will be analysed using a likelihood-based longitudinal data analysis model for the ITT population (25). The outcome (dependent variable) will consist of the baseline, 36 weeks' gestation or delivery (whichever came first), delivery, and 28 days postpartum values. The model will assume a common baseline mean across the two treatment groups due to random allocation. It will incorporate time point (study visit) as a categorical variable; thus it will not assume a specific trajectory over time. In addition to study visit, the model will include the stratification factor (site) and treatment as main factors and the treatment by study visit interaction. The variance-covariance among the repeated measurements will be

defined as unstructured. In case of non-convergence, we will consider alternative structures (first-order autoregressive, Toeplitz, compound symmetry). Using this model, study participants with a missing baseline or a missing post-baseline outcome will be included in the analysis but study participants with no outcome values will be excluded. We will obtain the estimate and two-sided 95% confidence interval of the mean difference in change from baseline to postbaseline between IV iron and standard-of-care (oral iron) and P-Values, whereby 36 weeks' gestation or delivery (whichever comes first) is the primary timepoint. Furthermore, we will display the estimate and 95% confidence interval of haemoglobin and anaemia over time in a Figure. Ferritin will be log base e transformed before fitting the model due to skewness and as a result we will report the treatment effect as a geometric mean ratio.

The repeated time point binary outcomes ID and IDA will be analysed similarly to anaemia for the ITT population. We will extract the treatment effect from this same model at 36 weeks' gestation or delivery (whichever came first), delivery, and 28 days postpartum.

In addition to the analysis described above, we will perform:

- The three adjusted analyses on the ITT population described in Section 5.3.1.1
- The model described above on the PP population
- The model described above on the PP population adjusted for baseline characteristics considered not balanced between the arms for the PP population

7.3 Neonate/Infant Study Variables

7.3.1 Birth weight

Birth weight will be analysed by fitting a linear regression model, adjusting for the stratification variable used during the randomization (site), for the ITT population (excluding those stillborn). We will examine the assumptions underlying the model, including normally distributed error terms, homoscedasticity, and influential observations. We will report the absolute difference in mean birth weight between IV iron and standard-of-care (oral iron) along with a corresponding two-sided 95% confidence interval and P-Value. Missing birth weight data will be handled as described in Section 5.4.2.

In addition to the analysis described above, we will perform:

- An analysis on those known to be liveborn in the ITT population (i.e., complete cases under the missing completely at random [MCAR] assumption)
- The three adjusted analyses on the ITT population described in Section 5.3.1.2. Of note, due to missing sex among those with unknown pregnancy outcome, the models will include only those known to be liveborn (i.e., complete cases).
- The model described above on the PP population
- The model described above on the PP population adjusted for baseline characteristics considered not balanced between the arms for the PP population
- An analysis consisting of a linear generalised estimating equation (GEE) model with either an exchangeable or independence (with robust variance estimation) working correlation structure to account for clustering of twin pairs on those with known singleton status (i.e., those known to be liveborn), given multiple births occurred in ~1% of the maternal ITT population (26).

7.3.2 Secondary Neonate/Infant Analyses

Birth length will be analysed similarly to birth weight for the ITT population (excluding stillbirths). The single time point continuous outcomes haemoglobin concentration~~,~~ ~~ferritin concentration~~, weight, length, length-for-age z-score, weight-for-age z-score, and weight-for-length z-score at one-month of age will be analysed similarly to birth weight for the ITT population (complete cases). Appropriate transformations may be applied to the variables before fitting the model if considered skewed. Missing data will be handled as described in Section 5.4.2.

The single time point binary outcomes of low birth weight for the ITT population (excluding stillbirths) and stillbirth for the ITT population (excluding those with unknown delivery outcome) will be analysed using a log-binomial regression model, adjusting for the stratification variable used during the randomization (site) (not for stillbirth due to data separation issues). A sensitivity analysis consisting of Firth logistic regression including site was performed for stillbirth. We will report the estimate of the risk ratio of IV iron versus standard-of-care (oral iron), two-sided 95% confidence interval and P-Value. In case of non-convergence, we will fit a binomial regression model with logit or complementary log-log (if applicable) link and an estimate of the risk ratio will be obtained using margins with confidence

intervals calculated by the delta-method. Missing data will be handled as described in Section 5.4.2.

In addition to the analysis described above, we will perform:

- An analysis on those known to be liveborn on the ITT population for birth length and low birth weight (i.e., complete cases under the MCAR assumption)
- The three adjusted analyses on the ITT population described in Section 5.3.1.2. Of note, due to missing sex among those with unknown pregnancy outcome, the models will include only those known to be liveborn (i.e., complete cases).
- The model described above on the PP population
- The model described above on the PP population adjusted for baseline characteristics considered not balanced between the arms for the PP population

Furthermore, we will report the number-needed-to-treat (NNT) and 95% confidence interval for low birth weight, with the confidence interval only if the treatment effect is statistically significant (24).

8 Safety Analyses

For all safety analyses, we will use the following principles:

- We will use the safety and modified safety populations for all safety analyses
- Comparisons of interest are IV iron versus standard-of-care (oral iron)
- All tests will be two-sided at the 5% level of significance
- All confidence intervals will be two-sided 95%
- All analyses will be adjusted for the stratification variable used during the randomisation (site), referred to as the unadjusted analyses, except for maternal and neonate adverse events due to data separation issues.
- A complete case analysis will be used for all outcomes.

8.1 (Serious) Adverse Events

(Serious) adverse event (SAEs) were collected at any time after randomisation.

Adverse events (AEs) will be coded using version 5.0 of the Common Terminology Criteria for Adverse Events (CTCAE) (US Department of Health Human Services, 27 November 2020) and use an appropriate Preferred Term (PT) and System Organ Class (SOC) for each AE verbatim term. AEs will be coded as one AE per presentation, unless

clearly unrelated. We will report the most 'upstream' condition: Stillbirth (pregnancy loss after 22 weeks and prior to delivery with no evidence of life at delivery) and neonatal death (death in the first 28 days of life of a child with evidence of life following delivery). Where the underlying cause is not discernible, AEs will be coded as pregnancy loss and neonatal death where appropriate. All AEs will be coded on an ongoing basis by two clinicians – blinded to each other's decisions – and without knowledge of the treatment-code. Discrepancies regarding the selection of an appropriate PT or SOC will be resolved via a discussion between the two clinicians. All AE coding will be finalised before unblinding of the study database.

8.1.1 Maternal

We will report the following for women:

- n (%) of women with at least one AE (overall)
- n (%) of women with at least one SAE (overall)
- n (%) of women who died
- number of AE
- number of SAE
- n (%) of women for the composite severe medical events including its components haemorrhage, need for transfusion, ICU admission, or mortality
- n (%) of women with clinical malaria (as per preferred term)
- n (%) of women with at least one common AE (>5% in any group)
- n (%) of women with at least one AE by system organ class
- n (%) of women with at least one AE by preferred term

Except for number of AE/SAE, each of the above listed adverse event outcomes will be compared between treatment arms using a log-binomial regression model. We will report the estimate of the risk ratio of IV iron versus standard-of-care (oral iron), two-sided 95% confidence interval and P-Value. In case of non-convergence, we will fit a binomial regression model with a logit or complementary log-log (if applicable) link and an estimate of the risk ratio will be obtained using margins with confidence intervals calculated by the delta-method.

In addition, we will report infusion-related AEs separately for the IV iron group only:

- n (%) of women with at least one treatment-related AE

- n (%) of women with headache, dizziness, discolouration of the skin, nausea, vomiting, upper abdominal pain, dyspepsia, flushing, shortness of breath, chest pains, anaphylactic shock, other

8.1.2 Neonates/Infants

We will report the following for neonates/infants:

- n (%) of neonates/infants with at least one AE
- n (%) of neonates/infants with at least one SAE
- n (%) of neonates/infants who died
- n (%) of neonates/infants who were stillborn or died
- number of AE
- number of SAE
- n (%) of neonates/infants with clinical malaria (as per preferred term)
- n (%) of neonates/infants with at least on common AE (>5% in any group)
- n (%) of neonates/infants with at least one AE by system organ class
- n (%) of neonates/infants with at least one AE by preferred term

Except for number of AE/SAE, these outcomes will be analysed using a log-binomial regression model. In case of non-convergence, we will fit a binomial model with a logit or complementary log-log (if applicable) link and an estimate of the risk ratio will be obtained using margins with confidence intervals calculated by the delta-method. Prevalence ratios and 95% confidence intervals will be presented for IV iron versus standard-of-care (oral iron).

8.2 Safety biomarkers/Clinical Laboratory Evaluations

8.2.1 Maternal

Hypophosphatemia will be derived from phosphate (PO4) (27, 28) as:

- Any: PO4 <0.80 mmol/L
    - Mild: 0.64 mmol/L < PO4 <0.80 mmol/L
    - Moderate: 0.32 mmol/L < PO4 <0.64 mmol/L
    - Severe: PO4 < 0.32 mmol/L

Hypophosphatemia, and inflammation will be analysed by time-point (36 weeks' gestation or delivery (whichever came first), delivery, and 28 days postpartum) using a log-binomial regression model. We will report the estimate of the risk ratio of IV

iron versus standard-of-care (oral iron), two-sided 95% confidence interval and P-Value. In case of non-convergence, we will fit a binomial regression model with a logit or complementary log-log (if applicable) link and an estimate of the risk ratio will be obtained using margins with confidence intervals calculated by the delta-method.

8.2.2 Neonates/infants

Hypophosphatemia will be derived from phosphate (PO4) (27, 28) as:

- Any: PO4 <0.80 mmol/L
    - Mild: 0.64 mmol/L < PO4 <0.80 mmol/L
    - Moderate: 0.32 mmol/L < PO4 <0.64 mmol/L
    - Severe: PO4 < 0.32 mmol/L

Hypophosphatemia will be analysed using a log-binomial regression model. We will report the estimate of the risk ratio of IV iron versus standard-of-care (oral iron), two-sided 95% confidence interval and P-Value. In case of non-convergence, we will fit a binomial regression model with a logit or complementary log-log link (if applicable) and an estimate of the risk ratio will be obtained using margins with confidence intervals calculated by the delta-method.

9 Technical Details

Analysis will be conducted using Stata/SE for Windows version 17.0 (64-bit x86-64) or higher. We will report the software and version used at the time of reporting.

10 Validation of Output

The analysis of the maternal anaemia and birth weight will be checked by a senior statistician. Discrepancies will be discussed and resolved by consensus. All other analyses will be conducted by the trial statistician only and reviewed by a senior statistician.

11 Summary of Changes to the Registry and Protocol

This statistical analysis plan outlined the details of the analyses beyond what was specified in the registry.

Compared to the registry, the following was changed:

- The alternative model in case of non-convergence of the log-binomial models was changed from a Poisson model with robust standard errors to a binomial model with either a logit or complementary log-log link. This change was implemented following advice of the DMC statistician.
- The analysis of the neonatal outcomes at birth was changed from complete cases to multiple imputation to handle non-negligible missing data. This change was implemented following advice of the DMC statistician.

Compared to the protocol (version 3.1), the following was changed:

- Clarified the description for the reporting and analysis of clinical malaria.
- Maternal and neonatal unplanned clinic visit analyses were omitted. The unplanned visit data will not provide any additional value over AE data already collected by the 24-hour cover of the study research sites. It was felt AE data provides the most complete capture of illnesses and symptoms experienced by women; it was recognised that some women may have presented for care at a non-study health centre, and this visit would not be captured in the unplanned visits outcome; however, these data are still captured as AEs occurring between fixed study visit timepoints.
- Study variables indicating premature birth (<37 weeks' gestation) or not, small for gestational age or not, and a composite outcome of adverse birth outcomes (presence of stillbirth, low birth weight, premature birth, or small for gestational age) will not be derived due to the inaccurate nature of the gestation duration.
- An additional analysis to explore the heterogeneity of the primary outcome has been added in response to a reviewer's comment from the Gates Open Research Publication.
- The alternative model in case of non-convergence of the log-binomial models was changed from a Poisson model with robust standard errors to a binomial model with either a logit or complementary log-log link. This change was implemented following advice of the DMC statistician.

12 Summary of Changes to the Statistical Analysis Plan

Compared to the Statistical Analysis Plan version 1.1, the following was known or changed [after unblinding]:

- Laboratory values only became available after unblinding. Phosphate values were set to missing in case of haemolysis due to the difficulty of clinically interpreting the measured value if this occurred. C-reactive protein values were not set to missing in this happened (Section 3.5.1).
- Some laboratory values came from Melbourne Health Shared Pathology Service, Royal Melbourne Hospital, accreditation number 2448, ISO 15189:2022 (Melbourne, Victoria, Australia. Both accredited laboratories use the same platforms. The neonate serum ferritin concentration and phosphate values were not available (Section 5.1).
- No pre-planned third adjusted model in case of unexpected imbalance between treatment groups in baseline characteristics was fitted to maternal efficacy outcomes (Section 5.3.1.1). A pre-planned third adjusted model was fitted to neonatal efficacy outcomes due to imbalance between the groups in maternal iron deficient status at baseline (Section 5.3.2.2).
- The pre-planned subgroup analysis model for severe anaemia could not be performed due to sparse data i.e., 23/569 (4%) (Section 5.3.2).
- Clarifications were added to the Estimand-to-analysis Tables 4 and 5. These clarifications did not affect the pre-planned analysis.
- The pre-planned model for stillbirth excluded the stratification factor (site) due to data separation issues (Section 7.3.2). A post-hoc sensitivity analysis was conducted using a Firth logistic regression model adjusted for site.
- In addition to stillbirth, several pre-planned models experienced data separation when including the stratification factor (site) in the model. As a result, site was not adjusted for in the analysis of maternal adverse events (Section 8.1.1) and neonate adverse events (Section 8.1.2).
- The 95% confidence interval was omitted for the NNT for low birth weight due to the treatment effect not being statistically significant (Section 7.3.2).

13 References

1. Source of SAP template: Cambridge University Hospitals – Clinical Trials Unit https://www.cuh.nhs.uk/document-library/cctu-standard-operating-procedures-sops.
2. World Health Organization. The global prevalence of anaemia in 2011. . Geneva: 2015.
3. Haider BA, Olofin I, Wang M, Spiegelman D, Ezzati M, Fawzi WW. Anaemia, prenatal iron use, and risk of adverse pregnancy outcomes: Systematic review and meta-analysis. BMJ (Online). 2013;347(7916). doi: 10.1136/bmj.f3443.
4. Pasricha SR, Tye-Din J, Muckenthaler MU, Swinkels DW. Iron deficiency. Lancet. 2021;397(10270):233-48. Epub 2020/12/08. doi: 10.1016/s0140-6736(20)32594-0. PubMed PMID: 33285139.
5. Premru-Srsen T, Verdenik I, Ponikvar BM, Steblovnik L, Geršak K, Cerar LK. Infant mortality and causes of death by birth weight for gestational age in non-malformed singleton infants: a 2002-2012 population-based study. J Perinat Med. 2018;46(5):547-53. Epub 2017/06/11. doi: 10.1515/jpm-2017-0103. PubMed PMID: 28599397.
6. World Health Organization, United Nations Children's Fund, United Nations University. Iron Deficiency Anaemia: Assessment, Prevention, and Control. A guide for programme managers. Geneva: 2001.
7. World Health Organization. Global Nutrition Targets 2025: Anaemia Policy Brief. . Geneva: 2014.
8. Low MSY, Speedy J, Styles CE, De-Regil LM, Pasricha SR. Daily iron supplementation for improving anaemia, iron status and health in menstruating women. Cochrane Database of Systematic Reviews. 2016;2016(4). doi: 10.1002/14651858.CD009747.pub2.
9. Medicinewise N. Ferinject - Ferric carboxymaltose. Consumer medicine information (CMI) leaflet: NPS Medicinewise; 2019 [cited 2019 14 Oct]. Available from: https://www.nps.org.au/medicine-finder/ferinject-solution-for-injection.
10. Mwangi MNM, G., Moya E, Braat S, Harding R, Robberstad B, Simpson JA, et al. Protocol for a multicentre, parallel-group, open-label randomised controlled trial comparing ferric carboxymaltose with the standard of care in anaemic Malawian pregnant women: the REVAMP trial. BMJ Open. 2021;0:e053288. doi: doi:10.1136/bmjopen-2021-053288.
11. Pasricha S-R, Mwangi MN, Moya E, Ataide R, Mzembe G, Harding R, et al. Ferric carboxymaltose versus standard-of-care oral iron to treat second-trimester anaemia in Malawian pregnant women: a randomised controlled trial. The Lancet. 2023;401(10388):1595-609. doi: 10.1016/S0140-6736(23)00278-7.
12. World Health Organization. Haemoglobin concentrations for the diagnosis of anaemia and assessment of severity. Vitamin and Mineral Nutrition Information

System. Geneva, World Health Organization, 2011 (WHO/NMH/NHD/MNM/11.1) 2011 [cited 2022 17 Feburary]. Available from: https://www.who.int/vmnis/indicators/haemoglobin.pdf.
13. World Health Organization. WHO child growth standards: length/height-for-age, weight-for-age, weight-for-length, weight-for-height and body mass index-for-age: methods and development. 2006.
14. Breymann C, Milman N, Mezzacasa A, Bernard R, Dudenhausen J, investigators F-A. Ferric carboxymaltose vs. oral iron in the treatment of pregnant women with iron deficiency anemia: an international, open-label, randomized controlled trial (FER-ASAP). J Perinat Med. 2017;45(4):443-53. Epub 2016/06/10. doi: 10.1515/jpm-2016-0050. PubMed PMID: 27278921.
15. Mwangi MN, Roth JM, Smit MR, Trijsburg L, Mwangi AM, Demir AY, et al. Effect of Daily Antenatal Iron Supplementation on Plasmodium Infection in Kenyan Women: A Randomized Clinical Trial. JAMA. 2015;314(10):1009-20. doi: 10.1001/jama.2015.9496.
16. Mehta CR, Pocock SJ. Adaptive increase in sample size when interim results are promising: a practical guide with examples. Stat Med. 2011;30(28):3267-84. Epub 20101130. doi: 10.1002/sim.4102. PubMed PMID: 22105690.
17. Yelland LN, Sullivan TR, Voysey M, Lee KJ, Cook JA, Forbes AB. Applying the intention-to-treat principle in practice: Guidance on handling randomisation errors. Clin Trials. 2015;12(4):418-23. Epub 20150601. doi: 10.1177/1740774515588097. PubMed PMID: 26033877; PubMed Central PMCID: PMC4509880.
18. Huque MH, Carlin JB, Simpson JA, Lee KJ. A comparison of multiple imputation methods for missing data in longitudinal studies. BMC Med Res Methodol. 2018;18(1):168. Epub 2018/12/14. doi: 10.1186/s12874-018-0615-6. PubMed PMID: 30541455; PubMed Central PMCID: PMC6292063.
19. Rezvan PH, Lee K, Simpson JA. Sensitivity analysis within multiple imputation framework using delta-adjustment: Application to Longitudinal Study of Australian Children. Longitudinal and life course studies. 2018;9:259-78.
20. Sullivan TR, Yelland LN, Moreno-Betancur M, Lee KJ. Multiple imputation for handling missing outcome data in randomized trials involving a mixture of independent and paired data. Stat Med. 2021;40(27):6008-20. Epub 20210815. doi: 10.1002/sim.9166. PubMed PMID: 34396577.
21. Holm S. A Simple Sequentially Rejective Multiple Test Procedure. Scandinavian Journal of Statistics. 1979;6(2):65-70.
22. International Council for Harmonisation of Technical Requirements for Pharmaceuticals for Human Use (ICH). Addendum on Estimands and Sensitivity Analysis in Clinical Trials to the Guideline on Statistical Principles for Clinical Trials. 2019 20 November 2019. Report No.

23. Williamson T, Eliasziw M, Fick GH. Log-binomial models: exploring failed convergence. Emerging Themes in Epidemiology. 2013;10(1):14. doi: 10.1186/1742-7622-10-14.
24. Altman DG. Confidence intervals for the number needed to treat. Bmj. 1998;317(7168):1309-12. doi: 10.1136/bmj.317.7168.1309. PubMed PMID: 9804726; PubMed Central PMCID: PMC1114210.
25. Liang K-Y, Zeger SL. Longitudinal data analysis of continuous and discrete responses for pre-post designs. Sankhyā: The Indian Journal of Statistics, Series B. 2000:134-48.
26. Yelland LN, Salter AB, Ryan P, Makrides M. Analysis of binary outcomes from randomised trials including multiple births: when should clustering be taken into account? Paediatr Perinat Epidemiol. 2011;25(3):283-97. doi: 10.1111/j.1365-3016.2011.01196.x. PubMed PMID: 21470268.
27. Koyfman A. Hypophosphatemia in Emergency Medicine. MedScape. 2021.
28. Yu AS, JR S. Hypophosphatemia: Clinical manifestations of phosphate depletion. UpToDate2022.

14 Listing of Key Tables, Listings and Figures

Number	Title	Population
Table 1	Characteristics at enrolment	ITT population – mothers
Table 2	Maternal outcomes	ITT population – mothers
Table 3	Neonate/infant outcomes	ITT population – neonates/infants
Table 4	Maternal adverse events	Safety population – mothers
Table 5	Infusion-related adverse events	Safety population – mothers
Table 6	Maternal safety biomarkers	Safety population – mothers
Table 7	Neonate/Infant adverse events	Safety population –neonates/infants
~~Table 8~~	~~Neonate safety biomarkers~~	~~Safety population –neonates/infants~~
Figure 1	CONSORT flow chart	All screened mothers
Figure 2	Estimate and 95% confidence interval of haemoglobin and anaemia over time	ITT population – mothers
Figure(s) 3a	Forest plots for subgroup analyses of maternal outcomes	ITT population – mothers
Figure(s) 3b	Forest plots for subgroup analyses of neonate/infant outcomes	ITT population – neonates/infants

Table 1 – Maternal characteristics at Enrolment *

	IV Iron<br>N = XXX	Standard of care – Oral Iron<br>N = XXX
Site		
Likangala	n (%)	n (%)
Bimbi	n (%)	n (%)
Lambulira	n (%)	n (%)
Domasi	n (%)	n (%)
Naisi	n (%)	n (%)
Matawale	n (%)	n (%)
City clinic	n (%)	n (%)
Sadzi	n (%)	n (%)
Age (years)	X.X ± X.X	X.X ± X.X
Primigravid, no. (%)‡	n (%)	n (%)
Gestational age (weeks), median (IQR)**	X.X (X.X – X.X)	X.X (X.X – X.X)
Height (cm)	X.X ± X.X	X.X ± X.X
Weight (kg)	X.X ± X.X	X.X ± X.X
Body mass index (kg/m^2)†	X.X ± X.X	X.X ± X.X
Religion, no. (%)‡		
None	n (%)	n (%)
Christian	n (%)	n (%)
Muslim	n (%)	n (%)
Other	n (%)	n (%)
Education, no. (%)‡		
None	n (%)	n (%)
Lower Primary (1-5)	n (%)	n (%)
Upper Primary (6-8)	n (%)	n (%)
Lower Secondary (1-2)	n (%)	n (%)
Upper Secondary (3-4)	n (%)	n (%)
Tertiary	n (%)	n (%)
Marital status, no. (%)‡		
Single	n (%)	n (%)
Married	n (%)	n (%)
Widowed	n (%)	n (%)
Divorced/Separated	n (%)	n (%)
Other	n (%)	n (%)
Income source, no. (%)‡		
None	n (%)	n (%)
Subsistence farming	n (%)	n (%)
Large scale farming	n (%)	n (%)
Employed	n (%)	n (%)
Casual work for wages	n (%)	n (%)

Business	n (%)	n (%)
Other	n (%)	n (%)
HIV positive, no. (%)‡	n (%)	n (%)
Malaria RDT positive, no. (%)	n (%)	n (%)
Capillary Hb<10 g/dL, no. (%)	n (%)	n (%)
Venous Hb, mean (SD)	X.X ± X.X	X.X ± X.X
Anaemia (venous), no. (%)§		
Mild	n (%)	n (%)
Moderate	n (%)	n (%)
Severe	n (%)	n (%)
Ferritin, median (IQR)	X.X (X.X – X.X)	X.X (X.X – X.X)
C-reactive protein, median (IQR)	X.X (X.X – X.X)	X.X (X.X – X.X)
Iron deficient, no. (%)¶	n (%)	n (%)
Iron deficient anaemia, no. (%)¶	n (%)	n (%)
Inflammation, no. (%) ||	n (%)	n (%)
Anaemia and inflammation, no. (%) ||	n (%)	n (%)

*Plus-minus values are means ±SD.
Hb denotes haemoglobin, HIV denotes human immunodeficiency virus, IQR inter-quartile range (25th to 75th percentile), and SD standard deviation.
** Estimated gestational age (weeks) is dated either using Last Menstrual Period or fundal height.
† Body mass index is the weight in kilograms divided by the square of the height in meters.
‡ Religion, Education, Marital status, Income source, parity, gravidity and HIV status were self-reported.
§ Mild anaemia indicates 10g/dL>Hb<11g/dL, moderate anaemia indicates 7g/dL≤Hb<10g/dL, and severe anaemia indicates Hb<7g/dL.
¶ Iron deficient indicates serum ferritin<15 ug/L or ferritin <30 ug/L if C-reactive protein >5 μg/L, and iron deficient anaemia indicates Hb<11g/dL and serum ferritin<15 ug/L or ferritin <30 ug/L if C-reactive protein >5 μg/L
|| Inflammation indicates C-reactive protein >5μg/L, and anaemia and inflammation indicates Hb<11.0g.dL and C-reactive protein >5μg/L

Table 2 – Primary and Secondary Maternal endpoints*

	IV Iron N = XXX	Standard of care – Oral Iron N = XXX	Prevalence Ratio or Mean Difference or Geometric Mean Ratio (95% CI) †	P-value‡
**Primary maternal outcome**				
Anaemia at 36 weeks' gestation or delivery (whichever comes first) §	n (%)	n (%)	X.X (X.X – X.X)	X.XXX
**Key secondary maternal outcomes**				
Venous Hb (g/dL) change from baseline				
36 weeks' gestation or delivery (whichever comes first)	X.X ± X.X	X.X ± X.X	X.X (X.X – X.X)	X.XXX**
1 month postpartum	X.X ± X.X	X.X ± X.X	X.X (X.X – X.X)	X.XXX**
Ferritin (μg/L) change from baseline				
36 weeks' gestation or delivery (whichever comes first)	X.X (X.X – X.X)	X.X (X.X – X.X)	X.X (X.X – X.X)	X.XXX**
1 month postpartum	X.X (X.X – X.X)	X.X (X.X – X.X)	X.X (X.X – X.X)	X.XXX**
**Other secondary maternal outcomes**				
Anaemia §				
Delivery	n (%)	n (%)	X.X (X.X – X.X)	
1 month postpartum	n (%)	n (%)	X.X (X.X – X.X)	
Moderate/Severe anaemia §				
36 weeks' gestation or delivery (whichever comes first)	n (%)	n (%)	X.X (X.X – X.X)	
Delivery	n (%)	n (%)	X.X (X.X – X.X)	
1 month postpartum	n (%)	n (%)	X.X (X.X – X.X)	
Venous Hb (g/dL) change from baseline				
Delivery	X.X ± X.X	X.X ± X.X	X.X (X.X – X.X)	

Ferritin (μg/L) change from baseline to baseline				
Delivery	X.X (X.X – X.X)	X.X (X.X – X.X)	X.X (X.X – X.X)	
Iron deficiency ¶				
36 weeks' gestation or delivery (whichever comes first)	n (%)	n (%)	X.X (X.X – X.X)	
Delivery	n (%)	n (%)	X.X (X.X – X.X)	
1 month postpartum	n (%)	n (%)	X.X (X.X – X.X)	
Iron deficient anaemia ¶				
36 weeks' gestation or delivery (whichever comes first)	n (%)	n (%)	X.X (X.X – X.X)	
Delivery	n (%)	n (%)	X.X (X.X – X.X)	
1 month postpartum	n (%)	n (%)	X.X (X.X – X.X)	

* Plus-minus values are means ±SD or median. Skewed values are presented as median(IQR). CI denotes confidence interval, ID iron deficiency, IDA iron deficiency anaemia, IQR, interquartile range (25th to 75th Percentile) and SD, standard deviation.

§ Anaemia indicates Hb<11.0 g/dL up to and including delivery and Hb<12.0 g/dL postpartum. Moderate/Severe anaemia indicates Hb<10g/dL up to and including delivery and Hb<11.0 g/dL postpartum.

¶ Iron deficient indicates serum ferritin<15 ug/L or ferritin <30 ug/L if C-reactive protein >5 μg/L, and iron deficient anaemia indicates Hb<11g/dL and serum ferritin<15 ug/L or ferritin <30 ug/L if C-reactive protein >5 μg/L

† A prevalence ratio of IV iron versus standard-of-care (oral iron) is displayed for anaemia, ID and IDA and risk ratio for moderate/severe anaemia at 36 weeks gestation or delivery (whichever comes first), delivery, and 28 days postpartum following analyses using a <final model to be specified after database unblinding> model, including random effect for participant. A mean difference of IV iron versus standard-of-care (oral iron) of the estimated change from baseline to at 36 weeks gestation or delivery (whichever comes first), delivery, and 28 days postpartum respectively is displayed for continuous haemoglobin and ferritin concentration following analyses using a likelihood-based longitudinal data analysis model. A geometric mean ratio is displayed for ferritin concentration after a log base e transformation due to skewness.

‡ The P-values and 95% confidence intervals presented have not been adjusted for multiple comparisons.

** The P-value for X and Y remained significant after controlling for multiple comparisons with the Holm procedure.

Table 3 – Primary and Secondary Neonate endpoints*

	IV Iron Number (percent)	IV Iron Mean ± SD	Standard of care – Oral Iron Number (percent)	Standard of care – Oral Iron Mean ± SD	Prevalence Ratio or Mean Difference or Geometric Mean Ratio (95% CI) †	P-value‡
**Key neonatal outcome**						
Birth weight (grams)	n	X.X ± X.X	n	X.X ± X.X	X.X (X.X – X.X)	X.XXX
**Secondary neonatal outcomes**						
Venous Hb (g/dL) at 1 month of age	n	X.X ± X.X	n	X.X ± X.X	X.X (X.X – X.X)	X.XXX**
~~Ferritin (µg/L) at 1 month of age~~	~~n~~	~~X.X (X.X – X.X)~~	~~n~~	~~X.X (X.X – X.X)~~	~~X.X (X.X – X.X)~~	~~X.XXX**~~
Weight (grams) at 1 month of age	n	X.X ± X.X	n	X.X ± X.X	X.X (X.X – X.X)	X.XXX**
**Other neonatal outcomes**						
Birth length (cm)	n	X.X ± X.X	n	X.X ± X.X	X.X (X.X – X.X)	
Low birth weight (<2500g) ‡‡	n (%)	N/A	n (%)	N/A	X.X (X.X – X.X)	
Stillbirth	n (%)	N/A	n (%)	N/A	X.X (X.X – X.X)	
Length (cm) at one month of age §	n	X.X ± X.X	n	X.X ± X.X	X.X (X.X – X.X)	
Weight for age z score at one month of age	n	X.X ± X.X	n	X.X ± X.X	X.X (X.X – X.X)	
Length for age z score at one month of age	n	X.X ± X.X	n	X.X ± X.X	X.X (X.X – X.X)	
Weight for length z score	n	X.X ± X.X	n	X.X ± X.X	X.X (X.X – X.X)	

at one month of age						

* Plus-minus values are means ±SD. Skewed values are presented as median(IQR). CI denotes confidence interval, IQR, interquartile range (25th to 75th Percentile) and SD, standard deviation.

† An absolute mean difference for birth weight, and birth length at delivery, and haemoglobin concentration~~, ferritin concentration~~, length-for-age z-score, weight-for-age z-score, and weight-for-length z-score at 1 month of age between IV iron and standard-of-care (oral iron) is displayed following fitting a linear regression model. ~~A geometric mean ratio is displayed for ferritin concentration at one month of age after a log base e transformation due to skewness.~~ A risk ratio of IV iron versus standard of care is displayed for low birth weight, and stillbirth following analyses using a <final model to be specified after database unblinding> regression model.

‡ The P-values and 95% confidence intervals presented have not been adjusted for multiple comparisons.

** The P-value for X and Y remained significant after controlling for multiple comparisons with the Holm procedure.

Table 4 – Maternal Adverse events *

	IV Iron N = XXX	Standard of care – Oral Iron N = XXX	Risk Ratio (95% CI)†	P-value‡
**Summary of subjects with AEs**				
At least one AE	n (%)	n (%)	X.X (X.X – X.X)	X.XXX
At least one SAE	n (%)	n (%)	X.X (X.X – X.X)	X.XXX
Death	n (%)	n (%)		
**Summary of number of AEs**				
AE	n	n		N/A
SAE	n	n		N/A
**Subjects with at least one AE of special interest**				
Composite Severe medical event§	n (%)	n (%)	X.X (X.X – X.X)	X.XXX
Death	n (%)	n (%)	X.X (X.X – X.X)	X.XXX
Haemorrhage	n (%)	n (%)	X.X (X.X – X.X)	X.XXX
Blood transfusion¶	n (%)	n (%)	X.X (X.X – X.X)	X.XXX
ICU care ¶	n (%)	n (%)	X.X (X.X – X.X)	X.XXX
Clinical Malaria (by PT)	n (%)	n (%)	X.X (X.X – X.X)	X.XXX
**Subjects with common AEs occurring in >5% in any treatment group**				
E.g. Headache	n (%)	n (%)	X.X (X.X – X.X)	X.XXX
<data driven>				
…				
**Subjects with at least one AE by SOC**				
Infections and infestations	n (%)	n (%)	X.X (X.X – X.X)	X.XXX
Congenital, familial and genetic disorders	n (%)	n (%)	X.X (X.X – X.X)	X.XXX
Nervous system disorders	n (%)	n (%)	X.X (X.X – X.X)	X.XXX
Musculoskeletal and connective tissue disorders	n (%)	n (%)	X.X (X.X – X.X)	X.XXX
Vascular disorders	n (%)	n (%)	X.X (X.X – X.X)	X.XXX
Respiratory, thoracic and mediastinal disorders	n (%)	n (%)	X.X (X.X – X.X)	X.XXX
Gastrointestinal disorders	n (%)	n (%)	X.X (X.X – X.X)	X.XXX
Pregnancy, puerperium and perinatal conditions	n (%)	n (%)	X.X (X.X – X.X)	X.XXX
Ear and labyrinth disorders	n (%)	n (%)	X.X (X.X – X.X)	X.XXX
Blood and lymphatic disorders	n (%)	n (%)	X.X (X.X – X.X)	X.XXX
Skin and subcutaneous disorders	n (%)	n (%)	X.X (X.X – X.X)	X.XXX

	IV Iron N = XXX	Standard of care – Oral Iron N = XXX	Risk Ratio (95% CI)†	P-value‡
General disorders and administration site conditions	n (%)	n (%)	X.X (X.X – X.X)	X.XXX
Cardiac disorders	n (%)	n (%)	X.X (X.X – X.X)	X.XXX
Neoplasms benign, malignant and unspecified (incl cysts and polyps)	n (%)	n (%)	X.X (X.X – X.X)	X.XXX
Injury, poisoning and procedural complications	n (%)	n (%)	X.X (X.X – X.X)	X.XXX
Reproductive system and breast disorders	n (%)	n (%)	X.X (X.X – X.X)	X.XXX
…				
**Subjects with at least one AE by SOC preferred term**				
E.g. Headache	n (%)	n (%)	X.X (X.X – X.X)	X.XXX
….				

*CI denotes confidence interval, AE Adverse Event, SAE Serious Adverse Event, SOC System Organ Class, ICU Intensive Care Unit, PT Preferred Term.
†Except for number of AE/SAE, these outcomes are analysed using a <final model to be specified after database unblinding> regression model and display the estimate of the risk ratio of IV iron versus standard-of-care (oral iron) and 95% CI and P-Value.
‡ Unadjusted P-values and 95% confidence intervals are presented here.
§ The composite severe medical event outcome was at least one severe medical event of death, haemorrhage, blood transfusion or ICU care recorded in the mother. Death, haemorrhage, blood transfusion and admission to ICU are captured across the whole trial period.
¶Blood transfusion denotes that a blood transfusion was required by the mother and ICU care denotes that ICU care was required by the mother.

Table 5 – Maternal Infusion related Adverse events *

	IV Iron<br>N = XXX
**Summary of number of infusion related AEs**	
At least one Infusion related AE	n (%)
**Summary of infusion related AEs † §**	
Headache	n (%)
Dizziness	n (%)
Discolouration of the skin	n (%)
Nausea	n (%)
Vomiting	n (%)
Upper abdominal pain	n (%)
Dyspepsia	n (%)
Anaphylactic shock	n (%)
Flushing	n (%)
Shortness of breath	n (%)
Chest pains	n (%)
Other	n (%)
None	n (%)

*reported for IV iron group only. AE denotes Adverse Event
†AEs as captured on the participant randomisation form at the time IV iron given
§Events sorted according to frequency from high to low.

Table 6 – Maternal Safety Biomarkers

	IV Iron N = XXX	Standard of care – Oral Iron N = XXX	Risk Ratio (95% CI)	P-value‡
**Hypophosphatemia** *†				
36 weeks' gestation or delivery (whichever comes first)				
Any	n (%)	n (%)	X.X (X.X – X.X)	X.XXX
Mild	n (%)	n (%)	–	–
Moderate	n (%)	n (%)	–	–
Severe	n (%)	n (%)	–	–
Delivery				
Any	n (%)	n (%)	X.X (X.X – X.X)	X.XXX
Mild	n (%)	n (%)	–	–
Moderate	n (%)	n (%)	–	–
Severe	n (%)	n (%)	–	–
1 month postpartum				
Any	n (%)	n (%)	X.X (X.X – X.X)	X.XXX
Mild	n (%)	n (%)	–	–
Moderate	n (%)	n (%)	–	–
Severe	n (%)	n (%)	–	–
**Inflammation (elevated CRP)** †				
36 weeks' gestation or delivery (whichever comes first)	n (%)	n (%)	X.X (X.X – X.X)	X.XXX
Delivery	n (%)	n (%)	X.X (X.X – X.X)	X.XXX
1 month postpartum	n (%)	n (%)	X.X (X.X – X.X)	X.XXX

CI denotes confidence interval, CRP C-reactive protein.
* Mild hypophosphatemia is defined as (0.64<PO4 <0.80 mmol/L), Moderate hypophosphatemia is defined as (0.32<PO4<0.64 mmol/L) and severe hypophosphatemia is defined as (PO4 (<0.32 mmol/L).
†These outcomes are analysed by time point (36 weeks' gestation or delivery (whichever comes first), delivery, and 1 month postpartum) using a <final model to be specified after database unblinding>regression model and display the estimate of the risk ratio of IV iron versus standard-of-care (oral iron) and 95% CI and P-Value.
‡ Unadjusted P-values and 95% confidence intervals are presented here.

Table 7 – Neonate Adverse events*

	IV Iron N = XXX	Standard of care – Oral Iron N = XXX	Risk Ratio (95% CI)†	P-value‡
**Summary of subjects AEs**				
At least one AE	n (%)	n (%)	X.X (X.X – X.X)	X.XXX
At least one SAE	n (%)	n (%)	X.X (X.X – X.X)	X.XXX
Death	n (%)	n (%)	X.X (X.X – X.X)	X.XXX
Death & stillbirth	n (%)	n (%)	X.X (X.X – X.X)	X.XXX
**Summary of number of AEs**				
AE	n	n		N/A
SAE	n	n		N/A
**Subjects with at least one AE of special interest**				
Clinical Malaria (by PT)	n (%)	n (%)	X.X (X.X – X.X)	X.XXX
**Subjects with common AEs occurring in >5% in any treatment group**				
E.g. Infection	n (%)	n (%)	X.X (X.X – X.X)	X.XXX
<data driven>				
**Subjects with at least one AE by SOC**				
General disorders and administration site conditions	n (%)	n (%)	X.X (X.X – X.X)	X.XXX
Pregnancy, puerperium and perinatal conditions	n (%)	n (%)	X.X (X.X – X.X)	X.XXX
Congenital, familial and genetic disorders	n (%)	n (%)	X.X (X.X – X.X)	X.XXX
Infections and Infestations	n (%)	n (%)	X.X (X.X – X.X)	X.XXX
Respiratory, thoracic and mediastinal disorders	n (%)	n (%)	X.X (X.X – X.X)	X.XXX
…				
**Subjects with at least one AE by preferred term**				
E.g Fever	n (%)	n (%)	X.X (X.X – X.X)	X.XXX
….				

*CI denotes confidence interval, AE Adverse Event, SAE Serious Adverse Event, SOC System Organ Class, PT Preferred Term

†Except for number of AE/SAE, these outcomes are analysed using a <final model to be specified after database unblinding>regression model and display the estimate of the risk ratio of IV iron versus standard-of-care (oral iron) and 95% CI and P-Value.

‡ Unadjusted P-values and 95% confidence intervals are presented here.

Table 8 – Neonate Safety Biomarkers at one month of age

	IV Iron N = XXX	Standard of care – Oral Iron N = XXX	Risk Ratio (95% CI)	P-value‡
**Hypophosphatemia *†**				
Any	n (%)	n (%)	X.X (X.X – X.X)	X.XXX
Mild	n (%)	n (%)	–	–
Moderate	n (%)	n (%)	–	–
Severe	n (%)	n (%)	–	–

CI denotes confidence interval, CRP C-reactive protein
* Mild hypophosphatemia is defined as (0.64<PO4 <0.80 mmol/L), Moderate hypophosphatemia is defined as (0.32<PO4<0.64 mmol/L) and severe hypophosphatemia is defined as (PO4 (<0.32 mmol/L).
†These outcomes are analysed using a <final model to be specified after database unblinding>regression model and display the estimate of the risk ratio of IV iron versus standard-of-care (oral iron) and 95% CI and P-Value.
‡ Unadjusted P-values and 95% confidence intervals are presented here.

Figure 1 – CONSORT flow chart

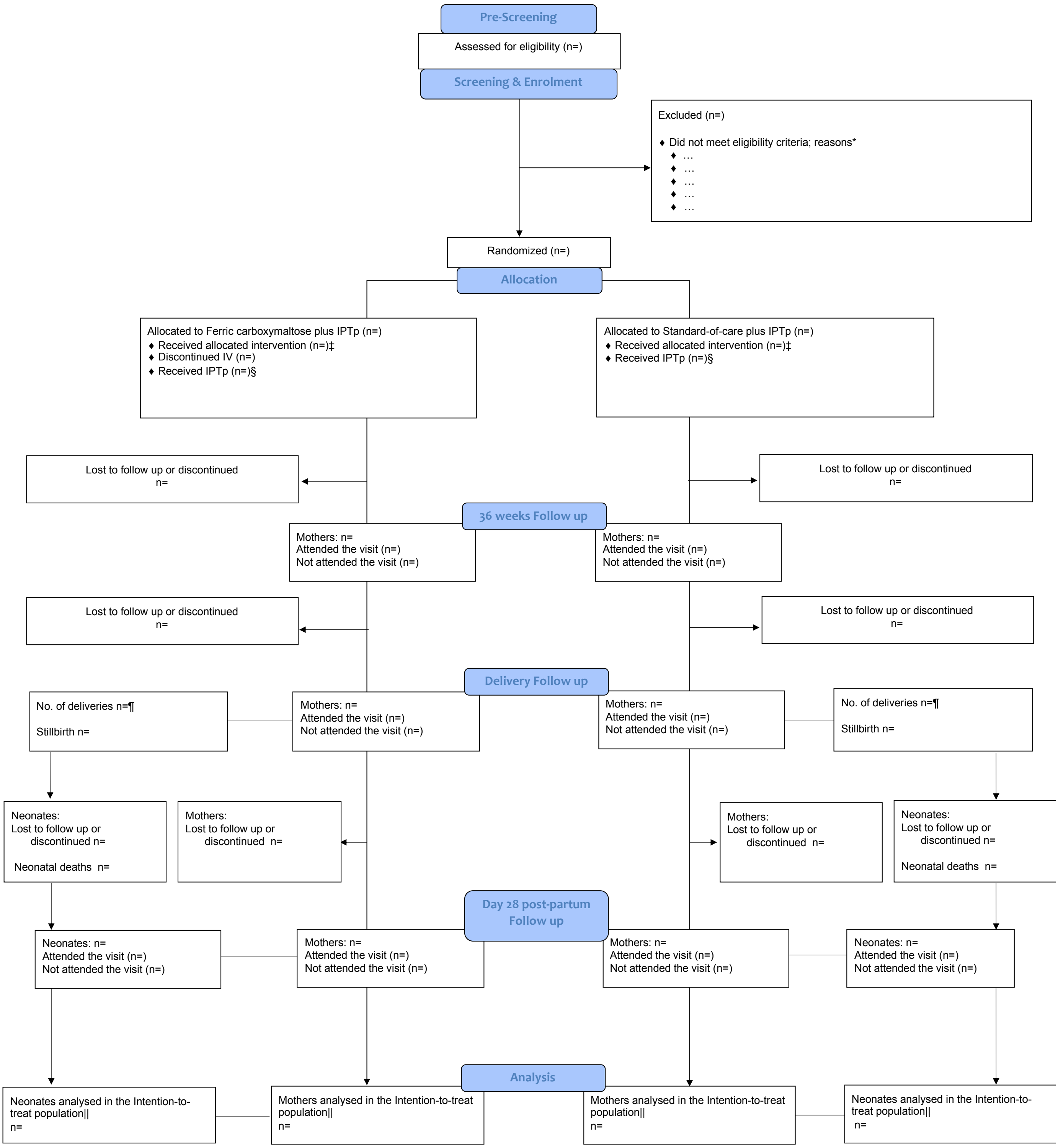

*Reasons for not meeting eligibility were assessed on the questions on the eligibility data collection forms
†Defined as those who answered No to the question "Accepts study procedures?" at enrolment.
‡Reasons for not receiving the treatment were collected on the participant randomisation form.
§ Defined as those who received Intermittent Preventive Treatment in pregnancy (IPTp) with suldadoxine pyrimethamine (SP) at enrolment
¶ there were X twins born in the IV iron group and X in the standard-of-care oral iron group
|| The Intention-to-treat basis indicates maternal and neonatal outcomes were analyzed according to randomly allocated group of the woman. One woman who was randomised but later found to be not pregnant was excluded from all analyses
