# Supplementary material for: Ferric carboxymaltose for anemia in late pregnancy: a randomized controlled trial
Source: Nat Med. 2025 Jan 6;31(1):197–206. doi: 10.1038/s41591-024-03385-w (PMC11750709; doi:10.1038/s41591-024-03385-w)
Supplement: Supplementary file 2 — Reporting Summary [file 41591_2024_3385_MOESM2_ESM.pdf]

nature portfolio

Corresponding author(s): Sant-Rayn Pasricha

Last updated by author(s): Oct 11, 2024

Reporting Summary

Nature Portfolio wishes to improve the reproducibility of the work that we publish. This form provides structure for consistency and transparency in reporting. For further information on Nature Portfolio policies, see our Editorial Policies and the Editorial Policy Checklist.

Statistics

For all statistical analyses, confirm that the following items are present in the figure legend, table legend, main text, or Methods section.

n/a	Confirmed	
☐	☒	The exact sample size (*n*) for each experimental group/condition, given as a discrete number and unit of measurement
☒	☐	A statement on whether measurements were taken from distinct samples or whether the same sample was measured repeatedly
☐	☒	The statistical test(s) used AND whether they are one- or two-sided<br>*Only common tests should be described solely by name; describe more complex techniques in the Methods section.*
☐	☒	A description of all covariates tested
☐	☒	A description of any assumptions or corrections, such as tests of normality and adjustment for multiple comparisons
☐	☒	A full description of the statistical parameters including central tendency (e.g. means) or other basic estimates (e.g. regression coefficient) AND variation (e.g. standard deviation) or associated estimates of uncertainty (e.g. confidence intervals)
☐	☒	For null hypothesis testing, the test statistic (e.g. *F*, *t*, *r*) with confidence intervals, effect sizes, degrees of freedom and *P* value noted<br>*Give P values as exact values whenever suitable.*
☒	☐	For Bayesian analysis, information on the choice of priors and Markov chain Monte Carlo settings
☐	☒	For hierarchical and complex designs, identification of the appropriate level for tests and full reporting of outcomes
☐	☒	Estimates of effect sizes (e.g. Cohen's *d*, Pearson's *r*), indicating how they were calculated

Our web collection on statistics for biologists contains articles on many of the points above.

Software and code

Policy information about availability of computer code

Data collection	Data were recorded in digital form with REDCap, using electronic tablets, and backed up daily to a local backup server at TRUE, Blantyre, Malawi, with a de-identified fortnightly backup to WEHI, Melbourne, Australia.
Data analysis	Data were analysed using Stata SE, version 18.0 (StataCorp, College Station, TX: StataCorp LLC.

For manuscripts utilizing custom algorithms or software that are central to the research but not yet described in published literature, software must be made available to editors and reviewers. We strongly encourage code deposition in a community repository (e.g. GitHub). See the Nature Portfolio guidelines for submitting code & software for further information.

Data

Policy information about availability of data

All manuscripts must include a data availability statement. This statement should provide the following information, where applicable:

- Accession codes, unique identifiers, or web links for publicly available datasets
- A description of any restrictions on data availability
- For clinical datasets or third party data, please ensure that the statement adheres to our policy

Underlying deidentified individual participant data encompassing the reported trial results and a data dictionary are accessible at figshare (https://doi.org/10.26188/26968171.v1). Data are available under the terms of Creative Commons Attribution 4.0 International License (CC-BY-4.0).

Research involving human participants, their data, or biological material

Policy information about studies with human participants or human data. See also policy information about sex, gender (identity/presentation), and sexual orientation and race, ethnicity and racism.

Reporting on sex and gender	All participants in the trial were pregnant women.
Reporting on race, ethnicity, or other socially relevant groupings	All participants in the trial were locals to the Zomba region of Southern Malawi. We have not elaborated on race/ ancestry any further.
Population characteristics	Study participants were eligible for the trial if they were female, had a confirmed pregnancy 27 to 35-weeks' gestation based on either last menstrual period or fundal height (ultrasound based gestational-age screening is not effective in late pregnancy44); a capillary haemoglobin concentration less than 10g/dL (moderate or severe anaemia) measured by HemoCue 301+ (Angelholm, Sweden), a negative malaria rapid diagnostic test, were afebrile, and were expecting to reside and deliver their baby within the Zomba study site catchment area. We utilised capillary haemoglobin measurement but not iron parameters as we judged that screening large populations of women for anaemia in pregnancy using venous blood and iron biomarkers is not presently feasible and that applying these parameters in inclusion criteria would limit the applicability of our findings. Women were excluded if they had been previously or were currently enrolled in another trial (including the REVAMP trial), had a known hypersensitivity to any study drug, exhibited clinical symptoms of malaria or other infection, had a known history of sickle cell or sickle-haemoglobin C anaemia, were clinically unstable with a low haemoglobin level requiring a blood transfusion (usually haemoglobin <5g/dL), or had evidence of pre-eclampsia. HIV positivity was not an exclusion criterion.
Recruitment	Women attending one of eight primary antenatal clinics in Zomba district (Likangala, Bimbi, Lambulira, Domasi, Naisi, Matawale, Sadzi and City) were screened for eligibility during their routine antenatal visit. Consent was received according to the study protocol. All women who attended for antenatal care were eligible for screening ensuring the screening population reflected the overall population.
Ethics oversight	The trial was approved by ethics committees in Malawi (National Health Sciences Research Committee of Malawi Approval – NHSRC 20/11/2622) and Australia (Human Research Ethics, WEHI 20/25).

Note that full information on the approval of the study protocol must also be provided in the manuscript.

Field-specific reporting

Please select the one below that is the best fit for your research. If you are not sure, read the appropriate sections before making your selection.

☒ Life sciences ☐ Behavioural & social sciences ☐ Ecological, evolutionary & environmental sciences

For a reference copy of the document with all sections, see nature.com/documents/nr-reporting-summary-flat.pdf

Life sciences study design

All studies must disclose on these points even when the disclosure is negative.

Sample size	The sample size was 590 women (295 women per group) when accounting for a 10% drop out at the primary outcome. We planned to detect a 14% absolute reduction in anaemia prevalence by FCM compared with SOC with 90% power (two-sided alpha of 5%). This sample size also had 72% to 97% power to detect a 100g to 150g absolute difference in birthweight between FCM and SOC, assuming a standard deviation (SD) of 450g7 (two-sided alpha of 5%).
Data exclusions	Data analysis of maternal and neonatal outcomes use all available data. In addition, the outcomes of birth weight, low birth weight, birth length were multiply imputed before analysis. Details on handling of missing data are included in the Statistical Analysis Plan included with the supplemental materials. Available data were not otherwise excluded from analyses.
Replication	All clinical data was analysed once and all biological samples were analysed once, and the trial as a whole was conducted once, befitting a single large randomised controlled trial. It was not feasible to undertake the trial more than once.
Randomization	A randomisation schedule of randomly permuted blocks of size 4 or 6, stratified by site was used to randomly allocate participants 1:1 to one of the two treatment groups within site. The randomisation list was computer-generated by an independent statistician. Allocation codes were contained in sealed, opaque envelopes to randomly assign participants.
Blinding	The trial was open-label. As trial treatments were delivered in primary care centres, it was judged unfeasible to provide placebo infusions. Laboratory scientists measuring haemoglobin concentration, midwives collecting birth outcome data, data managers, and investigators and statisticians at WEHI (Australia) were all blinded to the treatment allocation until the database was locked for unblinding.

Reporting for specific materials, systems and methods

We require information from authors about some types of materials, experimental systems and methods used in many studies. Here, indicate whether each material, system or method listed is relevant to your study. If you are not sure if a list item applies to your research, read the appropriate section before selecting a response.

Materials & experimental systems

n/a	Involved in the study
☒	☐ Antibodies
☒	☐ Eukaryotic cell lines
☒	☐ Palaeontology and archaeology
☒	☐ Animals and other organisms
☐	☒ Clinical data
☒	☐ Dual use research of concern
☒	☐ Plants

Methods

n/a	Involved in the study
☒	☐ ChIP-seq
☒	☐ Flow cytometry
☒	☐ MRI-based neuroimaging

Clinical data

Policy information about clinical studies

All manuscripts should comply with the ICMJE guidelines for publication of clinical research and a completed CONSORT checklist must be included with all submissions.

Clinical trial registration	ANZCTR12621001239853
Study protocol	Included in supplemental appendix. Also published https://www.ncbi.nlm.nih.gov/pmc/articles/PMC10858019/
Data collection	The study was set in 8 antenatal health centres in rural Malawi. Recruitment occurred between 24 November 2021 and 22 February 2023. Haemoglobin was measured using a Sysmex automated analyser in Zomba. Birth weight was measured using calibrated Seca scales at each centre. Ferritin and CRP were measured using automated analysers at Meander Health Centre, Netherlands.
Outcomes	The primary outcome was anaemia (venous haemoglobin <11g/dL) at 36 weeks gestation or during delivery, whichever occurred first, enabling the trial to evaluate the impact of FCM on anaemia as a woman enters childbirth.<br><br>Maternal secondary outcomes included haemoglobin and ferritin concentrations, and anaemia, iron deficiency (defined by ferritin<15ug/L or ferritin<30ug/L if C-reactive protein>5mg/L), and iron-deficiency anaemia at 36 weeks gestation or during delivery, whichever occurred first, delivery, and at 1 month postpartum. Neonatal outcomes included birthweight and low birthweight (birthweight <2,500g) measured within 24 hours of delivery, and infant length-for-age, weight-for-age, and weight-for-length z-scores, as well as haemoglobin concentration at one-month of age.

Plants

Seed stocks	NA
Novel plant genotypes	NA
Authentication	NA
